# Discovery and Hit-to-Lead Optimization of Benzothiazole Scaffold-Based DNA Gyrase Inhibitors with Potent Activity against *Acinetobacter baumannii* and *Pseudomonas aeruginosa*

**DOI:** 10.1021/acs.jmedchem.2c01597

**Published:** 2023-01-12

**Authors:** Andrej Emanuel Cotman, Martina Durcik, Davide Benedetto Tiz, Federica Fulgheri, Daniela Secci, Maša Sterle, Štefan Možina, Žiga Skok, Nace Zidar, Anamarija Zega, Janez Ilaš, Lucija Peterlin Mašič, Tihomir Tomašič, Diarmaid Hughes, Douglas L. Huseby, Sha Cao, Linnéa Garoff, Talía Berruga Fernández, Paraskevi Giachou, Lisa Crone, Ivailo Simoff, Richard Svensson, Bryndis Birnir, Sergiy V. Korol, Zhe Jin, Francisca Vicente, Maria C. Ramos, Mercedes de la Cruz, Björn Glinghammar, Lena Lenhammar, Sara R. Henderson, Julia E. A. Mundy, Anthony Maxwell, Clare E. M. Stevenson, David M. Lawson, Guido V. Janssen, Geert Jan Sterk, Danijel Kikelj

**Affiliations:** † Faculty of Pharmacy, University of Ljubljana, Aškerčeva cesta 7, 1000 Ljubljana, Slovenia; ‡ Department of Medical Biochemistry and Microbiology, Uppsala University, Husargatan 3, 75123 Uppsala, Sweden; § Drug Optimization and Pharmaceutical Profiling Platform (UDOPP), Department of Pharmacy, Uppsala University, Husargatan 3, 75123 Uppsala, Sweden; ∥ Department of Medical Cell Biology, Uppsala University, Husargatan 3, 75123 Uppsala, Sweden; ⊥ Fundación MEDINA, Avenida del Conocimiento 34, Parque Tecnológico Ciencias de la Salud, 18016 Granada, Spain; # Department Chemical Process and Pharmaceutical Development, Unit Chemical and Pharmaceutical Safety, RISE Research Institutes of Sweden, 15136 Södertälje, Sweden; ∇ Department of Medical Sciences, Uppsala University Hospital, 75185 Uppsala, Sweden; ○ Department of Biochemistry and Metabolism, John Innes Centre, Norwich Research Park, Norwich NR4 7UH, U.K; ◆ Medicinal Chemistry Division, Vrije Universiteit Amsterdam, De Boelelaan 1108, 1081 HZ Amsterdam, The Netherlands

## Abstract

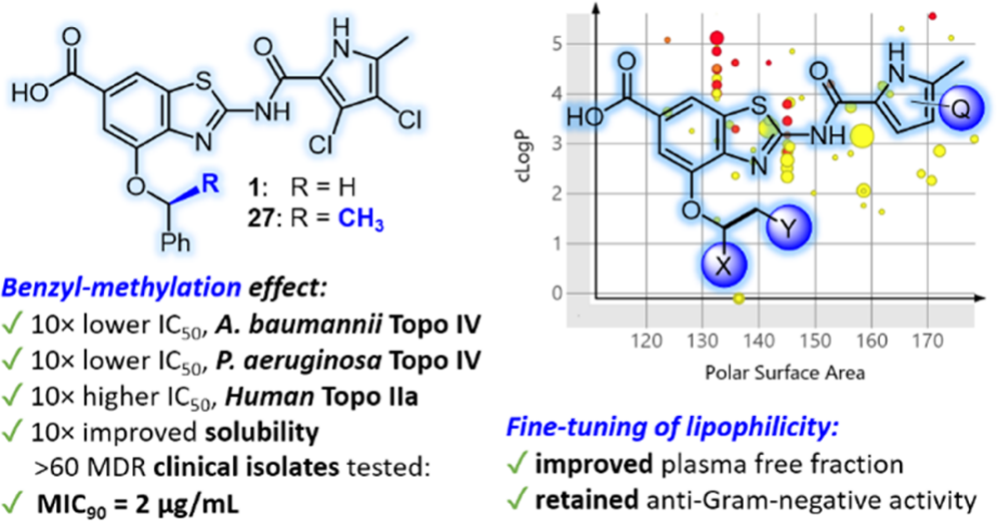

We have developed compounds with a promising activity against *Acinetobacter baumannii* and *Pseudomonas aeruginosa*, which are both on the WHO priority list of antibiotic-resistant bacteria. Starting from DNA gyrase inhibitor **1**, we identified compound **27**, featuring a 10-fold improved aqueous solubility, a 10-fold improved inhibition of topoisomerase IV from *A. baumannii* and *P. aeruginosa*, a 10-fold decreased inhibition of human topoisomerase IIα, and no cross-resistance to novobiocin. Cocrystal structures of **1** in complex with *Escherichia coli* GyrB24 and (*S*)-**27** in complex with *A. baumannii* GyrB23 and *P. aeruginosa* GyrB24 revealed their binding to the ATP-binding pocket of the GyrB subunit. In further optimization steps, solubility, plasma free fraction, and other ADME properties of **27** were improved by fine-tuning of lipophilicity. In particular, analogs of **27** with retained anti-Gram-negative activity and improved plasma free fraction were identified. The series was found to be nongenotoxic, nonmutagenic, devoid of mitochondrial toxicity, and possessed no ion channel liabilities.

## 1 Introduction

Carbapenem-resistant Gram-negative bacteria *Acinetobacter baumannii*, *Pseudomonas aeruginosa*, and *Enterobacteriaceae*, which include *Escherichia coli* and *Klebsiella pneumoniae*, are among the antibiotic-resistant bacteria on the WHO global priority list published in 2017 to guide research, discovery, and development of new antibiotics.^1^ These bacteria are all members of the ESKAPE group of six nosocomial pathogens, which also includes Gram-positive *Staphylococcus aureus* and *Enteroccocus faecium*, that exhibit multidrug resistance and high virulence. The increasing frequency of bacterial resistance against currently available antibiotics is a global problem that motivates the development of novel antibacterials with a distinct mode of action.^2−4^

Bacterial DNA gyrase and DNA topoisomerase IV (topo IV), both type II topoisomerases, responsible for the ATP-driven introduction of negative supercoils into DNA and ATP-driven supercoil relaxation and decatenation, respectively, are validated antibacterial targets.^5−7^ Gyrase is a heterotetramer of GyrA and GyrB subunits (A_2_B_2_), while topo IV is a heterotetramer of ParC and ParE subunits (C_2_E_2_). Whereas fluoroquinolones, which target both gyrase and topo IV, are widely used in the clinic, inhibitors interfering with ATP binding to GyrB/ParE subunits have not found significant clinical application, despite intensive research efforts during the last 70 years since the discovery of novobiocin, the first ATP-competitive GyrB inhibitor.^8^

**Figure 1:**
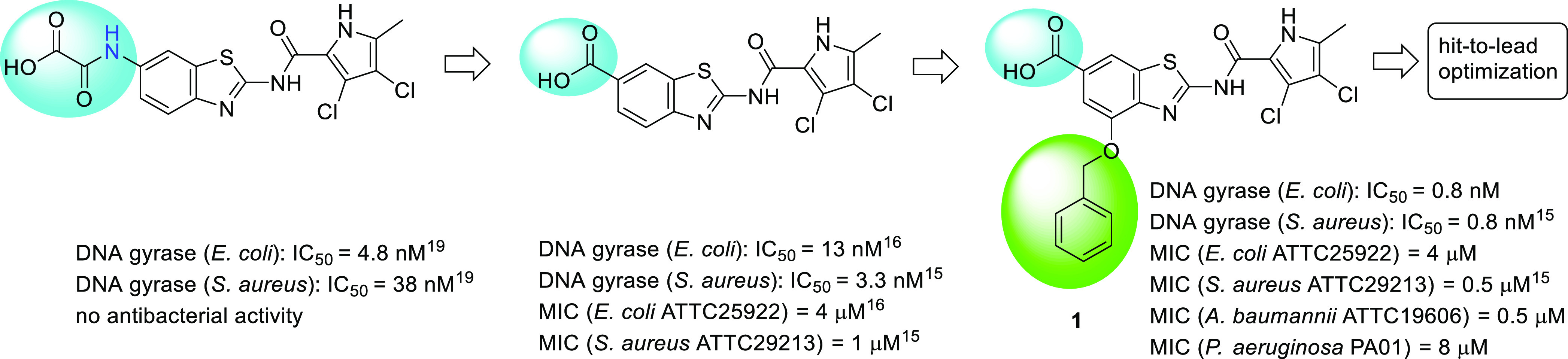
Evolution of a potent gyrase inhibitor 1 with antibacterial activity as a starting point for hit-to-lead optimization.

Having worked for several years on the development of benzothiazole scaffold-based GyrB inhibitors,^9−19^ the introduction of the carboxylic acid group to position 6 and attachment of a benzyloxy substituent to position 4 of the benzothiazole scaffold led us to the discovery of 4-(benzyloxy)-2-(3,4-dichloro-5-methyl-1*H*-pyrrole-2-carboxamido)benzo[*d*]thiazole-6-carboxylic acid (**1**, Figure 1), a potent gyrase inhibitor with excellent in vitro and in vivo activities, demonstrated in murine dermal and thigh infection models, against Gram-positive *S. aureus* as well as against methicillin-resistant- (MRSA) and vancomycin-resistant *S. aureus* (VISA).^15^ In this paper, we present the promising activity of compound **1** against the main Gram-negative ESKAPE pathogens *A. baumannii*, *P. aeruginosa*, and *Escherichia coli* and provide for it a solid biochemical rationale based on the inhibition of bacterial gyrase and topo IV. Further, we present our efforts to improve the ADMET properties of this frontrunner that resulted in the evolution of **1** to 2-(3,4-dichloro-5-methyl-1*H*-pyrrole-2-carboxamido)-4-(1-phenyl-ethoxy)benzo[*d*]thiazole-6-carboxylic acid (**27**) displaying improved activity against ESKAPE pathogens. Finally, the optimization of **27** in several directions to compounds with improved solubility, free fraction, and other ADMET properties is presented.

## 2 Results and Discussion

### 2.1 Frontrunner Compound **1** and Follow-up Compound **27**

**Figure 2:**
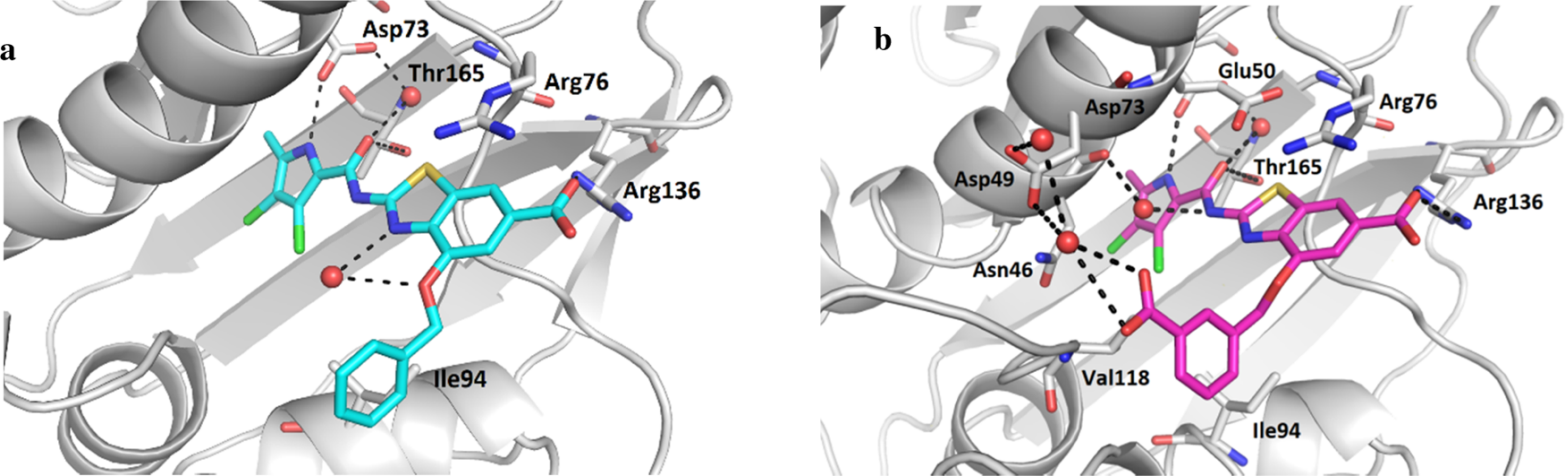
Cocrystal structures of (a) compound **1** (in cyan sticks, PDB 7P2M) and (b) compound **7** (in magenta sticks, PDB 7P2W) in complex with *E. coli* GyrB24 (in gray cartoon). For clarity, only amino acid residues that interact with ligands are shown as sticks. Water molecules are presented as red spheres, and hydrogen bonds are shown as dashed black lines.

Compound **1** is a potent inhibitor of DNA supercoiling catalyzed by *E. coli* DNA gyrase [IC_50_ = 0.8 nM (gel-based assay), IC_50_ < 10 nM (microtiter-plate-based assay)], *A. baumannii* DNA gyrase (IC_50_ < 10 nM), and *P. aeruginosa* DNA gyrase (IC_50_ < 10 nM) as well as of DNA decatenation induced by *E. coli* topo IV (IC_50_ = 352 nM), *A. baumannii* topo IV (IC_50_ = 64 nM), and *P. aeruginosa* topo IV (IC_50_ = 235 nM) and shows selectivity against a related target human topoisomerase IIα (IC_50_ = 1.95 μM). IC_50_ values for the inhibition of *E. coli* DNA gyrase-catalyzed supercoiling and topo IV-catalyzed relaxation of **1** measured in gel-based assays are consistent with those determined by high-throughput microtiter-plate-based assays. The compound does not stabilize the gyrase cleavage complex and does not show significant inhibition of ATP-independent relaxation (IC_50_ > 10 μM). A crystal structure of compound **1** in complex with *E. coli* GyrB24 (24 kDa amino-terminal subdomain of GyrB) obtained at a 1.16 Å resolution provides evidence of the inhibitor binding to the ATP-binding site of GyrB (Figure 2a). Compound **1** possesses excellent activity against the key Gram-negative pathogens (*E. coli*: MIC = 4 μg/mL; *K. pneumoniae*: MIC = 2 μg/mL; *P. aeruginosa*: MIC = 8 μg/mL; *A. baumannii*: MIC = 0.5 μg/mL) and also has encouraging activity against MDR *P. aeruginosa* and *A. baumannii* strains (MIC_90_ for *A. baumannii* = 2 μg/mL, MIC_90_ for *P. aeruginosa* = 8 μg/mL measured against 64 recent clinical isolates of each species) (Table S1). The compound is much less affected by efflux in *A. baumannii* than in the other Gram-negative species. Its frequency of resistance is lower than 10 × 10^–10^ at 4× and 8× MIC for the wild-type *A. baumannii* and for an efflux-defective strain of *P. aeruginosa*.

Mutant analysis in efflux-defective *P. aeruginosa* PA0750 (MH-II liquid MIC < 0.125 μg/mL, MH-II agar MIC < 0.125 μg/mL) identified mutations in seven out of eight mutants selected and showed that only one mutant carried a mutation in the *gyrB* gene, which encodes for GyrB, a target of **1**. Most other resistant mutants are probably associated with the induction of mechanisms to increase the efflux or reduce the influx of the compound. Sequencing of the *gyrB* gene in selected *A. baumannii* mutants revealed the mutation of Arg150, which is homologous to Arg136 in *E. coli* GyrB, to cysteine (R150C) and histidine (R150H). Most likely, the two mutants selected in the wild type that had no mutations in *gyrB* will have mutations affecting the intracellular concentration of the compound. For the tested *P. aeruginosa* strain as well as for *A. baumannii* efflux-proficient and efflux-deficient strains, the MIC increase in selected mutants was very large, typically 64- to >128-fold.

The frontrunner compound **1** is bactericidal in *K. pneumoniae* (MBC = 1 μg/mL) but not in *A. baumannii*, in which it reduced the colony counts to <1% but not to <0.1%, the survivors having unchanged MIC. As shown in time-kill assays, **1** is bacteriostatic in *A. baumannii*. There was no killing of *P. aeruginosa* up to 8-fold MIC, whereby MICs increased significantly as the concentration of bacterial cells grew (MIC increased to 32 with 5 × 10^6^ CFU/mL).

Compound **1** was found to have low cytotoxicity in the HepG2 human liver cell line by LDH^15^ (IC_50_ > 100 μM), MTS^17^ (IC_50_ = 78 μM), and FMC^18^ (IC_50_ = 10.3 μM) assays, having no hERG, Ca_V_1.2, and Na_V_1.5 ion channel liabilities at a 50 μM concentration (Figure S2) and possessing no in vitro mitochondrial toxicity in HepG2 cells at a 100 μM concentration (Figure S4). It did not show genotoxicity in a micronucleus test at concentrations up to 25 μM (Figure S6) and was found to be nonmutagenic against *Salmonella typhimurium* TA98 without or with S9 metabolic activation, as confirmed by a negative AMES test (Figure S9). The compound does not inhibit CYP3A4 (IC_50_ > 50 μM) (Figure S12), is stable in human (*t*_1/2_ = 71 min) and mouse hepatocytes (*t*_1/2_ = 132 min) as well as in human (*t*_1/2_ = 142 min) and mouse liver microsomes (*t*_1/2_ = 99 min) (Figure S13), and shows less than 1% hemolysis at a 100 μM concentration.

However, despite the promising on-target and antibacterial properties of inhibitor **1**, further development of this compound was hampered by its high lipophilicity (clog *P* = 5.8, clog *D* = 2.1) that resulted in low kinetic solubility (12 μM; 5.72 mg/L) and low thermodynamic solubility (6.6 μM; 3.14 mg/L). Furthermore, due to high plasma protein binding with less than 0.1% of free fraction in both human and mouse plasma, compound **1** lost its in vitro antibacterial activity against the target Gram-negative pathogens (MIC > 64 μg/mL) in the presence of 50% human serum. Therefore, we started an intensive optimization campaign aiming at increasing the solubility and free fraction while retaining or improving the excellent antibacterial activity of the initial hit compound **1**.

#### 2.1.1 Optimization Plan of Compound **1**

To establish the binding modes of novel gyrase inhibitors as a basis for structure-based optimization, we first solved cocrystal structures of compounds **1** and **7**(16) in complex with the 24 kDa fragment of *E. coli* GyrB (GyrB24) at resolutions of 1.16 Å (PDB code 7P2M) and 1.65 Å (PDB code 7P2W), respectively. Both inhibitors are bound in the ATP-binding site of GyrB24 and form similar interactions with the protein residues (Figure 2). The pyrrole NH group is involved in a hydrogen bond with the Asp73 side chain, while the carboxamide oxygen interacts with the conserved water molecule and Thr165 side-chain hydroxyl group. The 3,4-dichloro-5-methylpyrrole moiety forms several hydrophobic interactions with the residues in the lipophilic pocket of the enzyme, namely, Val43, Ala47, Val71, Ile78, Val120, and Val167. The benzothiazole scaffold forms a cation−π stacking interaction with the Arg76 side chain. In addition, a salt bridge is formed between the Arg136 side chain and the aromatic carboxylate group of inhibitors **1** and **7**. Some differences were observed in the binding of the substituent at position 4 of the benzothiazole moiety. In **1**, the nitrogen of the benzothiazole ring and the oxygen atom at position 4 form hydrogen bonds with a water molecule, while the phenyl ring has hydrophobic contacts with the side chain of Ile94. In **7**, the amide nitrogen atom interacts with a water molecule, which is in contact with the Asn46 backbone carbonyl, while the 4-benzyloxy group forms hydrophobic interactions with Ile94 and Val118. The 3-carboxylate group on the 4-benzyloxy moiety is not in direct contact with the protein but interacts with two water molecules bound to the Asp49 side chain. The described binding mode of **1** is very similar to the one reported by us for *S. aureus* GyrB24-**1** complex (PDB code 6TCK).^15^ These and other crystal structures reported herein, together with docking to explore additional interactions with the target, have been used to rationally design the new compounds.

**Figure 3:**
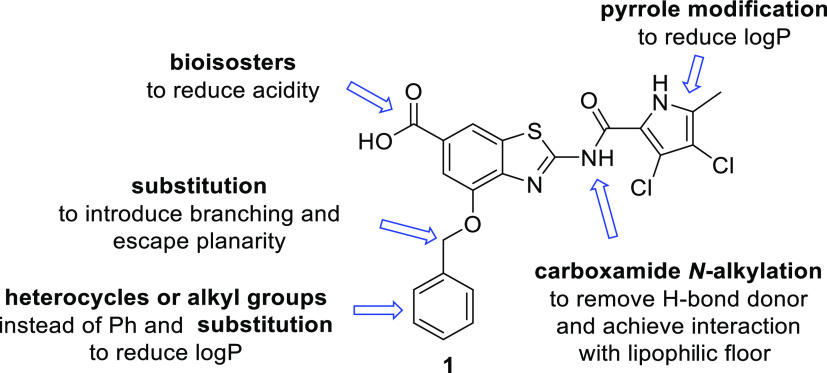
Plan for optimization of compound **1**.

Several chemical strategies were planned to increase the solubility and reduce plasma protein binding of the frontrunner **1**. Replacement of the phenyl ring by aza-heterocycles or alkyl groups, introduction of polar substituents to the phenyl ring, as well as bioisosteric replacement of the carboxylic group, and modification of the pyrrole moiety were attempted to lower lipophilicity. Carboxamide N-alkylation was aimed to remove the hydrogen bond donor and explore the interaction of the substituents with the lipophilic floor. Finally, substituents were introduced at the benzylic position to increase solubility by disrupting the planarity of the molecules^57,58^ (Figure 3).

##### 2.1.1.1 Phenyl Group Replacement and Substitution

Replacement of the phenyl ring of **1** with pyridine, pyrimidine, or *N*-methylpyridinium moiety (compounds **2**–**6**) retained the single-digit nanomolar DNA gyrase inhibitory activity, but the antibacterial activity was lost.

Although the introduction of the first nitrogen atom into the phenyl ring (compounds **2** and **3**) decreased the calculated log *D* values by one unit and introduction of the second nitrogen atom (compound **4**) or quaternization (compounds **5** and **6**) by an additional unit, the solubility remained low and all compounds were highly bound to plasma proteins (Table 1). We have shown earlier that the substitution of the phenyl ring of **1** with 4-carboxy or 3-carboxy group (compound **7**) retains good on-target activity, but again, this modification was detrimental for antibacterial activity against *E. coli*, *A. baumannii*, and *P. aeruginosa*.^16^ Whereas the same trend was observed also for 4-methylsulfonyl compound **8**, the 3-fluoro derivative **9**(16) possessed good on-target activity and activity against *A. baumannii* (MIC = 4 μg/mL) but poor physicochemical and ADME properties. Replacing the phenyl group of **1** with aminomethyl and morpholinomethyl moieties^16^ as well as with methoxymethyl moiety in **10** and 1-(dimethylamino)ethyl moiety in **11** was also detrimental for antibacterial activity although the inhibition of gyrase and topo IV was well retained. On the contrary, the gyrase inhibitory activity of 4-(2,2,2-trifluoroethoxy) analog **12** was translated to antibacterial activity, which was most pronounced in *A. baumannii* with an MIC of 8 μg/mL (Table 1). Compounds **2**–**12** were all active against the efflux-defective strain of *E. coli* with MIC ≤ 8 μg/mL, and compounds **2**, **3**, **9**, **10**, and **12** were active (MIC ≤64 μg/mL) against efflux-defective *P. aeruginosa*. This suggests that efflux is a major contributing factor to inactivity against wild-type Gram-negative bacteria. Compounds **2**, **3**, and **9**–**12** were all active against wild-type *S. aureus* (MIC values of all tested compounds are listed in Table S10).

**Table 1:**
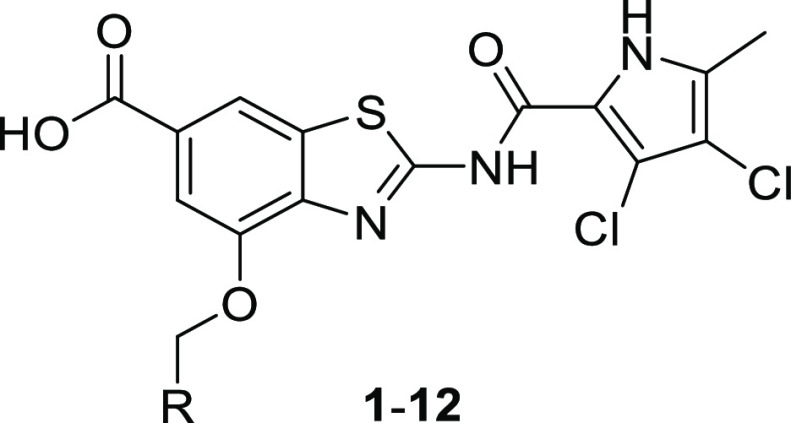

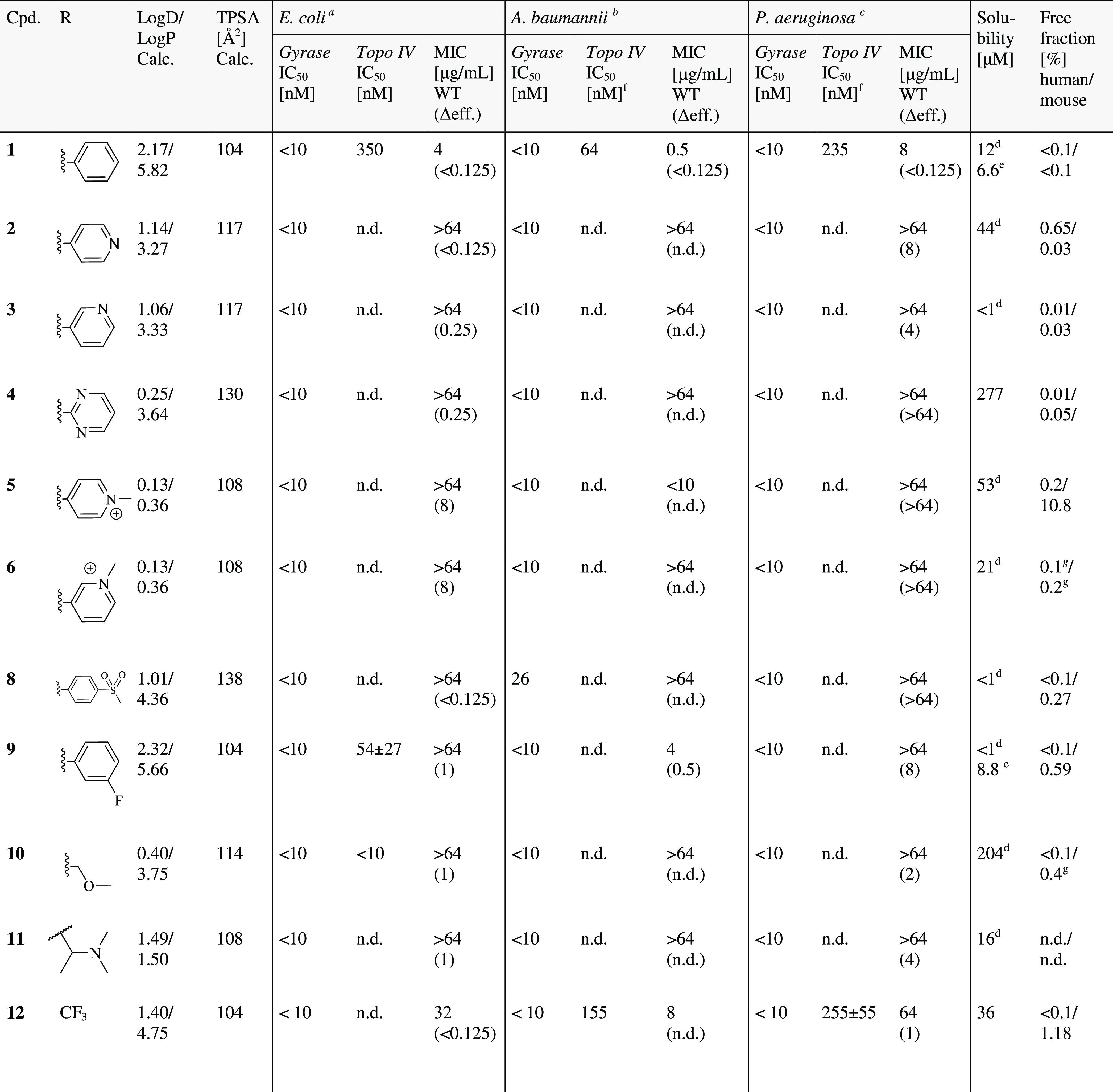
IC_50_ Values against *E. coli*, *A. baumannii*, and *P. aeruginosa* DNA Gyrase and Topoisomerase IV, MICs, Solubility, and Free Fraction Data of Analogs of the Frontrunner **1** with Replaced or Substituted Phenyl Moiety

a *E. coli* ATCC5922 (wild type), CH3130 (efflux-defective; ΔtolC-mutant isogenic to ATCC25922).

b *A. baumannii* ATCC19606 (wild type), BM4652 (efflux-defective derivative of BM4454).

c *P. aeruginosa* PAO1 (wild type), PAO750 (efflux-defective isogenic to PAO1).

d Kinetic solubility.

e Thermodynamic solubility.

f Gel-based assay.

g The results should be interpreted carefully due to low recovery; TPSA, total polar surface area; Calc., calculated; n.d., not determined; WT, wild type; Δeff., efflux-defective strain.

##### 2.1.1.2 Bioisosteric Replacement of the Carboxyl Group

The replacement of a carboxylic acid with an isosteric group to improve the physicochemical properties of a compound, while maintaining the features critical for biological activity, is a well-known medicinal chemistry strategy.^20^ We have successfully used 5-oxo-4,5-dihydro-1,3,4-oxadiazol-2-yl,^21^ 5-thioxo-4,5-dihydro-1,3,4-oxadiazol-2-yl,^22^ and 5-oxo-4,5-dihydro-1*H*-tetrazol-1-yl^22^ moieties as carboxyl group surrogates in *N*-phenylpyrrolamide gyrase inhibitors, and, following the same strategy, the 1*H*-tetrazol-5-yl (**13**),^16^ 5-oxo-4,5-dihydro-1,3,4-oxadiazol-2-yl (**14)**, and hydrazide (**15**) derivatives of **1** were prepared. Compounds **13**–**15** inhibited DNA gyrase but exhibited weak or no activity against *E. coli*, *A. baumannii*, and *P. aeruginosa* (Supporting Information Table S3).
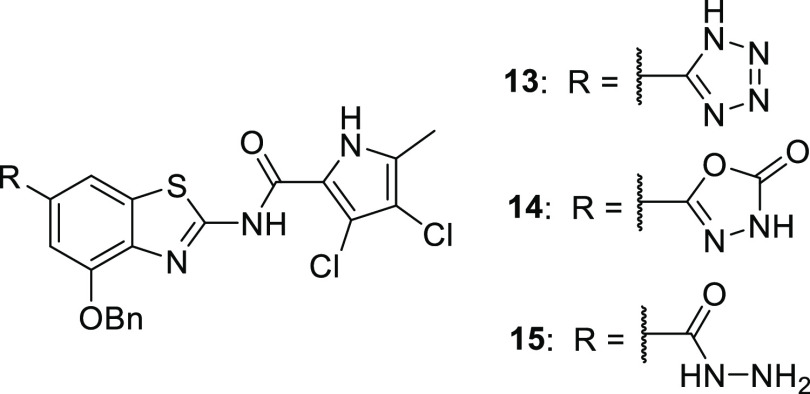


##### 2.1.1.3 Modification of Pyrrole Substitution

As revealed by the crystal structure of an *E. coli* GyrB24-**1** complex (PDB code 7P2M), a lipophilic pocket that binds the pyrrole 5-methyl group would allow lengthening it to an aminomethyl moiety that could interact with a negatively charged area and possibly increase the binding affinity. This modification is in line with the recent assumption that small molecules that are most likely to accumulate in Gram-negative bacteria contain an amine, are amphiphilic and rigid, and have low globularity.^23^ Guided by these observations and principles, the 5-aminomethylpyrrole derivative **16**(36) was prepared. Although it was a strong inhibitor of *E. coli* gyrase (IC_50_ < 10 nm), its IC_50_ values for the inhibition of *A. baumannii* and *P. aeruginosa* gyrase were only in the micromolar range, and the compound was inactive against the tested Gram-negative bacteria. The methyl ester derivative **17** was devoid of on-target and antibacterial activity, indicating the importance of the carboxylic group in position 6. Notably, the free fraction of **16** in human serum was increased to 0.59% (Supporting Information Table S4).
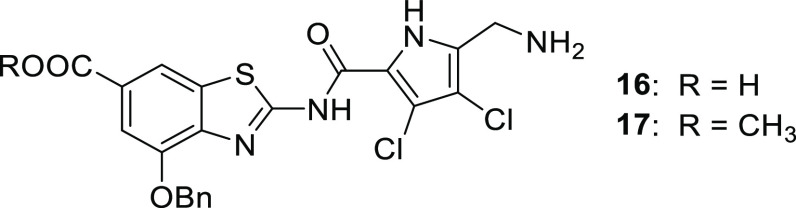


##### 2.1.1.4 N-Alkylation of Carboxamide or Thiazole

Inspection of the crystal structure of **1** in complex with *E. coli* GyrB24 (PDB code 7P2M) revealed that the carboxamide NH group is not involved in hydrogen bonding and the alkyl group attached to it would point toward the lipophilic floor of the ATP-binding site (Figure 2a) and thus possibly contribute to binding. Bearing in mind that carboxamide N-alkylation would also distort the planarity of the carboxamide group and thus possibly increase the solubility, compounds **18**, an *N-*methyl derivative of **1**, and **19**, an *N-*methyl derivative of **16**, were prepared. Whereas strong inhibition of gyrase was kept, the weaker inhibition of topo IV and solid in vitro activity against *E. coli*, *A. baumannii*, and *P. aeruginosa* were retained in **18** and the solubility and free fraction remained low. The amino derivative **19** was a weaker inhibitor of both gyrase and topo IV; it was devoid of antibacterial activity and possessed very low kinetic solubility. Because the carboxamide and benzothiazole N*-*substituents were expected to interact with the lipophilic floor similarly to the groups bound to oxygen in position 4, we tested the effect of moving a substituent from *O*-4 to the carboxamide *N*-atom (compound **20)** or removing the alkoxy substituent in position 4 and attaching its alkyl part to the thiazole (compounds **22**–**25**) or carboxamide *N*-atom (compounds **21**, **26**). All resulting compounds except **21** were low nanomolar inhibitors of *E. coli*, *A. baumannii*, and *P. aeruginosa* gyrase, but only **26** was active against the three pathogens. Compound **26** is also less bound to proteins in human and mouse plasma. From a comparison of the carboxamide *N*-methyl compounds **18**, **20**, and **26**, it can be concluded that a substituent in position 4 does not significantly affect gyrase inhibition but the 4-hydroxyl group is detrimental to antibacterial activity (Table 2).

**Table 2:**
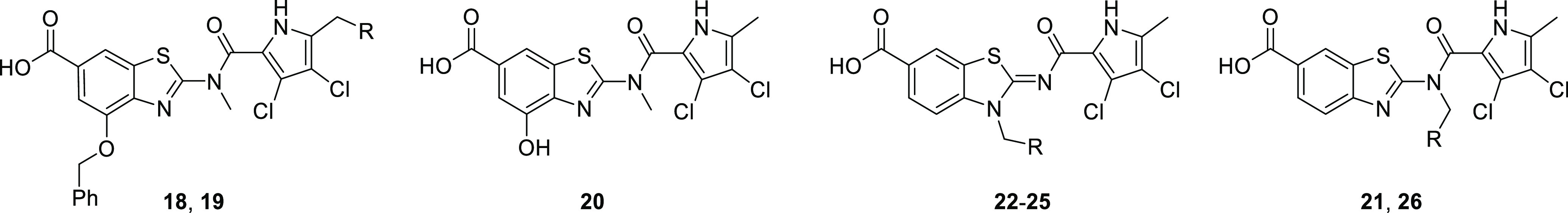

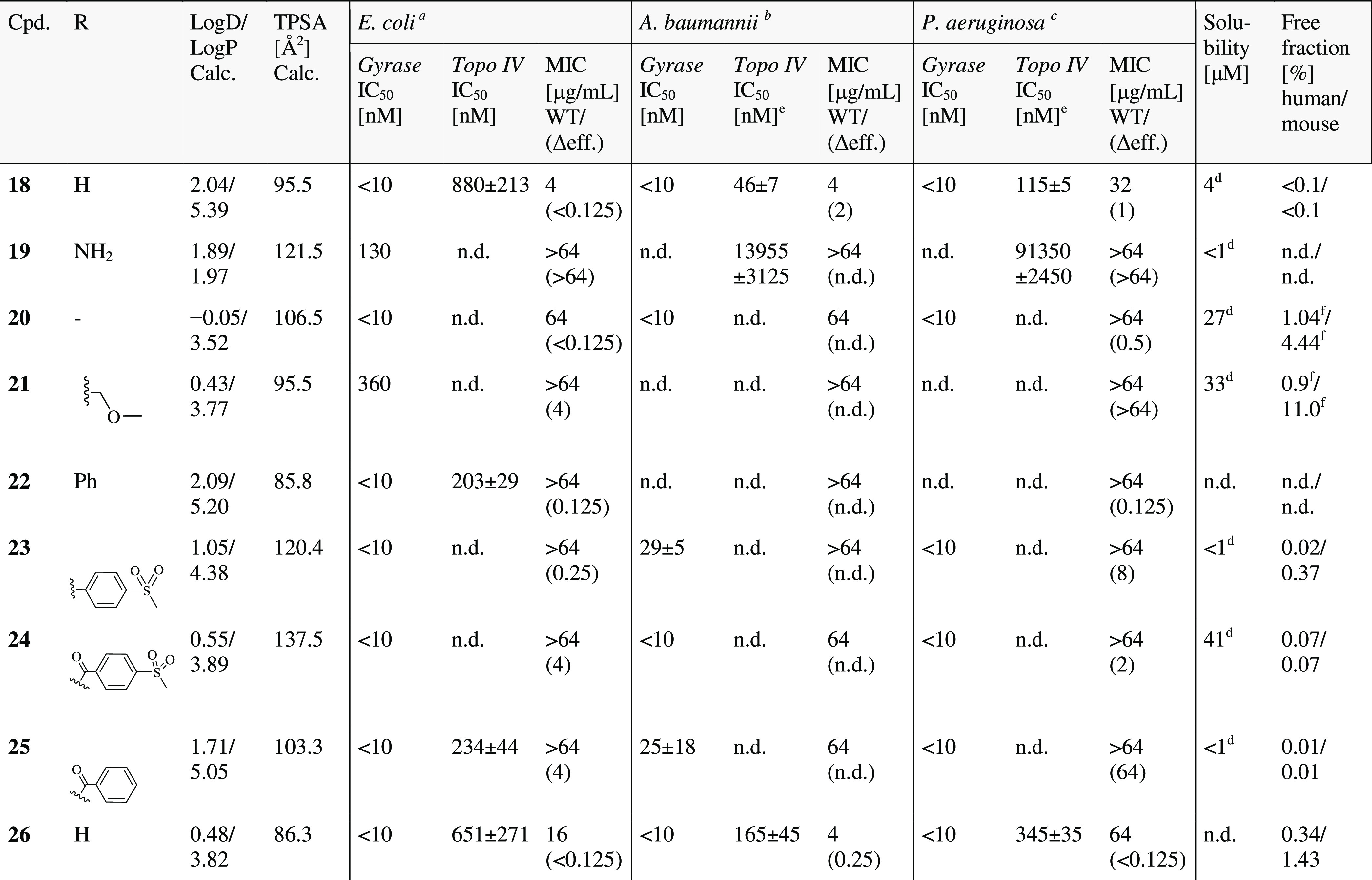
IC_50_ Values against *E. coli*, *A. baumannii*, and *P. aeruginosa* DNA Gyrase and Topoisomerase IV, MICs, Solubility, and Free Fraction Data of the N*-*Alkylated Analogs

a *E. coli* ATCC5922 (wild type), CH3130 (efflux-defective; ΔtolC-mutant isogenic to ATCC25922).

b *A. baumannii* ATCC19606 (wild type), BM4652 (efflux-defective derivative of BM4454).

c *P. aeruginosa* PAO1 (wild type), PAO750 (efflux-defective isogenic to PAO1).

d Kinetic solubility.

e Gel-based assay.

f The results should be interpreted carefully due to low recovery; TPSA, total polar surface area; Calc., calculated; n.d., not determined; WT, wild type; Δeff., efflux-defective strain.

##### 2.1.1.5 Substitution at the Benzylic Position

Substitution at the benzylic position to disrupt molecular planarity and symmetry has been applied for solubility improvement in several medicinal chemistry optimization programs.^24−29^ Introduction of a methyl group at the benzylic position of **1** resulted in compound **27**.
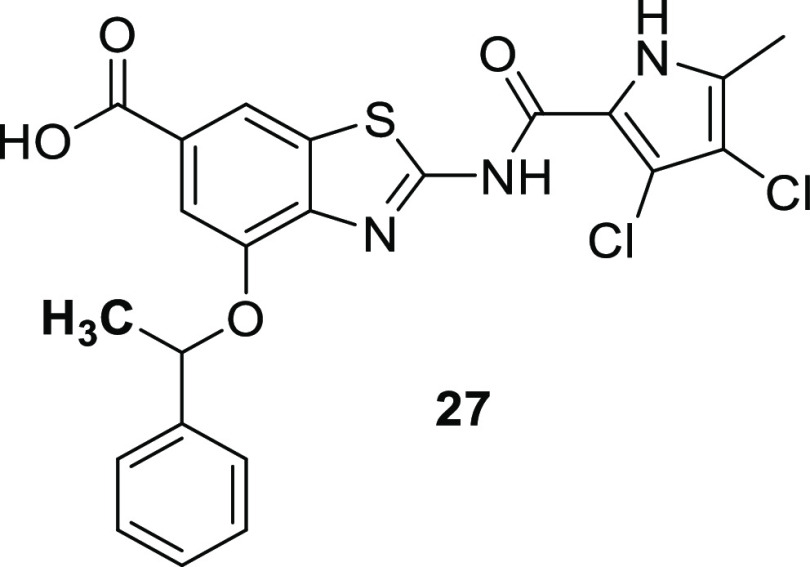


Compound **27** possessed at least 7-fold better kinetic solubility (>80 μM; >39 mg/L) and 14-fold better thermodynamic solubility (92 μM = 45.3 mg/L). Compound **27** was an excellent inhibitor of gyrase from *E. coli* (IC_50_ < 10 nM), *A. baumanni*i (IC_50_ = 15.6 nM), and *P. aeruginosa* (IC_50_ < 10 nM) and also inhibited topo IV from *E. coli* (IC_50_ = 320 nM), *A. baumannii* (IC_50_ < 10 nM), and *P. aeruginosa* (IC_50_ = 29 nM).

Similarly to **1**, compound **27** does not induce cleavage complex formation (IC_50_ > 100 μM) and very weakly inhibits ATP-independent DNA relaxation (IC_50_ = 87 μM). Importantly, compared to **1**, compound **27** shows a 12-fold better selectivity against a related human target topoisomerase IIα (IC_50_ = 25 μM). Overall, compound **27** possesses improved antibacterial activity compared to compound **1** (Table 3). Whereas there is no effluxing of **27** in *A. baumannii*, efflux occurs in *P. aeruginosa* but to a smaller extent than for **1**. While retaining excellent *in vitro* activity against *A. baumannii*, compound **27** also displayed improved activity against *P. aeruginosa*, possibly due to a 10-fold stronger inhibition of topo IV and more balanced inhibition of both DNA gyrase and topo IV (Table 3).

**Table 3:** Comparison of On-Target and Antibacterial Activities of Compounds **1** and **27**^a^

enzyme	Cpd. **1** IC_50_ [nM]	Cpd. **27** IC_50_ [nM]
*E. coli* gyrase	<10	<10
*E. coli* topo IV	350	320
*A. baumannii* gyrase	<10	16
*A. baumannii* topo IV	64	6.7
*P. aeruginosa* gyrase	<10	<10
*P. aeruginosa* topo IV	235	29
human topoisomerase IIα	1950	25,000

pathogen, strain ID (phenotype)	Cpd. **1** MIC [μg/mL]	Cpd. **27** MIC [μg/mL]
*E. coli*, ATTC25922 (WT)	4	1
*E. coli*, CH3130 (efflux-def.)	<0.125	<0.125
*K. pneumoniae*, ATCC13883 (WT)	2	1
*K. pneumoniae*, 1161486a (efflux-def.)	n.d.	n.d.
*P. aeruginosa*, PAO1 (WT)	8	1
*P. aeruginosa*, PAO750 (efflux-def.)	<0.125	<0.125
*A. baumannii*, ATCC19606 (WT)	0.5	0.5
*A. baumannii*, BM4454 (clinical MDR)	<0.125	0.5
*A. baumannii*, BM4652 (efflux-def.)	<0.5	0.5

a n.d., not determined; WT, wild type; MDR, multidrug-resistant.

Compound **27** has a very good activity against MDR *A. baumannii* strains (MIC_90_ = 2 μg/mL, based on broth microdilution assay with 61 clinical isolates; Table S2) and seven selected *P. aeruginosa* clinical isolates (Table 4). However, in the presence of 50% human serum, **27** lost its activity (MIC > 64 μg/mL) against *E. coli*, *P. aeruginosa*, and *A. baumannii*. The frequency of resistance of **27** in *A. baumannii* and *E. coli* remained low, beyond the detection limit; but it was higher in wild-type *P. aeruginosa* (4.1 × 10^–8^ at 8 × MIC) where the selected mutants were resistant to **27** with MIC increased between 4- and >16-fold compared to the parental. On the contrary, no resistant clones were isolated in the frequency of resistance studies of **27** in *A. baumannii*. Like **1**, compound **27** is bacteriostatic against *A. baumannii*, but an important difference was observed between both compounds in *P. aeruginosa* activity. Whereas for **1** there was no killing of *P. aeruginosa* up to 8-fold MIC, compound **27** was bacteriostatic with an MIC of 2 μg/mL.

**Table 4:** MIC Values of Compound **27** and Selected Derivatives against Some Multiresistant Strains of *A. baumannii* and *P. aeruginosa*

strain ID	species	original no.	genotype/phenotype	**27**	**30**	**31**	**34**	**35**	**36**	**46**	**47**	**48**	**51**	**65**
EN7	*A. baumannii*	ATCC19606	WT	1	16	1	8	4	4	2	1	2	1	2
EN16	*A. baumannii*	BM4454	WT	0.5	8	0.5	8	4	2	2	1	1	1	4
EN17	*A. baumannii*	BM4652	efflux-defective	0.5	0.125	0.125	0.125	0.25	0.5	0.5	1	1	2	1
EN273	*A. baumannii*	NMI 2692/14	clinical MDR	1	8	0.5	2	2	2	2	1	2	2	4
EN274	*A. baumannii*	NMI 2699/14	clinical MDR	1	64	1	2	2	2	1	1	2	2	8
EN276	*A. baumannii*	NMI 2704/14	clinical MDR	1	4	0.5	2	1	2	2	1	2	2	4
EN277	*A. baumannii*	NMI 2715/14	clinical MDR	1	16	1	1	4	4	4	4	4	2	8
CH8730	*A. baumannii*	CH8730	gyrB R150C	8	>64	>64	>64	>64	>64	>64	>64	>64	>64	>64
EN0146	*P. aeruginosa*	NMI 3793/07	clinical MDR	1	16	1	8	8	4	8	2	4	4	8
EN0147	*P. aeruginosa*	NMI 2500/14	clinical MDR	1	>64	1	8	8	4	8	1	4	2	8
EN0148	*P. aeruginosa*	RYC 03131390	clinical MDR	2	32	2	16	16	8	32	4	8	4	64
EN0149	*P. aeruginosa*	NMI 2620/03	clinical MDR	1	32	0.5	16	8	2	8	1	2	2	8
EN0340	*P. aeruginosa*	3947/14	clinical MDR	1	16	1	8	8	4	8	2	4	4	8
EN0343	*P. aeruginosa*	3957/14	clinical MDR	1	64	1	16	16	2	8	2	2	2	16
CH9393	*P. aeruginosa*	CH9393	gyrB R138C	>64	>64	>64	>64	>64	>64	>64	>64	>64	>64	>64
CH9389	*E. coli*	CH9389	gyrB R136H	>64	>64	>64	>64	>64	64	>64	>64	>64	>64	>64

An important difference between the original hit **1** and compound **27** was observed also in the dependence of MICs on the inoculum size. Whereas in **1** MICs against *P. aeruginosa* increased significantly, up to 32 μg/mL, as bacterial cell concentration increased, compound **27** did not show any MIC dependence on the inoculum size in *P. aeruginosa*.^59^ Insensitivity of **27** and derivatives (see below) to the inoculum effect against *P. aeruginosa* and its excellent MICs against multidrug-resistant *P. aeruginosa* (Table 4) are a significant improvement over the initial hit **1**.

Compound **27** possessed an IC_50_ of 19.7 μM (20-fold higher than MIC) in a HepG2 fluorometric microculture cytotoxicity assay (FMCA)^18^ and was thus less cytotoxic than the initial hit **1 (**IC_50_ = 10.3 μM). It had no hERG, Ca_V_1.2, and Na_V_1.5 ion channel liabilities at a 10 μM concentration (Figure S3) and was neither genotoxic nor mutagenic in both the absence or presence of S9 metabolic activation, as confirmed by micronucleus (Figure S8) and AMES tests (Figure S10). No mitochondrial toxicity of **27** could be observed in HepG2 cells in vitro (Figure S5). Compound **27** is stable in human (*t*_1/2_ = 92 min) and mouse hepatocytes (*t*_1/2_ = 45 min) and gives rise to less than 1% hemolysis in 1 h at 100 μM.

Both stereoisomers of compound **27** were synthesized, and the enantiomer (*S*)-**27** (74% ee) was found more active than (*R*)-**27** (62% ee) against wild-type *E. coli*, *A. baumannii*, and *P. aeruginosa* strains. Interestingly, compound **27** was the only compound in the whole series showing activity against the novobiocin-resistant *A. baumannii* mutant GyrB R150C (MIC = 8 μg/mL), which resides mainly in the (*S*)-enantiomer, as confirmed by several independent experiments. On the other hand, **27** was not active against novobiocin-resistant *E. coli* GyrB R136H mutant and *P. aeruginosa* GyrB R138C mutants. Comparing the inhibition of gyrase and topo IV, compound **27** as well as its two enantiomers are better inhibitors of gyrase in *E. coli* and *P. aeruginosa*, whereas in *A. baumannii*, the inhibition of topo IV is better.

**Figure 4:**
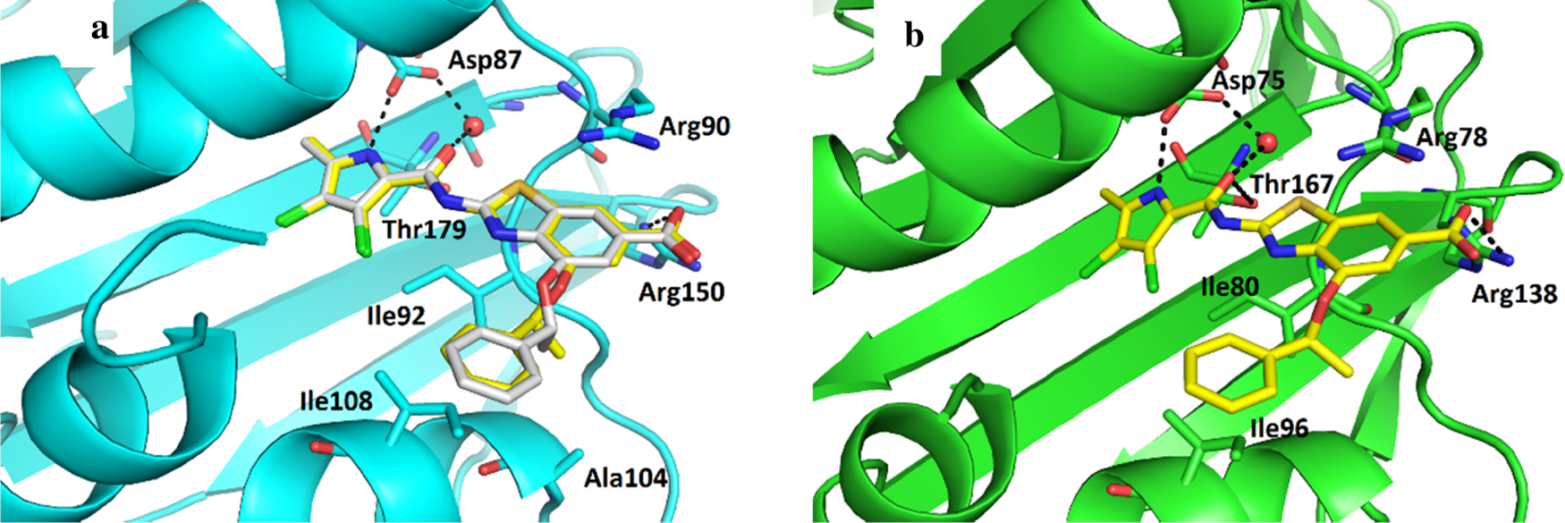
(a) Cocrystal structure of the inhibitor **27** [(*S*)-enantiomer in yellow sticks, (*R*)-enantiomer in gray sticks] in complex with *A. baumannii* GyrB23 (in cyan cartoon) (PDB 7PQL, 7PQM). (b) Cocrystal structure of (*S*)-**27** (in yellow sticks) in complex with *P. aeruginosa* GyrB24 (in green cartoon) (PDB 7PTG). For clarity, only amino acid residues that interact with inhibitor are shown as sticks. Water molecules are presented as red spheres, and hydrogen bonds are shown as dashed black lines.

To aid the development of GyrB inhibitors for the treatment of *A. baumannii* and *P. aeruginosa* infections, we solved the crystal structures of *A. baumannii* GyrB23 in complex with the racemic inhibitor **27** (PDB code 7PQL, resolution 1.60 Å) and in complex with its enantiomer (*S*)-**27** (PDB code 7PQM, resolution 1.55 Å) as well as the crystal structure of (*S*)-**27** in complex with *P. aeruginosa* GyrB24 (PDB code 7PTG) (Figure 4). Further on, the crystal structures of *A. baumannii* GyrB23 and *P. aeruginosa* GyrB24 subdomains in complex with novobiocin (PDB codes 7PQI and 7PTF) were solved for the first time (Supporting Information Figure S1). This structural information is important because so far there have been no crystal structures of *A. baumannii* GyrB complexes with small molecules in the PDB, and for *P. aeruginosa* GyrB only the structures of two complexes (PDB codes 6M1S and 6M1J) have been reported recently.^30^

The racemic inhibitor **27** was modeled in the electron density of its *A. baumannii* GyrB23 complex in the (*R*)- and (*S*)-configurations with occupancies of 40 and 60%, respectively. The observed binding modes of the (*S*)-configurated inhibitor in 7PQL and (*S*)-**27** in 7PQM were exactly the same, as expected. They formed similar interactions that are characteristic of this structural class of compounds, namely, hydrogen bonds with Asp87 and a conserved water molecule, Thr179 and Arg150, and cation−π interaction with Arg90. Hydrophobic interactions were formed between the pyrrole moiety and Val57, Ala61, Val85, Val181, and Val134, while the hydrophobic α-methylbenzyl group at position 4 of the benzothiazole core interacted with Ile92, Ala104, and Ile108 (Figure 4a).

In the crystal structure of (*S*)-**27** in complex with *P. aeruginosa* GyrB24 (Figure 4b; PDB code 7PTG), the observed inhibitor binding mode is very similar to the one in *A. baumannii* GyrB23 ATP-binding site with hydrogen bonds to Asp75, conserved water molecule, Thr167 and Arg138, cation−π stacking with Arg78 and the 4-(α-methyl)-benzyloxy moiety hydrophobic contacts with Ile80 and Ile96 side chains. The pyrrolamide moiety is bound in the lipophilic pocket and interacts with Val43, Val73, Met97, Val122, and Val169.

The binding modes of novobiocin in the *A. baumannii* GyrB23 complex 7PQI and in the *P. aeruginosa* GyrB24 complex 7PTF closely resemble that of *E. coli* and *S. aureus* GyrB-novobiocin complexes 1AJ6 and 4URO, respectively. Important interactions are hydrogen bonds formed with Asp87 in *A. baumannii* (Asp73 in *E. coli*, Asp75 in *P. aeruginosa*), the conserved water molecule, Asn60 in *A. baumannii* (Asn46 in *E. coli*, Asn48 in *P. aeruginosa*), Gly 95 in *A. baumannii* (Gly81 in *E. coli*, Asp83 in *P. aeruginosa*), and Arg150 in *A. baumannii* (Arg136 in *E. coli*, Asp138 in *P. aeruginosa*) as well as cation−π stacking with Arg90 in *A. baumannii* (Arg76 in *E. coli*, Arg78 in *P. aeruginosa*).

#### 2.1.2 Optimization of the Follow-Up Compound **27**

Improved antibacterial activity and partly ameliorated ADMET properties of the branched derivative **27** in comparison to the original hit **1** stimulated us to further optimize compound **27** toward more soluble and less plasma protein-bound analogs using devised strategies. This resulted in several derivatives, which retained activity against Gram-negative bacteria (Table 5, Tables S5–S9).

**Table 5:**
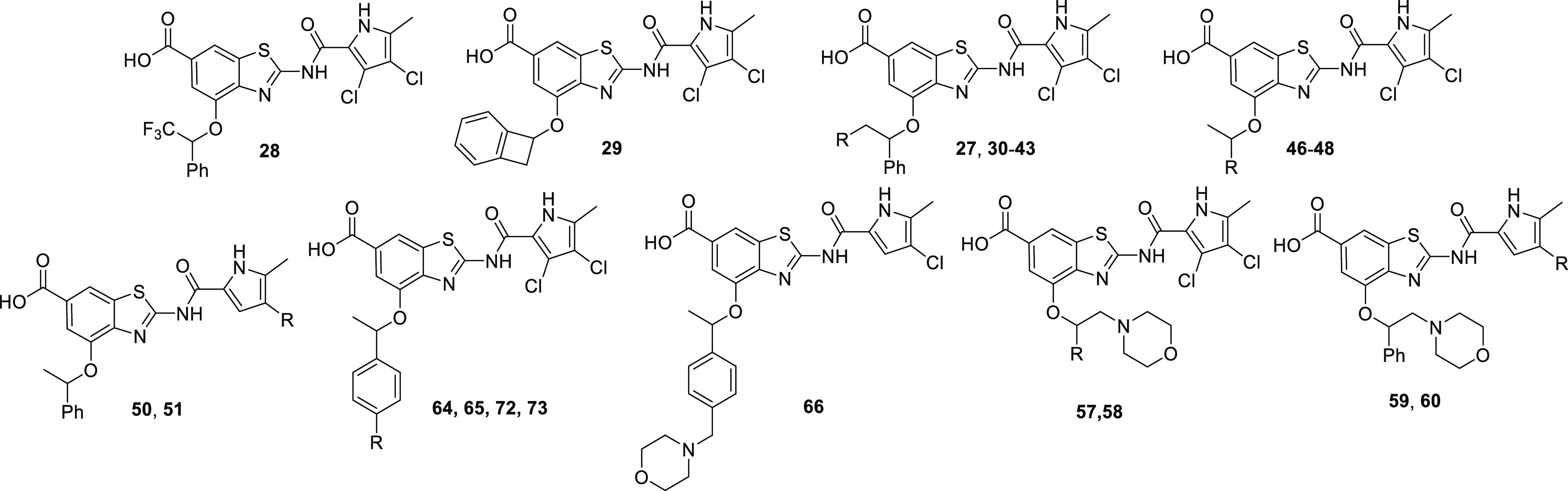

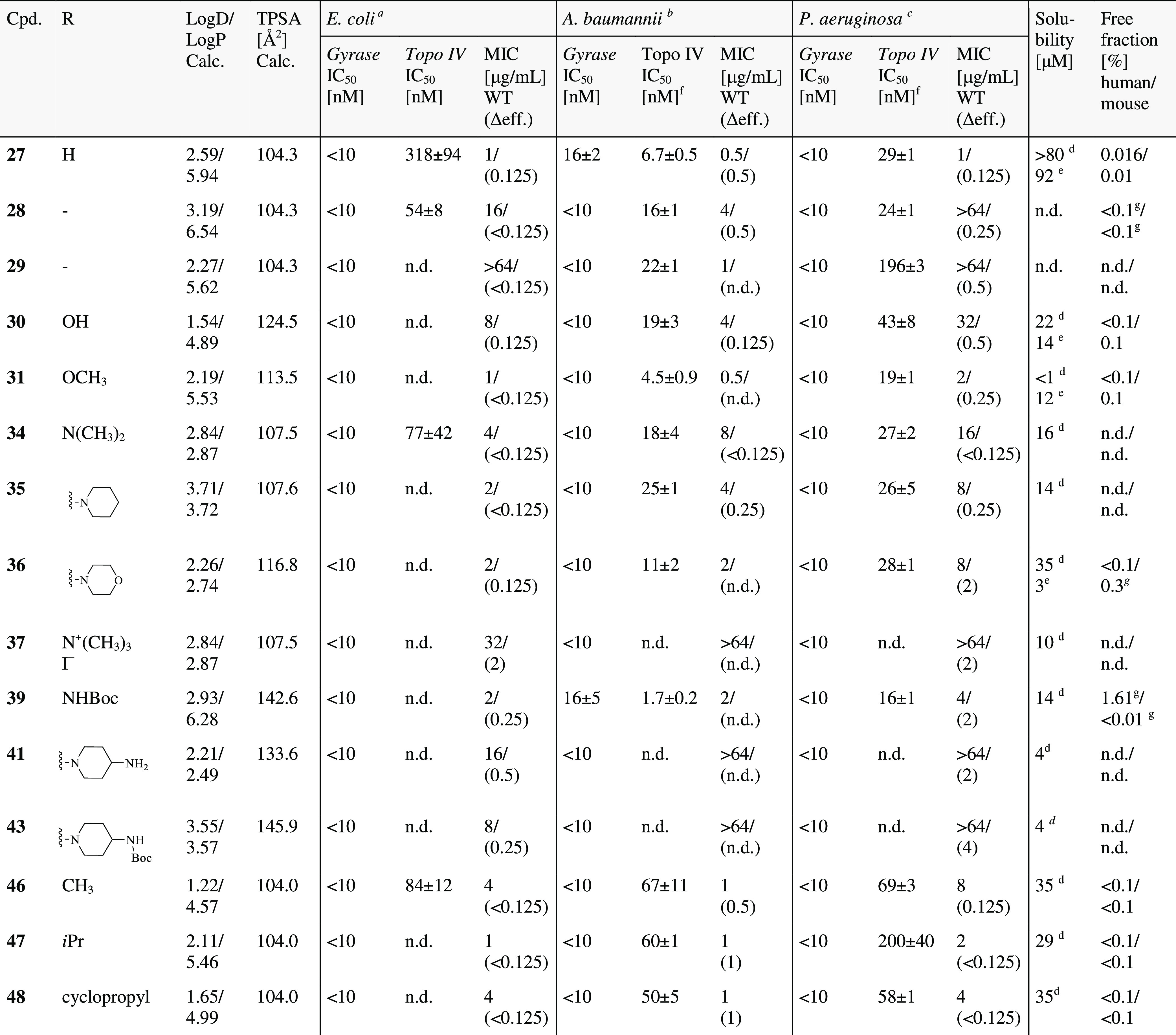

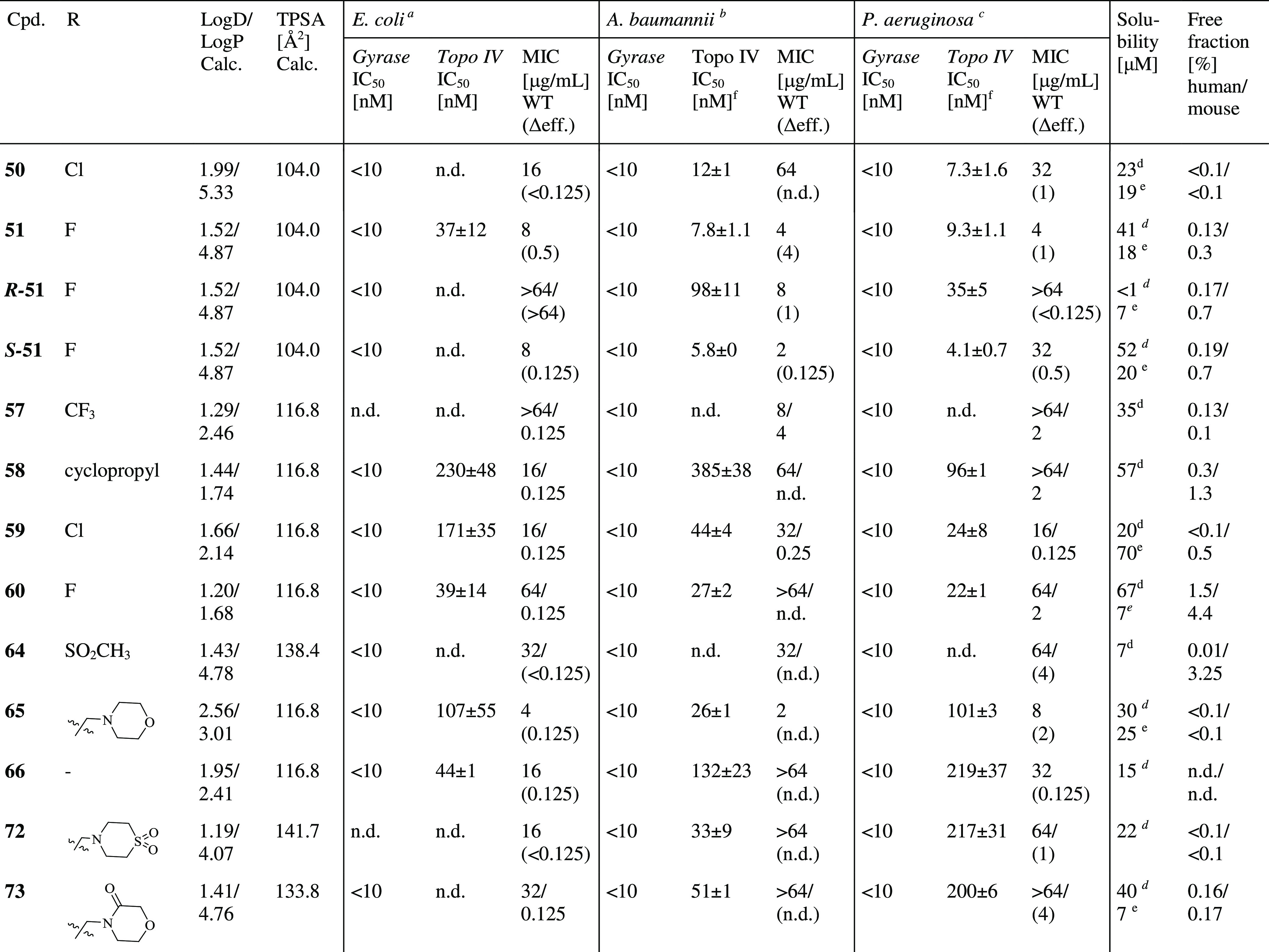
IC_50_ Values against *E. coli*, *A. baumannii*, and *P. aeruginosa* DNA Gyrase and Topoisomerase IV, MICs, Solubility, and Free Fraction Data of Derivatives of Compound **27**, Active against Gram-Negative Bacteria

a *E. coli* ATCC5922 (wild type), CH3130 (efflux-defective; ΔtolC-mutant isogenic to ATCC25922).

b *A. baumannii* ATCC19606 (wild type), BM4652 (efflux-defective derivative of BM4454).

c *P. aeruginosa* PAO1 (wild type), PAO750 (efflux-defective isogenic to PAO1).

d Kinetic solubility.

e Thermodynamic solubility.

f Gel-based assay.

g The results should be interpreted carefully due to low recovery; TPSA, total polar surface area; Calc., calculated; n.d., not determined; WT, wild type; Δeff., efflux-defective strain.

##### 2.1.2.1 Introduction of Polar Substituents to the Stereogenic Carbon Atom

To test the effect of increasing the polarity and size of the substituents attached to the stereogenic carbon atom, the compounds compiled in Table S5 were prepared. Replacement of the methyl by a trifluoromethyl group to give **28** slightly reduced the activity against *E. coli* and *A. baumannii*, whereas the activity against wild-type *P. aeruginosa* was lost. Compound **29** bearing a benzocyclobutyl substituent retained excellent activity against *A. baumannii* (MIC = 1 μg/mL) but was inactive (MIC > 64 μg/mL) against *E. coli* and *P. aeruginosa*. Notably, the antibacterial activity against *A. baumannii*, *P. aeruginosa*, and *E. coli* was preserved in compounds **30** and **31** containing oxy-substituents as well as in compounds **34**–**36** with tertiary amine substituents at the methylene group. The aminomethyl compound **33**, its quaternized derivative **37**, the *N*-acetylamino compound **38**, and the triazolomethyl derivative **40** were inactive, whereas the *N-*Boc compound **39** possessed activity against the three Gram-negative bacteria. Whereas the piperidinomethyl and morpholinomethyl compounds **35** and **36** displayed good antibacterial activity, the introduction of the second basic center or substitution in position 4 of piperazine ring (compounds **41**–**44**) was detrimental for antibacterial activity as was quaternization of the amino group (compound **45**).

Whereas good antibacterial activity of **30**, **31**, **34**–**36**, and **39** went hand in hand with good inhibition of gyrase and topo IV, the absence of antibacterial activity of the other compounds in Table S5 could not be attributed to lower or absence of on-target activity but rather to efflux issues. All active compounds retained good activity also against multidrug-resistant *A. baumannii* and *P. aeruginosa* strains, with compounds **31** and **36** showing the best performance against MDR strains (Table 4). Although for the five active compounds **30**, **31**, and **34**–**36** TPSA was increased by 3.2–20.2 Å^2^ and calculated log *P* values were decreased by 0.4–3.2 units, the solubility was not improved significantly and the compounds remained almost completely bound to the human and mouse plasma proteins. Compounds **28**–**45**, including quaternary amines, were all active against wild-type *S. aureus* (MIC < 0.125–64 μg/mL) (Table S10).

##### 2.1.2.2 Phenyl Group Replacements

The contribution of the phenyl substituent to lipophilicity of **27** in terms of clog *P* is almost 1.5; therefore, its replacement with less lipophilic smaller moieties appeared as a promising approach for the improvement of solubility and plasma protein binding (Table S6). Small alkyl moieties such as methyl (compound **46**), isopropyl (compound **47**), and cyclopropyl (compound **48**) were found to be good phenyl surrogates regarding gyrase inhibition and activity against *E. coli*, *A. baumannii*, and *P. aeruginosa*. However, in comparison to **27**, the inhibition of *A. baumannii* and *P. aeruginosa* topo IV was decreased. Unfortunately, the lower-calculated log *P* values of **46**–**48** did not result in solubility or free fraction improvement. Compound **49** with tetrahydrofuran-3-yl substituent replacing the 1-phenylethyl moiety in **27** retained a single-digit nanomolar inhibition of gyrase but its antibacterial activity against Gram-negative pathogens was almost lost (Table S6).

##### 2.1.2.3 Variation of the Pyrrole Ring Substitution

3,4-Dichloro-5-methyl-1*H*-pyrrole-2-carboxamide moiety of **27** is a major contributor to the compound’s high lipophilicity. Therefore, an array of analogs featuring less lipophilic pyrroles were prepared (Table S7). Removal of chlorine in the pyrrole position 3 of **27** resulted in the substantial loss of antibacterial activity (compound **50**), which was regained to a great extent by replacement of the chlorine in **50** by fluorine to give **51**. The latter also possessed good activity against clinical multidrug-resistant strains of *A. baumannii* and *P. aeruginosa* (Table 4). Because the gyrase and topo IV inhibitory activities of **50** and **51** remained comparable to those of compound **27**, a more intensive efflux, poor membrane permeability, and/or unknown off-target activity^31^ could be responsible for a weaker antibacterial activity of compound **50**. On the contrary, the replacement of chlorine in **50** with a cyano group (compound **52**) or hydrogen atom (compound **53**) further impaired the antibacterial activity (IC_50_ > 64 μg/mL). Between the two enantiomers of **51**, (*S*)-**51** (82% ee) possessed better activity against *P. aeruginosa* and *A. baumannii*, possibly due to 10- to 15-fold stronger inhibition of topo IV from both species than (*R*)-**51** (>95% ee). The results for compound (*S*)-**51** are particularly interesting if considered for development against *A. baumannii* only. The activity is still good, and the solubility/free fraction was improved relative to **1**. As evident from Table S7, moving the fluorine atom in **51** to position 3 to give compound **54** was again detrimental to antibacterial activity, most likely due to weaker inhibition of *A. baumannii* and *P. aeruginosa* topo IV. Finally, the elongation of the pyrrole 5-methyl group in **27** to aminomethyl substituent (compound **55**) was also detrimental to activity against *E. coli*, *A. baumannii*, and *P. aeruginosa*. The drop in antibacterial activity was more pronounced than in compound **16**; however, the introduction of 5-aminomethyl group increased the free fraction of **55** in human plasma to 1.28%.

**Table 6:**
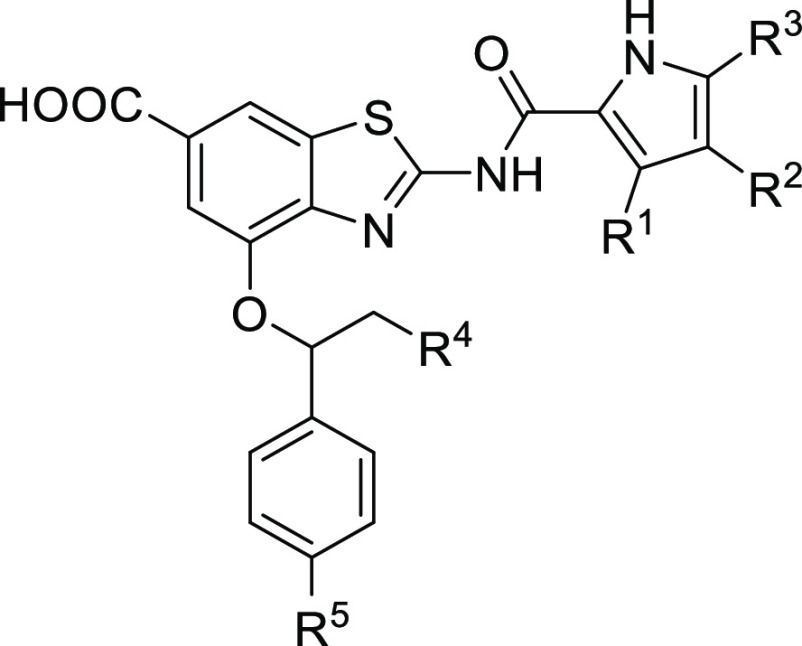

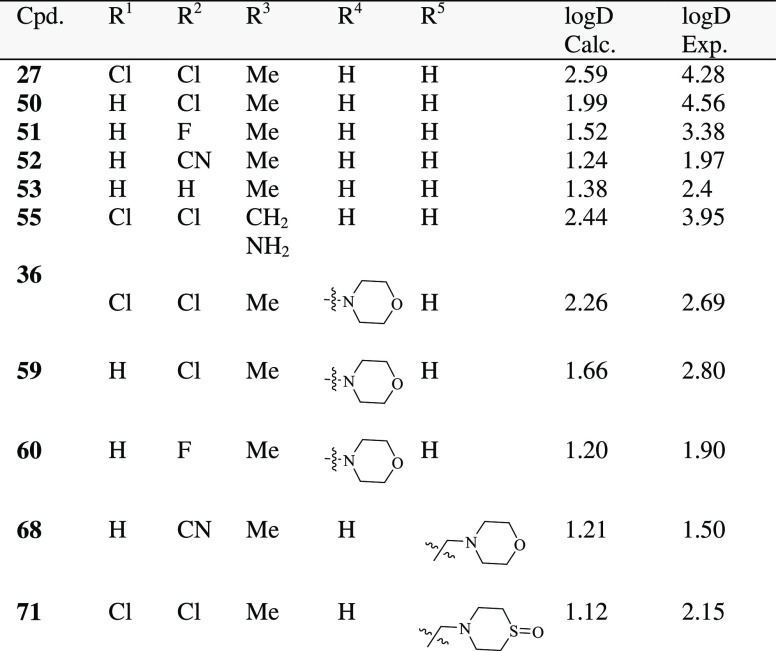
Experimental log *D* (pH 7.4) Values of Representative Compounds

##### 2.1.2.4 Combined Approaches

The modifications of compound **27** that included a combination of favorable substitution at the stereogenic carbon (see Section 2.1.2.1), phenyl group replacement (see Section 2.1.2.2), and variation of the pyrrole substituents (see Section 2.1.2.3) were also carried out (Table S8). A combination of morpholinomethyl moiety and methyl as a phenyl surrogate (compound **56**) was detrimental to antibacterial activity, but the trifluoromethyl analog **57** retained antibacterial activity against *A. baumannii* (wild type: MIC = 8 μg/mL; clinical MDR strains: IC_50_ = 8–16 μg/mL) and was found inactive (MIC > 64 μg/mL) against *E. coli* and *P. aeruginosa*. A combination of morpholinomethyl moiety and cyclopropyl group as a phenyl surrogate afforded compound **58**. It possessed improved kinetic solubility (57 μM) and plasma protein binding properties (free fraction 0.3 and 1.3% in human and mouse plasma, respectively), retained single-digit nanomolar inhibition of *E. coli*, *A. baumannii*, and *P. aeruginosa* gyrase, and inhibited topo IV of the three species in the 100 nM range. Compound **58** retained activity against wild-type strains of *E. coli* (MIC = 16 μg/mL) and *A. baumannii* (MIC = 64 μg/mL) and only against the efflux-defective strain of *P. aeruginosa*. The excellent antibacterial activity of **36** with morpholinomethyl and phenyl substituents at the branching point decreased or disappeared after the removal of chlorine in position 3 without or with the concomitant variation of halogen in position 4 (**59**: R = Cl; **60**: R = F), although no substantial changes in either gyrase or topo IV inhibition were observed. Notably, compound **60** showed promisingly increased free fraction in human and mouse plasma of 1.5 and 4.4%, respectively. It also possessed improved kinetic solubility and retained borderline antibacterial activity (MIC = 64 μg/mL) against wild-type *P. aeruginosa* and *E. coli*. Similarly, the removal of chlorine in position 3 of antibacterially active compound **46** and replacement of chlorine in position 4 with fluorine (compound **61**) or cyano group (compound **62**) were detrimental to the activity against *E. coli*, *A. baumannii*, and *P. aeruginosa* although the IC_50_ values of gyrase inhibition were still in the 10–150 nM range. The replacement of 3,4-dichloro-5-methylpyrrole moiety of **46** with 2-methyl-4-chloroimidazole in **63** further lowered log *D* but gyrase inhibition and antibacterial activity were drastically reduced.

##### 2.1.2.5 Introduction of *para* Substituents to the Phenyl Ring

4-Methylsulfonybenzyl derivative **64**, although possessing only weak antibacterial activity, suggested that the *para* substitution of the phenyl ring could be tolerated in the derivatives of compound **27**. After inspection of the crystal structures of **27** in complex with *A. baumannii* GyrB23 (PDB codes 7PQL, 7PQM) and *P. aeruginosa* GyrB24 (PDB code 7PTG), the 4-morpholinomethyl derivative **65** was prepared and found to possess good antibacterial activity against *E. coli* (MIC = 4 μg/mL), *A. baumannii* (MIC = 2 μg/mL), and *P. aeruginosa* (MIC = 8 μg/mL) as well as against some clinical MDR *A. baumannii* (MIC = 4–8 μg/mL) and *P. aeruginosa* strains (MIC = 8–64 μg/mL) (Table 4). Its efflux from *A. baumannii* and *P. aeruginosa* was much less pronounced than from *E. coli*. In an attempt to improve the antibacterial activity, solubility, and free fraction, *p*-morpholinomethyl substitution of the phenyl ring was first combined with different pyrrole substitution patterns. Unfortunately, modifications in the pyrrole ring of **65**, including the removal of the chlorine in position 3 to give compound **66** and its replacement with fluorine in **67** or cyano group in **68**, reduced antibacterial activity, mostly due to efflux issues. The morpholine moiety of **65** was then replaced by piperazine (**69**, **70**), 1-oxothiomorpholine (**71**), 1,1-dioxothiomorpholine (**72**), and 3-oxomorpholine (**73**), but again all of these variations resulted in the efflux-related reduction or loss of antibacterial activity against *E. coli*, *A. baumannii*, and *P. aeruginosa*. Notably, compounds **64**–**66**, **72**, and **73** retained activity against wild-type *E. coli* (Table S9) and compounds **64**–**68**, **72**, and **73** were active against wild-type *S. aureus* (Table S10). In comparison to **27**, compounds bearing the morpholinomethyl moiety and its surrogates in the *para* position of the phenyl ring retained or slightly increased the inhibition of gyrase from *A. baumannii* and *P. aeruginosa* but were weaker inhibitors of topo IV. The inhibition of both *E. coli* enzymes followed the opposite trend with weaker DNA gyrase inhibition and stronger inhibition of topo IV. To our satisfaction, the free fraction was substantially increased for compounds **64** (mouse: 3.25%), **67** (human: 0.48%; mouse: 1.7%), and **68** (human: 1.7%; mouse: 5.0%), and for compound **71** the thermodynamic solubility was strongly increased to 2760 μmol/L. These results indicate that increasing solubility and free fraction to acceptable levels is possible within the *p*-morpholinomethyl structural class, although combining good solubility and free fraction with the desired antibacterial activity against *P. aeruginosa* and *A. baumannii* remains a challenge.

### 2.2 Physicochemical and ADMET Properties

#### 2.2.1 p*K*_a_ and log *D*

As a part of compound **27** profiling, its p*K*_a_ (p*K*_a1_ = 4.30 ± 0.10; p*K*_a2_ = 8.20 ± 0.01) and log *D* (4.28 at pH 7.4) were determined. Because the substitution adjacent to the 6-carboxylic acid group was not varied, it can be assumed that also other analogs possess a similar p*K*_a1_ value (e.g., compound **59** has experimentally determined p*K*_a1_ 3.94 ± 0.02 and p*K*_a2_ 6.38.20 ± 0.06). The measured log *D* (pH 7.4) for the further 10 compounds was between 1.5 and 4.56 (Table 6). The effect of pyrrole substituents on log *D* in α-methyl compounds **27**, **50**–**53**, and **55**, and in the α-morpholinomethyl series (compounds **36**, **59**, and **60**) was examined in more detail. A clear trend was observed that, surprisingly, 3,4-dichloro derivatives are less lipophilic than the respective 4-monochloro derivatives (cf. compounds **27** and **50** in the α-methyl series and compounds **36** and **59** in the α-morpholinomethyl series). The decrease of log *D* by 1.6 due to the introduction of a morpholinomethyl group to the stereogenic carbon atom is evident by comparing pairs of compounds **27** and **36**, **50** and **59** as well as **51** and **60**. Introduction of a morpholinomethyl moiety to the *para* position of the phenyl ring of **52** to obtain **68** decreased the log *D* value by 0.47. Whereas the decrease in log *D* by the fluoro and cyano substitutions of the pyrrole ring is well evident, the decrease in log *D* of **27** coincided with good antibacterial activity only in compound **51** in the α-methyl series and in **36** in the α-morpholinomethyl series.

#### 2.2.2 Kinetic and Thermodynamic Solubility

The kinetic solubility of all final compounds was routinely assessed, and for compounds possessing promising antibacterial activity also thermodynamic solubility was measured. In the kinetic solubility assay, a small volume of DMSO solution of the test compound was added to the aqueous buffer whereupon after an incubation period the concentration was determined before equilibrium was reached.^32^ The kinetic solubility assay mimics the conditions of in vitro biological tests, and their purpose was to identify compounds that do not have good kinetic solubility even in aqueous buffer containing DMSO, and the results obtained were used to guide the modification of structures to improve solubility. For the determination of thermodynamic solubility that varies with the crystal form of the compound, water or buffer was added directly to solid crystalline material and solubility was measured after equilibrium was established between the dissolved and solid compounds. The kinetic and thermodynamic solubility data are reported in Tables 1, 2, 5, and S3–S9 and discussed there in relation to the chemical structure of the compounds. Solubility data for most representative compounds with activity against *A. baumannii*, together with the fraction of compounds not bound to plasma proteins, are compiled in Table 7. Among them, compound **71** has the best kinetic and thermodynamic solubility but, unfortunately, it did not possess significant antibacterial activity.

**Table 7:** Kinetic and Thermodynamic Solubility Data of Representative Compounds with Antibacterial Activity

					free fraction [%]	
Cpd.	log *D* Calc.	log *P* Calc.	kinetic solub. [μM]	thermodyn. solub. [μM]	human	mouse
**1**	2.1	5.8	12	6.6	<0.1	<0.1
**27**	2.59	5.94	>80	92.4	0.016	0.01
**36**	2.26	2.74	35	3	<0.1	0.3
**51**	1.52	4.87	41	18	0.13	0.3
**59**	1.66	2.14	20	70	<0.1	0.5
**65**	2.56	2.74	30	25	<0.1	<0.1
**71**	1.12	2.21	59	2760	0.11	0.2

#### 2.2.3 Plasma Protein Binding

Increasing the fraction of compounds not bound to plasma proteins while maintaining their activity against *A. baumannii* and *P. aeruginosa* was the main objective of structural modification of the initial hit **1**. Chemical strategies to reduce plasma protein binding included decreasing the lipophilicity, increasing the polar surface area and, in some cases, increasing the basicity of compounds. As demonstrated in Table S11, the free fraction was increased to a few percent in several derivatives of compounds **1** and **27**, which all displayed low nanomolar inhibition of gyrase and topo IV of *E. coli*, *A. baumannii*, and *P. aeruginosa* but were, unfortunately, devoid of antibacterial activity. For instance, the comparison of **27** with compounds **32** and **64**, both devoid of antibacterial activity but giving a decent free fraction in mouse plasma, demonstrates that the methylsulfonyl group increased the free fraction in mouse plasma by a few percent irrespective of its attachment position. With compounds **27**, **36**, and **51**, which possessed antibacterial activity, we succeeded in decreasing plasma protein binding to some extent but it remained lower than 1%. It seems that there is a narrow window of physicochemical properties within the structural class of our gyrase inhibitors, which is compatible with good permeability and low efflux rate in bacteria as well as with acceptable solubility and plasma protein binding in human plasma. Such properties have not been reached with the compounds synthesized so far, and reaching them would require a comprehensive multiparameter optimization approach.

#### 2.2.4 Genotoxicity and Mutagenic Potential

The initial hit **1** with a 97% purity was found to be genotoxic and able to induce chromosome breaks at a 20 μM concentration in in vitro cell micronucleus test (MNT). The hydrolyzed aromatic amine precursor, 2-amino-4-(phenoxymethyl)benzo[*d*]thiazole-6-carboxylic acid, which was the main impurity (1.5%) in the final product, was deemed responsible for the genotoxicity of **1**, but surprisingly it was found nongenotoxic in the MNT assay at a 500 μM concentration. However, 98.8% pure compound **1** did not show genotoxicity at concentrations up to 25 μM in the micronucleus test, which led to the conclusion that the observed genotoxicity of **1** very likely originated from another unidentified compound present in the remaining 1.5% impurities. Compound **1** was also found to be nonmutagenic up to 25 μM in the absence or presence of S9 metabolic activation against *S. typhimurium* TA98, as confirmed by the AMES test (Figure S9). Compound **18**, a carboxamide *N-*methyl derivative of **1**, was also found to be nongenotoxic at 37 μM in the MNT assay. (Figure S7), suggesting that the carboxamide N-alkylation could be a viable strategy for structure modification. Compound **27** was found nongenotoxic up to 75 μM, both in a metabolic-activated system and without metabolic activation as concluded from the in vitro MNT assay (Figure S8). In the AMES test, the compound was found nonmutagenic up to 25 μM in both the presence and absence of S9 metabolic activation. With metabolic activation, it showed the possible cytotoxic effect (against *S. typhimurium*) above 3 μM concentration (Figure S10). Also, the hydrolyzed precursor, 2-amino-4-(1-phenoxyethyl)benzo[*d*]thiazole-6-carboxylic acid, was neither genotoxic in the MNT test (assayed up to 350 μM) nor mutagenic (assayed in the AMES test up to a 25 μM concentration against *S. typhimurium* TA98 and TA100) in both the absence or presence of S9 metabolic activation. These results went hand in hand with better antibacterial activity and corroborated **27** as an improved derivative of the initial hit **1** worth of further structural optimization. Microbiologically promising derivatives **31**, **51**, and **59** as well as the hydrolyzed aromatic amine precursor of **31**, 2-amino-4-(2-methoxy-1-phenoxyethyl)benzo[*d*]thiazole-6-carboxylic acid, were also found nongenotoxic when tested in the MNT assay with and without metabolic activation. In the AMES test, **31** was found to be nonmutagenic against *S typhimurium* TA98 without metabolic activation at 25 μM, whereas with metabolic activation a possible cytotoxic effect was observed above 3 μM (Figure S11). Its hydrolyzed aromatic amine precursor did not show mutagenicity up to 25 μM concentration when tested with and without metabolic activation against *S. typhimurium* TA98 and TA100 in AMES assays. In conclusion, the presented results showed that the benzothiazole-2-amine precursors, as potential impurities in the gyrase inhibitors, presented in this paper do not possess an inherent genotoxic or mutagenic character.

#### 2.2.5 Mitochondrial Toxicity

Parallel to the chemical modification of the original hit **1**, the mitochondrial toxicity of the new derivatives and analogs was monitored. Mitochondrial toxicity was tested in vitro using HepG2 cells cultured in either glucose or galactose. When cells are exposed to galactose, they are forced to generate ATP using the mitochondria, rather than glycolysis, which is the case for cells in glucose medium. If a compound inhibits the mitochondrial function, then ATP levels will fall more drastically in galactose- than in glucose-exposed cells and cell death occurs at lower concentrations in galactose-exposed cells.^33^ Among 30 tested compounds, all were free of mitochondrial toxicity at 100 μM and most of them with the exception of **1**, **9**, **18**, **28**, **31**, **35**, **36**, **46**, and **47** were not toxic to mitochondria even at 300 μM. Whereas **1** was toxic at 300–1000 μM and showed cytotoxicity in glucose medium below 100 μM, **27** (Figure S5) and its enantiomers (*R*)-**27** and (*S*)-**27**, the monofluoro derivative **51** and its both enantiomers (*R*)-**51** and (*S*)-**51**, the *para*-substituted derivatives **69** and **72**, as well as *N*-dimethylamino derivative **34** were not toxic to mitochondria up to 1000 μM and not cytotoxic in glucose medium at 100 μM. In summary, the mitochondrial toxicity testing highlighted compounds **27** and **51** and their enantiomers, *para*-substituted compounds **69** and **72** as well as compound **34** bearing a simple tertiary amine attached to the methine carbon at the branching point as most promising.

#### 2.2.6 Metabolic Stability in Human and Mouse Hepatocytes and Microsomes

In vitro metabolic stability assay in cryopreserved hepatocytes was used to evaluate the metabolic stability of compounds representative of different structure classes in human and mouse hepatocytes. The assay closely resembles the conditions of in vivo liver cells, with all isoforms of metabolizing enzymes, cofactors, cellular components, and membrane permeation mechanisms.^34^ Compound **1** was stable in human (*t*_1/2_ = 71 min) and mouse hepatocytes (*t*_1/2_ = 132 min) (Figure S13), and **27** possessing potentially metabolically liable α-methyl group also remained relatively stable in human (*t*_1/2_ = 92 min) and mouse hepatocytes (*t*_1/2_ = 45 min) (Figure S14). Lower metabolic stability in mouse hepatocytes was observed for monofluoro compound **51** (*t*_1/2_ = 11 min) and its enantiomer (*S*)-**51** (*t*_1/2_ = 21 min), monochloro derivative **59** (*t*_1/2_ = 14 min) and α-hydroxymethyl derivative **30** (*t*_1/2_ = 26 min), suggesting that compounds possessing monohalogenated pyrrole moiety or polar substituent on the α-methyl group are more likely to be metabolized in hepatocytes. Microsomal stability assay using human and mouse liver microsomes and NADPH cofactor to assess phase I oxidations by cytochrome and flavin monooxygenases was applied to a broader set of compounds. In the human liver microsome assay, higher metabolic stability (*t*_1/2_ > 100 min) was observed for *N*-alkyl compounds **19**, **23**, and **24**, as well as for compounds **8**, **64**, and **73** bearing a *para* substituent on the phenyl ring. Lower metabolic stability was inherent to less substituted pyrroles, e.g., **51**, (*S*)-**51**, and **53**, as well as to α-hydroxymethyl derivative **30**. In mouse liver microsomes, no general trends could be observed for the tested compounds.

#### 2.2.7 Caco-2 Permeability Study of Compound **27**

Caco-2 permeability assay for compound **27** indicated its high permeability in the apical direction [*P*_app_ (A–B) = (2.9 ± 0.6) × 10^–6^ cm/s; *P*_app_ (B–A) = 9.9 ± 1.2 × 10^–5^ cm/s; efflux ratio = 35], which might be caused by *P*-glycoprotein and may limit its oral bioavailability and permeation through the blood–brain barrier.

#### 2.2.8 Relationship between Collected Physicochemical Data and In Vitro Antibacterial Activity

**Figure 5:**
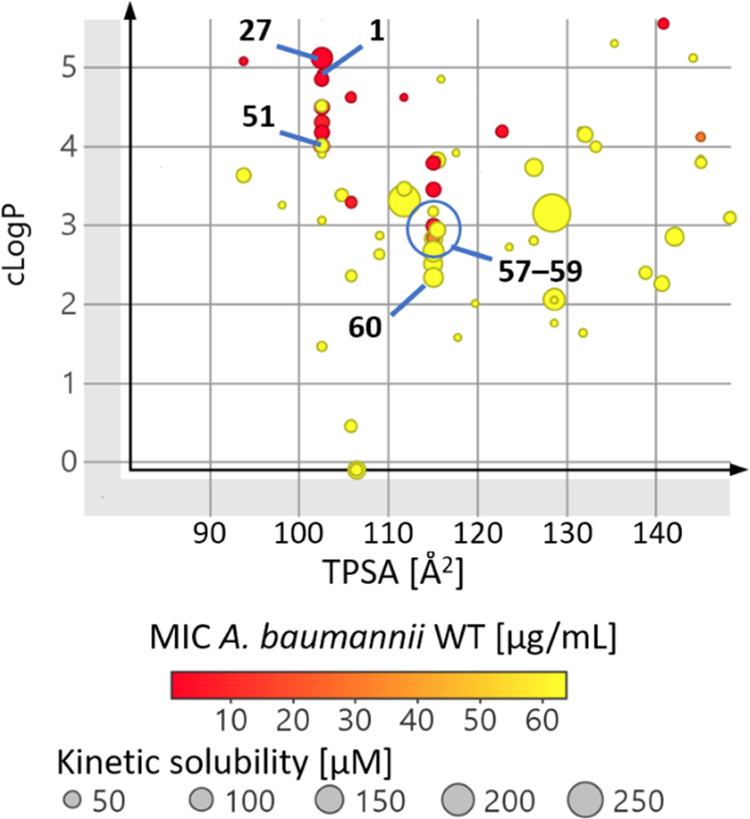
Relationship between clog *P*, TPSA, kinetic solubility, and MIC against *A. baumannii*.

The analysis of collected lipophilicity, solubility, and plasma protein binding data could provide useful guidance to the physicochemical thresholds required to obtain satisfactory MIC values. Calculated octanol/water partition coefficient (clog *P*) and total polar surface area (TPSA) were the major molecular descriptors guiding the design of improved analogs of compounds **1** and **27** (Figure 5). Analogs active against *A. baumannii* lay mostly around two major TPSA values, the initial TPSA of 104 Å^2^ and the final value of 117 Å^2^ (after introduction of morpholine moiety). Pushing down clog *P* at a fixed TPSA resulted in compounds with improved solubility and appreciable plasma free fraction (>0.1% in our case). For example, compound **51** retained good activity against *A. baumannii* (MIC = 2 μg/mL), and its solubility and free fraction were improved relative to **1**. Compounds **57**–**59** seemingly represent the limit of polarity for this structural class of gyrase inhibitors to retain activity against *A. baumannii*. Compound **60** is a borderline example with retained activity against *P. aeruginosa* (Figure S15) but not against *A. baumannii*. Its kinetic solubility is 67 μM, and its unbound fraction in human and mouse plasma is 1.5 and 4.4%, respectively. On the other hand, targeting the Gram-positive bacteria, for example, *S. aureus* (Figure S16), with the reported benzothiazole scaffold-based DNA gyrase inhibitors is an easier task. A much broader clog *P*/TPSA chemical space is tolerated; see, for example, compound **33** with a kinetic solubility of 90 μM and a plasma free fraction of 0.28 and 0.53%.

### 2.3 Chemistry

**Scheme 1:**
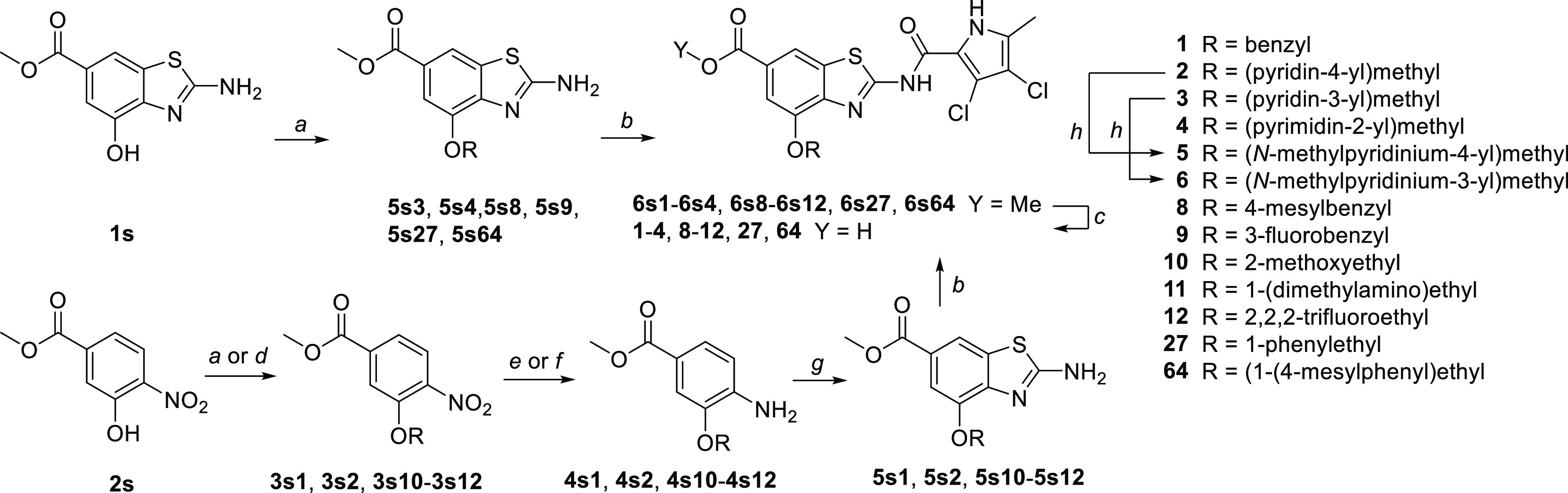
Synthesis of 4-Alkoxy Derivatives Reagents and conditions: (a) benzylic halide, K_2_CO_3_, KI (cat.), MeCN or DMF, 60 °C, 18 h; (b) 3,4-dichloro-5-methyl-1*H*-pyrrole-2-carbonyl chloride, toluene, 130 °C, 15 h; (c) 2 M NaOH, MeOH, 40 °C, 48 h; (d) ROH, PPh_3_, DIAD, THF, 22 °C, 18 h; (e) for benzylic ethers: Fe, AcOH, rt, 2 h; (f) for nonbenzylic ethers: H_2_, Pd/C, MeOH, 22 °C, 5 h; (g) KSCN, Br_2_, AcOH, rt, 15 h; and (h) iodomethane, THF/DMF, 60 °C, 18 h.

4-Hydroxybenzothiazole **1s**(35) was successfully alkylated with most benzylic halides of interest to get compounds **5s3**, **5s4**, **5s8**, **5s9**, **5s27**, and **5s64** together with *N*- and bis-alkylated products. The desired O-alkylated products were isolated in 13–38% yields by column chromatography and/or trituration with ethyl acetate. Because alkylation of **1s** with 4-(bromomethyl)pyridine and some other nonbenzylic halides failed, we opted for alkylation of the more nucleophilic *o*-nitrophenol **2s** either via nucleophilic substitution of alkyl halides or via Mitsunobu reaction to obtain **3s1**, **3s2**, and **3s10**–**3s12**. These were reduced to anilines **4s1**, **4s2**, and **4s10**–**4s12** by catalytic hydrogenation or (in the case of benzylic ether **3s1**) by iron/acetic acid. Benzothiazoles **5s1**, **5s2**, and **5s10**–**5s12** were obtained following our general protocol using potassium thiocyanate and bromine in glacial acetic acid.^35^ 2-Aminobenzothiazoles obtained via either pathway were *N*-acylated with 4-dichloro-5-methyl-1*H*-pyrrole-2-carbonyl chloride. Alkaline hydrolysis of the resulting methyl esters **6s1**–**6s4**, **6s8**–**6s12**, **6s27**, and **6s64** afforded the initial hit **1** and analogs **2**–**4** and **8**–**12**, **27**, and **64**. Pyridines **2** and **3** were further quaternized with iodomethane to obtain *N-*methylpyridinium compounds **5** and **6** (Scheme 1).

**Scheme 2:**
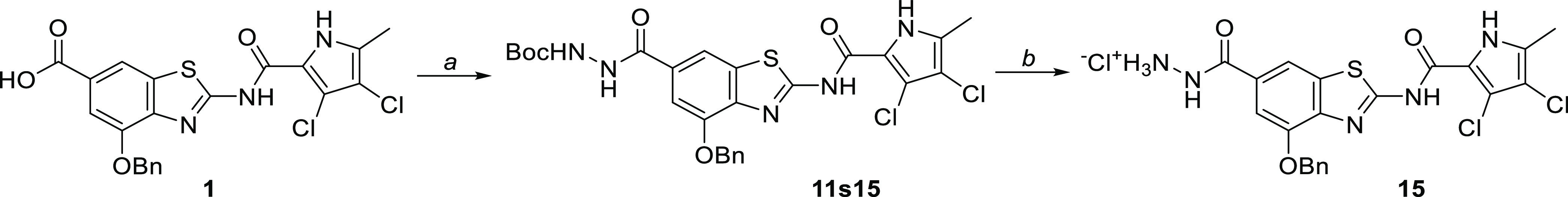
Synthesis of Hydrazide **15** Reagents and conditions: (a) *tert*-butyl carbazate, TBTU, DMF, *N*-methylmorpholine, 22 °C, 8 h; (b) 4 M HCl in 1,4-dioxane, 22 °C, 24 h.

**Scheme 3:**
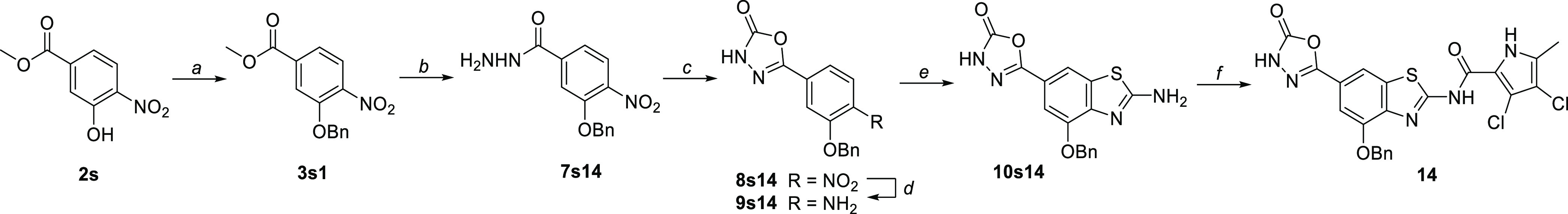
Synthesis of Oxadiazolone Isostere **14** Reagents and conditions: (a) benzyl bromide, K_2_CO_3_, MeCN, 60 °C, 3 h; (b) hydrazine monohydrate, EtOH, reflux, 48 h; (c) CDI, 1,4-dioxane, 100 °C, 15 h; (d) Fe, AcOH, 22 °C, 1 h; (e) KSCN, Br_2_, AcOH, 0–22 °C, 15 h; and (f) 3,4-dichloro-5-methyl-1*H*-pyrrole-2-carbonyl chloride, pyridine, CH_2_Cl_2_, 22 °C, 15 h.

Hydrazide **15** was prepared via a late-stage coupling of the carboxylic acid **1** and *tert*-butyl carbazate, followed by Boc-deprotection of the obtained **11s15** (Scheme 2). We were not able to convert **15** to oxadiazolone **14** after repeated experiments, so we resorted to a lengthier synthetic route. The oxadiazolone moiety was constructed via hydrazinolysis of ester **3s1**, followed by carbonylation using carbodiimidazole (CDI). Intermediate **8s14** was then converted to benzothiazole **14** following the reaction sequence outlined in Scheme 3. Compounds **13**,^16^ a tetrazole isostere of carboxylic acid **1**, and **16**,^36^ an aminomethyl derivative of the initial hit **1**, were prepared as previously described.

**Scheme 4:**
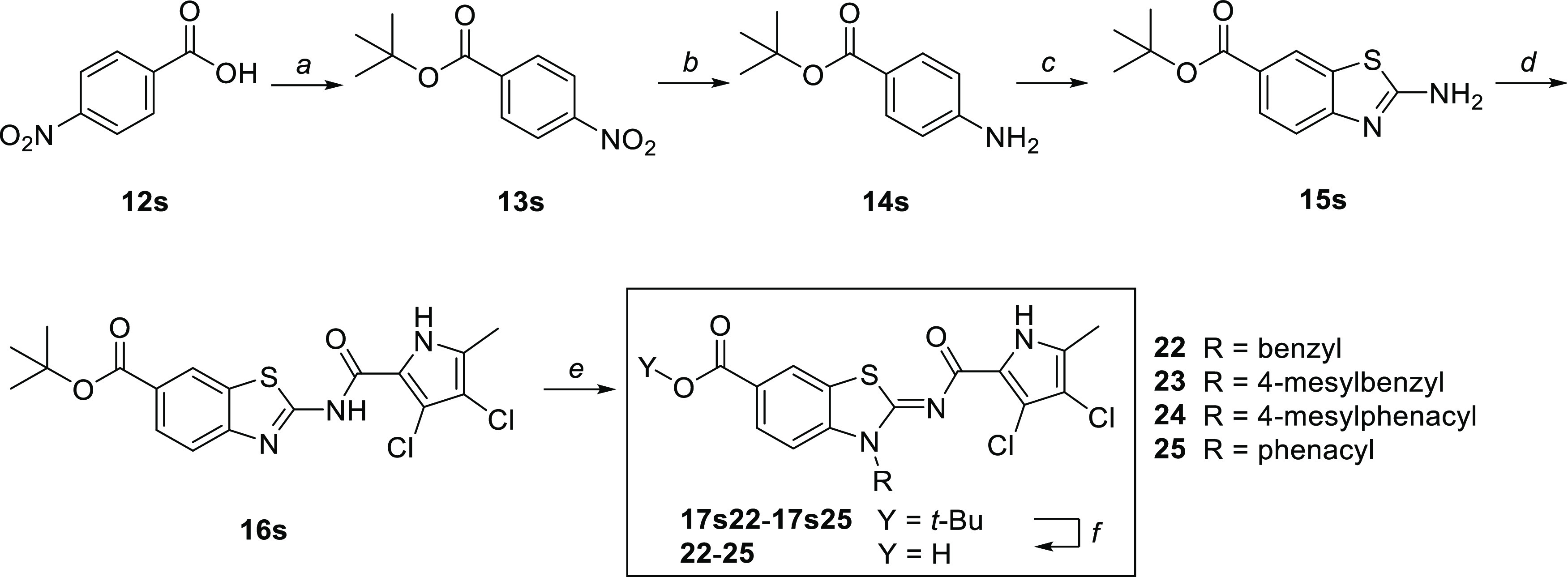
Synthesis of Thiazole-N-Alkylated Derivatives Reagents and conditions: (a) TsCl, *t*-BuOH, pyridine, 0 °C to 22 °C, 24 h; (b) H_2_, Pd/C, EtOAc, 2 h; (c) KSCN, Br_2_, AcOH, 0–22 °C, 24 h; (d) 3,4-dichloro-5-methyl-1*H*-pyrrole-2-carbonyl chloride, Et_3_N toluene, 130 °C, 15 h; (e) alkyl halide, KI, NaHCO_3_, DMF, 60 °C, 14 h; and (f) 1 M HCl in acetic acid, 22 °C, overnight.

Our attempt to alkylate the carboxamide nitrogen atom of **16s** with benzyl bromide using NaHCO_3_ as a base afforded a monoalkylated product for which ^1^H–^1^H NOESY experiment revealed that the regioselective benzylation occurred at the thiazole nitrogen, giving rise to **17s22** in which the 2-aminobenzothiazole scaffold is locked in its imino tautomer. As demonstrated in a systematic study,^37^ the observed benzothiazole N-alkylation appears to be general for reactions with benzyl- and phenacyl halides and enables the preparation of thiazole-N-alkylated products **17s22**–**17s25** that after deprotection under acidic conditions afforded compounds **22**–**25** (Scheme 4). Using the same approach, alkylation of intermediate **16s** with methyl iodide in a pressure tube at 60 °C was not successful. Therefore, we searched for a method that would allow selective access to carboxamide N-alkylated products.

**Scheme 5:**
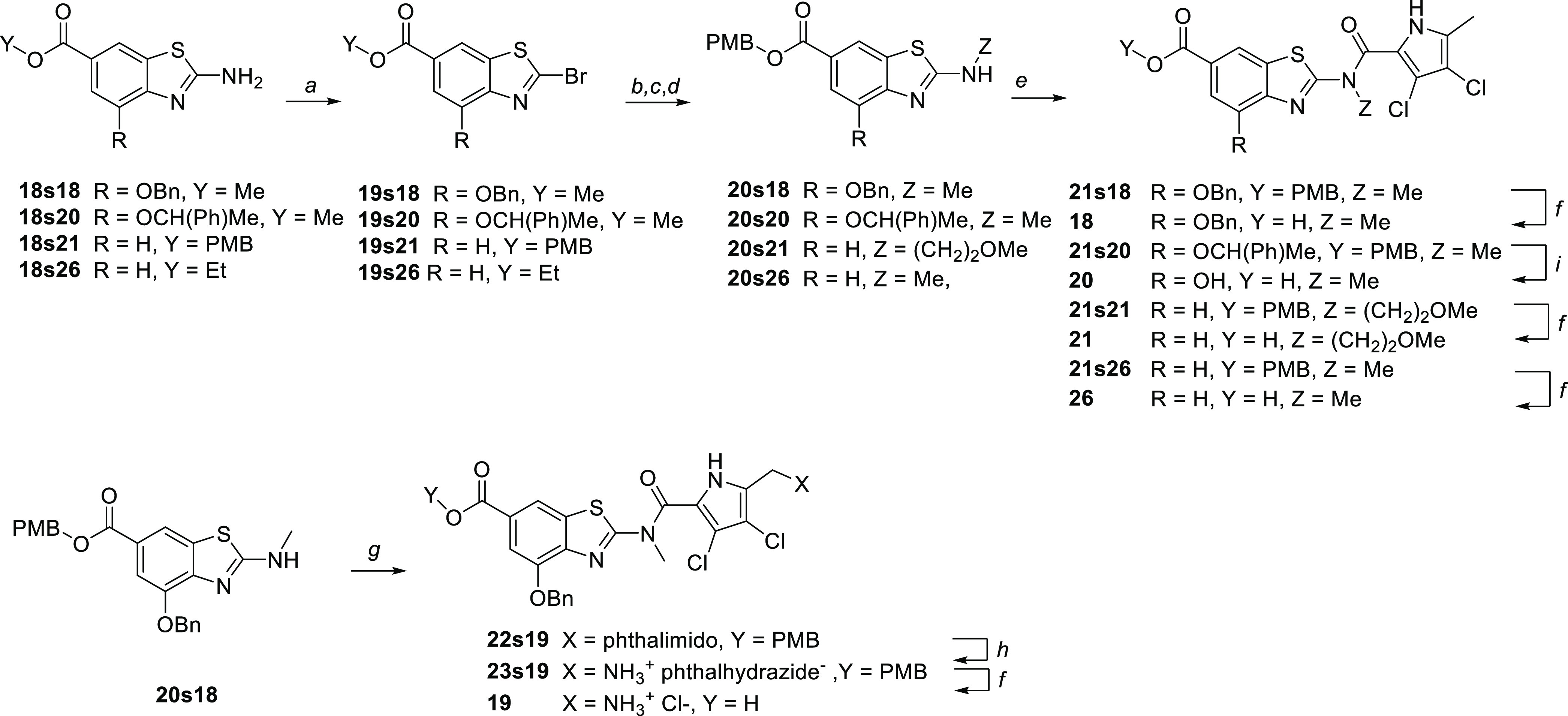
Synthesis of Carboxamide N-Alkylated Derivatives Reagents and conditions: (a) CuBr_2_, *t*-BuONO, CH_3_CN, 22 °C, 15 h; (b) Z-NH_2_, THF, 22 °C, 15 h; (c) 2 M NaOH, 1,4-dioxane, 50 °C, 24 h (for **20s18**, **20s20**, **20s26**); (d) *p*-methoxybenzyl chloride, K_2_CO_3_, DMF, rt, 15 h (for **20s18**, **20s20**, **20s26**); (e) 3,4-dichloro-5-methyl-1*H*-pyrrole-2-carbonyl chloride, toluene, 130 °C, 15 h; (f) 1 M HCl in AcOH, 22 °C, 18 h; (g) 3,4-dichloro-5-phthalimidomethyl-1*H*-pyrrole-2-carbonyl chloride,^36^ toluene, 130 °C, 15 h; (h) (1) hydrazine hydrate, EtOH, 50 °C, 40 min; (2) HCl, MeOH, 22 °C, 15 min; (3) EtOH, reflux, 18 h; and (i) SnCl_4_, DCM, 22 °C, 2 h.

Compounds **18**, **20**, **21**, and **26** possessing an *N*-alkyl carboxamide substituent were prepared according to the reaction sequence presented in Scheme 5. The starting 2-aminobenzo-thiazoles **18s18**, **18s20**, **18s21**, and **18s26** were first transformed to 2-bromobenzothiazoles **19s18**, **19s20**, **19s21**, and **19s26** via the Sandmeyer reaction. Methylamine or 2-methoxyethylamine was then applied to substitute bromine and give the corresponding N-substituted 2-aminobenzothiazoles, which were transformed into *p*-methoxybenzyl (PMB) esters **20s18**, **20s20**, **20s21**, and **20s26** and then coupled to 3,4-dichloro-5-methyl-1*H*-pyrrole-2-carboxylic acid. A switch from methyl/ethyl to PMB esters was required because tertiary amides resulting from the coupling of the alkyl esters were unstable during the final alkaline hydrolysis, as opposed to the analogous secondary amides. After acidolytic cleavage of the resulting PMB esters, the carboxylic acids **18**, **21**, and **26** were obtained. It is noteworthy that the 1-phenylethyl substituent was cleaved during the acidolysis step affording the 4-hydroxy analog **20** instead of the desired 4-(1-phenylethoxy) derivative. Using the same strategy together with the phthalimido protection, the methylated aminomethyl derivative **19** was obtained from **20s18**.

**Scheme 6:**
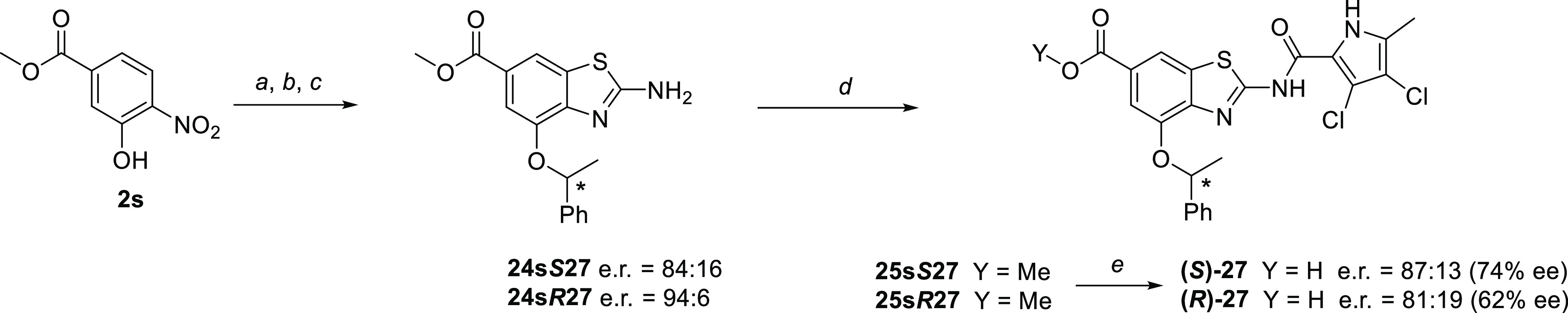
Synthesis of Compound **27** and its Enantiomers Reagents and conditions: (a) for (***S***)-**27**: (*R*)-1-phenylethanol, for (***R***)-**27**: (*S*)-1-phenylethanol, PPh_3_, DIAD, THF, 22 °C, 18 h; (b) Fe, AcOH, 22 °C, 2 h; (c) KSCN, Br_2_, AcOH, 22 °C, 15 h; (d) 3,4-dichloro-5-methyl-1*H*-pyrrole-2-carbonyl chloride, toluene, 130 °C, 15 h; and (e) 2 M NaOH, MeOH, 40 °C, 48 h.

For the synthesis of compound **27**, building block **5s27** was prepared from phenol **1s** and 1-chloro-1-phenylethane. Coupling of **5s27** with the pyrrole moiety and subsequent ester hydrolysis afforded **27** (Scheme 1). Starting from **2s** and the commercially available (*R*)- or (*S*)-1-phenylethanol, the enantiomerically enriched (*S*)-**27** and (*R*)-**27** (74 and 62% ee, respectively, determined by chiral HPLC analysis) were obtained in a 5-step reaction procedure with the inversion of the absolute configuration in the Mitsunobu reaction step (Scheme 6). Partial racemization most likely occurred during oxidative benzothiazole formation step (step c) or during coupling to acyl chloride (step d) via cleavage–reattachment of the 1-phenylethyl substituent through the carbocation intermediate.

**Scheme 7:**
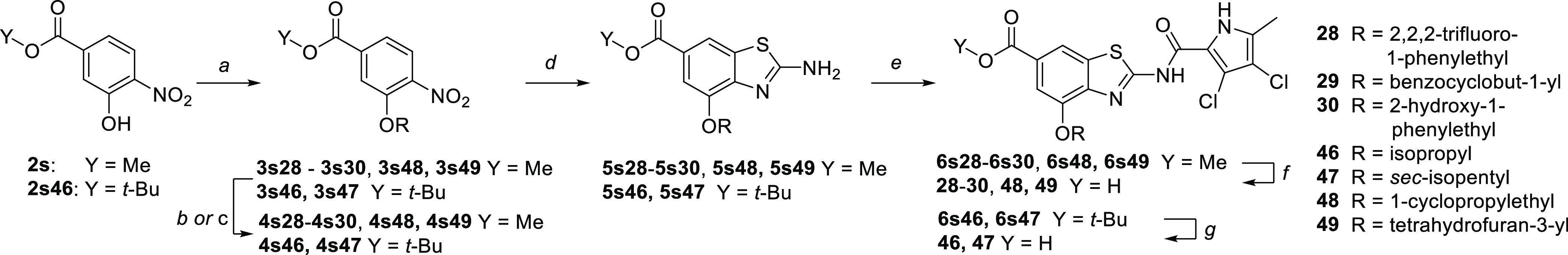
Synthesis of Branched 4-Alkoxy Analogs Reagents and conditions: (a) ROH, PPh_3_, DIAD, THF, 22 °C 18 h; (b) Fe, AcOH, 22 °C, 2 h; (c) H_2_, Pd/C, MeOH, 22 °C, 5 h; (d) KSCN, Br_2_, AcOH, 22 °C, 15 h; (e) 3,4-dichloro-5-methyl-1*H*-pyrrole-2-carbonyl chloride, toluene, 130 °C, 15 h; (f) 2 M NaOH, MeOH, 40 °C, 48 h; and (g) CF_3_COOH, dichloromethane, 22 °C, 12–30 h.

Stimulated by the improved antibacterial profile of compound **27**, 4-alkoxy analogs **28**, **29**, and **46–49** with variations at the stereogenic carbon atom were prepared, as outlined in Scheme 7. Alkylation of the phenol **2s** was again achieved using the Mitsunobu reaction and the commercially available alcohol building blocks. Either iron/acetic acid or catalytic hydrogenation was used for the reduction of the nitro group, and standard coupling and deprotection strategies were applied. Protection of the hydroxyl group as a benzoate ester was applied for the synthesis of **30**.

**Scheme 8:**
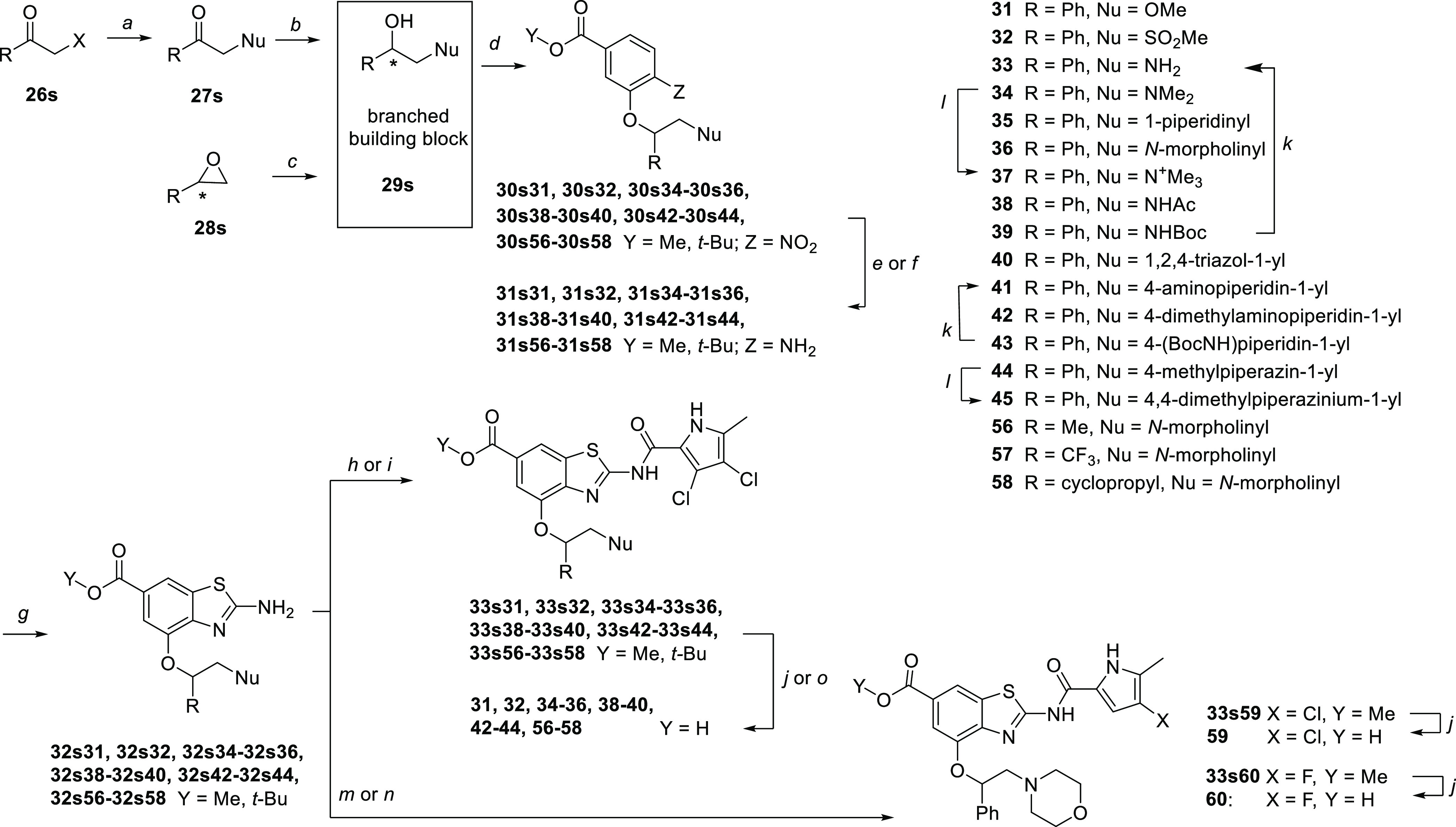
Synthesis of Branched 4-Alkoxy Analogs Containing a Polar Group Reagents and conditions: (a) nucleophile, K_2_CO_3_, KI, MeCN, 22 °C, 24 h; (b) NaBH_4_, MeOH, 0–22 °C, 2 h; (c) nucleophile, DMF or neat, heating; (d) **2s**, PPh_3_, DIAD, THF, 22 °C, 18 h; (e) Fe, AcOH, 22 °C, 2 h; (f) H_2_, Pd/C, MeOH, 22 °C, 5 h; (g) KSCN, Br_2_, AcOH, 22 °C, 15 h; (h) 3,4-dichloro-5-methyl-1*H*-pyrrole-2-carbonyl chloride, toluene, 130 °C, 15 h; (i) 2-trichloroacetyl-3,4-dichloro-5-methyl-1*H*-pyrrole, Na_2_CO_3_, DMF, 65 °C, 24 h; (j) 2 M NaOH, MeOH, 40 °C, 48 h; (k) 1 M HCl in 1,4-dioxane, 22 °C, 24 h; (l) MeI, THF, 60 °C, 24 h; (m) 2-trichloroacetyl-4-chloro-5-methyl-1*H*-pyrrole, Na_2_CO_3_, DMF, 65 °C, 18 h; (n) 4-fluoro-5-methyl-1*H*-pyrrole-2-carbonyl chloride, toluene, 130 °C, 18 h; and (o) CF_3_COOH, dichloromethane, 22 °C, 18 h.

A general strategy for the introduction of polar substituents to the methylene group at the branching point relied on the nucleophilic substitution of α-haloketones **26s** and the subsequent NaBH_4_-mediated reduction of the resulting ketones **27s** or anti-Markovnikov opening of epoxides **28s**. The etherification of the obtained alcohols **29s** with phenol **2s** was achieved via the Mitsunobu reaction, and the target compounds **31**–**45** and **56–58** were obtained after coupling to 3,4-dichloro-5-methylpyrrole-2-carbonyl chloride and ester cleavage. 2-Aminobenzothiazole **32s36**, containing morpholinomethyl moiety, was also coupled to monochloro- and monofluoropyrrole building blocks to obtain compounds **59** and **60**, analogs of **36** (Scheme 8).

**Scheme 9:**
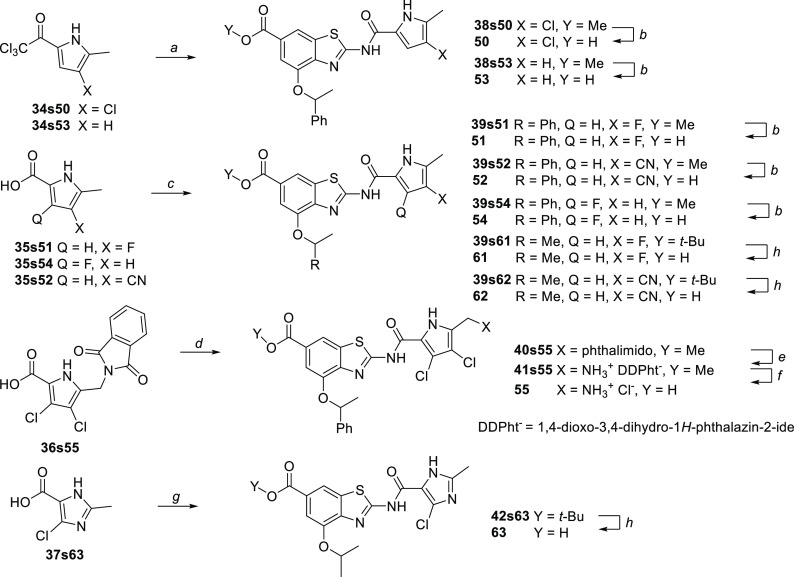
Synthesis of Derivatives Containing Less Lipophilic Pyrrole Moieties Reagents and conditions: (a) **24s27**, Na_2_CO_3_, DMF, 65 °C, 18 h; (b) 2 M NaOH, MeOH, 40 °C, 48 h; (c) oxalyl chloride, CH_2_Cl_2_, 22 °C, 24 h; then **24s27**, toluene, 130 °C, 24 h; (d) SO_2_Cl_2_, 75 °C, 1 h; then **24s27**, toluene, 130 °C, 24 h; (e) hydrazine hydrate, EtOH, 40 °C, 40 min; then HCl, MeOH, 22 °C, 15 min; then EtOH, reflux, 24 h; (f) 2 M NaOH, MeOH, 50 °C, 48 h; then HCl, MeOH; (g) SOCl_2_, DMF (cat.), 75 °C, 1.5 h; then *tert*-butyl 2-amino-4-isopropoxybenzo[*d*]thiazole-6-carboxylate (**42s63**), toluene 130 °C, 18 h; and (h) CF_3_CO_2_H, CH_2_Cl_2_, 22 °C, 18 h.

Being aware of the contribution of the dichloropyrrole moiety to the forbiddingly high lipophilicity of compound **27**, we prepared analogs **50**–**55** and **61**–**63** featuring a less lipophilic pyrrolamide moiety. The required pyrrole building blocks **34s50**, **35s51**, **35s54**, **36s55**,^36^**34s53**,^38^ and **35s52**(39,40) were prepared according to the published procedures, and the 4-chloro-2-methyl-1*H*-imidazole-5-carboxylic acid (**37s63**) was commercially available. Either 2-(trichloroacetyl)pyrroles or pyrrole-2-acyl chlorides were used as acylating agents for coupling to the 2-aminobenzothiazole building block **5s27**. Notably, the amide bond formed by the coupling of 2-aminobenzothiazole **5s27** and carboxylic acid **37s63** was not stable under the alkaline conditions required for the final-step methyl ester hydrolysis. When the *tert*-butyl ester protection was used instead, the 1-phenylethyl ether was found unstable under acidolytic conditions required for the *tert*-butyl ester cleavage. To avoid these restrictions, the 4-isopropoxy analog **63** was prepared from the corresponding *tert*-butyl ester building block **42s64** (Scheme 9).

**Scheme 10:**
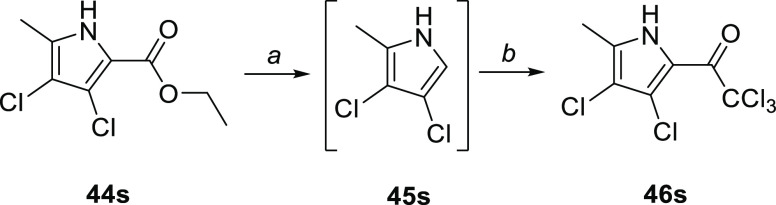
Synthesis of 2-Trichloroacetyl-3,4-dichloro-5-methyl-1*H*-pyrrole Reagents and conditions: (a) 0.5 M KOH, reflux, 4 h; (b) Cl_3_CCOCl, 1,2-dichloroethane, 22 °C, 18 h.

We found that compounds containing an aliphatic tertiary amine in the side chain, such as **34** and **35**, were not accessible via acylation of intermediates **32s34** and **32s35** with 3,4-dichloro-5-methyl-1*H*-pyrrole-2-carbonyl chloride. For example, in an attempted acylation of piperidine-containing intermediate **32s35** using 2.2 equivalents (in two portions) of the aforementioned acyl chloride, merely 10% conversion was achieved. This goes hand in hand with our general observation that the use of tertiary amine or pyridine was detrimental to the yield of coupling of acyl chlorides to 2-aminobenzothiazoles. An alternative 2-trichloroacetylpyrrole-based acylating agent **46s** was thus prepared, which in analogy to 2-trichloroacetyl-4,5-dibromo-1*H*-pyrrole^11^ should operate under milder alkaline reaction conditions. Because efficient direct dichlorination of 2-trichloroacetyl-5-methyl-1*H*-pyrrole failed using *N*-chlorosuccinimide or sulfuryl chloride, a two-step protocol from ester **44s** was developed. Alkaline hydrolysis accompanied by decarboxylation afforded compound **45s**, which was prone to oxidation and was therefore immediately used in the Friedel–Crafts acylation step to afford trichloroacetylpyrrole **46s** (Scheme 10).

**Scheme 11:**
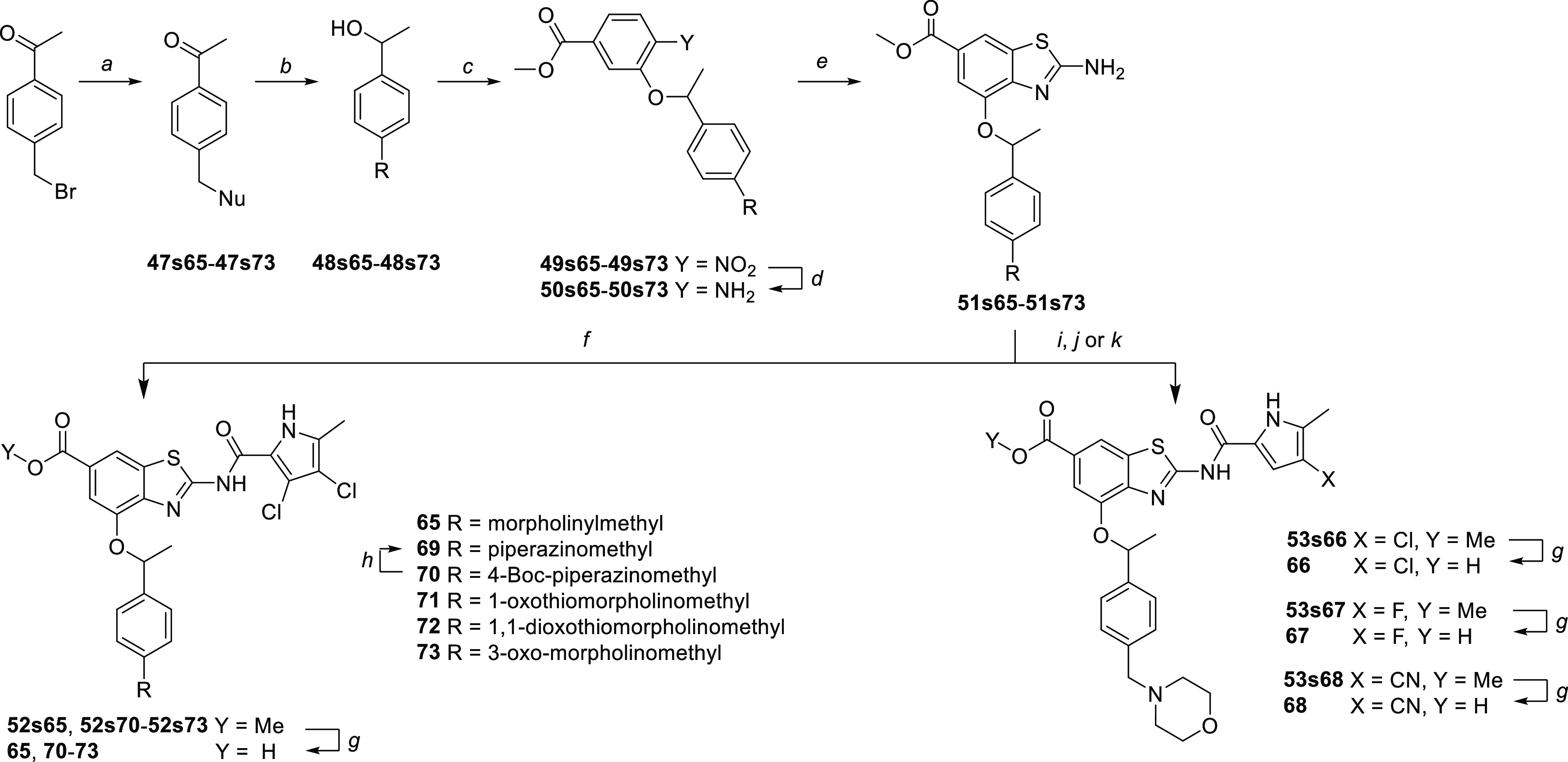
Synthesis of Derivatives with a *para* Substituent on the Phenyl Ring Reagents and conditions: (a) nucleophile, K_2_CO_3_, MeCN, 0–22 °C, 24 h. (b) NaBH_4_, 0–22 °C, 2 h; (c) **2s**, PPh_3_, DIAD, THF, 22 °C, 18 h; (d) Fe, AcOH, 22 °C, 2 h; (e) KSCN, Br_2_, AcOH, 22 °C, 15 h; (f) **46s**, Na_2_CO_3_, DMF, 65 °C, 24 h; (g) 2 M NaOH, MeOH, 40 °C, 48 h; (h) 4 M HCl in dioxane, 22 °C, 1 h; (i) 2-trichloroacetyl-4-chloro-5-methyl-1*H*-pyrrole, Na_2_CO_3_, DMF, 65 °C, 18 h; (j) 4-fluoro-5-methyl-1*H*-pyrrole-2-carbonyl chloride, toluene, 130 °C, 18 h; and (k) 4-cyano-5-methyl-1*H*-pyrrole-2-carbonyl chloride, toluene,130 °C, 18 h.

To obtain compounds **65–74**, the required building blocks **48s65**–**48s73** were prepared from 4′-(4-bromomethyl)acetophenone via nucleophilic substitution and subsequent reduction of ketones **47s65**–**47s73**, or they were commercially available. 2-Aminobenzothiazoles **51s65**–**51s73** were prepared following the usual reaction sequence and were coupled to substituted pyrroles to give esters **52s65**, **52s70**–**52s73**, and **53s66**–**53s68** that afforded *para*-substituted derivatives **65**–**73** after alkaline hydrolysis (Scheme 11).

## 3 Conclusions

In summary, modification of the initial hit **1** afforded compound **27**, which was endowed with excellent activity against *A. baumannii*, *P. aeruginosa*, and *E. coli*, but unfortunately possessed only partially ameliorated physicochemical and ADME properties. Attempts to improve these properties to target values needed for in vivo studies (thermodynamic solubility higher than 0.3 mg/mL, plasma free fraction 0.5–2%) while retaining the antibacterial activity against *A. baumannii* or *P. aeruginosa* (MIC < 4 μg/mL) resulted in several derivatives with improved solubility and free fraction but unfortunately with partial or complete loss of promising antibacterial activity against Gram-negative bacteria observed in **27**. The optimization of **27** to a lead compound turned out to be very difficult and would require a comprehensive multiparameter optimization, which could not be performed within this project. However, with over 70 compounds synthesized and tested, promising directions for the optimization of solubility and plasma protein binding were identified.

## 4 Experimental Section

### 4.1 Chemistry: General Procedures and Instrumentation

Chemicals were obtained from Acros Organics (Geel, Belgium), Enamine Ltd. (Kyiv, Ukraine), Sigma-Aldrich (St. Louis, MO), TCI (Tokyo, Japan), Fluorochem Ltd. (Derbyshire, U.K.), and Apollo Scientific (Stockport, U.K.) and were used without further purification. Analytical TLC was performed on silica gel Merck 60 F254 plates (0.25 mm), using visualization with UV light and spray reagents. Column chromatography was carried out on silica gel 60 (particle size 240–400 mesh). Analytical reversed-phase HPLC–UV–MS analyses were performed on a 1260 Infinity II LC system (Agilent Technologies Inc., SantaClara, CA). A Waters XBridge C18 column was used (4.6 mm × 150 mm, 3.5 μm), with a sample injection volume of 10 μL and a flow rate of 1.5 mL/min, coupled to an ADVION expression CMS^L^ quadrupole mass spectrometer (Advion Inc., Ithaca). The eluent consisted of acetonitrile (solvent A) and 0.1% formic acid in 1% acetonitrile in ultrapure water (solvent B). The gradient (defined for solvent A) was 0–1.0 min, 25%; 1.0–6.0 min, 25–98%; 6.0–6.5 min, 98%; 6.5–7.5 min, 98–25%; 7.5–10.5 min, 25%. ^1^H NMR (400 MHz, internal Me_4_Si), ^13^C NMR (101 MHz, internal CDCl_3_ or DMSO-d_6_), and ^19^F NMR (376 MHz, external CCl_3_F) spectra were recorded on a Bruker AVANCE III 400 spectrometer (Bruker Corporation, Billerica, MA) in DMSO-*d*_6_ or CDCl_3_ solution. High-resolution mass spectra were obtained using an Exactive Plus Orbitrap mass spectrometer (Thermo Fisher Scientific, Waltham, MA). All final compounds were >95% pure by HPLC analysis unless otherwise specified.

The tested compounds passed screening against the PAINS filter, as available in CANVAS (Schrödinger Release 2020-3: Canvas, Schrödinger, LLC, New York, NY, 2020).

### 4.2 Synthesis of Compounds

#### 4.2.1 4-(Benzyloxy)-2-(3,4-dichloro-5-methyl-1*H*-pyrrole-2-carb-oxamido)benzo[*d*]thiazole-6-carboxylic Acid (**1**)

The compound was prepared from methyl 3-hydroxy-4-nitrobenzoate (**2s**) according to our published procedure.^15^

#### 4.2.2 Methyl 4-Nitro-3-(pyridin-4-ylmethoxy)benzoate (**3s2**): Typical Procedure A for Alkylation of 3-Hydroxy-4-nitrobenzoates with Benzylic Halides

To a solution of methyl 3-hydroxy-4-nitrobenzoate (**2s**) (2.00 g, 10.1 mmol) in acetonitrile (90 mL), K_2_CO_3_ (5.60 g, 40.5 mmol), 4-(chloromethyl)pyridine hydrochloride (1.83 g, 11.2 mmol), and KI (0.37 g, 0.2 equiv) were added and the reaction mixture was stirred at 60 °C overnight. The volatiles were removed under reduced pressure, and the residue was partitioned between ethyl acetate and water. The organic layer was washed with brine, dried over Na_2_SO_4_, filtered, and concentrated. The crude product was triturated with methanol to get the title compound as a beige solid (1.08 g, 37% yield). ^1^H NMR (400 MHz, DMSO-*d*_6_): δ 8.62 (d, *J* = 3.7 Hz, 2H), 8.08 (d, *J* = 8.4 Hz, 1H), 7.87 (d, *J* = 1.4 Hz, 1H), 7.72 (dd, *J* = 8.4, 1.5 Hz, 1H), 7.45 (d, *J* = 5.5 Hz, 1H), 5.50 (s, 2H), 3.91 (s, 3H); MS (ESI): *m*/*z* calcd for C_14_H_12_N_2_O_5_: 288.07. Found: 289.0 [M + H]^+^.

#### 4.2.3 Methyl 4-Amino-3-(pyridin-4-ylmethoxy)benzoate (**4s2**): Typical Procedure B for Reduction of 4-Nitro-3-alkoxybenzoates with Iron

Methyl 4-nitro-3-(pyridin-4-ylmethoxy)benzoate (**3s2**) (500 mg, 1.74 mmol) was suspended in acetic acid (40 mL), and iron powder (486 mg, 8.68 mmol) was then added and the reaction mixture was stirred at 60 °C for 1 h. Water was added to dissolve the white precipitate, and residual iron was removed by filtration through celite. The residue was neutralized with saturated aqueous NaHCO_3_ and extracted with ethyl acetate. The crude product was purified by flash column chromatography, eluent ethyl acetate/hexane (3:1); colorless oil (380 mg, 85% yield). ^1^H NMR (400 MHz, DMSO-*d*_6_): δ 8.62–8.53 (m, 2H), 7.55 (d, *J* = 6.0 Hz, 2H), 7.41 (dd, *J* = 8.2, 1.8 Hz, 1H), 7.36 (d, *J* = 1.7 Hz, 1H), 6.69 (d, *J* = 8.2 Hz, 1H), 5.81 (s, 2H), 5.22 (s, 2H), 3.74 (s, 3H); MS (ESI): *m*/*z* calcd for C_14_H_14_N_2_O_3_: 258.10. Found: 258.8 [M + H]^+^.

#### 4.2.4 Methyl 2-Amino-4-(pyridin-4-ylmethoxy)benzo[*d*]thiazole-6-carboxylate (**5s2**): Typical Procedure C for Benzothiazole Formation

To a suspension of the above aniline **4s2** (395 mg, 1.52 mmol) in acetic acid (8 mL), KSCN (594 mg, 6.11 mmol) was added and stirred at 22 °C until a clear solution was obtained. The reaction mixture was cooled on an ice bath while a solution of bromine (160 μL, 3.04 mmol) in acetic acid (0.5 mL) was added dropwise. The resulting mixture was stirred at 22 °C overnight; then, it was neutralized with saturated aqueous NaHCO_3_, and the precipitate was collected, washed with water, and air-dried. The solid was percolated with methanol and then triturated with cold methanol to get the title compound as a white solid (324 mg, 67% yield). ^1^H NMR (400 MHz, DMSO-*d*_6_): δ 8.60 (d, *J* = 6.0 Hz, 2H), 8.00 (d, *J* = 1.5 Hz, 1H), 7.95 (s, 2H), 7.49 (d, *J* = 6.0 Hz, 2H), 7.43 (d, *J* = 1.5 Hz, 1H), 5.33 (s, 2H), 3.82 (s, 3H); MS (ESI): *m*/*z* calcd for C_15_H_13_N_3_O_3_S: 315.07. Found: 316.0 [M + H]^+^.

#### 4.2.5 Methyl 2-(3,4-Dichloro-5-methyl-1*H*-pyrrole-2-carboxamido)-4-(pyridin-4-ylmethoxy)benzo[*d*]thiazole-6-carboxylate (**6s2**): Typical Procedure D for Acylation with Pyrrole-2-carbonyl Chlorides

A suspension of 3,4-dichloro-5-methyl-1*H*-pyrrole-2-carbonyl chloride (prepared from the corresponding carboxylic acid (86 mg, 0.44 mmol) by refluxing in SOCl_2_ (2 mL) for 1 h and then concentrating under reduced pressure) and the above benzothiazole **5s2** (139 mg, 0.44 mmol) in toluene (9 mL) was refluxed overnight. Upon cooling, the precipitate was collected and washed with toluene to get the title compound as a gray solid (87 mg, 40% yield). ^1^H NMR (400 MHz, DMSO-*d*_6_): δ 12.31 (s, 1H), 12.20 (s, 1H), 8.73 (d, *J* = 5.3 Hz, 2H), 8.34 (d, *J* = 1 Hz, 1H), 7.75–7.67 (m, 2H), 7.60 (d, *J* = 1 Hz, 1H), 5.51 (s, 2H), 3.88 (s, 3H), 2.27 (s, 3H); MS (ESI): *m*/*z* calcd for C_21_H_16_Cl_2_N_4_O_4_S: 490.03. Found: 488.9 [M – H]^−^.

#### 4.2.6 2-(3,4-Dichloro-5-methyl-1*H*-pyrrole-2-carboxamido)-4-(pyridin-4-ylmethoxy)benzo[*d*]thiazole-6-carboxylic Acid (**2**): Typical procedure E for Methyl Ester Hydrolysis

A solution of the above methyl ester **6s2** (60 mg, 0.122 mmol) in MeOH (3 mL) and 2 M NaOH (0.3 mL) was stirred at 40 °C for 24 h. NaOH (2 M, 0.3 mL) was added and stirred at 40 °C for an additional 24 h and then concentrated. The solid residue was dispersed in water (3 mL), filtered through cotton, and the filtrate was brought to pH = 1 by adding 4 M HCl. The precipitate was collected on a frit, washed with water (0.6 mL), and air-dried. The crude product was triturated with MeOH (2 × 12 mL) to get the title compound as a brown solid (25 mg, 43% yield). ^1^H NMR (400 MHz, DMSO-*d*_6_): δ 13.02 (s, 1H), 12.38 (s, 1H), 12.18 (s, 1H), 8.83–8.76 (m, 2H), 8.30 (d, *J* = 1 Hz, 1H), 7.83 (d, *J* = 5.4 Hz, 2H), 7.60 (d, *J* = 1 Hz, 1H), 5.57 (s, 2H), 2.28 (s, 3H); HRMS (ESI) *m*/*z*: [M + H]^+^ calcd for C_20_H_15_Cl_2_N_4_O_4_S 477.0191; found 477.0187; HPLC purity (254 nm): 92%.

#### 4.2.7 Methyl 2-Amino-4-(pyridin-3-ylmethoxy)benzo[*d*]thiazole-6-carboxylate (**5s3**): Typical Procedure F for Alkylation of 2-Aminobenzo[*d*]thiazol-4-ols

To a solution of methyl 2-amino-4-hydroxybenzo[*d*]thiazole-6-carboxylate (**1s**) (682 mg, 3.04 mmol) and 3-(chloromethyl)pyridine hydrochloride (598 mg, 3.65 mmol) in DMF (30 mL), K_2_CO_3_ (841 mg, 6.09 mmol) and KI (101 mg, 0.61 mmol) were added. The reaction mixture was stirred at 60 °C overnight; then, the volatiles were removed under reduced pressure. The residue was percolated with ethyl acetate, and the crude product was purified by flash column chromatography, using as eluent 1:2 ethyl acetate/hexane and then 1:1 ethyl acetate/hexane to get the title compound as a yellow powder (79 mg, 8% yield). ^1^H NMR (400 MHz, DMSO-*d*_6_): δ 8.71 (d, *J* = 1.7 Hz, 1H), 8.56 (dd, *J* = 4.8, 1.7 Hz, 1H), 7.99 (d, *J* = 1.5 Hz, 1H), 7.96–7.88 (m, 3H), 7.48 (d, *J* = 1.5 Hz, 1H), 7.47–7.42 (m, 1H), 5.29 (s, 2H), 3.83 (s, 3H); MS (ESI): *m*/*z* calcd for C_15_H_13_N_3_O_3_S: 315.07. Found: 316.1 [M + H]^+^.

#### 4.2.8 Methyl 2-(3,4-dichloro-5-methyl-1*H*-pyrrole-2-carboxamido)-4-(pyridin-3-ylmethoxy)benzo[*d*]thiazole-6-carboxylate (**6s3**)

Prepared according to the typical procedure D from **5s3** (59 mg, 0.252 mmol); gray solid (57 mg 46% yield). ^1^H NMR (400 MHz, DMSO): δ 12.33 (s, 1H), 12.20 (s, 1H), 8.95–8.92 (m, 1H), 8.77 (dd, *J* = 5.2, 1.6 Hz, 1H), 8.37–8.31 (m, 2H), 7.83–7.78 (m, 1H), 7.66 (d, *J* = 1.5 Hz, 1H), 5.48 (s, 2H), 3.90 (s, 3H), 2.26 (s, 3H); MS (ESI): *m*/*z* calcd for C_21_H_16_Cl_2_N_4_O_4_S: 490.03. 489.2 [M – H]^−^.

#### 4.2.9 2-(3,4-Dichloro-5-methyl-1*H*-pyrrole-2-carboxamido)-4-(pyridin-3-ylmethoxy)benzo[*d*]thiazole-6-carboxylic Acid (**3**)

Prepared according to the typical procedure E from **6s3** (48 mg, 0.097 mmol); light brown powder (18 mg, 39% yield). ^1^H NMR (400 MHz, DMSO-*d*_6_): δ 13.03 (s, 1H), 12.30 (s, 1H), 12.17 (s, 1H), 8.94–8.88 (m, 1H), 8.75 (dd, *J* = 5.2, 1.6 Hz, 1H), 8.32–8.25 (m, 2H), 7.76 (dd, *J* = 7.9, 5.2 Hz, 1H), 7.66 (d, *J* = 1.5 Hz, 1H), 5.46 (s, 2H), 2.26 (s, 3H); HRMS (ESI) *m*/*z*: [M + H]^+^ calcd for C_20_H_15_Cl_2_N_4_O_4_S 477.0191; found 477.0187; HPLC purity (254 nm): 93.0%.

#### 4.2.10 Methyl 2-Amino-4-(pyrimidin-2-ylmethoxy)benzo[*d*]thiazole-6-carboxylate (**5s4**)

Prepared according to the typical procedure F from **1s** (99 mg, 0.441 mmol); yellow crystals (53 mg, 38% yield). ^1^H NMR (400 MHz, DMSO-*d*_6_): δ 8.85 (d, *J* = 4.9 Hz, 2H), 7.95 (d, *J* = 1.5 Hz, 1H), 7.89 (s, 2H), 7.48 (t, *J* = 4.9 Hz, 1H), 7.36 (d, *J* = 1.5 Hz, 1H), 5.40 (s, 2H), 3.80 (s, 3H); HRMS (ESI) *m*/*z*: [M + H]^+^ calcd for C_14_H_13_N_4_O_3_S: 317.0708; found 317.0700.

#### 4.2.11 Methyl 2-(3,4-Dichloro-5-methyl-1*H*-pyrrole-2-carboxamido)-4-(pyrimidin-2-ylmethoxy)benzo[*d*]thiazole-6-carboxylate (**6s4**)

Prepared according to the typical procedure D from **5s4** (52 mg, 0.164); gray solid (59 mg, 73% yield). ^1^H NMR (400 MHz, DMSO-*d*_6_): δ 12.27 (s, 1H), 12.20 (s, 1H), 8.87 (d, *J* = 4.9 Hz, 2H), 8.29 (s, 1H), 7.59–7.47 (m, 2H), 5.51 (s, 2H), 3.86 (s, 3H), 2.27 (s, 3H).

#### 4.2.12 2-(3,4-Dichloro-5-methyl-1*H*-pyrrole-2-carboxamido)-4-(pyrimidin-2-ylmethoxy)benzo[*d*]thiazole-6-carboxylic Acid (**4**)

Prepared according to the typical procedure E from **6s4** (55 mg, 0.112 mmol); brown solid (31 mg 58% yield). ^1^H NMR (400 MHz, DMSO-*d*_6_): δ 12.95 (s, 1H), 12.27 (s, 1H), 12.15 (s, 1H), 8.87 (d, *J* = 4.9 Hz, 2H), 8.24 (d, *J* = 1.4 Hz, 1H), 7.54–7.47 (m, 2H), 5.49 (s, 2H), 2.26 (s, 3H); HRMS (ESI) *m*/*z*: [M + H]^+^ calcd for C_19_H_14_Cl_2_N_5_O_4_S 478.0144; found 478.0136; HPLC purity (254 nm): 98%.

#### 4.2.13 4-(((6-Carboxy-2-(3,4-dichloro-5-methyl-1*H*-pyrrole-2-carboxamido)benzo[*d*]thiazol-4-yl)oxy)methyl)-1-methylpyri-din-1-ium Iodide (**5**): Typical Procedure G for Quaternization Using Iodomethane

To a solution of **2** (15 mg, 0.031 mmol) in a mixture of THF (4.5 mL) and DMF (3 mL), methyl iodide (19 μL, 0.31 mmol) was added and the reaction mixture was stirred in a pressure tube at 60 °C overnight. The volatiles were removed under reduced pressure, and the residue was triturated with a mixture of DMF (450 μL) and toluene (3 mL) to get the title compound as a gray solid (13.2 mg 68% yield). ^1^H NMR (400 MHz, DMSO-*d*_6_): δ 13.04 (s, 1H), 12.33 (s, 1H), 12.07 (s, 1H), 8.99 (d, *J* = 6 Hz, 2H), 8.33 (d, *J* = 1.4 Hz, 1H), 8.20 (d, *J* = 6 Hz, 2H), 7.95 (s, 1H), 7.60 (d, *J* = 1.4 Hz, 1H), 5.76 (s, 2H), 4.35 (s, 3H), 2.28 (s, 3H); HRMS (ESI) *m*/*z*: [M – I]^+^ calcd for C_21_H_17_Cl_2_N_4_O_4_S 491.0348; found 491.0334; HPLC purity (254 nm): 95%.

#### 4.2.14 3-(((6-Carboxy-2-(3,4-dichloro-5-methyl-1*H*-pyrrole-2-carboxamido)benzo[*d*]thiazol-4-yl)oxy)methyl)-1-methylpyri-din-1-ium Iodide (**6**)

Prepared according to the typical procedure G from **3** (10 mg, 0.021 mmol); beige solid (7.3 mg, 56% yield). ^1^H NMR (400 MHz, DMSO-*d*_6_): δ 13.08 (s, 1H), 12.31 (s, 1H), 12.02 (s, 1H), 9.22 (s, 1H), 9.02 (d, *J* = 6.1 Hz, 1H), 8.74 (d, *J* = 8.1 Hz, 1H), 8.34 (s, 1H), 8.23 (dd, *J* = 8.1, 6.1 Hz, 1H), 7.68 (d, *J* = 1.4 Hz, 1H), 5.58 (s, 2H), 4.41 (s, 3H), 2.27 (s, 3H); HRMS (ESI) *m*/*z*: [M – I]^+^ calcd for C_21_H_17_Cl_2_N_4_O_4_S 491.0348; found 491.0335; HPLC purity (254 nm): 97%.

#### 4.2.15 Methyl 2-Amino-4-((4-mesylbenzyl)oxy)benzo[*d*]thiazole-6-carboxylate (**5s8**)

Prepared according to the typical procedure F from **1s** (514 mg, 2.29 mmol); bright yellow powder (135 mg, 15% yield). ^1^H NMR (400 MHz, DMSO-*d*_6_): δ 7.99 (d, *J* = 1.5 Hz, 1H), 7.97 (d, *J* = 8.4 Hz, 2H), 7.94 (s, 2H), 7.76 (d, *J* = 8.4 Hz, 2H), 7.46 (d, *J* = 1.5 Hz, 1H), 5.39 (s, 2H), 3.83 (s, 3H). ^13^C NMR (101 MHz, DMSO-*d*_6_): δ 168.87, 166.04, 147.67, 146.76, 143.08, 140.17, 132.02, 128.15, 127.13, 122.20, 116.24, 110.18, 69.15, 51.99, 43.54.

#### 4.2.16 Methyl 2-(3,4-Dichloro-5-methyl-1*H*-pyrrole-2-carboxamido)-4-((4-mesylbenzyl)oxy)benzo[*d*]thiazole-6-carboxylate (**6s8**)

Prepared according to the typical procedure D from **5s8** (120 mg, 0.303 mmol); gray powder (76 mg, 44% yield). ^1^H NMR (400 MHz, DMSO-*d*_6_): δ 12.28 (s, 1H), 12.18 (s, 1H), 8.33 (s, 1H), 8.00 (d, *J* = 8 Hz, 2H), 7.81 (d, *J* = 8 Hz, 2H), 7.63 (s, 1H), 5.48 (s, 2H), 3.89 (s, 3H), 3.25 (s, 3H), 2.27 (s, 3H).

#### 4.2.17 2-(3,4-Dichloro-5-methyl-1*H*-pyrrole-2-carboxamido)-4-{(4-mesylphenyl)methoxy}benzo[*d*]thiazole-6-carboxylic Acid (**8**)

Prepared according to the typical procedure E from **6s8** (70 mg, 0.123 mmol); light brown powder (47 mg, 69% yield). ^1^H NMR (400 MHz, DMSO-*d*_6_): δ 13.03 (s, 1H), 12.27 (s, 1H), 12.15 (s, 1H), 8.28 (s, 1H), 8.00 (d, *J* = 8.2 Hz, 2H), 7.80 (d, *J* = 8.2 Hz, 2H), 7.62 (s, 1H), 5.47 (s, 2H), 3.25 (s, 3H), 2.27 (s, 3H); HRMS (ESI) *m*/*z*: [M – H]^−^ calcd for C_22_H_16_Cl_2_N_3_O_6_S_2_ 551.9858; found 551.9863; HPLC purity (254 nm): 97%.

#### 4.2.18 2-(3,4-Dichloro-5-methyl-1*H*-pyrrole-2-carboxamido)-4-((3-fluorobenzyl)oxy)benzo[*d*]thiazole-6-carboxylic Acid (**9**)

The compound was prepared according to our published procedure.^16^

#### 4.2.19 Methyl 3-(2-Methoxyethoxy)-4-nitrobenzoate (**3s10**)

The compound was prepared according to a typical procedure H (see below) from **2s** (0.70 g, 3.55 mmol) and was used in the next step without further purification.

#### 4.2.20 Methyl 4-Amino-3-(2-methoxyethoxy)benzoate (**4s10**)

Prepared according to the typical procedure I (see below) from crude **3s10** (1.00 g, 3.92 mmol); yellowish oil (640 mg, 80% yield for two steps). ^1^H NMR (400 MHz, CDCl_3_): δ 7.56 (dd, *J* = 8.2, 1.8 Hz, 1H), 7.47 (d, *J* = 1.8 Hz, 1H), 6.67 (d, *J* = 8.2 Hz, 1H), 4.31 (s, 2H), 4.24–4.16 (m, 2H), 3.85 (s, 3H), 3.80–3.73 (m, 2H), 3.44 (s, 3H).

#### 4.2.21 Methyl 2-Amino-4-(2-methoxyethoxy)benzo[*d*]thiazole-6-carboxylate (**5s10**)

Prepared according to the typical procedure C from **4s10** (299 mg, 1.33 mmol); yellow solid (150 mg, 40% yield); ^1^H NMR (400 MHz, DMSO-*d*_6_): δ 7.96 (d, *J* = 1.6 Hz, 1H), 7.91 (s, 2H), 7.35 (d, *J* = 1.6 Hz, 1H), 4.27–4.18 (m, 2H), 3.83 (s, 3H), 3.72–3.66 (m, 2H), 3.32 (s, 3H); ^13^C NMR (101 MHz, DMSO-*d*_6_): δ 168.60, 166.12, 148.17, 146.53, 131.82, 122.22, 115.80, 109.45, 70.44, 67.69, 58.16, 51.96; HRMS (ESI) *m*/*z*: [M + H]^+^ calcd for C_12_H_15_N_2_O_4_S 283.0753; found 283.0744.

#### 4.2.22 Methyl 2-(3,4-Dichloro-5-methyl-1*H*-pyrrole-2-carboxamido)-4-(2-methoxyethoxy)benzo[*d*]thiazole-6-carboxylate (**6s10**)

Prepared according to the typical procedure D from **5s10** (129 mg, 0.458 mmol); gray solid (143 mg, 68% yield). ^1^H NMR (400 MHz, DMSO-*d*_6_): δ 12.28 (s, 1H), 12.25 (s, 1H), 8.28 (d, *J* = 1.4 Hz, 1H), 7.49 (d, *J* = 1.4 Hz, 1H), 4.41–4.27 (m, 2H), 3.88 (s, 3H), 3.79–3.71 (m, 2H), 2.28 (s, 3H).

#### 4.2.23 2-(3,4-Dichloro-5-methyl-1*H*-pyrrole-2-carboxamido)-4-(2-methoxyethoxy)benzo[*d*]thiazole-6-carboxylic Acid (**10**)

Prepared according to the typical procedure E from **6s10** (90 mg, 0.196 mmol); off-white solid (54 mg, 62% yield). ^1^H NMR (400 MHz, DMSO-*d*_6_): δ 12.98 (s, 1H), 12.25 (s, 2H), 8.23 (d, *J* = 1.4 Hz, 1H), 7.49 (d, *J* = 1.5 Hz, 1H), 4.47–4.18 (m, 2H), 3.95–3.67 (m, 2H), 3.34 (s, 3H), 2.27 (s, 3H); ^13^C NMR (101 MHz, DMSO-*d*_6_): δ 167.08, 159.50, 156.59, 150.30, 141.65, 132.80, 129.98, 126.76, 116.87, 116.15, 115.66, 109.96, 108.45, 70.33, 67.48, 58.12, 11.08; HRMS (ESI) *m*/*z*: [M + H]^+^ calcd for C_17_H_16_Cl_2_N_3_O_5_S 444.0188; found 444.0184; HPLC purity (254 nm): 99%.

#### 4.2.24 Methyl 3-(2-(Dimethylamino)propoxy)-4-nitrobenzoate (**3s11**): Typical Procedure H for Alkylation of 3-Hydroxy-4-nitrobenzoates Using the Mitsunobu Reaction

To a stirred solution of methyl 3-hydroxy-4-nitrobenzoate (**2s**) (492 mg, 2.49 mmol) and triphenylphosphine (1.31 g, 4.98 mmol) in anhydrous tetrahydrofuran (15 mL) was added 2-(dimethylamino)propan-1-ol (283 mg, 2.74 mmol), and the mixture was stirred at 22 °C for 10 min. DIAD (1.01 g, 4.98 mmol) was added dropwise, and the mixture was stirred at 22 °C for 15 h. The volatiles were removed under reduced pressure, and a mixture of two regioisomers was separated by column chromatography on silica, using hexane/ethyl acetate (2:1) as an eluent to give a colorless oil (176 mg, 25% yield). ^1^H NMR (400 MHz, CDCl_3_): δ 7.83 (d, *J* = 8.3 Hz, 1H), 7.75 (s, 1H), 7.69 (d, *J* = 8.3 Hz, 1H), 4.24 (dd, *J* = 9.1, 5.3 Hz, 1H), 4.02 (dd, *J* = 9.1, 6.2 Hz, 1H), 3.06 (h, *J* = 6.2 Hz, 1H), 2.36 (s, 6H), 1.18 (d, *J* = 6.2 Hz, 3H); ^13^C NMR (101 MHz, CDCl_3_): δ 165.20, 151.84, 142.59, 134.80, 125.27, 121.41, 115.56, 77.32, 77.00, 76.68, 71.86, 58.01, 52.82, 41.54, 12.38; MS (ESI): *m*/*z* calcd for C_13_H_18_N_2_O_5_: 282.12. Found: 283.13 [M + H]^+^.

#### 4.2.25 Methyl 4-Amino-3-(2-(dimethylamino)propoxy)benzoate (**4s11**): Typical Procedure I for Reduction of 4-Nitrobenzoates with Pd/C-Catalyzed Hydrogenation

Methyl 3-(2-(dimethylamino)propoxy)-4-nitrobenzoate (**3s11**) (1.00 g, 3.54 mmol) was dissolved in methanol/tetrahydrofuran (7:3, 100 mL) and flushed with argon. Pd/C (10%, 16 mg) was added, and the reaction mixture was hydrogenated at 1 atm and 22 °C for 5 h. The catalyst was filtered off, and the solvent was removed in vacuo to get the title compound as a colorless oil (143 mg, quant. yield). ^1^H NMR (400 MHz, CDCl_3_): δ 7.55 (d, *J* = 8.2 Hz, 1H), 7.45 (s, 1H), 6.65 (d, *J* = 8.2 Hz, 1H), 4.50 (s, 1H), 4.07 (dd, *J* = 9.8, 6.4 Hz, 1H), 3.94 (dd, *J* = 9.8, 5.3 Hz, 1H), 3.86 (s, 3H), 3.02 (h, *J* = 6.5 Hz, 1H), 2.36 (s, 6H), 1.13 (d, *J* = 6.5 Hz, 3H) ppm; MS (ESI): *m*/*z* calcd for C_13_H_20_N_2_O_3_: 252.15. Found: 253.16 [M + H]^+^.

#### 4.2.26 Methyl 2-Amino-4-(2-(dimethylamino)propoxy)benzo[*d*]-thiazole-6-carboxylate (**5s11**)

Prepared according to the typical procedure C from aniline **4s11** (157 mg, 0.621 mmol); orange solid (73 mg, 38% yield); ^1^H NMR (400 MHz, DMSO-*d*_6_): δ = 8.02 (s, 1H), 7.94 (s, 2H), 7.45 (s, 1H), 4.30 (m, 1H), 3.83 (s, 3H), 1.23 (s, 3H) (some peaks overlapped by DMSO and water signals); MS (ESI): *m*/*z* calcd for C_14_H_19_N_3_O_3_S: 309.11. Found: 310.1 [M + H]^+^.

#### 4.2.27 Methyl 2-(3,4-Dichloro-5-methyl-1*H*-pyrrole-2-carboxamido)-4-(2-(dimethylamino)propoxy)benzo[*d*]thiazole-6-carboxylate (**6s11**): Typical Procedure J for Acylation with 2-Trichloroacetylpyrroles

A mixture of 2-aminobenzothiazole **5s11** (74 mg, 0.240 mmol), 2-trichloroacetyl-3,4-dichloro-5-methyl-1*H*-pyrrole (71 mg, 0.240 mmol), and Na_2_CO_3_ (25 mg, 0.240 mmol) was suspended in dry DMF (5 mL) under Ar and stirred at 60 °C overnight; then, it was concentrated under reduced pressure and triturated sequentially with 10% citric acid, water, EtOAc, and MeOH to get the title compound as a black solid (64 mg, 55% yield). ^1^H NMR (400 MHz, DMSO-d_6_): δ 12.36 (s, 1H), 8.33 (s, 1H), 7.62 (s, 1H), 4.50 (s, 3H), 3.89 (s, 3H), 2.27 (s, 3H), 1.39 (s, 3H) (some peaks overlapped by DMSO and water signals); MS (ESI): *m*/*z* calcd for C_20_H_22_Cl_2_N_4_O_4_S: 484.07. Found: 485.08 [M + H]^+^.

#### 4.2.28 2-(3,4-Dichloro-5-methyl-1*H*-pyrrole-2-carboxamido)-4-(2-(dimethylamino)propoxy)benzo[*d*]thiazole-6-carboxylic Acid (**11**)

Prepared according to the typical procedure E from **6s11** (65 mg 0.133 mmol); brown solid (25 mg, 40% yield). ^1^H NMR (400 MHz, DMSO-*d*_6_): δ characteristic peaks: 8.12 (s, 1H), 7.49 (s, 1H), 4.25 (m, 1H), 4.13 (m, 1H), 2.24 (s, 3H), 1.14 (d, *J* = 6.4 Hz, 3H); HRMS (ESI) *m*/*z*: [M + H]^+^ calcd for C_19_H_21_Cl_2_N_4_O_4_S 471.0661; found 471.0654; HPLC purity (254 nm): 96%.

#### 4.2.29 Methyl 4-Nitro-3-(2,2,2-trifluoroethoxy)benzoate (**3s12**)

Prepared according to the typical procedure A from **2s** (1.25 g, 6.34 mmol), and used in the next step without any further purification.

#### 4.2.30 Methyl 4-Amino-3-(2,2,2-trifluoroethoxy)benzoate (**4s12**)

Prepared according to the typical procedure B from **3s12** (500 mg, 1.79 mmol); white crystals (181 mg 41% yield). ^1^H NMR (400 MHz, DMSO-*d*_6_): δ 7.46 (dd, *J* = 8.2, 1.8 Hz, 1H), 7.43 (d, *J* = 1.8 Hz, 1H), 6.71 (d, *J* = 8.2 Hz, 1H), 5.70 (s, 2H), 4.76 (q, *J* = 8.9 Hz, 2H), 3.76 (s, 3H); MS (ESI): *m*/*z* calcd for C_10_H_10_F_3_NO_3_: 249.06. Found: 249.9 [M + H]^+^.

#### 4.2.31 Methyl 2-Amino-4-(2,2,2-trifluoroethoxy)benzo[*d*]thiazole-6-carboxylate (**5s12**)

Prepared according to the typical procedure C from **4s12** (169 mg, 0.680 mmol); yellow solid (75 mg, 36% yield). ^1^H NMR (400 MHz, DMSO-*d*_6_); δ 8.09–8.02 (m, 3H), 7.45 (d, *J* = 1.6 Hz, 1H), 4.97–4.88 (m, 2H), 3.84 (s, 3H); MS (ESI): *m*/*z* calcd for C_11_H_9_F_3_N_2_O_3_S: 306.03. Found: 306.8 [M + H]^+^.

#### 4.2.32 Methyl 2-(3,4-Dichloro-5-methyl-1*H*-pyrrole-2-carboxamido)-4-(2,2,2-trifluoroethoxy)benzo[*d*]thiazole-6-carboxylate (**6s12**)

Prepared according to the typical procedure D from **5s12** (75 mg, 0.245 mmol); gray solid (102 mg 86% yield). ^1^H NMR (400 MHz, DMSO-*d*_6_): δ 12.32 (s, 1H), 12.29 (s, 1H), 8.40 (d, *J* = 1.4 Hz, 1H), 7.62 (d, *J* = 1.5 Hz, 1H), 5.06 (q, *J* = 8.8 Hz, 2H), 3.90 (s, 3H), 2.28 (s, 3H); MS (ESI): *m*/*z* calcd for C_17_H_12_Cl_2_F_3_N_3_O_4_S: 480.99. Found: 480.1 [M – H]^−^.

#### 4.2.33 2-(3,4-Dichloro-5-methyl-1*H*-pyrrole-2-carboxamido)-4-(2,2,2-trifluoroethoxy)benzo[*d*]thiazole-6-carboxylic Acid (**12**)

Prepared according to the typical procedure E from **6s12** (75 mg, 0.156 mmol); brown solid (60 mg, 82% yield). ^1^H NMR (400 MHz, DMSO-*d*_6_): δ 13.09 (s, 1H), 12.29 (s, 2H), 8.35 (d, *J* = 1.4 Hz, 1H), 7.61 (d, *J* = 1.4 Hz, 1H), 5.04 (q, *J* = 8.8 Hz, 2H), 2.28 (s, 3H); ^13^C NMR (101 MHz, DMSO-*d*_6_); δ 166.87, 160.28, 156.58, 148.44, 141.62, 133.28, 130.06, 126.69, 124.05 (q, *J* = 277 Hz), 117.72, 116.70, 116.05, 110.11, 109.79, 65.19 (q, *J* = 34.2 Hz), 11.12; HRMS (ESI) *m*/*z*: [M – H]^−^ calcd for C_16_H_9_Cl_2_F_3_N_3_O_4_S: 465.9643; found 465.9646; HPLC purity (254 nm): 95%.

#### 4.2.34 Methyl 3-(Benzyloxy)-4-nitrobenzoate (**3s1**)

Prepared according to the typical procedure A from **2s (**500 mg, 2.54 mmol); yellow solid (620 mg, 85% yield). mp 90–93 °C. ^1^H NMR (400 MHz, CDCl_3_): δ 7.85 (d, *J* = 8.3 Hz, 1H), 7.83 (d, *J* = 1.6 Hz, 1H), 7.70 (dd, *J* = 8.3, 1.6 Hz, 1H), 7.51–7.43 (m, 2H), 7.45–7.36 (m, 2H), 7.39–7.30 (m, 1H), 5.29 (s, 2H), 3.96 (s, 3H); MS (ESI): *m*/*z* calcd for C_15_H_13_NO_5_: 287.08. Found: 310.1 [M + Na]^+^.

#### 4.2.35 3-(Benzyloxy)-4-nitrobenzohydrazide (**7s14**)

To a solution of ester **3s1** (4.60 g, 16.0 mmol) in MeOH (100 mL) and THF (100 mL), 80% of hydrazine monohydrate solution in water (7.78 mL, 160 mmol) was added. The reaction mixture was stirred at 65 °C overnight, and the solvent was removed under reduced pressure. The residue was suspended in ethanol and macerated at 5 °C for 1 h. The precipitate was collected and triturated with water to give the first crop (1.44 g) of the title compound. Ethanol from the mother liquor of the preceding filtration was evaporated, and the residue was purified by flash column chromatography (dichloromethane/methanol = 10:1) to give the second crop (2.10 g) of the title compound as beige solid (3.54 g, 77% combined yield); ^1^H NMR (400 MHz, DMSO-*d*_6_): δ 10.06 (s, 1H), 7.98 (d, *J* = 8.4 Hz, 1H), 7.83 (d, *J* = 1.6 Hz, 1H), 7.55 (dd, *J* = 8.4, 1.6 Hz, 1H), 7.50–7.39 (m, 4H), 7.38–7.33 (m, 1H), 5.37 (s, 2H), 4.63 (s, 2H); MS (ESI): *m*/*z* calcd for C_14_H_13_N_3_O_4_: 287.09. Found: 285.9 [M – H]^−^.

#### 4.2.36 5-(3-(Benzyloxy)-4-nitrophenyl)-1,3,4-oxadiazol-2(3*H*)-one (**8s14**)

To a solution of hydrazide **7s14** (3.50 g, 12.2 mmol) in 1,4-dioxane (175 mL), CDI (2.96 g, 18.3 mmol) was added and the reaction mixture was stirred at 100 °C overnight. The solvent was removed under reduced pressure, and the residue was triturated with hot methanol to give 3.22 g of the title compound. The mother liquor was evaporated and purified by flash column chromatography (dichloromethane/methanol 30:1) to give a second crop (0.52 g) of the product as a light yellow fine powder (3.74 g, 98% combined yield). ^1^H NMR (400 MHz, DMSO-*d*_6_): δ 12.91 (s, 1H), 8.06 (d, *J* = 8.4 Hz, 1H), 7.73 (d, *J* = 1.6 Hz, 1H), 7.54 (dd, *J* = 8.4, 1.6 Hz, 1H), 7.51–7.43 (m, 2H), 7.47–7.38 (m, 2H), 7.40–7.31 (m, 1H), 5.43 (s, 2H); MS (ESI): *m*/*z* calcd for C_15_H_11_N_3_O_5_: 313.07. Found: 312.0 [M – H]^−^.

#### 4.2.37 5-(4-Amino-3-(benzyloxy)phenyl)-1,3,4-oxadiazol-2(3*H*)-one (**9s14**)

Prepared according to the typical procedure B from **8s14** (523 mg, 1.67 mmol); white solid; (269 mg, 57% yield). ^1^H NMR (400 MHz, DMSO-*d*_6_): δ 12.21 (s, 1H), 7.55–7.47 (m, 2H), 7.45–7.35 (m, 2H), 7.37–7.28 (m, 1H), 7.21 (d, *J* = 1.9 Hz, 1H), 7.16 (dd, *J* = 8.2, 1.8 Hz, 1H), 6.73 (d, *J* = 8.2 Hz, 1H), 5.54 (s, 2H), 5.18 (s, 2H); MS (ESI): *m*/*z* calcd for C_15_H_13_N_3_O_3_: 283.09. Found: 283.9 [M + H]^+^.

#### 4.2.38 5-(2-Amino-4-(benzyloxy)benzo[*d*]thiazol-6-yl)-1,3,4-oxadiazol-2(3*H*)-one (**10s14**)

Prepared according to the typical procedure C from aniline **9s14** (201 mg, 0.709 mmol); yellow solid (200 mg, 83% yield). ^1^H NMR (400 MHz, DMSO-*d*_6_): δ 12.49 (s, 1H), 7.97 (s, 2H), 7.80 (d, *J* = 1.5 Hz, 1H), 7.52–7.48 (m, 2H), 7.45–7.38 (m, 2H), 7.38–7.33 (m, 1H), 7.29 (d, *J* = 1.5 Hz, 1H), 5.27 (s, 2H); MS (ESI): *m*/*z* calcd for C_16_H_12_N_4_O_3_S_3_: 340.06. Found: 339.1 [M – H]^−^.

#### 4.2.39 *N*-(4-(Benzyloxy)-6-(5-oxo-4,5-dihydro-1,3,4-oxadiazol-2-yl)benzo[*d*]thiazol-2-yl)-3,4-dichloro-5-methyl-1*H*-pyrrole-2-carboxamide (**14**)

Prepared according to the typical procedure D from the 2-aminobenzothiazole **10s14** (146 mg, 430 mmol); brown solid (200 mg, 90% yield). ^1^H NMR (400 MHz, DMSO-*d*_6_): δ 12.61 (s, 1H), 12.22 (s, 1H), 12.14 (s, 1H), 8.17–8.04 (m, 1H), 7.61–7.49 (m, 2H), 7.50–7.32 (m, 4H), 5.34 (s, 2H), 2.26 (s, 3H); ^13^C NMR (101 MHz, DMSO-*d*_6_): δ 158.88, 156.37, 154.45, 153.84, 150.75, 140.91, 136.45, 133.65, 129.96, 128.47, 128.31, 128.16, 119.76, 116.75, 115.63, 112.04, 109.94, 104.98, 70.21, 11.04; HRMS (ESI) *m*/*z*: [M + H]^+^ calcd for C_22_H_16_Cl_2_N_5_O_4_S 516.0300; found 516.0296; HPLC purity (254 nm): 98%.

#### 4.2.40 *tert*-Butyl 2-(4-(Benzyloxy)-2-(3,4-dichloro-5-methyl-1*H*-pyrrole-2-carboxamido)benzo[*d*]thiazole-6-carbonyl)hydrazine-1-carboxylate (**11s15**)

To a suspension of **1** (400 mg, 0.840 mmol) in DMF (20 mL), TBTU (324 mg, 1.00 mmol) and *N*-methylmorpholine (0.18 mL, 1.68 mmol) were added. After stirring the reaction mixture for 30 min, *tert*-butyl carbazate (122 mg, 0.923 mmol) was added. The resulting solution was stirred at 22 °C for 8 h; then, the volatiles were removed under reduced pressure. The residue was triturated with water and ethyl acetate, and the precipitated product was collected (468 mg, 94% yield). ^1^H NMR (400 MHz, DMSO-*d*_6_): δ 12.23 (s, 1H), 12.15 (s, 1H), 10.31–10.22 (m, 1H), 8.97 (s, 1H), 8.13 (s, 1H), 7.96 (s, 1H), 7.62 (s, 1H), 7.55 (d, *J* = 7.0 Hz, 2H), 7.49–7.34 (m, 3H), 5.32 (s, 2H), 2.26 (s, 3H), 1.45 (s, 9H); MS (ESI): *m*/*z* calcd for C_26_H_25_Cl_2_N_5_O_5_S: 589.10. Found: 588.1 [M – H]^−^.

#### 4.2.41 *N*-(4-(Benzyloxy)-6-(hydrazinecarbonyl)benzo[*d*]thiazol-2-yl)-3,4-dichloro-5-methyl-1*H*-pyrrole-2-carboxamide Hydrochloride (**15**)

Boc-protected compound **11s15** (400 mg, 0.677 mmol) was suspended in THF (5.0 mL), treated with 4 M HCl in dioxane (2.0 mL), and stirred at 22 °C overnight. The precipitate was collected and washed with THF to obtain the title compound as a white solid (260 mg, 73% yield). ^1^H NMR (400 MHz, DMSO-*d*_6_): δ 12.36 (s, 1H), 12.24 (s, 1H), 11.81 (s, 1H), 10.70 (s, 3H), 8.25 (d, *J* = 1.5 Hz, 1H), 7.72 (d, *J* = 1.5 Hz, 1H), 7.60–7.51 (m, 2H), 7.49–7.40 (m, 2H), 7.43–7.34 (m, 1H), 5.36 (s, 2H), 2.26 (s, 3H); HRMS (ESI) *m*/*z*: [M – Cl]^−^ calcd for C_21_H_18_Cl_2_N_5_O_3_S 490.0502; found 490.0506; HPLC purity (254 nm): 95%.

#### 4.2.42 Methyl 4-(Benzyloxy)-2-bromobenzo[*d*]thiazole-6-carboxylate (**19s18**): Typical Procedure K for the Sandmeyer Reaction of 2-Aminobenzothiazoles

Methyl 2-aminobenzo[*d*]thiazole-6-carboxylate (**18s18**) (1.39 g, 4.42 mmol) and CuBr_2_ (1.98 g, 8.85 mmol) were dissolved in acetonitrile (30 mL) and *tert*-butyl nitrite (1.05 mL, 8.85 mmol) was added dropwise at 0 °C. The mixture was stirred at 22 °C overnight; then, the volatiles were removed under reduced pressure. The residue was partitioned between EtOAc (30 mL) and brine (30 mL), and the organic layer was washed with brine, dried (Na_2_SO_4_), and concentrated to obtain a light brown solid (1.27 g, 76% yield). ^1^H NMR (400 MHz, DMSO-*d*_6_): δ 8.39 (d, *J* = 1.5 Hz, 1H), 7.65 (d, *J* = 1.5 Hz, 1H), 7.57–7.49 (m, 2H), 7.49–7.39 (m, 2H), 7.42–7.33 (m, 1H), 5.38 (s, 2H), 3.90 (s, 3H).

#### 4.2.43 Methyl 4-(Benzyloxy)-2-(methylamino)benzo[*d*]thiazole-6-carboxylate: Typical Procedure L for the Nucleophilic Substitution of 2-Bromobenzothiazoles

The Sandmeyer product **19s18** (1.27 g, 3.36 mmol) was dissolved in THF (100 mL), and methylamine (40% aqueous solution; 3.0 mL, 34 mmol) was added; the mixture was stirred at 22 °C overnight and concentrated under reduced pressure. The residue was partitioned between NH_4_Cl(aq) and EtOAc. The organic layer was dried (Na_2_SO_4_) and concentrated to give the title product (850 mg, 77% yield). ^1^H NMR (400 MHz, DMSO-*d*_6_) δ 8.35 (q, *J* = 4.7 Hz, 1H), 7.99 (d, *J* = 1.5 Hz, 1H), 7.53–7.46 (m, 2H), 7.47–7.36 (m, 3H), 7.38–7.29 (m, 1H), 5.28 (s, 2H), 3.82 (s, 3H), 2.96 (d, *J* = 4.7 Hz, 3H).

#### 4.2.44 4-(Benzyloxy)-2-(methylamino)benzo[*d*]thiazole-6-carboxylic Acid

Prepared according to the typical procedure E from methyl 4-(benzyloxy)-2-(methylamino)benzo[*d*]thiazole-6-carboxylate (850 mg, 2.59 mmol); white solid (750 mg, 92% yield). ^1^H NMR (400 MHz, DMSO-*d*_6_): δ 12.67 (s, 1H), 8.34 (q, *J* = 4.5 Hz, 1H), 7.94 (d, *J* = 1.5 Hz, 1H), 7.53–7.45 (m, 2H), 7.46–7.36 (m, 3H), 7.38–7.29 (m, 1H), 5.28 (s, 2H), 2.96 (d, *J* = 4.5 Hz, 3H).

#### 4.2.45 4-Methoxybenzyl 4-(Benzyloxy)-2-(methylamino)benzo[*d*]-thiazole-6-carboxylate (**20s18**): Typical Procedure M for the Synthesis of 4-Methoxybenzyl Esters

A solution of 4-(benzyloxy)-2-(methylamino)benzo[*d*]thiazole-6-carboxylic acid (750 mg, 2.38 mmol) in DMF (8 mL) was treated with K_2_CO_3_ (495 mg, 3.58 mmol) and 4-methoxybenzyl chloride (0.39 mL, 2.85 mmol). The resulting mixture was stirred at 22 °C overnight. After concentration under reduced pressure, the residue was partitioned between EtOAc and water, and the organic layer was dried over Na_2_SO_4_ and concentrated. The title compound was obtained after trituration with ether; beige solid (850 mg, 82% yield); ^1^H NMR (400 MHz, DMSO-*d*_6_): δ = 8.36 (q, *J* = 4.7 Hz, 1H), 7.98 (d, *J* = 1.5 Hz, 1H), 7.52–7.44 (m, 2H), 7.44 (d, *J* = 1.6 Hz, 1H), 7.44–7.34 (m, 4H), 7.38–7.28 (m, 1H), 5.28 (s, 2H), 5.23 (s, 2H), 3.76 (s, 3H), 2.95 (d, *J* = 4.6 Hz, 3H) ^13^C NMR (101 MHz, DMSO-*d*_6_): δ 165.43, 159.16, 148.06, 146.83, 137.08, 131.43, 129.90, 128.41, 128.23, 127.86, 127.83, 122.17, 116.03, 113.86, 110.77, 70.18, 65.79, 55.12, 30.80.

#### 4.2.46 4-Methoxybenzyl 4-(Benzyloxy)-2-(3,4-dichloro-*N*,5-dimethyl-1*H*-pyrrole-2-carboxamido)benzo[*d*]thiazole-6-carboxylate (**21s18**)

Prepared according to the typical procedure D from **20s18** (850 mg, 1.96 mmol); gray solid (831 mg, 73% yield); ^1^H NMR (400 MHz, DMSO-*d*_6_): δ 12.41 (s, 1H), 8.29 (d, *J* = 1.5 Hz, 1H), 7.59 (d, *J* = 1.5 Hz, 1H), 7.56–7.49 (m, 2H), 7.47–7.30 (m, 5H), 6.97 (d, *J* = 8.7 Hz, 2H), 5.41 (s, 2H), 5.29 (s, 2H), 3.79 (s, 3H), 3.77 (s, 3H), 2.25 (s, 3H).

#### 4.2.47 4-(Benzyloxy)-2-(3,4-dichloro-*N*,5-dimethyl-1*H*-pyrrole-2-carboxamido)benzo[*d*]thiazole-6-carboxylic acid (**18**): Typical Procedure N for Deprotection of 4-Methoxybenzyl and *tert*-Butyl Esters

4-Methoxybenzyl ester **21s18** (120 mg, 0.20 mmol) was dissolved in 1 M HCl in acetic acid and stirred at 22 °C overnight. The volatiles were removed under reduced pressure, and the solid residue was triturated with diethyl ether to give the title compound as a brown solid (20 mg, 20% yield). ^1^H NMR (400 MHz, DMSO-*d*_6_): δ = 13.01 (s, 1H), 12.41 (s, 1H), 8.25 (d, *J* = 1.4 Hz, 1H), 7.59 (d, *J* = 1.4 Hz, 1H), 7.57–7.50 (m, 2H), 7.47–7.38 (m, 2H), 7.40–7.30 (m, 1H), 5.40 (s, 2H), 3.80 (s, 3H), 2.26 (s, 3H); HRMS (ESI) *m*/*z*: [M – H]^−^ calcd for C_22_H_16_Cl_2_N_3_O_4_S 488.0244; found 488.0242; HPLC purity (254 nm): 96%.

#### 4.2.48 4-Methoxybenzyl 4-(Benzyloxy)-2-(3,4-dichloro-5-(phthalimidomethyl)-*N*-methyl-1*H*-pyrrole-2-carboxamido)benzo-[*d*]thiazole-6-carboxylate (**22s19**)

A suspension of 3,4-dichloro-5-(phthalimidomethyl)-1*H*-pyrrole-2-carboxylic acid^36^ (63 mg, 0.186 mmol) in SOCl_2_ (1 mL) was refluxed for 1 h; then, the reaction mixture was concentrated under reduced pressure to get a red tinted white solid. To this, crude acyl chloride **20s18** (81 mg, 0.186 mmol) and toluene (64 mL) were added and the resulting suspension was refluxed overnight. Upon cooling, the precipitate was collected, washed with toluene, and air-dried to get the product as a white crystalline solid (112 mg, 80% yield). ^1^H NMR (400 MHz, DMSO-*d*_6_): δ = 12.67 (s, 1H), 8.27 (s, 1H), 7.94–7.89 (m, 2H), 7.89–7.84 (m, 2H), 7.57 (s, 1H), 7.54–7.48 (m, 2H), 7.45–7.37 (m, 4H), 7.37–7.31 (m, 1H), 6.96 (d, *J* = 8.7 Hz, 2H), 5.39 (s, 2H), 5.28 (s, 2H), 4.81 (s, 2H), 3.76 (s, 3H), 3.74 (s, 3H); ^13^C NMR (101 MHz, DMSO-*d*_6_): δ 167.32, 165.26, 161.51, 159.22, 150.49, 141.77, 136.78, 134.53, 133.92, 131.75, 129.98, 128.50, 128.02, 127.95, 127.71, 127.66, 127.39, 126.12, 123.24, 118.77, 116.36, 113.88, 112.92, 109.90, 108.67, 70.30, 66.18, 55.14, 38.17, 33.04.

#### 4.2.49 (5-((4-(Benzyloxy)-6-carboxybenzo[*d*]thiazol-2-yl)(methyl)-carbamoyl)-3,4-dichloro-1*H*-pyrrol-2-yl)methanaminium Chloride (**19**)

To phthalimide **22s19** (100 mg, 0.132 mmol) in dry EtOH (2.6 mL) was added 80% hydrazine hydrate (85 μL, 1.35 mmol) and the suspension was stirred at 50 °C for 40 min. The reaction mixture was concentrated under reduced pressure, suspended in MeOH (5 mL), treated with 37% HCl(aq) (3 drops), and evaporated to get a white solid; ^1^H NMR (400 MHz, DMSO-*d*_6_): δ 12.54 (s, 1H), 11.25 (s, 1H), 10.66–9.74 (br s, 2H), 9.02 (t, *J* = 5.0 Hz, 1H), 8.30 (d, *J* = 1.4 Hz, 1H), 7.75–7.70 (m, 1H), 7.65–7.56 (m, 3H), 7.55–7.49 (m, 3H), 7.45–7.32 (m, 6H), 6.97 (d, *J* = 8.7 Hz, 2H), 5.41 (s, 2H), 5.29 (s, 2H), 4.47 (d, *J* = 5.0 Hz, 2H), 3.79 (s, 3H), 3.77 (s, 3H). The solid was suspended in 96% EtOH, stirred at 80 °C overnight, concentrated, and triturated with methanol to get **23s19** phthalhydrazide salt as an off-white solid (68 mg, 65%); ^1^H NMR (400 MHz, DMSO-d_6_): δ 12.67 (s, 1H), 11.66 (s, 1H), 8.31 (d, *J* = 1.3 Hz, 1H), 8.24 (m, 2H), 7.89 (m, 2H), 7.61 (d, *J* = 1.3 Hz, 1H), 7.56–7.49 (m, 2H), 7.47–7.32 (m, 5H), 6.97 (d, *J* = 8.7 Hz, 2H), 5.41 (s, 2H), 5.30 (s, 2H), 4.07 (s, 2H), 3.81 (s, 3H), 3.77 (s, 3H). This solid was suspended in 1 M solution of HCl in AcOH (5 mL), stirred at 22 °C overnight, and then concentrated under reduced pressure. The crude product in the form of phthalhydrazide salt was triturated with a fresh solution of HCl in acetone (5 drops of 37% HCl(aq) added to 5 mL of acetone) and washed with acetone to get the chloride salt 19 as a white solid (41 mg, 57% yield). ^1^H NMR (400 MHz, DMSO-*d*_6_): δ 13.05 (s, 1H), 12.77 (s, 1H), 8.32 (s, 3H), 8.27 (d, *J* = 0.7 Hz, 1H), 7.61 (d, *J* = 0.7 Hz, 1H), 7.54 (d, *J* = 7.2 Hz, 2H), 7.43 (t, *J* = 7.4 Hz, 2H), 7.36 (t, *J* = 7.1 Hz, 1H), 5.41 (s, 2H), 4.08 (s, 2H), 3.82 (s, 3H); HRMS (ESI) *m*/*z*: [M + H]^+^ calcd for C_22_H_19_Cl_2_N_4_O_4_S 505.0499; found 505.0488; HPLC purity (254 nm): 98%.

#### 4.2.50 Methyl 4-(1-Phenylethoxy)-2-bromobenzo[*d*]thiazole-6-carboxylate (**19s20**)

Prepared according to the typical procedure K from **18s20** (1.58 g, 4.32 mmol); light brown solid (1.7 g, 90% yield); ^1^H NMR (400 MHz, DMSO-*d*_6_): δ 8.30 (d, *J* = 1.5 Hz, 1H), 7.52–7.42 (m, 3H), 7.36 (t, *J* = 7.6 Hz, 2H), 7.32–7.22 (m, 1H), 3.83 (s, 3H), 1.66 (d, *J* = 6.3 Hz, 3H).

#### 4.2.51 4-Methoxybenzyl 4-(1-Phenylethoxy)-2-(methylamino)benzo[*d*]thiazole-6-carboxylate (**20s20**)

##### 4.2.51.1 Methyl 4-(1-Phenylethoxy)-2-(methylamino)benzo[*d*]thiazole-6-carboxylate

Prepared according to the typical procedure L from **19s20** (1.60 g, 4.08 mmol); light yellow solid (852 mg, 61% yield). ^1^H NMR (400 MHz, DMSO-d_6_): δ 8.40 (q, *J* = 4.7 Hz, 1H), 7.92 (d, *J* = 1.6 Hz, 1H), 7.48–7.40 (m, 2H), 7.38–7.29 (m, 2H), 7.28 (d, *J* = 1.6 Hz, 1H), 7.29–7.19 (m, 1H), 5.79 (q, *J* = 6.4 Hz, 1H), 3.76 (s, 3H), 3.00 (d, *J* = 4.7 Hz, 3H), 1.59 (d, *J* = 6.4 Hz, 3H).

##### 4.2.51.2 4-(1-Phenylethoxy)-2-(methylamino)benzo[*d*]thiazole-6-carboxylic Acid

Prepared according to the typical procedure E from methyl 4-(1-phenylethoxy)-2-(methylamino)benzo[*d*]-thiazole-6-carboxylate (798 mg, 2.33 mmol); beige solid (750 mg, 98% yield). ^1^H NMR (400 MHz, DMSO-*d*_6_): δ 8.45–8.34 (m, 1H), 7.88 (d, *J* = 1.5 Hz, 1H), 7.48–7.41 (m, 2H), 7.33 (dd, *J* = 8.3, 6.7 Hz, 2H), 7.27 (d, *J* = 1.5 Hz, 1H), 7.27–7.22 (m, 1H), 5.77 (q, *J* = 6.4 Hz, 1H), 3.00 (d, *J* = 3.9 Hz, 3H), 1.60 (d, *J* = 6.4 Hz, 3H).

#### 4.2.52 4-Methoxybenzyl 4-(1-Phenylethoxy)-2-(methylamino)benzo[*d*]thiazole-6-carboxylate (**20s20**)

Prepared according to the typical procedure M from 4-(1-phenylethoxy)-2-(methylamino)benzo[*d*]thiazole-6-carboxylic acid (703 mg, 2.14 mmol); white solid (594 mg, 62% yield). ^1^H NMR (400 MHz, DMSO-*d*_6_): δ 8.39 (q, *J* = 4.7 Hz, 1H), 7.90 (d, *J* = 1.6 Hz, 1H), 7.45–7.38 (m, 2H), 7.39–7.25 (m, 5H), 7.28–7.19 (m, 1H), 6.95 (d, *J* = 8.7 Hz, 2H), 5.74 (q, *J* = 6.4 Hz, 1H), 5.20 (d, *J* = 12.1 Hz, 1H), 5.15 (d, *J* = 12.1 Hz, 1H), 3.76 (s, 3H), 2.99 (d, *J* = 4.7 Hz, 3H), 1.59 (d, *J* = 6.3 Hz, 3H).

#### 4.2.53 4-Methoxybenzyl 2-(3,4-Dichloro-*N*,5-dimethyl-1*H*-pyrrole-2-carboxamido)-4-(1-phenylethoxy)benzo[*d*]thiazole-6-carboxylate (**21s20**)

Prepared according to the typical procedure D from **20s20** (365 mg, 0.814 mmol); white solid (213 mg, 44% yield). ^1^H NMR (400 MHz, DMSO-*d*_6_): δ 12.42 (s, 1H), 8.22 (d, *J* = 1.5 Hz, 1H), 7.50–7.41 (m, 3H), 7.38 (d, *J* = 8.7 Hz, 2H), 7.38–7.28 (m, 2H), 7.30–7.21 (m, 1H), 6.97 (d, *J* = 8.7 Hz, 2H), 5.85 (q, *J* = 6.4 Hz, 1H), 5.25 (d, *J* = 12.1 Hz, 1H), 5.21 (d, *J* = 12.1 Hz, 1H), 3.85 (s, 3H), 3.77 (s, 3H), 2.26 (s, 3H), 1.66 (d, *J* = 6.4 Hz, 3H).

#### 4.2.54 2-(3,4-Dichloro-*N*,5-dimethyl-1*H*-pyrrole-2-carboxamido)-4-hydroxybenzo[*d*]thiazole-6-carboxylic Acid (**20**)

The above 4-methoxybenzyl ester **21s20** (48 mg, 0.08 mmol) was dissolved in dichloromethane (2 mL), and 1 M solution of SnCl_4_ in dichloromethane (0.08 mL, 0.08 mmol) was added. The reaction mixture was stirred at 22 °C for 2 h, water was added to the mixture, and the light purple precipitate of product **20** was filtered off (30 mg, 94% yield). ^1^H NMR (400 MHz, DMSO-*d*_6_): δ 12.85 (s, 1H), 12.40 (s, 1H), 10.27 (s, 1H), 8.05 (d, *J* = 1.6 Hz, 1H), 7.45 (d, *J* = 1.7 Hz, 1H), 3.80 (s, 3H), 2.26 (s, 3H); HRMS (ESI) *m*/*z*: [M + H]^+^ calcd for C_15_H_12_Cl_2_N_3_O_4_S 399.9926; found 399.9942; HPLC purity (254 nm): 96%.

#### 4.2.55 4-Methoxybenzyl 2-Aminobenzo[*d*]thiazole-6-carboxylate (**18s21**)

Prepared according to the typical procedure M from 2-aminobenzo[*d*]thiazole-6-carboxylic acid (1.00 g, 5.16 mmol); light yellow solid (730 mg 45% yield). ^1^H NMR (400 MHz, DMSO-*d*_6_): δ 8.84–8.81 (m, 1H), 8.11–8.07 (m, 2H), 7.45 (d, *J* = 8.7 Hz, 2H), 6.97 (d, *J* = 8.7 Hz, 2H), 5.32 (s, 2H), 3.77 (s, 3H); MS (ESI): *m*/*z* calcd for C_16_H_14_N_2_O_3_S: 314.07. Found: 313.0 [M – H]^−^.

#### 4.2.56 4-Methoxybenzyl 2-Bromobenzo[*d*]thiazole-6-carboxylate (**19s21**)

Prepared according to the typical procedure K from amine **18s21** (232 mg, 0.740 mmol); orange powder (280 mg, 100% yield). ^1^H NMR (400 MHz, DMSO-*d*_6_): δ 8.87–8.79 (m, 1H), 8.14–8.04 (m, 2H), 7.45 (d, *J* = 8.6 Hz, 2H), 6.97 (d, *J* = 8.6 Hz, 2H), 5.32 (s, 2H), 3.77 (s, 3H); MS (ESI): *m*/*z* calcd for C_16_H_12_BrNO_3_S: 376.97. Found: 398.8 [M + Na – 2H]^−^.

#### 4.2.57 4-Methoxybenzyl 2-((2-Methoxyethyl)amino)benzo[*d*]thiazole-6-carboxylate (**20s21**)

Prepared according to the typical procedure L from **19s21** (451 mg, 1.19 mmol) and 2-methoxyethylamine; beige solid (244 mg, 55% yield). ^1^H NMR (400 MHz, DMSO-*d*_6_): δ 8.54 (t, *J* = 5.1 Hz, 1H), 8.30 (d, *J* = 1.7 Hz, 1H), 7.82 (dd, *J* = 8.4, 1.9 Hz, 1H), 7.45–7.38 (m, 3H), 6.96 (d, *J* = 8.7 Hz, 1H), 5.25 (s, 2H), 3.76 (s, 3H), 3.61–3.48 (m, 4H), 3.28 (s, 3H); MS (ESI): *m*/*z* calcd for C_19_H_20_N_2_O_4_S: 372.11. Found: 371.0 [M – H]^−^.

#### 4.2.58 4-Methoxybenzyl 2-(3,4-Dichloro-N-(2-methoxyethyl)-5-methyl-1*H*-pyrrole-2-carboxamido)benzo[*d*]thiazole-6-carboxylate (**21s21**)

Prepared according to the typical procedure D from **20s21** (123 mg, 0.331 mmol); pink solid 96 mg (56% yield). ^1^H NMR (400 MHz, DMSO-*d*_6_): δ 12.51 (s, 1H), 8.68 (d, *J* = 1.7 Hz, 1H), 8.03 (dd, *J* = 8.6, 1.8 Hz, 1H), 7.90 (d, *J* = 8.5 Hz, 1H), 7.45 (d, *J* = 8.7 Hz, 2H), 6.97 (d, *J* = 8.7 Hz, 2H), 5.31 (s, 2H), 4.54 (t, *J* = 4.8 Hz, 2H), 3.77 (s, 3H), 3.60 (t, *J* = 4.8 Hz, 2H), 3.07 (s, 3H), 2.25 (s, 3H); MS (ESI): *m*/*z* calcd for C_25_H_23_Cl_2_N_3_O_5_S: 547.07. Found: 546.0 [M – H]^−^.

#### 4.2.59 2-(3,4-Dichloro-*N*-(2-methoxyethyl)-5-methyl-1*H*-pyrrole-2-carboxamido)benzo[*d*]thiazole-6-carboxylic Acid (**21**)

Prepared according to the typical procedure N from ester **21s21** (56 mg, 0.108 mmol); light pink powder (19 mg, 41% yield). ^1^H NMR (400 MHz, DMSO-*d*_6_): δ 12.98 (s, 1H), 12.50 (s, 1H), 8.63 (d, *J* = 1.6 Hz, 1H), 8.02 (dd, *J* = 8.5, 1.6 Hz, 1H), 7.88 (d, *J* = 8.5 Hz, 1H), 4.55 (t, *J* = 4.8 Hz, 2H), 3.61 (t, *J* = 4.8 Hz, 2H), 3.08 (s, 3H), 2.25 (s, 3H); HRMS (ESI) *m*/*z*: [M + H]^+^ calcd for C_17_H_16_Cl_2_N_3_O_4_S 428.0239; found: 428.0229; HPLC purity (254 nm): 97%.

#### 4.2.60 *tert*-Butyl 4-Nitrobenzoate (**13s**)

To a solution of 4-nitrobenzoic acid (1.67 g, 10.0 mmol) in pyridine (50 mL), tosyl chloride (3.8 g, 20 mmol) was added and the reaction mixture was cooled to 0 °C. *tert*-Butanol (1.48 g, 20 mmol) was added, and the mixture was stirred for 2 h at 0 °C and 16 h at 22 °C. The volatiles were removed under reduced pressure, and the mixture was partitioned between DCM (150 mL) and saturated NaHCO_3_ solution (150 mL). The organic layer was washed twice with saturated NaHCO_3_ solution, and the combined NaHCO_3_ layers were extracted once with DCM (100 mL). The combined organic layers were washed with brine and subsequently with 1 M NaHSO_4_ (3 × 100 mL), dried over Na_2_SO_4_, filtered, and concentrated in vacuo to give a yellow solid (2.0 g, 90% yield). ^1^H NMR (500 MHz, CDCl_3_): δ 8.28–8.23 (m, 2H), 8.17–8.12 (m, 2H), 1.62 (s, 9H).

#### 4.2.61 *tert*-Butyl 4-Aminobenzoate (**14s**)

Prepared according to the typical procedure I from **13s** (2.0 g, 9 mmol); off-white solid (1.60 g, 92% yield). ^1^H NMR (500 MHz, CDCl_3_): δ 7.80 (d, *J* = 8.2 Hz, 2H), 6.62 (d, *J* = 8.1 Hz, 2H), 3.93 (bs, 2H), 1.56 (s, 9H).

#### 4.2.62 *tert*-Butyl 2-Aminobenzo[*d*]thiazole-6-carboxylate (**15s**)

Prepared according to the typical procedure C from **14s** (1.60 g, 8.28 mmol); dark yellow solid (1.70 g, 82% yield). ^1^H NMR (500 MHz, DMSO-*d*_6_): δ 8.21 (d, *J* = 1.8 Hz, 1H), 7.87 (s, 2H), 7.75 (dd, *J* = 8.4 Hz, 1.8 Hz, 1H), 7.34 (d, *J* = 8.4 Hz, 1H), 1.54 (s, 9H). ^13^C NMR (126 MHz, DMSO-*d*_6_): δ 169.99, 165.39, 157.07, 131.45, 127.44, 123.96, 122.84, 117.42, 80.60, 28.37. MS (ESI): *m*/*z* calcd for C_12_H_14_N_2_O_2_S: 250.08. Found: 251.2 [M + H]^+^.

#### 4.2.63 *tert*-Butyl 2-(3,4-Dichloro-5-methyl-1*H*-pyrrole-2-carboxamido)benzo[*d*]thiazole-6-carboxylate (**16s**)

Prepared according to the typical procedure D from 2-amino-benzothiazole **15s** (150 mg, 0.597 mmol); gray solid (145 mg, 57% yield). ^1^H NMR (400 MHz, DMSO-d_6_): δ 12.38 (s, 1H), 11.93 (s, 1H), 8.59 (s, 1H), 7.97 (dd, *J* = 8.5 Hz, 1.8 Hz, 1H), 7.81 (s, 1H), 2.27 (s, 3H), 1.58 (s, 9H).

#### 4.2.64 *tert*-Butyl 3-Benzyl-2-((3,4-dichloro-5-methyl-1*H*-pyrrole-2-carbonyl)imino)-2,3-dihydrobenzo[*d*]thiazole-6-carboxylate (**17s22**): Typical Procedure O for Benzothiazole N-Alkylation

To a suspension of **16s** (100 mg, 0.235 mmol), KI (20 mg, 0.12 mmol), and NaHCO_3_ (24 mg, 0.28 mmol) in DMF (5 mL) was added benzyl bromide (31 μL, 0.26 mmol), and the resulting mixture was stirred at 60 °C overnight. The volatiles were removed under reduced pressure, and the residue was triturated successively with water and methanol to obtain the title compound as a gray solid (98 mg, 81% yield). ^1^H NMR (400 MHz, DMSO-*d*_6_): δ 12.09 (s, 1H), 8.46 (d, *J* = 1.7 Hz, 1H), 7.95 (dd, *J* = 8.6 Hz, 1.7 Hz, 1H), 7.64 (d, *J* = 8.6 Hz, 1H), 7.42–7.37 (m, 2H), 7.35–7.30 (m, 2H), 7.30–7.23 (m, 1H), 5.92 (s, 2H), 2.25 (s, 3H), 1.55 (s, 9H).

#### 4.2.65 3-Benzyl-2-((3,4-dichloro-5-methyl-1*H*-pyrrole-2-carbonyl)imino)-2,3-dihydrobenzo[*d*]thiazole-6-carboxylic Acid (**22**)

Prepared according to the typical procedure N from **17s22** (80 mg, 0.155 mmol); gray solid (60 mg, 84% yield). ^1^H NMR (400 MHz, DMSO-*d*_6_): δ 13.0 (s, 1H), 12.08 (s, 1H), 8.50 (s, 1H), 7.99 (d, *J* = 8.5 Hz, 1H), 7.64 (d, *J* = 8.5 Hz, 1H), 7.50–7.15 (m, 5H), 5.92 (s, 2H), 2.25 (s, 3H); ^13^C NMR (101 MHz, DMSO-*d*_6_): δ 167.15, 166.51, 165.67, 139.62, 135.71, 129.24, 128.75, 128.40, 127.73, 127.36, 126.32, 126.23, 124.65, 121.93, 114.41, 112.37, 109.68, 48.23, 10.92; ^1^H–^1^H NOE observed between benzylic hydrogens and benzothiazole 4-H; HRMS calcd for C_21_H_16_Cl_2_N_3_O_3_S: 460.0289. Found: 460.0287 [M + H]^+^; HPLC purity (254 nm): 99%.

#### 4.2.66 *tert*-Butyl 2-((3,4-Dichloro-5-methyl-1*H*-pyrrole-2-carbon-yl)imino)-3-(4-(methylsulfonyl)benzyl)-2,3-dihydroben-zo[*d*]thiazole-6-carboxylate (**17s23**)

Prepared according to the typical procedure O from **16s** (123 mg, 0.288 mmol) and 4-mesylbenzyl bromide (79 mg, 0.317 mmol); gray solid (125 mg, 73% yield). ^1^H NMR (400 MHz, DMSO-*d*_6_): δ 12.10 (s, 1H), 8.49 (d, *J* = 1.7 Hz, 1H), 7.96 (dd, *J* = 8.6 Hz, 1.7 Hz, 1H), 7.89 (d, *J* = 8.5 Hz, 2H), 7.70–7.55 (m, 3H), 6.03 (s, 2H), 3.17 (s, 3H), 2.25 (s, 3H), 1.55 (s, 9H).

#### 4.2.67 2-((3,4-Dichloro-5-methyl-1*H*-pyrrole-2-carbonyl)imino)-3-(4-(methylsulfonyl)benzyl)-2,3-dihydrobenzo[*d*]thiazole-6-carboxylic Acid (**23**)

Prepared according to the typical procedure N from *tert*-butyl ester **17s23** (100 mg, 0.169 mmol); gray solid (70 mg, 77% yield). ^1^H NMR (400 MHz, DMSO-d_6_): δ 13.08 (s, 1H), 12.08 (s, 1H), 8.53 (d, *J* = 1.7 Hz, 1H), 8.00 (dd, *J* = 8.6, 1.7 Hz, 1H), 7.89 (d, *J* = 8.5 Hz, 2H), 7.67–7.58 (m, 3H), 6.03 (s, 2H), 3.17 (s, 3H), 2.25 (s, 3H); HRMS (ESI) *m*/*z*: [M – H]^−^ calcd for C_22_H_16_Cl_2_N_3_O_5_S_2_ 535.9908; found 535.9913; HPLC purity (254 nm): 95%.

#### 4.2.68 *tert*-Butyl 2-((3,4-Dichloro-5-methyl-1*H*-pyrrole-2-carbon-yl)imino)-3-(2-(4-(methylsulfonyl)phenyl)-2-oxoethyl)-2,3-dihydrobenzo[*d*]thiazole-6-carboxylate (**17s24**)

Prepared according to the typical procedure O from **16s** (117 mg, 0.275 mmol) and 4-methylsulfonylphenacyl bromide (84 mg, 0.302 mmol); gray solid (120 mg, 70% yield). ^1^H NMR (400 MHz, DMSO-*d*_6_): δ 12.13 (s, 1H), 8.53 (d, *J* = 1.7 Hz, 1H), 8.40 (d, *J* = 8.5 Hz, 2H), 8.21 (d, *J* = 8.5 Hz, 2H), 7.98 (dd, *J* = 8.7 Hz, 1.7 Hz, 1H), 7.79 (d, *J* = 8.7 Hz, 1H), 6.33 (s, 2H), 2.20 (s, 3H), 1.58 (s, 9H).

#### 4.2.69 2-((3,4-Dichloro-5-methyl-1*H*-pyrrole-2-carbonyl)imino)-3-(2-(4-(methylsulfonyl)phenyl)-2-oxoethyl)-2,3-dihydro-benzo[*d*]thiazole-6-carboxylic Acid (**24**)

Prepared according to the typical procedure N from **17s24** (100 mg, 0.160 mmol); gray solid (48 mg, 53% yield). ^1^H NMR (400 MHz, DMSO-*d*_6_): δ 13.09 (s, 1H), 12.11 (s, 1H), 8.56 (d, *J* = 1.7 Hz, 1H), 8.40 (d, *J* = 8.6 Hz, 2H), 8.21 (d, *J* = 8.6 Hz, 2H), 8.03 (dd, *J* = 8.6 Hz, 1.7 Hz, 1H), 7.79 (d, *J* = 8.6 Hz, 1H), 6.33 (s, 2H), 3.36 (s, 3H), 2.19 (s, 3H); HRMS (ESI) *m*/*z*: [M + H]^+^ calcd for C_23_H_18_Cl_2_N_3_O_5_S_2_: 566.0014; found: 566.0007; HPLC purity (254 nm): 96%.

#### 4.2.70 *tert*-Butyl 2-((3,4-Dichloro-5-methyl-1*H*-pyrrole-2-carbon-yl)imino)-3-(2-oxo-2-phenylethyl)-2,3-dihydrobenzo[*d*]thiazole-6-carboxylate (**17s25**)

Prepared according to the typical procedure O from 16s (100 mg, 0.234 mmol) and phenacyl bromide (51 mg, 0.257 mmol); gray solid (80 mg, 63% yield). ^1^H NMR (400 MHz, DMSO-*d*_6_): δ 12.13 (s, 1H), 8.52 (d, *J* = 1.7 Hz, 1H), 8.20–8.13 (m, 2H), 7.97 (dd, *J* = 8.7 Hz, 1.7 Hz, 1H), 7.81–7.73 (m, 2H), 7.70–7.62 (m, 2H), 6.27 (s, 2H), 2.19 (s, 3H), 1.58 (s, 9H).

#### 4.2.71 2-((3,4-Dichloro-5-methyl-1*H*-pyrrole-2-carbonyl)imino)-3-(2-oxo-2-phenylethyl)-2,3-dihydrobenzo[*d*]thiazole-6-carboxylic Acid (**25**)

Prepared according to the typical procedure N from *tert*-butyl ester **17s25** (70 mg, 0.128 mmol); gray solid (10 mg, 16% yield). ^1^H NMR (400 MHz, DMSO-*d*_6_): δ 13.08 (s, 1H), 12.11 (s, 1H), 8.55 (d, *J* = 1.7 Hz, 1H), 8.16 (d, *J* = 7.6 Hz, 2H), 8.02 (dd, *J* = 8.6 Hz, 1.7 Hz, 1H), 7.84–7.72 (m, 2H), 7.70–7.61 (m, 2H), 6.28 (s, 2H), 2.19 (s, 3H); HRMS (ESI) *m*/*z*: [M + H]^+^ calcd for C_22_H_16_Cl_2_N_3_O_4_S 488.0239; found 488.0232; HPLC purity (254 nm): 95%.

#### 4.2.72 Ethyl 2-Bromobenzo[*d*]thiazole-6-carboxylate (**19s26**)

Prepared according to the typical procedure K from ethyl 2-aminobenzo[*d*]thiazole-6-carboxylate (2.95 g, 13.27 mmol); light brown solid (3.80 g, 100% yield). ^1^H NMR (400 MHz, DMSO-d_6_): δ 8.82 (dd, *J* = 1.6 Hz, 0.8 Hz, 1H), 8.14–8.07 (m, 1H), 8.12–8.04 (m, 1H), 4.36 (q, *J* = 7.1 Hz, 2H), 1.35 (t, *J* = 7.1 Hz, 3H).

#### 4.2.73 4-Methoxybenzyl 2-(Methylamino)benzo[*d*]thiazole-6-carboxylate (**20s26**)

##### 4.2.73.1 Ethyl 2-(Methylamino)benzo[*d*]thiazole-6-carboxylate

Prepared from the Sandmeyer product **19s26** (1.01 g, 3.53 mmol); beige solid (250 mg, 30% yield). ^1^H NMR (400 MHz, DMSO-*d*_6_): δ 8.35 (q, *J* = 5 Hz, 1H), 8.30 (d, *J* = 1.8 Hz, 1H), 7.83 (dd, *J* = 8.4 Hz, 1.8 Hz, 1H), 7.43 (d, *J* = 8.4 Hz, 1H), 4.29 (q, *J* = 7.1 Hz, 2H), 2.97 (d, *J* = 4.7 Hz, 3H), 1.32 (t, *J* = 7.1 Hz, 3H).

##### 4.2.73.2 2-(Methylamino)benzo[*d*]thiazole-6-carboxylic Acid

Prepared according to the typical procedure E from ethyl 2-(methylamino)benzo[*d*]thiazole-6-carboxylate (603 mg, 2.55 mmol); white solid (340 mg, 64% yield). ^1^H NMR (400 MHz, DMSO-*d*_6_): δ 12.90–12.43 (m, 1H), 8.50–8.05 (m, 2H), 8.10–7.67 (m, 1H), 7.59–7.26 (m, 1H), 3.04–2.86 (m, 3H); MS (ESI): *m*/*z* calcd for C_9_H_8_N_2_O_2_S: 208.03. Found: 208.8 [M + H]^+^.

#### 4.2.74 4-Methoxybenzyl 2-(Methylamino)benzo[*d*]thiazole-6-carboxylate (**20s26**)

Prepared according to the typical procedure M from 2-(methylamino)benzo[*d*]thiazole-6-carboxylic acid (327 mg, 1.57 mmol); off-white solid (155 mg, 30% yield). ^1^H NMR (400 MHz, DMSO-*d*_6_): δ 8.36 (q, *J* = 4.7 Hz, 1H), 8.31 (d, *J* = 1.8 Hz, 1H), 7.83 (dd, *J* = 8.5 Hz, 1.8 Hz, 1H), 7.46–7.38 (m, 3H), 6.96 (d, *J* = 8.7 Hz, 2H), 5.25 (s, 2H), 3.76 (s, 3H), 2.96 (d, *J* = 4.7 Hz, 3H).

#### 4.2.75 4-Methoxybenzyl 2-(3,4-Dichloro-*N*,5-dimethyl-1*H*-pyrrole-2-carboxamido)benzo[*d*]thiazole-6-carboxylate (**21s26**)

Prepared according to the typical procedure D from **20s26** (154 mg, 0.468 mmol); off-white solid (40 mg, 18% yield). ^1^H NMR (400 MHz, DMSO-*d*_6_): δ 12.43 (s, 1H), 8.70 (d, *J* = 1.7 Hz, 1H), 8.04 (dd, *J* = 8.5 Hz, 1.7 Hz, 1H), 7.93 (d, *J* = 8.5 Hz, 1H), 7.45 (d, *J* = 8.7 Hz, 2H), 6.97 (d, *J* = 8.7 Hz, 2H), 5.31 (s, 2H), 3.80 (s, 3H), 3.77 (s, 3H), 2.26 (s, 3H); MS (ESI): *m*/*z* calcd for C_23_H_19_Cl_2_N_3_O_3_S: 503.1. Found: 501.9 [M – H]^−^.

#### 4.2.76 2-(3,4-Dichloro-*N*,5-dimethyl-1*H*-pyrrole-2-carboxamido)-benzo[*d*]thiazole-6-carboxylic Acid (**26**)

Prepared according to the typical procedure N from PMB ester **21s26** (37 mg, 0.078 mmol); light brown solid (20 mg, 66% yield). ^1^H NMR (400 MHz, DMSO-*d*_6_): δ 13.0 (s, 1H), 12.43 (s, 1H), 8.65 (d, *J* = 1.7 Hz, 1H), 8.02 (dd, *J* = 8.5 Hz, 1.7 Hz, 1H), 7.91 (d, *J* = 8.5 Hz, 1H), 3.80 (s, 3H), 2.26 (s, 3H); HRMS (ESI) *m*/*z*: [M – H]^−^ calcd for C_15_H_10_Cl_2_N_3_O_3_S 381.9820; found 381.9825; HPLC purity (254 nm): 96%.

#### 4.2.77 Methyl 2-Amino-4-(1-phenylethoxy)benzo[*d*]thiazole-6-carboxylate (**5s27**)

Prepared according to the typical procedure F from **1s**(33) (682 mg, 3.04 mmol) and 1-chloro-1-phenylethan (0.50 mL; 3.65 mmol); white solid (131 mg, 13% yield). ^1^H NMR (400 MHz, DMSO-*d*_6_): δ 7.94 (s, 2H), 7.87 (d, *J* = 1.5 Hz, 1H), 7.45–7.39 (m, 2H), 7.33 (app t, *J* = 7.5 Hz, 2H), 7.27–7.23 (m, 2H), 5.73 (q, *J* = 6.3 Hz, 1H), 3.76 (s, 3H), 1.57 (d, *J* = 6.4 Hz, 3H).

#### 4.2.78 Methyl 2-(3,4-Dichloro-5-methyl-1*H*-pyrrole-2-carboxamido)-4-(1-phenylethoxy)benzo[*d*]thiazole-6-carboxylate (**6s27**)

Prepared according to the typical procedure D from **24s27** (250 mg, 0.762 mmol); white solid (210 mg, 55% yield). ^1^H NMR (400 MHz, DMSO-*d*_6_): δ 12.32 (s, 1H), 12.28 (s, 1H), 8.19 (s, 1H), 7.46 (d, *J* = 7.3 Hz, 2H), 7.40–7.33 (m, 3H), 7.29–7.24 (m, 1H), 5.81 (q, *J* = 6.5 Hz, 1H), 3.81 (s, 3H), 2.29 (s, 3H), 1.65 (d, *J* = 6.5 Hz, 3H); ^13^C NMR (101 MHz, DMSO-*d*_6_): δ 166.25, 160.33, 156.97, 149.52, 142.95, 133.39, 130.36, 129.10 (2C), 128.09, 126.03 (2C), 125.59, 117.21, 116.36, 116.33, 110.74, 110.52, 76.08, 52.59, 24.75, 11.55.

#### 4.2.79 2-(3,4-Dichloro-5-methyl-1*H*-pyrrole-2-carboxamido)-4-(1-phenylethoxy)benzo[*d*]thiazole-6-carboxylic Acid (**27**)

Prepared according to the typical procedure E from the methyl ester **6s27** (100 mg, 0.198 mmol); white solid (75 mg, 77% yield). Mp > 300 °C; ^1^H NMR (400 MHz, DMSO-*d*_6_): δ 12.87 (s, 1H), 12.29 (s, 2H), 8.15 (s, 1H), 7.49–7.43 (m, 2H), 7.40–7.32 (m, 3H), 7.26 (t, *J* = 7.3 Hz, 1H), 5.79 (q, *J* = 6.3 Hz, 1H), 2.28 (s, 3H), 1.64 (d, *J* = 6.3 Hz, 3H); ^13^C NMR (101 MHz, DMSO-*d*_6_): δ 166.88, 159.56, 156.65, 149.00, 142.56, 142.12, 132.81, 129.90, 128.61, 127.56, 126.51, 125.54, 116.86, 115.98, 115.76, 110.59, 109.98, 75.57, 24.31, 11.06; HRMS (ESI) *m*/*z*: [M + H]^+^ calcd for C_22_H_18_Cl_2_N_3_O_4_S 490.0395; found 490.0382; HPLC purity (254 nm): 96%.

#### 4.2.80 Methyl (*S*)-2-Amino-4-(1-phenylethoxy)benzo[*d*]thiazole-6-carboxylate (**24s*****S*****27**)

##### 4.2.80.1 Methyl (*S*)-4-Nitro-3-(1-phenylethoxy)benzoate

Prepared according to the typical procedure H from **2s** (2.52 g, 12.77 mmol) and (*R*)-1-phenylethanol (1.72 g,14.05 mmol); white solid (3.00 g, 78% yield). ^1^H NMR (400 MHz, CDCl_3_): δ 7.76 (d, *J* = 8.3 Hz, 1H), 7.64 (d, *J* = 1.5 Hz, 1H), 7.60 (dd, *J* = 8.3 Hz, 1.5 Hz, 1H), 7.44–7.40 (m, 2H), 7.39–7.33 (m, 2H), 7.31–7.26 (m, 1H), 5.54 (q, *J* = 6.4 Hz, 1H), 3.89 (s, 3H), 1.70 (d, *J* = 6.4 Hz, 3H).

##### 4.2.80.2 Methyl (*S*)-4-Amino-3-(1-phenylethoxy)benzoate

Prepared according to the typical procedure B from methyl (*S*)-4-nitro-3-(1-phenylethoxy)benzoate (3.00 g, 9.95 mmol); white solid (2.70 g, 100% yield). ^1^H NMR (400 MHz, DMSO-*d*_6_): δ 7.44 (d, *J* = 7.2 Hz, 2H), 7.34 (t, *J* = 7.5 Hz, 2H), 7.30 (dd, *J* = 8.2 Hz, 1.7 Hz, 1H), 7.25 (t, *J* = 7.3 Hz, 1H), 7.21 (d, *J* = 1.7 Hz, 1H), 6.62 (d, *J* = 8. Hz, 1H), 5.70 (s, 2H), 5.49 (q, *J* = 6.3 Hz, 1H), 3.68 (s, 3H), 1.56 (d, *J* = 6.3 Hz, 3H).

#### 4.2.81 Methyl (*S*)-2-Amino-4-(1-phenylethoxy)benzo[*d*]thiazole-6-carboxylate (**24s*****S*****27**)

Prepared according to the typical procedure C from methyl (*S*)-4-amino-3-(1-phenylethoxy)-benzoate (2.31 g, 8.53 mol); white powder (925 mg, 33% yield). ^1^H NMR (400 MHz, DMSO-*d*_6_): δ 8.02 (s, 2H), 7.88 (d, *J* = 1.5 Hz, 1H), 7.45–7.40 (m, 2H), 7.34 (t, *J* = 7.5 Hz, 2H), 7.28–7.22 (m, 2H), 5.73 (q, *J* = 6.3 Hz, 1H), 3.76 (s, 3H), 1.58 (d, *J* = 6.3 Hz, 3H); 68% ee determined by chiral HPLC analysis on a Kromasil 3-CelluCoat column (4.6 mm × 150 mm), eluent hexane/MeOH/*i*-PrOH = 90:5:5; *t*_R_ = 6.8 min (*S* enantiomer), 7.4 min (*R* enantiomer).

#### 4.2.82 Methyl (*S*)-2-(3,4-Dichloro-5-methyl-1*H*-pyrrole-2-carboxamido)-4-(1-phenylethoxy)benzo[*d*]thiazole-6-carboxylate (**25s*****S*****27**)

Prepared according to the typical procedure D from 2-aminobenzothiazole **24s*****S*****27** (253 mg, 0.77 mmol); white solid (220 mg, 57% yield). ^1^H NMR (400 MHz, DMSO-*d*_6_): *δ* 12.30 (s, 1H), 12.26 (s, 1H), 8.18 (s, 1H), 7.46 (d, *J* = 7.4 Hz, 2H), 7.43–7.33 (m, 3H), 7.26 (t, *J* = 7.3 Hz, 1H), 5.81 (q, *J* = 6.4 Hz, 1H), 3.81 (s, 3H), 2.28 (s, 3H), 1.65 (d, *J* = 6.4 Hz, 3H).

#### 4.2.83 (*S*)-2-(3,4-Dichloro-5-methyl-1*H*-pyrrole-2-carboxamido)-4-(1-phenylethoxy)benzo[*d*]thiazole-6-carboxylic Acid [**(*****S*****)-27**]

Prepared according to the typical procedure E from methyl ester **25s*****S*****27** (150 mg, 0.297 mmol); brown powder (125 mg, 86% yield). ^1^H NMR (400 MHz, DMSO-d_6_): δ = 12.85 (s, 1H), 12.26 (s, 2H), 8.15 (s, 1H), 7.46 (app d, *J* = 7.2 Hz, 2H), 7.40–7.32 (m, 3H), 7.26 (app t, *J* = 7.5 Hz, 1H), 5.79 (q, *J* = 6.3 Hz, 1H), 2.28 (s, 3H), 1.65 (d, *J* = 6.3 Hz, 3H); MS (ESI): *m*/*z* calcd for C_22_H_17_Cl_2_N_3_O_4_S: 489.03. Found 488.2 [M – H]^−^; HPLC purity (254 nm): 96% (sum of enantiomers); 74% ee determined by chiral HPLC analysis on a Kromasil 3-CelluCoat column (4.6 mm × 150 mm), eluent hexane/MeOH/0.1%TFA in *i*-PrOH = 90:5:5, flow rate: 1 mL/min; *t*_R_ = 7.7 min (*S* enantiomer), 9.5 min (*R* enantiomer). The absolute configuration of the major product was assigned based on the starting (*R*)-1-phenylethanol.

#### 4.2.84 Methyl (*R*)-2-Amino-4-(1-phenylethoxy)benzo[*d*]thiazole-6-carboxylate (**24sR27**)

##### 4.2.84.1 Methyl (*R*)-4-Nitro-3-(1-phenylethoxy)benzoate

Prepared according to the typical procedure H from **2s** (2.62 g, 13.28 mmol) and (*S*)-1-phenylethanol (1.79 g, 14,62 mmol); white solid (2.08 g, 52% yield). MS (ESI): *m*/*z* calcd for C_16_H_15_NO_5_: 301.09. Found: 300.3 [M – H]^−^.

##### 4.2.84.2 Methyl (*R*)-4-Amino-3-(1-phenylethoxy)benzoate

Prepared according to the typical procedure B from methyl (*R*)-4-nitro-3-(1-phenylethoxy)benzoate (2.05 g, 6.80 mmol); (1.42g, 77% yield). ^1^H NMR (400 MHz, DMSO-*d*_6_): δ 7.47–7.39 (m, 2H), 7.36–7.19 (m, 5H), 6.62 (d, *J* = 8.0 Hz, 1H), 5.70 (s, 2H), 5.48 (d, *J* = 6 Hz, 1H), 3.68 (s, 3H), 1.56 (d, *J* = 6 Hz, 3H); MS (ESI): *m*/*z* calcd for C_16_H_17_NO_5_: 271.12. Found: 272.2 [M + H]^+^.

#### 4.2.85 Methyl (*R*)-2-Amino-4-(1-phenylethoxy)benzo[*d*]thiazole-6-carboxylate (**24s*****R*****27**)

Prepared according to the typical procedure C from methyl (*R*)-4-amino-3-(1-phenylethoxy)-benzoate (1.36 g, 5.00 mmol); (443 mg, 27% yield). ^1^H NMR (400 MHz, DMSO-*d*_6_): δ 7.95 (s, 2H), 7.88 (d, *J* = 1.5 Hz, 1H), 7.44–7.41 (m, 2H), 7.34 (app t, *J* = 7.5 Hz, 2H), 7.29–7.20 (m, 2H), 5.73 (q, *J* = 6.3 Hz, 1H), 3.76 (s, 3H), 1.58 (d, *J* = 6.3 Hz, 3H); MS (ESI): *m*/*z* calcd for C_17_H_16_N_2_O_3_S: 328.09. Found: 327.3 [M – H]^−^; 88.6% ee determined by chiral HPLC analysis on a Kromasil 3-CelluCoat column (4.6 mm × 150 mm), eluent hexane/MeOH/*i*-PrOH = 90:5:5; *t*_R_ = 6.8 min (*S* enantiomer), 7.4 min (*R* enantiomer).

#### 4.2.86 Methyl (*R*)-2-(3,4-Dichloro-5-methyl-1*H*-pyrrole-2-carboxamido)-4-(1-phenylethoxy)benzo[*d*]thiazole-6-carboxylate (**25s*****R*****27**)

Prepared according to the typical procedure D from 2-aminobenzothiazole **24s*****R*****27** (240 mg, 0.73 mmol); (48 mg, 13% yield). ^1^H NMR (400 MHz, DMSO-*d*_6_): δ 12.30 (s, 1H), 12.26 (s, 1H), 8.19 (s, 1H), 7.46 (app d, *J* = 7.5 Hz, 2H), 7.40–7.33 (m, 3H), 7.29–7.24 (m, 1H), 5.81 (q, *J* = 6 Hz, 1H), 3.81 (s, 3H), 2.29 (s, 3H), 1.65 (d, *J* = 6 Hz, 3H).

#### 4.2.87 (*R*)-2-(3,4-Dichloro-5-methyl-1*H*-pyrrole-2-carboxamido)-4-(1-phenylethoxy)benzo[*d*]thiazole-6-carboxylic Acid [**(*****R*****)-27**]

Prepared according to the typical procedure E from the methyl ester **25s*****R*****27** (45 mg, 0.089 mmol); red-brown powder (26 mg, 61% yield). ^1^H NMR (400 MHz, DMSO-*d*_6_): δ 12.86 (s, 1H), 12.26 (s, 2H), 8.14 (d, *J* = 1.1 Hz, 1H), 7.46 (app d, *J* = 7.2 Hz, 2H), 7.39–7.32 (m, 3H)., 7.29–7.23 (m, 1H), 5.79 (q, *J* = 6.3 Hz, 1H), 2.28 (s, 3H), 1.65 (d, *J* = 6.3 Hz, 3H); HRMS (ESI) *m*/*z*: [M + H]^+^ calcd for C_22_H_18_Cl_2_N_3_O_4_S 490.0395; found 490.0389; HPLC purity (254 nm): 97% (sum of enantiomers); 63% ee determined by chiral HPLC analysis on a Kromasil 3-CelluCoat column (4.6 mm × 150 mm), eluent hexane/MeOH/0.1% TFA in *i*-PrOH = 90:5:5; *t*_R_ = 7.6 min (*S* enantiomer), 9.5 min (*R* enantiomer). The absolute configuration of the major product was assigned based on the starting (*S*)-1-phenylethanol.

#### 4.2.88 Methyl 4-Amino-3-(2,2,2-trifluoro-1-phenylethoxy)benzoate (**4s28**)

2,2,2-Trifluoro-1-phenylethanol (0.546 mL, 4.02 mmol) was dissolved in dry DMF (20 mL) on an ice bath and flushed with argon; then, NaH (60% suspension in mineral oil; 177 mg, 4.42 mmol) was added and the mixture was stirred on an ice bath for 30 min. Methyl 3-fluoro-4-nitrobenzoate (396 mg, 2.01 mmol) was added, and the reaction mixture was stirred at 22 °C overnight. The reaction was quenched with water (1 mL), and ethyl acetate (50 mL) was added. The organic phase was washed with water (30 mL), saturated aqueous NaHCO_3_ (30 mL), and brine (30 mL) and then dried over Na_2_SO_4_ and filtered. The solvent was removed in vacuo to obtain crude methyl 4-nitro-3-(2,2,2-trifluoro-1-phenylethoxy)benzoate (**3s28**) as a brown oil that was immediately transformed according to the general procedure B to give **4s28** as a pale yellow solid (360 mg, 55% yield over the two steps). ^1^H NMR (400 MHz, DMSO-*d*_6_): δ 7.72–7.62 (m, 2H), 7.47–7.41 (m, 3H), 7.39–7.35 (m, 2H), 6.69 (d, *J* = 8.7 Hz, 1H), 6.35 (q, *J* = 6.7 Hz, 1H), 5.76 (s, 2H), 3.69 (s, 3H); MS (ESI): *m*/*z* calcd for C_16_H_14_F_3_NO_3_: 325.09. Found: 357.7 [M + MeOH + H]^+^.

#### 4.2.89 Methyl 2-Amino-4-(2,2,2-trifluoro-1-phenylethoxy)benzo-[*d*]thiazole-6-carboxylate (**5s28**)

Prepared according to the general procedure C from aniline **4s28** (342 mg, 1.05 mmol); yellow solid (80 mg, 20% yield). ^1^H NMR (400 MHz, DMSO-*d*_6_): δ 8.09 (s, 2H), 7.97 (d, *J* = 1.5 Hz, 1H), 7.65–7.57 (m, 2H), 7.49–7.36 (m, 4H), 6.63 (q, *J* = 6.7 Hz, 1H), 3.77 (s, 3H).

#### 4.2.90 Methyl 2-(3,4-Dichloro-5-methyl-1*H*-pyrrole-2-carboxamido)-4-(2,2,2-trifluoro-1-phenylethoxy)benzo[*d*]thiazole-6-carboxylate (**6s28**)

Prepared according to the typical procedure D from 2-aminobenzothiazole **5s28** (80 mg, 0.209 mmol); white solid (80 mg, 69% yield). ^1^H NMR (400 MHz, DMSO-*d*_6_): δ 12.33 (s, 2H), 8.30 (d, *J* = 1.4 Hz, 1H), 7.65 (d, *J* = 7.0 Hz, 2H), 7.53 (d, *J* = 1.5 Hz, 1H), 7.51–7.38 (m, 3H), 6.74 (q, *J* = 6.7 Hz, 1H), 3.82 (s, 3H), 2.30 (s, 3H).

#### 4.2.91 2-(3,4-Dichloro-5-methyl-1*H*-pyrrole-2-carboxamido)-4-(2,2,2-trifluoro-1-phenylethoxy)benzo[*d*]thiazole-6-carboxylic Acid (**28**)

Prepared according to the typical procedure E from methyl ester **6s28** (60 mg, 0.108 mmol); brown solid (30 mg, 51% yield). ^1^H NMR (400 MHz, DMSO-*d*_6_): δ 12.97 (s, 1H), 12.48–12.12 (m, 2H), 8.25 (s, 1H), 7.73–7.59 (m, 2H), 7.57–7.35 (m, 4H), 6.70 (q, *J* = 6.7, 6.3 Hz, 1H), 2.29 (s, 3H); HRMS (ESI) *m*/*z*: [M + H]^+^ calcd for C_22_H_15_Cl_2_F_3_N_3_O_4_S 544.0112; found 544.0083; HPLC purity (254 nm): >99%.

#### 4.2.92 Methyl 3-(Benzocyclobutan-1-yloxy)-4-nitrobenzoate (**3s29**)

Prepared according to the typical procedure H from **2s** (909 mg, 4.61 mmol) and benzocyclobutan-1-ol (607 mg, 5.05 mmol); orange solid (1.27 g, 84% yield). ^1^H NMR (400 MHz, DMSO-*d*_6_): δ 8.06 (d, *J* = 8.4 Hz, 1H), 7.97 (d, *J* = 1.6 Hz, 1H), 7.74 (dd, *J* = 8.4, 1.6 Hz, 1H), 7.41 (td, *J* = 7.2, 1.5 Hz, 1H), 7.35–7.26 (m, 3H), 6.09 (dd, *J* = 4.3, 1.8 Hz, 1H), 3.94 (s, 3H), 3.79 (dd, *J* = 14, 4.3 Hz, 1H), 3.25 (d, *J* = 14.0 Hz, 1H).

#### 4.2.93 Methyl 4-Amino-3-(benzocyclobutan-1-yloxy)benzoate (**4s29**)

Prepared according to the typical procedure B from **3s29** (1.21 g, 4.04 mmol); white solid (500 mg, 46% yield). ^1^H NMR (400 MHz, DMSO-*d*_6_): δ 7.52 (d, *J* = 1.8 Hz, 1H), 7.43 (dd, *J* = 8.2, 1.8 Hz, 1H), 7.37 (app td, *J* = 7.3, 1.4 Hz, 1H), 7.30–7.23 (m, 4H), 6.67 (d, *J* = 8.2 Hz, 1H), 5.78–5.74 (m, 1H), 5.69 (s, 2H), 3.78 (s, 3H), 3.72 (dd, *J* = 14.2, 4.5 Hz, 1H), 3.30 (d, *J* = 14.2 Hz, 1H).

#### 4.2.94 Methyl 2-Amino-4-(benzocyclobutan-1-yloxy)benzo[*d*]thiazole-6-carboxylate (**5s29**)

Prepared according to the typical procedure C from aniline **4s29** (415 mg, 1.54 mmol); white powder (161 mg, 32% yield). ^1^H NMR (400 MHz, DMSO-*d*_6_): δ 8.01 (d, *J* = 1.4 Hz, 1H), 7.94 (s, 2H), 7.55 (d, *J* = 1.4 Hz, 1H), 7.38 (app td, *J* = 7.2, 1.5 Hz, 1H), 7.33–7.22 (m, 3H), 5.94 (dd, *J* = 4.3, 1.7 Hz, 1H), 3.86 (s, 3H), 3.74 (dd, *J* = 14.2, 4.3 Hz, 1H), 3.22 (d, *J* = 14.2 Hz, 1H).

#### 4.2.95 Methyl 4-(Benzocyclobutan-1-yloxy)-2-(3,4-dichloro-5-methyl-1*H*-pyrrole-2-carboxamido)benzo[*d*]thiazole-6-carboxylate (**6s29**)

Prepared according to the typical procedure D from 2-aminobenzothiazole **5s29** (150 mg, 0.459 mmol). Upon cooling, the precipitate was collected and washed with toluene and MeOH to get the crude product (47 mg), containing 20 mol % of *O*-dealkylated impurity (by ^1^H NMR). The evaporated toluene and MeOH filtrates contained no product, as established by ^1^H NMR. The crude product was dissolved in pyridine (0.5 mL) and treated with excess *tert*-butyldimethylsilyl chloride (TBDMSCl) (50 mg). After stirring at 22 °C for 3 h, the volatiles were removed in high vacuum (<10^–2^ mbar) and the solid residue was washed several times with hot toluene to remove most of the 4-TBDMSO impurity, yielding the title compound; gray solid (30 mg, 13% yield). ^1^H NMR (400 MHz, DMSO-*d*_6_) (characteristic signals): δ 8.35 (s, 1H), 7.71 (s, 1H), 6.06 (s, 1H), 3.92 (s, 3H), 2.26 (s, 3H).

#### 4.2.96 4-(Benzocyclobutan-1-yloxy)-2-(3,4-dichloro-5-methyl-1*H*-pyrrole-2-carboxamido)-benzo[*d*]thiazole-6-carboxylic Acid (**29**)

Prepared according to the typical procedure E from methyl ester **6s29** (30 mg, 0.060 mmol); brown powder (12 mg, 41% yield). ^1^H NMR (400 MHz, DMSO-*d*_6_): δ 13.0 (br s, 1H), 12.24 (s, 1H), 12.19 (s, 1H), 8.30 (s, 1H), 7.71 (s, 1H), 7.46–7.35 (m, 1H), 7.34–7.22 (m, 3H), 6.04 (d, *J* = 2.5 Hz, 1H), 3.82 (dd, *J* = 14.2, 4.1 Hz, 1H), 3.27 (d, *J* = 13.5 Hz, 1H), 2.26 (s, 3H). HRMS (ESI) *m*/*z*: [M + H]^+^ calcd for C_22_H_16_Cl_2_N_3_O_4_S 488. 0239; found 488.0226; HPLC purity (220 nm): 91%.

#### 4.2.97 Methyl 3-(2-(Benzoyloxy)-1-phenylethoxy)-4-nitrobenzoate (**3s30**)

Prepared according to the typical procedure H from **2s** (203 mg, 1.03 mmol) and 2-hydroxy-2-phenylethyl benzoate (259 mg, 1.13 mmol); white crystals (341 mg, 79% yield). ^1^H NMR (400 MHz, DMSO-*d*_6_): δ 7.97 (d, *J* = 8.4 Hz, 1H), 7.91–7.85 (m, 1H), 7.82 (d, *J* = 1.5 Hz, 1H), 7.69–7.59 (m, 2H), 7.60–7.53 (m, 2H), 7.53–7.48 (m, 2H), 7.48–7.40 (m, 2H), 7.40–7.32 (m, 1H), 6.29–6.18 (m, 1H), 4.69–4.59 (m, 2H), 3.84 (s, 3H).

#### 4.2.98 Methyl 4-Amino-3-(2-(benzoyloxy)-1-phenylethoxy)benzoate (**4s30**)

Prepared according to the typical procedure B from **3s30** (649 mg, 1.54 mmol); yellow oil (414 mg, 69% yield). ^1^H NMR (400 MHz, DMSO-*d*_6_): δ 7.95–7.91 (m, 2H), 7.70–7.61 (m, 1H), 7.62–7.56 (m, 2H), 7.54–7.48 (m, 2H), 7.44–7.38 (m, 2H), 7.36–7.31 (m, 2H), 7.29 (d, *J* = 1.8 Hz, 1H), 6.64 (d, *J* = 8.3 Hz, 1H), 5.78 (t, *J* = 5.3 Hz, 1H), 4.72 (dd, *J* = 11.5, 7.6 Hz, 1H), 4.56 (dd, *J* = 11.5, 3.6 Hz, 1H), 3.68 (s, 3H); MS (ESI): *m*/*z* calcd for C_23_H_21_NO_5_: 391.14. Found: 392.2 [M + H]^+^.

#### 4.2.99 Methyl 2-Amino-4-(2-(benzoyloxy)-1-phenylethoxy)benzo-[*d*]thiazole-6-carboxylate (**5s30**)

Prepared according to the typical procedure C from aniline **4s30** (344 mg, 0.879 mmol); light yellow solid (126 mg, 32% yield). ^1^H NMR (400 MHz, DMSO-*d*_6_): δ 7.96 (s, 2H), 7.91 (d, *J* = 1.4 Hz, 1H), 7.89–7.84 (m, 2H), 7.64 (t, *J* = 7.4 Hz, 1H), 7.57 (d, *J* = 7.3 Hz, 2H), 7.49 (t, *J* = 7.7 Hz, 2H), 7.43–7.36 (m, 3H), 7.35–7.28 (m, 1H), 6.25–6.03 (m, 1H), 4.74–4.47 (m, 2H), 3.76 (s, 3H); MS (ESI): *m*/*z* calcd for C_24_H_20_N_2_O_5_S: 448.11. Found: 449.2 [M + H]^+^.

#### 4.2.100 Methyl 4-(2-(Benzoyloxy)-1-phenylethoxy)-2-(3,4-dichloro-5-methyl-1*H*-pyrrole-2-carboxamido)benzo[*d*]thiazole-6-carboxylate (**6s30**)

Prepared according to the typical procedure D from **5s30** (87 mg, 0.194 mmol); gray solid (96 mg, 79% yield). ^1^H NMR (400 MHz, DMSO-d_6_): δ = 12.29 (s, 1H), 12.25 (s, 1H), 8.23 (s, 1H), 7.92–7.86 (m, 2H), 7.68–7.58 (m, 3H), 7.55–7.46 (m, 3H), 7.46–7.39 (m, 2H), 7.39–7.31 (m, 1H), 6.23–6.14 (m, 1H), 4.79–4.62 (m, 2H), 3.82 (s, 3H), 2.28 (s, 3H); MS (ESI): *m*/*z* calcd for C_30_H_23_Cl_2_N_3_O_6_S: 623.07. Found: 424.5 [M + H]^+^.

#### 4.2.101 2-(3,4-Dichloro-5-methyl-1*H*-pyrrole-2-carboxamido)-4-(2-hydroxy-1-phenylethoxy)benzo[*d*]thiazole-6-carboxylic Acid (**30**)

Prepared according to the typical procedure E from ester **6s30** (82 mg, 0.131 mmol); brown solid (51 mg, 77% yield). ^1^H NMR (400 MHz, DMSO-*d*_6_): δ 12.85 (s, 1H), 12.27 (s, 2H), 8.14 (s, 1H), 7.53–7.20 (m, 6H), 5.66–5.53 (m, 1H), 5.11 (s, 1H), 3.93–3.80 (m, 1H), 3.79–3.68 (m, 1H), 2.29 (s, 3H); ^13^C NMR (101 MHz, DMSO-*d*_6_): δ 166.86, 159.63, 156.80, 149.28, 141.62, 138.51, 132.72, 129.93, 128.48, 127.85, 126.51, 126.45, 117.15, 116.06, 115.64, 110.34, 109.96, 81.07, 65.88, 11.08. HRMS (ESI) *m*/*z*: [M + H]^+^ calcd for C_22_H_18_Cl_2_N_3_O_5_S 506.0344; found 506.0337; HPLC purity (254 nm): 93%.

#### 4.2.102 Methyl 3-(2-Methoxy-1-phenylethoxy)-4-nitrobenzoate (**30s31**)

Prepared according to the typical procedure H from **2s** (2.00 g, 10.14 mmol) and 2-methoxy-1-phenylethanol (1.70 g, 11.15 mmol); white crystals (2.85 g, 85% yield). ^1^H NMR (400 MHz, DMSO-*d*_6_): δ 8.00–7.93 (m, 1H), 7.75 (d, *J* = 1.5 Hz, 1H), 7.60 (dd, *J* = 8.3, 1.5 Hz, 1H), 7.49–7.44 (m, 2H), 7.42–7.36 (m, 2H), 7.35–7.29 (m, 1H), 5.94 (dd, *J* = 7.5, 3.3 Hz, 1H), 3.84 (s, 3H), 3.73 (dd, *J* = 11.3, 7.6 Hz, 1H), 3.61 (dd, *J* = 11.3, 3.4 Hz, 1H), 3.32 (s, 3H).

#### 4.2.103 Methyl 4-Amino-3-(2-methoxy-1-phenylethoxy)benzoate (**31s31**)

Prepared according to the typical procedure B from **30s31** (2.56 g, 7.74 mmol); white solid (2.24 g, 96% yield). ^1^H NMR (400 MHz, DMSO-*d*_6_) δ 7.50–7.44 (m, 2H), 7.39–7.25 (m, 4H), 7.19 (d, *J* = 1.8 Hz, 1H), 6.64 (d, *J* = 8.3 Hz, 1H), 5.78 (s, 2H), 5.42 (dd, *J* = 7.8, 3.5 Hz, 1H), 3.79 (dd, *J* = 10.8, 7.9 Hz, 1H), 3.67 (s, 3H), 3.55 (dd, *J* = 10.8, 3.5 Hz, 1H), 3.36–3.33 (m, 3H); MS (ESI): *m*/*z* calcd for C_17_H_19_NO_4_: 301.13. Found: 324.0 [M + Na]^+^.

#### 4.2.104 Methyl 2-Amino-4-(2-methoxy-1-phenylethoxy)benzo[*d*]-thiazole-6-carboxylate (**32s31**)

Prepared according to the general procedure C from aniline **31s31** (2.25 g, 7.48 mmol); light yellow solid (456 mg, 17% yield). ^1^H NMR (400 MHz, DMSO-*d*_6_): δ 7.97 (s, 2H), 7.87 (d, *J* = 1.5 Hz, 1H), 7.49–7.40 (m, 2H), 7.39–7.30 (m, 2H), 7.30–7.22 (m, 2H), 5.78 (dd, *J* = 7.3, 3.8 Hz, 1H), 3.80–3.70 (m, 4H), 3.60 (dd, *J* = 10.8, 3.8 Hz, 1H), 3.33 (s, 3H); MS (ESI): *m*/*z* calcd for C_18_H_18_N_2_O_4_S: 358.09. Found: 359.0 [M + H]^+^.

#### 4.2.105 Methyl 2-(3,4-Dichloro-5-methyl-1*H*-pyrrole-2-carboxamido)-4-(2-methoxy-1-phenylethoxy)benzo[*d*]thiazole-6-carboxylate (**33s31**)

Prepared according to the typical procedure D from **32s31** (200 mg, 0.557 mmol); gray solid (247 mg, 83% yield). ^1^H NMR (400 MHz, DMSO-*d*_6_): δ 12.35 (s, 1H), 12.28 (s, 1H), 8.19 (s, 1H), 7.54–7.44 (m, 2H), 7.43–7.32 (m, 3H), 7.32–7.26 (m, 1H), 5.86 (dd, J = 7.5, 3.4 Hz, 1H), 3.87–3.74 (m, 4H), 3.65 (dd, J = 10.8, 3.4 Hz, 1H), 2.29 (s, 3H); MS (ESI): *m*/*z* calcd for C_24_H_21_Cl_2_N_3_O_5_S: 533.06. Found: 555.9 [M + Na]^+^.

#### 4.2.106 2-(3,4-Dichloro-5-methyl-1*H*-pyrrole-2-carboxamido)-4-(2-methoxy-1-phenylethoxy)benzo[*d*]thiazole-6-carboxylic Acid (**31**)

Prepared according to the typical procedure E from methyl ester **33s31** (113 mg, 0.211 mmol); off-white solid (78 mg, 71% yield). ^1^H NMR (400 MHz, DMSO-*d*_6_): δ 12.88 (s, 1H), 12.32 (s, 1H), 12.28 (s, 1H), 8.15 (s, 1H), 7.53–7.46 (m, 2H), 7.41–7.32 (m, 3H), 7.28 (ddd, *J* = 8.5, 2.3, 1.2 Hz, 1H), 5.84 (dd, *J* = 7.6, 3.5 Hz, 1H), 3.81 (dd, *J* = 10.8, 7.6 Hz, 1H), 3.64 (dd, *J* = 10.8, 3.5 Hz, 1H), 2.29 (s, 3H); ^13^C NMR (101 MHz, DMSO-*d*_6_): δ 166.84, 159.65, 156.63, 149.03, 141.99, 138.01, 132.86, 129.92, 128.55, 128.02, 126.50, 126.43, 116.90, 116.13, 115.74, 78.20, 76.00, 58.37, 11.08; HRMS (ESI) *m*/*z*: [M – H]^−^ calcd for C_23_H_20_Cl_2_N_3_O_5_S 520.0501; found 520.0497; HPLC purity (254 nm): 98%.

#### 4.2.107 *tert*-Butyl 3-(2-(Methylsulfonyl)-1-phenylethoxy)-4-nitro-benzoate (**30s32**)

Prepared according to the typical procedure H from *tert*-butyl 3-hydroxy-4-nitrobenzoate (2.62 g, 10.96 mmol) and 2-mesyl-1-phenylethanol (2.41 g, 12.06 mmol); white solid (877 mg, 19% yield). ^1^H NMR (400 MHz, CDCl_3_): δ 7.81 (d, *J* = 8.8 Hz, 1H), 7.64–7.58 (m, 2H), 7.45–7.32 (m, 5H), 6.02 (dd, *J* = 10.2, 2.2 Hz, 1H), 3.89 (dd, *J* = 15.5, 10.2 Hz, 1H), 3.30 (ddq, J = 15.5, 2.1, 1 Hz, 1H), 3.12 (d, J = 1 Hz, 3H), 1.54 (s, 9H); MS (ESI): *m*/*z* calcd for C_20_H_23_NO_7_S: 421.12. Found: 485.1 [M + Na + CH_3_CN]^+^.

#### 4.2.108 *tert*-Butyl 3-(2-(Methylsulfonyl)-1-phenylethoxy)-4-amino-benzoate (**31s32**)

Prepared according to the typical procedure B from **30s32** (847 mg, 2.01 mmol); white solid (472 mg, 60% yield). ^1^H NMR (400 MHz, DMSO-*d*_6_) δ 7.54–7.48 (m, 2H), 7.43–7.37 (m, 2H), 7.36–7.30 (m, 1H), 7.25 (dd, *J* = 8.3, 1.8 Hz, 1H), 7.08 (d, *J* = 1.7 Hz, 1H), 5.80 (s, 2H), 5.69 (dd, *J* = 10.1, 2.2 Hz, 1H), 4.07 (dd, *J* = 15.1, 10.6 Hz, 1H), 3.52 (d, *J* = 14 Hz, 1H), 3.10 (s, 3H), 1.43 (s, 9H); MS (ESI): *m*/*z* calcd for C_20_H_25_NO_5_S: 391.14. Found: 392.2 [M + H]^+^.

#### 4.2.109 *tert*-Butyl 2-Amino-4-(2-(methylsulfonyl)-1-phenylethoxy)-benzo[*d*]thiazole-6-carboxylate (**32s32**)

Prepared according to the typical procedure C from aniline **31s32** (429 mg, 1.10 mmol); light yellow solid (359 mg, 73% yield). ^1^H NMR (400 MHz, DMSO-*d*_6_): δ 7.88 (s, 2H), 7.82 (d, *J* = 1.5 Hz, 1H), 7.56–7.52 (m, 2H), 7.43–7.37 (m, 2H), 7.35–7.30 (m, 1H), 7.17 (d, *J* = 1.4 Hz, 1H), 5.85 (dd, *J* = 10.4, 2.7 Hz, 1H), 4.12 (dd, *J* = 15.4, 10.3 Hz, 1H), 3.52–3.44 (m, 1H), 3.32 (s, 3H), 1.47 (s, 9H); MS (ESI): *m*/*z* calcd for C_21_H_24_N_2_O_5_S_2_: 448.11. Found: 449.2 [M + H]^+^.

#### 4.2.110 *tert*-Butyl 2-(3,4-Dichloro-5-methyl-1*H*-pyrrole-2-carboxamido)-4-(2-(methylsulfonyl)-1-phenylethoxy)benzo[*d*]thiazole-6-carboxylate (**33s32**)

Prepared according to the typical procedure D from 2-aminobenzothiazole **32s32** (320 mg, 713 mmol); violet powder (147 mg, 33% yield). ^1^H NMR (400 MHz, DMSO-*d*_6_): δ 12.41 (s, 1H), 12.11 (s, 1H), 8.16 (s, 1H), 7.58 (d, J = 7.2 Hz, 2H), 7.45–7.31 (m, 4H), 6.01 (dd, *J* = 10.3, 2.3 Hz, 1H), 4.14 (dd, *J* = 15.1, 10.2 Hz, 2H), 3.65–3.58 (m, 1H), 3.29 (s, 3H), 2.29 (s, 3H), 1.51 (s, 9H); MS (ESI): *m*/*z* calcd for C_27_H_27_Cl_2_N_3_O_6_S_2_: 623.07. Found: 624.1 [M + H]^+^

#### 4.2.111 2-(3,4-Dichloro-5-methyl-1*H*-pyrrole-2-carboxamido)-4-(3-(methylsulfonyl)-2-phenylpropyl)benzo[*d*]thiazole-6-carboxylic Acid (**32**)

Trifluoroacetic acid (0.04 mL, 0.48 mmol) was added to a suspension of *tert*-butyl ester **33s32** (30 mg, 0.048 mmol) in dichloromethane (2 mL). The reaction mixture was stirred at 22 °C overnight, the solvent was removed under reduced pressure, and the residue was triturated with MeOH to give the product as a dark violet solid (15 mg, 56% yield). ^1^H NMR (400 MHz, DMSO-*d*_6_): δ 12.95 (s, 1H), 12.37 (s, 1H), 12.13 (s, 1H), 8.21 (s, 1H), 7.56 (app d, *J* = 7.3 Hz, 2H), 7.43–7.37 (m, 3H), 7.35–7.30 (m, 1H), 6.08 (dd, *J* = 10, 2.7 Hz, 1H), 4.10 (dd, *J* = 15.0, 10 Hz, 1H), 3.64 (d, *J* = 13 Hz, 1H), 3.27 (s, 3H), 2.29 (s, 3H); ^13^C NMR (101 MHz, DMSO-*d*_6_): δ (characteristic signals) 167.18, 138.47, 130.54, 129.39, 129.03, 127.01, 126.73, 117.31, 110.80, 110.43, 75.47, 60.70, 43.59, 11.54; HRMS (ESI) *m*/*z*: [M + H]^+^ calcd for C_23_H_20_Cl_2_N_3_O_6_S_2_ 568.0171; found 568.0166; HPLC purity (254 nm): 97%.

#### 4.2.112 2-((6-Carboxy-2-(3,4-dichloro-5-methyl-1*H*-pyrrole-2-carboxamido)benzo[*d*]thiazol-4-yl)oxy)-2-phenylethan-1-aminium Chloride (**33**)

The Boc-protected amine **39** (53 mg, 0.088 mmol) was suspended in 4 M HCl/dioxane (4 mL) and stirred at 22 °C for 24 h. The precipitate was filtered off and washed with dioxane to obtain the title compound as a violet-tinted solid (13 mg, 46% yield). ^1^H NMR (400 MHz, DMSO-*d*_6_): δ 13.06 (s, 1H), 12.64 (s, 1H), 12.30 (s, 1H), 8.39–8.20 (m, 4H), 7.54–7.49 (m, 2H), 7.48–7.39 (m, 3H), 7.35 (t, *J* = 7.2 Hz, 1H), 5.90 (t, *J* = 5.8 Hz, 1H), 2.30 (s, 3H) (CH_2_–NH_2_ signals overlayed by the water peak); ^13^C NMR (101 MHz, DMSO-*d*_6_): δ (characteristic signals) 166.62, 137.10, 132.92, 130.05, 128.89, 128.80, 126.47, 126.42, 118.13, 116.09, 114.08, 110.12, 78.72, 66.34, 44.89, 11.03; HRMS (ESI) *m*/*z*: [M – Cl]^+^ calcd for C_22_H_19_Cl_2_N_4_O_4_S 505.0499; found 505.0491; HPLC purity (254 nm): 95%.

#### 4.2.113 Methyl 3-(2-(Dimethylamino)-1-phenylethoxy)-4-nitrobenzoate (**30s34**)

Prepared according to the typical procedure H from **2s** (2.00 g, 10.14 mmol) and 2-(dimethylamino)-1-phenylethan-1-ol (1.84 g, 11.15 mmol); thick oil (1.84 g, 53% yield). ^1^H NMR (400 MHz, DMSO-*d*_6_): δ 7.94 (d, *J* = 8.3 Hz, 1H), 7.75 (d, *J* = 1.5 Hz, 1H), 7.58 (dd, *J* = 8.3, 1.5 Hz, 1H), 7.46–7.41 (m, 2H), 7.40–7.33 (m, 2H), 7.32–7.25 (m, 1H), 5.83 (dd, *J* = 7.6, 4.1 Hz, 1H), 3.83 (s, 3H), 2.23 (s, 6H); MS (ESI): *m*/*z* calcd for C_18_H_20_N_2_O_5_: 344.14. Found: 345.4 [M + H]^+^.

#### 4.2.114 Methyl 4-Amino-3-(2-(dimethylamino)-1-phenylethoxy)-benzoate (**31s34**)

Prepared according to the typical procedure B from **30s34** (1.84 g, 5.34 mmol); white powder (965 mg, 57% yield). ^1^H NMR (400 MHz, DMSO-*d*_6_): δ 7.52–7.36 (m, 4H), 7.36–7.27 (m, 2H), 7.17 (d, *J* = 1.4 Hz, 1H), 6.61 (d, *J* = 8.3 Hz, 1H), 6.16 (s, 2H), 5.92 (dd, J = 10.0, 1 Hz, 1H), 3.71–3.58 (m, 4H), 3.39 (dd, *J* = 13.3, 1 Hz, 1H), 2.88 (s, 6H); MS (ESI): *m*/*z* calcd for C_18_H_22_N_2_O_3_: 314.16. Found: 315.5 [M + H]^+^.

#### 4.2.115 Methyl 2-Amino-4-(2-(dimethylamino)-1-phenylethoxy)-benzo[*d*]thiazole-6-carboxylate (**32s34**)

Prepared according to the typical procedure C from aniline **31s34** (940 mg, 2.99 mmol); off-white solid (585 mg, 53% yield). ^1^H NMR (400 MHz, DMSO-*d*_6_): δ 8.10 (s, 2H), 8.01 (d, *J* = 1.5 Hz, 1H), 7.51–7.31 (m, 5H), 7.25 (d, *J* = 1.5 Hz, 1H), 6.06 (dd, *J* = 10.8, 1.0 Hz, 1H), 3.83–3.71 (m, 4H), 3.48–3.40 (m, 1H), 3.04 (s, 6H); MS (ESI): *m*/*z* calcd for C_19_H_21_N_3_O_3_S: 371.13. Found: 372.2 [M + H]^+^.

#### 4.2.116 Methyl 2-(3,4-Dichloro-5-methyl-1*H*-pyrrole-2-carboxamido)-4-(2-(dimethylamino)-1-phenylethoxy)benzo[*d*]thiazole-6-carboxylate (**33s34**)

Prepared according to the typical procedure J from 2-aminobenzothiazole **32s34** (200 mg, 0.538 mmol); gray powder (85 mg, 29% yield). ^1^H NMR (400 MHz, DMSO-*d*_6_): δ 12.20 (s, 2H), 8.22 (s, 1H), 7.66–7.29 (m, 6H), 6.18–5.78 (m, 1H), 3.81 (s, 3H), 2.90 (s, 6H), 2.28 (s, 3H) (the peaks of CH_2_N are overlapped with DMSO and water signal); MS (ESI): *m*/*z* calcd for C_25_H_24_Cl_2_N_4_O_4_S: 546.09. Found: 547.4 [M + H]^+^; HPLC purity (254 nm): 98%.

#### 4.2.117 2-(3,4-Dichloro-5-methyl-1*H*-pyrrole-2-carboxamido)-4-(2-(dimethylamino)-1-phenylethoxy)benzo[*d*]thiazole-6-carboxylic Acid (**34**)

Prepared according to the typical procedure E from methyl ester **33s34** (80 mg, 0.147 mmol); brown powder (43 mg, 55%). ^1^H NMR (400 MHz, DMSO-*d*_6_): δ 13.08–11.69 (m, 3H), 8.11 (s, 1H), 7.60–7.23 (m, 6H), 5.79–5.54 (m, 1H), 3.46–2.70 (m, 8H), 2.25 (s, 3H); HRMS (ESI) *m*/*z*: [M + H]^+^ calcd for C_24_H_23_Cl_2_N_4_O_4_S 533.0817; found 533.0802; HPLC purity (254 nm): 98%.

#### 4.2.118 Methyl 4-Nitro-3-(1-phenyl-2-(piperidin-1-yl)ethoxy)benzoate (**30s35**)

Prepared according to the typical procedure H from **2s** (1.05 g, 5.35 mmol) and 1-phenyl-2-(piperidin-1-yl)ethanol (1.21 g, 5.88 mmol); yellow oil (1.83 g, 89% yield). ^1^H NMR (400 MHz, CDCl_3_): δ 7.76 (d, *J* = 8.4 Hz, 1H), 7.70 (d, *J* = 1.5 Hz, 1H), 7.59 (dd, *J* = 8.4, 1.5 Hz, 1H), 7.41 (m, 2H), 7.37–7.27 (m, 3H), 5.56 (dd, *J* = 8.1, 3.0 Hz, 1H), 3.89 (s, 3H), 3.02 (dd, *J* = 14.2, 8.1 Hz, 1H), 2.69 (dd, *J* = 14.2, 3.1 Hz, 1H), 2.59–2.44 (m, 4H), 1.51 (m, 4H), 1.39 (m, 2H); MS (ESI): *m*/*z* calcd for C_21_H_24_N_2_O_5_: 384.17. Found: 385.2 [M + H]^+^.

#### 4.2.119 Methyl 4-Amino-3-(1-phenyl-2-(piperidin-1-yl)ethoxy)benzoate (**31s35**)

Prepared according to the typical procedure B from **30s35** (1.61 g, 4.18 mmol); light yellow oil (1.29 g, 87% yield). ^1^H NMR (400 MHz, DMSO-*d*_6_): δ 7.47–7.41 (m, 2H), 7.41–7.27 (m, 4H), 7.06 (d, *J* = 1.9 Hz, 1H), 6.64 (d, *J* = 8.3 Hz, 1H), 6.36 (s, 2H), 5.01 (dd, *J* = 9.2, 2.2 Hz, 1H), 3.64 (s, 3H), 2.91 (dd, *J* = 13.6, 9.3 Hz, 1H), 2.64–2.56 (m, 2H), 2.49–2.42 (m, 2H), 2.36 (dd, *J* = 13.6, 2.5 Hz, 1H), 1.58–1.46 (m, 4H), 1.40 (d, *J* = 4.4 Hz, 2H); MS (ESI): *m*/*z* calcd for C_21_H_26_N_2_O_3_: 354.19. Found: 355.3 [M + H]^+^.

#### 4.2.120 Methyl 2-Amino-4-(1-phenyl-2-(piperidin-1-yl)ethoxy)benzo[*d*]thiazole-6-carboxylate (**32s35**)

Prepared according to the typical procedure C from aniline **31s35** (613 mg, 1.73 mmol); light yellow solid (534 mg, 75% yield). ^1^H NMR (400 MHz, DMSO-*d*_6_): δ 8.00 (s, 2H), 7.98 (d, *J* = 0.8 Hz, 1H), 7.49–7.44 (m, 2H), 7.40 (app t, *J* = 7.3 Hz, 2H), 7.34 (t, *J* = 7.1 Hz, 1H), 7.27 (d, *J* = 1.5 Hz, 1H), 6.24–6.02 (m, 1H), 4.05–3.90 (m, 1H), 3.76 (s, 3H), 3.77–3.63 (m, 2H), 3.21–3.03 (m, 2H), 1.99–1.62 (m, 5H), 1.53–1.34 (m, 1H); MS (ESI): *m*/*z* calcd for C_22_H_25_N_3_O_3_S: 411.16. Found: 412.2 [M + H]^+^.

#### 4.2.121 Methyl 2-(3,4-Dichloro-5-methyl-1*H*-pyrrole-2-carboxamido)-4-(1-phenyl-2-(piperidin-1-yl)ethoxy)benzo[*d*]thiazole-6-carboxylate Monocitrate Salt (**33s35**)

Prepared according to the typical procedure J from 2-aminobenzothiazole **32s35** (108 mg, 0.261 mmol); dark violet solid (62 mg, 31% yield). ^1^H NMR (characteristic signals) (400 MHz, DMSO-*d*_6_): δ 12.45 (s, 1H), 11.94 (s, 1H), 9.49 (s, 1H), 8.28 (s, 1H), 7.52 (d, *J* = 7.3 Hz, 2H), 7.48 (s, 1H), 7.41 (t, *J* = 7.4 Hz, 2H), 7.34 (t, *J* = 7.3 Hz, 1H), 6.30 (d, *J* = 8.7 Hz, 1H), 3.89–3.76 (m, 5H), 3.70 (dd, *J* = 15.4, 8.0 Hz, 1H), 3.61–3.50 (m, 1H), 3.23–3.06 (m, 2H), 2.29 (s, 3H), 1.95–1.83 (m, 2H), 1.80–1.64 (m, 3H), 1.51–1.34 (m, 1H); MS (ESI): *m*/*z* calcd for C_28_H_28_Cl_2_N_4_O_4_S: 586.12. Found: 587.4 [M + H]^+^.

#### 4.2.122 2-(3,4-Dichloro-5-methyl-1*H*-pyrrole-2-carboxamido)-4-(1-phenyl-2-(piperidin-1-yl)ethoxy)benzo[*d*]thiazole-6-carboxylate Sodium Salt (**35**)

To a suspension of methyl ester **33s35** (77 mg, 0.100 mmol) in methanol (2 mL), 1 M NaOH (0.400 mL, 0,400 mmol) was added and the reaction mixture was stirred at 40 °C overnight. An additional 1 M of NaOH (0.400 mL, 0.400 mmol) was added and stirred at 40 °C for a further 24 h. The solvent was removed, and the residue was purified by reverse phase column chromatography, eluent 20–80% acetonitrile in water; brown solid (12.5 mg, 21% yield). ^1^H NMR (400 MHz, DMSO-*d*_6_): δ 7.68 (d, *J* = 1.3 Hz, 1H), 7.47–7.39 (m, 2H), 7.32–7.24 (m, 3H), 7.23–7.14 (m, 1H), 5.76 (dd, *J* = 7.5, 4.2 Hz, 1H), 2.92 (dd, *J* = 13.4, 7.5 Hz, 1H), 2.59 (dd, *J* = 13.4, 4 Hz, 1H), 2.18 (s, 3H), 1.53–1.41 (m, 4H), 1.39–1.30 (m, 2H) (peaks of pyrrole and amide NH are not observed, and peaks of four piperidine protons are covered by the DMSO signal); HRMS (ESI) *m*/*z*: [M–Na + 2H]^+^ calcd for C_27_H_27_Cl_2_N_4_O_4_S 573.1130; found: 573.1112; HPLC purity (254 nm): 94%.

#### 4.2.123 Methyl 3-(2-Morpholino-1-phenylethoxy)-4-nitrobenzoate (**30s36**)

Prepared according to the typical procedure H from **2s** (2.51 g, 12.73 mmol) and 2-morpholino-1-phenylethanol (2.90 g, 14.00 mmol); yellow solid (4.77 g, 97%). ^1^H NMR (400 MHz, CDCl_3_) δ 7.77 (d, *J* = 8.4 Hz, 1H), 7.68 (d, *J* = 1.5 Hz, 1H), 7.60 (dd, *J* = 8.4, 1.5 Hz, 1H), 7.44–7.39 (m, 2H), 7.38–7.33 (m, 2H), 7.32–7.27 (m, 1H), 5.55 (dd, *J* = 8.1, 3.2 Hz, 1H), 3.88 (s, 3H), 3.64 (t, *J* = 4.7 Hz, 4H), 3.04 (dd, *J* = 14.1, 8.1 Hz, 1H), 2.73 (dd, *J* = 14.1, 3.2 Hz, 1H), 2.66–2.50 (m, 4H); MS (ESI): *m*/*z* calcd for C_20_H_22_N_2_O_6_: 386.15. Found: 387.2 [M + H]^+^.

#### 4.2.124 Methyl 4-Amino-3-(2-morpholino-1-phenylethoxy)benzoate (**31s36**)

Prepared according to the typical procedure B from **30s36 (**4.68 g, 12.12 mmol); white solid (4.32 g, 100%). ^1^H NMR (400 MHz, DMSO-*d*_6_) δ 7.45 (app d, *J* = 7.1 Hz, 2H), 7.40–7.33 (m, 3H), 7.29 (t, *J* = 7.2 Hz, 1H), 7.12 (d, *J* = 1.7 Hz, 1H), 6.64 (d, *J* = 8.3 Hz, 1H), 6.15 (s, 2H), 5.18 (dd, *J* = 8.9, 2.6 Hz, 1H), 3.65 (s, 3H), 3.58 (t, *J* = 4.5 Hz, 4H), 2.97 (dd, *J* = 13.5, 9.0 Hz, 1H), 2.66–2.51 (m, 4H), 2.47 (dd, *J* = 13.7, 2.9 Hz, 1H); MS (ESI): *m*/*z* calcd for C_20_H_24_N_2_O_4_: 356.17. Found: 357.1 [M + H]^+^.

#### 4.2.125 Methyl 2-Amino-4-(2-morpholino-1-phenylethoxy)benzo-[*d*]thiazole-6-carboxylate (**32s36**)

Prepared according to the typical procedure C from aniline **31s36** (1.11 g, 3.13 mmol); light yellow solid (556 mg, 43% yield). ^1^H NMR (400 MHz, DMSO): δ 7.93 (s, 2H), 7.86 (d, *J* = 1.5 Hz, 1H), 7.47–7.40 (m, 2H), 7.35–7.29 (m, 3H), 7.27–7.18 (m, 1H), 5.80 (dd, *J* = 7.8, 3.9 Hz, 1H), 3.76 (s, 3H), 3.52 (app t, *J* = 4.6 Hz, 4H), 2.92 (dd, *J* = 13.4, 7.9 Hz, 1H), 2.62 (dd, *J* = 13.5, 4.0 Hz, 1H), 2.59–2.51 (m, 4H); ^19^F NMR (376 MHz, DMSO-*d*_6_): δ −67.92 (d, *J* = 9.0 Hz); ^13^C NMR (101 MHz, DMSO-*d*_6_): δ 168.76, 166.01, 147.43, 146.93, 132.26, 126.15 (q, *J* = 288.0 Hz), 122.24, 116.58, 111.44, 66.79, 64.56, 63.75 (q, *J* = 25.0 Hz), 51.98, 50.15; MS (ESI): *m*/*z* calcd for C_21_H_23_N_3_O_4_S: 413.14. Found: 414.2 [M + H]^+^.

#### 4.2.126 Methyl 2-(3,4-Dichloro-5-methyl-1*H*-pyrrole-2-carboxamido)-4-(2-morpholino-1-phenylethoxy)benzo[*d*]-thiazole-6-carboxylate (**33s36**)

Prepared according to the typical procedure D from 2-aminobenzothiazole **32s36** (250 mg, 0.605 mmol); light pink solid (150 mg, 42% yield). ^1^H NMR (400 MHz, DMSO-*d*_6_): δ 12.27 (s, 2H), 8.18 (s, 1H), 7.51–7.46 (m, 3H), 7.35 (app t, *J* = 7.5 Hz, 2H), 7.26 (t, *J* = 7.4 Hz, 1H), 5.86 (dd, *J* = 7.9, 3.8 Hz, 1H), 3.81 (s, 3H), 3.58–3.51 (m, 4H), 3.07–2.95 (m, 1H), 2.65–2.54 (m, 4H), 2.54–2.51 (m, 1H), 2.29 (s, 3H); MS (ESI): *m*/*z* calcd for C_27_H_26_Cl_2_N_4_O_5_S: 588.10. Found: 589.3 [M + H]^+^.

#### 4.2.127 2-(3,4-Dichloro-5-methyl-1*H*-pyrrole-2-carboxamido)-4-(2-morpholino-1-phenylethoxy)benzo[*d*]thiazole-6-carboxylic Acid (**36**)

Prepared according to the typical procedure E from methyl ester **33s36** (75 mg, 0.127 mmol); light pink solid (60 mg, 82% yield). ^1^H NMR (400 MHz, DMSO-*d*_6_): δ 13.37–12.65 (m, 2H), 12.32 (s, 1H), 10.70 (s, 1H), 8.24 (s, 1H), 7.55–7.47 (m, 2H), 7.46–7.37 (m, 3H), 7.39–7.30 (m, 1H), 6.40 (d, *J* = 10.0 Hz, 1H), 4.14–4.02 (m, 2H), 3.95–3.74 (m, 5H), 3.68–3.55 (m, 1H), 2.30 (s, 3H) (2H overlapped with the water peak); ^13^C NMR (101 MHz, DMSO-*d*_6_): δ (representative signals) 166.62, 136.71, 130.10, 128.98, 126.52, 126.42, 117.73, 110.04, 75.72, 63.27, 60.91, 53.10, 52.01, 10.98; HRMS (ESI) *m*/*z*: [M + H]^+^ calcd for C_26_H_25_Cl_2_N_4_O_5_S 575.0923; found 575.0917; HPLC purity (254 nm): 97%.

#### 4.2.128 2-((6-Carboxy-2-(3,4-dichloro-5-methyl-1*H*-pyrrole-2-carboxamido)benzo[*d*]thiazol-4-yl)oxy)-*N*,*N*,*N*-trimethyl-2-phenylethan-1-aminium Iodide (**37**)

Prepared according to the general procedure G from the tertiary amine 34 (10 mg, 0.019 mmol); gray solid (4.7 mg, 37%) yield. ^1^H NMR (400 MHz, CD_3_OD): δ 8.22 (s, 1H), 7.58–7.50 (m, 2H), 7.49 (s, 1H), 7.45–7.32 (m, 3H), 6.38 (d, *J* = 10 Hz, 1H), 4.25 (dd, *J* = 14, 10 Hz, 1H), 3.75 (d, *J* = 14 Hz, 1H), 3.50 (s, 9H), 2.33 (s, 3H); HRMS (ESI) *m*/*z*: [M – I]^+^ calcd for C_25_H_25_Cl_2_N_4_O_4_S 547.0968; found 547.0960; HPLC purity (254 nm): 97%.

#### 4.2.129 Methyl 3-(2-Amino-1-phenylethoxy)-4-nitrobenzoate (**30s33**)

Boc-protected derivative **30s39** (1.09 g, 2.62 mmol) was dissolved in 4 M HCl/dioxane (9 mL, 36 mmol) and stirred at 22 °C for 45 min. The volatiles were evaporated, and the residue was neutralized with saturated aqueous NaHCO_3_ solution. After extraction with EtOAc, the organic layer was washed with brine, dried over Na_2_SO_4_, filtered, and concentrated to get 800 mg (97% yield) of a 60:40 mixture of **30s30** (major isomer) and methyl 3-((2-hydroxy-2-phenylethyl)amino)-4-nitrobenzoate (rearranged isomer), which was used without separation in the next step. Major isomer **30s33**: ^1^H NMR (400 MHz, DMSO-*d*_6_): δ 7.98 (d, *J* = 8.3 Hz, 1H), 7.64 (s, 1H), 7.60 (d, *J* = 8.3 Hz, 1H), 7.49–7.25 (m, 5H), 5.56 (dd, *J* = 7.1 Hz, 4.0 Hz, 1H), 3.83 (s, 3H), 2.97 (dd, *J* = 13.4 Hz, 7.7 Hz, 1H), 2.88 (dd, *J* = 13.5 Hz, 3.2 Hz, 1H), 1.68 (s, 2H); for ^1^H NMR spectrum of the isolated minor isomer, see the next section.

#### 4.2.130 Methyl 3-(2-Acetamido-1-phenylethoxy)-4-nitrobenzoate (**30s38**)

The above mixture of two isomers (768 mg, 2.43 mmol) was dissolved in prop-1-en-2-yl acetate (5.35 mL, 48.6 mmol) and stirred overnight at 22 °C. The excess reagent was removed under reduced pressure, and the residue was purified by column chromatography, eluent EtOAc/hexane = 1:1 → 3:1, to obtain product **30s38** (562 mg, 65% yield) and 224 mg of unreacted methyl 3-((2-hydroxy-2-phenylethyl)amino)-4-nitrobenzoate.

#### 4.2.131 Methyl 3-(2-Acetamido-1-phenylethoxy)-4-nitrobenzoate (**30s38**)

^1^H NMR (400 MHz, DMSO-*d*_6_): δ 8.15 (t, *J* = 5.4 Hz, 1H), 7.97 (d, *J* = 8.3 Hz, 1H), 7.68 (d, *J* = 1.5 Hz, 1H), 7.61 (dd, *J* = 8.3 Hz, 1.5 Hz, 1H), 7.42 (m, 4H), 7.36–7.28 (m, 1H), 5.69 (m, 1H), 3.84 (s, 3H), 3.54–3.39 (m, 1H), 1.80 (s, 3H); MS (ESI): *m*/*z* calcd for C_18_H_18_N_2_O_6_: 358.12. Found: 359.1 [M + H]^+^.

#### 4.2.132 Methyl 3-((2-Hydroxy-2-phenylethyl)amino)-4-nitrobenzoate

^1^H NMR (400 MHz, DMSO-*d*_6_) δ 8.33 (t, *J* = 5.3 Hz, 1H), 8.17 (d, *J* = 8.8 Hz, 1H), 7.55 (d, *J* = 1.7 Hz, 1H), 7.49–7.43 (m, 2H), 7.39–7.33 (m, 2H), 7.31–7.25 (m, 1H), 7.14 (dd, *J* = 8.8, 1.7 Hz, 1H), 5.88 (d, *J* = 4.4 Hz, 1H), 4.91 (dt, *J* = 8.2, 4.2 Hz, 1H), 3.87 (s, 3H), 3.71–3.58 (m, 1H), 3.51–3.43 (m, 1H).

#### 4.2.133 Methyl 3-(2-Acetamido-1-phenylethoxy)-4-aminobenzoate (**31s38**)

Prepared according to the typical procedure B from **30s38** (500 mg, 1.40 mmol); white solid (312 mg, 68% yield). ^1^H NMR (400 MHz, DMSO-*d*_6_): δ 8.19 (t, *J* = 5.8 Hz, 1H), 7.40–7.32 (m, 4H), 7.32–7.24 (m, 2H), 7.04 (d, *J* = 1.7 Hz, 1H), 6.61 (d, *J* = 8.3 Hz, 1H), 5.86 (s, 2H), 5.28 (dd, *J* = 7.1 Hz, 4.1 Hz, 1H), 3.64 (s, 3H), 3.57–3.42 (m, 2H), 1.81 (s, 3H); MS (ESI): *m*/*z* calcd for C_18_H_20_N_2_O_4_: 328.14. Found: 329.1 [M + H]^+^.

#### 4.2.134 Methyl 4-(2-Acetamido-1-phenylethoxy)-2-aminobenzo-[*d*]thiazole-6-carboxylate (**32s38**)

Prepared according to the typical procedure C from aniline **31s38** (283 mg, 0.862 mmol); yellow solid (216 mg, 65% yield). ^1^H NMR (400 MHz, DMSO-*d*_6_): δ 8.20 (t, *J* = 5.2 Hz, 1H), 7.95 (s, 2H), 7.89 (d, *J* = 1.5 Hz, 1H), 7.44–7.39 (m, 2H), 7.39–7.31 (m, 2H), 7.31–7.24 (m, 2H), 5.62–5.55 (m, 1H), 3.76 (3, 1H), 3.47 (t, *J* = 5.9 Hz, 2H), 1.82 (s, 3H); MS (ESI): *m*/*z* calcd for C_19_H_19_N_3_O_4_S: 385.11. Found: 386.0 [M + H]^+^.

#### 4.2.135 Methyl 4-(2-Acetamido-1-phenylethoxy)-2-(3,4-dichloro-5-methyl-1*H*-pyrrole-2-carboxamido)benzo[*d*]thiazole-6-carboxylate (**33s38**)

Prepared according to the typical procedure D from 2-aminobenzothiazole 32s38 (110 mg, 0.285 mmol); gray solid (90 mg, 56% yield). ^1^H NMR (400 MHz, DMSO-*d*_6_): δ 12.36 (s, 2H), 8.32–8.22 (m, 2H), 7.56–7.50 (m, 2H), 7.49 (s, 1H), 7.47–7.41 (m, 2H), 7.39–7.34 (m, 1H), 5.78 (dd, *J* = 7.1 Hz, 5.7 Hz, 1H), 3.89 (s, 3H), 3.72–3.63 (m, 1H), 3.63–3.54 (m, 1H), 2.37 (s, 3H), 1.91 (s, 3H); MS (ESI): *m*/*z* calcd for C_25_H_22_Cl_2_N_4_O_5_S: 560.07. Found: 559.0 [M – H]^−^.

#### 4.2.136 4-(2-Acetamido-1-phenylethoxy)-2-(3,4-dichloro-5-methyl-1*H*-pyrrole-2-carboxamido)benzo[*d*]thiazole-6-carboxylic Acid (**38**)

Prepared according to the typical procedure E from the methyl ester **33s38** (70 mg, 0.125 mmol); dark gray solid (30 mg, 44% yield). ^1^H NMR (400 MHz, DMSO-*d*_6_): δ 12.90 (s, 1H), 12.28 (s, 2H), 8.19 (t, *J* = 5.3 Hz, 1H), 8.16 (s, 1H), 7.48–7.42 (m, 2H), 7.41–7.33 (m, 3H), 7.33–7.25 (m, 1H), 5.68 (dd, *J* = 7.4 Hz, 5.2 Hz, 1H), 3.66–3.45 (m, 2H), 2.29 (s, 3H), 1.83 (s, 3H); ^13^C NMR (101 MHz, DMSO-*d*_6_): δ 169.70, 166.84, 159.88, 156.94, 148.94, 142.09, 138.88, 132.83, 130.00, 128.70 (2C), 128.14, 126.52, 126.22 (2C), 117.03, 116.34, 115.71, 110.49, 110.01, 78.10, 45.75, 22.51, 11.10; HRMS (ESI) *m*/*z*: [M + H]^+^ calcd for C_24_H_21_Cl_2_N_4_O_5_S 547.0610; found 547.0602; HPLC purity (254 nm): 93%.

#### 4.2.137 Methyl 3-(2-((*tert*-Butoxycarbonyl)amino)-1-phenyletho-xy)-4-nitrobenzoate (**30s39**)

Prepared according to the typical procedure H from **2s** (2.31 g, 11.72 mmol) and *tert*-butyl (2-hydroxy-2-phenylethyl)carbamate (3.06 g, 12.89 mmol); yellow oil (3.76 g, 77% yield). ^1^H NMR (400 MHz, DMSO-*d*_6_): δ 7.97 (d, *J* = 8.3 Hz, 1H), 7.68 (s, 1H), 7.60 (dd, *J* = 8.3 Hz, 1.5 Hz, 1H), 7.47–7.28 (m, 5H), 7.09 (t, *J* = 5.5 Hz, 1H), 5.73–5.65 (m, 1H), 3.84 (s, 3H), 3.47–3.37 (m, 1H), 3.34–3.27 (m, 1H), 1.35 (s, 9H); MS (ESI): *m*/*z* calcd for C_21_H_24_N_2_O_7_: 416.16. Found: 439.0 [M + Na]^+^.

#### 4.2.138 Methyl 4-Amino-3-(2-((*tert*-butoxycarbonyl)amino)-1-phenylethoxy)benzoate (**31s39**)

Prepared according to the typical procedure B from **30s39** (3.08 g, 7.39 mmol); white solid (2.17 g, 76% yield). ^1^H NMR (400 MHz, DMSO-*d*_6_): δ 7.38–7.32 (m, 4H), 7.32–7.24 (m, 3H), 7.01 (d, *J* = 1.6 Hz, 1H), 6.61 (d, *J* = 8.3 Hz, 1H), 5.92 (s, 2H), 5.27–5.21 (m, 1H), 3.64 (s, 3H), 3.47–3.35 (m, 2H), 1.41–1.20 (m, 9H); MS (ESI): *m*/*z* calcd for C_21_H_26_N_2_O_5_: 386.18. Found: 408.9 [M + Na]^+^.

#### 4.2.139 Methyl 2-Amino-4-(2-((*tert*-butoxycarbonyl)amino)-1-phenylethoxy)benzo[*d*]thiazole-6-carboxylate (**32s39**)

Prepared according to the typical procedure C from aniline **31s39** (2.04 g, 5.28 mmol); yellow solid (726 mg, 31% yield). ^1^H NMR (400 MHz, DMSO-*d*_6_): δ 7.96 (s, 2H), 7.88 (s, 1H), 7.44–7.37 (m, 2H), 7.38–7.30 (m, 2H), 7.30–7.21 (m, 2H), 7.14–7.02 (m, 1H), 5.66–5.50 (m, 1H), 3.76 (s, 3H), 3.44–3.24 (m, 2H), 1.34 (s, 9H); MS (ESI): *m*/*z* calcd for C_22_H_25_N_3_O_5_S: 443.15. Found: 442.0 [M – H]^−^.

#### 4.2.140 Methyl 4-(2-((*tert*-Butoxycarbonyl)amino)-1-phenyletho-xy)-2-(3,4-dichloro-5-methyl-1*H*-pyrrole-2-carboxamido)-benzo[*d*]thiazole-6-carboxylate (**33s39**)

Prepared according to the typical procedure D from **32s39** (256 mg, 0.578 mmol); gray solid (68 mg, 19% yield). ^1^H NMR (400 MHz, DMSO-*d*_6_): δ 12.32 (s, 1H), 12.30 (s, 1H), 8.21 (s, 1H), 7.47–7.25 (m, 6H), 7.14 (s, 1H), 5.68 (t, *J* = 6.2 Hz, 1H), 3.81 (s, 3H), 3.55–3.40 (m, 2H), 2.29 (s, 3H), 1.34 (s, 9H); MS (ESI): *m*/*z* calcd for C_28_H_28_Cl_2_N_4_O_6_S: 618.11. Found: 617.0 [M – H]^−^.

#### 4.2.141 4-(2-((*tert*-Butoxycarbonyl)amino)-1-phenylethoxy)-2-(3,4-dichloro-5-methyl-1*H*-pyrrole-2-carboxamido)benzo[*d*]thiazole-6-carboxylic Acid (**39**)

Prepared according to the typical procedure E from methyl ester **33s39** (56 mg, 0.091 mmol); pink-tinted solid (50 mg, 91% yield). ^1^H NMR (400 MHz, DMSO-d_6_): δ = 12.98 (s, 1H), 12.36 (s, 2H), 8.24 (s, 1H), 7.54–7.49 (m, 2H), 7.48–7.42 (m, 3H), 7.36 (t, *J* = 7.3 Hz, 1H), 7.20 (s, 1H), 5.75 (dd, *J* = 6.6 Hz, 5.7 Hz, 1H), 3.63–3.53 (m, 2H), 2.37 (s, 3H), 1.42 (s, 9H); HRMS (ESI) *m*/*z*: [M + H]^+^ calcd for C_27_H_27_Cl_2_N_4_O_6_S 605.1028; found 605.1017; HPLC purity (254 nm): 93%.

#### 4.2.142 Methyl 4-Nitro-3-(1-phenyl-2-(1*H*-1,2,4-triazol-1-yl)ethoxy)benzoate (**30s40**)

#### 4.2.142.1 1-Phenyl-2-(1*H*-1,2,4-triazol-1-yl)ethan-1-ol (**29s40**)

To a solution of styrene oxide (**28s**; R = Ph) (3.00 g, 24.97 mmol) and 1,2,4-triazole (1.72 g, 24.97 mmol) in DMF (125 mL), K_2_CO_3_ (5.18 g, 37.5 mmol) was added and the reaction mixture was stirred at 90 °C for 20 h. The volatiles were removed under reduced pressure, and the residue was suspended in boiling EtOAc and filtered. The filtrate was concentrated, and the residue was triturated with cold EtOAc to give a white solid (1.91 g, 40% yield). ^1^NMR (400 MHz, DMSO-*d*_6_): δ 8.36 (s, 1H), 7.95 (s, 1H), 7.38–7.25 (m, 5H), 5.75 (s, 1H), 4.93 (dd, *J* = 7.3 Hz, 5.5 Hz, 1H), 4.37–4.26 (m, 2H); MS (ESI): *m*/*z* calcd for C_10_H_11_N_3_O: 189.09. Found: 190.1 [M + H]^+^.

#### 4.2.143 Methyl 4-Nitro-3-(1-phenyl-2-(1*H*-1,2,4-triazol-1-yl)ethoxy)benzoate (**30s40**)

Prepared according to the typical procedure H from **2s** (2.17 g, 11.03 mmol) and **29s40** (2.30 g, 12.13 mmol); white solid (2.52 g, 62% yield). ^1^H NMR (400 MHz, DMSO-*d*_6_): δ 8.41 (s, 1H), 7.94 (m, 2H), 7.65–7.54 (m, 2H), 7.51–7.26 (m, 5H), 6.17 (dd, *J* = 8.1 Hz, 3.5 Hz, 1H), 4.80 (dd, *J* = 14.5 Hz, 8.3 Hz, 1H), 4.63 (dd, *J* = 14.4 Hz, 3.6 Hz, 1H), 3.82 (s, 3H).

#### 4.2.144 Methyl 4-Amino-3-(1-phenyl-2-(1*H*-1,2,4-triazol-1-yl)ethoxy)benzoate (**31s40**)

Prepared according to the typical procedure B from **30s40** (2.50 g, 6.78 mmol); colorless oil (1.56 g, 68% yield). ^1^H NMR (400 MHz, DMSO-*d*_6_): δ 8.64 (s, 1H), 7.97 (s, 1H), 7.47 (m, 2H), 7.33 (m, 3H), 7.27 (dd, *J* = 8.3 Hz, 1.7 Hz, 1H), 7.06 (d, *J* = 1.7 Hz, 1H), 6.58 (d, *J* = 8.3 Hz, 1H), 5.83 (s, 2H), 5.69 (dd, *J* = 8.8 Hz, 3.3 Hz, 1H), 4.75 (dd, *J* = 14.2 Hz, 8.8 Hz, 1H), 4.54 (dd, *J* = 14.1 Hz, 3.3 Hz, 1H), 3.65 (s, 3H); MS (ESI): *m*/*z* calcd for C_18_H_18_N_4_O_3_: 338.14. Found: 339.2 [M + H]^+^.

#### 4.2.145 Methyl 2-Amino-4-(1-phenyl-2-(1*H*-1,2,4-triazol-1-yl)ethoxy)benzo[*d*]thiazole-6-carboxylate (**32s40**)

Prepared according to the typical procedure C from aniline **31s40**; (1.39 g, 4.12 mmol); white solid (228 mg, 14% yield). ^1^H NMR (400 MHz, DMSO-*d*_6_): δ 8.53 (s, 1H), 7.96 (s, 2H), 7.94 (s, 1H), 7.88 (d, *J* = 1.5 Hz, 1H), 7.47–7.39 (m, 2H), 7.39–7.30 (m, 2H), 7.34–7.24 (m, 1H), 7.23 (d, *J* = 1.6 Hz, 1H), 6.09 (dd, *J* = 8.0 Hz, 4.4 Hz, 1H), 4.77 (dd, *J* = 14.2 Hz, 8.1 Hz, 1H), 4.62 (dd, *J* = 14.2 Hz, 4.4 Hz, 1H), 3.75 (s, 3H); MS (ESI): *m*/*z* calcd for C_19_H_17_N_5_O_3_S: 395.11. Found: 396.2 [M + H]^+^.

#### 4.2.146 Methyl 2-(3,4-Dichloro-5-methyl-1*H*-pyrrole-2-carboxamido)-4-(1-phenyl-2-(1*H*-1,2,4-triazol-1-yl)ethoxy)benzo[*d*]-thiazole-6-carboxylate (**33s40**)

Prepared according to the typical procedure D from 32s40 (105 mg, 0.266 mmol); gray solid (140 mg, 92% yield). ^1^H NMR (400 MHz, DMSO-*d*_6_): δ 12.34 (s, 1H), 12.24 (s, 1H), 8.55 (s, 1H), 8.20 (s, 1H), 7.97 (s, 1H), 7.49 (d, *J* = 7.5 Hz, 2H), 7.43–7.27 (m, 4H), 6.19 (dd, *J* = 8.1 Hz, 4.4 Hz, 1H), 4.83 (dd, *J* = 14.2 Hz, 8.2 Hz, 1H), 4.69 (dd, *J* = 14.2 Hz, 4.5 Hz, 1H), 3.81 (s, 3H), 2.30 (s, 3H); MS (ESI): *m*/*z* calcd for C_25_H_20_Cl_2_N_6_O_4_S: 570.06. Found: 571.4 [M + H]^+^.

#### 4.2.147 2-(3,4-Dichloro-5-methyl-1*H*-pyrrole-2-carboxamido)-4-(1-phenyl-2-(1*H*-1,2,4-triazol-1-yl)ethoxy)benzo[*d*]thiazole-6-carboxylic Acid (**40**)

Prepared according to the typical procedure E from methyl ester **33s40** (51 mg, 0.090 mmol); light brown solid (18 mg, 36% yield). ^1^H NMR (400 MHz, DMSO-*d*_6_): δ 12.90 (s, 1H), 12.34 (s, 1H), 12.21 (s, 1H), 8.54 (s, 1H), 8.17 (s, 1H), 7.96 (s, 1H), 7.48 (d, *J* = 7.6 Hz, 2H), 7.42–7.26 (m, 4H), 6.27–6.10 (m, 1H), 4.94–4.76 (m, 1H), 4.75–4.59 (m, 1H), 2.30 (s, 3H); HRMS (ESI) *m*/*z*: [M + H]^+^ calcd for C_24_H_19_Cl_2_N_6_O_4_S 557.0566; found 557.0555; HPLC purity (254 nm): 94%.

#### 4.2.148 4-(2-(4-Aminopiperidin-1-yl)-1-phenylethoxy)-2-(3,4-dichloro-5-methyl-1*H*-pyrrole-2-carboxamido)benzo[*d*]thiazole-6-carboxylic Acid Hydrochloride (**41**)

Boc-protected compound **43** (30 mg, 0.044 mmol) was dissolved in THF (1 mL), and 4 M of HCl in 1,4-dioxane (2 mL) was added and the reaction mixture was stirred at 22 °C overnight. After the addition of an additional 2 mL of 4 M HCl in 1,4-dioxane, the reaction mixture was stirred for 2 more days and followed with HPLC-MS. The precipitate that formed in the reaction mixture was filtered off, washed with ether, and dried; pale pink solid (25 mg, 92% yield). ^1^H NMR (400 MHz, DMSO-*d*_6_): δ 13.24 (s, 1H), 12.49 (s, 1H), 10.63 (s, 1H), 8.45–8.26 (m, 3H), 8.24 (s, 1H), 7.59–7.47 (m, 2H), 7.46–7.38 (m, 2H), 7.38–7.31 (m, 2H), 6.42 (d, *J* = 9.9 Hz, 1H), 4.16–3.93 (m, 2H), 3.91–3.69 (m, 2H), 3.40–3.22 (m, 3H), 2.35–2.21 (m, 5H), 2.06–1.89 (m, 2H); HRMS (ESI) *m*/*z*: [M – Cl]^+^ calcd for C_27_H_28_Cl_2_N_5_O_4_S 588.1239; found 588.1223; HPLC purity (254 nm): 98%.

#### 4.2.149 Methyl 3-(2-(4-(Dimethylamino)piperidin-1-yl)-1-phenyl-ethoxy)-4-nitrobenzoate (**30s42**)

#### 4.2.149.1 Methyl 3-(2-(4-Aminopiperidin-1-yl)-1-phenylethoxy)-4-nitrobenzoate

To a suspension of methyl 3-(2-(4-((*tert*-butoxycarbonyl)amino)piperidin-1-yl)-1-phenylethoxy)-4-nitrobenzoate (**30s43**) (1.3 g, 2.6 mmol) in 1,4-dioxane (4 mL), 4 M HCl in 1,4-dioxane (5 mL) was added. The reaction mixture was stirred at 22 °C for 3 days while monitoring with HPLC-MS. The volatiles were evaporated under reduced pressure, and the residue was partitioned between EtOAc and saturated aqueous NaHCO_3_ solution. The organic layer was washed with brine, dried over Na_2_SO_4_, filtered, and concentrated to give the product as a yellow oil (955 mg, 92% yield). ^1^H NMR (400 MHz, DMSO-*d*_6_) δ 7.94 (d, *J* = 8.3 Hz, 1H), 7.82 (d, *J* = 1.6 Hz, 1H), 7.59 (dd, *J* = 8.4, 1.6 Hz, 1H), 7.47–7.39 (m, 2H), 7.41–7.32 (m, 2H), 7.34–7.23 (m, 1H), 5.83 (dd, *J* = 8.0, 3.7 Hz, 1H), 3.85 (s, 3H), 3.37–2.98 (m, 4H), 2.93–2.76 (m, 4H), 2.59 (dd, *J* = 13.8, 3.7 Hz, 1H), 2.10 (dtd, *J* = 23.1, 11.3, 2.5 Hz, 2H), 1.65–1.53 (m, 2H), 1.26–1.03 (m, 2H) (some signals overlayed by the DMSO peak). MS (ESI): *m*/*z* calcd for C_21_H_25_N_3_O_5_: 399.18. Found: 400.0 [M + H]^+^.

#### 4.2.150 Methyl 3-(2-(4-(Dimethylamino)piperidin-1-yl)-1-phenyl-ethoxy)-4-nitrobenzoate (**30s42**)

To a solution of methyl 3-(2-(4-aminopiperidin-1-yl)-1-phenylethoxy)-4-nitrobenzoate (899 mg, 2.25 mmol) in methanol (25 mL), paraformaldehyde (612 mg, 20.38 mmol) was added and the mixture was stirred for 30 min at 0 °C. NaCNBH_3_ (198 mg, 3.15 mmol) was added, and the reaction mixture was stirred at 22 °C overnight. The solvent was evaporated in vacuo, ethyl acetate was added, and the suspension was washed with water and brine. The organic layer was dried over Na_2_SO_4_, filtered, and evaporated to give a crude product that was purified by flash column chromatography (eluent: dichloromethane/methanol 40:1–9:1); white solid (355 mg, 37% yield). ^1^H NMR (400 MHz, DMSO-*d*_6_): δ 7.94 (d, *J* = 8.4 Hz, 1H), 7.85 (d, *J* = 1.6 Hz, 1H), 7.59 (dd, *J* = 8.3, 1.6 Hz, 1H), 7.48–7.40 (m, 2H), 7.42–7.33 (m, 2H), 7.34–7.25 (m, 1H), 5.86 (dd, *J* = 8.2, 3.4 Hz, 1H), 3.85 (s, 3H), 3.01–2.87 (m, 2H), 2.84 (dd, *J* = 13.8, 8.3 Hz, 1H), 2.59 (dd, *J* = 13.9, 3.4 Hz, 1H), 2.54–2.51 (m, 1H), 2.15 (s, 6H), 2.12–1.99 (m, 2H), 1.69–1.57 (m, 2H), 1.29–1.11 (m, 2H). MS (ESI): *m*/*z* calcd for C_23_H_29_N_3_O_5_: 427.21. Found: 427.9 [M + H]^+^.

#### 4.2.151 Methyl 4-Amino-3-(2-(4-(dimethylamino)piperidin-1-yl)-1-phenylethoxy)benzoate (**31s42**)

To a solution of **30s42** (350 mg, 0.819 mmol) in ethyl acetate/methanol (2:1, 30 mL), SnCl_2_ (621 mg, 3.27 mmol) was added and the reaction mixture was stirred at 55 °C overnight. It was concentrated in vacuo, neutralized with saturated NaHCO_3_ solution to pH = 8, and the water layer was extracted with ethyl acetate (4 × 70 mL). The combined organic layers were dried over Na_2_SO_4_, filtered, and the solvent was removed in vacuo; yellow oil (304 mg, 94% yield). ^1^H NMR (400 MHz, DMSO-*d*_6_) δ 7.47–7.39 (m, 2H), 7.41–7.31 (m, 3H), 7.34–7.24 (m, 1H), 7.09 (d, *J* = 1.9 Hz, 1H), 6.64 (d, *J* = 8.3 Hz, 1H), 6.20 (s, 2H), 5.11 (d, *J* = 8.7 Hz, 1H), 3.16–3.03 (m, 2H), 2.97 (dd, *J* = 13.6, 9.1 Hz, 1H), 2.49–2.31 (m, 7H), 2.22 (t, *J* = 11.5 Hz, 1H), 2.13–2.02 (m, 1H), 1.86–1.75 (m, 2H), 1.55–1.38 (m, 2H) (1H overlapped by the DMSO peak). MS (ESI): *m*/*z* calcd for C_23_H_31_N_3_O_3_: 397.24. Found: 398.0 [M + H]^+^.

#### 4.2.152 Methyl 2-Amino-4-(2-(4-(dimethylamino)piperidin-1-yl)-1-phenylethoxy)benzo[*d*]thiazole-6-carboxylate (**32s42**)

Prepared according to the typical procedure C from aniline **31s42** (292 mg, 0.734 mmol); pale yellow oil (107 mg, 32% yield). ^1^H NMR (400 MHz, DMSO-*d*_6_): δ 7.93 (s, 2H), 7.87 (d, *J* = 1.5 Hz, 1H), 7.49–7.39 (m, 2H), 7.37–7.28 (m, 3H), 7.24 (t, *J* = 7.2 Hz, 1H), 5.78 (dd, *J* = 8.9, 3.4 Hz, 1H), 3.76 (s, 3H), 3.24–3.10 (m, 2H), 2.97 (dd, *J* = 13.5, 8.1 Hz, 2H), 2.75–2.61 (m, 7H), 2.25–2.14 (m, 2H), 1.95–1.81 (m, 2H), 1.62–1.40 (m, 1H).

#### 4.2.153 Methyl 2-(3,4-Dichloro-5-methyl-1*H*-pyrrole-2-carboxamido)-4-(2-(4-(dimethylamino)piperidin-1-yl)-1-phenylethoxy)benzo[*d*]thiazole-6-carboxylate (**33s42**)

Prepared according to the typical procedure J from 2-aminobenzothiazole 32s42 (94 mg, 0.207 mmol); black solid (34 mg, 26% yield). ^1^H NMR (characteristic signals) (400 MHz, DMSO-*d*_6_): δ 8.02 (s, 1H), 7.47 (d, *J* = 7.5 Hz, 2H), 7.38–7.30 (m, 3H), 7.26 (t, *J* = 7.3 Hz, 1H), 5.81 (dd, *J* = 8.5, 3.7 Hz, 1H), 3.78 (s, 3H), 2.34 (s, 6H), 2.24 (s, 3H), 1.85–1.73 (m, 2H), 1.55–1.37 (m, 2H); HRMS (ESI) *m*/*z*: [M + H]^+^ calcd for C_30_H_34_Cl_2_N_5_O_4_S 630.1709; found 630.1687.

#### 4.2.154 2-(3,4-Dichloro-5-methyl-1*H*-pyrrole-2-carboxamido)-4-(2-(4-(dimethylamino)piperidin-1-yl)-1-phenylethoxy)benzo-[*d*]thiazole-6-carboxylic Acid (**42**)

Prepared according to the typical procedure E from the methyl ester 33s42 (25 mg, 0.040 mmol); white solid (11 mg, 45% yield). ^1^H NMR (400 MHz, DMSO-*d*_6_): δ 13.60 (s, 1H), 12.93 (s, 1H), 12.64 (s, 1H), 11.21 (s, 1H), 10.92 (s, 1H), 8.23 (s, 1H), 7.52 (d, *J* = 8.0 Hz, 2H), 7.41 (t, *J* = 7.4 Hz, 3H), 7.35 (d, *J* = 8.2 Hz, 2H), 6.46 (d, *J* = 10.0 Hz, 1H), 2.71 (s, 6H), 2.31 (s, 3H); HRMS (ESI): *m*/*z* calcd for C_29_H_30_Cl_2_N_5_O_4_S 614.1396; found 614.1403 [M – H]^−^; HPLC purity (254 nm): 95%.

#### 4.2.154.1 *tert*-Butyl (1-(2-Hydroxy-2-phenylethyl)piperidin-4-yl)carbamate (**29s43**)

4-Boc-aminopiperidine (3.00 g, 14.99 mmol) and styrene oxide (1.71 mL, 14.99 mmol) were stirred in a pressure tube at 90 °C for 4 h. The solid crude product was suspended in hexane and filtered off to give a white solid (3.50 g, 73% yield); ^1^H NMR (400 MHz, DMSO-*d*_6_) δ 7.36–7.16 (m, 5H), 6.74 (d, *J* = 7.9 Hz, 1H), 4.92 (d, *J* = 3.8 Hz, 1H), 4.65 (dt, *J* = 7.9, 4.0 Hz, 1H), 3.24–3.11 (m, 1H), 2.92–2.79 (m, 2H), 2.49–2.39 (m, 1H), 2.33 (dd, *J* = 12.8, 4.5 Hz, 1H), 2.11–1.95 (m, 2H), 1.70–1.56 (m, 2H), 1.39–1.35 (m, 11H); MS (ESI): *m*/*z* calcd for C_18_H_28_N_2_O_3_: 320.21. Found: 321.1 [M + H]^+^.

#### 4.2.155 Methyl 3-(2-(4-((*tert*-Butoxycarbonyl)amino)piperidin-1-yl)-1-phenylethoxy)-4-nitrobenzoate (**30s43**)

Prepared according to the typical procedure H from **2s** (1.79 g, 9.07 mmol) and **29s43** (3.20 g 9.98 mmol); yellow solid (2.81 g, 62%). ^1^H NMR (400 MHz, DMSO-*d*_6_): δ 7.94 (d, *J* = 8.4 Hz, 1H), 7.80 (d, *J* = 1.6 Hz, 1H), 7.59 (dd, *J* = 8.4, 1.5 Hz, 1H), 7.47–7.39 (m, 2H), 7.42–7.33 (m, 2H), 7.34–7.25 (m, 1H), 6.71 (d, *J* = 7.8 Hz, 1H), 5.85 (dd, *J* = 7.9, 3.6 Hz, 1H), 3.85 (s, 3H), 3.20–3.08 (m, 1H), 2.95–2.79 (m, 3H), 2.60 (dd, *J* = 13.8, 3.6 Hz, 1H), 2.19–2.05 (m, 2H), 1.66–1.55 (m, 2H), 1.37 (s, 9H), 1.31–1.21 (m, 2H); MS (ESI): *m*/*z* calcd for C_26_H_33_N_3_O_7_: 499.23. Found: 500.1 [M + H]^+^.

#### 4.2.156 Methyl 4-Amino-3-(2-(4-((*tert*-butoxycarbonyl)amino)piperidin-1-yl)-1-phenylethoxy)benzoate (**31s43**)

Prepared according to the typical procedure B from **30s43** (1.90 g, 3.80 mmol); yellow solid (1.57 g, 88% yield). ^1^H NMR (400 MHz, DMSO-*d*_6_): δ 7.47–7.39 (m, 2H), 7.40–7.31 (m, 3H), 7.33–7.24 (m, 1H), 7.10 (d, *J* = 1.9 Hz, 1H), 6.71 (d, *J* = 7.6 Hz, 1H), 6.65 (d, *J* = 8.4 Hz, 1H), 6.12 (s, 2H), 5.11 (dd, *J* = 8.9, 2.9 Hz, 1H), 3.65 (s, 3H), 3.26–3.14 (m, 1H), 3.03–2.85 (m, 3H), 2.43 (dd, *J* = 13.5, 3.0 Hz, 1H), 2.28–2.19 (m, 1H), 2.15–2.05 (m, 1H), 1.75–1.65 (m, 2H), 1.46–1.30 (m, 11H); MS (ESI): *m*/*z* calcd for C_26_H_35_N_3_O_5_: 469.26. Found: 470.2 [M + H]^+^.

#### 4.2.157 Methyl 2-Amino-4-(2-(4-((*tert*-butoxycarbonyl)amino)piperidin-1-yl)-1-phenylethoxy)benzo[*d*]thiazole-6-carboxylate (**32s43**)

Prepared according to the typical procedure C from aniline **31s43** (1.36 g, 2.90 mmol); yellow solid (443 mg. 29% yield). ^1^H NMR (400 MHz, DMSO-*d*_6_) δ 7.94 (s, 2H), 7.85 (d, *J* = 1.5 Hz, 1H), 7.45–7.38 (m, 2H), 7.36–7.27 (m, 3H), 7.28–7.18 (m, 1H), 6.73 (d, *J* = 7.9 Hz, 1H), 5.74 (dd, *J* = 7.6, 4.1 Hz, 1H), 3.76 (s, 3H), 3.23–3.11 (m, 1H), 3.00 (d, *J* = 10.4 Hz, 1H), 2.95–2.86 (m, 2H), 2.59 (dd, *J* = 13.5, 4.0 Hz, 1H), 2.19–2.05 (m, 2H), 1.64 (d, *J* = 9.4 Hz, 2H), 1.41–1.27 (m, 11H); MS (ESI): *m*/*z* calcd for C_27_H_34_N_4_O_5_S: 526.23. Found: 527.0 [M + H]^+^.

#### 4.2.158 Methyl 4-(2-(4-((*tert-*Butoxycarbonyl)amino)piperidin-1-yl)-1-phenylethoxy)-2-(3,4-dichloro-5-methyl-1*H*-pyrrole-2-carboxamido)benzo[*d*]thiazole-6-carboxylate (**33s43**)

Prepared according to the typical procedure J from 2-aminobenzothiazole **32s43** (139 mg, 0.265 mmol); gray solid (93 mg, 50% yield). ^1^H NMR (400 MHz, DMSO-*d*_6_): δ 12.27 (s, 2H), 8.16 (s, 1H), 7.53–7.21 (m, 6H), 6.74 (s, 1H), 5.79 (s, 1H), 3.80 (s, 3H), 3.06 (d, *J* = 23.1 Hz, 3H), 2.28 (s, 3H), 1.82–1.60 (m, 2H), 1.43–1.31 (m, 13H) (some piperidine signals are overlapped by water and DMSO peaks); MS (ESI): *m*/*z* calcd for C_33_H_37_Cl_2_N_5_O_6_S: 701.18. Found: 702.2 [M + H]^+^.

#### 4.2.159 4-(2-(4-((*tert*-Butoxycarbonyl)amino)piperidin-1-yl)-1-phenylethoxy)-2-(3,4-dichloro-5-methyl-1*H*-pyrrole-2-carboxamido)benzo[*d*]thiazole-6-carboxylic Acid (**43**)

Prepared according to the typical procedure E from methyl ester **33s43** (80 mg, 0.113 mmol); off-white solid (43 mg, 55% yield). ^1^H NMR (400 MHz, DMSO-*d*_6_) δ 12.77 (s, 1H), 12.24 (s, 2H), 8.12 (s, 1H), 7.53–7.22 (m, 6H), 6.73 (d, *J* = 6.6 Hz, 1H), 5.77 (s, 1H), 3.28–3.19 (m, 1H), 3.13–2.89 (m, 1H), 2.28 (s, 3H), 1.79–1.61 (m, 2H), 1.43–1.30 (m, 13H) (some piperidine signals are overlapped by water and DMSO peaks); HRMS (ESI) *m*/*z*: [M + H]^+^ calcd for C_32_H_36_Cl_2_N_5_O_6_S 688.1763; found 688.1747; HPLC purity (254 nm): 97%.

#### 4.2.160 2-(3,4-Dichloro-5-methyl-1*H*-pyrrole-2-carboxamido)-4-(2-(4-methylpiperazin-1-yl)-1-phenylethoxy)benzo[*d*]thiazole-6-carboxylic Acid (**44**)

Prepared according to the typical procedure E from methyl ester **33s44** (42 mg, 0.070 mmol) that was synthesized in analogy to **33s36**; white crispy foam (7 mg, 17% yield). ^1^H NMR (500 MHz, CD_3_OD): δ 8.49 (s, 1H), 8.07 (d, *J* = 1.3 Hz, 1H), 7.58 (d, *J* = 1.4 Hz, 1H), 7.53–7.48 (m, 2H), 7.39–7.32 (m, 2H), 7.31–7.25 (m, 1H), 5.87 (dd, *J* = 8.8 Hz, 3.1 Hz, 1H), 3.30 (dd, *J* = 14.2 Hz, 8.9, Hz, 1H), 3.18–3.00 (m, 8H), 2.96 (dd, *J* = 13.9 Hz, 3.0 Hz, 1H), 2.72 (s, 3H), 2.34 (s, 3H); HRMS (ESI) *m*/*z*: [M + H]^+^ calcd for C_27_H_28_Cl_2_N_5_O_4_S 588.1239; found 588.1207; HPLC (254 nm): 97%.

#### 4.2.161 4-(2-((6-Carboxy-2-(3,4-dichloro-5-methyl-1*H*-pyrrole-2-carboxamido)benzo[*d*]thiazol-4-yl)oxy)-2-phenylethyl)-1,1-dimethylpiperazin-1-ium Formate (**45**)

Prepared according to the typical procedure E from hydrochloride of methyl ester 33s45 (154 mg, 0.236 mmol; synthesized by methylation of methyl ester 33s44) and purified by reverse phase chromatography to give the title compound as a formic acid salt; white solid (75 mg, 49% yield). ^1^H NMR (500 MHz, CD_3_OD): δ 8.33 (s, 1H), 8.08 (d, *J* = 1.3 Hz, 1H), 7.53 (d, *J* = 1.5 Hz, 1H), 7.50–7.45 (m, 2H), 7.33 (t, *J* = 7.6 Hz, 2H), 7.25 (t, *J* = 7.4 Hz, 1H), 5.77 (dd, *J* = 8.5 Hz, 3.3 Hz, 1H), 3.40 (t, *J* = 5.2 Hz, 4H), 3.24 (dd, *J* = 14.4 Hz, 9.0 Hz, 1H), 3.12 (s, 6H), 3.16–3.07 (m, 2H), 3.07–3.00 (m, 2H), 2.93 (dd, *J* = 14.0 Hz, 3.3 Hz, 1H), 2.31 (s, 3H); ^13^C NMR (APT) (126 MHz, CD_3_OD): δ 170.82, 161.14, 158.22, 150.41, 140.62, 134.40, 132.79, 130.99, 129.76, 129.24, 127.51, 118.35, 117.10, 115.28, 113.14, 111.93, 80.59, 64.65, 63.13, 48.05, 40.39, 11.06; HRMS (ESI): *m*/*z* calcd for C_28_H_30_Cl_2_N_5_O_4_S: 602.1396. Found 602.1364 [M – HCOO^–^]^+^; HPLC purity (254 nm): 94%.

#### 4.2.162 *tert*-Butyl 4-Nitro-3-isopropoxybenzoate (**3s46**)

Prepared according to the typical procedure H from *tert*-butyl 3-hydroxy-4-nitrobenzoate (2.50 g, 10.45 mmol) and 2-propanol (0.88 mL, 11.50 mmol); white solid (2.67 g, 91% yield). ^1^H NMR (400 MHz, DMSO-*d*_6_): δ 7.93 (d, *J* = 8.3 Hz, 1H), 7.73 (d, *J* = 1.6 Hz, 1H), 7.57 (dd, *J* = 8.3 Hz, 1.6 Hz, 1H), 4.90 (hept, *J* = 6.0 Hz, 1H), 1.57 (s, 9H), 1.31 (d, *J* = 6.0 Hz, 6H).

#### 4.2.163 *tert*-Butyl 4-Amino-3-isopropoxybenzoate (**4s46**)

Prepared according to the typical procedure I from **3s46** (2.66 g, 9.44 mmol); white crystals (1.78 g, 75% yield). ^1^H NMR (400 MHz, DMSO-*d*_6_) δ 7.30 (dd, *J* = 8.2 Hz, 1.8 Hz, 1H), 7.26 (d, *J* = 1.8 Hz, 1H), 6.62 (d, *J* = 8.2 Hz, 1H), 5.46 (s, 2H), 4.50 (hept, *J* = 6.1 Hz, 1H), 1.50 (s, 9H), 1.27 (d, *J* = 6.1 Hz, 6H). MS (ESI): *m*/*z* calcd for C_14_H_21_NO_3_: 251.15. Found: 250.2 [M – H]^−^, 252.2 [M + H]^+^.

#### 4.2.164 *tert*-Butyl 2-Amino-4-isopropoxybenzo[*d*]thiazole-6-carboxylate (**5s46**)

Prepared according to the typical procedure C from aniline **4s46** (1.47 g, 5.84 mmol); light yellow solid (0.99 g, 55% yield). ^1^H NMR (400 MHz, DMSO-*d*_6_): δ 7.87–7.83 (m, 3H), 7.29 (d, *J* = 1.5 Hz, 1H), 4.81 (hept, *J* = 6.1 Hz, 1H), 1.54 (s, 9H), 1.28 (d, *J* = 6.1 Hz, 6H); MS (ESI): *m*/*z* calcd for C_15_H_20_N_2_O_3_S: 308.12. Found: 309.3 [M + H]^+^.

#### 4.2.165 *tert*-Butyl 2-(3,4-Dichloro-5-methyl-1*H*-pyrrole-2-carboxamido)-4-isopropoxybenzo[*d*]thiazole-6-carboxylate (**6s46**)

Prepared according to the typical procedure D from 2-aminobenzothiazole **5s46** (254 mg, 0.825 mmol); gray solid (40 mg, 10% yield). ^1^H NMR (400 MHz, DMSO-*d*_6_): δ 12.26 (s, 1H), 12.20 (s, 1H), 8.15 (s, 1H), 7.45 (s, 1H), 4.97–4.83 (m, 1H), 2.27 (s, 3H), 1.58 (s, 9H), 1.36 (d, *J* = 6.0 Hz, 6H).

#### 4.2.166 2-(3,4-Dichloro-5-methyl-1*H*-pyrrole-2-carboxamido)-4-isopropoxybenzo[*d*]thiazole-6-carboxylic Acid (**46**)

A solution of the *tert*-Bu ester **6s46** (40 mg, 0.083 mmol) in DCM (3.0 mL) and CF_3_COOH (0.1 mL) was stirred at room temperature for 9 h (97% conversion by ^1^H NMR). Additional CF_3_COOH (0.1 mL) was added and stirred at 22 °C for a further 3.5 h. The reaction mixture was concentrated under reduced pressure, and the solid residue was washed with Et_2_O (2 × 5 mL) and filtered. The solid residue was washed with MeOH (3 mL) and air-dried. The title compound was obtained as an off-white solid (24 mg, 68% yield). ^1^H NMR (400 MHz, DMSO-*d*_6_) δ 12.99 (s, 1H), 12.26 (s, 1H), 12.19 (s, 1H), 8.20 (s, 1H), 7.49 (s, 1H), 4.96–4.83 (m, 1H), 2.27 (s, 3H), 1.36 (d, *J* = 6.0 Hz, 6H); HRMS (ESI): *m*/*z* calcd for C_17_H_14_Cl_2_N_3_O_4_S: 426.0082. Found 426.0089 [M – H]^−^; HPLC purity (254 nm): 97%.

#### 4.2.167 *tert*-Butyl 3-((3-Methylbutan-2-yl)oxy)-4-nitrobenzoate (**3s47**)

Prepared according to the typical procedure H from *tert*-butyl 3-hydroxy-4-nitrobenzoate (2.50 g, 10.50 mmol) and *sec*-iso-amyl alcohol (1.01 g, 11.50 mmol); white solid (1.68 g, 52% yield). ^1^H NMR (400 MHz, CDCl_3_): δ 7.74 (d, *J* = 8.3 Hz, 1H), 7.68 (d, *J* = 1.5 Hz, 1H), 7.55 (dd, *J* = 8.3 Hz, 1.5 Hz, 1H), 4.44–4.36 (m, 1H), 2.06–1.90 (m, 1H), 1.31 (d, *J* = 6.2 Hz, 3H), 1.01 (d, *J* = 6.8 Hz, 3H), 0.99 (d, *J* = 6.8 Hz, 3H).

#### 4.2.168 *tert*-Butyl 4-Amino-3-((3-methylbutan-2-yl)oxy)benzoate (**4s47**)

Prepared according to the typical procedure I from **3s47** (1.70 g, 5.50 mmol); colorless oil, which solidified upon standing (1.46 g, 95% yield). ^1^H NMR (400 MHz, CDCl_3_): δ 7.45 (dd, *J* = 8.1 Hz, 1.7 Hz, 1H), 7.42 (d, *J* = 1.7 Hz, 1H), 6.64 (d, *J* = 8.1 Hz, 1H), 4.26 (p, *J* = 6.0 Hz, 1H), 4.16 (s, 2H), 2.05–1.88 (m, 1H), 1.25 (d, *J* = 6.2 Hz, 3H), 1.01 (d, *J* = 6.8 Hz, 3H), 0.98 (d, *J* = 6.8 Hz, 3H).

#### 4.2.169 *tert*-Butyl 2-Amino-4-((3-methylbutan-2-yl)oxy)benzo[*d*]-thiazole-6-carboxylate (**5s47**)

Prepared according to the typical procedure C from aniline **4s47** (1.46 g, 5.22 mmol); light yellow solid (0.65 g, 37% yield). ^1^H NMR (400 MHz, DMSO-*d*_6_): δ 7.86–7.81 (m, 3H), 7.29 (d, *J* = 1.4 Hz, 1H), 4.44 (p, *J* = 6.1 Hz, 1H), 1.91 (dq, *J* = 13.5 Hz, 6.7 Hz, 1H), 1.54 (s, 9H), 1.19 (d, *J* = 6.2 Hz, 3H), 0.96 (dd, *J* = 9.3 Hz, 6.8 Hz, 6H); MS (ESI): *m*/*z* calcd for C_17_H_24_N_2_O_3_S: 336.15. Found: 335.4 [M – H]^−^.

#### 4.2.170 *tert*-Butyl 2-(3,4-Dichloro-5-methyl-1*H*-pyrrole-2-carboxamido)-4-((3-methylbutan-2-yl)oxy)benzo[*d*]thiazole-6-carboxylate (**6s47**)

Prepared according to the typical procedure D from 5s47 (248 mg, 0.736 mmol); gray solid (200 mg, 53% yield). ^1^H NMR (400 MHz, DMSO-*d*_6_) δ 12.28 (s, 1H), 12.18 (s, 1H), 8.14 (s, 1H), 7.44 (s, 1H), 4.52 (p, *J* = 5.9 Hz, 1H), 2.27 (s, 3H), 1.99 (dq, *J* = 13.3 Hz, 6.8 Hz, 1H), 1.57 (s, 9H), 1.27 (d, *J* = 6.1 Hz, 3H), 0.99 (dd, *J* = 15.0 Hz, 6.8 Hz, 6H); MS (ESI): *m*/*z* calcd for C_23_H_27_Cl_2_N_3_O_4_S: 511.11. Found: 512.4 [M + H]^+^.

#### 4.2.171 2-(3,4-Dichloro-5-methyl-1*H*-pyrrole-2-carboxamido)-4-((3-methylbutan-2-yl)oxy)benzo[*d*]thiazole-6-carboxylic Acid (**47**)

A solution of the *tert*-butyl ester **6s47** (50 mg, 0.098 mmol) and CF_3_COOH (0.1 mL) in dichloromethane (3 mL) was stirred at 22 °C for 5.5 h (55% conversion by ^1^H NMR). An additional CF_3_COOH (0.1 mL) was added, and the mixture was stirred at 22 °C for a further 24 h. The reaction mixture was concentrated under reduced pressure, and the solid residue was dispersed in MeOH (5 mL), filtered, washed with Et_2_O (3 mL), and air-dried to give an off-white solid (23 mg, 52% yield). ^1^H NMR (400 MHz, DMSO-*d*_6_): δ 12.97 (s, 1H), 12.27 (s, 1H), 12.17 (s, 1H), 8.19 (s, 1H), 7.48 (s, 1H), 4.56–4.47 (m, 1H), 2.27 (s, 3H), 2.02–1.95 (m, 1H), 1.27 (d, *J* = 6.1 Hz, 3H), 1.01 (*J* = 6.8 Hz, 3H), 0.98 (*J* = 6.7 hZ, 3H); ^13^C NMR (101 MHz, DMSO-*d*_6_) (representative signals): δ 167.10, 149.49, 133.04, 129.83, 126.75, 116.87, 115.71, 115.58, 109.95, 109.76, 78.48, 32.38, 18.60, 17.45, 15.89, 11.05; HRMS (ESI) *m*/*z*: [M – H]^−^ calcd for C_19_H_20_Cl_2_N_3_O_4_S 456.0552; found 456.0536; HPLC purity (254 nm): 99%.

#### 4.2.172 Methyl 3-(1-Cyclopropylethoxy)-4-nitrobenzoate (**3s48**)

Prepared according to the typical procedure H from **2s** (1.97 g, 10.00 mmol) and 1-cyclopropylethanol (947 mg, 11.00 mmol); white solid (2.39 g, 90% yield). ^1^H NMR (400 MHz, CDCl_3_): δ 7.76 (d, *J* = 8.4 Hz, 1H), 7.73 (d, *J* = 1.5 Hz, 1H), 7.66 (dd, *J* = 8.4 Hz, 1.5 Hz, 1H), 4.13–4.06 (m, 1H), 3.96 (s, 3H), 1.45 (d, *J* = 6.2 Hz, 3H), 1.21–1.11 (m, 1H), 0.62–0.57 (m, 1H), 0.57–0.52 (m, 1H), 0.43–0.34 (m, 1H), 0.34–0.25 (m, 1H).

#### 4.2.173 Methyl 4-Amino-3-(1-cyclopropylethoxy)benzoate (**4s48**)

Prepared according to the typical procedure I from **3s48** (2.21 g, 8.33 mmol); white solid (1.78 g, 91% yield). ^1^H NMR (400 MHz, DMSO-*d*_6_): δ 7.41–7.33 (m, 1H), 7.34–7.27 (m, 1H), 6.72–6.60 (m, 1H), 5.54 (s, 2H), 3.95–3.79 (m, 1H), 3.74 (s, 1H), 1.35–1.22 (m, 3H), 1.18–1.01 (m, 1H), 0.53–0.36 (m, 2H), 0.38–0.21 (m, 2H).

#### 4.2.174 Methyl 2-Amino-4-(1-cyclopropylethoxy)benzo[*d*]thiazole-6-carboxylate (**5s48**)

Prepared according to the typical procedure C from aniline **4s48** (1.06 g, 4.50 mmol); light yellow powder (724 mg, 55% yield). ^1^H NMR (400 MHz, DMSO-*d*_6_) δ 7.92 (d, *J* = 1.2 Hz, 1H), 7.86 (s, 2H), 7.33 (d, *J* = 1.2 Hz, 1H), 4.19–4.10 (m, 1H), 3.82 (s, 3H), 1.30 (d, *J* = 6.1 Hz, 3H), 1.14–1.04 (m, 1H), 0.52–0.40 (m, 2H), 0.35–0.24 (m, 2H); ^13^C NMR (101 MHz, DMSO-*d*_6_): δ 168.36, 166.13, 147.78, 147.17, 132.15, 122.21, 115.73, 112.75, 78.23, 51.93, 19.53, 16.76, 3.82, 1.60.

#### 4.2.175 Methyl 4-(1-Cyclopropylethoxy)-2-(3,4-dichloro-5-methyl-1*H*-pyrrole-2-carboxamido)benzo[*d*]thiazole-6-carboxylate (**6s48**)

Prepared according to the typical procedure D from 2-aminobenzothiazole **5s48** (250 mg, 0.854 mmol); white solid (40 mg, 10% yield). ^1^H NMR (400 MHz, DMSO-*d*_6_): δ 12.25 (s, 1H), 12.22 (s, 1H), 8.23 (s, 1H), 7.47 (s, 1H), 4.29–4.15 (m, 1H), 3.87 (s, 3H), 2.27 (s, 3H), 1.38 (d, *J* = 6.1 Hz, 3H), 1.22–1.11 (m, 1H), 0.58–0.46 (m, 2H), 0.43–0.31 (m, 2H).

#### 4.2.176 4-(1-Cyclopropylethoxy)-2-(3,4-dichloro-5-methyl-1*H*-pyrrole-2-carboxamido)benzo[*d*]thiazole-6-carboxylic Acid (**48**)

Prepared according to the typical procedure E from methyl ester **6s48** (33 mg, 0.070 mmol); brown powder (15 mg, 47% yield). ^1^H NMR (400 MHz, DMSO-*d*_6_): δ 12.94 (s, 1H), 12.25 (s, 1H), 12.19 (s, 1H), 8.18 (s, 1H), 7.47 (s, 1H), 4.33–4.11 (m, 1H), 2.28 (s, 3H), 1.49–1.31 (m, 3H), 1.24–1.07 (m, 1H), 0.63–0.46 (m, 2H), 0.44–0.26 (m, 2H); HRMS (ESI) *m*/*z*: [M + H]^+^ calcd for C_19_H_18_Cl_2_N_3_O_4_S 454.0395; found 454.0380; HPLC purity (254 nm): 94%.

#### 4.2.177 Methyl 4-Nitro-3-((tetrahydrofuran-3-yl)oxy)benzoate (**3s49**)

Prepared following the typical procedure H from **2s** (2.11 g, 10.70 mmol) and 3-hydroxytetrahydrofuran (1.04 g, 11.77 mmol); white solid (2.37 g, 83% yield). ^1^H NMR (400 MHz, DMSO-*d*_6_): δ 8.00 (d, *J* = 8.4 Hz, 1H), 7.76 (d, *J* = 1.6 Hz, 1H), 7.68 (dd, *J* = 8.3, 1.6 Hz, 1H), 5.43–5.35 (m, 1H), 3.96–3.87 (m, 4H), 3.87–3.73 (m, 3H), 2.32–2.19 (m, 1H), 2.06–1.94 (m, 1H).

#### 4.2.178 Methyl 4-Amino-3-((tetrahydrofuran-3-yl)oxy)benzoate (**4s49**)

Prepared according to the typical procedure I from **3s49** (2.09 g, 7.81 mmol); white solid (1.00 g, 54% yield). ^1^H NMR (400 MHz, DMSO-*d*_6_): δ 7.38 (dd, *J* = 8.2 Hz, 1.8 Hz, 1H), 7.24 (d, *J* = 1.8 Hz, 1H), 6.65 (d, *J* = 8.2 Hz, 1H), 5.63 (s, 2H), 5.07–4.93 (m, 1H), 3.92–3.75 (m, 4H), 3.75 (s, 3H), 2.25–2.13 (m, 1H), 2.06–1.97 (m, 1H); MS (ESI): *m*/*z* calcd for C_12_H_15_NO_4_: 237.10. Found: 238.2 [M + H]^+^.

#### 4.2.179 Methyl 2-Amino-4-((tetrahydrofuran-3-yl)oxy)benzo[*d*]-thiazole-6-carboxylate (**5s49**)

Prepared according to the typical procedure C from aniline **4s49** (765 mg, 2.60 mmol); light yellow solid (660 mg, 54% yield). ^1^H NMR (400 MHz, DMSO-*d*_6_): δ 7.97 (d, *J* = 1.5 Hz, 1H), 7.93 (s, 2H), 7.31 (d, *J* = 1.5 Hz, 1H), 5.30–5.22 (m, 1H), 3.91–3.84 (m, 3H), 3.83 (s, 3H), 3.81–3.75 (m, 1H), 2.24–2.13 (m, 1H), 2.05–1.97 (m, 1H); MS (ESI): *m*/*z* calcd for C_13_H_14_N_2_O_4_S: 294.07. Found: 295.2 [M + H]^+^.

#### 4.2.180 Methyl 2-(3,4-Dichloro-5-methyl-1*H*-pyrrole-2-carboxamido)-4-((tetrahydrofuran-3-yl)oxy)benzo[*d*]thiazole-6-carboxylate (**6s49**)

Prepared according to the typical procedure D from 2-aminobenzothiazole 5s49 (250 mg, 0.850 mmol); gray powder (296 mg, 74% yield). ^1^H NMR (400 MHz, DMSO-*d*_6_): δ 12.23 (s, 2H), 8.27 (s, 1H), 7.46 (s, 1H), 5.39–5.28 (m, 1H), 3.99–3.91 (m, 2H), 3.88 (s, 3H), 3.85–3.79 (m, 2H), 2.34–2.28 (m, 1H), 2.27 (s, 3H), 2.12–2.03 (m, 1H); MS (ESI): *m*/*z* calcd for C_19_H_17_Cl_2_N_3_O_5_S: 469.07. Found: 470.3 [M + H]^+^.

#### 4.2.181 2-(3,4-Dichloro-5-methyl-1*H*-pyrrole-2-carboxamido)-4-((tetrahydrofuran-3-yl)oxy)benzo[*d*]thiazole-6-carboxylic Acid (**49**)

Prepared according to the typical procedure E from methyl ester **6s49** (153 mg, 0.325 mmol); brown solid (89 mg, 60% yield). ^1^H NMR (400 MHz, DMSO-*d*_6_): δ 12.92 (s, 1H), 12.23 (s, 2H), 8.23 (s, 1H), 7.45 (s, 1H), 5.32 (s, 1H), 4.06–3.69 (m, 4H), 2.27 (s, 4H), 2.16–1.96 (m, 1H). ^13^C NMR (101 MHz, DMSO-d_6_): δ = 167.05, 159.69, 156.66, 156.64, 148.78, 133.06, 129.97, 126.70, 116.87, 116.28, 115.79, 110.01, 109.68, 78.06, 72.34, 66.50, 39.52, 32.36, 11.07; HRMS (ESI) *m*/*z*: [M + H]^+^ calcd for C_18_H_16_Cl_2_N_3_O_5_S 456.0188; found 456.0182; HPLC purity (254 nm): 97%.

#### 4.2.182 Methyl 2-(4-Chloro-5-methyl-1*H*-pyrrole-2-carboxamido)-4-(1-phenylethoxy)benzo[*d*]thiazole-6-carboxylate (**38s50**)

Prepared according to the general procedure J from methyl 2-amino-4-(1-phenylethoxy)benzo[*d*]thiazole-6-carboxylate (**5s27**) (131 mg, 0.400 mmol) and 2-trichloroacetyl-4-chloro-5-methyl-1*H*-pyrrole (**34s50**) (104 mg, 0.400 mmol); white solid (141 mg, 75% yield). ^1^H NMR (400 MHz, DMSO-*d*_6_): δ 12.84 (s, 1H), 12.18 (s, 1H), 8.14 (s, 1H), 7.49–7.42 (m, 3H), 7.40–7.32 (m, 3H), 7.26 (t, *J* = 7.1 Hz, 1H), 5.81 (q, *J* = 6.2 Hz, 1H), 3.80 (s, 3H), 2.22 (s, 3H), 1.65 (d, *J* = 6.2 Hz, 3H); MS (ESI): *m*/*z* calcd for C_23_H_20_ClN_3_O_4_S: 469.09. Found: 470.2 [M + H]^+^.

#### 4.2.183 2-(4-Chloro-5-methyl-1*H*-pyrrole-2-carboxamido)-4-(1-ph-enylethoxy)benzo[*d*]thiazole-6-carboxylic Acid (**50**)

Prepared according to the typical procedure E from methyl ester **38s50** (100 mg, 0.213 mmol); white solid (83 mg, 86% yield). ^1^H NMR (400 MHz, DMSO-*d*_6_): δ 12.82 (s, 2H), 12.20 (s, 1H), 8.12 (d, *J* = 0.9 Hz, 1H), 7.49–7.42 (m, 3H), 7.40–7.33 (m, 3H), 7.26 (t, *J* = 7.3 Hz, 1H), 5.79 (q, *J* = 6.2 Hz, 1H), 2.23 (s, 3H), 1.65 (d, *J* = 6.2 Hz, 3H); ^13^C NMR (101 MHz, DMSO-*d*_6_): δ 166.95, 160.15, 158.01, 149.03, 142.58, 142.37, 132.87, 131.67, 128.63, 127.55, 126.25, 125.59, 125.52, 120.73, 115.76, 113.24, 110.23, 109.75, 75.42, 24.40, 10.34; HRMS (ESI) *m*/*z*: [M + H]^+^ calcd for C_22_H_19_ClN_3_O_4_S 456.0785; found 456.0771; HPLC purity (254 nm): 96%.

#### 4.2.184 Methyl 2-(4-Fluoro-5-methyl-1*H*-pyrrole-2-carboxamido)-4-(1-phenylethoxy)benzo[*d*]thiazole-6-carboxylate (**39s51**)

Oxalyl chloride (0.42 mL, 4.88 mmol) was added to a suspension of 4-fluoro-5-methyl-1*H*-pyrrole-2-carboxylic acid (**35s51)** (70 mg, 0.489 mmol) in anhydrous dichloromethane (5 mL), and the mixture was stirred at 22 °C overnight. The resulting light brown solution was concentrated under reduced pressure, and the reaction vessel was backfilled with argon to obtain the corresponding acyl chloride as a light brown powder. Methyl 2-amino-4-(1-phenylethoxy)benzo[*d*]-thiazole-6-carboxylate (**5s27**) (161 mg, 0.489 mmol) and normal grade toluene (10.5 mL) were added, and the resulting suspension equipped with a CaCl_2_ tube was refluxed (oil bath 130 °C) overnight. The reaction mixture was cooled to 22 °C, and the precipitate was collected and washed with toluene. The product was obtained after trituration with cold MeOH as a light brown powder (136 mg, 57% yield). It contained 2–3 mol % (by ^1^H NMR) of the 4-OH impurity, resulting from acidolytic cleavage of the phenethyl ether. ^1^H NMR (400 MHz, DMSO-*d*_6_): δ 12.74 (s, 1H), 11.85 (s, 1H), 8.15 (d, *J* = 1.4 Hz, 1H), 7.47–7.42 (m, 2H), 7.38–7.32 (m, 3H), 7.30–7.23 (m, 2H), 5.79 (q, *J* = 6.3 Hz, 1H), 3.80 (s, 3H), 2.20 (s, 3H), 1.64 (d, *J* = 6.3 Hz, 3H); ^19^F NMR (376 MHz, DMSO-*d*_6_) δ −166.53; ^13^C NMR (101 MHz, DMSO-*d*_6_): δ 165.90, 160.57, 158.45 (d, *J* = 3.2 Hz), 149.17, 149.09, 146.82, 133.04, 128.67, 127.62, 125.57, 124.90, 119.14 (d, *J* = 25.2 Hz), 116.70 (d, *J* = 7.0 Hz), 115.74, 110.06, 100.84 (d, *J* = 15.8 Hz), 75.54, 52.16, 24.36, 8.83 (d, *J* = 2.2 Hz).

#### 4.2.185 2-(4-Fluoro-5-methyl-1*H*-pyrrole-2-carboxamido)-4-(1-phenylethoxy)benzo[*d*]thiazole-6-carboxylic Acid (**51**)

Prepared according to the typical procedure E from methyl ester **39s51** (101 mg, 0.222 mmol); light brown solid (80 mg, 82% yield). ^1^H NMR (400 MHz, DMSO-*d*_6_): δ 12.79 (s, 1H), 12.72 (s, 1H), 11.85 (s, 1H), 8.11 (s, 1H), 7.45 (d, *J* = 7.5 Hz, 2H), 7.40–7.31 (m, 3H), 7.32–7.21 (m, 2H), 5.78 (q, *J* = 6.3 Hz, 1H), 2.20 (s, 3H), 1.64 (d, *J* = 6.3 Hz, 3H); ^19^F NMR (376 MHz, DMSO-*d*_6_): δ −166.57; ^13^C NMR (101 MHz, DMSO-*d*_6_): δ 167.45, 160.69, 158.91, 149.49, 147.26, 143.07, 142.89, 133.35, 129.11 (2C), 128.03, 126.68, 126.00 (2C), 119.53 (d, *J* = 25 Hz), 117.18 (d, *J* = 7.1 Hz), 116.24, 110.72, 101.25 (d, *J* = 16 Hz), 75.89, 24.88, 9.29; HRMS (ESI) *m*/*z*: [M – H]^−^ calcd for C_22_H_17_FN_3_O_4_S 438.0924. Found 438.09335; HPLC purity (254 nm): 95%.

#### 4.2.186 Methyl (*S*)-2-(4-Fluoro-5-methyl-1*H*-pyrrole-2-carboxamido)-4-(1-phenylethoxy)benzo[*d*]thiazole-6-carboxylate (**39s*****S*****51**)

Prepared from 24s*S*27 (173 mg, 0.526 mmol) and 35s51 (75 mg, 0.526 mmol) as described above for the synthesis of 39s51; light brown powder (136 mg, 57% yield). ^1^H NMR and MS data were identical to those of the racemic product.

#### 4.2.187 (*S*)-2-(4-Fluoro-5-methyl-1*H*-pyrrole-2-carboxamido)-4-(1-phenylethoxy)benzo[*d*]thiazole-6-carboxylic Acid [**(*****S*****)-51**]

Prepared according to the typical procedure E from methyl ester **39s*****S*****51** (100 mg, 0.221 mmol); brown solid (80 mg, 83% yield). ^1^H NMR and MS data were identical to those of the racemic product; HPLC purity (254 nm): 93% (sum of enantiomers); 82% ee (determined by chiral HPLC analysis on a Kromasil 3-CelluCoat column (4.6 mm × 150 mm)), eluent hexane/MeOH/0.1%TFA in *i*-PrOH = 90:5:5; *t*_R_ = 5.4 min (*S* enantiomer), 6.3 min (*R* enantiomer). The absolute configuration of the major product was assigned based on the starting (*R*)-1-phenylethanol.

#### 4.2.188 Methyl (*R*)-2-(4-Fluoro-5-methyl-1*H*-pyrrole-2-carboxamido)-4-(1-phenylethoxy)benzo[*d*]thiazole-6-carboxylate (**39s*****R*****51**)

Prepared from **24s*R*27** (158 mg, 0.481 mmol) and **35s51** (69 mg, 0.481 mmol) as described above for the synthesis of **39s51**; white solid (72 mg, 33% yield). ^1^H NMR and MS data were identical to those of the racemic product. HPLC purity (254 nm): 97.3%.

#### 4.2.189 (*R*)-2-(4-Fluoro-5-methyl-1*H*-pyrrole-2-carboxamido)-4-(1-phenylethoxy)benzo[*d*]thiazole-6-carboxylic Acid [**(*****R*****)-51**]

Prepared according to the typical procedure E from methyl ester **39s*****R*****51** (60 mg, 0.132 mmol); off-white solid (45 mg, 78% yield). ^1^H NMR and MS data were identical to those of the racemic product. HPLC purity (254 nm): 96.4% (sum of enantiomers); >95% ee (determined by chiral HPLC analysis on a Kromasil 3-CelluCoat column (4.6 mm × 150 mm)), eluent hexane/MeOH/0.1% TFA in *i*-PrOH = 90:5:5; *t*_R_ = 5.4 min (*S* enantiomer), 6.3 min (*R* enantiomer). The absolute configuration of the major product was assigned based on the starting (*S*)-1-phenylethanol.

#### 4.2.190 Methyl 2-(4-Cyano-5-methyl-1*H*-pyrrole-2-carboxamido)-4-(1-phenylethoxy) benzo[*d*]thiazole-6-carboxylate (**39s52**)

4-Cyano-5-methyl-1*H*-pyrrole-2-carboxylic acid (**35s52**) (64 mg, 0.43 mmol) was suspended in SOCl_2_ (1.55 mL, 21.3 mmol). The mixture was stirred at 22 °C overnight and concentrated under reduced pressure. Compound **5s27** (70 mg, 0.213 mmol) and toluene (4 mL) were added, and the mixture was stirred at 130 °C for 3 h. The precipitate was collected and purified by column chromatography, eluent DCM/THF = 10:1 to get product **39s52** as a beige solid (59 mg, 60% yield). ^1^H NMR (400 MHz, DMSO-*d*_6_): δ 13.08 (s, 1H), 12.76 (s, 1H), 7.50–7.42 (m, 2H), 7.41–7.31 (m, 3H), 7.30–7.23 (m, 1H), 5.80 (q, *J* = 6 Hz, 1H), 3.80 (s, 3H), 2.40 (s, 3H), 1.65 (d, *J* = 6 Hz, 3H); MS (ESI): *m*/*z* calcd for C_24_H_20_N_4_O_4_S: 460.12. Found: 459.1 [M – H]^−^.

#### 4.2.191 2-(4-Cyano-5-methyl-1*H*-pyrrole-2-carboxamido)-4-(1-phenylethoxy)benzo[*d*]thiazole-6-carboxylic Acid (**52**)

Prepared according to the typical procedure E from methyl ester **39s52** (44 mg, 0.096 mmol): white solid (30 mg, 70% yield). ^1^H NMR (400 MHz, DMSO-*d*_6_) δ: 13.05 (s, 1H), 12.84 (s, 1H), 12.76 (s, 1H), 8.14 (d, *J* = 1.4 Hz, 1H), 7.74 (d, *J* = 2.3 Hz, 1H), 7.48–7.42 (m, 2H), 7.40–7.31 (m, 3H), 7.29–7.22 (m, 1H), 5.79 (q, *J* = 6.3 Hz, 1H), 2.40 (s, 3H), 1.65 (d, *J* = 6.3 Hz, 3H); HRMS (ESI) *m*/*z*: [M + H]^+^ calcd for C_23_H_19_N_4_O_4_S 447.1127; found 447.1133; HPLC purity (254 nm): 97%.

#### 4.2.192 Methyl 2-(5-Methyl-1*H*-pyrrole-2-carboxamido)-4-(1-phenylethoxy)benzo[*d*]thiazole-6-carboxylate (**38s53**)

Prepared according to the typical procedure J from **5s27** (86 mg, 0.261 mmol) and 2-trichloroacetyl-5-methyl-1*H*-pyrrole (**34s53**) (59 mg, 0.261 mmol); white solid (100 mg, 88% yield). ^1^H NMR (400 MHz, DMSO-*d*_6_): δ 12.67 (s, 1H), 11.75 (s, 1H), 8.14 (d, *J* = 1.2 Hz, 1H), 7.45 (d, *J* = 7.3 Hz, 2H), 7.41–7.32 (m, 4H), 7.26 (t, *J* = 7.3 Hz, 1H), 6.00–5.94 (m, 1H), 5.80 (q, *J* = 6.2 Hz, 1H), 3.80 (s, 3H), 2.26 (s, 3H), 1.65 (d, *J* = 6.2 Hz, 3H); ^13^C NMR (101 MHz, DMSO-*d*_6_): δ 165.93, 160.80, 158.47, 149.02, 142.84, 142.53, 135.73, 133.04, 128.66, 127.61, 125.57, 124.73, 122.12, 115.72, 115.59, 110.09, 108.82, 75.52, 52.15, 24.36, 12.77; MS (ESI): *m*/*z* calcd for C_23_H_21_N_3_O_4_S: 435.13. Found: 434.5 [M – H]^−^.

#### 4.2.193 2-(5-Methyl-1*H*-pyrrole-2-carboxamido)-4-(1-phenylethoxy)benzo[*d*]thiazole-6-carboxylic Acid (**53**)

Prepared according to the typical procedure E from methyl ester **38s53** (85 mg, 0.195 mmol); beige solid (73 mg, 89% yield). ^1^H NMR (400 MHz, DMSO-*d*_6_): δ 12.79 (s, 1H), 12.64 (s, 1H), 11.75 (s, 1H), 8.10 (s, 1H), 7.55–7.21 (m, 7H), 5.96 (s, 1H), 5.78 (q, *J* = 6 Hz, 1H), 2.26 (s, 3H), 1.64 (d, *J* = 6 Hz, 3H); ^13^C NMR (101 MHz, DMSO-*d*_6_): δ 167.00, 160.44, 158.47, 148.94, 142.63, 142.51, 135.65, 132.87, 128.63, 127.54, 126.03, 125.53, 122.15, 115.73, 115.53, 110.26, 108.78, 75.41, 24.41, 12.75; HRMS (ESI) *m*/*z*: [M + H]^+^ calcd for C_22_H_20_N_3_O_4_S 422.1175; found 422.1161; HPLC purity (254 nm): 98%.

#### 4.2.194 Methyl 2-(3-Fluoro-5-methyl-1*H*-pyrrole-2-carboxamido)-4-(1-phenylethoxy)benzo[*d*]thiazole-6-carboxylate (**39s54**)

A suspension of 3-fluoro-5-methyl-1*H*-pyrrole-2-carbonyl chloride (56 mg, 0.347 mmol), prepared from 3-fluoro-1*H*-pyrrole-2-carboxylic acid (**35s54**) and oxalyl chloride as described above, and **5s27** (114 mg, 0.347 mmol) in toluene (7 mL) was equipped with a CaCl_2_ tube and refluxed overnight. After cooling to 22 °C, the precipitate was collected and washed with toluene to obtain the title compound as a light gray powder (91 mg, 57% yield). ^1^H NMR (400 MHz, DMSO-*d*_6_): δ 12.23 (s, 1H), 11.37 (s, 1H), 8.17 (d, *J* = 1.5 Hz, 1H), 7.49–7.42 (m, 2H), 7.40–7.31 (m, 3H), 7.29–7.25 (m, 1H), 6.91 (app t, *J* = 4.2 Hz, 1H), 5.80 (q, *J* = 6.2 Hz, 1H), 3.80 (s, 3H), 1.99 (s, 3H), 1.64 (d, *J* = 6.2 Hz, 3H); ^19^F NMR (376 MHz, DMSO-*d*_6_): δ −153.97 (d, *J* = 4 Hz); ^13^C NMR (101 MHz, DMSO-*d*_6_) δ 165.87, 160.07, 156.71, 154.47, 151.93, 149.08, 142.67, 142.50, 133.03, 128.64, 128.54 (d, *J* = 70.2 Hz), 127.61, 125.58, 125.01, 121.03 (d, *J* = 6.0 Hz), 115.90, 110.32, 107.78 (d, *J* = 17.0 Hz), 106.23 (d, *J* = 13.5 Hz), 75.62, 52.16, 24.28, 7.19; HRMS (ESI) *m*/*z*: [M – H]^−^ calcd for C_23_H_19_FN_3_O_4_S 452.1080; found 452.1092.

#### 4.2.195 2-(3-Fluoro-5-methyl-1*H*-pyrrole-2-carboxamido)-4-(1-phenylethoxy)benzo[*d*]thiazole-6-carboxylic Acid (**54**)

A solution of methyl ester **39s54** (70 mg, 0.154 mmol) and 2 M NaOH (0.40 mL) in MeOH (3.0 mL) was stirred at 40 °C overnight. NaOH (2 M, 0.40 mL) was added, and the mixture was stirred for another night and concentrated. The residue was suspended in water (2 mL), pH was adjusted to 2 by adding 4 M HCl, the precipitate was collected, washed with water, air-dried, and triturated with MeOH to get the title compound as a beige solid (55 mg, 81% yield). ^1^H NMR (400 MHz, DMSO-*d*_6_): δ 12.82 (s, 1H), 12.20 (s, 1H), 11.37 (s, 1H), 8.13 (s, 1H), 7.52–7.40 (m, 2H), 7.40–7.31 (m, 3H), 7.31–7.20 (m, 1H), 6.91 (app t, *J* = 4.2 Hz, 1H), 5.79 (q, *J* = 6.3 Hz, 1H), 1.99 (s, 3H), 1.64 (d, *J* = 6.3 Hz, 3H); ^19^F NMR (376 MHz, DMSO-*d*_6_): δ −154.06 (d, *J* = 4 Hz); ^13^C NMR (101 MHz, DMSO-*d*_6_): δ 166.92, 159.75, 156.78, 154.36, 151.82, 148.98, 142.57, 142.28, 132.83, 128.61, 127.55, 126.31, 125.53, 120.98 (d, *J* = 5.3 Hz), 115.90, 110.44, 107.83 (d, *J* = 15 Hz), 106.18 (d, *J* = 14 Hz), 75.48, 24.33, 7.17; HRMS (ESI) *m*/*z*: [M – H]^−^ calcd for C_22_H_17_FN_3_O_4_FS 438.0924; found 438.0934; HPLC purity (254 nm): 94%.

#### 4.2.196 Methyl 2-(3,4-Dichloro-5-(phthalimidomethyl)-1*H*-pyrrole-2-carboxamido)-4-(1-phenylethoxy)benzo[*d*]thiazole-6-carboxylate (**40s55**)

A suspension of 3,4-dichloro-5-(phthalimidomethyl)-1*H*-pyrrole-2-carboxylic acid (**36s55**)^36^ (155 mg, 0.457 mmol) in SOCl_2_ (2 mL) was refluxed for 1 h and then concentrated under reduced pressure. To the solid residue were added **5s27** (150 mg, 0.457 mmol) and toluene (9 mL), and the resulting suspension was refluxed overnight. Upon cooling, the precipitate was collected and washed with MeOH to get the crude product (200 mg), containing 10 mol % of an *O*-dealkylated impurity. After purification with flash chromatography on silica (20 mL), eluent dichloromethane/THF = 20:1, dry-loading with THF, the title compound was obtained as a white solid (126 mg, 42% yield). ^1^H NMR (400 MHz, DMSO-*d*_6_): δ 12.57 (s, 1H), 12.41 (s, 1H), 8.18 (s, 1H), 7.970 7.92 (m, 2H), 7.91–7.86 (m, 2H), 7.44 (d, *J* = 7.3 Hz, 2H), 7.39–7.31 (m, 3H), 7.25 (app t, *J* = 7.3 Hz, 1H), 5.79 (q, *J* = 6.4 Hz, 1H), 4.84 (s, 2H), 3.80 (s, 3H), 1.62 (d, *J* = 6.4 Hz, 3H).

#### 4.2.197 (3,4-Dichloro-5-((6-(methoxycarbonyl)-4-(1-phenylethoxy)-benzo[*d*]thiazol-2-yl)carbamoyl)-1*H*-pyrrol-2-yl)methanaminium 1,4-Dioxo-3,4-dihydro-1*H*-phthalazin-2-ide (**41s55**)

Hydrazine hydrate (80%, 0.1 mL, 10 equiv) was added to 40s55 (100 mg, 0.161 mg) in abs. EtOH (3.5 mL), and the suspension was stirred at 40 °C for 40 min. The reaction mixture was concentrated on a water pump, suspended in MeOH, 37% HCl(aq) (3 drops) was added, sonicated to reslurry, and concentrated. The white residue was refluxed in EtOH overnight, then concentrated and triturated with acetone to give **41s55** as an off-white solid (100 mg, 91% yield). ^1^H NMR (400 MHz, DMSO-*d*_6_): δ 12.68 (s, 2H), 11.71 (br s, 1H), 8.37 (s, 3H), 8.21 (d, *J* = 1 Hz, 1H), 8.16–8.01 (m, 1H), 7.92–7.86 (m, 2H), 7.47 (d, *J* = 7.3 Hz, 2H), 7.41 (d, *J* = 1 Hz, 1H), 7.36 (t, *J* = 7 Hz, 2H), 7.27 (t, *J* = 7.3 Hz, 1H), 5.82 (q, *J* = 6.3 Hz, 1H), 4.09 (q, *J* = 5.3 Hz, 2H), 3.81 (s, 3H), 1.65 (d, *J* = 6.3 Hz, 3H).

#### 4.2.198 (5-((6-Carboxy-4-(1-phenylethoxy)benzo[*d*]thiazol-2-yl)-carbamoyl)-3,4-dichloro-1*H*-pyrrol-2-yl)methanaminium Chloride (**55**)

A mixture of methyl ester **41s55** (100 mg, 0.147 mmol), MeOH (3.7 mL), and 2 M NaOH (0.370 mL) was stirred at 50 °C for 24 h. NaOH (2 M, 0.370 mL) was added, and stirring was continued for a further 24 h. The reaction mixture was concentrated, water (1 mL) was added, and the solids were filtered off. The filtrate was acidified to pH 2 by adding 2 M HCl and cooled to 0 °C, and the precipitate was collected, washed with water, and air-dried. The resulting phthalate salt was triturated with a solution of HCl in MeOH (2 × 1 mL) to get **55** as a light yellow powder (23 mg, 29% yield). ^1^H NMR (400 MHz, DMSO-*d*_6_) δ 12.90 (s, 1H), 12.67 (s, 1H), 12.61 (s, 1H), 8.35 (s, 3H), 8.16 (s, 1H), 7.60–7.19 (m, 6H), 5.81 (q, *J* = 6 Hz, 1H), 4.09 (s, 2H), 1.65 (d, *J* = 6 Hz, 3H); HRMS (ESI) *m*/*z*: [M – Cl]^+^ calcd for C_22_H_19_Cl_2_N_4_O_4_S 505.0499; found 505.0487; HPLC purity (254 nm): 98%.

#### 4.2.199 Methyl 4-Nitro-3-((1,1,1-trifluoro-3-morpholinopropan-2-yl)oxy)benzoate (**30s57**)

Prepared according to the typical procedure H from **2s** (2.01 g, 10.19 mmol) and 1,1,1-trifluoro-3-morpholinopropan-2-ol (2.23 g, 11.21 mmol); light yellow oil solid (810 mg, 21% yield); ^1^H NMR (400 MHz, CDCl_3_): δ 7.86 (dd, *J* = 8.0 Hz, 0.6 Hz, 1H), 7.81–7.73 (m, 2H), 4.46 (dd, *J* = 10.2 Hz, 8.2 Hz, 1H), 4.39 (dd, *J* = 10.2 Hz, 3.9 Hz, 1H), 3.99 (s, 3H), 3.72–3.57 (m, 4H), 3.61–3.49 (m, 1H), 2.91–2.81 (m, 4H); MS (ESI): *m*/*z* calcd for C_15_H_17_F_3_N_2_O_6_: 378.10. Found: 379.1 [M + H]^+^.

#### 4.2.200 Methyl 4-Amino-3-((1,1,1-trifluoro-3-morpholinopropan-2-yl)oxy)benzoate (**31s57**)

Prepared according to the typical procedure I from **30s57** (836 mg, 2.21 mmol); colorless oil (770 mg, 100% yield); ^1^H NMR (400 MHz, CDCl_3_): δ 7.59 (dd, *J* = 8.2 Hz, 1.8 Hz, 1H), 7.49 (d, *J* = 1.8 Hz, 1H), 6.69 (d, *J* = 8.3 Hz, 1H), 4.33 (d, *J* = 6.1 Hz, 2H), 4.26 (s, 2H), 3.87 (s, 3H), 3.76–3.63 (m, 4H), 3.57–3.42 (m, 1H), 2.95–2.81 (m, 4H); ^19^F NMR (376 MHz, CDCl_3_): δ −68.37 (d, *J* = 8.6 Hz).

#### 4.2.201 Methyl 2-Amino-4-((1,1,1-trifluoro-3-morpholinopropan-2-yl)oxy)benzo[*d*]thiazole-6-carboxylate (**32s57**)

Prepared according to the typical procedure C from aniline **31s57** (781 mg, 2.24 mmol); light orange crispy foam (309 mg, 34% yield). ^1^H NMR (400 MHz, CDCl_3_): δ 8.01 (d, *J* = 1.4 Hz, 1H), 7.56 (d, *J* = 1.4 Hz, 1H), 5.73 (s, 2H), 4.53 (dd, *J* = 10.9 Hz, 7.8 Hz, 1H), 4.47 (dd, *J* = 10.8 Hz, 4.3 Hz, 1H), 3.93 (s, 3H), 3.73–3.58 (m, 5H), 2.94–2.88 (m, 4H); MS (ESI): *m*/*z* calcd for C_16_H_18_F_3_N_3_O_4_S: 405.09. Found: 406.0 [M + H]^+^.

#### 4.2.202 Methyl 2-(3,4-Dichloro-5-methyl-1*H*-pyrrole-2-carboxamido)-4-((1,1,1-trifluoro-3-morpholinopropan-2-yl)oxy)benzo[*d*]thiazole-6-carboxylate (**33s57**)

Prepared according to the typical procedure J from **32s57** (152 mg, 0.375 mmol); white solid (48 mg, 22% yield). ^1^H NMR (400 MHz, DMSO-*d*_6_): δ 12.26 (s, 1H), 12.09 (s, 1H), 8.26 (s, 1H), 7.59 (s, 1H), 4.66 (dd, *J* = 10.9 Hz, 6.8 Hz, 1H), 4.52 (dd, *J* = 11.0 Hz, 4.6 Hz, 1H), 3.95–3.78 (m, 4H), 3.59–3.47 (m, 4H), 2.91–2.80 (m, 4H), 2.26 (s, 3H); MS (ESI): *m*/*z* calcd for C_22_H_21_Cl_2_F_3_N_4_O_5_S: 580.06. Found: 580.9 [M + H]^+^.

#### 4.2.203 2-(3,4-Dichloro-5-methyl-1*H*-pyrrole-2-carboxamido)-4-((1,1,1-trifluoro-3-morpholinopropan-2-yl)oxy)benzo[*d*]-thiazole-6-carboxylic Acid (**57**)

Prepared according to the typical procedure E from methyl ester **33s57** (46 mg, 0.079 mmol); light brown solid (30 mg, 67% yield). ^1^H NMR (400 MHz, DMSO-*d*_6_): δ 13.01 (s, 1H), 12.34 (s, 1H), 12.04 (s, 1H), 8.29 (s, 1H), 7.63 (s, 1H), 4.73–4.59 (m, 1H), 4.58–4.46 (m, 1H), 3.93–3.80 (m, 1H), 3.53 (s, 4H), 2.86 (s, 4H), 2.28 (s, 3H); ^19^F NMR (376 MHz, DMSO-*d*_6_): δ −67.73 (d, *J* = 9.4 Hz); HRMS (ESI) *m*/*z*: [M – H]^−^ calcd for C_21_H_18_Cl_2_F_3_N_4_O_5_S 565.0327; found 565.0331; HPLC purity (254 nm): 96%.

#### 4.2.204 Methyl 3-(1-Cyclopropyl-2-morpholinoethoxy)-4-nitrobenzoate (**30s58**)

Prepared according to the typical procedure H from **2s** (2.10 g, 10.65 mmol) and 1-cyclopropyl-2-morpholinoethan-1-ol (2.01 g, 11.72 mmol); yellow oil containing ca. 25 mol % of a DIAD-derived impurity (2.80 g, 75% yield), used as such in the next step. ^1^H NMR (400 MHz, CDCl_3_): δ 7.91 (d, *J* = 1.6 Hz, 1H), 7.76 (d, *J* = 8.4 Hz, 1H), 7.67 (dd, *J* = 8.4 Hz, 1.6 Hz, 1H), 4.06 (app td, J = 7.9 Hz, 2.9 Hz, 1H), 3.96 (s, 3H), 3.63–3.48 (m, 4H), 2.83 (dd, *J* = 13.8 Hz, 8.2 Hz, 1H), 2.66 (dd, *J* = 13.8 Hz, 2.8 Hz, 1H), 2.51 (ddd, *J* = 11.5 Hz, 5.8 Hz, 3.5 Hz, 2H), 2.49–2.39 (m, 2H), 1.21–1.06 (m, 1H), 0.65–0.56 (m, 1H), 0.61–0.52 (m, 1H), 0.48–0.34 (m, 1H), 0.36–0.22 (m, 1H).

#### 4.2.205 Methyl 4-Amino-3-(1-cyclopropyl-2-morpholinoethoxy)-benzoate (**31s58**)

Prepared according to the typical procedure I from crude **30s58** (2.80 g); colorless oil containing ca. 0.25 mol % of a DIAD-derived impurity (2.7 g, 100% yield), used as such in the next step. ^1^H NMR (400 MHz, CDCl_3_): δ 7.71 (d, *J* = 1.9 Hz, 1H), 7.58 (dd, *J* = 8.3 Hz, 1.9 Hz, 1H), 6.63 (d, *J* = 8.3 Hz, 1H), 4.91 (s, 2H), 3.85 (s, 3H), 3.76–3.61 (m, 5H), 3.42 (app td, *J* = 8.8 Hz, 2.5 Hz, 1H), 2.83 (dd, *J* = 13.6 Hz, 9.0 Hz, 1H), 2.65–2.50 (m, 4H), 1.16–1.02 (m, 1H), 0.66–0.58 (m, 1H), 0.57–0.49 (m, 1H), 0.35–0.26 (m, 1H), 0.16–0.08 (m, 1H); MS (ESI): *m*/*z* calcd for C_17_H_24_N_2_O_4_: 320.17. Found: 320.9 [M + H]^+^.

#### 4.2.206 Methyl 2-Amino-4-(1-cyclopropyl-2-morpholinoethoxy)-benzo[*d*]thiazole-6-carboxylate (**32s58**)

Prepared according to the typical procedure C from aniline **31s57** (2.67 g, 8.33 mmol); yellow powder (1.1 g, 35% yield). ^1^H NMR (400 MHz, DMSO-*d*_6_): δ 7.91 (s, 1H), 7.84 (s, 2H), 7.48 (s, 1H), 4.28 (td, *J* = 7.3 Hz, 3.8 Hz, 1H), 3.82 (s, 3H), 3.51–3.44 (m, 3H), 2.73–2.30 (m, 6H), 1.16–1.03 (m, 1H), 0.51–0.34 (m, 2H), 0.37–0.23 (m, 2H); MS (ESI): *m*/*z* calcd for C_18_H_23_N_3_O_4_S: 377.14. Found: 377.9 [M + H]^+^.

#### 4.2.207 Methyl 4-(1-Cyclopropyl-2-morpholinoethoxy)-2-(3,4-dichloro-5-methyl-1*H*-pyrrole-2-carboxamido)benzo[*d*]thiazole-6-carboxylate (**33s58**)

Prepared according to the typical procedure J from **32s57** (200 mg, 0.529 mmol); gray solid (141 mg, 48% yield). ^1^H NMR (400 MHz, DMSO-*d*_6_): δ 12.25 (br s, 2H), 8.25 (s, 1H), 7.67 (s, 1H), 4.35 (s, 1H), 3.88 (s, 3H), 3.71–3.48 (m, 4H), 3.01–2.59 (m, 6H), 2.27 (s, 3H), 1.23–1.09 (m, 1H), 0.61–0.22 (m, 4H); MS (ESI): *m*/*z* calcd for C_24_H_26_Cl_2_N_4_O_5_S: 552.10. Found: 553.2 [M + H]^+^.

#### 4.2.208 4-(1-Cyclopropyl-2-morpholinoethoxy)-2-(3,4-dichloro-5-methyl-1*H*-pyrrole-2-carboxamido)benzo[*d*]thiazole-6-carboxylic Acid (**58**)

Prepared according to the typical procedure E from methyl ester **33s58** (120 mg, 0.216 mmol); brown solid (56 mg, 48% yield). ^1^H NMR (400 MHz, DMSO-*d*_6_): δ 12.90 (s, 1H), 12.20 (s, 2H), 8.18 (s, 1H), 7.64 (s, 1H), 4.43–4.21 (m, 1H), 3.74–3.43 (m, 4H), 2.97–2.65 (m, 6H), 2.27 (s, 3H), 1.27–1.05 (m, 1H), 0.64–0.22 (m, 4H); ^13^C NMR (101 MHz, DMSO-*d*_6_): δ 167.10, 160.30, 157.41, 149.37, 142.63, 132.87, 129.77, 126.51, 117.43, 116.51, 115.33, 112.94, 109.87, 80.19, 65.46, 61.76, 53.52, 14.10, 11.04, 3.43, 1.88; HRMS (ESI) *m*/*z*: [M + H]^+^ calcd for C_23_H_25_Cl_2_N_4_O_5_S 539.0923; found 539.0916; HPLC purity (254 nm): 98%.

#### 4.2.209 Methyl 2-(4-Chloro-5-methyl-1*H*-pyrrole-2-carboxamido)-4-(2-morpholino-1-phenylethoxy)benzo[*d*]thiazole-6-carboxylate (**33s59**)

Methyl 2-amino-4-(2-morpholino-1-phenylethoxy)benzo[*d*]thiazole-6-carboxylate (**32s36**) (92 mg, 0.222 mmol) was suspended in anhydrous DMF (2.5 mL), and 2,2,2-trichloro-1-(4-chloro-5-methyl-1*H*-pyrrol-2-yl)ethan-1-one (34s50) (70 mg, 0.267 mmol) and Na_2_CO_3_ (24 mg, 0.222 mmol) were added, and the reaction mixture was stirred overnight at 60 °C. DMF was removed under reduced pressure, and the residue was triturated successively with 10% aqueous citric acid, 1 M NaOH, and ethyl acetate; light yellow solid (82 mg, 66% yield). ^1^H NMR (400 MHz, DMSO-*d*_6_): δ 12.82 (s, 1H), 12.18 (s, 1H), 8.18–8.13 (m, 1H), 7.52–7.42 (m, 4H), 7.39–7.30 (m, 2H), 7.30–7.21 (m, 1H), 5.85 (dd, *J* = 7.9, 4.2 Hz, 1H), 3.81 (s, 3H), 3.58–3.50 (m, 4H), 3.01 (dd, *J* = 13.4, 7.9 Hz, 1H), 2.70–2.62 (m, 1H), 2.56 (dd, *J* = 9.7, 4.7 Hz, 4H), 2.22 (s, 3H).

#### 4.2.210 4-(2-((6-Carboxy-2-(4-chloro-5-methyl-1*H*-pyrrole-2-carboxamido)benzo[*d*]thiazol-4-yl)oxy)-2-phenylethyl)morpholin-4-ium Chloride (**59**)

Prepared according to the typical procedure E from methyl ester **33s59** (71 mg, 0.128 mmol); off-white solid (42 mg, 57% yield). ^1^H NMR (400 MHz, DMSO-*d*_6_): δ 13.36–12.59 (m, 2H), 12.34 (s, 1H), 11.40 (s, 1H), 8.22 (s, 1H), 7.70–7.18 (m, 6H), 6.53 (d, *J* = 8.0 Hz, 1H), 4.20–3.49 (m, 10H), 2.24 (s, 3H); ^13^C NMR (101 MHz, DMSO-*d*_6_): δ 166.72, 160.72, 158.05, 147.47, 142.65, 137.04, 133.22, 131.77, 129.00, 128.85, 126.28, 126.24, 120.69, 117.29, 113.67, 112.23, 109.80, 75.39, 63.22, 61.24, 52.78, 52.29, 10.37. HRMS (ESI) *m*/*z*: [M – Cl]^+^ calcd for C_26_H_26_ClN_4_O_5_S 541.1312; found 541.1298; HPLC purity (254 nm): 98%.

#### 4.2.211 4-(2-((2-(4-Fluoro-5-methyl-1*H*-pyrrole-2-carboxamido)-6-(methoxycarbonyl)benzo[*d*]thiazol-4-yl)oxy)-2-phenylethyl)morpholin-4-ium Chloride (**33s60×HCl**)

Oxalyl chloride (0.40 mL, 4.76 mmol) was added to a suspension of 4-fluoro-5-methyl-1*H*-pyrrole-2-carboxylic acid (**35s51**) (68 mg, 0.476 mmol) in anhydrous dichloromethane (5 mL) and stirred at 22 °C overnight. The resulting light brown solution was concentrated under reduced pressure and filled with argon to obtain the acyl chloride, containing 5 mol % of the starting acid, as a light brown solid. Toluene (9.5 mL) and methyl 2-amino-4-(2-morpholino-1-phenylethoxy)benzo[*d*]-thiazole-6-carboxylate (**32s36**) (197 mg, 0.476 mmol) were added, and the resulting suspension was refluxed overnight. The reaction mixture was cooled to 22 °C, and the precipitate was collected, washed with toluene, and triturated with methanol to obtain the title compound as a white powder (85 mg, 31% yield). ^1^H NMR (400 MHz, DMSO-*d*_6_): δ 12.67 (s, 1H), 11.92 (s, 1H), 10.50 (s, 1H), 8.25 (s, 1H), 7.56–7.27 (m, 7H), 6.44–6.35 (m, 1H), 4.12–3.96 (m, 2H), 3.87–3.61 (m, 11H), 2.21 (s, 3H). HPLC purity (254 nm): 98.8%.

#### 4.2.212 4-(2-((6-Carboxy-2-(4-fluoro-5-methyl-1*H*-pyrrole-2-carboxamido)benzo[*d*]thiazol-4-yl)oxy)-2-phenylethyl)morpholin-4-ium Chloride (**60×HCl**)

Prepared according to the typical procedure E from methyl ester **33s60×HCl** (81 mg, 0.140 mmol); off-white solid (22 mg, 28% yield). ^1^H NMR (400 MHz, DMSO-*d*_6_): δ 12.91 (s, 1H), 12.65 (s, 1H), 11.91 (s, 1H), 10.57 (s, 1H), 8.20 (s, 1H), 7.57–7.21 (m, 7H), 6.49–6.29 (m, 1H), 4.17–3.33 (m, 10H), 2.21 (s, 3H); ^19^F NMR (376 MHz, DMSO-*d*_6_): δ −166.52; HRMS (ESI) *m*/*z*: [M – Cl]^+^ calcd for C_26_H_26_FN_4_O_5_S 525.1608; found 525.1599; HPLC purity (254 nm): 95%.

#### 4.2.213 *tert*-Butyl 2-(4-Fluoro-5-methyl-1*H*-pyrrole-2-carboxamido)-4-isopropoxybenzo[*d*]thiazole-6-carboxylate (**39s61**)

To a suspension of 4-fluoro-5-methyl-1*H*-pyrrole-2-carboxylic acid **35s51** (52 mg, 0.362 mmol) in anhydrous dichloromethane (4 mL), oxalyl chloride (0.31 mL. 3.62 mmol) was added and the reaction mixture was stirred at 22 °C overnight under an argon atmosphere. After the volatiles were removed under reduced pressure, **5s46** (93 mg, 0.302 mmol) and toluene (7 mL) were added to the residue and the reaction mixture was stirred at 130 °C overnight. The gray precipitate was collected and washed with toluene (104 mg, 79% yield). ^1^H NMR (400 MHz, DMSO-*d*_6_): δ 12.66 (s, 1H), 11.85 (s, 1H), 8.13 (d, *J* = 1 Hz, 1H), 7.44 (d, *J* = 1 Hz, 1H), 7.26 (d, *J* = 2.3 Hz, 1H), 4.95–4.84 (m, 1H), 2.20 (s, 3H), 1.36 (d, *J* = 6.0 Hz, 6H). MS (ESI): *m*/*z* calcd for C_21_H_24_FN_3_O_4_S: 433.15, found 432.2 [M – H]^−^.

#### 4.2.214 2-(4-Fluoro-5-methyl-1*H*-pyrrole-2-carboxamido)-4-*iso*-propoxybenzo[*d*]thiazole-6-carboxylic Acid (**61**)

*tert*-Butyl ester **39s61** (75 mg, 0.173 mmol) was suspended in dichloromethane (3 mL), trifluoroacetic acid (0.13 mL, 1.73 mmol) was added, and the suspension turned into a brown solution. The reaction mixture was stirred at 22 °C overnight, the solvent was removed under reduced pressure, and the residue was triturated with methanol; light brown solid (64 mg, 98% yield). ^1^H NMR (400 MHz, DMSO-*d*_6_): δ 12.92 (s, 1H), 12.65 (s, 1H), 11.84 (s, 1H), 8.17 (d, *J* = 1.2 Hz, 1H), 7.48 (s, 1H), 7.26 (d, *J* = 2.7 Hz, 1H), 4.89 (hept, *J* = 6 Hz, 1H), 2.19 (s, 3H), 1.36 (d, *J* = 6 Hz, 6H); HRMS (ESI) *m*/*z*: [M + H]^+^ calcd for C_17_H_17_FN_3_O_4_S 378.0924; found 378.0920; HPLC purity (254 nm): 93%.

#### 4.2.215 *tert*-Butyl 2-(4-Cyano-5-methyl-1*H*-pyrrole-2-carboxamido)-4-isopropoxybenzo[*d*]thiazole-6-carboxylate (**39s62**)

A suspension of 4-cyano-5-methyl-1*H*-pyrrole-2-carboxylic acid (**35s52**) (60 mg, 0.400 mmol) in thionyl chloride (1.5 mL) was stirred at 22 °C overnight, and the volatiles were removed under reduced pressure. Compound **5s46** (100 mg, 0.324 mmol) and toluene (4 mL) were added to the residue, and the reaction mixture was stirred at 130 °C overnight. The precipitate was collected, washed with toluene, and triturated with MeOH; white solid (93 mg, 65% yield). ^1^H NMR (400 MHz, DMSO-*d*_6_): δ 12.99 (s, 1H), 12.76 (s, 1H), 8.15 (s, 1H), 7.71 (s, 1H), 7.45 (s, 1H), 4.96–4.83 (m, 1H), 2.39 (s, 3H), 1.57 (s, 9H), 1.36 (d, *J* = 6 Hz, 6H). MS (ESI): *m*/*z* calcd for C_22_H_24_FN_4_O_4_S: 440.15. Found 439.2 [M – H]^−^. 60% yield.

#### 4.2.216 2-(4-Cyano-5-methyl-1*H*-pyrrole-2-carboxamido)-4-isopropoxybenzo[*d*]thiazole-6-carboxylic Acid (**62**)

To a suspension of *tert*-butyl ester **39s62** (80 mg, 0.182 mmol) in dichloromethane (3 mL) was added trifluoroacetic acid (0.14 mL, 1.82 mmol). The suspension turned into a light brown solution. The reaction mixture was stirred at 22 °C overnight. The white precipitate was collected and washed with dichloromethane, then triturated with MeOH to get the title compound as a white solid (42 mg, 60% yield). ^1^H NMR (400 MHz, DMSO-*d*_6_) δ 12.97 (s, 2H), 12.75 (s, 1H), 8.19 (s, 1H), 7.71 (s, 1H), 7.49 (s, 1H), 4.96–4.84 (m, 1H), 2.39 (s, 3H), 1.36 (d, *J* = 5.3 Hz, 6H). HRMS (ESI) *m*/*z*: [M + H]^+^ calcd for C_18_H_17_N_4_O_4_S 385.0971; found 385.0962; HPLC purity (254 nm): 98%.

#### 4.2.217 *tert*-Butyl 2-(4-Chloro-2-methyl-1*H*-imidazole-5-carboxamido)-4-isopropoxybenzo[*d*]thiazole-6-carboxylate (**42s63**)

A mixture of 4-chloro-2-methyl-1*H*-imidazole-5-carboxylic acid (**37s63**) (90 mg, 0.561 mmol), SOCl_2_ (2 mL, 28.1 mmol), and DMF (2 drops) was refluxed for 1.5 h. The reaction mixture was concentrated under reduced pressure to obtain the crude acyl chloride as a white solid. Dry toluene (11 mL) and **5s46** (173 mg, 0.561 mmol) were added, and the resulting suspension was stirred at 130 °C overnight. After cooling to 22 °C, the precipitate (starting carboxylic acid) was filtered off and the filtrate was concentrated. To the oily residue was added EtOAc (1 mL), the precipitate (starting 2-aminobenzo[*d*]thiazole) was filtered off, and the filtrate was concentrated. The residue was purified by preparative layer chromatography (20 cm × 20 cm plate, 2 mm silica layer; eluent EtOAc/hexane 2:1) to obtain the crude product (85% pure (by HPLC)). This was dissolved in EtOAc (20 mL) and washed with K_2_CO_3_(aq) (2 × 15 mL). The organic layer was washed with brine, dried over Na_2_SO_4_, filtered, and concentrated to obtain the title compound as a white solid (59 mg, 23% yield). ^1^H NMR (400 MHz, DMSO-*d*_6_): δ = 12.53 (br s, 1H), 7.95 (s, 1H), 7.31 (s, 1H), 4.97 (hept, *J* = 6.0 Hz, 1H), 2.28 (s, 3H), 1.56 (s, 9H), 1.30 (d, *J* = 6.0 Hz, 6H) ppm; MS (ESI): *m*/*z* calcd for C_20_H_23_ClN_4_O_4_S: 450.11. Found: 451.0 [M + H]^+^; HPLC purity (254 nm): 93.5%.

#### 4.2.218 2-(4-Chloro-2-methyl-1*H*-imidazole-5-carboxamido)-4-*iso*-propoxybenzo[*d*]thiazole-6-carboxylic Acid (**63**)

A suspension of *tert*-butyl ester **42s63** (59 mg, 0.131 mmol) in dichloromethane (3 mL) was treated with trifluoroacetic acid (0.2 mL), and the resulting solution was stirred at room temperature and monitored by HPLC-MS. After complete conversion, the reaction mixture was concentrated under reduced pressure and the solid residue was triturated with water to obtain a beige solid (41 mg, 79% yield). ^1^H NMR (400 MHz, DMSO-*d*_6_) δ 12.9 (br s, 2H), 12.25 (s, 1H), 8.20 (d, *J* = 1.4 Hz, 1H), 7.49 (d, *J* = 1.4 Hz, 1H), 4.90 (hept, *J* = 6.0 Hz, 1H), 2.36 (s, 3H), 1.36 (d, *J* = 6.0 Hz, 7H); HRMS (ESI) *m*/*z*: [M + H]^+^ calcd for C_16_H_16_ClN_4_O_4_S 395.0581; found 395.0577; HPLC purity (254 nm): 97%.

#### 4.2.219 2-Trichloroacetyl-3,4-dichloro-5-methyl-1*H*-pyrrole (**46s**)

Ethyl 3,4-dichloro-5-methyl-1*H*-pyrrole-2-carboxylate (**44s**) (2.00 g, 9.0 mmol) was suspended in water (90 mL), and KOH (2.52 g, 45 mmol) was added. The flask was connected to a Dean–Stark apparatus, evacuated, and backfilled with nitrogen three times. The apparatus was then connected to a nitrogen line with a bubbler. The receiving tube of the Dean–Stark apparatus was prefilled with 1,2-dichloroethane (3 mL) and the rest with water. The reaction mixture was then refluxed (bath temperature between 150 and 190 °C). After 30 min, the starting material was dissolved and the mixture color turned dark brown. The decarboxylated pyrrole started to distill over, and the reaction was finished after 4 h. Refluxing longer sometimes resulted in more pyrrole being collected. The 1,2-dichloroethane solution was directly used in the next reaction step. A round bottom flask was charged with Na_2_SO_4_ and 1,2-dichloroethane (36 mL) under nitrogen. Under a positive stream of nitrogen, the dichloroethane layer was withdrawn from the Dean–Stark apparatus. To this solution, trichloroacetyl chloride (2.45 g, 13.5 mmol) was added dropwise, and the mixture was stirred at 22 °C overnight (color change from colorless to yellow, pink, dark brown, and black was observed). Saturated aqueous NaHCO_3_ was added, and the resulting mixture was vigorously stirred (gas evolution). The water layer was separated and extracted three times with dichloromethane; the combined organic layers were washed with water, dried over Na_2_SO_4_, and concentrated to afford **46s** as a dark green to black oil that solidified upon standing (1.33–0.66 g, 25–50% yield over two steps). 1H NMR (500 MHz CDCl_3_): δ 9.16 (bs, 1H), 2.39 (s, 3H); ^13^C NMR (101 MHz, CDCl_3_) δ 169.72, 133.56, 123.62, 117.11, 114.70, 94.94, 12.22; MS (ESI): *m*/*z* calcd for C_7_H_4_Cl_5_NO: 294.87. Found: 296.2 [M + H]^+^.

#### 4.2.220 Methyl 2-Amino-4-(1-(4-(methylsulfonyl)phenyl)ethoxy)-benzo[*d*]thiazole-6-carboxylate (**5s64**)

Prepared according to the typical procedure F from **1s** (508 mg, 2.27 mmol) and 1-(1-chloroethyl)-4-methylsulfonylbenzene (595 mg, 2.72 mmol); yellow powder (175 mg, 19% yield). ^1^H NMR (400 MHz, DMSO-*d*_6_): δ 7.99 (s, 2H), 7.91 (d, *J* = 8.4 Hz, 2H), 7.91 (d, *J* = 1.5 Hz, 1H), 7.71 (d, *J* = 8.4 Hz, 2H), 7.27 (d, *J* = 1.5 Hz, 1H), 5.91 (q, *J* = 6.3 Hz, 1H), 3.76 (s, 3H), 3.20 (s, 3H), 1.59 (d, *J* = 6.3 Hz, 3H).

#### 4.2.221 Methyl 2-(3,4-Dichloro-5-methyl-1*H*-pyrrole-2-carboxamido)-4-(1-(4-(methylsulfonyl)phenyl)ethoxy)benzo[*d*]thiazole-6-carboxylate (**6s64**)

Prepared according to the typical procedure D from 2-aminobenzothiazole **5s64** (155 mg, 0.381 mmol); (191 mg, 86% yield). ^1^H NMR (400 MHz, DMSO-*d*_6_): δ 12.32 (s, 1H), 12.29 (s, 1H), 8.23 (s, 1H), 7.94 (d, *J* = 8.4 Hz, 2H), 7.74 (d, *J* = 8.4 Hz, 2H), 7.38 (s, 1H), 6.00 (q, *J* = 6.3 Hz, 1H), 3.81 (s, 3H), 3.20 (s, 3H), 2.29 (s, 3H), 1.66 (d, *J* = 6.3 Hz, 3H).

#### 4.2.222 2-(3,4-Dichloro-5-methyl-1*H*-pyrrole-2-carboxamido)-4-(1-(4-(methylsulfonyl)phenyl)ethoxy)benzo[*d*]thiazole-6-carboxylic Acid (**64**)

Prepared according to the typical procedure E from methyl ester **6s64** (161 mg, 0.277 mmol); brown solid (148 mg, 94% yield). ^1^H NMR (400 MHz, DMSO-d_6_): δ 12.93 (s, 1H), 12.29 (s, 2H), 8.19 (d, *J* = 1 Hz, 1H), 7.94 (d, *J* = 8.5 Hz, 2H), 7.74 (d, *J* = 8.5 Hz, 2H), 7.37 (d, *J* = 1 Hz, 1H), 5.98 (q, *J* = 6.3 Hz, 1H), 3.20 (s, 3H), 2.29 (s, 3H), 1.66 (d, *J* = 6.3 Hz, 3H); ^13^C NMR (101 MHz, DMSO-*d*_6_): δ 166.86, 159.90, 156.66, 148.66, 148.50, 140.07, 133.01, 130.03, 127.50, 126.57, 126.45, 116.89, 116.45, 115.78, 110.46, 110.03, 74.86, 43.41, 24.10, 11.10; HRMS (ESI) *m*/*z*: [M + H]^+^ calcd for C_23_H_20_Cl_2_N_3_O_6_S_2_ 568.0171; found 568.0167; HPLC purity (254 nm): 94%.

#### 4.2.223 1-(4-(Morpholinomethyl)phenyl)ethan-1-one (**47s65**)

4′-(Bromomethyl)acetophenone (2.0 g, 9.39 mmol) was dissolved in MeCN (45 mL) followed by the addition of K_2_CO_3_ (1.30 g, 9.39 mmol). The mixture was cooled to 0 °C, and morpholine (0.81 mL, 9.39 mmol) was added dropwise. The reaction mixture was stirred overnight at 22 °C. The solvent was removed under reduced pressure, and the residue was dissolved in EtOAc. The organic layer was washed with water and brine and dried with Na_2_SO_4_. After filtration, the solvent was removed under reduced pressure; yellow oil (2.02 g, 98% yield). ^1^H NMR (400 MHz, DMSO-*d*_6_): δ 7.92 (d, *J* = 8.3 Hz, 2H), 7.46 (d, *J* = 8.3 Hz, 2H), 3.61–3.55 (m, 4H), 3.53 (s, 2H), 2.57 (s, 3H), 2.39–2.29 (m, 4H); MS (ESI): *m*/*z* calcd for C_13_H_17_NO_2_: 219.12. Found: 220.0 [M + H]^+^.

#### 4.2.224 1-(4-(Morpholinomethyl)phenyl)ethan-1-ol (**48s65**)

To a mixture of ketone **47s65** (1.9 g, 8.66 mmol) and dry EtOH (30 mL), NaBH_4_ (328 mg, 8.66 mmol) was added in small portions at 0 °C and the reaction mixture was stirred at 22 °C for 1.5 h. The solvent was removed under reduced pressure, and the residue was dissolved in EtOAc. The organic layer was washed with water and brine and dried over Na_2_SO_4_. After filtration, the solvent was removed under reduced pressure; yellow oil (1.78 g, 93% yield). ^1^H NMR (400 MHz, DMSO-*d*_6_): δ 7.29 (d, *J* = 8.1 Hz, 2H), 7.24 (d, *J* = 8.1 Hz, 2H), 5.10 (d, *J* = 4.2 Hz, 1H), 4.75–4.65 (m, 1H), 3.61–3.51 (m, 4H), 3.43 (s, 2H), 2.37–2.29 (m, 4H), 1.31 (d, *J* = 6.4 Hz, 3H); MS (ESI): *m*/*z* calcd for C_13_H_19_NO_2_: 221.14. Found: 222.1 [M + H]^+^.

#### 4.2.225 Methyl 3-(1-(4-(Morpholinomethyl)phenyl)ethoxy)-4-nitrobenzoate (**49s65**)

Prepared according to the typical procedure H from **2s** (1.17 g, 5.94 mmol) and alcohol **48s65** (1.45 g, 6.53 mmol); yellow oil (1.76 g, 74% yield). ^1^H NMR (400 MHz, DMSO-*d*_6_) δ 7.95 (d, *J* = 8.3 Hz, 1H), 7.71 (d, *J* = 1.5 Hz, 1H), 7.59 (dd, *J* = 8.3 Hz, 1.5 Hz, 1H), 7.39 (d, *J* = 8.1 Hz, 2H), 7.31 (d, *J* = 8.1 Hz, 2H), 5.86 (q, *J* = 6.3 Hz, 1H), 3.84 (s, 3H), 3.60–3.49 (m, 4H), 3.42 (s, 2H), 2.39–2.22 (m, 4H), 1.57 (d, *J* = 6.3 Hz, 3H); MS (ESI): *m*/*z* calcd for C_21_H_24_N_2_O_6_: 400.16. Found: 400.9 [M + H]^+^.

#### 4.2.226 Methyl 4-Amino-3-(1-(4-(morpholinomethyl)phenyl)ethoxy)benzoate (**50s65**)

Prepared according to the typical procedure B from **49s65** (1.68 g, 4.20 mmol); orange oil (1.23 g, 79% yield). ^1^H NMR (400 MHz, DMSO-*d*_6_): δ 7.40 (d, *J* = 8.0 Hz, 2H), 7.31 (dd, *J* = 8.3, 1.7 Hz, 1H), 7.27 (d, *J* = 8.0 Hz, 2H), 7.21 (d, *J* = 1.7 Hz, 1H), 6.63 (d, *J* = 8.3 Hz, 1H), 5.69 (s, 2H), 5.46 (q, *J* = 6.3 Hz, 1H), 3.68 (s, 3H), 3.60–3.51 (m, 4H), 3.42 (s, 2H), 2.42–2.21 (m, 4H), 1.56 (d, *J* = 6.3 Hz, 3H). MS (ESI): *m*/*z* calcd for C_21_H_26_N_2_O_4_: 370.19. Found: 370.2 [M + H]^+^.

#### 4.2.227 Methyl 2-Amino-4-(1-(4-(morpholinomethyl)phenyl)ethoxy)benzo[*d*]thiazole-6-carboxylate (**51s65**)

Prepared according to the typical procedure C from aniline **50s65** (1.11 g, 3.00 mmol); yellow solid (654 mg, 51% yield). ^1^H NMR (400 MHz, DMSO-*d*_6_): δ 7.94 (s, 2H), 7.87 (d, *J* = 1.5 Hz, 1H), 7.38 (d, *J* = 8.1 Hz, 2H), 7.27 (d, *J* = 8.1 Hz, 2H), 7.25 (d, *J* = 1.5 Hz, 1H), 5.71 (q, *J* = 6.3 Hz, 1H), 3.76 (s, 3H), 3.60–3.50 (m, 4H), 3.41 (s, 2H), 2.39–2.20 (m, 4H), 1.57 (d, *J* = 6.3 Hz, 3H); MS (ESI): *m*/*z* calcd for C_22_H_25_N_3_O_4_S: 427.16. Found: 428.1 [M + H]^+^.

#### 4.2.228 Methyl 2-(3,4-Dichloro-5-methyl-1*H*-pyrrole-2-carboxamido)-4-(1-(4-(morpholinomethyl)phenyl)ethoxy)benzo[*d*]thiazole-6-carboxylate (**52s65**)

Prepared according to the typical procedure J from **51s65** (130 mg, 0.304 mmol); gray solid (75 mg, 41% yield). ^1^H NMR (400 MHz, DMSO-*d*_6_) δ 12.29 (br s, 2H), 8.16 (s, 1H), 7.41 (d, *J* = 8.1 Hz, 2H), 7.37 (s, 1H), 7.28 (d, *J* = 8.1 Hz, 2H), 5.79 (q, *J* = 6.3 Hz, 1H), 3.80 (s, 3H), 3.60–3.49 (m, 4H), 3.41 (s, 2H), 2.36–2.29 (m, 4H), 2.28 (s, 3H), 1.64 (d, *J* = 6.3 Hz, 3H); MS (ESI): *m*/*z* calcd for C_28_H_28_Cl_2_N_4_O_5_S: 602.16. Found: 603.2 [M + H]^+^.

#### 4.2.229 4-(4-(1-((6-Carboxy-2-((3,4-dichloro-5-methyl-1*H*-pyrrole-2-carbonyl)oxy)benzo[*d*]thiazol-4-yl)oxy)ethyl)benzyl)morpholin-4-ium Chloride (**65×HCl**)

Prepared according to the typical procedure E from methyl ester **52s65** (59 mg, 0.097 mmol); brown solid (12 mg, 21% yield). ^1^H NMR (400 MHz, DMSO-*d*_6_): δ 12.88 (s, 1H), 12.32 (s, 1H), 12.25 (s, 1H), 10.29 (s, 1H), 8.17 (s, 1H), 7.90–7.73 (m, 1H), 7.66–7.48 (m, 4H), 7.36 (s, 1H), 5.86 (q, *J* = 6.3 Hz, 1H), 4.35–4.23 (m, 2H), 4.01–3.86 (m, 2H), 3.74–3.58 (m, 2H), 3.25–3.15 (m, 2H), 3.14–3.00 (m, 2H), 2.29 (s, 3H), 1.65 (d, *J* = 6.3 Hz, 3H); HRMS (ESI) *m*/*z*: [M – Cl]^+^ calcd for C_27_H_27_Cl_2_N_4_O_5_S 589.1079; found 589.1068; HPLC purity (220 nm): 91%.

#### 4.2.230 Methyl 2-(4-Chloro-5-methyl-1*H*-pyrrole-2-carboxamido)-4-(1-(4-(morpholinomethyl)phenyl)ethoxy)benzo[*d*]thiazole-6-carboxylate Monocitrate (**53s66**)

2,2,2-Trichloro-1-(4-chloro-5-methyl-1*H*-pyrrol-2-yl)ethan-1-one (**34s50**) (117 mg, 0.398 mmol) and **51s65** (170 mg, 0.398 mmol) were dissolved in DMF (4 mL), and Na_2_CO_3_ (42 mg, 0.398 mmol) was added, and the reaction was stirred at 60 °C overnight. The volatiles were removed under reduced pressure, and the crude residue was triturated with 10% aqueous citric acid and EtOAc to obtain the product as a monocitrate salt; gray solid (209 mg, 69% yield). ^1^H NMR (400 MHz, DMSO-*d*_6_): δ 12.83 (br s, 1H), 12.20 (s, 1H), 8.16 (d, *J* = 1.1 Hz, 1H), 7.46 (d, *J* = 2.6 Hz, 1H), 7.43 (d, *J* = 7.8 Hz, 2H), 7.37 (s, 1H), 7.31 (d, *J* = 7.8 Hz, 3H), 5.79 (q, *J* = 6 Hz, 1H), 4.10 (br s, 1H), 3.80 (s, 3H), 3.58 (m, 8H), 3.17 (s, 2H), 2.73 (d, *J* = 15.3 Hz, 2H), 2.63 (d, *J* = 15.3 Hz, 2H), 2.44 (s, 3H), 2.22 (s, 3H), 1.64 (d, *J* = 6 Hz, 3H); MS (ESI): *m*/*z* calcd for C_28_H_30_ClN_4_O_5_S: 569.16. Found: 569.4 [M + H]^+^.

#### 4.2.231 4-(4-(1-((6-Carboxy-2-(4-chloro-5-methyl-1*H*-pyrrole-2-carboxamido)benzo[*d*]thiazol-4-yl)oxy)ethyl)benzyl)morpholin-4-ium Chloride (**66×HCl**)

Prepared according to the typical procedure E from methyl ester **53s66** (157 mg, 0.206 mmol); white solid (71 mg, 62% yield). ^1^H NMR (400 MHz, DMSO-*d*_6_): δ 12.81 (s, 2H), 12.20 (d, *J* = 2.8 Hz, 1H), 10.67 (s, 1H), 8.14 (s, 1H), 7.62–7.51 (m, 4H), 7.46 (d, *J* = 2.8 Hz, 1H), 7.35 (s, 1H), 5.85 (q, *J* = 6.2 Hz, 2H), 4.29 (s, 2H), 3.92 (d, *J* = 12.7 Hz, 2H), 3.70 (t, *J* = 12.3 Hz, 2H), 3.24–3.13 (m, 2H), 3.13–2.98 (m, 2H), 2.22 (s, 3H), 1.65 (d, *J* = 6.2 Hz, 4H); HRMS (ESI) *m*/*z*: [M – Cl]^+^ calcd for C_27_H_28_ClN_4_O_5_S 555.1469; found 555.1457; HPLC purity (254 nm): 98%.

#### 4.2.232 4-(4-(1-((2-(4-Fluoro-5-methyl-1*H*-pyrrole-2-carboxamido)-6-(methoxycarbonyl)benzo[*d*]thiazol-4-yl)oxy)ethyl)-benzyl)morpholin-4-ium Chloride (**53s67×HCl**)

A mixture of 4-fluoro-5-methyl-1*H*-pyrrole-2-carboxylic acid (**35s51**) (43 mg, 0.300 mmol) and oxalyl chloride (0.26 mL, 3 mmol) in dichloromethane (3 mL) was stirred at 22 °C overnight, and the resulting solution was concentrated under reduced pressure. To the solid residue were added 51s65 (128 mg, 0.300 mmol) and toluene (6 mL), and the reaction mixture was stirred at 130 °C overnight. The precipitate was collected and washed with toluene and EtOAc to get the product; gray solid (49 mg, 27% yield). ^1^H NMR (400 MHz, DMSO-*d*_6_): δ 12.75 (s, 1 H), 11.86 (s, 1 H), 10.28 (br s, 1H), 8.17 (s, 1H), 7.55 (m, 4H), 7.35 (s, 1H), 7.29 (d, *J* = 2.9 Hz, 1H), 5.87 (q, *J* = 6.3 Hz, 1H), 4.30 (s, 2H), 3.93 (d, *J* = 12.6 Hz, 2H), 3.80 (s, 3H), 3.65 (t, *J* =12.3 Hz, 2H), 3.20 (d, *J* = 12.6 Hz, 2H), 3.07 (m, 2H), 2.20 (s, 3H), 1.65 (d, *J* = 6.3 Hz, 3H); ^19^F NMR (376 MHz, DMSO-*d*_6_): δ −166.51; MS (ESI): *m*/*z* calcd for C_28_H_30_FN_4_O_5_S: 553.19. Found: 553.3 [M – Cl]^+^.

#### 4.2.233 4-(4-(1-((6-Carboxy-2-(4-fluoro-5-methyl-1*H*-pyrrole-2-carboxamido)benzo[*d*]thiazol-4-yl)oxy)ethyl)benzyl)morpholin-4-ium Chloride (**67×HCl**)

Prepared according to the typical procedure E from methyl ester **53s67** hydrochloride (47 mg, 0.080 mmol); brown solid (10 mg, 22% yield). ^1^H NMR (400 MHz, DMSO-*d*_6_): δ 12.8 (brs, 1H), 12.72 (s, 1H), 11.86 (s, 1H), (brs, 1H), 8.13 (s, 1H), 7.55 (s, 4H), 7.34 (s, 1H), 7.29 (s, 1H), 5.85 (q, *J* = 6.3 Hz, 1H), 4.29 (s, 2H), 3.92 (d, *J* = 12.6 Hz, 2H), 3.67 (t, *J* = 12.2 Hz, 2H), 3.19 (m, 2H), 3.07 (m, 2H), 2.20 (s, 3H), 1.65 (d, *J* = 6.3 Hz, 3H); ^19^F NMR (376 MHz, DMSO-*d*_6_): δ −166.54; HRMS (ESI) *m*/*z*: [M – Cl]^+^ calcd for C_27_H_28_FN_4_O_5_S 539.1764; found 539.1758; HPLC purity (254 nm): 97%.

#### 4.2.234 4-(4-(1-((2-(4-Cyano-5-methyl-1*H*-pyrrole-2-carboxamido)-6-(methoxycarbonyl)benzo[*d*]thiazol-4-yl)oxy)ethyl)-benzyl)morpholin-4-ium Chloride (**53s68×HCl**)

Prepared according to the typical procedure D from **51s65** (93 mg, 0.218 mmol) and 4-cyano-5-methyl-1*H*-pyrrole-2-carboxylic acid (**35s52**) (33 mg, 0.218 mmol); white solid (95 mg, 73% yield). 1H NMR (400 MHz, DMSO-*d*_6_): δ 13.07 (s, 1H), 12.76 (s, 1H), 8.18 (d, *J* = 1.1 Hz, 1H), 7.73 (s, 1H), 7.40 (d, *J* = 8.1 Hz, 2H), 7.38 (d, *J* = 1.1 Hz, 1H), 7.28 (d, *J* = 8.1 Hz, 2H), 5.82–5.74 (m, 1H), 3.80 (s, 3H), 3.59–3.50 (m, 4H), 3.41 (s, 2H), 2.33–2.25 (m, 5H), 1.64 (d, *J* = 6.3 Hz, 3H).

#### 4.2.235 4-(4-(1-((6-Carboxy-2-(4-cyano-5-methyl-1*H*-pyrrole-2-carboxamido)benzo[*d*]thiazol-4-yl)oxy)ethyl)benzyl)morpholin-4-ium Chloride (**68×HCl**)

Prepared according to the typical procedure E from methyl ester **53s68** hydrochloride (78 mg, 0.131 mmol); white solid (26 mg, 34% yield). ^1^H NMR (400 MHz, DMSO-*d*_6_): δ 13.05 (s, 1H), 12.86 (s, 1H), 12.77 (s, 1H), 10.53–10.23 (m, 1H), 8.16 (s, 1H), 7.75 (s, 1H), 7.64–7.44 (m, 4H), 7.35 (s, 1H), 5.86 (q, *J* = 6.1 Hz, 1H), 4.41–4.18 (m, 1H), 4.03–3.82 (m, *J* = 12.1 Hz, 2H), 3.75–3.57 (m, 2H), 3.26–3.15 (m, 2H), 3.14–2.95 (m, 2H), 2.40 (s, 3H), 1.65 (d, *J* = 6.2 Hz, 3H); HRMS (ESI) *m*/*z*: [M – Cl]^+^ calcd for C_28_H_28_N_5_O_5_S 546.1811; found 546.1802; HPLC purity (254 nm): 98%.

#### 4.2.236 2-(3,4-Dichloro-5-methyl-1*H*-pyrrole-2-carboxamido)-4-(1-(4-(piperazin-1-ylmethyl)phenyl)ethoxy)benzo[*d*]thiazole-6-carboxylic Acid (**69**)

Hydrochloride of the Boc-protected compound **70** (55 mg, 0.076 mmol) was suspended in 4 M HCl in dioxane (1 mL). The reaction mixture was stirred at 22 °C for 1 h. The solvent was removed under reduced pressure, and to the residue, EtOAc and water were added. The two phases were separated, and to the water phase, 1 M NaOH was added to reach pH 6. The precipitate was collected to obtain the product; brown powder (16 mg, 36% yield). ^1^H NMR (400 MHz, DMSO-*d*_6_): δ 7.92 (s, 1H), 7.38 (d, *J* = 7.8 Hz, 2H), 7.24 (d, *J* = 7.8 Hz, 2H), 7.15 (s, 1H), 5.72 (q, *J* = 6.2 Hz, 1H), 3.51 (s, 2H), 2.95 (s, 4H), 2.44 (s, 4H), 2.23 (s, 3H), 1.63 (d, *J* = 6.2 Hz, 3H); HRMS (ESI) *m*/*z*: [M + H]^+^ calcd for C_27_H_28_Cl_2_N_5_O_4_S 588.1239; found 588.1227; HPLC purity (254 nm): 93%.

#### 4.2.237 *tert*-Butyl 4-(4-Acetylbenzyl)piperazine-1-carboxylate (**47s70**)

4′-(Bromomethyl)acetophenone (2.00 g, 9.39 mmol) and Boc-piperazine (1.75 g, 9.39 mmol) were dissolved in acetonitrile (45 mL) and DMF (7 mL). K_2_CO_3_ (1.30 g, 9.39 mmol) was added, and the reaction mixture was stirred vigorously at 60 °C overnight. The solvent was removed under reduced pressure, and the residue was dissolved in EtOAc. The organic layer was washed two times with water, dried over Na_2_SO_4_, filtered, and concentrated to get a yellow solid (2.77 g, 93% yield). ^1^H NMR (400 MHz, DMSO-*d*_6_): δ 7.92 (d, *J* = 8.3 Hz, 2H), 7.45 (d, *J* = 8.3 Hz, 2H), 3.55 (s, 2H), 3.32 (m, 4H), 2.57 (s, 3H), 2.32 (t, *J* = 5.1 Hz, 4H), 1.39 (s, 9H); MS (ESI): *m*/*z* calcd for C_18_H_26_N_2_O_3_: 318.19. Found: 319.0 [M + H]^+^.

#### 4.2.238 *tert*-Butyl 4-(4-(1-Hydroxyethyl)benzyl)piperazine-1-carboxylate (**48s70**)

Ketone **47s70** (2.65 g, 8.32 mmol) was dissolved in dry EtOH (30 mL) and cooled to 0 °C. NaBH_4_ (315 mg, 8.32 mmol) was added in small portions, and the reaction mixture was stirred at 22 °C for 2 h. The solvent was removed under reduced pressure, and the residue was dissolved in EtOAc. The organic layer was washed with water and brine, dried over Na_2_SO_4_, filtered, and concentrated to get a yellow solid (2.53 g, 95% yield). ^1^H NMR (400 MHz, DMSO-*d*_6_): δ 7.28 (d, *J* = 8.0 Hz, 2H), 7.22 (d, *J* = 8.0 Hz, 2H), 5.09 (d, *J* = 4.2 Hz, 1H), 4.70 (m, 1H), 3.43 (s, 2H), 3.30 (m, 4H), 2.28 (m, 4H), 1.38 (s, 9H), 1.31 (d, *J* = 6.4 Hz, 3H); MS (ESI): *m*/*z* calcd for C_18_H_28_N_2_O_3_: 320.21. Found: 321.0 [M + H]^+^.

#### 4.2.239 *tert*-Butyl 4-(4-(1-(5-(Methoxycarbonyl)-2-nitrophenoxy)-ethyl)benzyl)piperazine-1-carboxylate (**49s70**)

Prepared according to the typical procedure H from **2s** (1.11 g, 5.62 mmol) and alcohol **48s70** (1.98 g, 6.19 mmol); brown oil (2.50 g, 89% yield). ^1^H NMR (400 MHz, DMSO-*d*_6_): δ 7.95 (d, *J* = 8.3 Hz, 1H), 7.71 (d, *J* = 1.5 Hz, 1H), 7.60 (dd, *J* = 8.3 Hz, 1.5 Hz, 1H), 7.39 (d, *J* = 8.2 Hz, 2H), 7.30 (d, *J* = 8.2 Hz, 2H), 5.86 (q, *J* = 6.2 Hz, 1H), 3.84 (s, 3H), 3.44 (s, 2H), 3.29 (m, 4H), 2.27 (m, 4H), 1.56 (d, *J* = 6.2 Hz, 3H), 1.38 (s, 9H); MS (ESI): *m*/*z* calcd for C_26_H_33_N_3_O_7_: 499.23. Found: 500.1 [M + H]^+^.

#### 4.2.240 *tert*-Butyl 4-(4-(1-(2-Amino-5-(methoxycarbonyl)phenoxy)-ethyl)benzyl)piperazine-1-carboxylate (**50s70**)

Prepared according to the typical procedure B from **49s70** (2.29 g, 4.57 mmol); orange oil (1.74 g, 81% yield). ^1^H NMR (400 MHz, DMSO-*d*_6_): δ 7.39 (d, *J* = 8.0 Hz, 2H), 7.30 (dd, *J* = 8.3 Hz, 1.5 Hz, 1H), 7.26 (d, *J* = 8.0 Hz, 2H), 7.20 (d, *J* = 1.5 Hz, 1H), 6.62 (d, *J* = 8.3 Hz, 1H), 5.69 (s, 2H), 5.46 (q, *J* = 6.2 Hz, 1H), 3.68 (s, 3H), 3.43 (s, 2H), 3.29 (s, 4H), 2.28 (s, 4H), 1.55 (d, *J* = 6.2 Hz, 3H), 1.38 (s, 9H); MS (ESI): *m*/*z* calcd for C_26_H_35_N_3_O_5_: 469.26. Found: 470.2 [M + H]^+^.

#### 4.2.241 Methyl 2-Amino-4-(1-(4-((4-(*tert*-butoxycarbonyl)piperazin-1-yl)methyl)phenyl)ethoxy)benzo[*d*]thiazole-6-carboxylate (**51s70**)

Prepared according to the typical procedure C from aniline **50s70** (1.69 g, 3.61 mmol); yellow solid (855 mg, 45% yield). ^1^H NMR (400 MHz, DMSO-*d*_6_): δ 7.94 (s, 2H), 7.88 (d, *J* = 1.5 Hz, 1H), 7.38 (d, *J* = 8.1 Hz, 2H), 7.29–7.23 (m, 3H), 5.76 (s, 1H), 5.70 (q, *J* = 6.3 Hz, 1H), 3.76 (s, 3H), 3.44 (s, 2H), 3.29 (s, 4H), 2.28 (s, 4H), 1.57 (d, *J* = 6.3 Hz, 3H), 1.38 (s, 9H); MS (ESI): *m*/*z* calcd for C_27_H_34_N_4_O_5_S: 526.22. Found: 527.2 [M + H]^+^.

#### 4.2.242 Methyl 4-(1-(4-((4-(*tert*-Butoxycarbonyl)piperazin-1-yl)methyl)phenyl)ethoxy)-2-(3,4-dichloro-5-methyl-1*H*-pyrrole-2-carboxamido)benzo[*d*]thiazole-6-carboxylate (**52s70**)

Prepared according to the typical procedure J from **51s70** (203 mg, 0.385 mmol); dark brown solid (100 mg, 37% yield); ^1^H NMR (400 MHz, DMSO-*d*_6_): δ 12.26 (s, 2H), 8.19 (s, 1H), 7.42 (d, *J* = 7.8 Hz, 2H), 7.40–7.38 (d, *J* = 1.1 Hz, 1H), 7.29 (d, *J* = 7.6 Hz, 2H), 5.80 (q, *J* = 6.4 Hz, 1H), 3.81 (s, 3H), 3.44 (s, 2H), 3.29 (m, 4H), 2.29 (m, 7H), 1.65 (d, *J* = 6.5 Hz, 3H), 1.38 (s, 9H); MS (ESI): *m*/*z* calcd for C_33_H_36_Cl_2_N_5_O_6_S: 700.18. Found: 700.2 [M – H]^−^.

#### 4.2.243 4-(*tert*-Butoxycarbonyl)-1-(4-(1-((6-carboxy-2-(3,4-dichloro-5-methyl-1*H*-pyrrole-2-carboxamido)benzo[*d*]thiazol-4-yl)oxy)ethyl)benzyl)piperazin-1-ium Chloride (**70×HCl**)

Prepared according to the typical procedure E from methyl ester **52s70** (80 mg, 0.115 mmol); dark brown powder (68 mg, 82% yield). ^1^H NMR (400 MHz, DMSO-*d*_6_) δ 12.87 (s, 1H), 12.33 (s, 1H), 12.26 (s, 1H), 10.22 (s, 1H), 8.17 (s, 1H), 7.57 (d, *J* = 8.2 Hz, 2H), 7.53 (d, *J* = 8.2 Hz, 2H), 7.37 (s, 1H), 5.87 (q, *J* = 6.3 Hz, 1H), 4.30 (s, 2H), 4.01 (d, *J* = 14.0 Hz, 2H), 3.25 (m, 2H), 3.11 (m, 2H), 2.99 (m, 2H), 2.29 (s, 3H), 1.66 (d, *J* = 6.3 Hz, 3H), 1.41 (s, 9H); HRMS (ESI) *m*/*z*: [M – Cl]^+^ calcd for C_32_H_36_Cl_2_N_5_O_6_S: 688.1763; found 688.1748; HPLC purity (254 nm): 81% (contains 13% of Boc-deprotected compound 69×HCl).

#### 4.2.244 1-(4-((1-Oxidothiomorpholino)methyl)phenyl)ethanone (**47s71**)

A mixture of 4′-(bromomethyl)acetophenone (639 mg, 3.00 mmol), K_2_CO_3_ (1.67 g, 12 mmol), and 4-thiomor-pholin-1-one hydrochloride (467 mg, 3 mmol) in MeCN (9 mL) was stirred at 22 °C overnight and then for 4 h at 40 °C. EtOAc and water were added, and the organic layer was washed with brine, dried over Na_2_SO_4_, filtered, and concentrated to get an off-white solid (738 mg, 98% yield). ^1^H NMR (400 MHz, CDCl_3_): δ 7.93 (d, *J* = 8.3 Hz, 2H), 7.43 (d, *J* = 8.3 Hz, 2H), 3.64 (s, 3H), 3.08 (m, 2H), 2.85 (m, 4H), 2.69 (m, 2H), 2.60 (s, 3H). MS (ESI): *m*/*z* calcd for C_13_H_17_NO_2_S: 251.10. Found: 251.9 [M + H]^+^.

#### 4.2.245 4-(4-(1-Hydroxyethyl)benzyl)thiomorpholine 1-Oxide (**48s71**)

Ketone **47s71** (738 mg, 2.94 mmol) was dissolved in MeOH (7.3 mL) and cooled to 0 °C and then NaBH_4_ (111 mg, 2.94 mmol) was added. After stirring for 30 min at 0 °C, the reaction mixture was allowed to warm to 22 °C and stirred for an additional 2.5 h. EtOAc and water were added, and the organic layer was washed with brine. The aqueous phase was additionally washed with dichloromethane; both organic phases were combined, dried over Na_2_SO_4_, filtered, and evaporated to give a white solid 488 mg (66% yield). ^1^H NMR (400 MHz, CDCl_3_): δ 7.35 (d, *J* = 8.1 Hz, 2H), 7.30 (d, *J* = 8.1 Hz, 2H), 7.30 (d, *J* = 8.1 Hz, 2H), 4.91 (q, *J* = 6.5 Hz, 1H), 3.57 (s, 2H), 3.06 (m, 1H), 2.94–2.76 (m, 3H), 2.69 (m, Hz, 1H), 1.83 (br s, 1H), 1.50 (d, *J* = 6.5 Hz, 3H); MS (ESI): *m*/*z* calcd for C_13_H_19_NO_2_S: 253.11. Found: 253.9 [M + H]^+^.

#### 4.2.246 Methyl 4-Nitro-3-(1-(4-((1-oxidothiomorpholino)methyl)-phenyl)ethoxy)benzoate (**49s71**)

Prepared according to the typical procedure H from **2s** (376 mg, 1.91 mmol) and alcohol **48s71** (532 mg, 2.10 mmol); white solid (322 mg, 39% yield). ^1^H NMR (400 MHz, CDCl_3_): δ 7.79 (d, *J* = 8.4 Hz, 1H), 7.66 (m, 3H), 7.60 (d, *J* = 1.5 Hz, 1H), 7.54 (d, *J* = 7.8 Hz, 2H), 5.59 (q, *J* = 6.4 Hz, 1H), 4.18 (m, 2H), 3.91 (s, 3H) 3.73 (m, 4H), 3.39 (m, 2H), 3.01 (d, *J* = 14.0 Hz, 2H), 1.70 (d, *J* = 6.4 Hz, 2H); MS (ESI): *m*/*z* calcd for C_21_H_24_N_2_O_6_S: 432.14. Found: 433.4 [M + H]^+^.

#### 4.2.247 Methyl 4-Amino-3-(1-(4-((1-oxidothiomorpholino)methyl)-phenyl)ethoxy)benzoate (**50s71**)

Prepared according to the typical procedure B from **49s71** (321 mg, 0.742 mmol); white solid (227 mg, 76% yield). ^1^H NMR (400 MHz, CDCl_3_): δ 7.48 (dd, *J* = 8.2 Hz, 1.8 Hz, 1H), 7.39–7.34 (m, 2H), 7.28 (d, *J* = 8.2 Hz, 2H), 6.65 (d, *J* = 8.2 Hz, 1H), 5.42 (q, *J* = 6.4 Hz, 1H), 4.30 (br s, 2H), 3.79 (s, 3H), 3.55 (s, 2H), 3.05 (m, 2H), 2.85 (m, 4H), 2.68 (m, 2H), 1.66 (d, *J* = 6.4 Hz, 3H) ppm; MS (ESI): *m*/*z* calcd for C_21_H_26_N_2_O_4_S: 402.16. Found: 402.9 [M + H]^+^.

#### 4.2.248 Methyl 2-Amino-4-(1-(4-((1-oxidothiomorpholino)methyl)-phenyl)ethoxy)benzo[*d*]thiazole-6-carboxylate (**51s71**)

Prepared according to the typical procedure C from aniline **50s71** (225 mg, 0.559 mmol); off-white solid (95 mg, 37% yield). ^1^H NMR (400 MHz, DMSO-*d*_6_): δ 7.94 (s, 2H), 7.87 (s, 1H), 7.39 (d, *J* = 7.9 Hz, 2H), 7.28 (d, *J* = 7.9 Hz, 2H), 7.25 (s, 1H), 5.71 (q, *J* = 6.3 Hz, 1H), 3.76 (s, 3H), 3.51 (s, 2H), 2.88–2.77 (m, 4H), 2.70 (m, 2H), 2.62–2.54 (m, 2H), 1.57 (d, *J* = 6.3 Hz, 2H); MS (ESI): *m*/*z* calcd for C_22_H_25_N_3_O_4_S_2_: 459.13. Found: 460.0 [M + H]^+^.

#### 4.2.249 Methyl 2-(3,4-Dichloro-5-methyl-1*H*-pyrrole-2-carboxamido)-4-(1-(4-((1-oxidothiomorpholino)methyl)phenyl)ethoxy)benzo[*d*]thiazole-6-carboxylate (**52s71**)

Prepared according to the typical procedure J from **51s71** (97 mg, 0.211 mmol); black powder (71 mg, 53% yield). ^1^H NMR (400 MHz, DMSO-*d*_6_): δ 12.30 (br s, 1H), 12.27 (br s, 1H), 8.20 (s, 1H), 7.44 (d, *J* = 8.0 Hz, 2H), 7.40 (s, 1H), 7.31 (d, *J* = 8.0 Hz, 2H), 5.81 (q, *J* = 6.2 Hz, 1H), 3.82 (s, 3H), 3.55 (s, 2H), 2.86 (m, 4H), 2.72 (m, 2H), 2.64 (m, 2H), 2.29 (s, 3H), 1.65 (d, *J* = 6.2 Hz, 3H); MS (ESI): *m*/*z* calcd for C_28_H_28_Cl_2_N_4_O_5_S_2_: 634.09. Found: 634.9 [M + H]^+^.

#### 4.2.250 2-(3,4-Dichloro-5-methyl-1*H*-pyrrole-2-carboxamido)-4-(1-(4-((1-oxidothiomorpholino)methyl)phenyl)ethoxy)benzo-[*d*]thiazole-6-carboxylic Acid (**71**)

Prepared according to the typical procedure E from methyl ester **52s71** (70 mg, 0.110 mmol); black powder (38 mg, 56% yield). ^1^H NMR (400 MHz, DMSO-*d*_6_): δ 12.84 (br s, 1H), 12.26 (br s, 2H), 8.14 (s, 1H), 7.42 (d, *J* = 7.7 Hz, 2H), 7.37 (s, 1H), 7.30 (d, *J* = 7.7 Hz, 2H), 5.78 (q, *J* = 6.2 Hz, 1H), 3.52 (s, 2H), 2.88–2.80 (m, 4H), 2.74–2.64 (m, 4H), 2.28 (s, 3H), 1.64 (d, *J* = 6.2 Hz, 3H); ^13^C NMR (101 MHz, DMSO-*d*_6_): δ 166.96, 159.68, 156.72, 149.02, 142.08, 141.33, 137.20, 132.82, 129.93, 129.09, 126.55, 125.56, 116.95, 116.00, 115.71, 110.56, 109.98, 75.46, 61.16, 45.47, 43.64, 24.21, 11.09; HRMS (ESI) *m*/*z*: [M + H]^+^ calcd for C_27_H_27_Cl_2_N_4_O_5_S_2_ 621.0800; found 621.0788; HPLC purity (254 nm): 92%.

#### 4.2.251 1-(4-((1,1-Dioxidothiomorpholino)methyl)phenyl)ethan-1-one (**47s72**)

4′-(Bromomethyl)acetophenone (1.16 g, 5.44 mmol) and thiomorpholine 1,1-dioxide (735 mg, 5.44 mmol) were dissolved in acetonitrile (35 mL) and DMF (0.5 mL). K_2_CO_3_ (1.51 g, 10.9 mmol) was added, and the reaction mixture was stirred at 60 °C overnight. The volatiles were removed under reduced pressure, the residue was dissolved in EtOAc, and the organic layer was washed with water, dried over Na_2_SO_4_, filtered, and evaporated to get the product as yellow crystals (990 mg, 68% yield). ^1^H NMR (400 MHz, DMSO-*d*_6_): δ 7.93 (d, *J* = 8.3 Hz, 2H), 7.49 (d, *J* = 8.3 Hz, 2H), 3.75 (s, 2H), 3.20–3.05 (m, 4H), 2.88 2.92–2.84 (m, 4H), 2.57 (s, 3H); MS (ESI): *m*/*z* calcd for C_13_H_17_NO_3_S: 267.09. Found: 267.8 [M + H]^+^.

#### 4.2.252 4-(4-(1-Hydroxyethyl)benzyl)thiomorpholine 1,1-Dioxide (**48s72**)

A solution of ketone **47s72** (990 mg, 3.70 mmol) in dry ethanol (13 mL) was cooled to 0 °C, followed by the addition of NaBH_4_ (140 mg, 3.70 mmol) in small portions. The reaction was stirred at 22 °C for 2.5 h, the solvent was removed under reduced pressure, and the residue was dissolved in EtOAc. The organic layer was washed with water and brine, dried over Na_2_SO_4_, filtered, and concentrated to get a yellow oil (828 mg, 83% yield). ^1^H NMR (400 MHz, DMSO-*d*_6_): δ 7.30 (d, *J* = 8.1 Hz, 2H), 7.26 (d, *J* = 8.1 Hz, 2H), 5.10 (d, *J* = 4.2 Hz, 1H), 4.70 (dq, *J* = 4.2 Hz, 6.4 Hz, 1H), 3.63 (s, 2H), 3.09 (m, 4H), 2.84 (m, 4H), 1.31 (d, *J* = 6.4 Hz, 3H); MS (ESI): *m*/*z* calcd for C_13_H_19_NO_3_S: 269.11. Found: 269.9 [M + H]^+^.

#### 4.2.253 Methyl 3-(1-(4-((1,1-Dioxidothiomorpholino)methyl)phenyl)ethoxy)-4-nitrobenzoate (**49s72**)

Prepared according to the typical procedure H from **2s** (442 mg, 2.24 mmol) and alcohol **48s72** (665 mg, 2.47 mmol); orange oil (855 mg, 85% yield). ^1^H NMR (400 MHz, DMSO-*d*_6_): δ 7.95 (d, *J* = 8.3 Hz, 1H), 7.72 (d, *J* = 1.5 Hz, 1H), 7.60 (dd, *J* = 8.3 Hz, 1.5 Hz, 1H), 7.41 (d, *J* = 8.2 Hz, 2H), 7.34 (d, *J* = 8.2 Hz, 2H), 5.87 (q, *J* = 6.2 Hz, 1H), 3.85 (s, 3H), 3.64 (s, 2H), 3.09 (m, 4H), 2.84 (m, 4H), 1.56 (d, *J* = 6.2 Hz, 3H); MS (ESI): *m*/*z* calcd for C_21_H_24_N_2_O_7_S: 448.13. Found: 449.0 [M + H]^+^.

#### 4.2.254 Methyl 4-Amino-3-(1-(4-((1,1-dioxidothiomorpholino)methyl)phenyl)ethoxy)benzoate (**50s72**)

Prepared according to the typical procedure B from **49s72** (810 mg, 1.81 mmol); yellow solid (121 mg, 16% yield). ^1^H NMR (400 MHz, DMSO-*d*_6_): δ 7.41 (d, *J* = 8.0 Hz, 2H), 7.30 (d, *J* = 8.0 Hz, 3H), 7.21 (d, *J* = 1.5 Hz, 1H), 6.62 (d, *J* = 8.2 Hz, 1H), 5.69 (s, 2H), 5.47 (q, *J* = 6.1 Hz, 1H), 3.68 (s, 3H), 3.63 (s, 2H), 3.13–3.05 (s, 4H), 2.89–2.81 (s, 4H), 1.55 (d, *J* = 6.1 Hz, 3H); MS (ESI): *m*/*z* calcd for C_21_H_26_N_2_O_5_S: 418.16. Found: 419.0 [M + H]^+^.

#### 4.2.255 Methyl 2-Amino-4-(1-(4-((1,1-dioxidothiomorpholino)methyl)phenyl)ethoxy)benzo[*d*]thiazole-6-carboxylate (**51s72**)

Prepared according to the typical procedure C from aniline **50s72** (130 mg, 0.310 mmol); light yellow solid (93 mg, 63% yield). ^1^H NMR (400 MHz, DMSO-*d*_6_): δ 7.95 (s, 2H), 7.88 (s, 1H), 7.40 (d, *J* = 6.1 Hz, 2H), 7.29 (m, 3H), 5.73 (m, 1H), 3.76 (s, 3H), 3.64 (s, 2H), 3.10 (s, 4H), 2.85 (s, 4H), 1.58 (s, 3H); MS (ESI): *m*/*z* calcd for C_22_H_25_N_3_O_5_S_2_: 475.12. Found: 476.1 [M + H]^+^.

#### 4.2.256 Methyl 2-(3,4-Dichloro-5-methyl-1*H*-pyrrole-2-carboxamido)-4-(1-(4-((1,1-dioxidothiomorpholino)methyl)phenyl)-ethoxy)benzo[*d*]thiazole-6-carboxylate (**52s72**)

Prepared according to the typical procedure J from **51s72** (50 mg, 0.106 mmol); gray solid (33 mg, 48% yield). ^1^H NMR (400 MHz, DMSO-*d*_6_): δ 12.30 (s, 1H), 12.27 (s, 1H), 8.20 (s, 1H), 7.44 (d, *J* = 7.9 Hz, 2H), 7.40 (s, 1H), 7.32 (d, *J* = 7.9 Hz, 2H), 5.81 (q, *J* = 6.4 Hz, 1H), 3.82 (s, 3H), 3.63 (s, 2H), 3.14–3.04 (m, 4H), 2.91–2.80 (m, 4H), 2.29 (s, 3H), 1.65 (d, *J* = 6.4 Hz, 3H); MS (ESI): *m*/*z* calcd for C_28_H_28_Cl_2_N_4_O_6_S_2_: 650.08. Found: 651.2 [M + H]^+^.

#### 4.2.257 4-(4-(1-((6-Carboxy-2-(3,4-dichloro-5-methyl-1*H*-pyrrole-2-carboxamido)benzo[*d*]thiazol-4-yl)oxy)ethyl)benzyl)thio-morpholin-4-ium 1,1-Dioxide Chloride (**72×HCl**)

Prepared according to the typical procedure E from methyl ester **52s72** (30 mg, 0.046 mmol); gray solid (19 mg, 61% yield). ^1^H NMR (400 MHz, DMSO-*d*_6_): δ 12.87 (s, 1H), 12.29 (s, 1H), 12.26 (s, 1H), 8.16 (s, 1H), 7.58–7.41 (m, 4H), 7.37 (s, 1H), 5.83 (q, *J* = 6.4, 5.9 Hz, 1H), 2.29 (s, 3H), 1.65 (d, *J* = 6.3 Hz, 3H); some peaks overlaid by DMSO and water signals; HRMS (ESI) *m*/*z*: [M – Cl]^+^ calcd for C_27_H_27_Cl_2_N_4_O_6_S_2_ 637.0749; found 637.0739; HPLC purity (254 nm): 96%.

#### 4.2.258 4-(4-Acetylbenzyl)morpholin-3-one (**47s73**)

Sodium hydride (60% dispersion in mineral oil, 208 mg, 5.20 mmol) was added to a stirred solution of morpholin-3-one (404 mg, 4.00 mmol) in DMF (12 mL) at 0 °C. After the addition of 4′-(bromomethyl)acetophenone (852 mg, 4.00 mmol), the reaction mixture was stirred at 22 °C overnight. EtOAc was added and the resulting solution was washed with water and brine, dried over Na_2_SO_4_, filtered, and concentrated to give a yellow oil (903 mg, 97% yield). ^1^H NMR (400 MHz, CDCl_3_): δ 7.94 (d, *J* = 8.1 Hz, 2H), 7.37 (d, *J* = 8.1 Hz, 2H), 4.68 (s, 2H), 4.27 (s, 2H), 3.87 (dd, *J* = 5.8 Hz, 4.5 Hz, 2H), 3.30 (dd, *J* = 5.8 Hz, 4.5 Hz, 2H), 2.60 (s, 3H); MS (ESI): *m*/*z* calcd for C_13_H_15_NO_3_: 233.11. Found: 234.1 [M + H]^+^.

#### 4.2.259 4-(4-(1-Hydroxyethyl)benzyl)morpholin-3-one (**48s73**)

Sodium borohydride (7.74 mmol, 293 mg) was added to a solution of ketone **47s73** (903 mg, 3.87 mmol) in methanol (6 mL) and cooled to 0 °C. After 30 min at 0 °C, the reaction mixture was stirred at 22 °C for 3 h. EtOAc was added, and the resulting solution was washed with brine, dried over Na_2_SO_4_, filtered, and concentrated to give a colorless oil (877 mg, 96% yield). ^1^H NMR (400 MHz, CDCl_3_): δ 7.36 (d, *J* = 7.8 Hz, 2H), 7.26 (d, *J* = 7.8 Hz, 2H), 4.91 (dt, *J* = 11.0, 6.4 Hz, 1H), 4.62 (s, 2H), 4.25 (s, 2H), 3.84 (m, 2H), 3.27 (m, 2H), 1.50 (d, *J* = 6.4 Hz, 3H); MS (ESI): *m*/*z* calcd for C_13_H_17_NO_3_: 235.12. Found: MS 236.1 [M + H]^+^.

#### 4.2.260 Methyl 4-Nitro-3-(1-(4-((3-oxomorpholino)methyl)phenyl)-ethoxy)benzoate (**49s73**)

Prepared according to the typical procedure H from **2s** (723 mg, 3.67 mmol) and alcohol **48s73** (949 g, 4.03 mmol); yellow oil (380 mg, 25% yield). ^1^H NMR (400 MHz, CDCl_3_): δ 7.76 (d, *J* = 8.3 Hz, 1H), 7.65–7.59 (m, 2H), 7.40 (d, *J* = 8.3 Hz, 2H), 7.26 (d, *J* = 8.1 Hz, 1H), 5.55 (q, *J* = 6.4 Hz, 2H), 4.63 (d, *J* = 14.8 Hz, 1H), 4.57 (d, *J* = 14.8 Hz, 1H), 4.25 (s, 2H), 3.90 (s, 3H), 3.87–3.82 (m, 2H), 3.29–3.25 (m, 2H), 1.68 (d, *J* = 6.4 Hz, 3H) ppm; MS (ESI): *m*/*z* calcd for C_21_H_22_N_2_O_7_: 414.14. Found: 414.9 [M + H]^+^.

#### 4.2.261 Methyl 4-Amino-3-(1-(4-((3-oxomorpholino)methyl)phenyl)ethoxy)benzoate (**50s73**)

Prepared according to the typical procedure B from **49s73** (384 mg, 0.928 mmol); yellow solid (296 mg, 83% yield). ^1^H NMR (400 MHz, CDCl_3_): δ 7.48 (d, *J* = 8.2 Hz, 1H), 7.36 (d, *J* = 8.0 Hz, 4H), 7.24 (d, *J* = 8.0 Hz, 3H), 6.65 (d, *J* = 8.2 Hz, 1H), 5.42 (q, *J* = 6.4 Hz, 1H), 4.63 (d, *J* = 14.6 Hz, 1H), 4.57 (d, *J* = 14.6 Hz, 1H), 4.28 (s, 2H), 4.24 (s, 2H), 3.84 (t, *J* = 5.1 Hz, 2H), 3.80 (s, 3H), 3.27 (t, *J* = 5.1 Hz, 2H), 1.65 (d, *J* = 6.4 Hz, 3H); MS (ESI): *m*/*z* calcd for C_21_H_24_N_2_O_5_: 384.17. Found: 384.9 [M + H]^+^.

#### 4.2.262 Methyl 2-Amino-4-(1-(4-((3-oxomorpholino)methyl)phenyl)ethoxy)benzo[*d*]thiazole-6-carboxylate (**51s73**)

Prepared according to the typical procedure C from aniline **50s73** (298 mg, 0.775 mmol); orange powder (229 mg, 67% yield). ^1^H NMR (400 MHz, DMSO-*d*_6_): δ 7.94 (s, 2H), 7.87 (d, *J* = 1.6 Hz, 1H), 7.41 (d, *J* = 7.9 Hz, 2H), 7.27–7.20 (m, 3H), 5.73 (q, *J* = 6.4 Hz, 1H), 4.55–4.44 (m, 2H), 4.09 (s, 2H), 3.79 (t, *J* = 5.2 Hz, 2H), 3.76 (s, 3H), 3.23 (t, *J* = 5.2 Hz, 2H), 1.56 (d, *J* = 6.4 Hz, 3H); MS (ESI): *m*/*z* calcd for C_22_H_23_N_3_O_5_S: 441.14. Found: 441.9 [M + H]^+^.

#### 4.2.263 Methyl 2-(3,4-Dichloro-5-methyl-1*H*-pyrrole-2-carboxamido)-4-(1-(4-((3-oxomorpholino)methyl)phenyl)ethoxy)benzo[*d*]thiazole-6-carboxylate (**52s73**)

Prepared according to the typical procedure J from **51s73** (210 mg, 0.475 mmol); brown powder (132 mg, 45% yield). ^1^H NMR (400 MHz, DMSO-*d*_6_): δ 12.29 (br s, 1H), 12.26 (br s, 1H), 8.19 (s, 1H), 7.45 (d, *J* = 7.8 Hz, 2H), 7.40 (s, 1H), 7.24 (d, *J* = 7.8 Hz, 2H), 5.81 (q, *J* = 5.7 Hz, 1H), 4.57–4.45 (m, 2H), 4.09 (s, 2H), 3.80 (m, 5H), 3.26–3.22 (m, 2H), 2.28 (s, 3H), 1.64 (d, *J* = 6.4 Hz, 3H); MS (ESI): *m*/*z* calcd for C_28_H_26_Cl_2_N_4_O_6_S: 616.10. Found: 617.2 [M + H]^+^.

#### 4.2.264 2-(3,4-Dichloro-5-methyl-1*H*-pyrrole-2-carboxamido)-4-(1-(4-((3-oxomorpholino)methyl)phenyl)ethoxy)benzo[*d*]thiazole-6-carboxylic Acid (**73**)

Prepared according to the typical procedure E from methyl ester **52s73** (125 mg, 0.202 mmol); brown solid (15 mg, 12% yield). ^1^H NMR (400 MHz, DMSO-*d*_6_): δ 12.28 (s, 3H), 8.14 (s, 1H), 7.42 (d, *J* = 7.9 Hz, 2H), 7.37 (s, 1H), 7.30 (d, *J* = 7.9 Hz, 2H), 5.78 (q, *J* = 6.5 Hz, 1H), 3.52 (s, 2H), 2.92–2.78 (m, 4H), 2.76–2.65 (m, 2H), 2.28 (s, 3H), 1.64 (d, *J* = 6.3 Hz, 3H); HRMS (ESI) *m*/*z*: [M – H]^−^ calcd for C_27_H_23_Cl_2_N_4_O_6_S 601.0715; found 601.0725; HPLC purity (254 nm): 93%.

### 4.3 X-ray Crystallography

#### 4.3.1 Protein Expression and Purification

*E. coli* GyrB24 protein, purified as described previously,^41^ was concentrated to approximately 11 mg/mL in 50 mM of tris–HCl pH 7.9, 50 mM NaCl, 5 mM DTT. This construct corresponds to residues 1–220 of the full-length wild-type protein (UniProtKB entry P0AES6), with a calculated molecular weight of 24,157 Da, and is referred to as EcGyrB24.

The equivalent ATPase subdomain from *A. baumannii* 1419130 DNA gyrase B, corresponding to residues 28–233 of the full-length wild-type protein (UniProtKB entry A0A009KIJ4), was cloned into a modified pET28 vector and expressed in T7 Express *E. coli* cells (New England BioLabs) with an N-terminal His-tag. This was purified using a Ni-chelate column, and the tag was cleaved off using TEV protease before further purification on a second Ni-chelate column and a monoQ ion exchange column. The protein was concentrated to approximately 14 mg/mL in 50 mM of tris–HCl pH 7.5, 1 mM EDTA, 1 mM DTT. The resulting protein had a calculated molecular weight of 22,741 Da and is referred to as AbGyrB23.

The equivalent ATPase subdomain from *P. aeruginosa* PAO1 DNA gyrase B, corresponding to residues 1–221 of the full-length wild-type protein (UniProtKB entry Q9I7C2), was cloned into a modified pTTQ18 vector and expressed in T7 Express *E. coli* cells (New England BioLabs) without an affinity tag. This was purified using successive Q-sepharose, monoQ, and phenyl-sepharose columns. The protein was concentrated to approximately 10 mg/mL in 50 mM of tris–HCl pH 7.5, 10% (v/v) glycerol, 1 mM EDTA, 1 mM DTT. The resulting protein had a calculated molecular weight of 24,502 Da and is referred to as PaGyrB24.

#### 4.3.2 Crystallization, X-ray Data Collection, and Structure Solution

Crystals were grown using the vapor diffusion method from proteins at the aforementioned concentrations in the presence of 1 mM ligand. Commercially available (Molecular Dimensions, Qiagen) and in-house crystallization screens were set up in MRC2 96-well crystallization plates (Swissci) with drops comprised of 0.3 μL of precipitant and 0.3 μL of protein solution using an Oryx 8 liquid handling robot (Douglas Instruments) and then equilibrated against 50 μL of reservoir solution at a constant temperature of 19 °C. In most cases, the optimization of initial hits was required to obtain suitable crystals, which were cryoprotected as necessary and mounted in Litholoops (Molecular Dimensions) before flash-cooling by plunging into liquid nitrogen prior to transport to the synchrotron. X-ray data sets were recorded from single crystals on beamline I03, I04, I04-1, or I24 at the Diamond Light Source (Oxfordshire, U.K.) using an Eiger2 XE 16M, a Pilatus 6M, or a Pilatus 6M-F hybrid photon counting detector (Dectris), with crystals maintained at 100 K by a Cryojet cryocooler (Oxford Instruments).

X-ray data were integrated and scaled using DIALS^42^ via the XIA2 expert system^43^ and merged using AIMLESS^44^ (data statistics are shown in Tables S12–S14). All successive data processing was carried out using programs in the CCP4 suite via the CCP4i2 graphical user interface.^45^ All structures were solved via molecular replacement in PHASER.^46^ Where the template structure was of the same protein as the target structure, the protein component of one GyrB domain was used directly as the input to PHASER, and the resulting models were finalized by successive iterations of model building in COOT^47^ and restrained refinement in REFMAC5^48^ until no further improvements could be achieved. Where the template structure was from a related protein to the target structure, an input model for PHASER was prepared from one subunit of the template structure by removing nonconserved side chains using SCULPTOR^49^ with reference to an alignment of the template and target sequences. Furthermore, in these cases, the PHASER solution was automatically rebuilt using BUCCANEER^50^ prior to finalizing with COOT and REFMAC. Starting coordinates and restraints for the various ligands were generated using AceDRG^51^ before docking these into a suitable electron density. Final models were validated using MOLPROBITY^52^ and the PDB-validation server (https://validate-rcsb-2.wwpdb.org). Refinement and validation statistics for all models are summarized in Tables S12–S14.

Crystals of the EcGyrB24-**1** complex were obtained using a precipitant comprised of 33% (w/v) PEG 4000, 100 mM tris–HCl pH 8.0, 75 mM MgCl_2_, and these were cryoprotected using the same solution supplemented with 17.5% (v/v) glycerol and ∼1 mM **1**. Data were recorded to a 1.16 Å resolution in space group C2. The structure was solved by molecular replacement using a nonisomorphous structure of EcGyrB24 (PDB accession code 1KZN), giving a single copy of the protein chain in the asymmetric unit (ASU) with an estimated solvent content of 47%.

Crystals of the EcGyrB24-**7** complex were obtained using a precipitant comprised of 34% (w/v) PEG 4000, 100 mM tris–HCl pH 8.0, 128 mM MgCl_2_. A similar solution was used for the cryoprotectant, although the PEG 4000 concentration was increased to 40% (w/v) and the mixture was supplemented with ∼1 mM **7**. This time, the space group was *P*2_1_2_1_2_1_ and data were collected to a 1.65 Å resolution. Again, the structure was solved by molecular replacement using the same EcGyrB24 structure (PDB accession code 1KZN), giving a single copy of the protein chain in the ASU and an estimated solvent content of 44%.

Crystals of the AbGyrB23-novobiocin complex grew from 0.2 M Mg(NO_3_)_2_, 20% (w/v) PEG 3350, and were cryoprotected with this solution supplemented with 20% (v/v) ethylene glycol. Data were collected in space group *P*4_1_2_1_2 to a resolution of 1.9 Å, and the structure was solved by molecular replacement using a template derived from another EcGyrB24 structure (PDB accession code 6YD9; the corresponding domains share a 73% sequence identity), giving a single copy of the protein chain in the ASU, with an estimated solvent content of 52%.

Crystals of the AbGyrB23-**27** complex were obtained from 30% (w/v) PEG 3350, 0.2 M MgCl_2_, and 10% (v/v) of ethylene glycol was added to this solution for cryoprotection. The complex crystallized in space group C2, and data were taken to a 1.6 Å resolution. The above model of the AbGyrB23-novobiocin complex (PDB accession code 7PQI) was used as the input template for molecular replacement, which gave two copies of the domain per ASU, corresponding to an estimated solvent content of 45%.

Crystals of the AbGyrB23-**(*****S*****)*****-*****27** complex were produced using a precipitant comprised of 31% (w/v) PEG 3350, 0.2 M calcium acetate, and cryoprotected using this solution with the addition of 10% (v/v) of ethylene glycol. These crystals were isomorphous with the AbGyrB23-**27** complex above, and the structure was solved in the same way using data to a 1.55 Å resolution.

Crystals of the PaGyrB24-novobiocin complex grew from 12.5% (v/v) MPD, 12.5% (v/v) PEG 1000, 25% (w/v) PEG 3350, 30 mM MgCl_2_, 30 mM CaCl_2_, and 0.1 M imidazole/MES pH 6.5, and these could be flash-cooled directly without additional cryoprotectant. Data were collected in space group *P*2_1_ to a resolution of 1.32 Å, and the structure was solved by molecular replacement using a template derived from the EcGyrB43 structure (PDB accession code 6XTJ; the corresponding domains share a 74% sequence identity), giving three copies of the protein chain in the ASU, with an estimated solvent content of 44%.

Crystals of the PaGyrB24-**(*****S*****)*****-*****27** complex were obtained from 22% (w/v) PEG 3350, 0.1 M HEPES pH 7, and cryoprotected using this solution with the addition of 20% (v/v) of ethylene glycol. This complex also crystallized in space group *P*2_1_ but was not isomorphous with the PaGyrB24-novobiocin complex. A single protein chain from the latter (PDB accession code 7PTF) was used as the molecular replacement template using data collected to a 2.2 Å resolution. This gave two copies of the domain per ASU corresponding to an estimated solvent content of 37%.

### 4.4 Microtiter-Plate-Based Assays for Inhibition of *E. coli*, *P. aeruginosa*, and *A. baumannii* Gyrase Supercoiling and *E. coli* Topo IV Relaxation

Commercially available assay kits from Inspiralis for the determination of IC_50_ values of the test compounds for the inhibition of DNA gyrase supercoiling and topoisomerase IV relaxation were used, as described in ref.^11^^11^

### 4.5 Gel-Based Assays for Inhibition of *E. coli* Gyrase Supercoiling, *E. coli* Gyrase Relaxation, *E. coli* Gyrase Cleavage, and Inhibition of *A. baumannii* and *P. aeruginosa* Topo IV Decatenation

In all experiments, the activity of the enzymes was determined prior to the testing of the compounds and 1 unit (U) was defined as the amount of enzyme required to just fully supercoil, relax, reach the maximum cleavage of the substrate, or fully decatenate the substrate. This amount of enzyme was initially used in the determination of control inhibitor activity. The experiments were performed in duplicate. For all assays, the final DMSO concentration was 1%. Bands were visualized by ethidium staining for 20 min and destaining for 20 min. Gels were scanned using documentation equipment (GeneGenius, Syngene, Cambridge, U.K.), and % inhibition levels were obtained with gel scanning software (GeneTools, Syngene, Cambridge, U.K.).

#### 4.5.1 Inhibition of *E. coli* Gyrase Supercoiling

DNA gyrase (1 U) was incubated with 0.5 μg of relaxed pBR322 DNA in a 30 μL reaction (containing the test compound in 0.001, 0.005, 0.01, 0.05, 0.1, 0.5, 1.0, 5.0, 10, 25, 50, and 100 μM final concentrations) at 37 °C for 30 min under the following conditions: 35 mM tris–HCl (pH 7.5), 24 mM KCl, 4 mM MgCl_2_, 2 mM DTT, 1.8 mM Spermidine, 1 mM ATP, 6.5% (w/v) glycerol, and 0.1 mg/mL BSA. Each reaction was stopped by the addition of 30 μL of chloroform/*iso*-amyl alcohol (26:1) and 20 μL of Stop Dye (40% sucrose, 100 mM tris–HCl (pH 7.5), 10 mM EDTA, 0.5 μg/mL bromophenol blue), before being loaded on a 1.0% TAE (tris acetate 0.04 mM, EDTA 0.002 mM) gel run at 80 V for 2 h.

#### 4.5.2 *E. coli* DNA Relaxation

DNA gyrase (1 U) was incubated with 0.5 μg of supercoiled pBR322 DNA in a 30 μL reaction (containing the test compound in 0.001, 0.005, 0.01, 0.05, 0.1, 0.5, 1.0, 5.0, 10, 25, 50, and 100 μM final concentrations) at 37 °C for 2 h under the following conditions: 35 mM tris–HCl (pH 7.5), 24 mM KCl, 4 mM MgCl_2_, 2 mM DTT, 1.8 mM Spermidine, 6.5% (w/v) glycerol, and 0.1 mg/mL BSA. Each reaction was stopped by the addition of 30 μL of chloroform/*iso*-amyl alcohol (26:1) and 20 μL of Stop Dye (40% sucrose, 100 mM tris–HCl (pH 7.5), 10 mM EDTA, 0.5 μg/mL bromophenol blue), before being loaded on a 1.0% TAE (tris acetate 0.04 mM, EDTA 0.002 mM) gel run at 80 V for 2 h.

#### 4.5.3 *E. coli* DNA Gyrase Cleavage Assay

Gyrase (1 U) was incubated with 0.5 μg of supercoiled pBR322 in a reaction volume of 30 μL at 37 °C for 1 h in assay buffer (see above) minus the ATP, in the presence of test compound. SDS (0.2%) and 0.1 mg/mL of proteinase K were added before a further incubation at 37 °C for 30 min. Each reaction was stopped by the addition of 30 μL of chloroform/*iso*-amyl alcohol (26:1) and 20 μL of Stop Dye (40% sucrose, 100 mM tris–HCl (pH 7.5), 10 mM EDTA, 0.5 μg/mL bromophenol blue), before being loaded on a 1.0% TAE (tris acetate 0.04 mM, EDTA 0.002 mM) gel run at 80 V for 2 h.

#### 4.5.4 Inhibition of *A. baumannii* and *P. aeruginosa* Topo IV Decatenation

Topo IV (1 U) was incubated with 0.2 μg of kDNA in a 30 μL reaction (containing the test compound in 0.001, 0.005, 0.01, 0.05, 0.1, 0.5, 1.0, 5.0, 10, 25, 50, and 100 μM final concentrations) at 37 °C for 30 min under the following conditions: 50 mM HEPES–KOH (pH 7.9), 6 mM magnesium acetate, 4 mM DTT, 1 mM ATP, 100 mM potassium glutamate, 2 mM spermidine, and 0.05 mg/mL albumin. Ciprofloxacin and novobiocin at a 5 μM final concentration were used as controls.

Each reaction was stopped by the addition of 30 μL chloroform/*iso*-amyl alcohol (24:1) and 30 μL Stop Dye (40% sucrose (w/v), 100 mM tris–HCl (pH 7.5), 10 mM EDTA, 0.5 μg/mL bromophenol blue), before being loaded on a 1.0% TAE gel run at 80 V for 2 h. Due to time constraints, all compounds were assayed at the higher test range of 100 μM only, but the data points were fitted up to 10 μM (apart from the controls) to improve the graph fits.

### 4.6 Microbiology

#### 4.6.1 Susceptibility Testing

MICs for susceptibility testing and MIC_90_ assays were determined according to CLSI standards for liquid MIC (M07) using the direct colony suspension method for inoculum preparation. Appropriate inoculation suspension density was assessed using a Sensititre Nephelometer (Thermo Fisher Scientific) with a Sensititre 0.5 McFarland Standard. MBC and time-kill assays were conducted according to CLSI guidelines (M26). MIC in the presence of serum was done using a 50% (final concentration) pooled, heat-inactivated (56 °C, 1 h), sterile-filtered (Filtropur S plus 0.2, Sarstedt) human serum.

#### 4.6.2 Frequency of Resistance and Whole Genome Sequencing

Frequency of resistance was determined by plating dilutions of freshly grown cultures onto freshly poured MH-II plates containing concentrations of the test compounds corresponding to 4- or 8-times the MIC. Serial dilutions of the starting cultures were plated nonselectively on MH-II plates to determine the initial culture density. Colonies were counted on selection plates after 24 and 48 h, and the number of colonies after 48 h was divided by the calculated number of cells plated to yield the frequency of resistance. Colonies that grew were restreaked on plates with identical concentrations of the test compounds that they were selected on. Reduced susceptibility of the clones was confirmed by MIC testing.

Genomic DNA was extracted from resistant isolates using an Epicentre Masterpure Complete DNA & RNA Purification kit (Lucigen). Genomic DNA was used to prepare genomic libraries for whole genome sequencing using a Nextera XT Library Preparation Kit (Illumina) and Nextera Indexes according to the manufacturer’s protocols. Libraries were sequenced using a Miseq device (Illumina), and the resulting sequences were analyzed using CLC Genomics Workbench V11 (Qiagen).

#### 4.6.3 Hemolysis

Hemolysis was tested as previously described by DeRosa et al.^53^

### 4.7 Cytotoxicity

#### 4.7.1 Lactate Dehydrogenase Assay and MTS Assay

Lactate dehydrogenase (LDH) assay^15^ and MTS assay^17^ were performed as described.

In vitro fluorometric microculture cytotoxicity (FMC) assay was performed according to the published procedure.^18^

### 4.8 Genotoxicity and Mutagenicity

#### 4.8.1 In Vitro Cell Micronucleus Test

The protocol followed the recommendations of the Test Guideline 487 of the OECD guideline for the testing of chemicals.^54^ The test was performed on rodent CHO cells (ECACC ref: 85050302). The cells were seeded at a density of 2000/well in a black 96-well plate with a clear bottom and were incubated in a humidified atmosphere at 37 °C with 5% CO_2_. To estimate the micronuclei frequency, the cells scored must have completed one mitosis during the treatment or the post-treatment incubation period. Compounds were prepared at 10 mM in 100% DMSO and were assayed at 100, 50, 25, 12.5, and 6.25 μM for 24 h in six replicates. DMSO did not exceed 1% according to TG-487. Mitomycin C (MitC, Sigma-Aldrich), a known inducer of micronuclei formation, was the positive control used to demonstrate the sensitivity of the test, and cells untreated were used as a negative control. After treatment, cytochalasin B (cytoB) was used as a cytokinesis-blocker of cultures for 28 h. Cells were then fixed with 3.7% formaldehyde and 1% Triton X-100, and nuclei were stained with bisbenzimide (Hoechst dye no. 33258) for 30 min at 22 °C. Imaging acquisition was performed by using the Operetta CLS High-Content Analysis System (Perkin Elmer). Analysis was performed using Harmony software of Perkin Elmer and the Fundacin MEDINA in-house App NucleusFinder, based on an open source processing image program, ImageJ. NucleusFinder identified the regularly shaped mononuclear, binuclear, and multinuclear cells, excluded irregular, small, and isolated nuclei (odd nuclei), and detected valid micronuclei following very conservative conditions as cytoplasmic location without connection with the main nuclei and proper size. NucleusFinder chose the best analysis algorithm for each image capture from six implemented filters, using a smart fit choice calculation. It allowed us to perform classification and a counting of the different elements. The cytokinesis-block proliferation index (CBPI), which indicates the average number of cell cycles per cell during the period of exposure to cytoB, was used to estimate the cytostatic activity of a treatment by comparing values in the treated and control cultures. Cytostasis percentage did not exceed 60% because higher levels may induce micronuclei as a secondary effect of cytotoxicity and was calculated as follows
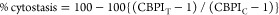
where T is the test compound treatment culture, C is the vehicle control culture, and CBPI = {(No. mononucleate cells) + (2 × No. binucleate cells) + (3 × No. multinucleate cells)}/(total number of cells).

#### 4.8.2 AMES Test

A commercial test kit, AMES MPFTM 98/100 Microplate Format Mutagenicity Assay, from Xenometrix AG (Allschwil, Switzerland), was used to evaluate the mutagenicity of compounds with and without metabolic activation S9 using amino acid requiring *S. typhimurium* strains TA100 and TA98.

### 4.9 Mitochondrial Toxicity

The protocol adopted by Swiss et al.^33^ and Marroquin et al.^55^ was applied for in vitro testing using HepG2 cells cultured in either glucose or galactose. HepG2 cells (50,000) were seeded in each well of two 96-well plates, and 100 μL of growth medium (glucose media) was added. Cells were allowed to adhere overnight in a cell incubator at 37 °C. The next day, the medium was removed, cells were washed with PBS, and 100 μL of medium (with glucose) containing positive control (Rotenone) was added, before the addition of the test compounds. The same procedure was repeated for plate 2 with medium replaced with galactose. Cells were again incubated at 37 °C for 24 h. CellTiter-Glo 2.0 Cell Viability (100 μL) (Promega) was then added to each well to measure the amount of ATP. The plate was placed in a shaker for 2 min and incubated for 15 min in the dark at 22 °C. Luminescence was read in a luminometer Tecan Infinite 200 pro. Luminescence from each well treated with the compound was compared to the negative control (medium only).

### 4.10 Ion Channel Screening with Manual Patch-Clamp Method

Stably transfected hERG, Nav1.5 cells (CHO), and inducible Cav1.2 (HEK-293) used in this study were obtained from Dr. Brian T. Donovan (hERG; GSK) and B’SYS GmbH (Nav1.5 and Cav1.2; Witterswil, Switzerland). The cells were maintained as previously described^56^ or according to the provided datasheets. All culture medium components and chemicals used for patch-clamp solutions were purchased from Thermo Fisher Scientific and Sigma-Aldrich (Germany), respectively. Tetracycline (2.5 μg/mL) was added to the culture media 48 h prior to recordings to induce the expression of Cav1.2.

The extracellular solution for hERG, Nav1.5, and Cav1.2 contained hERG/Nav1.5—137 mM NaCl, 4 mM KCl, 1.8 mM CaCl_2_, 1 mM MgCl_2_, 10 mM d-glucose, 10 mM HEPES, pH 7.4 adjusted with NaOH; Cav1.2—100 mM NaCl, 4 mM KCl, 40 mM NMDG, 5 mM CaCl_2_, 1 mM MgCl_2_, 5 mM d-glucose, 10 mM HEPES, and 5 mM sorbitol, pH 7.4 adjusted with HCl; osmolarity 290–300 mOsm. The intracellular solutions for hERG, Nav1.5, and Cav1.2 contained hERG—130 mM KCl, 1 mM MgCl_2_, 5 mM EGTA, 5 mM Mg-ATP, 10 mM HEPES; Nav1.5—120 mM KCl, 6 mM MgCl_2_, 5 mM EGTA, 10 mM NaCl, 10 mM HEPES, pH 7.2 adjusted with KOH; Cav1.2—132 mM CsCl, 1 mM KCl, 0.1 mM CaCl_2_, 4 mM Mg-ATP, 0.4 mM NaGTP, 10 mM EGTA, 10 mM HEPES, pH 7.2 adjusted with CsOH; osmolarity 280–290 mOsm. The test compounds were first dissolved in DMSO to make a stock solution (10 mM) and then diluted in the extracellular solution to 10 μM. The final DMSO concentration was 0.1%.

All recordings (five or six for each assay) were performed at 22 °C. Currents were measured using the whole-cell voltage-clamp method with an Axopatch 200B patch-clamp amplifier (sampling frequency, hERG—2.5 kHz, Nav1.5—20 kHz, Cav1.2—5 kHz; low-pass filter frequency, hERG/Cav1.2—1 kHz, Nav1.5—2 kHz), digitized with a Digidata 1440A/1550A interface under the control of the pCLAMP 10 software (Molecular Devices). Glass pipettes were pulled from borosilicate glass (Harvard Apparatus) by a horizontal puller (DMS universal puller, Germany) and had a resistance of 4–8 MΩ when filled with intracellular solutions. After the rupture of the cell membrane, the cell was allowed to stabilize for 3–5 min before recordings. Currents were induced using multiple sweeps of a voltage waveform at a holding potential of −80 mV for 200 ms, stepping to −50 mV for 200 ms, stepping to +20 mV for 5 s, stepping to −50 mV for 5 s, and returning to −80 mV (delivered once every 15 s for hERG); a holding potential of −120 mV for 20 ms, stepping to −30 mV for 50 ms, and returning to −120 mV (delivered once every 2 s for Nav1.5); a holding potential of −80 mV for 50 ms, stepping to −50 mV for 100 ms, stepping to 0 mV for 200 ms, and returning to −80 mV (delivered once every 10 s for Cav1.2). The access resistance was continuously monitored. A negative control, consisting of extracellular solutions with 0.1% DMSO, was applied until a stable current amplitude was achieved, followed by the application of test compounds. The compound was given 5 min to reach a steady-state block, followed by wash-out with the second negative control. A positive control consisting of quinidine (hERG—50 μM, Nav1.5—1 mM) or verapamil (Cav1.2—1 mM) was further applied after the wash-out. The peak hERG tail current amplitude was measured as the peak positive current at the second −50 mV step minus the initial −50 mV step and further normalized to the peak tail current amplitude in the first negative control to evaluate the level of hERG inhibition. The Nav1.5 current amplitude was measured as the difference between the maximum inward current during the first 10 ms of the −30 mV step minus the mean current during the last 5 ms of the same voltage step and further normalized to the Nav1.5 current amplitude in the negative control to determine the level of Nav1.5 inhibition. The Cav1.2 peak current amplitude was measured as the difference between the maximum inward current of the 0 mV depolarizing step and the current of the −50 mV step and further normalized to the Cav1.2 current amplitude in the negative control to evaluate the level of Cav1.2 inhibition The normalized hERG/Nav1.5/Cav1.2 currents were presented as mean ± standard error of the mean (SEM).

### 4.11 ADME Assays

#### 4.11.1 Kinetic Solubility

Kinetic solubility, utilizing a test compound from 10 mM DMSO stock solution, was measured at a final compound concentration of 100 μM and 1% DMSO. The test compound was added to 100 mM potassium phosphate buffer and incubated at 37 °C for at least 20 h in a heater-shaker. After incubation, the samples were centrifuged at 3000*g* at 37 °C for 30 min to pellet the insoluble material, and an aliquot of the supernatant was taken for analysis. After dilution of the sample, the concentration of the dissolved compound was quantified by liquid chromatography coupled to triple quadrupole mass spectrometry (LC-MS/MS).

#### 4.11.2 Thermodynamic Solubility

Thermodynamic solubility assay utilized the solid form of a test compound. The solid test compound (2–3 mg) was weighed in a glass HPLC vial, and 100 mM K_3_PO4 buffer, pH 7.4 was added to give a theoretical max. concentration of ∼5–6 mg/mL. The vial was incubated in a rotational shaker at 900 rpm, 37 °C for 24 h. After the incubation, an aliquot (200 μL) was transferred to a glass insert and centrifuged at 10,000*g*, 37 °C for 20 min to separate any solid material from the solution. The supernatant was transferred to a new HPLC vial and analyzed by liquid chromatography coupled to triple quadrupole mass spectrometry (LC-MS/MS).

#### 4.11.3 Chemical Stability

A test compound was pipetted (0.5 μL) into HPLC vials from 10 mM DMSO stocks to yield 5 μM final concentration (1000 μL inc. volume). To the reaction, start buffer/H_2_O (1:1) or buffer/isopropanol (1:1) was added. The following buffers were used: pH 2 (H_3_PO_4_/KH_2_PO_4_ 10 mM), pH 4 (ammonium formate 50 mM, isotonic), pH 7.4 (KH_2_PO_4_/K_2_HPO_4_ 10 mM), and pH 10 (glycine/NaOH 10 mM). Immediately (<1 min) after buffer or buffer/IPA addition, a 50 μL aliquot was added to a separate plate containing 150 μL of acetonitrile supplemented with Warfarin as the internal standard (IS), sealed, and stored at −80 °C. This was repeated at 15 and 45 min and after 1, 2, 4, and 24 h. The samples were analyzed by liquid chromatography coupled to triple quadrupole mass spectrometry (LC-MS/MS).

#### 4.11.4 p*K*_a_ Determination

The p*K*_a_ measurements were performed on a Sirius T3 automated instrument from Sirius Analytical Ltd. (East Sussex, U.K.) equipped with a D-PAS (dip probe absorption spectroscopy) lamp for spectrophotometric titrations and electrode for potentiometric titrations. The spectrophotometric titrations were performed using 2–5 μL of 10 mM DMSO compound stock. During the titration, the instrument added a predetermined volume of ionic-strength-adjusted (ISA) water or a combination of ISA and ISA containing 80% methanol in the potentiometric titrations. A titration from high-to-low or low-to-high was performed between pH 2 and 12. During the titration, the instrument collected a UV–vis spectrum by using the D-PAS technique to establish a titration curve. In the potentiometric method, the instrument instead bases the titration on the amount of acid (HCl) and base (KOH) that was added. The electrode was calibrated using a blank titration from pH 1.8 to pH 12.0 before every individual determination. The measurements were performed under argon to minimize the effect of dissolved CO_2_. Precipitation was continuously monitored at 500 nm. The temperature was controlled throughout the experiment at 25 ± 1 °C.

#### 4.11.5 log *D* Determination

A miniaturized shake-flask method in HPLC vials was applied. Solutions used were potassium phosphate 0.05 M pH 7.4 (KP) saturated with octanol and octanol saturated with KP. The phase ratios used were 1:1 and 1:3, i.e., 0.8/0.4 mL of octanol + 0.8/1.2 mL of KP. Compound (1.6 μL) from 10 mM DMSO stock was pipetted into a HPLC vial. Octanol was added followed by KP. The vial was sealed and vortexed, and phase separation was set for 48 h at an ambient temperature (ca 23 °C) in the dark. Next, the octanol phase was carefully separated with a pipette. Both phases were then analyzed against a separate standard curve by liquid chromatography coupled to triple quadrupole mass spectrometry (LC-MS/MS).

#### 4.11.6 Metabolic Stability in the Presence of Human and Animal Liver Microsomes

Metabolic stability was determined in 0.5 mg/mL of human or animal liver microsomes at a compound concentration of 1 μM in 100 mM of K_3_PO_4_ buffer pH 7.4 in a total incubation volume of 500 μL. The reaction was initiated by the addition of 1 mM NADPH. At various incubation times, i.e., at 0, 5, 10, 20, 40, and 60 min, a sample was withdrawn from the incubation and the reaction was terminated by the addition of cold acetonitrile with warfarin as an internal standard. The amount of parent compound remaining was analyzed by liquid chromatography coupled to triple quadrupole mass spectrometry (LC-MS/MS).

#### 4.11.7 Plasma Protein Binding and Stability in Human and Animal Plasma

Pooled human plasma was provided by Uppsala Academic Hospital and was collected from two donors (nonsmoking) (citric acid). In brief, 0.2 mL of the plasma (50% plasma, 50% isotonic buffer) test solution (typically 10 μM of the final compound concentration) was transferred to the membrane tube in the RED insert (Thermo Fisher Scientific). Isotonic phosphate buffer (0.35 mL, pH 7.4) was added to the other side of the membrane. The 96-well base plate was then sealed with an adhesive plastic film (Scotch Pad) to prevent evaporation. The sample was incubated with rapid rotation (>900 rpm) on a Kisker rotational incubator at 37 °C for 4 h to achieve equilibrium. A stability test of the test solution was prepared (to allow the detection of drug degradation), and >100 μL of the plasma test solution (in a plastic vial or on a sealed plate) was incubated at 37 °C for 4 h (or as long as the dialysis time). The plasma test solution was frozen directly after the administration to prevent any degradation. Prior to LC-MS/MS analysis, the plasma and buffer sample were treated with the addition of methanol (1:3) containing warfarin as the internal standard to precipitate proteins. The standard curve was created using the plasma standard. The plate was then sealed and centrifuged, and the supernatant was analyzed by liquid chromatography coupled to triple quadrupole mass spectrometry (LC-MS/MS).

#### 4.11.8 Caco-2 Cell Permeability Assay

Caco-2 cell monolayers (passages 94–105) were grown on a permeable filter support and used for transport study on day 21 after seeding. Prior to the experiment, a 10 μM drug solution was prepared and warmed to 37 °C. The Caco-2 filters were washed with prewarmed HBSS prior to the experiment, and thereafter the experiment was started by applying the donor solution on the apical or basolateral side. The transport experiments were carried out at pH 7.4 in both the apical and basolateral chambers. The experiments were performed at 37 °C and with a stirring rate of 500 rpm. The receiver compartment was sampled at 15, 30, and 60 min, and at 60 min also a final sample from the donor chamber was taken to calculate the mass balance of the compound. The samples (100 μL) were transferred to a 96-well plate containing 100 μL of methanol and warfarin as IS and was sealed until liquid chromatography coupled to triple quadrupole mass spectrometry (LC-MS/MS). The borders for low-moderate-high permeability (*P*_app_) in the assay setup is
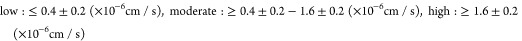


#### 4.11.9 Liquid Chromatography Coupled to Triple Quadrupole Mass Spectrometry (LC-MS/MS)

The test compounds were optimized on a Waters Acquity UPLC XEVO TQ-S micro system (Waters Corp.) operating in multiple reaction monitoring (MRM) mode with positive or negative electrospray ionization using the QuanOptimize software (Waters Corp.).

For chromatographic separation, a C18 BEH 1.7 μm column was used, with a general gradient of 1–90% of mobile phase B over a total running time of 2 min. Mobile phase A consisted of 5% acetonitrile and 0.1% formic acid in purified water, and mobile phase B consisted of 0.1% formic acid in 100% acetonitrile. The flow rate was set to 0.5 mL/min, and 5 μL of the sample was injected.

## Supporting Information Available

IC_50_ and MIC values, solubility and free fraction data, X-ray crystallography details, ADMET data, NMR spectra, and HPLC traces (PDF)
Molecular formula strings (CSV)

## Accession Codes

PDB ID Codes complex of *E. coli* GyrB24 with **1**: 7P2M; complex of *E. coli* GyrB24 with **7**: 7P2W; complex of *A. baumannii* GyrB23 with **27**: 7PQL; complex of *A. baumannii* GyrB23 with (*S*)-**27**: 7PQM; complex of *A. baumannii* GyrB23 with novobiocin: 7PQI; complex of *P. aeruginosa* GyrB24 with (*S*)-**27**: 7PTG; complex of *P. aeruginosa* GyrB24 with novobiocin: 7PTF.

## Notes

The authors will release the atomic coordinates and experimental data upon article publication.

## Abbreviations Used

CDI: 1,1′-carbonyldiimidazole
CFU: colony-forming unit
DCM: dichloromethane
DIAD: diisopropyl azodicarboxylate
DIPEA: *N*,*N*-diisopropylethylamine
DMF: *N*,*N*-dimethylformamide
FMCA: fluorometric microculture cytotoxicity assay
hERG: human Ether-à-go-go-Related Gene
HPLC: high-performance liquid chromatography
LDH: lactate dehydrogenase
MBC: minimal bactericidal concentration
MDR: multidrug-resistant
MIC: minimum inhibitory concentration
MNT: in vitro cell micronucleus test
MTS: (3-[4,5,dimethylthiazol-2-yl]-5-[3-carboxymethoxy phenyl]-2-[4-sulfophenyl]-2*H*-tetrazolium)
PMB: 4-methoxybenzyl
THF: tetrahydrofurane
TLC: thin-layer chromatography
TBTU: 2-(1*H*-benzotriazole-1-yl)-1,1,3,3-tetramethylaminium tetrafluoroborate
TPSA: total polar surface area
