# Supplementary material for: Discovery and Hit-to-Lead Optimization of Benzothiazole Scaffold-Based DNA Gyrase Inhibitors with Potent Activity against Acinetobacter baumannii and Pseudomonas aeruginosa
Source: J Med Chem. 2023 Jan 12;66(2):1380–425. doi: 10.1021/acs.jmedchem.2c01597 (PMC9884090; doi:10.1021/acs.jmedchem.2c01597)
Supplement: Supplementary file 1 — jm2c01597_si_001.pdf [file jm2c01597_si_001.pdf]

Discovery and Hit-to-Lead Optimization of Benzothiazole Scaffold-Based DNA Gyrase Inhibitors with Potent Activity Against *Acinetobacter baumannii* and *Pseudomonas aeruginosa*

Andrej Emanuel Cotman,[1] Martina Durcik,[1] Davide Benedetto Tiz,[1,#] Federica Fulgheri,[1,⊥] Daniela Secci,[1,⊥] Maša Sterle,[1] Štefan Možina,[1,◊] Žiga Skok,[1,◊] Nace Zidar,[1] Anamarija Zega,[1] Janez Ilaš,[1] Lucija Peterlin Mašič,[1] Tihomir Tomašič,[1] Diarmaid Hughes,[2] Douglas L. Huseby,[2] Sha Cao,[2] Linnéa Garoff,[2] Talía Berruga Fernández,[2] Paraskevi Giachou,[2] Lisa Crone,[2] Ivailo Simoff,[3] Richard Svensson,[3] Bryndis Birnir,[4] Sergiy V. Korol,[4] Zhe Jin,[4] Francisca Vicente,[5] Maria C. Ramos,[5] Mercedes de la Cruz,[5] Björn Glinghammar,[6] Lena Lenhammar,[7] Sara R. Henderson,[8,†] Julia E. A. Mundy,[8] Anthony Maxwell,[8] Clare E. M. Stevenson,[8] David M. Lawson,[8] Guido V. Janssen,[9] Geert Jan Sterk,[9] Danijel Kikelj*,[1]

[1] University of Ljubljana, Faculty of Pharmacy, Aškerčeva cesta 7, 1000 Ljubljana, Slovenia

[2] Uppsala University, Department of Medical Biochemistry and Microbiology, Husargatan 3, 75123 Uppsala, Sweden

[3] Uppsala University, Drug Optimization and Pharmaceutical Profiling Platform (UDOPP), Department of Pharmacy, Husargatan 3, 75123 Uppsala, Sweden

[4] Uppsala University, Department of Medical Cell Biology, Husargatan 3, 75123 Uppsala, Sweden

[5] Fundación MEDINA, Avenida del Conocimiento 34, Parque Tecnológico Ciencias de la Salud, 18016 Granada, Spain

[6] RISE Research Institutes of Sweden, Department Chemical Process and Pharmaceutical Development, Unit Chemical and Pharmaceutical Safety, 15136 Södertälje, Sweden

[7] Department of Medical Sciences, Uppsala University Hospital, 75185 Uppsala, Sweden

[8] Department of Biochemistry and Metabolism, John Innes Centre, Norwich Research Park, Norwich NR4 7UH, U.K.

[9] Vrije Universiteit Amsterdam, Medicinal Chemistry Division, De Boelelaan 1108, 1081 HZ Amsterdam, The Netherlands

* *danijel.kikelj@ffa.uni-lj.si*

Supporting Information

Contents

Table S1. MIC_{90} Values of Compound **1** Against MDR Clinical Isolates of *Acinetobacter spp.* and *P. aeruginosa*. 3

Table S2. MIC_{90} Values of Compound **27** Against MDR Clinical Isolates of *Acinetobacter spp.* 4

Table S3. IC_{50} Values Against *E. coli*, *A. baumannii* and *P. aeruginosa* DNA Gyrase and Topoisomerase IV, MICs, Solubility and Free Fraction Data of Analogs of Compound **1** Containing a Carboxylic Group Surrogate. 5

Table S4. IC_{50} Values Against *E. coli*, *A. baumannii* and *P. aeruginosa* DNA Gyrase and Topoisomerase IV, MICs, Solubility and Free Fraction Data of 5-Aminomethylpyrrole Derivatives of Compound **1**. 5

Table S5: IC_{50} Values Against *E. coli*, *A. baumannii* and *P. aeruginosa* DNA Gyrase and Topoisomerase IV, MICs, Solubility and Free Fraction Data of Derivatives of Compound **27** with Polar Substituents at the Stereogenic Carbon Atom. 6

Table S6. IC_{50} Values Against *E. coli*, *A. baumannii* and *P. aeruginosa* DNA Gyrase and Topoisomerase IV, MICs, Solubility and Free Fraction Data of Analogs of Compound **27** with Phenyl Group Replacements..........................7

Table S7. IC_{50} Values Against *E. coli*, *A. baumannii* and *P. aeruginosa* DNA Gyrase and Topoisomerase IV, MICs, Solubility and Free Fraction Data of Analogs of Compound **27** with Differently Substituted Pyrrole Ring. 8

Table S8. IC_{50} Values Against *E. coli*, *A. baumannii* and *P. aeruginosa* DNA Gyrase and Topoisomerase IV, MICs, Solubility and Free Fraction Data of Compounds Resulting from Combined Approaches to Modification of Compound **27**. 9

Table S9: IC_{50} Values Against *E. coli*, *A. baumannii* and *P. aeruginosa* DNA Gyrase and Topoisomerase IV, MICs, Solubility and Free Fraction Data of Derivatives of Compound **27** With *para* Substituted Phenyl Ring..................10

Table S10. Antibacterial Activity of Compounds **1**–**73** Against *S. aureus* and *K. pneumoniae*................................11

Table S11. Plasma Protein Binding of Selected Optimized Derivatives of Compounds **1** and **27**. 14

Table S12. X-ray Data Collection and Refinement Statistics for Inhibitor Complexes of *E. coli* GyrB24 15

Table S13. X-ray Data Collection and Refinement Statistics for Inhibitor Complexes of *Acinetobacter baumannii* GyrB23 16

Table S14. X-ray Data Collection and Refinement Statistics for Inhibitor Complexes of *Pseudomonas aeruginosa* GyrB24 17

Figure S1. Co-crystal structures of novobiocin in complex with A. baumannii GyrB23 and P. aeruginosa GyrB24.18

Figure S2. hERG, Cav1.2 and Nav1.5 ion channel inhibition assay for Compound **1** (EBL-2560). 18

Figure S3. hERG (A), Nav1.5 (B) and Cav1.2 (C) ion channel inhibition assay for Compound **27**. 19

Figure S4. Mitochondrial toxicity assay of Compound **1** in HepG2 cells. 19

Figure S5. Mitochondrial toxicity assay of Compound **27** in HepG2 cells. 20

Figure S6. Micronucleus test (MNT) results for Compound **1** (EBL2560). 20

Figure S7. Micronucleus test (MNT) results for Compound **18**. 20

Figure S8. Micronucleus test (MNT) results for Compound **27** (EBL-2704)...20

Figure S9. Mutagenic activity of Compound **1** (EBL-2560) using *S. typhimurium* TA100 with and without S9 activation scored on days 3 to 7 of growth (AMES test). 21

Figure S10. Mutagenic activity of Compound **27** (EBL-2704) using *S. typhimurium* TA100 with (top) and without (bottom) S9 activation (AMES test). Positive controls: 4-NQQ = 4-nitroquinoline-*N*-oxide; 2-AA = 2-aminoanthracene. 21

Figure S11. Mutagenic activity of Compound **31** (EBL-2837) using *S. typhimurium* TA98 with (top) and without (bottom) S9 activation (AMES test). Positive controls: 2-NF = 2-nitrofluorene; 2-AA = 2-aminoanthracene. 21

Figure S12. CYP3A4 inhibition assay of Compound 1. 22

Figure S13. Metabolism of Compound **1** (EBL-2560) in human and mouse hepatocytes, and human (HLM) and mouse (MLM) liver microsomes. 22

Figure S14. Metabolism of Compound **27** (EBL-2704) in human (left) and mouse (right) hepatocytes. 22

Figure S15. Relationship between cLogP, TPSA, kinetic solubility, and MIC against *P. aeruginosa*........................23

Figure S16. Relationship between cLogP, TPSA, kinetic solubility, and MIC against *S. aureus*. 23

NMR Spectra 24

HPLC traces 69

Table S1. MIC_{90} Values of Compound 1 Against MDR Clinical Isolates of *Acinetobacter spp.* and *P. aeruginosa*.

	Strain ID	species	MIC_{90} [µg/mL]	Strain ID	species	MIC_{90} [µg/mL]
1	EN0117	*A. baumannii*	2	EN0064	*P. aeruginosa*	0,5
2	EN0118	*A. baumannii*	1	EN0065	*P. aeruginosa*	1
3	EN0119	*A. baumannii*	1	EN0066	*P. aeruginosa*	1
4	EN0174	*A. pittii or nosocomialis*	0,5	EN0067	*P. aeruginosa*	2
5	EN0175	*A. baumannii*	1	EN0068	*P. aeruginosa*	1
6	EN0176	*A. baumannii*	1	EN0069	*P. aeruginosa*	0,5
7	EN0177	*A. baumannii*	1	EN0070	*P. aeruginosa*	2
8	EN0178	*A. baumannii*	0,5	EN0071	*P. aeruginosa*	2
9	EN0179	*A. baumannii*	<0,125	EN0072	*P. aeruginosa*	8
10	EN0180	*A. pittii or nosocomialis*	0,25	EN0073	*P. aeruginosa*	>64
11	EN0181	*A. pittii or nosocomialis*	0,5	EN0074	*P. aeruginosa*	8
12	EN0182	*A. pittii or nosocomialis*	0,5	EN0075	*P. aeruginosa*	4
13	EN0183	*A. pittii or nosocomialis*	1	EN0076	*P. aeruginosa*	2
14	EN0184	*A. baumannii*	2	EN0077	*P. aeruginosa*	1
15	EN0185	*A. baumannii*	2	EN0078	*P. aeruginosa*	4
16	EN0186	*A. baumannii*	2	EN0079	*P. aeruginosa*	8
17	EN0187	*A. baumannii*	2	EN0129	*P. aeruginosa*	0,5
18	EN0188	*A. baumannii*	0,5	EN0215	*P. aeruginosa*	4
19	EN0189	*A. pittii or nosocomialis*	2	EN0216	*P. aeruginosa*	16
20	EN0190	*A. baumannii*	2	EN0217	*P. aeruginosa*	4
21	EN0191	*A. baumannii*	1	EN0218	*P. aeruginosa*	2
22	EN0192	*A. baumannii*	1	EN0220	*P. aeruginosa*	2
23	EN0193	*A. baumannii*	1	EN0221	*P. aeruginosa*	4
24	EN0194	*A. baumannii*	2	EN0222	*P. aeruginosa*	2
25	EN0273	*A. baumannii*	8	EN0223	*P. aeruginosa*	0,5
26	EN0274	*A. baumannii*	16	EN0224	*P. aeruginosa*	2
27	EN0276	*A. baumannii*	16	EN0226	*P. aeruginosa*	2
28	EN0277	*A. baumannii*	2	EN0227	*P. aeruginosa*	4
29	EN0278	*A. baumannii*	8	EN0228	*P. aeruginosa*	4
30	EN0279	*A. baumannii*	2	EN0230	*P. aeruginosa*	2
31	EN0280	*A. baumannii*	1	EN0231	*P. aeruginosa*	8
32	EN0281	*A. baumannii*	1	EN0232	*P. aeruginosa*	4
33	EN0282	*A. baumannii*	2	EN0337	*P. aeruginosa*	4
34	EN0283	*A. baumannii*	1	EN0338	*P. aeruginosa*	2
35	EN0284	*A. baumannii*	1	EN0339	*P. aeruginosa*	4
36	EN0285	*A. baumannii*	2	EN0340	*P. aeruginosa*	2
37	EN0286	*A. baumannii*	1	EN0341	*P. aeruginosa*	4
38	EN0287	*A. baumannii*	1	EN0342	*P. aeruginosa*	8
39	EN0288	*A. baumannii*	1	EN0343	*P. aeruginosa*	4
40	EN0289	*A. baumannii*	1	EN0344	*P. aeruginosa*	8
41	EN0290	*A. baumannii*	1	EN0345	*P. aeruginosa*	2
42	EN0291	*A. baumannii*	1	EN0346	*P. aeruginosa*	4
43	EN0292	*A. baumannii*	1	EN0347	*P. aeruginosa*	8
44	EN0293	*A. baumannii*	2	EN0348	*P. aeruginosa*	4
45	EN0294	*A. baumannii*	2	EN0349	*P. aeruginosa*	2
46	EN0295	*A. baumannii*	4	EN0350	*P. aeruginosa*	2
47	EN0296	*A. baumannii*	2	EN0351	*P. aeruginosa*	4
48	EN0297	*A. baumannii*	2	EN0352	*P. aeruginosa*	2
49	EN0298	*A. baumannii*	2	EN0407	*P. aeruginosa*	4
50	EN0299	*A. baumannii*	1	EN0408	*P. aeruginosa*	4
51	EN0300	*A. baumannii*	2	EN0409	*P. aeruginosa*	1
52	EN0301	*A. baumannii*	1	EN0410	*P. aeruginosa*	0,5
53	EN0427	*A. baumannii*	2	EN0411	*P. aeruginosa*	2
54	EN0428	*A. baumannii*	0,5	EN0412	*P. aeruginosa*	4
55	EN0429	*A. baumannii*	2	EN0413	*P. aeruginosa*	4
56	EN0430	*A. baumannii*	2	EN0414	*P. aeruginosa*	0,5
57	EN0431	*A. baumannii*	0,25	EN0415	*P. aeruginosa*	4
58	EN0432	*A. baumannii*	8	EN0416	*P. aeruginosa*	4
59	EN0433	*A. baumannii*	1	EN0418	*P. aeruginosa*	4
60	EN0434	*A. baumannii*	1	EN0419	*P. aeruginosa*	2
61	EN0435	*A. baumannii*	2	EN0420	*P. aeruginosa*	16
62	EN0795	*A. baumannii*	2	EN0422	*P. aeruginosa*	4
63	EN0800	*A. baumannii*	1	EN0423	*P. aeruginosa*	4
64	EN0801	*A. baumannii*	2	EN0425	*P. aeruginosa*	4
		**MIC90**	**8**		**MIC90**	**2**

Table S2. MIC_{90} Values of Compound 27 Against MDR Clinical Isolates of *Acinetobacter spp.*

	Strain ID	species	MIC_{90} [µg/mL]
1	EN0117	*A. baumannii*	1
2	EN0118	*A. baumannii*	1
3	EN0119	*A. baumannii*	2
4	EN0174	*A. pittii or nosocomialis*	0,5
5	EN0175	*A. baumannii*	2
6	EN0176	*A. baumannii*	1
7	EN0177	*A. baumannii*	1
8	EN0178	*A. baumannii*	0,5
9	EN0179	*A. baumannii*	0,125
10	EN0180	*A. pittii or nosocomialis*	0,5
11	EN0181	*A. pittii or nosocomialis*	2
12	EN0182	*A. pittii or nosocomialis*	0,25
13	EN0183	*A. pittii or nosocomialis*	1
14	EN0184	*A. baumannii*	1
15	EN0185	*A. baumannii*	1
16	EN0186	*A. baumannii*	0,5
17	EN0187	*A. baumannii*	2
18	EN0188	*A. baumannii*	0,5
19	EN0189	*A. pittii or nosocomialis*	2
20	EN0190	*A. baumannii*	2
21	EN0191	*A. baumannii*	2
22	EN0192	*A. baumannii*	1
23	EN0193	*A. baumannii*	0,5
24	EN0194	*A. baumannii*	1
25	EN0273	*A. baumannii*	0,5
26	EN0274	*A. baumannii*	0,5
27	EN0276	*A. baumannii*	0,5
28	EN0277	*A. baumannii*	0,5
29	EN0278	*A. baumannii*	0,5
30	EN0279	*A. baumannii*	1
31	EN0280	*A. baumannii*	0,5
32	EN0281	*A. baumannii*	0,5
33	EN0282	*A. baumannii*	0,5
34	EN0283	*A. baumannii*	0,5
35	EN0284	*A. baumannii*	0,5
36	EN0285	*A. baumannii*	1
37	EN0286	*A. baumannii*	1
38	EN0287	*A. baumannii*	0,5
39	EN0288	*A. baumannii*	0,5
40	EN0289	*A. baumannii*	0,5
41	EN0290	*A. baumannii*	1
42	EN0291	*A. baumannii*	0,5
43	EN0292	*A. baumannii*	1
44	EN0293	*A. baumannii*	2
45	EN0294	*A. baumannii*	0,5
46	EN0295	*A. baumannii*	1
47	EN0296	*A. baumannii*	1
48	EN0297	*A. baumannii*	2
49	EN0298	*A. baumannii*	1
50	EN0299	*A. baumannii*	1
51	EN0300	*A. baumannii*	2
52	EN0301	*A. baumannii*	1
53	EN0427	*A. baumannii*	2
54	EN0428	*A. baumannii*	1
55	EN0429	*A. baumannii*	2
56	EN0430	*A. baumannii*	2
57	EN0431	*A. baumannii*	0,5
58	EN0432	*A. baumannii*	2
59	EN0433	*A. baumannii*	2
60	EN0434	*A. baumannii*	1
61	EN0435	*A. baumannii*	2
		**MIC90**	**2**

Table S3. IC_{50} Values Against *E. coli*, *A. baumannii* and *P. aeruginosa* DNA Gyrase and Topoisomerase IV, MICs, Solubility and Free Fraction Data of Analogs of Compound 1 Containing a Carboxylic Group Surrogate.

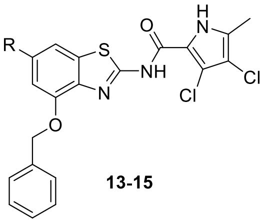

13-15

Cpd.	R	LogD/ LogP Calc.	TPSA [Å²] Calc.	*E. coli* [a]			*A. baumannii* [b]			*P. aeruginosa* [c]			Solu-bility [μM]	Free fraction [%] human/ mouse
				Gyrase IC_{50} [nM]	*Topo IV* IC_{50} [nM]	MIC [μg/mL] WT (Δeff.)	*Gyrase* IC_{50} [nM]	*Topo IV* IC_{50} [nM][e]	MIC [μg/mL] WT (Δeff.)	*Gyrase* IC_{50} [nM]	*Topo IV* IC_{50} [nM][e]	MIC [μg/mL] WT (Δeff.)		
**13**	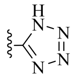	-/ 4.30	117.8	<10	680	>64 (16)	n.d.	n.d.	64 (n.d.)	n.d.	n.d.	>64 (1)	n.d.	n.d./ n.d.
**14**	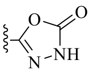	5.24/ 5.55	117.7	<10	n.d.	>64 (>64)	31±12	n.d.	>64 (n.d.)	14±4	n.d.	>64 (>64)	<1[d]	n.d. n.d.
**15**	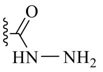	4.42/ 4.42	122.1	16±11	n.d.	>64 (64)	351±40	n.d.	32 (n.d.)	226±86	n.d.	64 (>64)	n.d.	<0.1/ <0.1

[a]*E. coli* ATCC5922 (wild type), CH3130 (efflux-defective; ΔtolC mutant isogenic to ATCC25922); [b]*A. baumannii* ATCC19606 (wild type), BM4652 (efflux-defective derivative of BM4454); [c]*P. aeruginosa* PAO1 (wild type), PAO750 (efflux-defective isogenic to PAO1); [d] kinetic solubility; [e]gel-based assay; TPSA = total polar surface area; Calc. = calculated; n.d. = not determined; WT = wild type; Δeff. = efflux-defective strain.

Table S4. IC_{50} Values Against *E. coli*, *A. baumannii* and *P. aeruginosa* DNA Gyrase and Topoisomerase IV, MICs, Solubility and Free Fraction Data of 5-Aminomethylpyrrole Derivatives of Compound 1.

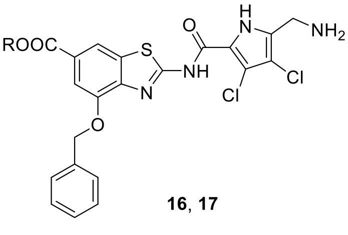

16, **17**

Cpd.	R	LogD/ LogP Calc.	TPSA [Å²] Calc.	*E. coli* [a]			*A. baumannii* [b]			*P. aeruginosa* [c]			Solu-bility [μM]	Free fraction [%] human/ mouse
				Gyrase IC_{50} [nM]	*Topo IV* IC_{50} [nM]	MIC [μg/mL] WT (Δeff.)	*Gyrase*IC_{50} [nM]	*Topo IV* IC_{50} [nM][e]	MIC [μg/mL] WT (Δeff.)	*Gyrase* IC_{50} [nM]	*Topo IV* IC_{50} [nM][e]	MIC [μg/mL] WT (Δeff.)		
**16**	H	2.03/ 2.10	130.3	<10	not active	>64 (>64)	487±141	3000±640	>64 (n.d.)	1140±398	5165±585	64 (n.d.)	<1[d]	0.59 <0.1[f]
**17**	CH_3	3.96/ 4.71	119.3	22200±4800	n.d.	>64 (>64)	n.d.	n.d.	>64 (n.d.)	n.d.	n.d.	>64 (>64)	<1[d]	<0.1 0[f]

[a]*E. coli* ATCC5922 (wild type), CH3130 (efflux-defective; ΔtolC mutant isogenic to ATCC25922); [b]*A. baumannii* ATCC19606 (wild type), BM4652 (efflux-defective derivative of BM4454); [c]*P. aeruginosa* PAO1 (wild type), PAO750 (efflux-defective isogenic to PAO1); [d] kinetic solubility; [e]gel-based assay; [f]The results should be interpreted carefully due to low recovery; TPSA = total polar surface area; Calc. = calculated; n.d. = not determined; WT = wild type; Δeff. = efflux-defective strain.

Table S5: IC_{50} Values Against *E. coli*, *A. baumannii* and *P. aeruginosa* DNA Gyrase and Topoisomerase IV, MICs, Solubility and Free Fraction Data of Derivatives of Compound 27 with Polar Substituents at the Stereogenic Carbon Atom.

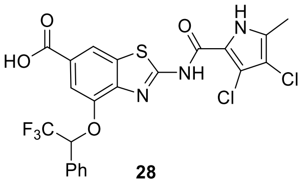

28

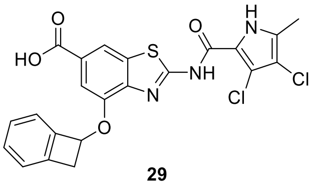

29

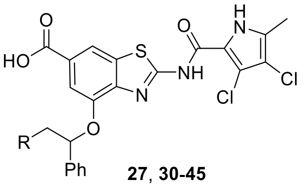

27, **30-45**

Cpd.	R	LogD/ LogP Calc.	TPSA [$Å^2$] Calc.	*E. coli* [a]			*A. baumannii* [b]			*P. aeruginosa* [c]			Solu-bility [µM]	Free fraction [%] human/ mouse
				Gyrase IC_{50} [nM]	*Topo IV* IC_{50} [nM]	MIC [µg/mL] WT (Δeff.)	*Gyrase* IC_{50} [nM]	Topo IV IC_{50} [nM][f]	MIC [µg/mL] WT (Δeff.)	*Gyrase* IC_{50} [nM]	*Topo IV* IC_{50} [nM][f]	MIC [µg/mL] WT (Δeff.)		
**27**	H	2.59/ 5.94	104.3	<10	318±94	1/ (0.125)	16±2	6.7±0.5	0.5/ (0.5)	<10	29±1	1/ (0.125)	>80 [d] 92 [e]	0.016/ 0.01
**28**	-	3.19/ 6.54	104.3	<10	54±8	16/ (<0.125)	<10	16±1	4/ (0.5)	<10	24±1	>64/ (0.25)	n.d.	<0.1[g]/ <0.1[g]
**29**	-	2.27/ 5.62	104.3	<10	n.d.	>64/ (<0.125)	<10	22±1	1/ (n.d.)	<10	196±3	>64/ (0.5)	n.d.	n.d./ n.d.
**30**	OH	1.54/ 4.89	124.5	<10	n.d.	8/ (0.125)	<10	19±3	4/ (0.125)	<10	43±8	32/ (0.5)	22 [d] 14 [e]	<0.1/ 0.1
**31**	OCH_3	2.19/ 5.53	113.5	<10	n.d.	1/ (<0.125)	<10	4.5±0.9	0.5/ (n.d.)	<10	19±1	2/ (0.25)	<1 [d] 12 [e]	<0.1/ 0.1
**32**	SO_2CH_3	0.91/ 4.26	138.5	<10	n.d.	>64/ (<0.125)	<10	n.d.	>64/ (n.d.)	<10	n.d.	>64/ (2)	16 [d]	0.05[g]/ 4.32[g]
**33**	NH_2	2.51/ 2.52	130.3	<10	n.d.	>64/ (0.5)	<10	n.d.	>64/ (n.d.)	<10	n.d.	>64/ (1)	90 [d]	0.28/ 0.53[g]
**34**	$N(CH_3)_2$	2.84/ 2.87	107.5	<10	77±42	4/ (<0.125)	<10	18±4	8/ (<0.125)	<10	27±2	16/ (<0.125)	16 [d]	n.d./ n.d.
**35**	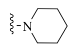	3.71/ 3.72	107.6	<10	n.d.	2/ (<0.125)	<10	25±1	4/ (0.25)	<10	26±5	8/ (0.25)	14 [d]	n.d./ n.d.
**36**	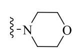	2.26/ 2.74	116.8	<10	n.d.	2/ (0.125)	<10	11±2	2/ (n.d.)	<10	28±1	8/ (2)	35 [d] 3[e]	<0.1/ 0.3[g]
**37**	$N^+(CH_3)_3$ I^-	2.84/ 2.87	107.5	<10	n.d.	32/ (2)	<10	n.d.	>64/ (n.d.)	<10	n.d.	>64/ (2)	10 [d]	n.d./ n.d.
**38**	NHAc	1.25/ 4.60	133.4	<10	n.d.	>64/ (<0.125)	<10	n.d.	>64/ (n.d.)	<10	n.d.	>64/ (16)	<1[d]	0.06[g]/ 0.23[g]
**39**	NHBoc	2.93/ 6.28	142.6	<10	n.d.	2/ (0.25)	16±5	1.7±0.2	2/ (n.d.)	<10	16±1	4/ (2)	14 [d]	1.61[g]/ <0.01 [g]
**40**	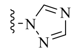	1.78/ 4.99	135.0	<10	n.d.	64/ (0.25)	25±4	n.d.	>64/ (n.d.)	17±6	n.d.	>64/ (16)	19[d]	n.d./ n.d.
**41**	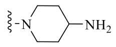	2.21/ 2.49	133.6	<10	n.d.	16/ (0.5)	<10	n.d.	>64/ (n.d.)	<10	n.d.	>64/ (2)	4[d]	n.d./ n.d.
**42**	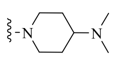	2.80/ 2.86	110.8	n.d.	n.d.	>64/ (1)	31±8	n.d.	>64/ (n.d.)	32±15	n.d.	>64/ (16)	4 [d]	<0.1/ 0.3
**43**	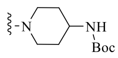	3.55/ 3.57	145.9	<10	n.d.	8/ (0.25)	<10	n.d.	>64/ (n.d.)	<10	n.d.	>64/ (4)	4 [d]	n.d./ n.d.
**44**	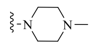	2.63/ 2.72	110.8	<10	n.d.	>64/ (2)	14±3	n.d.	>64/ (n.d.)	17±6	n.d.	>64/ (8)	12[d]	n.d./ n.d.
**45**	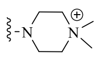	2.05/ 1.44	107.6	<10	-	>64/ (4)	<10	n.d.	>64/ (n.d.)	<10	n.d.	>64/ (8)	23[d]	0.3/ 1.9

[a]*E. coli* ATCC5922 (wild type), CH3130 (efflux-defective; ΔtolC mutant isogenic to ATCC25922); [b]*A. baumannii* ATCC19606 (wild type), BM4652 (efflux-defective derivative of BM4454); [c]*P. aeruginosa* PAO1 (wild type), PAO750 (efflux-defective isogenic to PAO1); [d]kinetic solubility; [e]thermodynamic solubility; [f]gel-based assay; [g]The results should be interpreted carefully due to low recovery; TPSA = total polar surface area; Calc. = calculated; n.d. = not determined; WT = wild type; Δeff. = efflux-defective strain.

Table S6. IC_{50} Values Against *E. coli*, *A. baumannii* and *P. aeruginosa* DNA Gyrase and Topoisomerase IV, MICs, Solubility and Free Fraction Data of Analogs of Compound 27 with Phenyl Group Replacements.

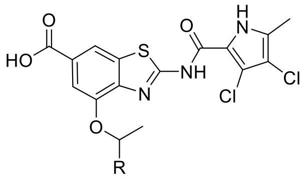

46-48

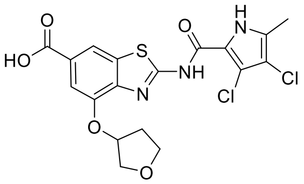

49

Cpd.	R	LogD/ LogP Calc.	TPSA [Å^2] Calc.	*E. coli* [a]			*A. baumannii* [b]			*P. aeruginosa* [c]			Solu-bility [µM]	Free fraction [%] human/ mouse
				Gyrase IC_{50} [nM]	*Topo IV* IC_{50} [nM]	MIC [µg/mL] WT (Δeff.)	*Gyrase* IC_{50} [nM]	*Topo IV* IC_{50} [nM][f]	MIC [µg/mL] WT (Δeff.)	*Gyrase* IC_{50} [nM]	*Topo IV* IC_{50} [nM][f]	MIC [µg/mL] WT (Δeff.)		
**27**	Ph	2.59/ 5.94	104	<10	318±94	1 (0.125)	16±2	6.7±0.5	0.5 (0.5)	<10	29±1	1 (0.125)	>80 [d] 92 [e]	0.016 0.01
**46**	Me	1.22/ 4.57	104	<10	84±12	4 (<0.125)	<10	67±11	1 (0.5)	<10	69±3	8 (0.125)	35 [d]	<0.1 <0.1
**47**	*i*Pr	2.11/ 5.46	104	<10	n.d.	1 (<0.125)	<10	60±1	1 (1)	<10	200±40	2 (<0.125)	29 [d]	<0.1 <0.1
**48**	cyclopropyl	1.65/ 4.99	104	<10	n.d.	4 (<0.125)	<10	50±5	1 (1)	<10	58±1	4 (<0.125)	35 [d]	<0.1 <0.1
**49**	-	0.35/ 3.70	114	<10	58±15	>64 (<0.125)	<10	n.d.	64 (n.d.)	<10	n.d.	>64 (8)	35[d]	0.03[g] 0.12[g]

[a]*E. coli* ATCC5922 (wild type), CH3130 (efflux-defective; ΔtolC mutant isogenic to ATCC25922); [b]*A. baumannii* ATCC19606 (wild type), BM4652 (efflux-defective derivative of BM4454); [c]*P. aeruginosa* PAO1 (wild type), PAO750 (efflux-defective isogenic to PAO1); [d] kinetic solubility; ethermodynamic solubility; [f]gel-based assay; [g] The results should be interpreted carefully due to low recovery; TPSA = total polar surface area; Calc. = calculated; n.d. = not determined; WT = wild type; Δeff. = efflux-defective strain.

Table S7. IC_{50} Values Against *E. coli*, *A. baumannii* and *P. aeruginosa* DNA Gyrase and Topoisomerase IV, MICs, Solubility and Free Fraction Data of Analogs of Compound 27 with Differently Substituted Pyrrole Ring.

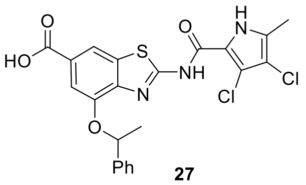

27

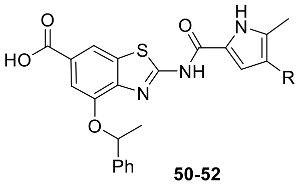

50-52

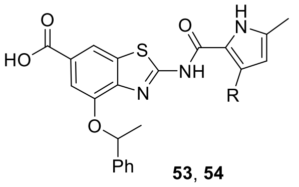

53, **54**

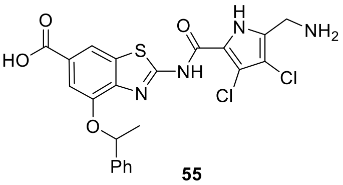

55

Cpd.	R	LogD/ LogP Calc.	TPSA [Å^2] Calc.	*E. coli* [a]			*A. baumannii* [b]			*P. aeruginosa* [c]			Solu-bility [µM]	Free fraction [%] human/ mouse
				Gyrase IC_{50} [nM]	*Topo IV* IC_{50} [nM]	MIC [µg/mL] WT (Δeff.)	*Gyrase* IC_{50} [nM]	*Topo IV* IC_{50} [nM][f]	MIC [µg/mL] WT (Δeff.)	*Gyrase* IC_{50} [nM]	*Topo IV* IC_{50} [nM][f]	MIC [µg/mL] WT (Δeff.)		
**27**	-	2.59/ 5.94	104	<10	318±94	1 (0.125)	16±2	6.7±0.5	0.5 (0.5)	<10	29±1	1 (0.125)	>80 [d] 92 [e]	0.016/ 0.01
**50**	Cl	1.99/ 5.33	104	<10	n.d.	16 (<0.125)	<10	12±1	64 (n.d.)	<10	7.3±1.6	32 (1)	23 [d] 19 [e]	<0.1/ <0.1
**51**	F	1.52/ 4.87	104	<10	37±12	8 (0.5)	<10	7.8±1.1	4 (4)	<10	9.3±1.1	4 (1)	41 [d] 18 [e]	0.13/ 0.3
***R*-51**	F	1.52/ 4.87	104	<10	n.d.	>64/ (>64)	<10	98±11	8 (1)	<10	35±5	>64 (<0.125)	<1[d] 7[e]	0.17/ 0.7
***S*-51**	F	1.52/ 4.87	104	<10	n.d.	8 (0.125)	<10	5.8±0	2 (0.125)	<10	4.1±0.7	32 (0.5)	52 [d] 20 [e]	0.19/ 0.7
**52**	CN	1.24/ 4.59	128	<10	126±27	>64 (16)	<10	167±15	>64 (n.d.)	<10	29±2	>64 (>64)	57 [d]	0.4/ 1.2
**53**	H	1.38/ 4.73	104	17	51±18	>64 (0.25)	24±14	n.d.	>64 (n.d.)	<10	n.d.	>64 (0.25)	3[d]	n.d./ n.d.
**54**	F	1.52/ 4.87	104	32	229±31	>64 (0.125)	15±12	818±13	64 (n.d.)	<10	954±40	>64 (1)	30[d]	n.d./ n.d.
**55**	-	2.44/ 2.52	130	41	n.d.	>64 (1)	18±4	n.d.	>64 (n.d.)	770	n.d.	>64 (64)	2[d]	1.28/ 0.3

[a]*E. coli* ATCC5922 (wild type), CH3130 (efflux-defective; ΔtolC mutant isogenic to ATCC25922); [b]*A. baumannii* ATCC19606 (wild type), BM4652 (efflux-defective derivative of BM4454); [c]*P. aeruginosa* PAO1 (wild type), PAO750 (efflux-defective isogenic to PAO1); [d]kinetic solubility; [e]thermodynamic solubility; [f]gel-based assay; TPSA = total polar surface area; Calc. = calculated; n.d. = not determined; WT = wild type; Δeff. = efflux-defective strain.

Table S8. IC_{50} Values Against *E. coli*, *A. baumannii* and *P. aeruginosa* DNA Gyrase and Topoisomerase IV, MICs, Solubility and Free Fraction Data of Compounds Resulting from Combined Approaches to Modification of Compound 27.

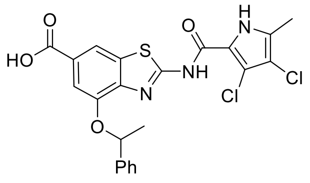

27

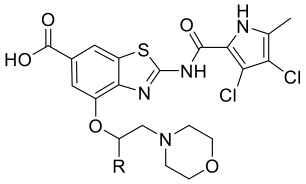

56-58

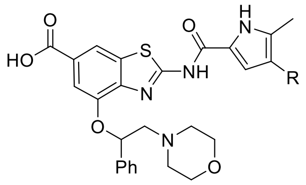

59, **60**

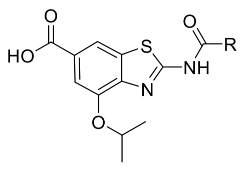

61-63

Cpd.	R	LogD/<br>LogP<br>Calc.	TPSA<br>[Å^2]<br>Calc.	*E. coli* [a]			*A. baumannii* [b]			*P. aeruginosa* [c]			Solu-<br>bility<br>[μM]	Free<br>fraction<br>[%]<br>human/<br>mouse
				Gyrase<br>IC_{50}<br>[nM]	*Topo IV*<br>IC_{50}<br>[nM]	MIC<br>[μg/mL]<br>WT/<br>Δefflux	*Gyrase*<br>IC_{50}<br>[nM]	*Topo IV*<br>IC_{50}<br>[nM][f]	MIC<br>[μg/mL]<br>WT/<br>Δefflux	*Gyrase*<br>IC_{50}<br>[nM]	*Topo IV*<br>IC_{50}<br>[nM][f]	MIC<br>[μg/mL]<br>WT/<br>Δefflux		
27	-	2.59/<br>5.94	104.3	<10	318±94	1/<br>0.125	16±2	6.7±0.5	0.5/<br>0.5	<10	29±1	1/<br>0.125	>80[d]<br>92[e]	0.016/<br>0.01
56	Me	0.91/<br>1.37	116.8	<10	280±27	>64/<br><0.125	<10	460±0	>64/<br>n.d.	<10	107±9	>64/<br>2	62[d]	0.3/<br>1.5
57	CF_3	1.29/<br>2.46	116.8	n.d.	n.d.	>64/<br>0.125	<10	n.d.	8/<br>4	<10	n.d.	>64/<br>2	35[d]	0.13/<br>0.1
58	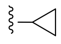	1.44/<br>1.74	116.8	<10	230±48	16/<br>0.125	<10	385±38	64/<br>n.d.	<10	96±1	>64/<br>2	57[d]	0.3/<br>1.3
59	Cl	1.66/<br>2.14	116.8	<10	171±35	16/<br>0.125	<10	44±4	32/<br>0.25	<10	24±8	16/<br>0.125	20[d]<br>70[e]	<0.1/<br>0.5
60	F	1.20/<br>1.68	116.8	<10	39±14	64/<br>0.125	<10	27±2	>64/<br>n.d.	<10	22±1	64/<br>2	67[d]<br>7[e]	1.5/<br>4.4
61	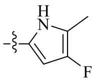	0.16/<br>3.51	104.3	148±63	2200±<br>3156	>64/<br>0.125	120	n.d.	>64/<br>n.d.	29±11	n.d.	>64/<br>>64	3[d]	n.d.
62	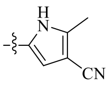	-0.13/<br>3.22	128.1	48±1	n.d.	>64/<br>16	25	n.d.	>64/<br>>64	<10	n.d.	>64/<br>>64	7[d]	n.d.
63	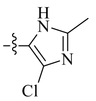	-0.45/<br>2.48	117.2	4290±<br>330		>64/<br>>64	n.d.	n.d.	>64/<br>n.d.	655±195	n.d.	>64/<br>>64	42[d]	0.7[g]/<br>19.9[g] /

[a]*E. coli* ATCC5922 (wild type), CH3130 (efflux-defective; ΔtolC mutant isogenic to ATCC25922); [b]*A. baumannii* ATCC19606 (wild type), BM4652 (efflux-defective derivative of BM4454); [c]*P. aeruginosa* PAO1 (wild type), PAO750 (efflux-defective isogenic to PAO1); [d] kinetic solubility; [e]thermodynamic solubility; [f]gel-based assay; [g]The results should be interpreted carefully due to low recovery; TPSA = total polar surface area; Calc. = calculated; n.d. = not determined; WT = wild type; Δeff. = efflux-defective strain.

Table S9: IC_{50} Values Against *E. coli*, *A. baumannii* and *P. aeruginosa* DNA Gyrase and Topoisomerase IV, MICs, Solubility and Free Fraction Data of Derivatives of Compound 27 With *para* Substituted Phenyl Ring.

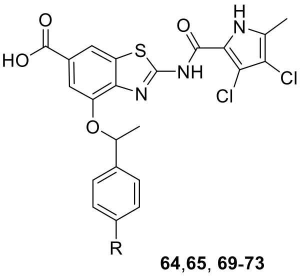

64,**65**, **69-73**

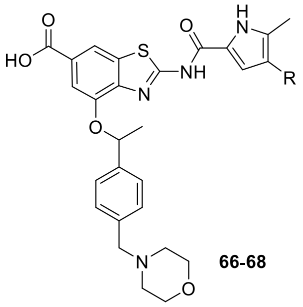

66-68

Cpd.	R	LogD/<br>LogP<br>Calc.	TPSA<br>[Å^2]<br>Calc.	*E. coli* [a]			*A. baumannii* [b]			*P. aeruginosa* [c]			Solu-<br>bility<br>[μM]	Free<br>fraction<br>[%]<br>human/<br>mouse
				Gyrase<br>IC_{50}<br>[nM]	*Topo IV*<br>IC_{50}<br>[nM]	MIC<br>[μg/mL]<br>WT<br>(Δeff.)	*Gyrase*<br>IC_{50}<br>[nM]	*Topo IV*<br>IC_{50}<br>[nM][f]	MIC<br>[μg/mL]<br>WT<br>(Δeff.)	*Gyrase*<br>IC_{50}<br>[nM]	*Topo IV*<br>IC_{50}<br>[nM][f]	MIC<br>[μg/mL]<br>WT<br>(Δeff.)		
**27**	-	2.59/<br>5.94	104	<10	318±94	1<br>(0.125)	16±2	6.7±0.5	0.5<br>(0.5)	<10	29±1	1<br>(0.125)	>80[d]<br>92[e]	0.016/<br>0.01
**64**	SO_2CH_3	1.43/<br>4.78	138	<10	n.d.	32<br>(<0.125)	<10	n.d.	32<br>(n.d.)	<10	n.d.	>64<br>(4)	7[d]	0.01/<br>3.25
**65**	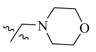	2.56/<br>3.01	117	<10	107±55	4<br>(0.125)	<10	26±1	2<br>(n.d.)	<10	101±3	8<br>(2)	30[d]<br>25[e]	<0.1/<br><0.1
**66**	Cl	1.95/<br>2.41	117	<10	44±1	16<br>(0.125)	<10	132±23	>64<br>(n.d.)	<10	219±37	32<br>(0.125)	15[d]	n.d./<br>n.d.
**67**	F	1.49/<br>1.95	117	<10	34±16	>64<br>(0.125)	<10	201±2	>64<br>(n.d.)	<10	95±3	>64/<br>(0.5)	78[d]<br>17[e]	0.48/<br>1.7
**68**	CN	1.21/<br>1.66	141	13±1	172±52	>64<br>(2)	<10	490±30	>64<br>(n.d.)	<10	238±5	>64/<br>(16)	26[d]	1.7/<br>5.0
**69**	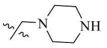	2.85/<br>2.86	120	n.d.	n.d.	>64<br>(2)	<10	145±29	>64<br>(n.d.)	<10	420±18	>64/<br>(>64)	2[d]	<0.1/<br>0.32
**70**	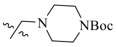	3.45/<br>4.03	137	n.d.	n.d.	>64<br>(2)	21	n.d.	>64<br>(n.d.)	<10	n.d.	>64/<br>(>64)	<1[d]<br>[e]	<0.1[h]/<br><0.1[h]
**71**	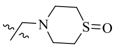	1.12/<br>2.21	125	<10	n.d.	>64<br>(4)	<10	146±16	>64<br>(n.d.)	<10	271±35	>64/<br>(>64)	59 [d]<br>2760[e]	0.11/<br>0.2
**72**	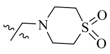	1.19/<br>4.07	142	n.d.	n.d.	16<br>(<0.125)	<10	33±9	>64<br>(n.d.)	<10	217±31	64/<br>(1)	22 [d]	<0.1/<br><0.1
**73**	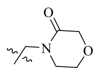	1.41/<br>4.76	134	<10	n.d.	32/<br>0.125	<10	51±1	>64/<br>(n.d.)	<10	200±6	>64/<br>(4)	40[d]<br>7[e]	0.16/<br>0.17

[a]*E. coli* ATCC5922 (wild type), CH3130 (efflux-defective; ΔtolC mutant isogenic to ATCC25922); [b]*A. baumannii* ATCC19606 (wild type), BM4652 (efflux-defective derivative of BM4454); [c]*P. aeruginosa* PAO1 (wild type), PAO750 (efflux-defective isogenic to PAO1); [d]kinetic solubility; [e]thermodynamic solubility; [f]gel-based assay; [g]The results should be interpreted carefully due to low recovery; TPSA = total polar surface area; Calc. = calculated; n.d. = not determined; WT = wild type; Δeff. = efflux-defective strain.

Table S10. Antibacterial Activity of Compounds 1–73 Against *S. aureus* and *K. pneumoniae*

Cpd.	LogD/ LogP Calc.	TPSA [Å^2] Calc.	*S. aureus* [a] MIC[µg/mL]	*K. pneumoniae* [b] MIC[µg/mL]	Solu- bility [µM]	Free fraction [%] human/ mouse
**1**	2.17/ 5.52	104	<0.125	2	<1[d] 6.6[e]	<0.1/ <0.1
**2**	1.14/ 3.27	117	2	>64	44[d]	0.65/ 0.03
**3**	1.06/ 3.33	117	4	>64	<1[d]	0.01/ 0.03
**4**	0.25/ 3.64	130	>64	>64	277	0.01/ 0.05/
**5**	0.13/ 0.36	108	>64	>64	53[d]	0.2/ 10.8
**6**	0.13/ 0.36	108	>64	>64	21[d]	0.1/ 0.2
**8**	1.01/ 4.36	138	>64	>64	<1[d]	<0.1/ 0.27
**9**	2.32/ 5.66	104	0.5	32	<1[d] 8.8[e]	<0.1/ 0.59
**10**	0.40/ 3.75	114	2	>64	204[d]	<0.1/ 0.4
**11**	1.49/ 1.50	108	16	>64	16[d]	n.d./ n.d.
**12**	1.40/ 4.75	104	16	32	36	<0.1/ 1.18
**13**	-/ 4.30	117.8	0.5	>64	n.d.	n.d.
**14**	5.24/ 5.55	117.7	8	>64	n.d.	n.d.
**15**	4.42/ 4.42	122.1	16	>64	n.d.	<0.1/ <0.1
**16**	2.03/ 2.10	130.3	1	>64	<1	<0.1/ <0.1
**17**	3.96/ 4.71	119.3	>64	>64	n.d.	n.d.
**18**	2.04/ 5.39	95.5	<0.125	16	4[d]	<0.1/ <0.1
**19**	1.89/ 1.97	121.5	>64	>64	<1[d]	n.d./ n.d.
**20**	−0.05/ 3.52	106.5	0.5	32	27[d]	1.04/ 4.44[f]
**21**	0.43/ 3.77	95.5	8	>64	33[d]	0.9/ 11.0[f]
**22**	2.09/ 5.20	85.8	0.125	>64	n.d.	n.d./ n.d.
**23**	1.05/ 4.38	120.4	8	>64	<1[d]	0.02/ 0.37
**24**	0.55/ 3.89	137.5	4	64	41[d]	0.07/ 0.07

Table S10. Antibacterial Activity of Compounds 1–73 Against *S. aureus* and *K. pneumoniae* *(Continued)*

Cpd.	LogD/ LogP Calc.	TPSA [Å^2] Calc.	*S. aureus*[a] MIC[µg/mL]	*K. pneumoniae*[b] MIC[µg/mL]	Solubility [µM]	Free fraction [%] human/ mouse
**25**	1.71/ 5.05	103.3	8	>64	<1[d]	0.01/ 0.01
**26**	0.48/ 3.82	86.3	<0.125	16	n.d.	0.34/ 1.43
**27**	2.59/ 5.94	104.3	0.125	4	>80 [d] 92 [e]	0.016/ 0.01
**28**	3.19/ 6.54	104.3	0.5	32	n.d.	<0.1/ <0.1
**29**	2.27/ 5.62	104.3	<0.125	8	n.d.	n.d./ n.d.
**30**	1.54/ 4.89	124.5	4	64	22 [d] 14 [e]	<0.1/ 0.1
**31**	2.19/ 5.53	113.5	<0.125	2	<1 [d] 12 [e]	<0.1/ 0.1
**32**	0.91/ 4.26	138.5	2	>64	16 [d]	0.05/ 4.32
**33**	2.51/ 2.52	130.3	0.5	>64	2 90[d]	0.28/ 0.53
**34**	2.84/ 2.87	107.5	<0.125	8	16 [d]	n.d./ n.d.
**35**	3.71/ 3.72	107.6	0.25	8	14 [d]	n.d./ n.d.
**36**	2.26/ 2.74	116.8	0.125	8	35 [d] 3[e]	<0.1/ 0.3
**37**	2.84/ 2.87	107.5	4	64	10 [d]	n.d./ n.d.
**38**	1.25/ 4.60	133.4	2	>64	<1[d]	0.06/ 0.23[g]
**39**	2.93/ 6.28	142.6	2	4	14 [d]	1.61/ <0.01
**40**	1.78/ 4.99	135.0	4	>64	19[d]	n.d./ n.d.
**41**	2.21/ 2.49	133.6	2	>64	4[d]	n.d./ n.d.
**42**	2.80/ 2.86	110.8	16	>64	4 [d]	<0.1/ 0.3
**43**	3.55/ 3.57	145.9	4	>64	4 [d]	n.d./ n.d.
**44**	2.63/ 2.72	110.8	2	>64	12[d]	n.d./ n.d.
**45**	2.05/ 1.44	107.6	64	>64	23[d]	0.3/ 1.9
**46**	1.22/ 4.57	104	n.d.	n.d.	35 [d]	<0.1/ <0.1
**47**	2.11/ 5.46	104	<0.125	1	29 [d]	<0.1/ <0.1
**48**	1.65/ 4.99	104	<0.125	4	35 [d]	<0.1/ <0.1
**49**	0.35/ 3.70	114	4	>64	35[d]	0.03/ 0.12

Table S10. Antibacterial Activity of Compounds 1–73 Against *S. aureus* and *K. pneumoniae* *(Continued)*

Cpd.	LogD/ LogP Calc.	TPSA [Å²] Calc.	*S. aureus*[a] MIC[µg/mL]	*K. pneumoniae*[b] MIC[µg/mL]	Solu-bility [µM]	Free fraction [%] human/ mouse
**50**	1.99/ 5.33	104	4	64	23 [d] 19 [e]	<0.1/ <0.1
**51**	1.52/ 4.87	104	0.25	4	41 [d] 18 [e]	0.13/ 0.3
**52**	1.52/ 4.87	104	8	>64	<1[d] 7[e]	0.17/ 0.7
**53**	1.52/ 4.87	104	0.5	>64	52 [d] 20 [e]	0.19/ 0.7
**54**	1.24/ 4.59	128	64	>64	57 [d]	0.4/ 1.2
**55**	1.38/ 4.73	104	4	>64	3[d]	n.d./ n.d.
**56**	0.91/ 1.37	116.8	32	>64	62[d]	0.3/ 1.5
**57**	1.29/ 2.46	116.8	0.5	>64	35[d]	0.13/ 0.1
**58**	1.44/ 1.74	116.8	4	64	57[d]	0.3/ 1.3
**59**	1.66/ 2.14	116.8	<0.125	8	20[d] 70[e]	<0.1/ 0.5
**60**	1.20/ 1.68	116.8	64	>64	67[d] 7[e]	1.5/ 4.4
**61**	0.16/ 3.51	104.3	>64	>64	3[d]	n.d./ n.d.
**62**	-0.13/ 3.22	128.1	>64	>64	7[d]	n.d./ n.d.
**63**	-0.45/ 2.48	117.2	>64	>64	42[d]	0.7/ 19.9
**64**	1.43/ 4.78	138	2	>64	7[d]	0.01/ 3.25
**65**	2.56/ 3.01	117	0.125	8	30[d] 25[e]	<0.1/ <0.1
**66**	1.95/ 2.41	117	16	64	15[d]	n.d./ n.d.
**67**	1.49/ 1.95	117	64	>64	78[d] 17[e]	0.48/ 1.7
**68**	1.21/ 1.66	141	64	>64	26[d]	1.7/ 5.0
**69**	2.85/ 2.86	120	>64	>64	2[d]	<0.1/ 0.32
**70**	3.45/ 4.03	137	0.5	>64	<1[d]	<0.1/ <0.1
**71**	1.12/ 2.21	125	>64	>64	59 [d] 2760[e]	0.11/ 0.2
**72**	1.19/ 4.07	142	1	64	22 [d]	<0.1/ <0.1
**73**	1.41/ 4.76	134	8	64	40[d] 7[e]	0.16/ 0.17

[a] *S. aureus* ATCC29213 (wild type); [b]*K. pneumoniae* ATCC13883 (wild type); [d] kinetic solubility; [e]thermodynamic solubility; [g]The results should be interpreted carefully due to low recovery; TPSA = total polar surface area; Calc. = calculated; n.d. = not determined; WT = wild type; Δeff. = efflux-defective strain.

Table S11. Plasma Protein Binding of Selected Optimized Derivatives of Compounds 1 and 27.

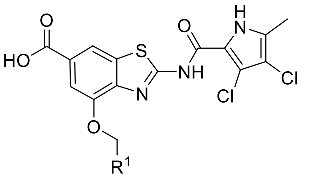

1, **2**, **9**, **12**

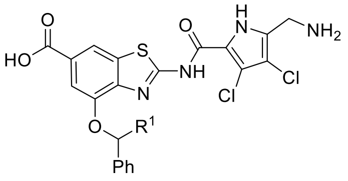

16, **55**

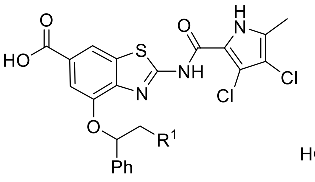

27, **36**, **45**

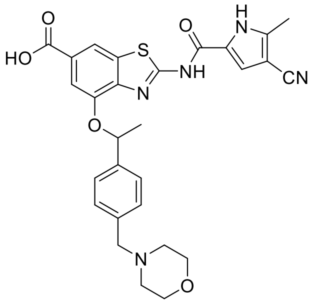

68

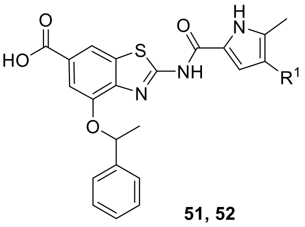

51, **52**

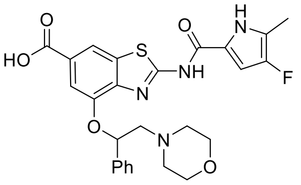

60

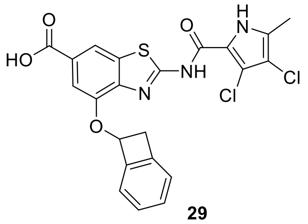

29

Cpd.	R[1]	LogD/ LogP Calc.	TPSA[Å^2] Calc.	*E. coli* [a]			*A. baumannii* [b]			*P. aeruginosa* [c]			Solu-bility	**Free fraction**	
				Gyrase IC_{50} [nM]	*Topo IV* IC_{50} [nM]	MIC[µg/ mL]	*Gyrase* IC_{50} [nM]	*Topo IV* IC_{50} [nM][f]	MIC [µg/ mL]	*Gyrase* IC_{50} [nM]	*Topo IV* IC_{50} [nM][f]	MIC [µg/mL]		Human [%]	Mouse [%]
**1**	Ph	2.17/ 5.82	104.3	<10	350	4	<10	64	0.5	<10	235	8	<1[d] 6.6[e]	<0.1	<0.1
**2**	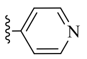	1.14/ 3.27	117.2	<10	n.d.	>64	<10	n.d.	>64	<10	n.d.	>64	44[d]	0.65	0.03
**9**	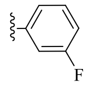	2.32/ 5.66	104.3	<10	54±28	>64	<10	n.d.	4	<10	n.d.	>64	<1[d] 8.8 [e]	<0.1	0.59
**12**	CF_3	1.40/ 4.75	104.3	<10	n.d.	32	<10	155±5	8	<10	255±35	64	36	<0.1	1.18
**16**	H	2.03/ 2.10	130.3	<10	not active	>64	490	3020±640	32	1140	5160±585	64	<1[d]	0.59	<0.1[g]
**29**	-	0.15/ 3.63	133.21	<10	n.d.	>64	<10	n.d.	>64	<10	n.d.	>64	<1[d]	0.53	3.83
**27**	H	2.59/ 5.94	104.3	<10	318±94	1	16±2	6.7±0.5	0.5	<10	29±1	1	36 [d] 92 [e]	0.016	0.01
**36**	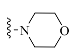	2.26/ 2.74	116.8	<10	-	2	<10	11±2	2	<10	28±1	8	35 [d] 3[e]	<0.1	0.3[g]
**45**	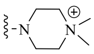	2.05/ 1.44	107.6	n.d.	n.d.	>64	n.d.	n.d.	64	n.d.	n.d.	>64	23[d]	0.3	1.9
**51**	F	1.52/ 4.87	104.3	<10	37±9	4	<10	7.8±1.1	2	<10	9.3±1.1	4	41[d] 18[e]	0.13	0.3
**52**	CN	1.24/ 4.59	128.1	<10	126±22	>64	<10	167±15	>64	<10	29±2	>64	57[d]	0.4	1.2
**55**	Me	2.44/ 2.52	130.3	41±12	18500 ±2080	>64	n.d.	n.d.		n.d.	n.d.		2[d]	1.28	0.3
**60**	F	1.20/ 1.68	116.8	<10	39±14	64	<10	27±2	>64	<10	22±1	64	67[d] 7[e]	1.5	4.4
**68**	CN	1.21/ 1.66	140.6	<10	172±29	>64	<10	490±30	>64	<10	238±5	>64	26 [d]	1.7	5.0

[a]*E. coli* ATTN25922; [b]*A. baumannii* ATTN19606; [c]*P. aeruginosa* PA01; [d] kinetic solubility; [e]thermodynamic solubility; [f]gel-based assay; [g] The results should be interpreted carefully due to low recovery; n.d. = not determined.

Table S12. X-ray Data Collection and Refinement Statistics for Inhibitor Complexes of *E. coli* GyrB24

Inhibitor	Compound **1**	Compound **7**
Data collection		
Beamline	I03 DLS, UK	I03 DLS, UK
Wavelength (Å)	0.9763	0.9762
Detector	Pilatus3 6M	Eiger2 XE 16M
Resolution range (Å)	51.50 – 1.16 (1.18 – 1.16)	52.62 – 1.65 (1.68 – 1.65)
Space Group	*C*2	$P2_12_12_1$
Cell parameters (Å/°)	*a* = 76.7, *b* = 50.3, *c* = 52.7, *β* = 102.3	*a* = 49.8, *b* = 52.6, *c* = 81.5
Total no. of measured intensities	433042 (17809)	339970 (17285)
Unique reflections	65952 (3124)	26432 (1263)
Multiplicity	6.6 (5.7)	12.9 (13.7)
Mean $I/\sigma(I)$	12.5 (1.1)	9.4 (1.2)
Completeness (%)	97.2 (93.0)	99.8 (99.8)
R_{merge}[a]	0.064 (1.387)	0.196 (2.294)
R_{meas}[b]	0.070 (1.528)	0.205 (2.382)
$CC_{½}$[c]	0.999 (0.465)	0.997 (0.581)
Wilson *B* value (Å^2)	11.3	18.3
Refinement		
Resolution range (Å)	41.81 – 1.16 (1.19 – 1.16)	44.24 – 1.65 (1.69 – 1.65)
Reflections: working/free[d]	62697/3244	25156/1221
R_{work}/ R_{free}[e]	0.134/0.161 (0.272/0.269)	0.188/0.221 (0.316/0.341)
Ramachandran plot: favoured/allowed/disallowed[f] (%)	96.3/3.2/0.5	96.5/2.5/1.0
R.m.s. bond distance deviation (Å)	0.011	0.010
R.m.s. bond angle deviation (°)	1.58	1.52
RSCC for inhibitor[g]	0.99	0.98
Protein residues (ranges)	193 (8-98, 117-218)	204 (14-111, 114-219)
No. of water/inhibitor/other[h]	210/1/1	197/1/2
Mean *B* factors: protein/water/inhibitor/other (Å^2)	20/36/18/29	26/36/21/37
Accession code	7P2M	7P2W

Values in parentheses are for the outer resolution shell.

[a] Rmerge = ∑hkl ∑i |Ii(hkl) – ⟨I(hkl)⟩|/ ∑hkl ∑iIi(hkl).

[b] Rmeas = ∑hkl [N/(N – 1)]1/2 × ∑i |Ii(hkl) – ⟨I(hkl)⟩|/ ∑hkl ∑iIi(hkl), where Ii(hkl) is the ith observation of reflection hkl, ⟨I(hkl)⟩ is the weighted average intensity for all observations i of reflection hkl and N is the number of observations of reflection hkl.

[c] CC½ is the correlation coefficient between symmetry equivalent intensities from random halves of the dataset.

[d] The data set was split into "working" and "free" sets consisting of 95 and 5% of the data respectively. The free set was not used for refinement.

[e] The R-factors Rwork and Rfree are calculated as follows: R = ∑(| Fobs - Fcalc |)/∑| Fobs |, where Fobs and Fcalc are the observed and calculated structure factor amplitudes, respectively.

[f] As calculated using MolProbity, *Nucleic Acids Res.* **2007**, *35*, W375-383.

[g] Real Space Correlation Coefficients from PDB validation report indicating density fit of ligand.

[h] Other = buffer components

Table S13. X-ray Data Collection and Refinement Statistics for Inhibitor Complexes of *Acinetobacter baumannii* GyrB23

Inhibitor	Novobiocin	Compound **27**	Compound **(*S*)-27**
Data collection			
Beamline	I24 DLS, UK	I04 DLS, UK	I04 DLS, UK
Wavelength (Å)	0.9999	0.9795	0.9795
Detector	Pilatus3 6M	Eiger2 XE 16M	Eiger2 XE 16M
Resolution range (Å)	64.13 – 1.90 (1.94 – 1.90)	43.65 – 1.60 (1.63 – 1.60)	43.54 – 1.55 (1.58 – 1.55)
Space Group	$P4_12_12$	$C2$	$C2$
Cell parameters (Å/°)	$a = b = 42.9$, $c = 256.2$	$a = 121.1$, $b = 48.4$, $c = 83.3$, $\beta = 124.2$	$a = 120.6$, $b = 48.3$, $c = 82.7$, $\beta = 123.8$
Total no. of measured intensities	530228 (34649)	345218 (11402)	626669 (30739)
Unique reflections	20179 (1269)	52822 (2479)	57534 (2777)
Multiplicity	26.3 (27.3)	6.5 (4.6)	10.9 (11.1)
Mean $I/\sigma(I)$	8.8 (1.2)	13.1 (0.6)	8.9 (0.5)
Completeness (%)	100.0 (100.0)	99.7 (94.3)	99.9 (99.0)
R_{merge}[a]	0.223 (2.620)	0.090 (1.638)	0.105 (1.550)
R_{meas}[b]	0.228 (2.668)	0.098 (1.853)	0.110 (1.625)
$CC_{½}$[c]	0.998 (0.894)	0.998 (0.427)	0.999 (0.660)
Wilson B value ($Å^2$)	29.3	20.1	18.6
Refinement			
Resolution range (Å)	64.13 – 1.90 (1.95 – 1.90)	43.65 – 1.60 (1.64 – 1.60)	43.54 – 1.55 (1.58 – 1.55)
Reflections: working/free[d]	19078/989	50096/2724	54572/2969
R_{work}/ R_{free}[e]	0.217/0.237 (0.320/0.357)	0.161/0.219 (0.312/0.315)	0.180/0.224 (0.342/0.380)
Ramachandran plot: favoured/allowed/disallowed[f] (%)	98.5/1.5.0.0	98.3/1.7/0.0	97.8/2.2/0.0
R.m.s. bond distance deviation (Å)	0.008	0.006	0.006
R.m.s. bond angle deviation (º)	1.44	1.28	1.24
RSCC for inhibitor(s)[g]	A: 0.95	A: 0.98, B: 0.99	A: 0.98, B: 0.98
Protein residues per chain (ranges)	A: 198 (28-117, 126-233)	A: 185 (29-112, 131-231)<br>B: 185 (30-112, 130-231)	A: 184 (29-112, 132-231)<br>B: 185 (30-112, 130-231)
No. of molecules: water/inhibitor/other[h]	70/1/5	321/2/0	354/2/1
Mean B factors: protein/water/inhibitor/other ($Å^2$)	38/43/30/51	30/40/31/-	34/45/35/31
Accession code	7PQI	7PQL	7PQM

Values in parentheses are for the outer resolution shell.

[a] Rmerge = ∑hkl ∑i |Ii(hkl) – ⟨I(hkl)⟩|/ ∑hkl ∑iIi(hkl).

[b] Rmeas = ∑hkl [N/(N – 1)]1/2 × ∑i |Ii(hkl) – ⟨I(hkl)⟩|/ ∑hkl ∑iIi(hkl), where Ii(hkl) is the ith observation of reflection hkl, ⟨I(hkl)⟩ is the weighted average intensity for all observations i of reflection hkl and N is the number of observations of reflection hkl.

[c] CC½ is the correlation coefficient between symmetry equivalent intensities from random halves of the dataset.

[d] The data set was split into "working" and "free" sets consisting of 95 and 5% of the data respectively. The free set was not used for refinement.

[e] The R-factors Rwork and Rfree are calculated as follows: R = ∑(| Fobs - Fcalc |)/∑| Fobs |, where Fobs and Fcalc are the observed and calculated structure factor amplitudes, respectively.

[f] As calculated using MolProbity, *Nucleic Acids Res.* **2007**, *35*, W375-383.

[g] Real Space Correlation Coefficients from PDB validation report indicating density fit of ligand.

[h] Other = buffer components

Table S14. X-ray Data Collection and Refinement Statistics for Inhibitor Complexes of *Pseudomonas aeruginosa* GyrB24

Inhibitor	Novobiocin	Compound **(*S*)-27**
Data collection		
Beamline	I04-1	I03
Wavelength (Å)	0.9126	0.9796
Detector	Pilatus 6M-F	Eiger2 XE 16M
Resolution range (Å)	109.11 – 1.32 (1.34 – 1.32)	41.89 – 2.20 (2.27 – 2.20)
Space Group	$P2_1$	$P2_1$
Cell parameters (Å/°)	*a* = 62.7, *b* = 47.4, *c* = 110.6, *β* = 99.5	*a* = 52.6, *b* = 47.9, *c* = 75.3, *β* = 93.5
Total no. of measured intensities	977797 (39772)	130013 (10689)
Unique reflections	150584 (7368)	19239 (1638)
Multiplicity	6.5 (5.4)	6.8 (6.5)
Mean $I/\sigma(I)$	9.9 (1.0)	5.9 (1.5)
Completeness (%)	100.0 (99.8)	100.0 (100.0)
R_{merge}[a]	0.071 (1.249)	0.219 (1.193)
R_{meas}[b]	0.078 (1.386)	0.237 (1.296)
$CC_{½}$[c]	0.999 (0.474)	0.991 (0.646)
Wilson *B* value (Å^2)	15.1	25.9
Refinement		
Resolution range (Å)	109.11 – 1.32 (1.35 – 1.32)	41.89 – 2.20 (2.27 – 2.20)
Reflections: working/free[d]	143035/7512	18270/953
R_{work}/ R_{free}[e]	0.148/0.177 (0.289/0.302)	0.194/0.236 (0.278/0.355)
Ramachandran plot: favoured/allowed/disallowed[f] (%)	98.7/1.3/0.0	98.0/2.0/0.0
R.m.s. bond distance deviation (Å)	0.008	0.010
R.m.s. bond angle deviation (º)	1.31	1.52
RSCC for inhibitor(s)[g]	A: 0.98, B: 0.97, C: 0.95	A: 0.98, B: 0.97
Protein residues per chain (ranges)	A: 212 (10-221), B 212 (9-220), C: 205 (17-221)	A: 183 (17-100, 121-219), B: 183 (19-101, 121-220)
No. of molecules: water/inhibitor/other[h]	642/3/3	92/2/0
Mean *B* factors: protein/water/inhibitor/other (Å^2)	22/34/18/26	16/31/17/-
Accession code	7PTF	7PTG

Values in parentheses are for the outer resolution shell.

[a] Rmerge = ∑hkl ∑i |Ii(hkl) – ⟨I(hkl)⟩|/ ∑hkl ∑iIi(hkl).

[b] Rmeas = ∑hkl [N/(N – 1)]1/2 × ∑i |Ii(hkl) – ⟨I(hkl)⟩|/ ∑hkl ∑iIi(hkl), where Ii(hkl) is the ith observation of reflection hkl, ⟨I(hkl)⟩ is the weighted average intensity for all observations i of reflection hkl and N is the number of observations of reflection hkl.

[c] CC½ is the correlation coefficient between symmetry equivalent intensities from random halves of the dataset.

[d] The data set was split into "working" and "free" sets consisting of 95 and 5% of the data respectively. The free set was not used for refinement.

[e] The R-factors Rwork and Rfree are calculated as follows: R = ∑(| Fobs - Fcalc |)/∑| Fobs |, where Fobs and Fcalc are the observed and calculated structure factor amplitudes, respectively.

[f] As calculated using MolProbity, *Nucleic Acids Res.* **2007**, *35*, W375-383.

[g] Real Space Correlation Coefficients from PDB validation report indicating density fit of ligand.

[h] Other = buffer components

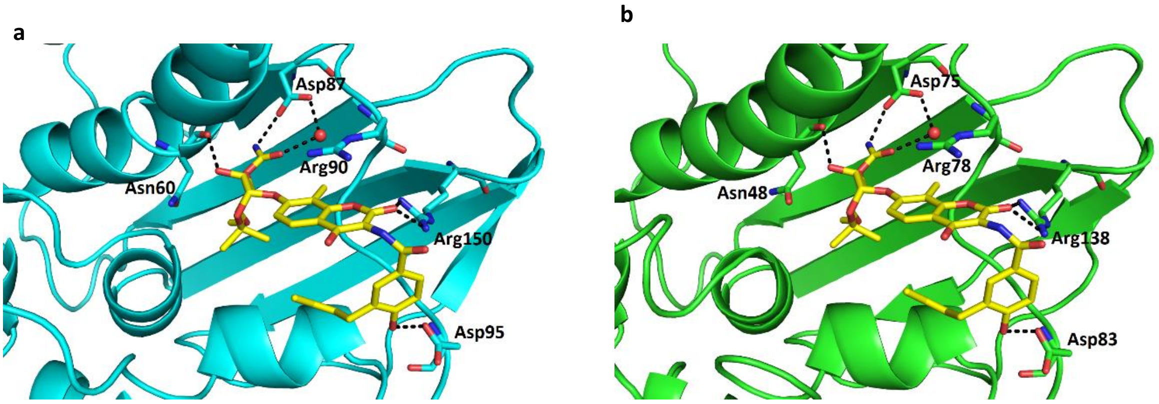

Figure S1. Co-crystal structures of novobiocin in complex with A. baumannii GyrB23 and P. aeruginosa GyrB24. (a) *A. baumannii* GyrB23 (in cyan cartoon, PDB 7PQI) and (b) *P. aeruginosa* GyrB24 (in green cartoon, PDB 7PTF). Novobiocin is represented in yellow sticks. For clarity, only amino acid residues that interact with novobiocin are shown as sticks. Water molecules are presented as red spheres and hydrogen bonds are shown as dashed black lines.

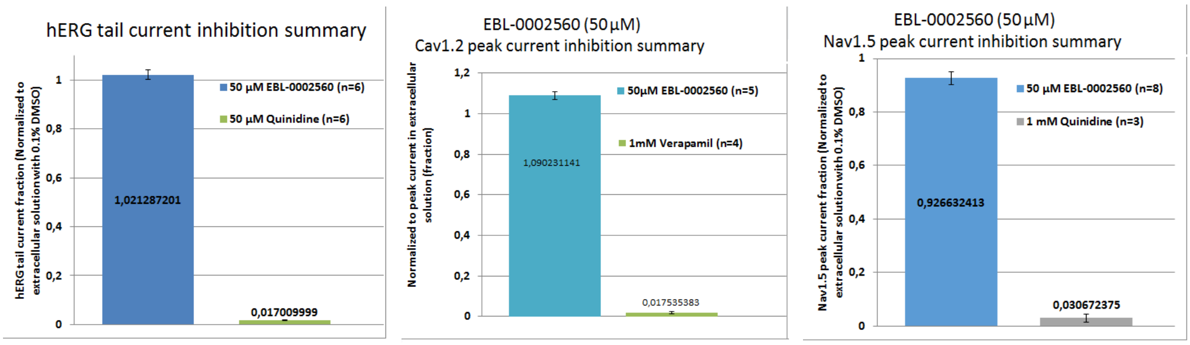

Figure S2. hERG, Cav1.2 and Nav1.5 ion channel inhibition assay for Compound 1 (EBL-2560).

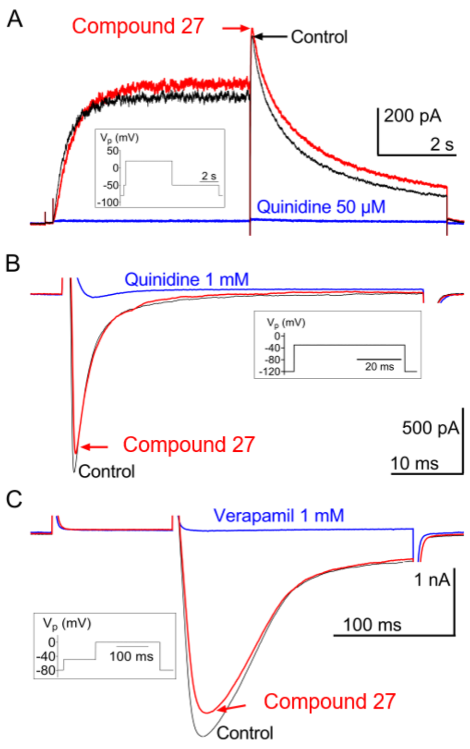

Figure S3. hERG (A), Nav1.5 (B) and Cav1.2 (C) ion channel inhibition assay for Compound 27.

Representative recordings of the hERG tail current (A), Nav1.5 (B) and Cav1.2 (C) currents when the Compound **27** was applied (red). Control recordings are shown in black. Inhibition by specific channel blockers served as positive controls (blue). The stimulation protocol, shown as inset in each panel, evoked the respective currents. Vp, pipette potential.

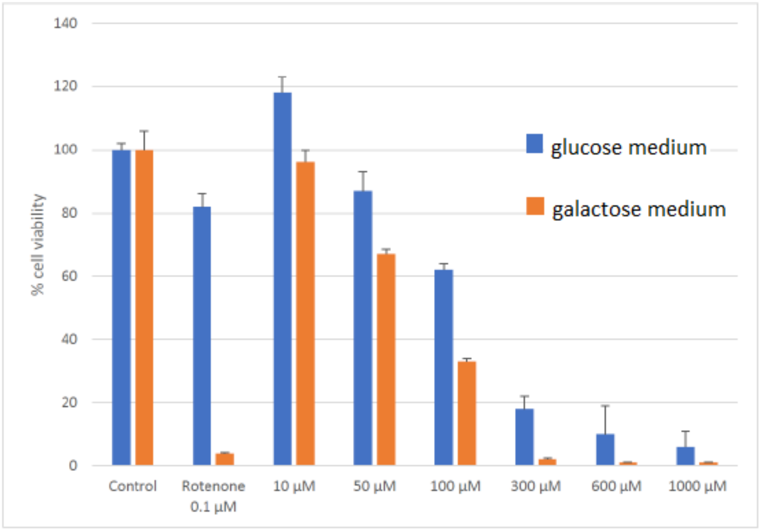

Figure S4. Mitochondrial toxicity assay of Compound 1 in HepG2 cells.

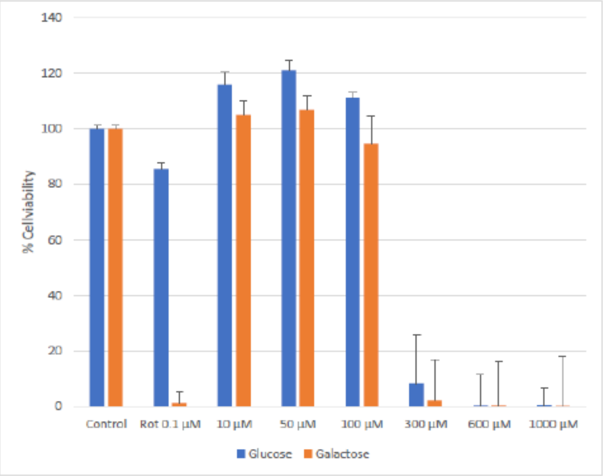

Figure S5. Mitochondrial toxicity assay of Compound 27 in HepG2 cells.

A3070 (EBL-2560)

Sample	*Concentration*	*No. binucleate cells*	*% Cytostasis (± STD)*	*% MN (± STD)*
CTR		2094	-	2.8 ± 1.5
A3070	50 µM	-	55.0 ± 4.3 (TOX)	1.6 ± 2.6
A3070	25 µM	1649	16.6±7.9	2.4 ± 2.1
A3070	12 µM	1889	9.3 ± 11.0	2.8 ± 1.9
A3070	6 µM	2021	-	2.4 ± 1.7
A3070	3 µM	2054	-	2.1 ± 0.7

Figure S6. Micronucleus test (MNT) results for Compound 1 (EBL2560).
TOX indicates high toxicity in the culture observed de visu by microscopy.

Sample	*Concentration*	*No. binucleate cells*	*% Cytostasis (± STD)*	*% MN (± STD)*
CTR		2094	-	2.8 ± 1.5
A3069	150 µM	-	TOX	
A3069	75 µM	-	59±13 (TOX)	1.1 ± 2.7
A3069	37 µM	1803	26.2±8.7	2.6 ± 2.0
A3069	18 µM	1894	0.1±16.5	2.6 ± 1.5
A3069	9 µM	1998	-	2.7 ± 1.2

Figure S7. Micronucleus test (MNT) results for Compound 18.
TOX indicates high toxicity in the culture observed de visu by microscopy.

Table 1 (without metabolic activation)

Sample	*Concentration*	*No. binucleate cells*	*% Cytostasis (± STD)*	*% MN (± STD)*
**CTR-**		**1972**	**-**	**2.0 ± 0.8**
EBL-0002704	150 µM	-	TOX	-
EBL-0002704	75 µM	703	51.1 ± 22.3 (TOX)	1.9 ± 2.5
EBL-0002704	37.5 µM	1446	6.9 ± 16.1	1.5 ± 1.6
EBL-0002704	18.75 µM	1518	-	1.3 ± 0.8

Table 2 (with metabolic activation)

Sample	*Concentration*	*No. binucleate cells*	*% Cytostasis (± STD)*	*% MN (± STD)*
**CTR-**		**2026**	**-**	**4.9 ± 1.0**
EBL-0002704	150 µM	1961	-	4.7 ± 1.3
EBL-0002704	75 µM	2176	-	4.2 ± 1.5
EBL-0002704	37.5 µM	2143	-	4.9 ± 1.7

Figure S8. Micronucleus test (MNT) results for Compound 27 (EBL-2704).
TOX indicates high toxicity in the culture observed de visu by microscopy.

Plate Number	Plate Description	Bacterial Strain	Number of Positively Scored Wells				
			Day 3	Day 4	Day 5	Day 6	Day 7
1	Background	TA 100	8	19	25	41	44
2	Positive Control: NaN3	TA 100	81	95	96	96	96
3	Blanks	TA 100	0	0	0	0	0
4	EBL2560 (LMD43) 25 uM	TA 100	0	0	0	0	0
5	EBL2560 (LMD43) 12.25 uM	TA 100	0	0	0	0	0
6	EBL2560 (LMD43) 6.3 uM	TA 100	0	0	0	0	0
7	EBL-2560 (LMD43) 2 uM	TA 100	0	0	0	0	0
8	Background S9	TA 100+S9	25	27	31	32	35
9	Positive Control: 2-AA S9	TA 100+S9	81	84	85	85	85
10	Blanks S9	TA 100+S9	0	0	0	0	0
11	EBL2560 (LMD43) 25 uM S9	TA 100+S9	0	0	0	0	0
12	EBL2560 (LMD43) 12.25 uM S9	TA 100+S9	0	0	0	0	0
13	EBL2560 (LMD43) 6.3 uM S9	TA 100+S9	0	0	0	0	0
14	EBL2560 (LMD43) 2 uM S9	TA 100+S9	0	0	0	0	0

Figure S9. Mutagenic activity of Compound 1 (EBL-2560) using *S. typhimurium* TA100 with and without S9 activation scored on days 3 to 7 of growth (AMES test).

TA100 +S9					
Sample	Conc. (µM)	Number positive wells			Mutagenic Conc. / Cytotoxic Effect
		Replicate #1	Replicate #2	Replicate #3	
EBL2704 (vial 3136)	0	6	5	9	
	0.1	9	8	5	
	3	2	2	4	Cytotoxic effect?
	6	2	1	5	Cytotoxic effect?
	12.25	1	1	3	Cytotoxic effect?
	25	1	0	0	Cytotoxic effect?
2-AA	2.5 µg/mL	48	48	48	Mutagenic Conc.

TA100 -S9					
Sample	Conc. (µM)	Number positive wells			Mutagenic Conc. / Cytotoxic Effect
		Replicate #1	Replicate #2	Replicate #3	
EBL2704 (vial 3136)	0	3	3	3	
	0.1	4	2	6	
	3	4	3	5	
	6	1	3	2	
	12.25	2	1	4	
	25	0	0	0	Cytotoxic effect?
4-NQQ	0.1 µg/mL	48	48	47	Mutagenic Conc.

Figure S10. Mutagenic activity of Compound 27 (EBL-2704) using *S. typhimurium* TA100 with (top) and without (bottom) S9 activation (AMES test). Positive controls: 4-NQQ = 4-nitroquinoline-*N*-oxide; 2-AA = 2-aminoanthracene.

TA98 -S9					
Sample	Conc. (µM)	Number positive wells			Mutagenic Conc. / Cytotoxic Effect
		Replicate #1	Replicate #2	Replicate #3	
EBL2837 (vial 3137)	0	1	0	0	
	0.1	0	0	0	
	3	0	0	0	
	6	0	0	0	
	12.25	0	0	0	
	25	0	0	0	
2-NF	2 µg/mL	34	36	33	Mutagenic Conc.

TA98 +S9					
Sample	Conc. (µM)	Number positive wells			Mutagenic Conc. / Cytotoxic Effect
		Replicate #1	Replicate #2	Replicate #3	
EBL2837 (vial 3137))	0	0	2	3	
	0.1	1	1	2	
	3	0	0	0	Cytotoxic effect?
	6	0	0	0	Cytotoxic effect?
	12.25	0	0	0	Cytotoxic effect?
	25	0	0	0	Cytotoxic effect?
2-AA	1 µg/mL	42	45	44	Mutagenic Conc.

Figure S11. Mutagenic activity of Compound 31 (EBL-2837) using *S. typhimurium* TA98 with (top) and without (bottom) S9 activation (AMES test). Positive controls: 2-NF = 2-nitrofluorene; 2-AA = 2-aminoanthracene.

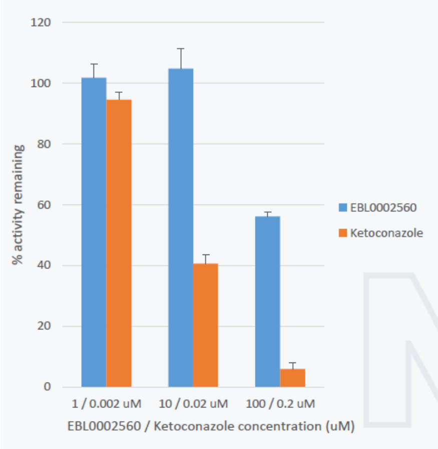

Figure S12. CYP3A4 inhibition assay of Compound 1.

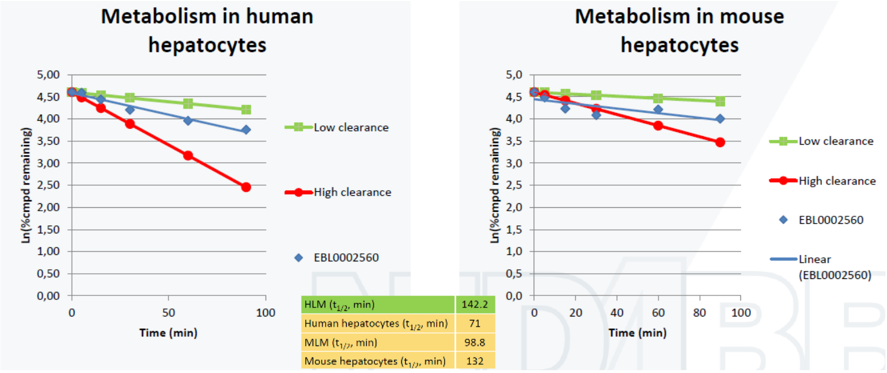

HLM ($t_{1/2}$, min)	142.2
Human hepatocytes ($t_{1/2}$, min)	71
MLM ($t_{1/2}$, min)	98.8
Mouse hepatocytes ($t_{1/2}$, min)	132

Figure S13. Metabolism of Compound 1 (EBL-2560) in human and mouse hepatocytes, and human (HLM) and mouse (MLM) liver microsomes.

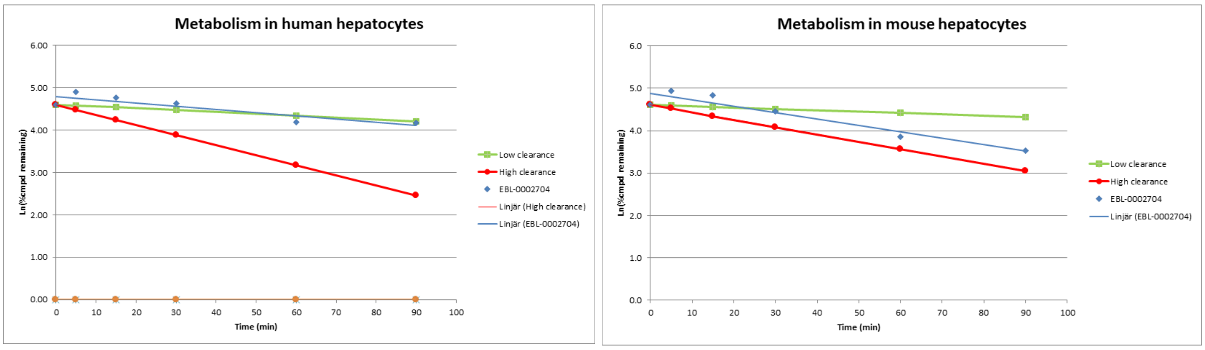

Figure S14. Metabolism of Compound 27 (EBL-2704) in human (left) and mouse (right) hepatocytes.

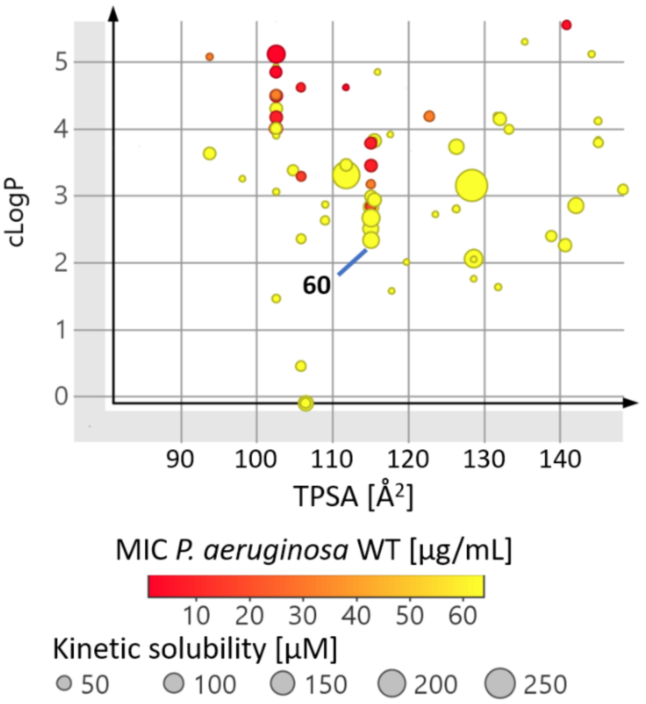

Figure S15. Relationship between cLogP, TPSA, kinetic solubility, and MIC against *P. aeruginosa*.

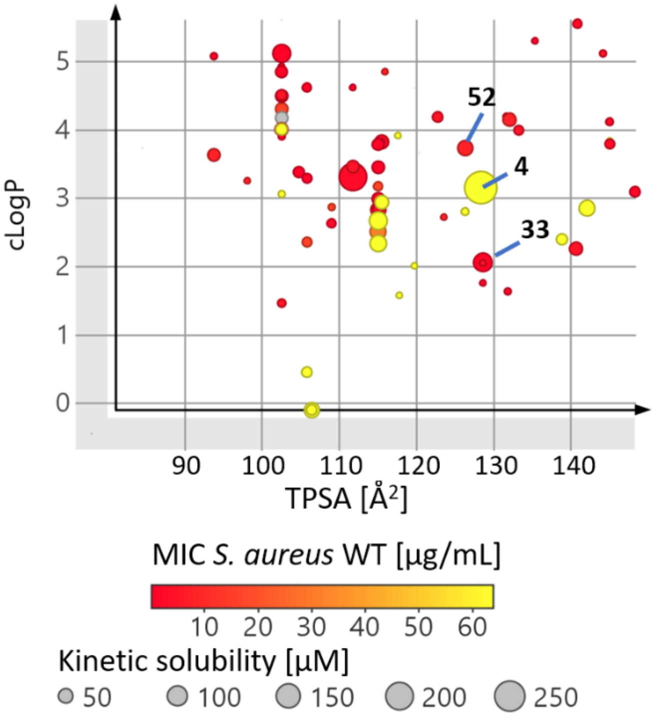

Figure S16. Relationship between cLogP, TPSA, kinetic solubility, and MIC against *S. aureus*.

NMR Spectra

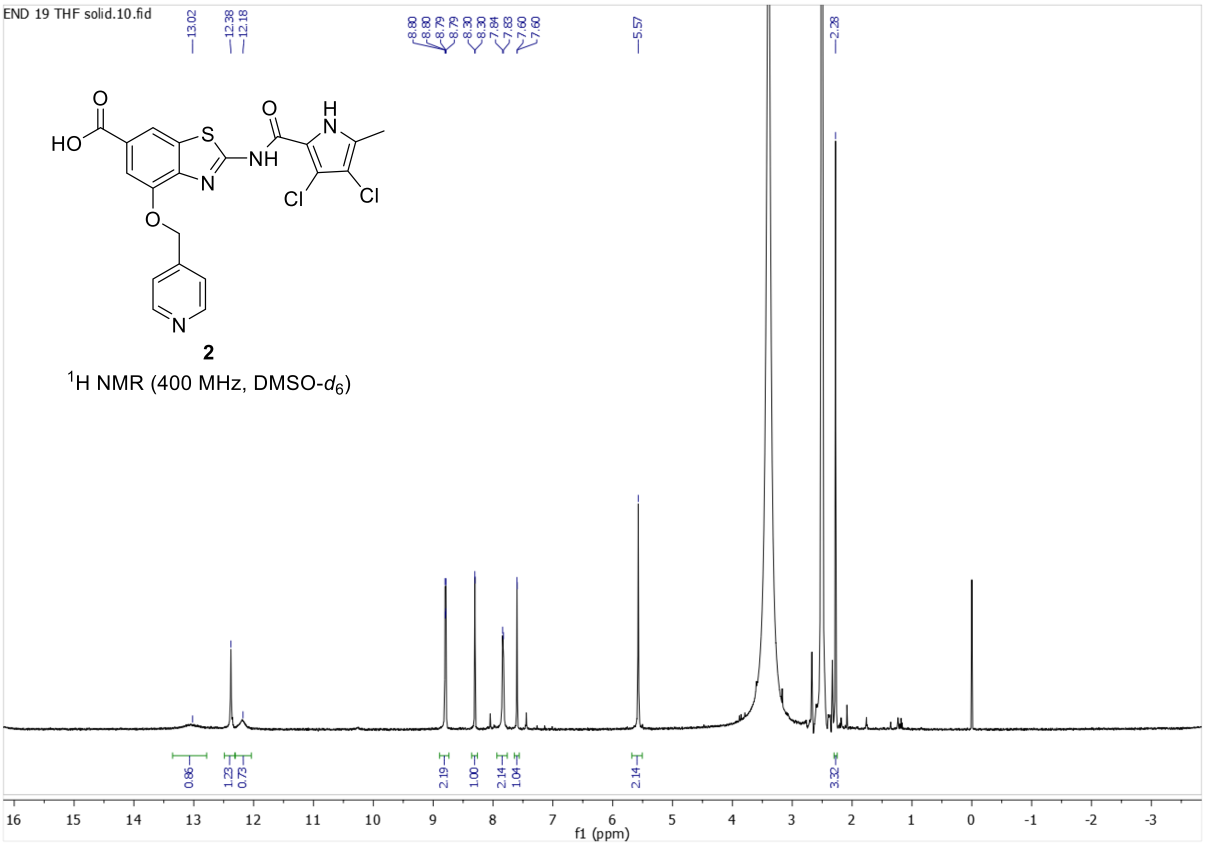
END 19 THF solid.10.fid
2
^{1}H NMR (400 MHz, DMSO-d_6)
f1 (ppm)

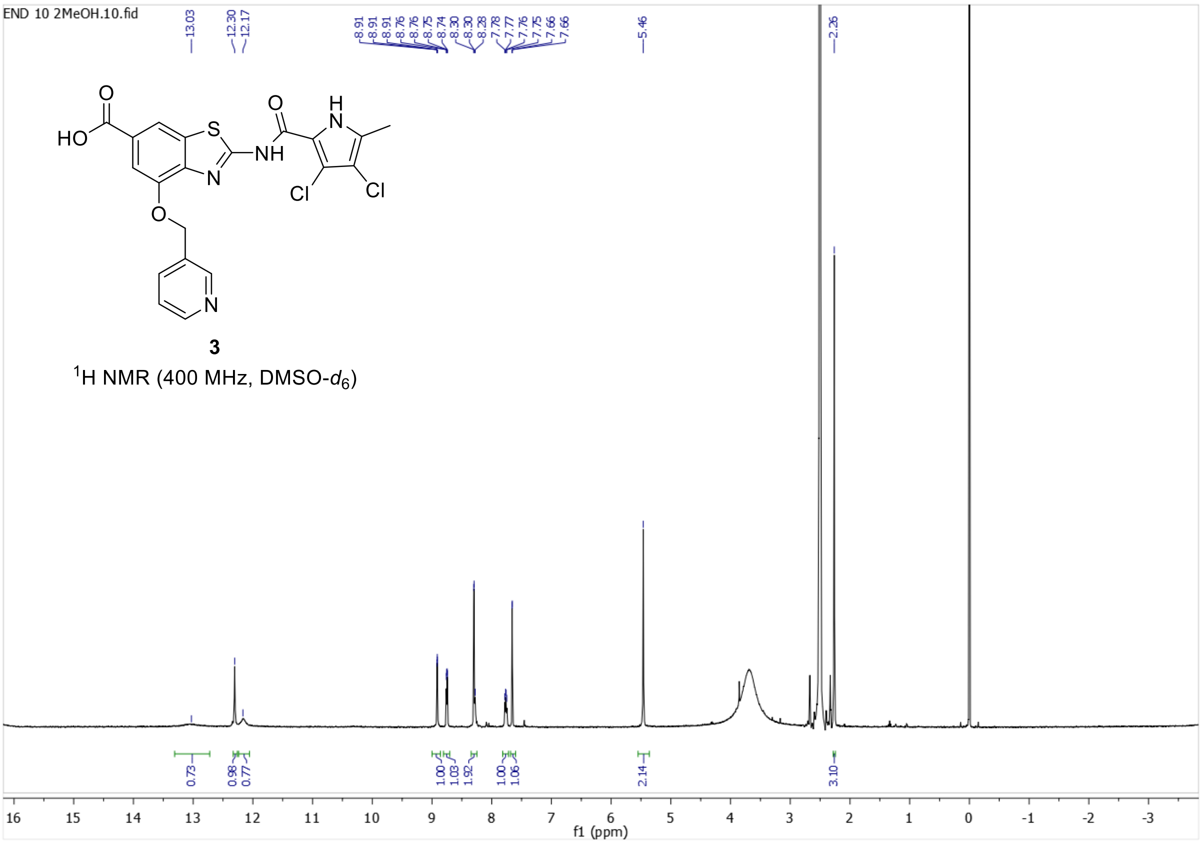
END 10 2MeOH.10.fid
3
^{1}H NMR (400 MHz, DMSO-d_6)
f1 (ppm)

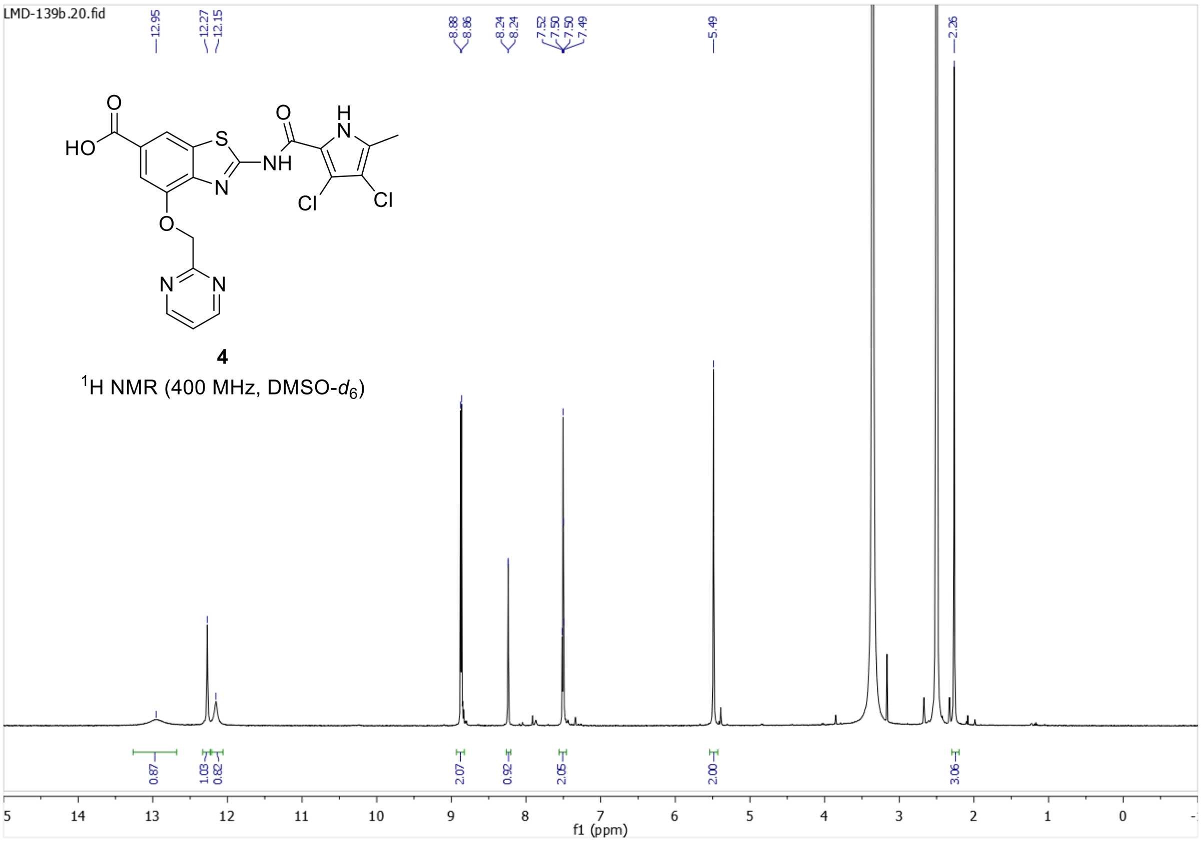
LMD-139b.20.fid
4
1H NMR (400 MHz, DMSO-d6)
f1 (ppm)

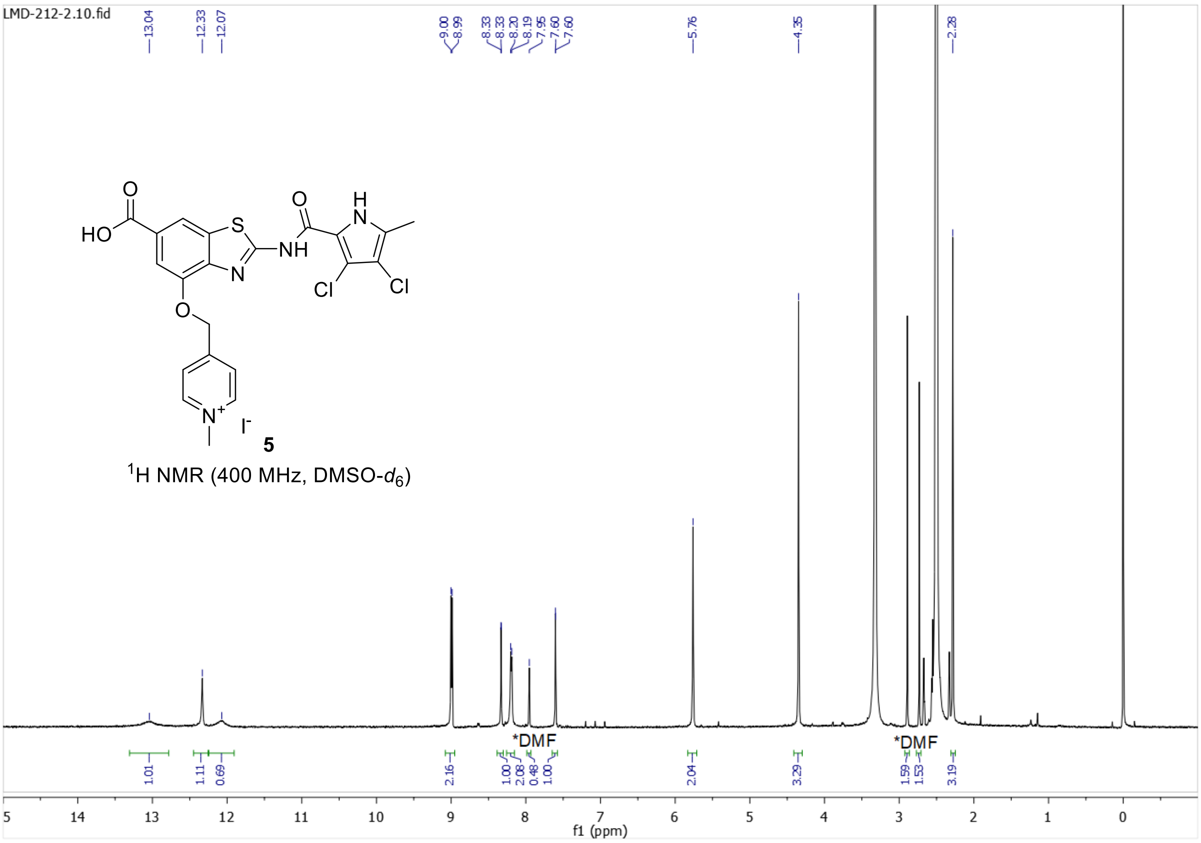
LMD-212-2.10.fid
5
1H NMR (400 MHz, DMSO-d6)
*DMF
*DMF
f1 (ppm)

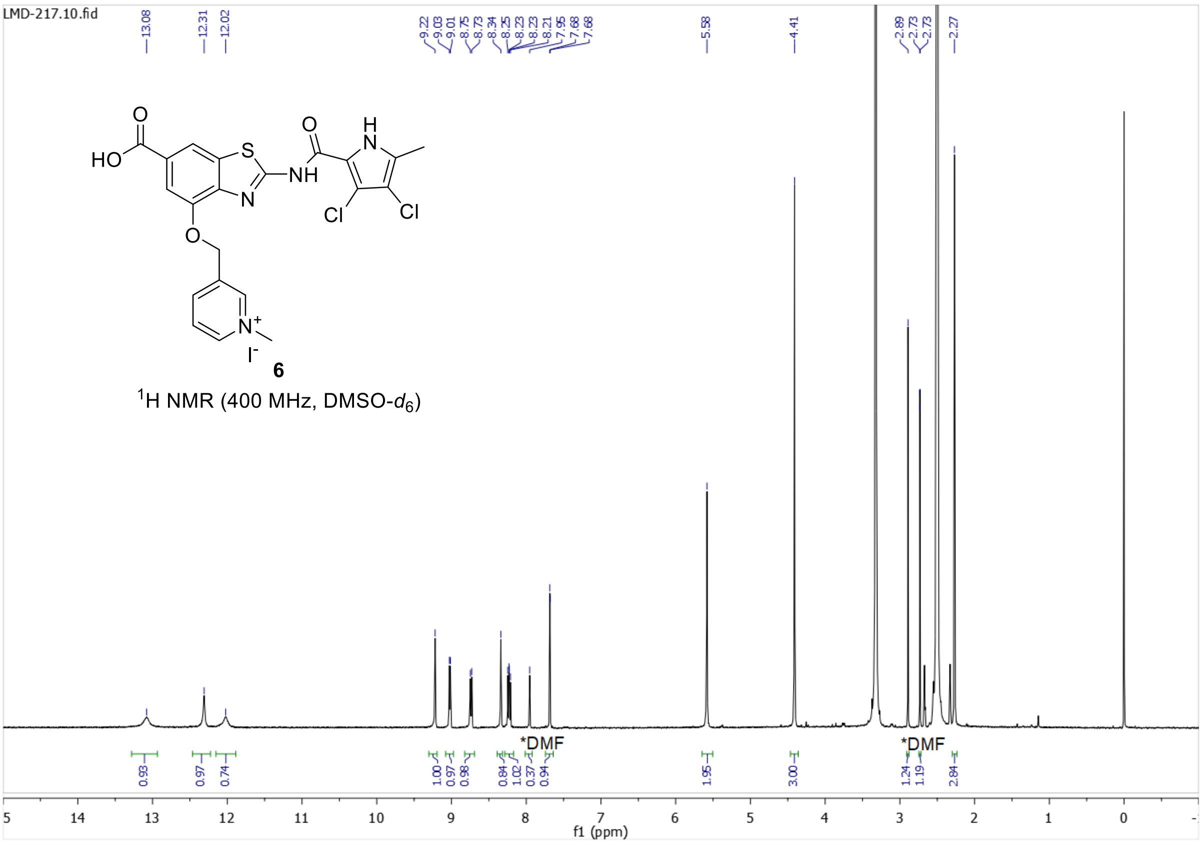
LMD-217.10.fid
13.08
12.31
12.02
9.22
9.03
9.01
8.75
8.73
8.34
8.25
8.23
8.23
8.21
7.95
7.68
7.68
5.58
4.41
2.89
2.73
2.73
2.27
HO
O
S
N
NH
O
H
N
Cl
Cl
O
N+
I-
6
1H NMR (400 MHz, DMSO-d6)
*DMF
*DMF
0.93
0.97
0.74
1.00
0.97
0.98
0.84
1.02
0.37
0.94
1.95
3.00
1.24
1.19
2.84
14
13
12
11
10
9
8
7
6
5
4
3
2
1
0
f1 (ppm)

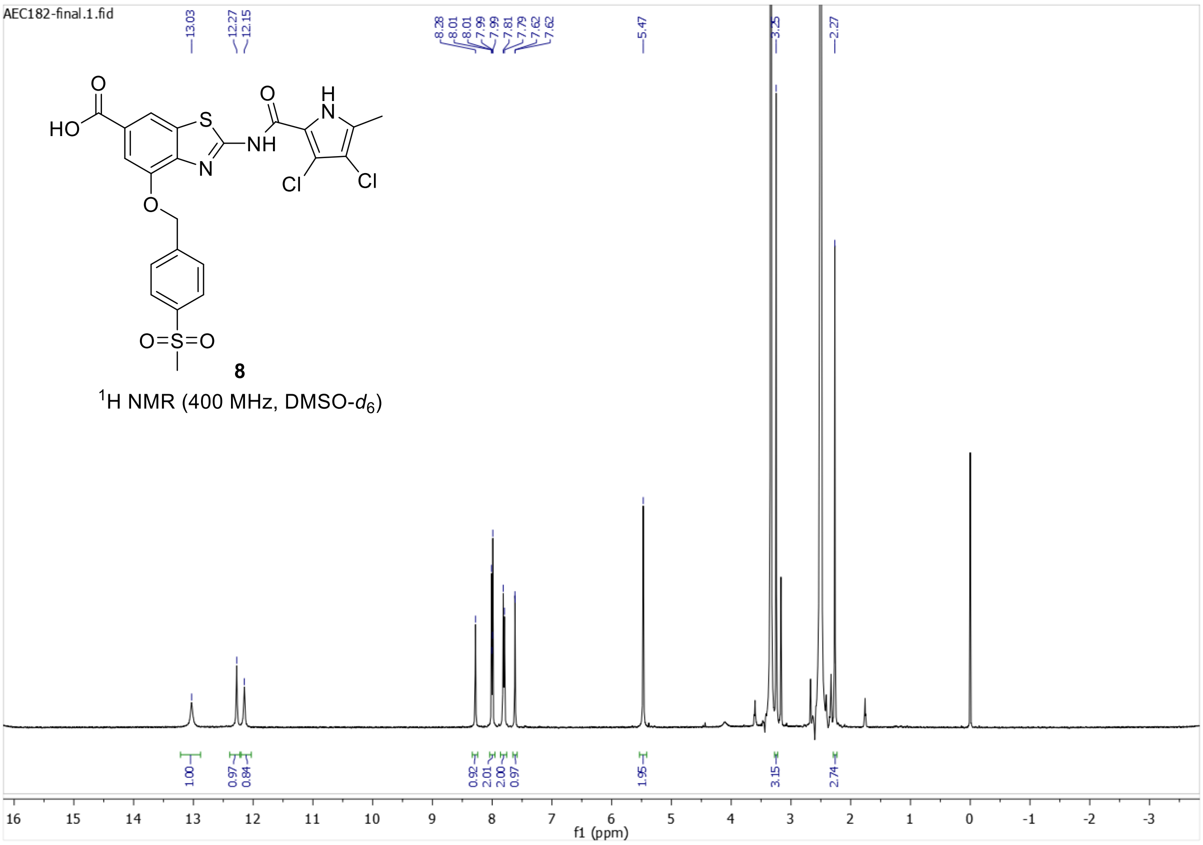
AEC182-final.1.fid
8
^{1}H NMR (400 MHz, DMSO-d_6)
f1 (ppm)

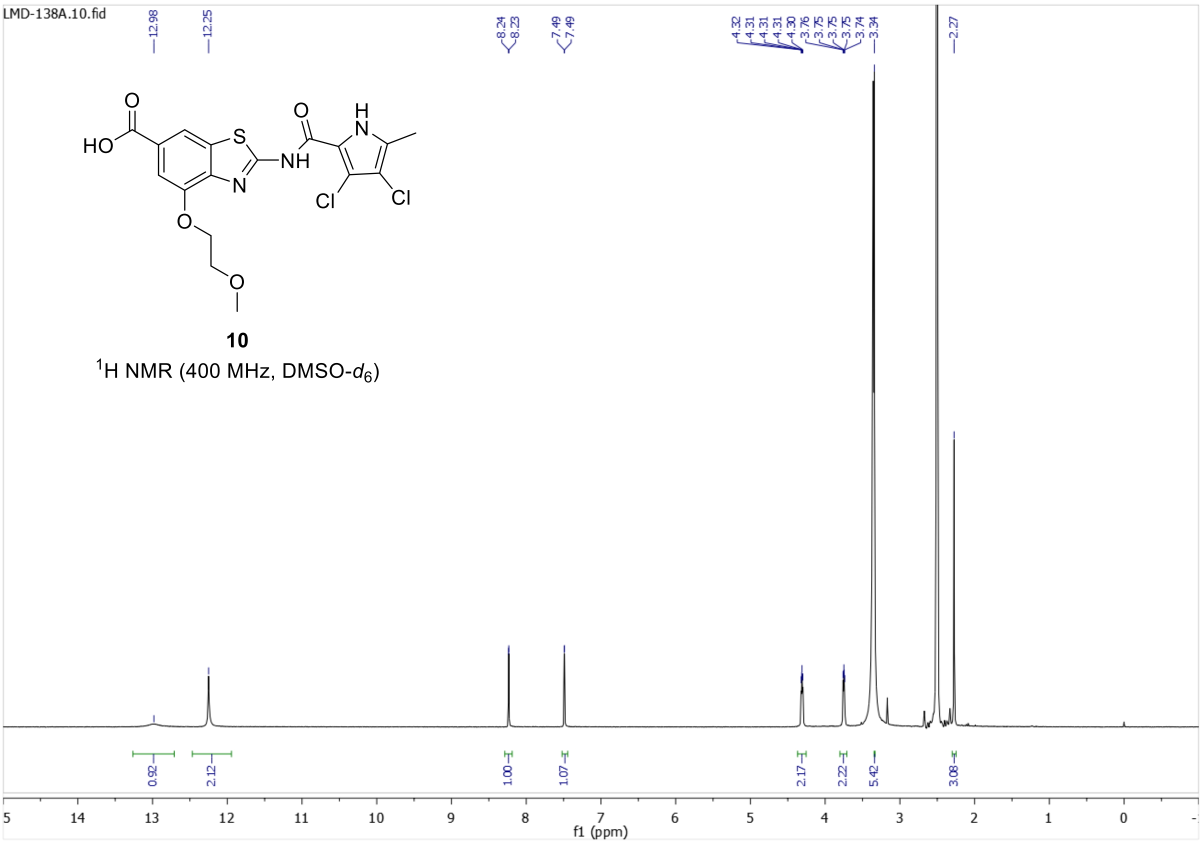
LMD-138A.10.fid
10
^{1}H NMR (400 MHz, DMSO-d_6)
f1 (ppm)

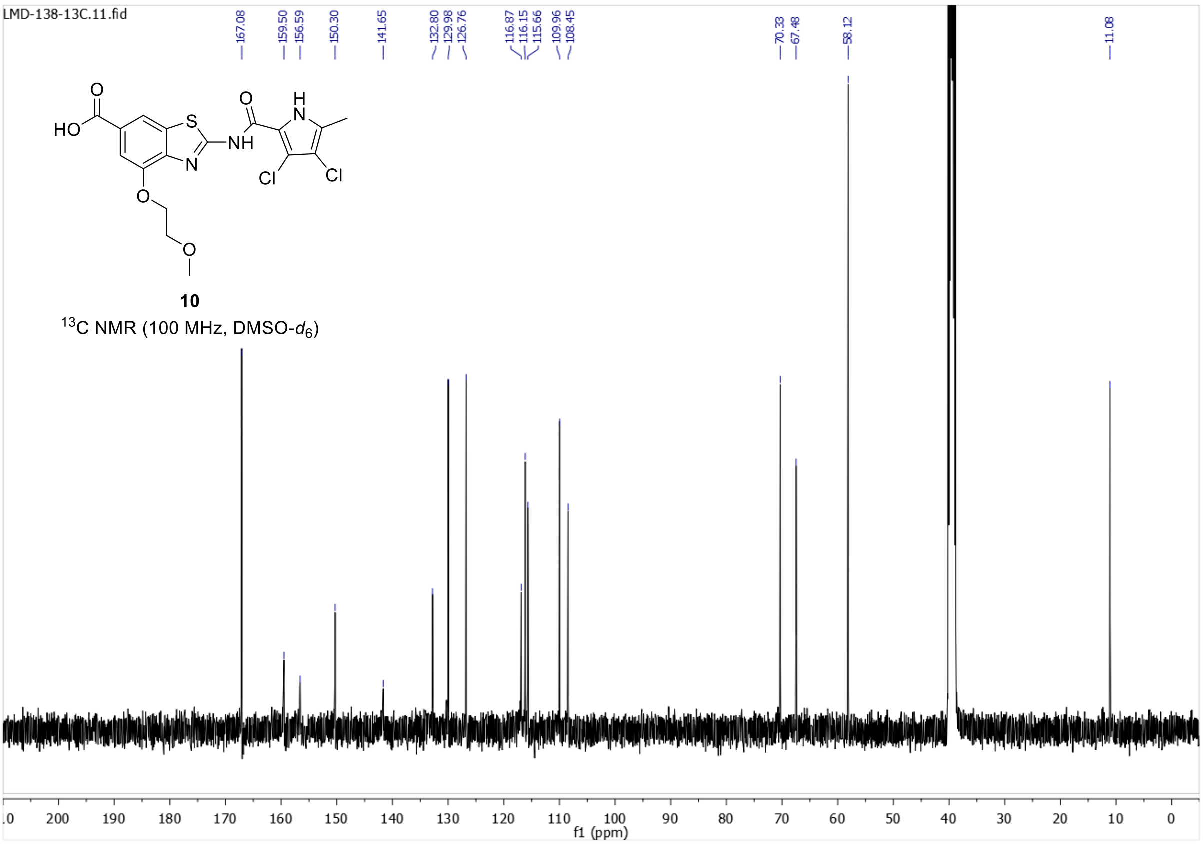

10

^{13}C NMR (100 MHz, DMSO-d_6)

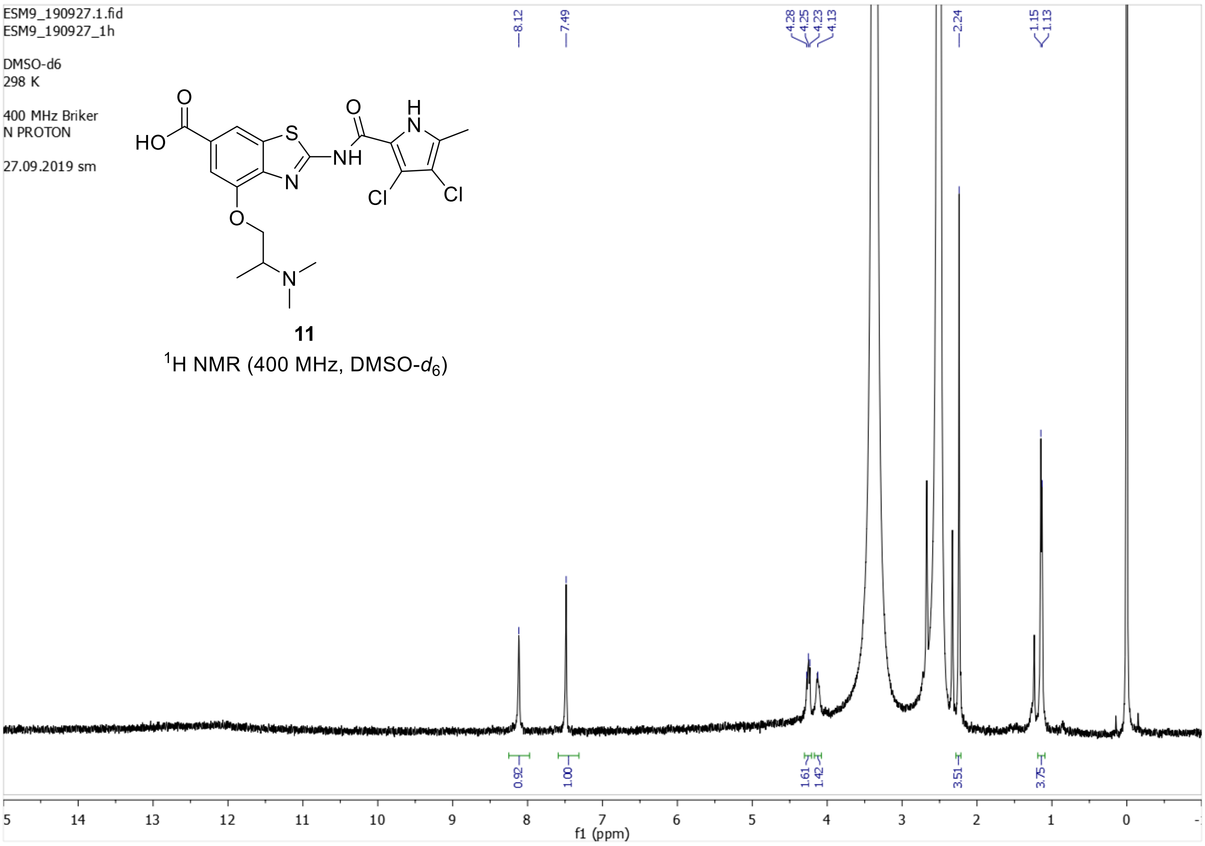

11

^{1}H NMR (400 MHz, DMSO-d_6)

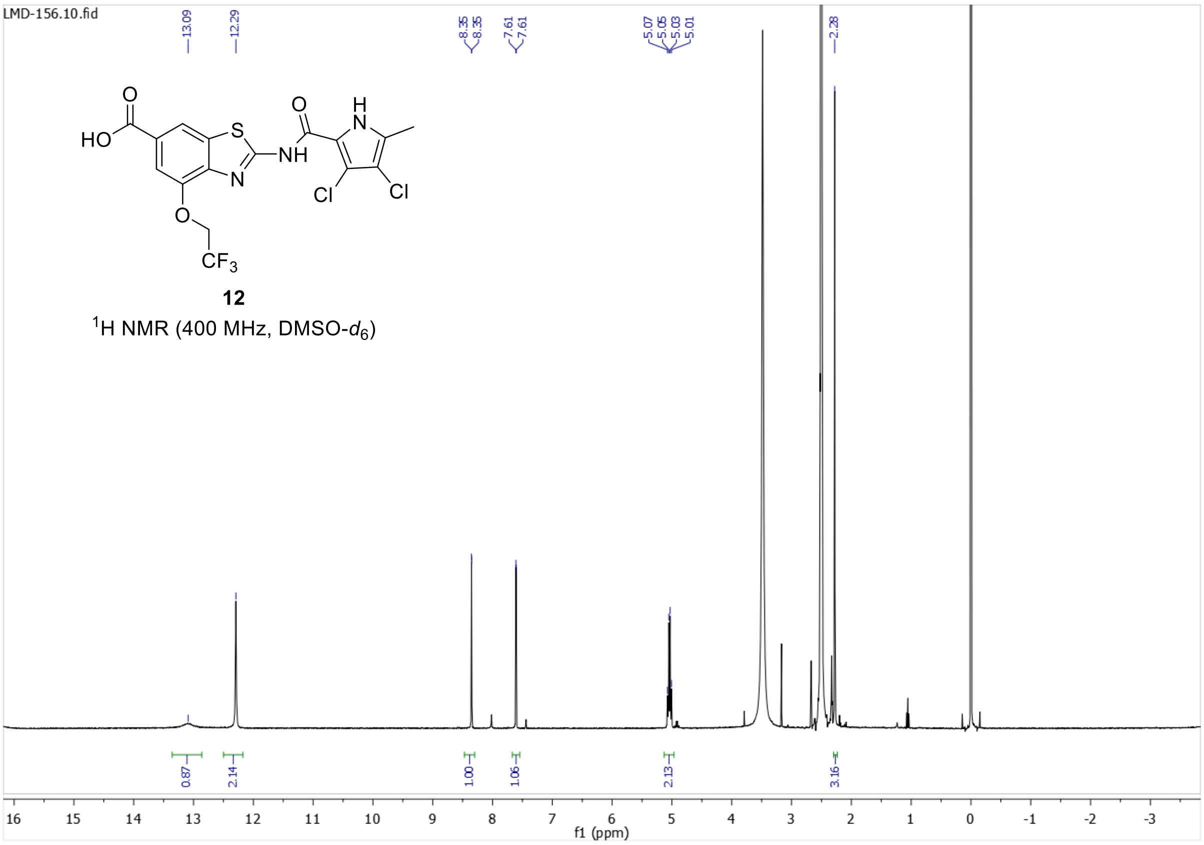
LMD-156.10.fid
13.09
12.29
8.35
8.35
7.61
7.61
5.07
5.05
5.03
5.01
2.28
12
1H NMR (400 MHz, DMSO-d6)
0.87
2.14
1.00
1.06
2.13
3.16
f1 (ppm)

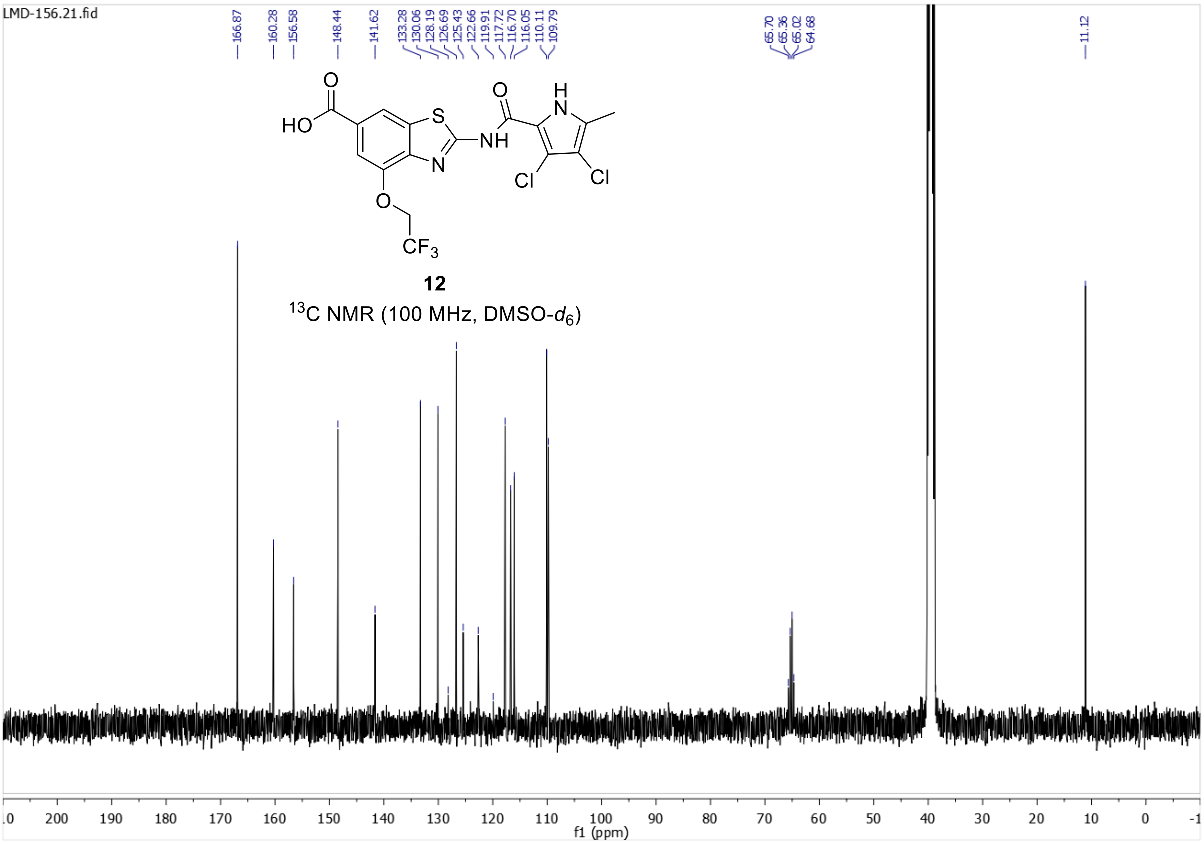
LMD-156.21.fid
166.87
160.28
156.58
148.44
141.62
133.28
130.06
128.19
126.69
125.43
122.66
119.91
117.72
116.70
116.05
110.11
109.79
65.70
65.36
65.02
64.68
11.12
12
13C NMR (100 MHz, DMSO-d6)
f1 (ppm)

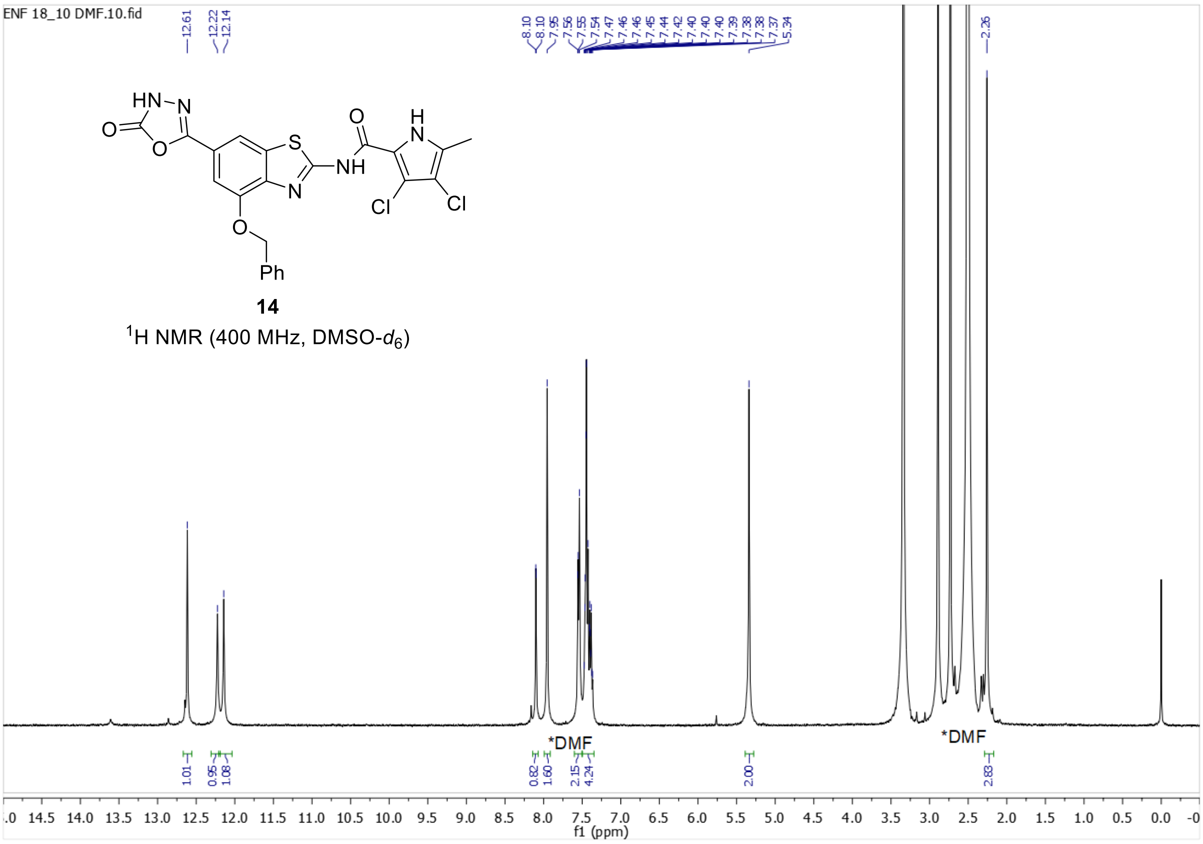
ENF 18_10 DMF.10.fid
14
1H NMR (400 MHz, DMSO-d6)
*DMF
*DMF
f1 (ppm)

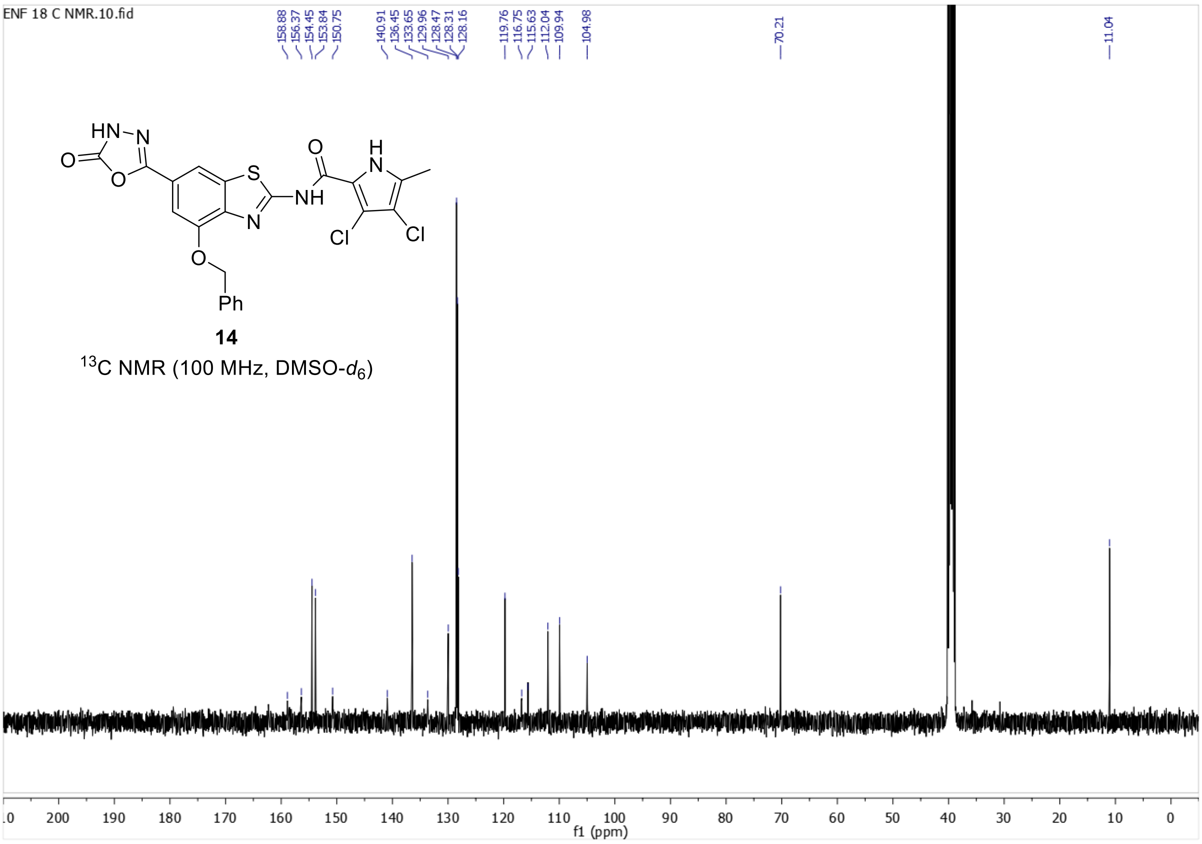
ENF 18 C NMR.10.fid
14
13C NMR (100 MHz, DMSO-d6)
f1 (ppm)

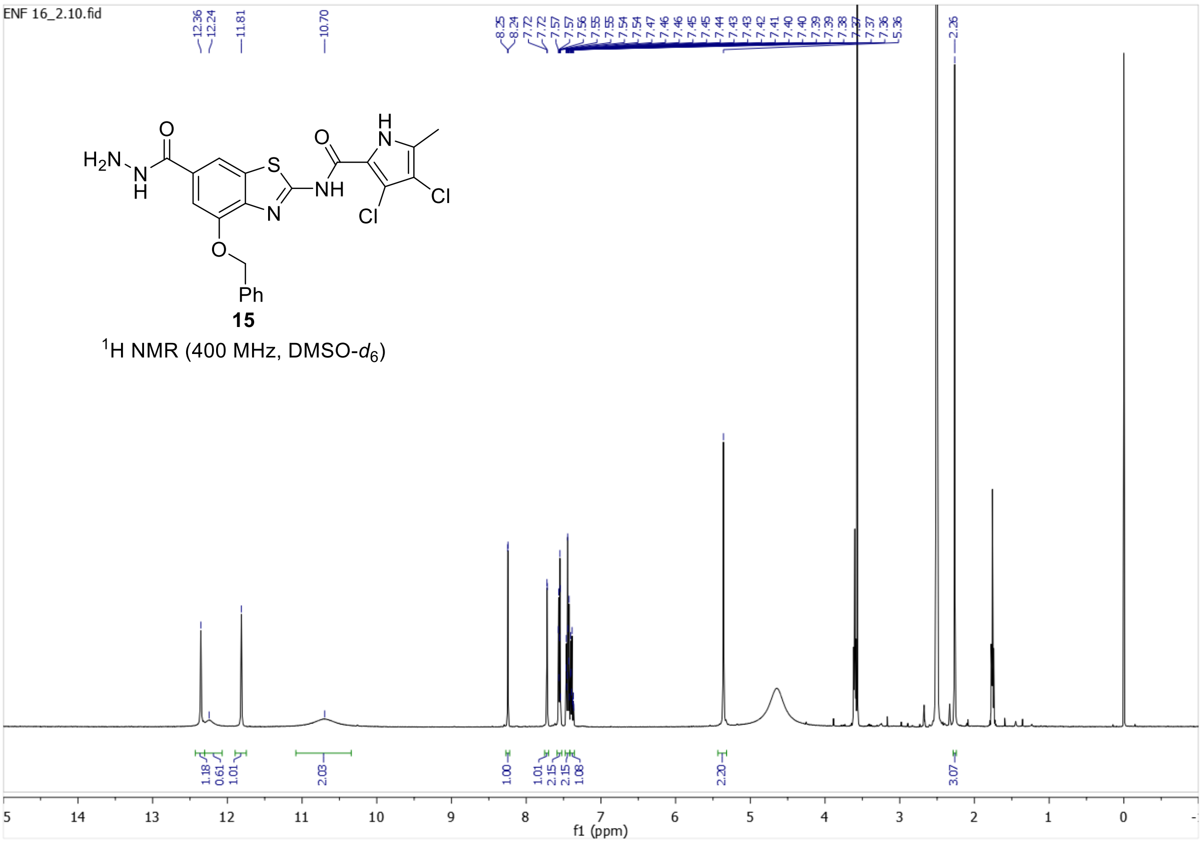
ENF 16_2.10.fid
H2N
N
H
O
S
N
NH
O
H
N
Cl
Cl
O
Ph
15
1H NMR (400 MHz, DMSO-d6)
f1 (ppm)

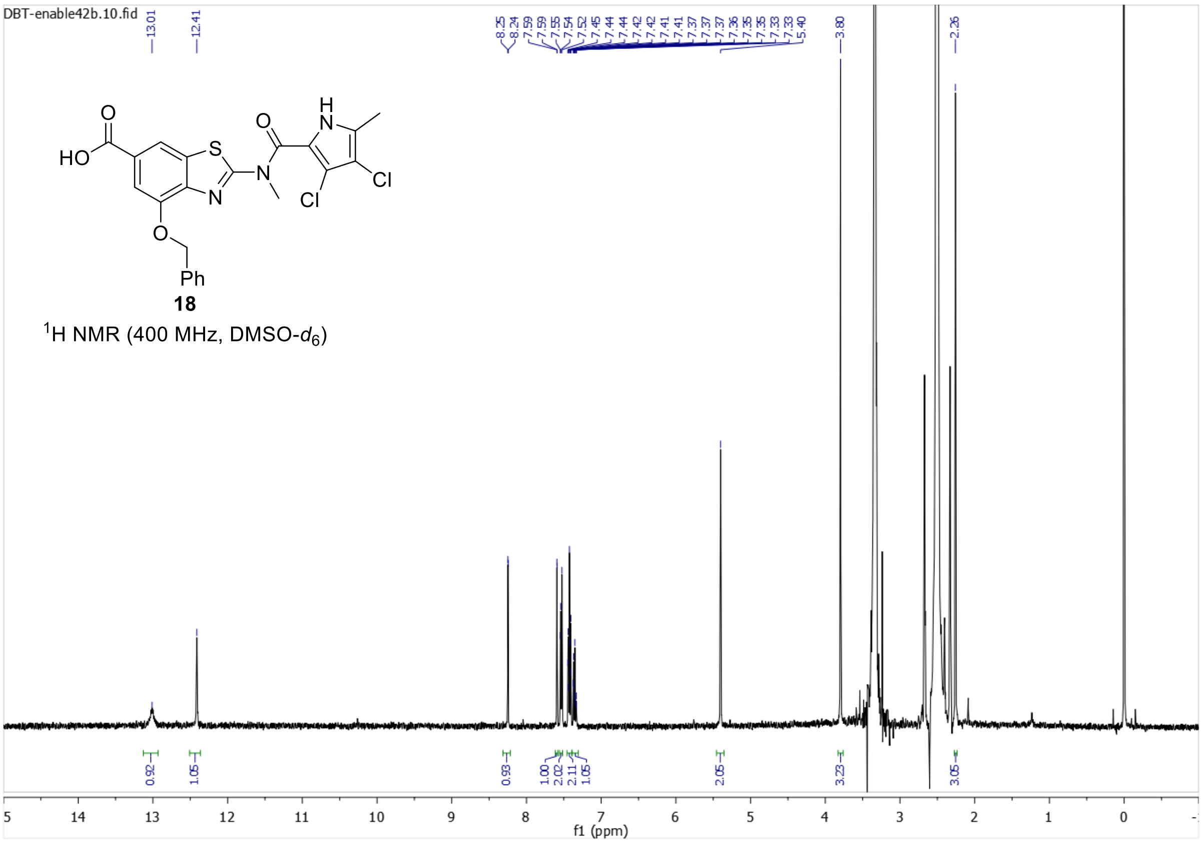
DBT-enable42b.10.fid
HO
O
S
N
N
O
H
N
Cl
Cl
O
Ph
18
1H NMR (400 MHz, DMSO-d6)
f1 (ppm)

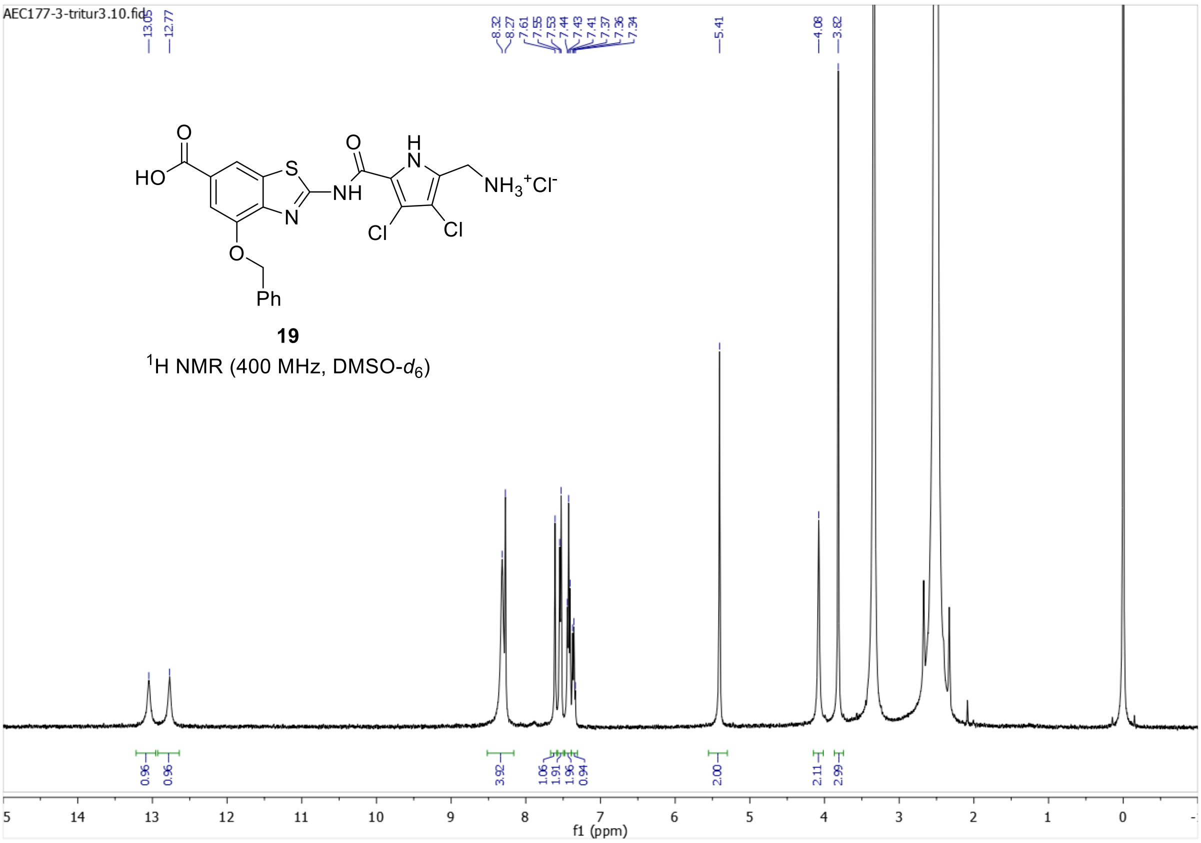
AEC177-3-tritur3.10.fid
19
1H NMR (400 MHz, DMSO-d6)
f1 (ppm)

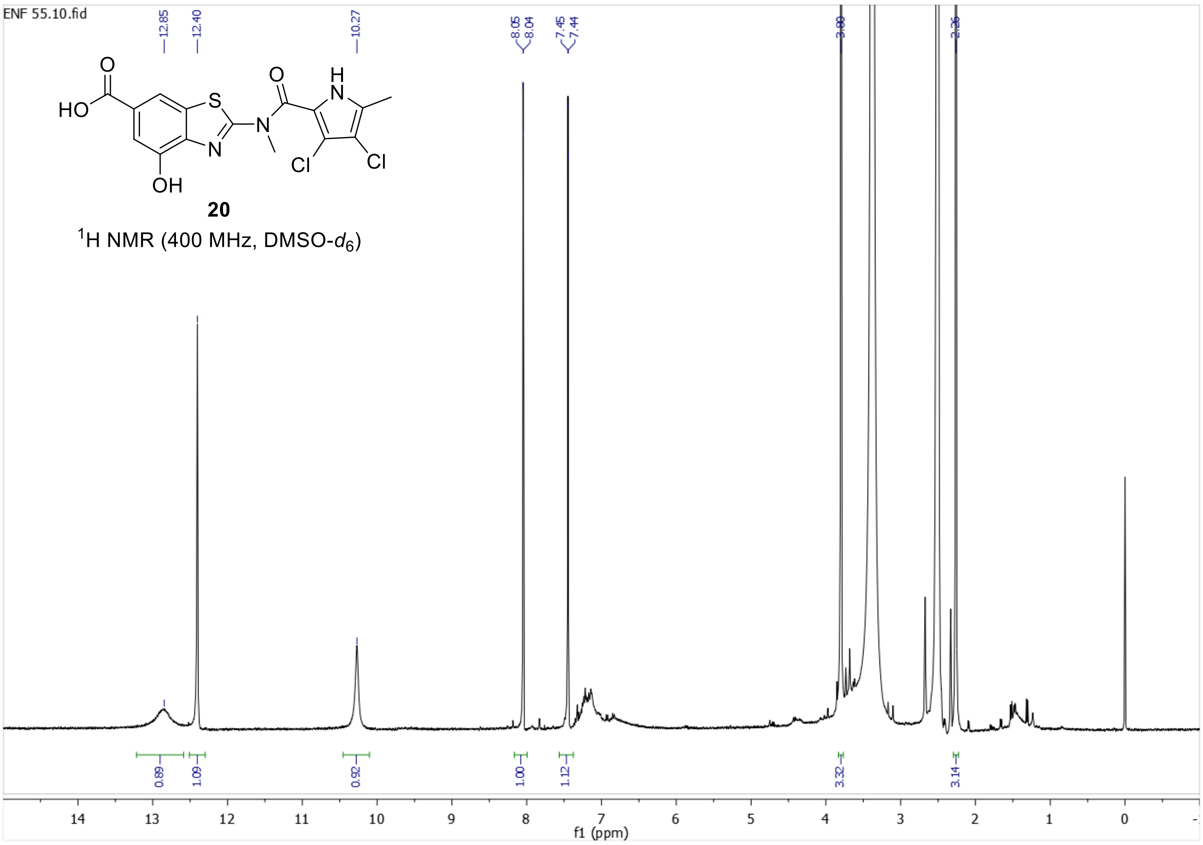
ENF 55.10.fid
20
1H NMR (400 MHz, DMSO-d6)
f1 (ppm)

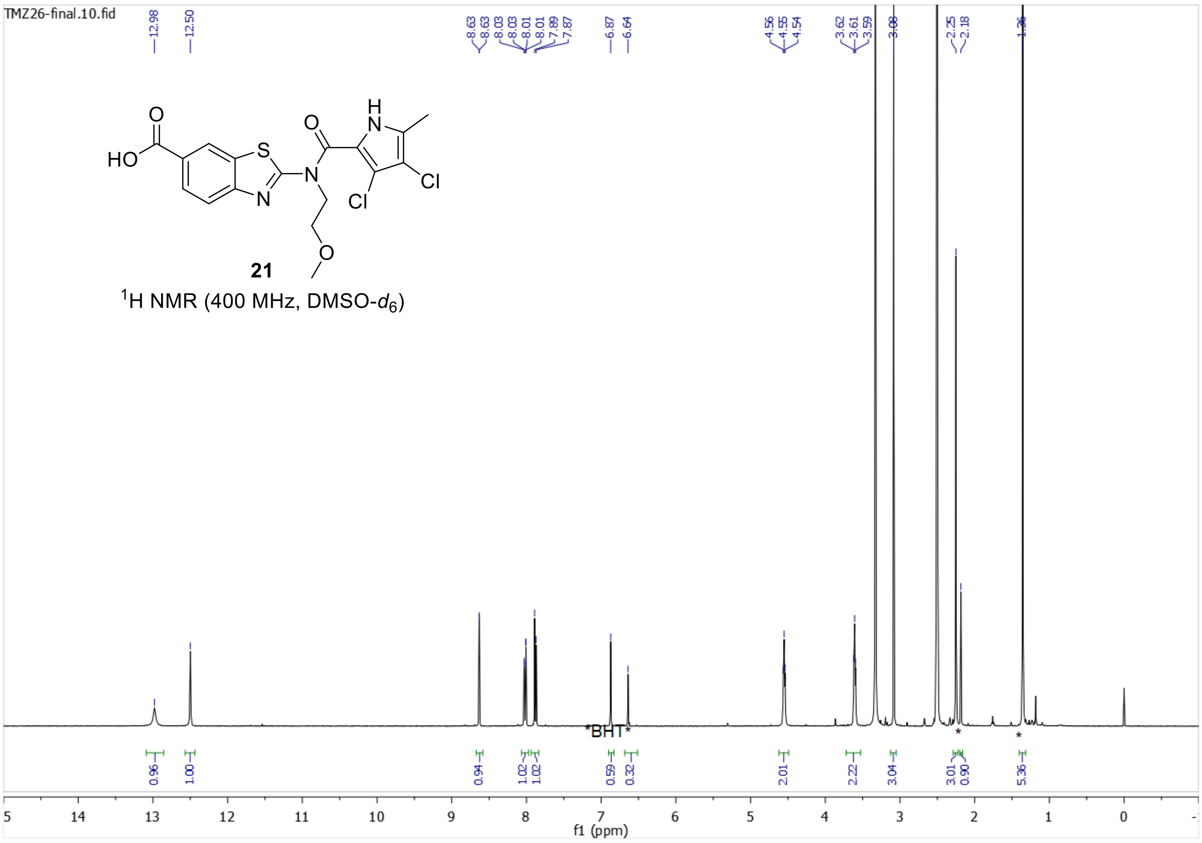
TMZ26-final.10.fid
HO
O
S
N
N
O
H
N
Cl
Cl
O
21
1H NMR (400 MHz, DMSO-d6)
BHT
f1 (ppm)

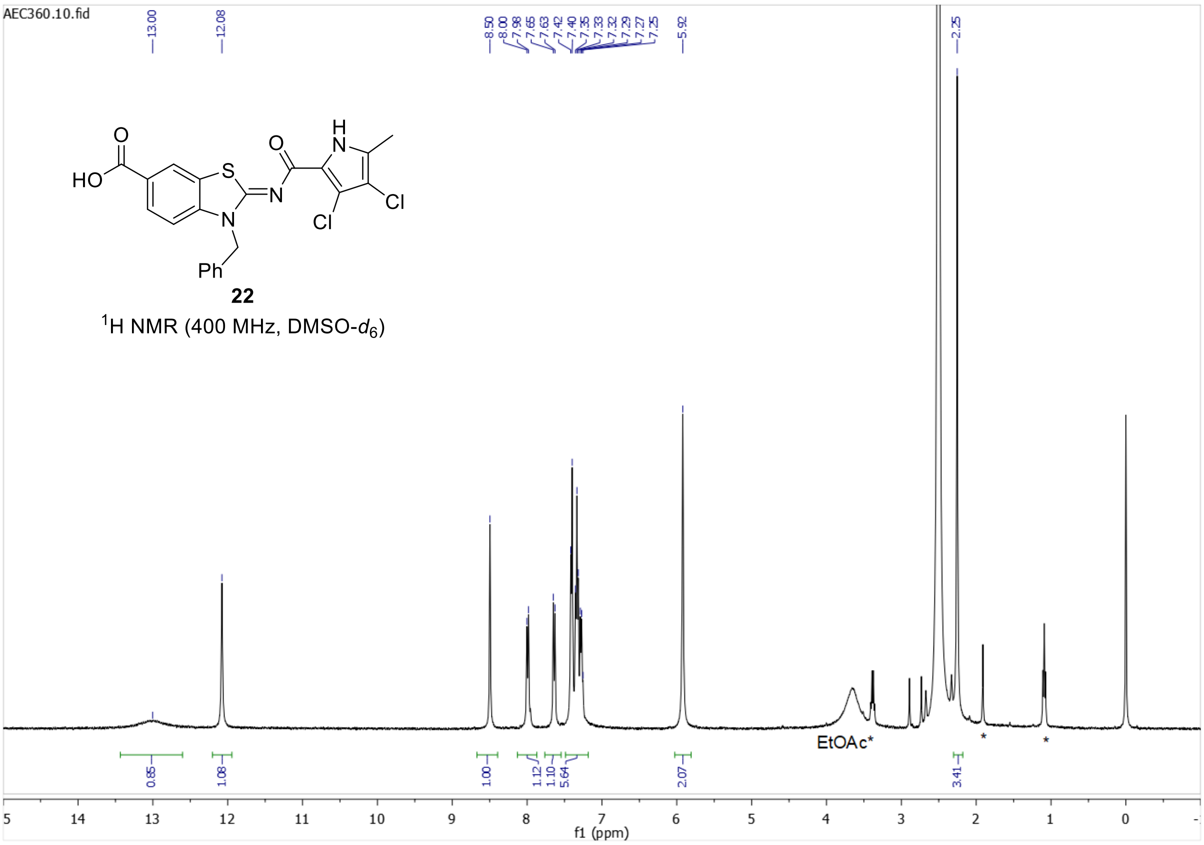
AEC360.10.fid
HO
O
S
N
N
O
H
N
Cl
Cl
Ph
22
1H NMR (400 MHz, DMSO-d6)
EtOAc*
f1 (ppm)

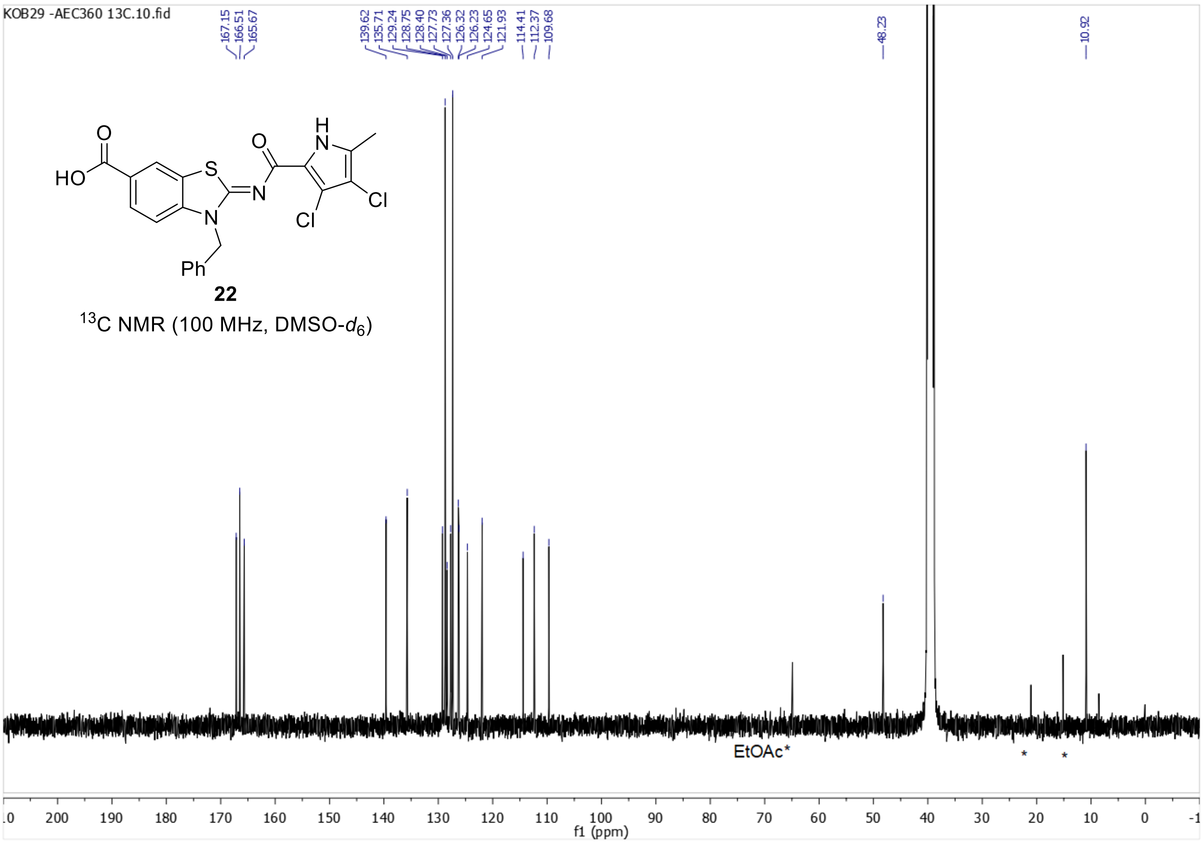
KOB29 -AEC360 13C.10.fid
167.15
166.51
165.67
139.62
135.71
129.24
128.75
128.40
127.73
127.36
126.32
126.23
124.65
121.93
114.41
112.37
109.68
48.23
10.92
HO
O
S
N
N
O
H
N
Cl
Cl
Ph
22
13C NMR (100 MHz, DMSO-d6)
EtOAc*
f1 (ppm)

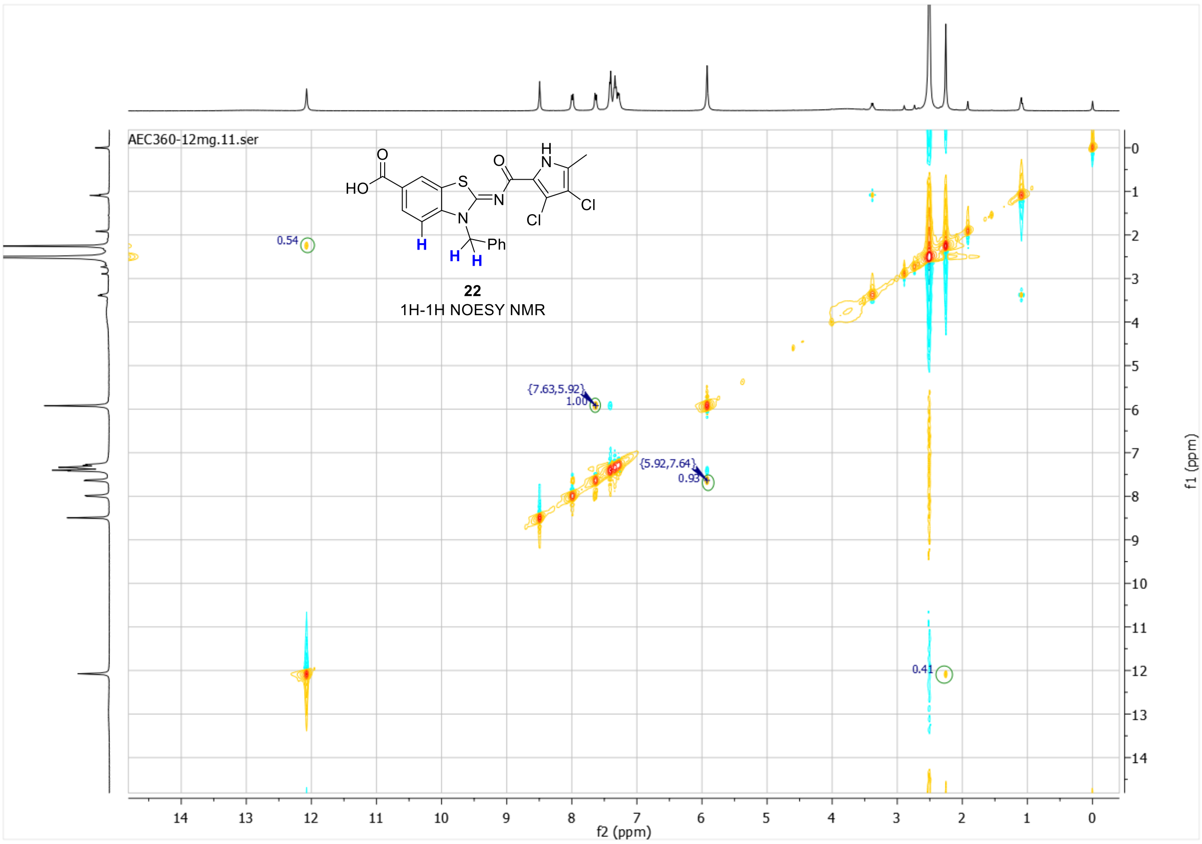
AEC360-12mg.11.ser
22
1H-1H NOESY NMR
0.54
{7.63,5.92}
1.00
{5.92,7.64}
0.93
0.41
f2 (ppm)
f1 (ppm)

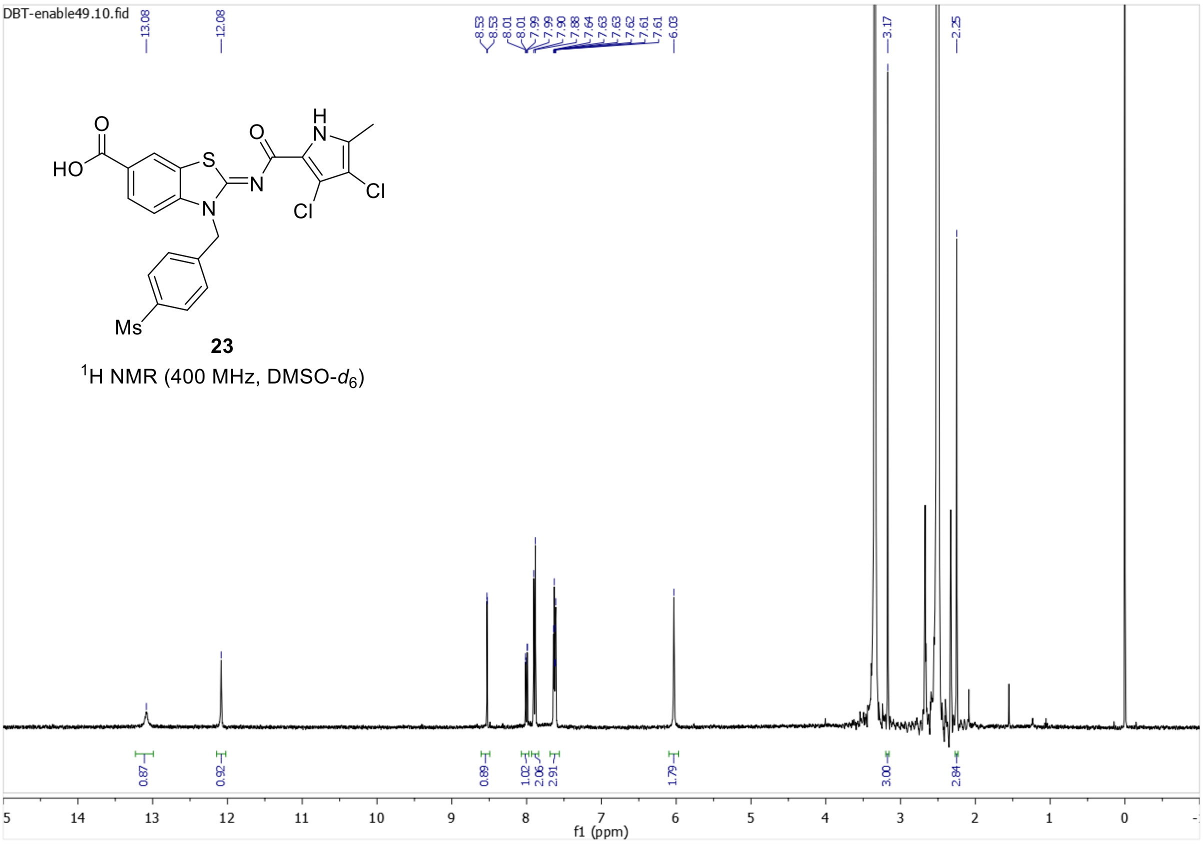
DBT-enable49.10.fid
23
^{1}H NMR (400 MHz, DMSO-d_6)
f1 (ppm)

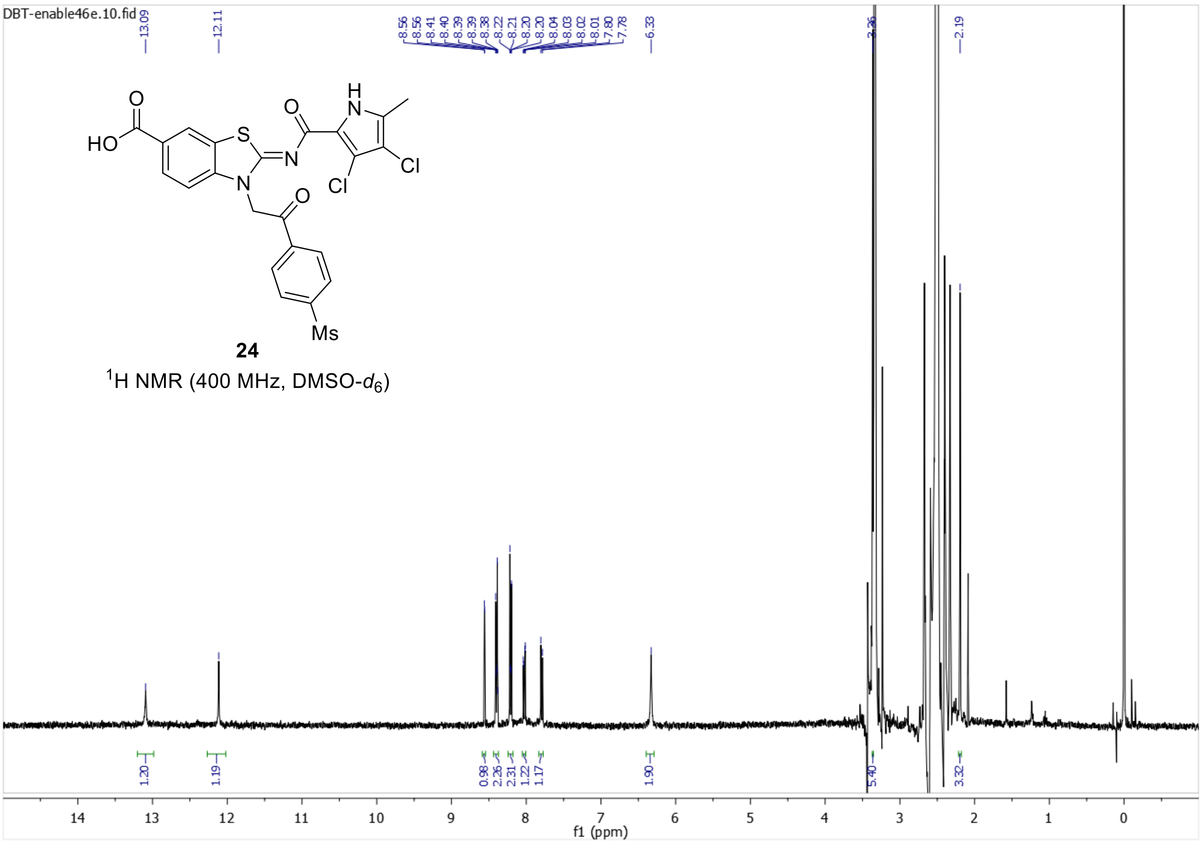
DBT-enable46e.10.fid
24
^{1}H NMR (400 MHz, DMSO-d_6)
f1 (ppm)

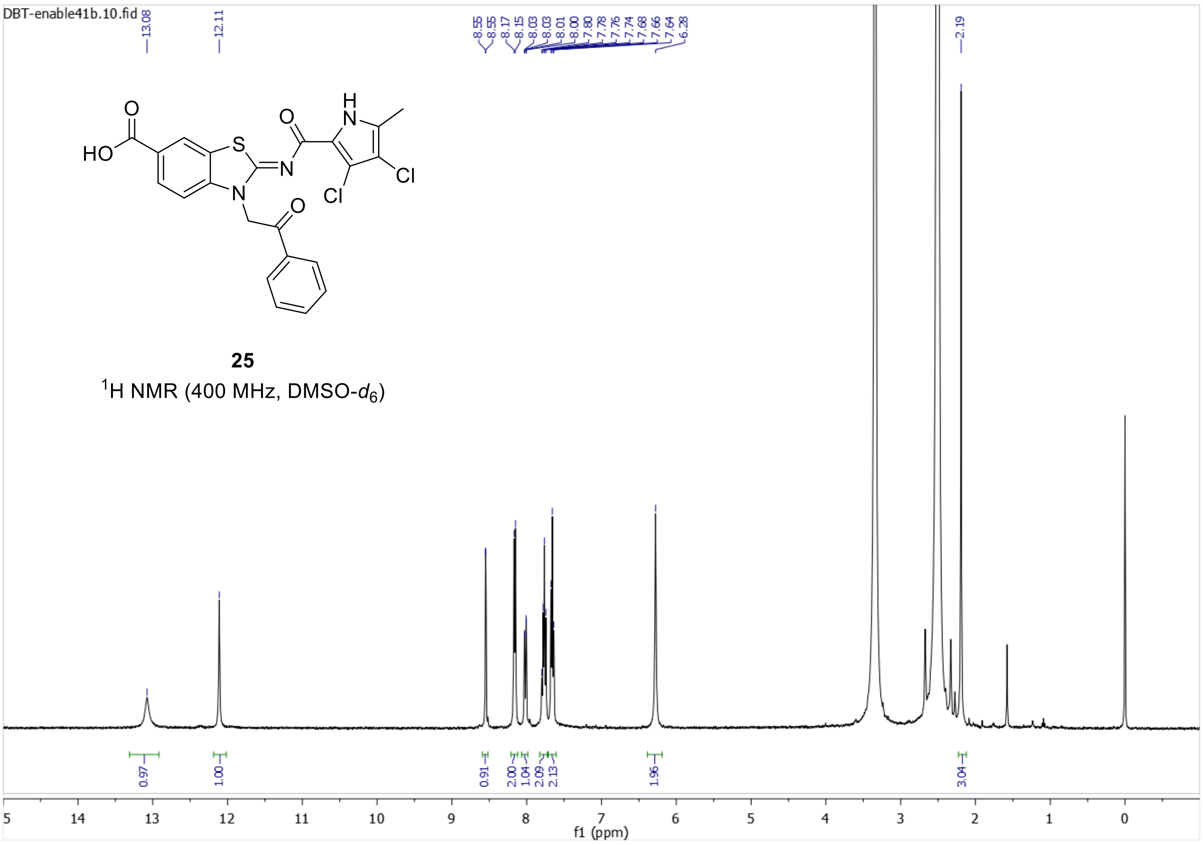

25

^{1}H NMR (400 MHz, DMSO-d_6)

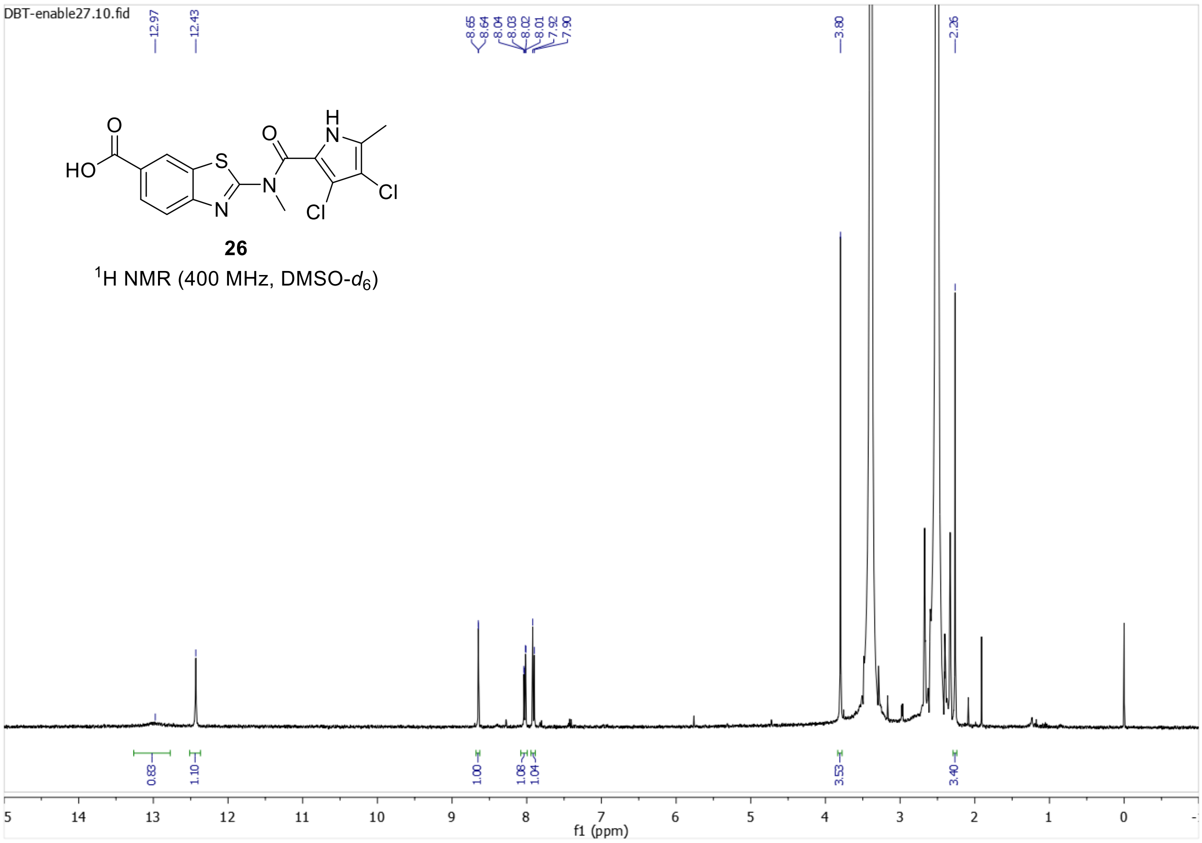

26

^{1}H NMR (400 MHz, DMSO-d_6)

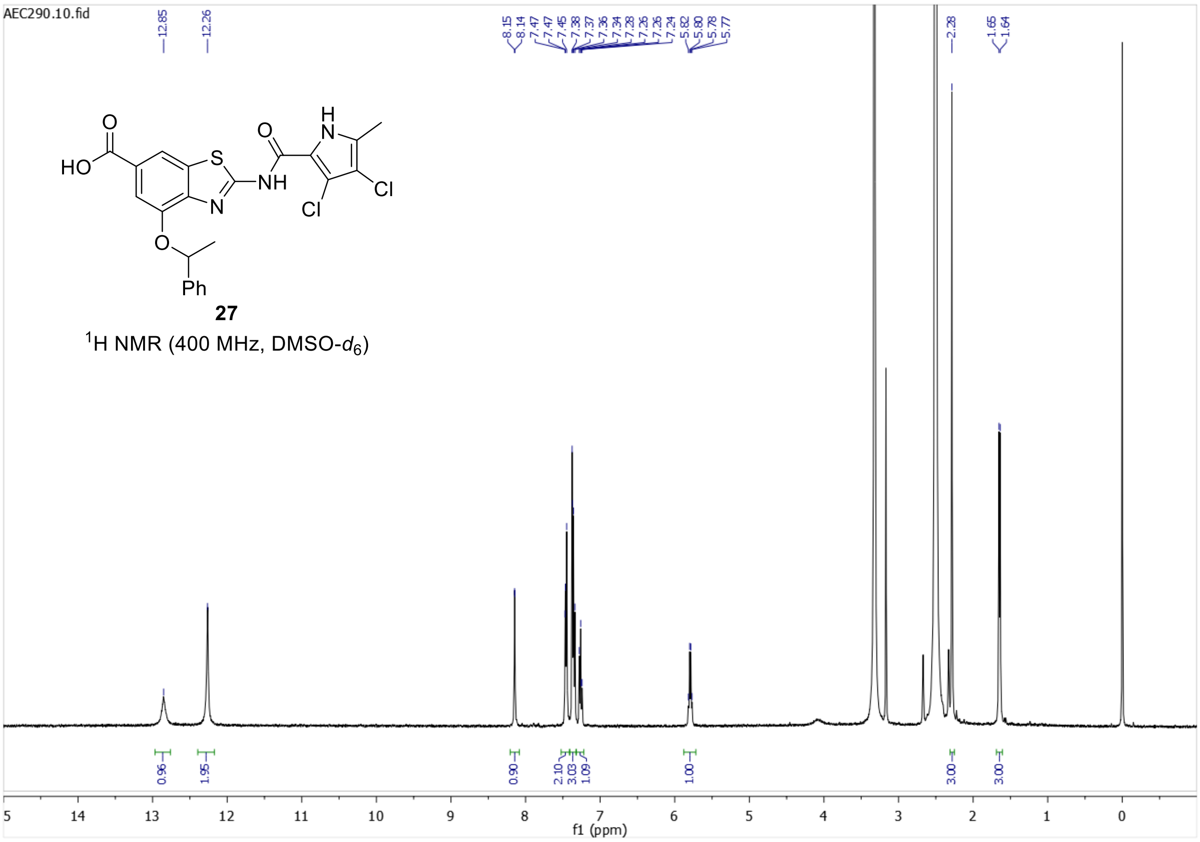
AEC290.10.fid
27
1H NMR (400 MHz, DMSO-d6)
f1 (ppm)

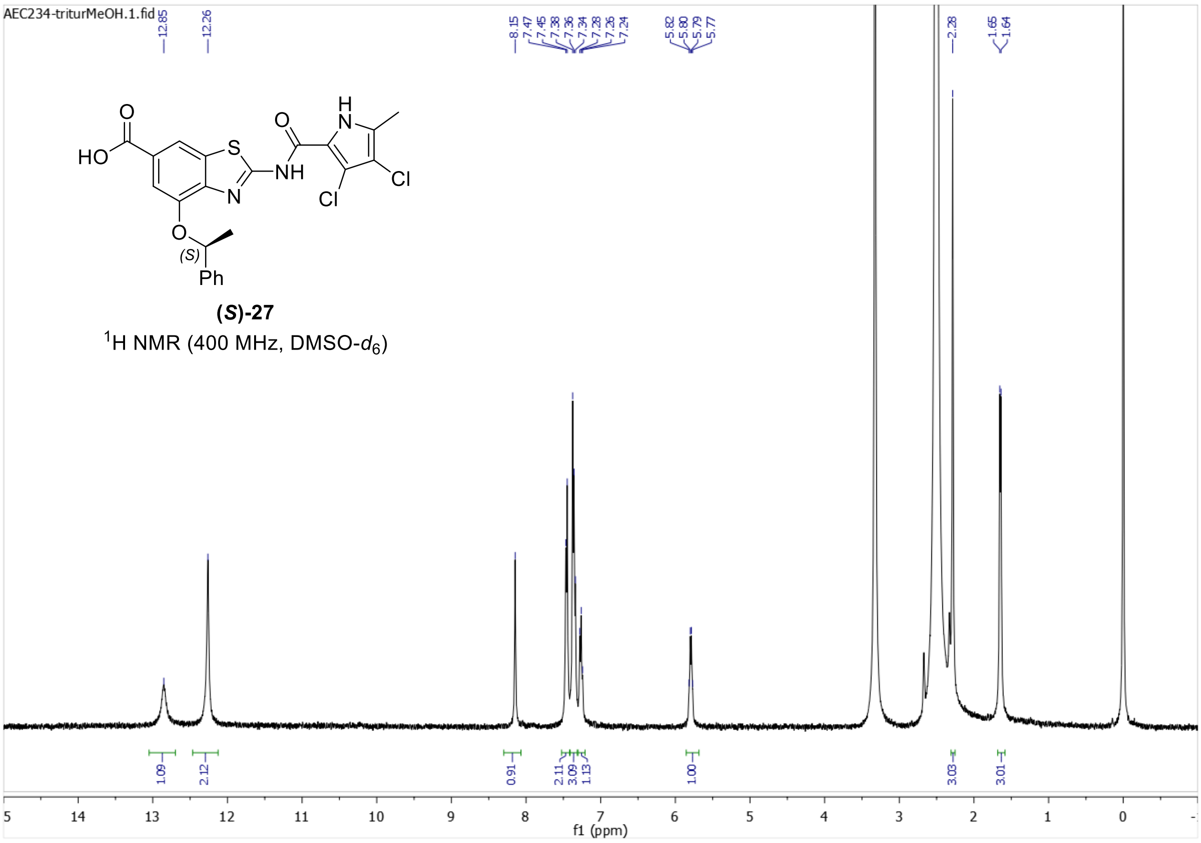
AEC234-triturMeOH.1.fid
(S)-27
1H NMR (400 MHz, DMSO-d6)
f1 (ppm)

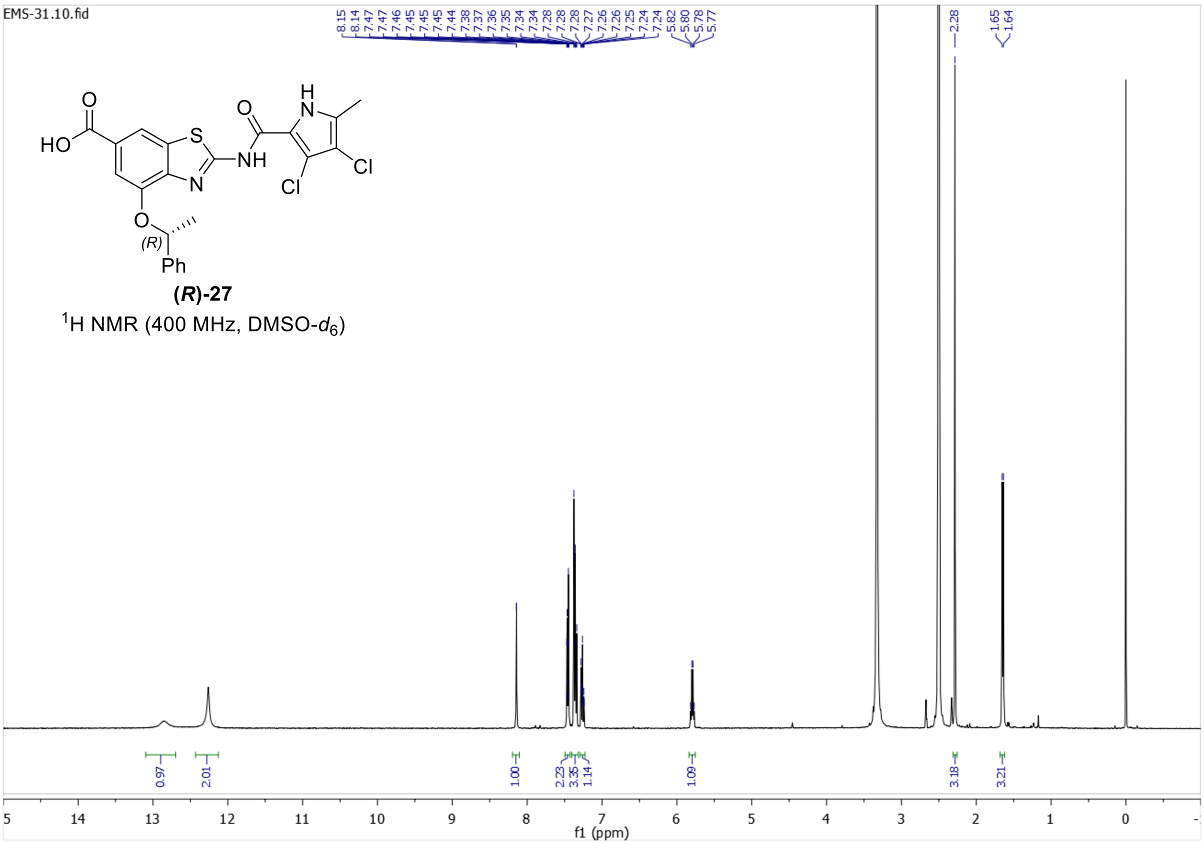
EMS-31.10.fid
8.15 8.14 7.47 7.46 7.45 7.45 7.45 7.44 7.38 7.37 7.36 7.35 7.34 7.34 7.28 7.28 7.28 7.27 7.26 7.25 7.24 5.82 5.80 5.78 5.77
2.28
1.65 1.64
HO
O
S
N
NH
O
H
N
Cl
Cl
O
(R)
Ph
(R)-27
^{1}H NMR (400 MHz, DMSO-d_6)
0.97 2.01 1.00 2.23 3.35 1.14 1.09 3.18 3.21
f1 (ppm)

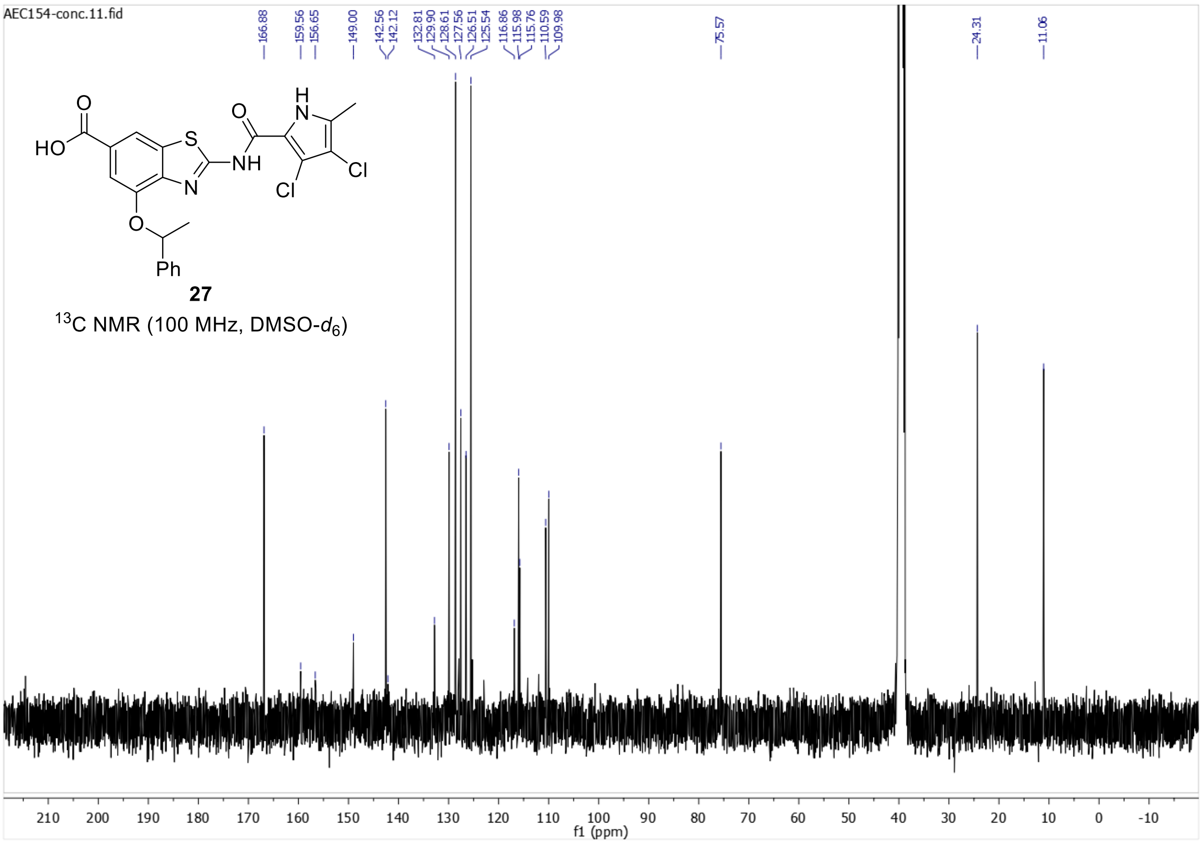
AEC154-conc.11.fid
166.88 159.56 156.65 149.00 142.56 142.12 132.81 129.90 128.61 127.56 126.51 125.54 116.86 115.98 115.76 110.59 109.98
75.57
24.31
11.06
HO
O
S
N
NH
O
H
N
Cl
Cl
O
Ph
27
^{13}C NMR (100 MHz, DMSO-d_6)
f1 (ppm)

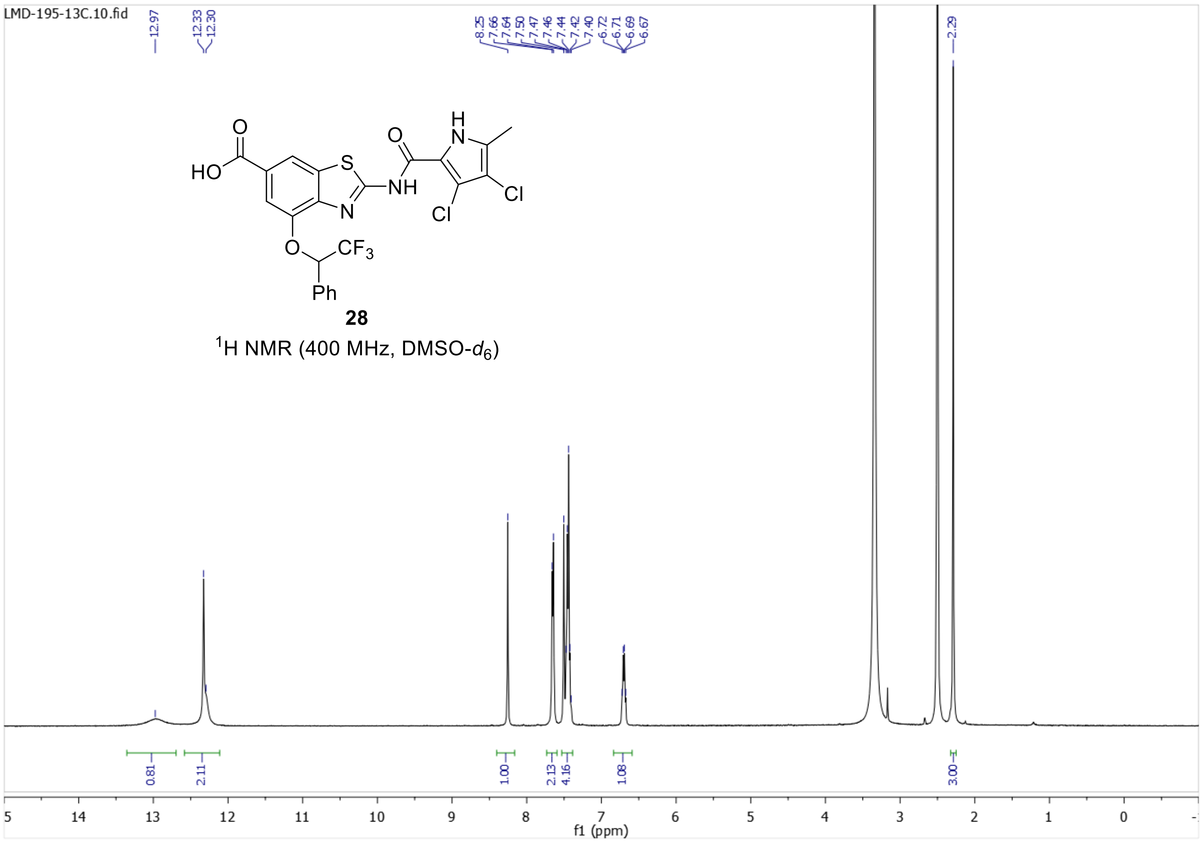

28

^{1}H NMR (400 MHz, DMSO-d_6)

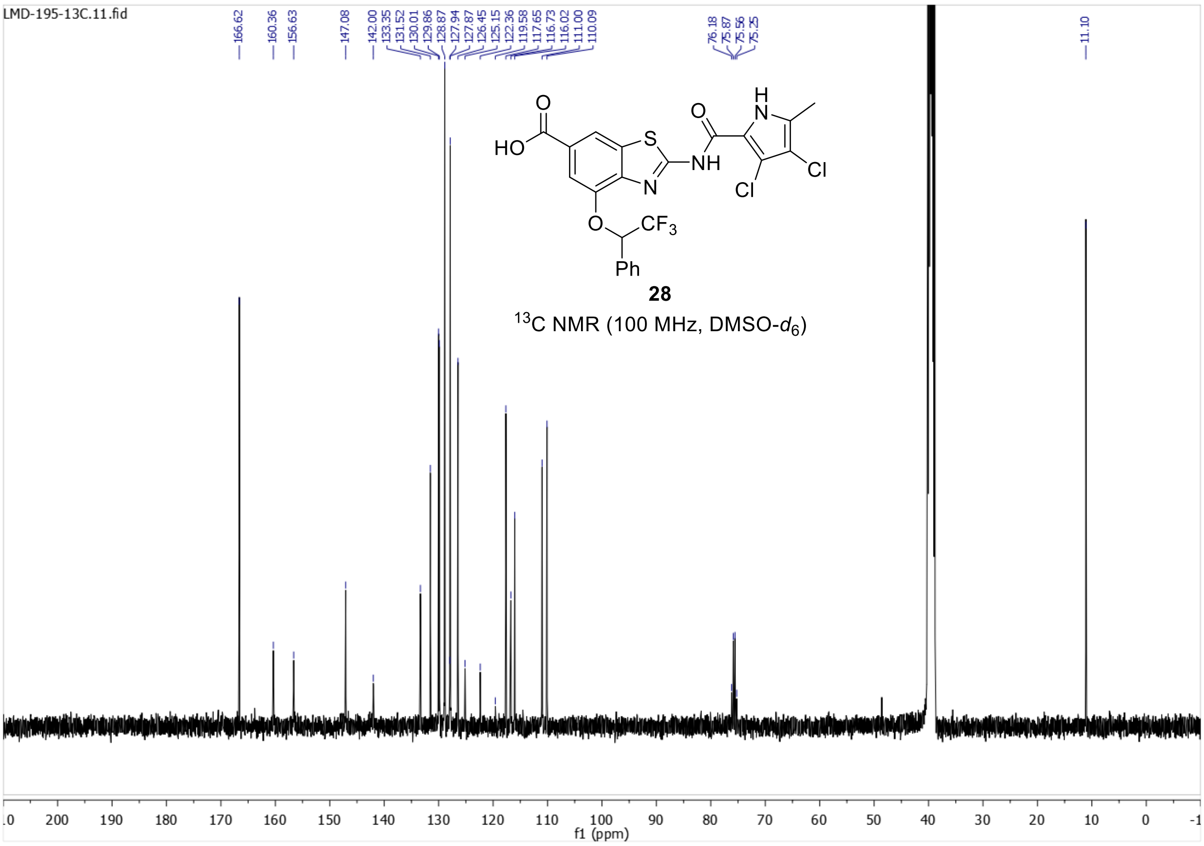

28

^{13}C NMR (100 MHz, DMSO-d_6)

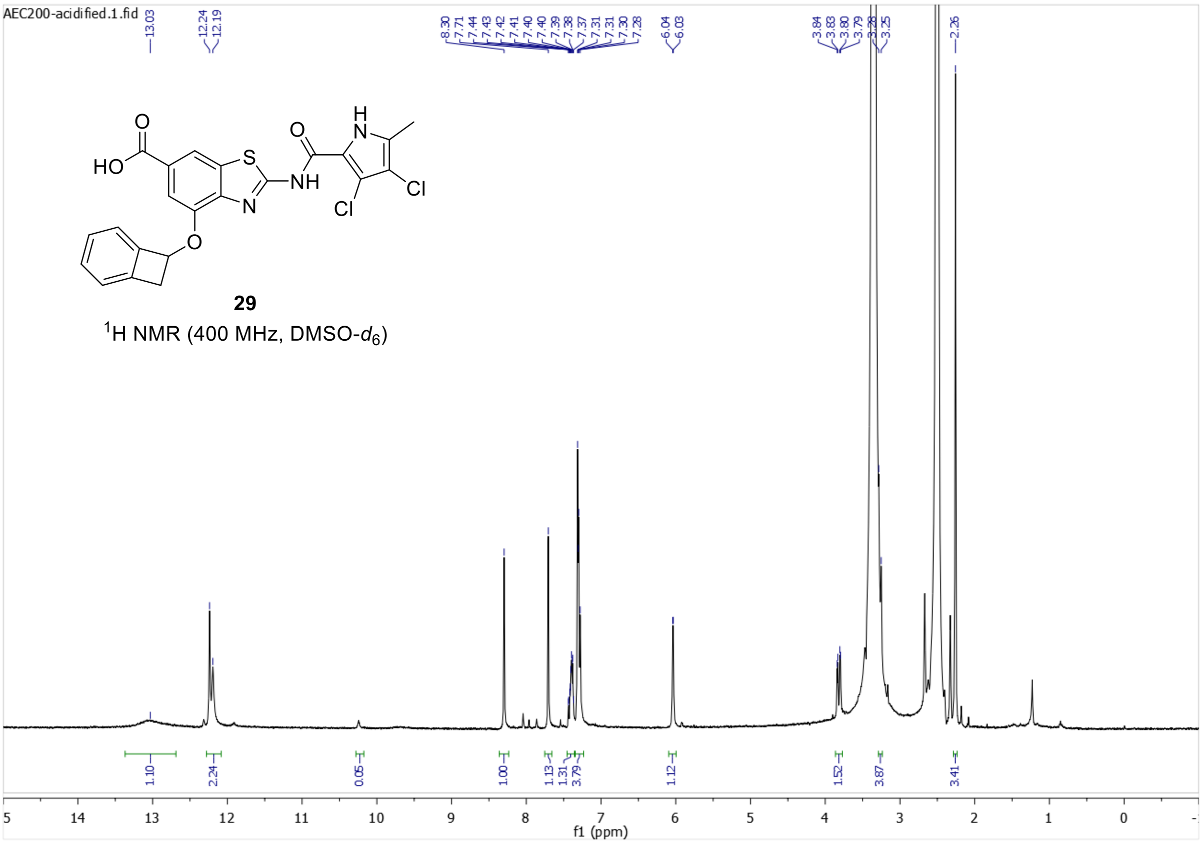
AEC200-acidified.1.fid
HO
O
S
N
NH
O
H
N
Cl
Cl
O
29
¹H NMR (400 MHz, DMSO-d6)
f1 (ppm)

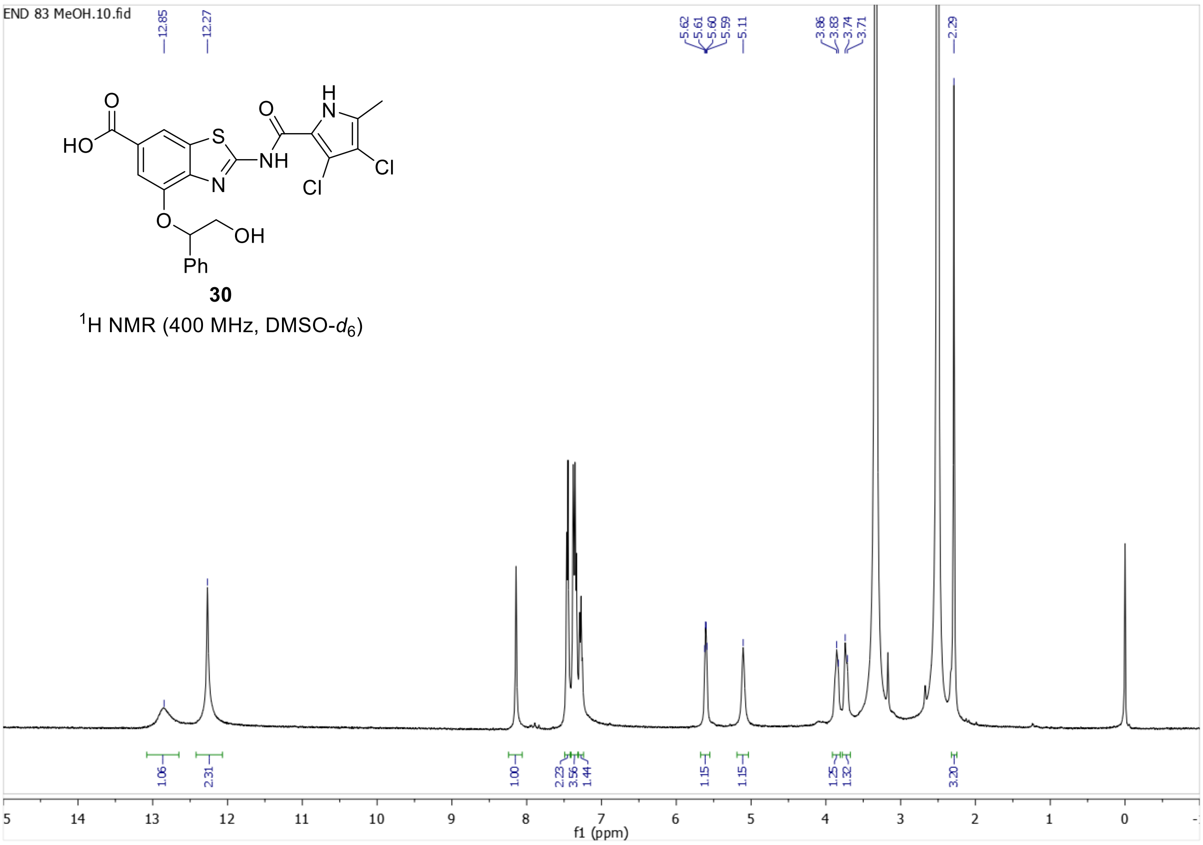
END 83 MeOH.10.fid
HO
O
S
N
NH
O
H
N
Cl
Cl
O
OH
Ph
30
¹H NMR (400 MHz, DMSO-d6)
f1 (ppm)

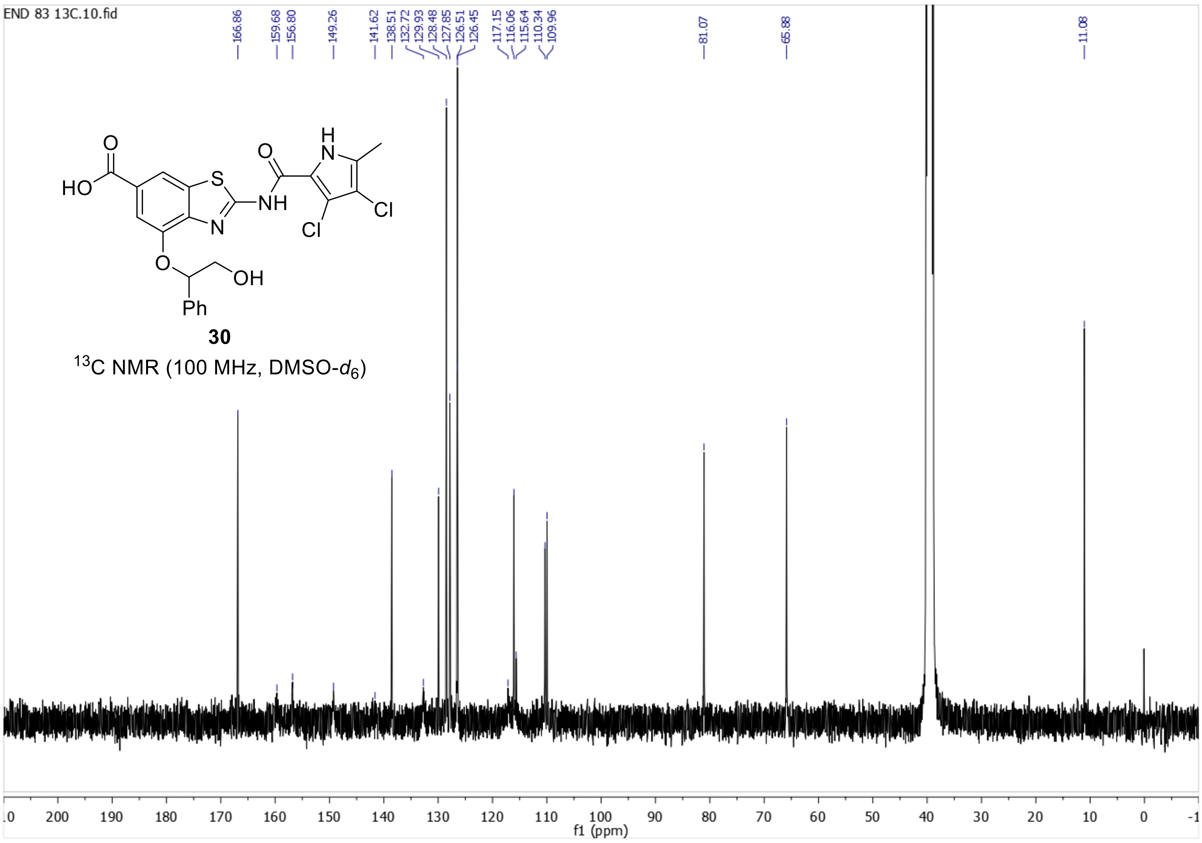

END 83 13C.10.fid
166.86
159.68
156.80
149.26
141.62
138.51
132.72
129.93
128.48
127.85
126.51
126.45
117.15
116.06
115.64
110.34
109.96
81.07
65.88
11.08
HO
O
S
N
NH
H
N
Cl
Cl
OH
Ph
30
13C NMR (100 MHz, DMSO-d6)
f1 (ppm)

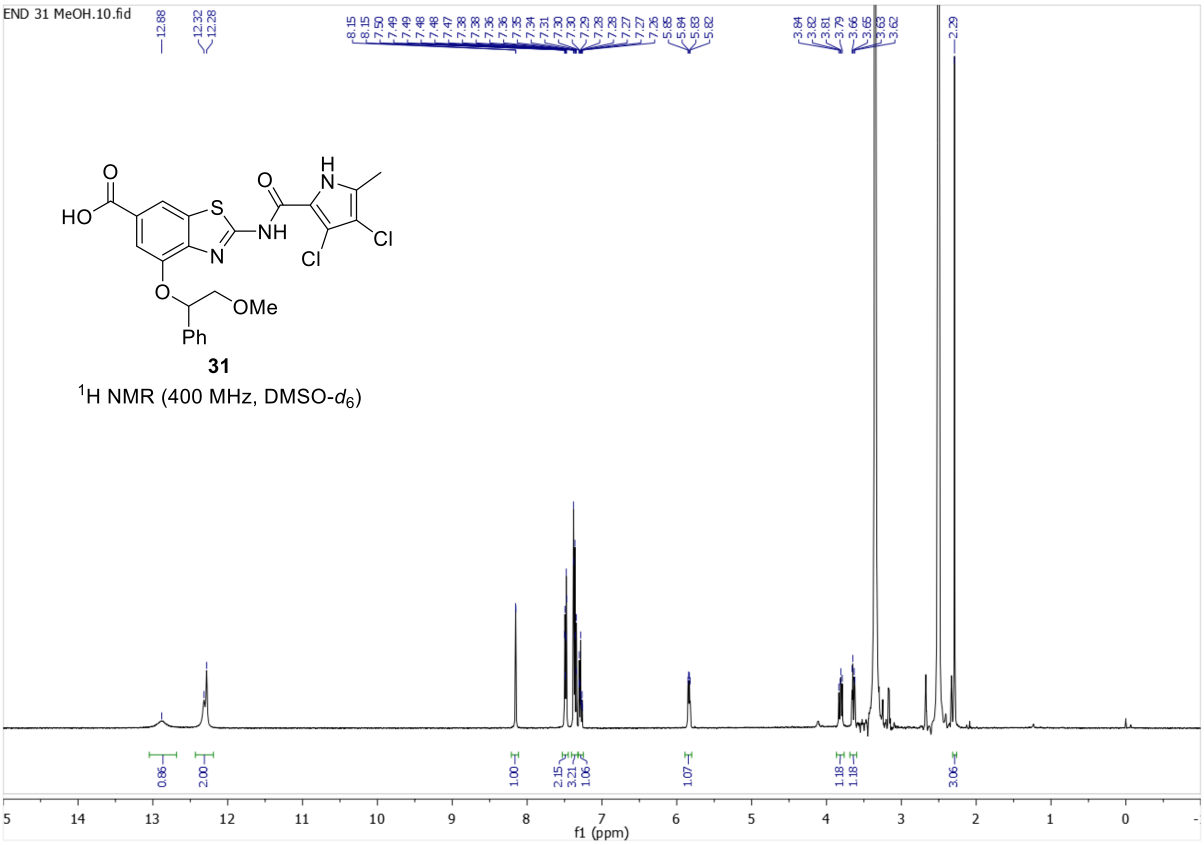

END 31 MeOH.10.fid
12.88
12.32
12.28
8.15
8.15
7.50
7.49
7.49
7.48
7.47
7.38
7.38
7.36
7.36
7.35
7.34
7.31
7.30
7.29
7.28
7.28
7.27
7.27
7.26
5.85
5.84
5.83
5.82
3.84
3.82
3.81
3.79
3.66
3.65
3.63
3.62
2.29
HO
O
S
N
NH
H
N
Cl
Cl
OMe
Ph
31
1H NMR (400 MHz, DMSO-d6)
0.86
2.00
1.00
2.15
3.21
1.06
1.07
1.18
1.18
3.06
f1 (ppm)

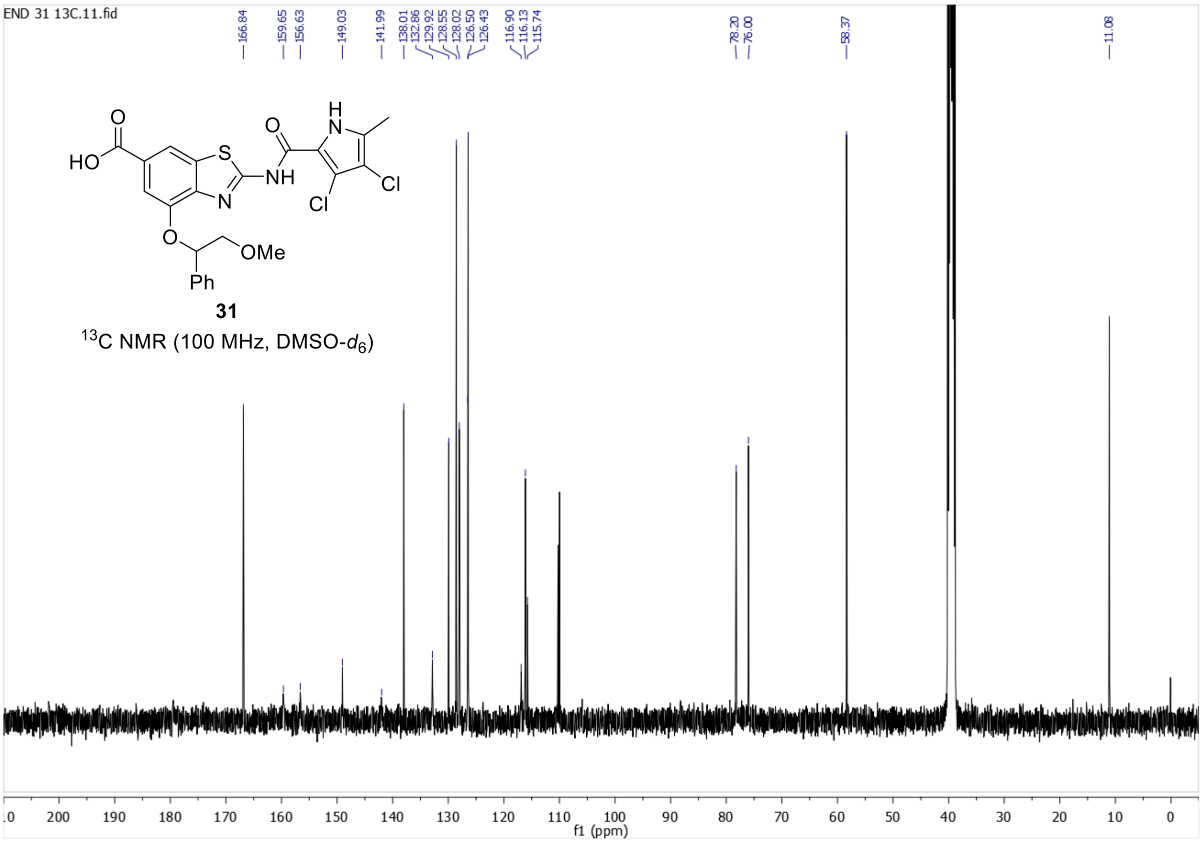
END 31 13C.11.fid
166.84
159.65
156.63
149.03
141.99
138.01
132.86
129.92
128.55
128.02
126.50
126.43
116.90
116.13
115.74
78.20
76.00
58.37
11.08
HO
O
S
N
NH
O
H
N
Cl
Cl
O
OMe
Ph
31
13C NMR (100 MHz, DMSO-d6)
f1 (ppm)

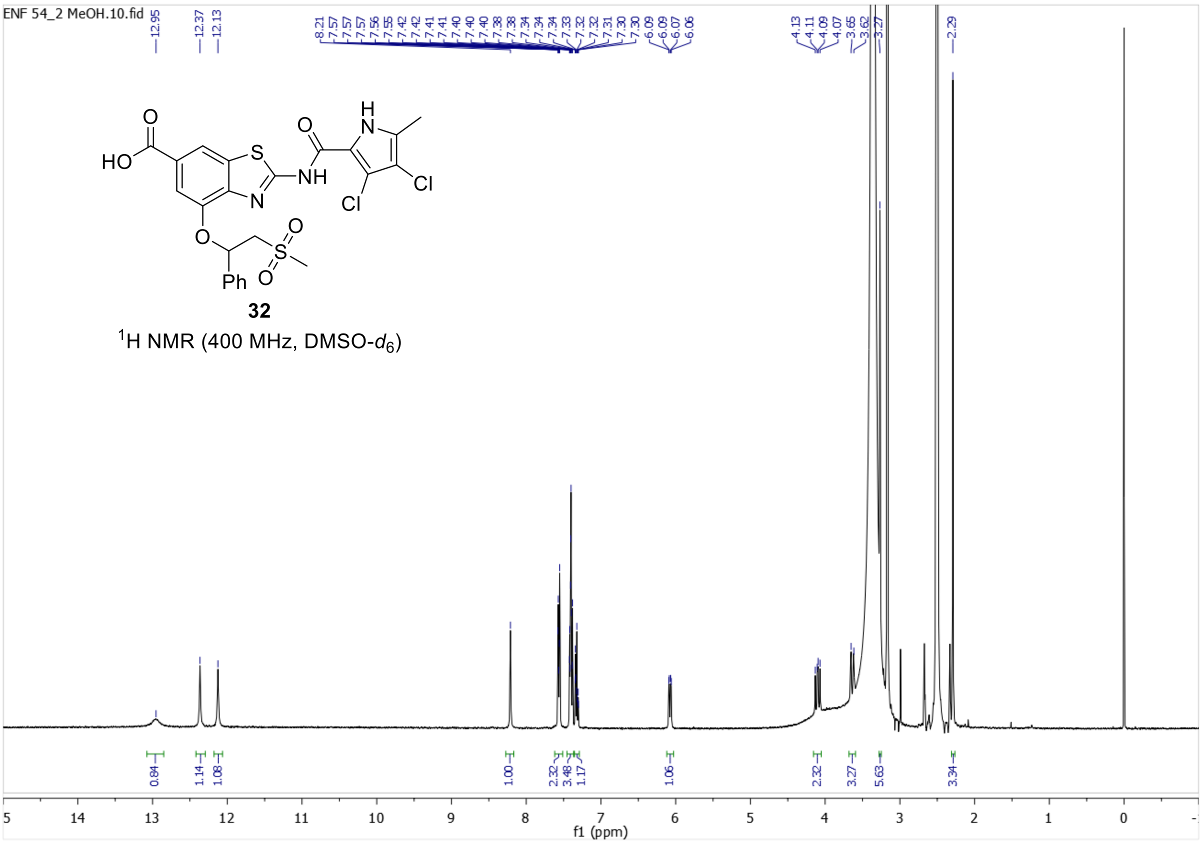
ENF 54_2 MeOH.10.fid
12.95
12.37
12.13
8.21
7.57
7.57
7.57
7.56
7.55
7.42
7.42
7.41
7.41
7.40
7.40
7.40
7.38
7.38
7.34
7.34
7.34
7.34
7.33
7.32
7.32
7.31
7.30
7.30
6.09
6.09
6.07
6.06
4.13
4.11
4.09
4.07
3.65
3.62
3.27
2.29
HO
O
S
N
NH
O
H
N
Cl
Cl
O
S
O
O
Ph
32
1H NMR (400 MHz, DMSO-d6)
0.84
1.14
1.08
1.00
2.32
3.48
1.17
1.06
2.32
3.27
5.63
3.34
f1 (ppm)

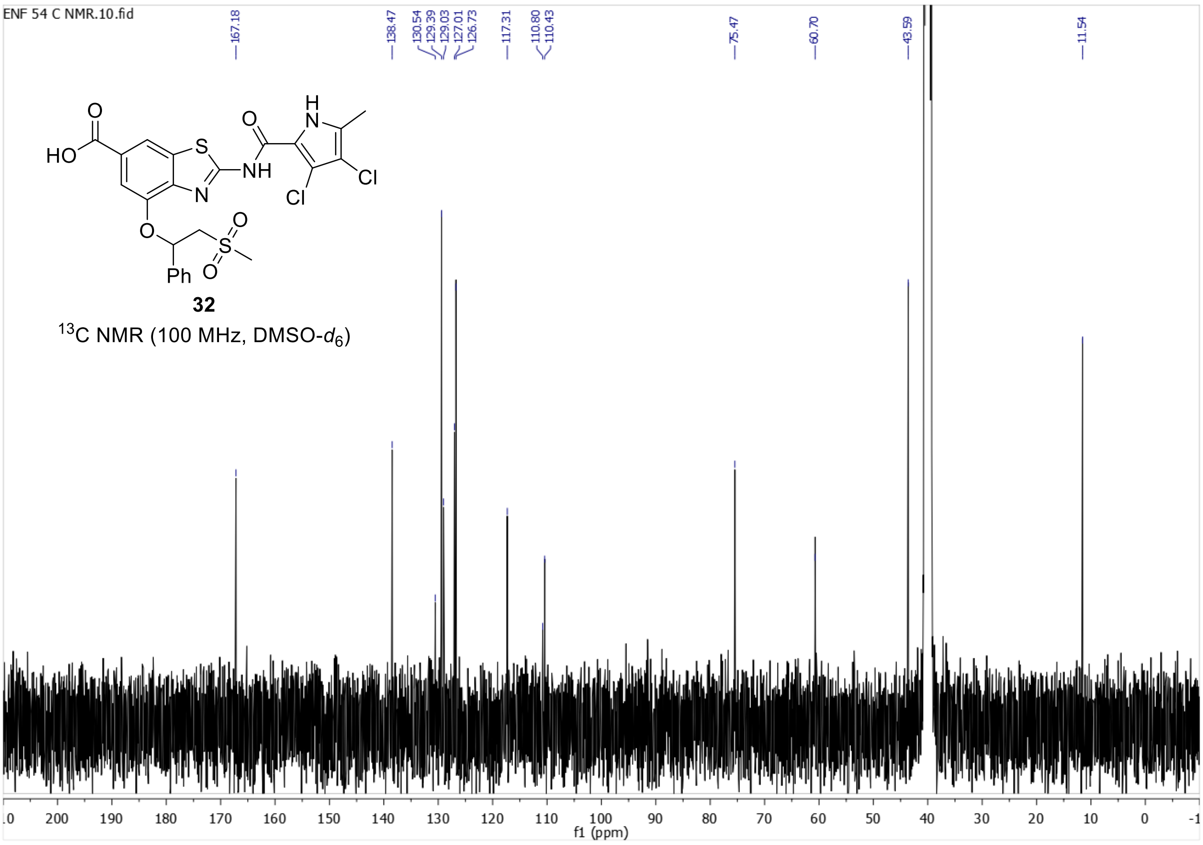
ENF 54 C NMR.10.fid
167.18
138.47
130.54
129.39
129.03
127.01
126.73
117.31
110.80
110.43
75.47
60.70
43.59
11.54
HO
O
S
N
NH
O
H
N
Cl
Cl
O
Ph
S
O
O
32
^{13}C NMR (100 MHz, DMSO-d_6)
f1 (ppm)

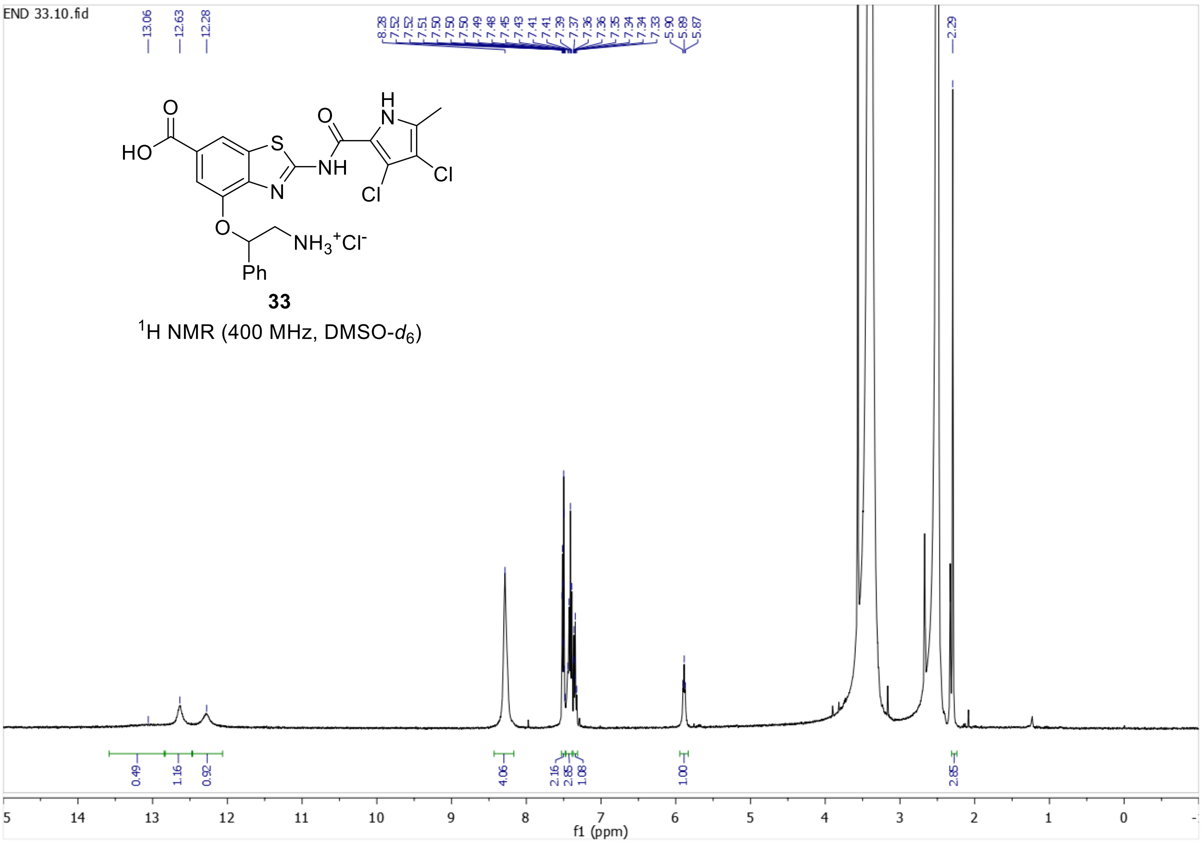
END 33.10.fid
13.06
12.63
12.28
8.28
7.52
7.52
7.51
7.50
7.50
7.50
7.49
7.48
7.45
7.43
7.41
7.41
7.39
7.37
7.36
7.36
7.35
7.34
7.34
7.33
5.90
5.89
5.87
2.29
HO
O
S
N
NH
O
H
N
Cl
Cl
O
Ph
NH$_3^+$Cl$^-$
33
^{1}H NMR (400 MHz, DMSO-d_6)
0.49
1.16
0.92
4.06
2.16
2.85
1.08
1.00
2.85
f1 (ppm)

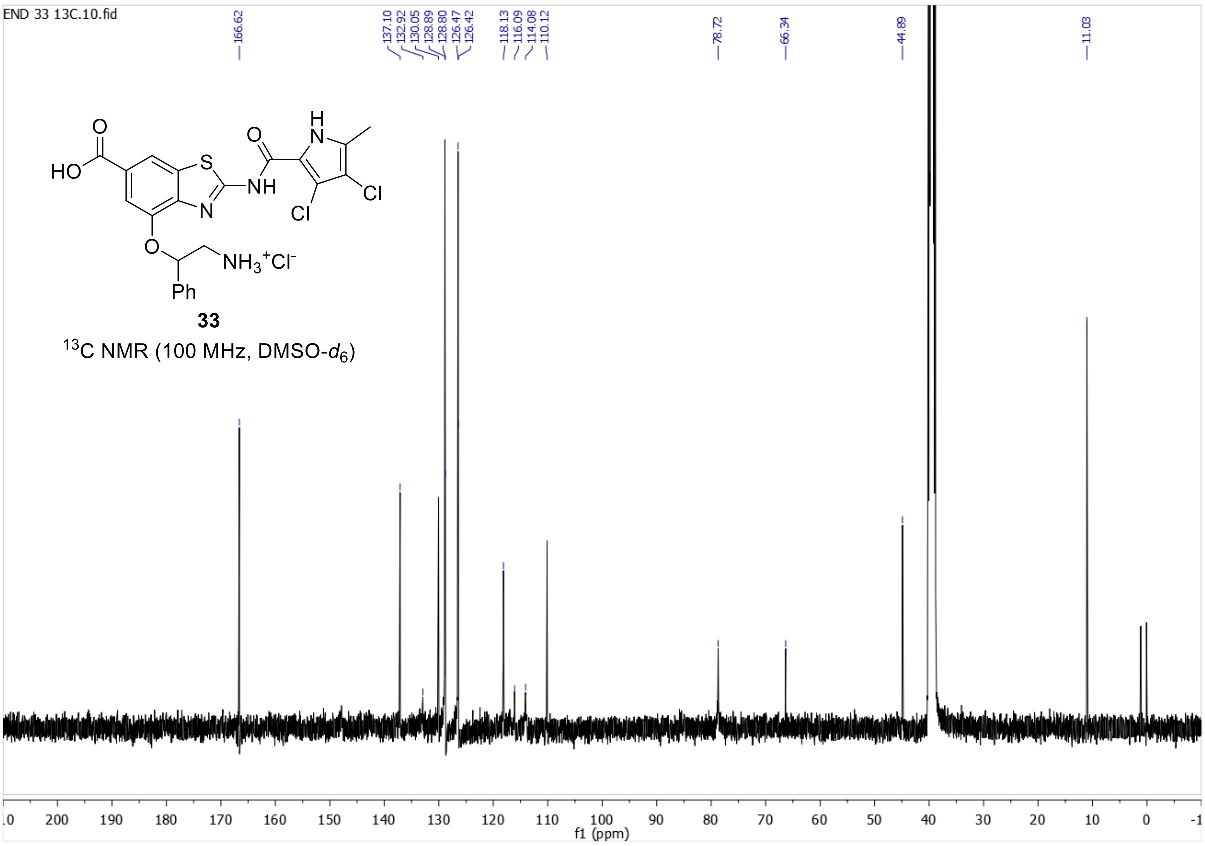
END 33 13C.10.fid
166.62
137.10
132.92
130.05
128.89
128.80
126.47
126.42
118.13
116.09
114.08
110.12
78.72
66.34
44.89
11.03
33
13C NMR (100 MHz, DMSO-d6)
f1 (ppm)

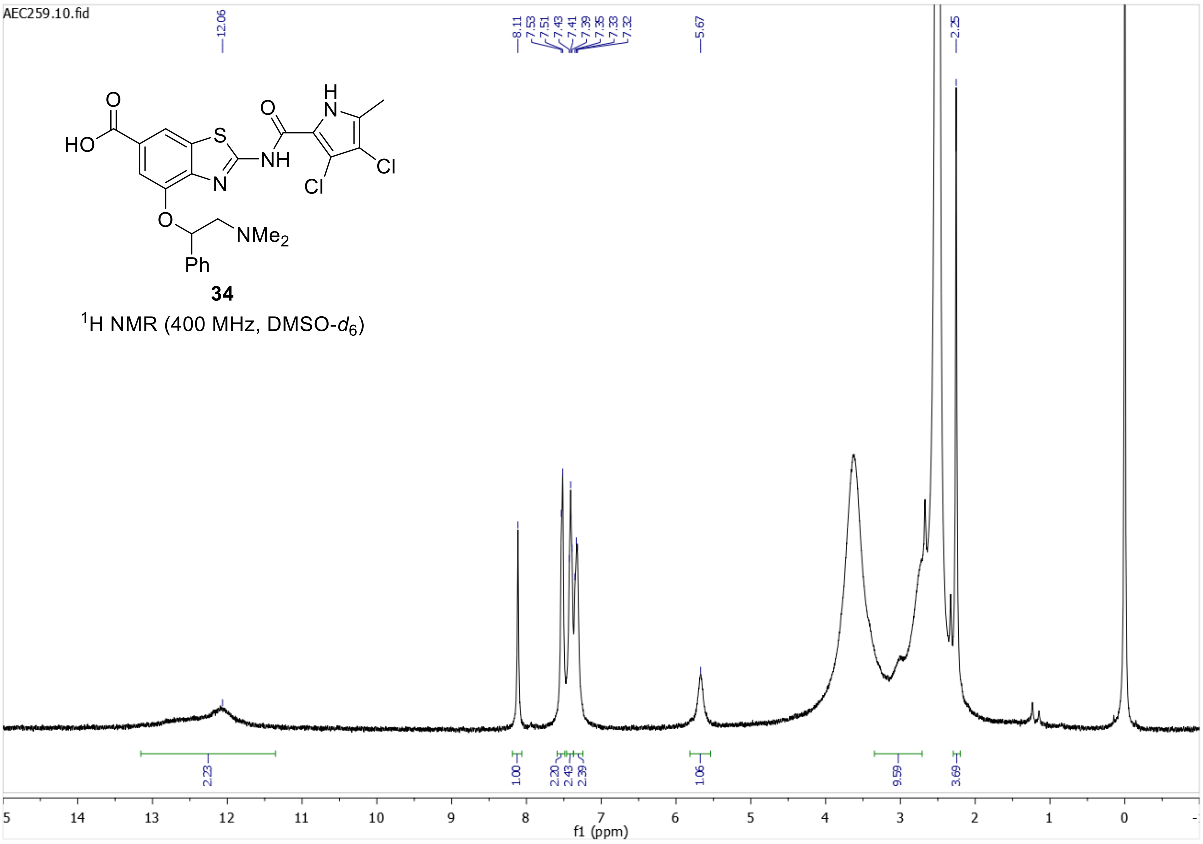
AEC259.10.fid
12.06
8.11
7.53
7.51
7.43
7.41
7.39
7.35
7.33
7.32
5.67
2.25
34
1H NMR (400 MHz, DMSO-d6)
2.23
1.00
2.20
2.43
2.39
1.06
9.59
3.69
f1 (ppm)

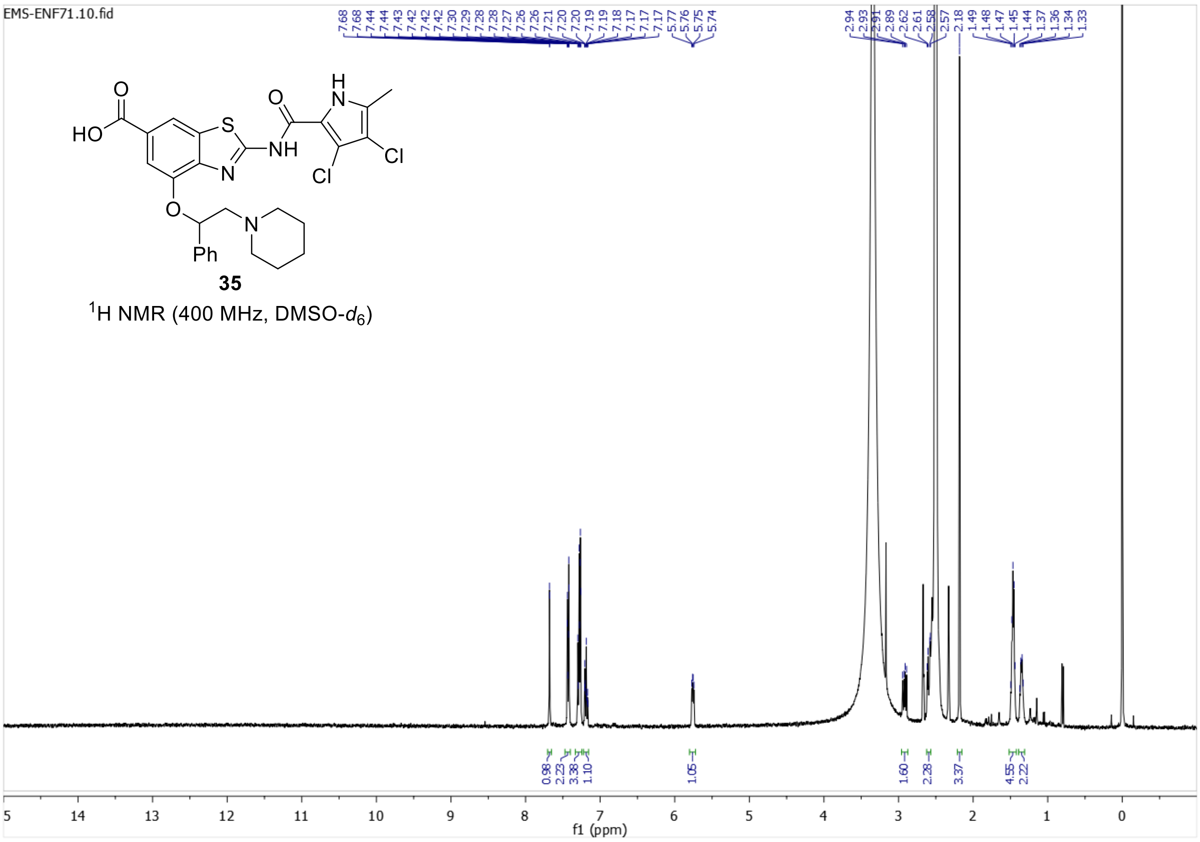
EMS-ENF71.10.fid
35
1H NMR (400 MHz, DMSO-d6)
f1 (ppm)

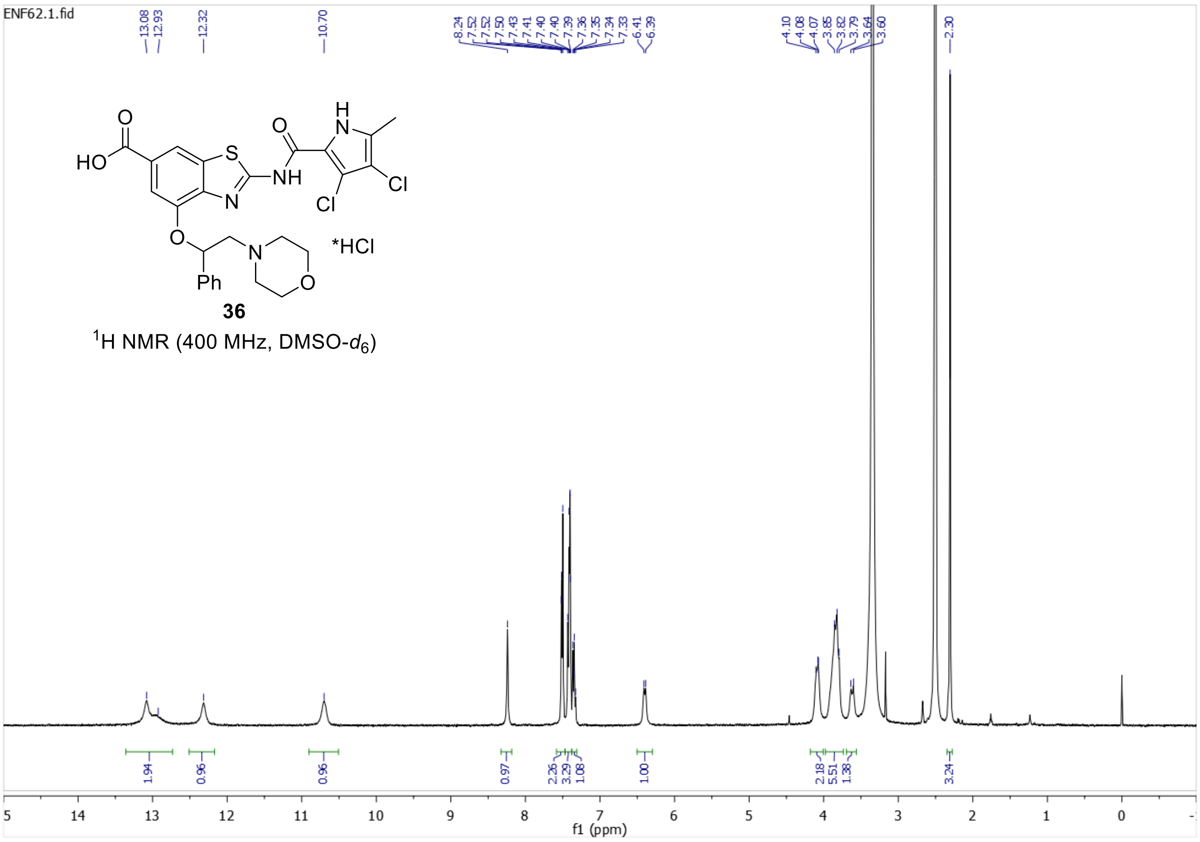
ENF62.1.fid
*HCl
36
1H NMR (400 MHz, DMSO-d6)
f1 (ppm)

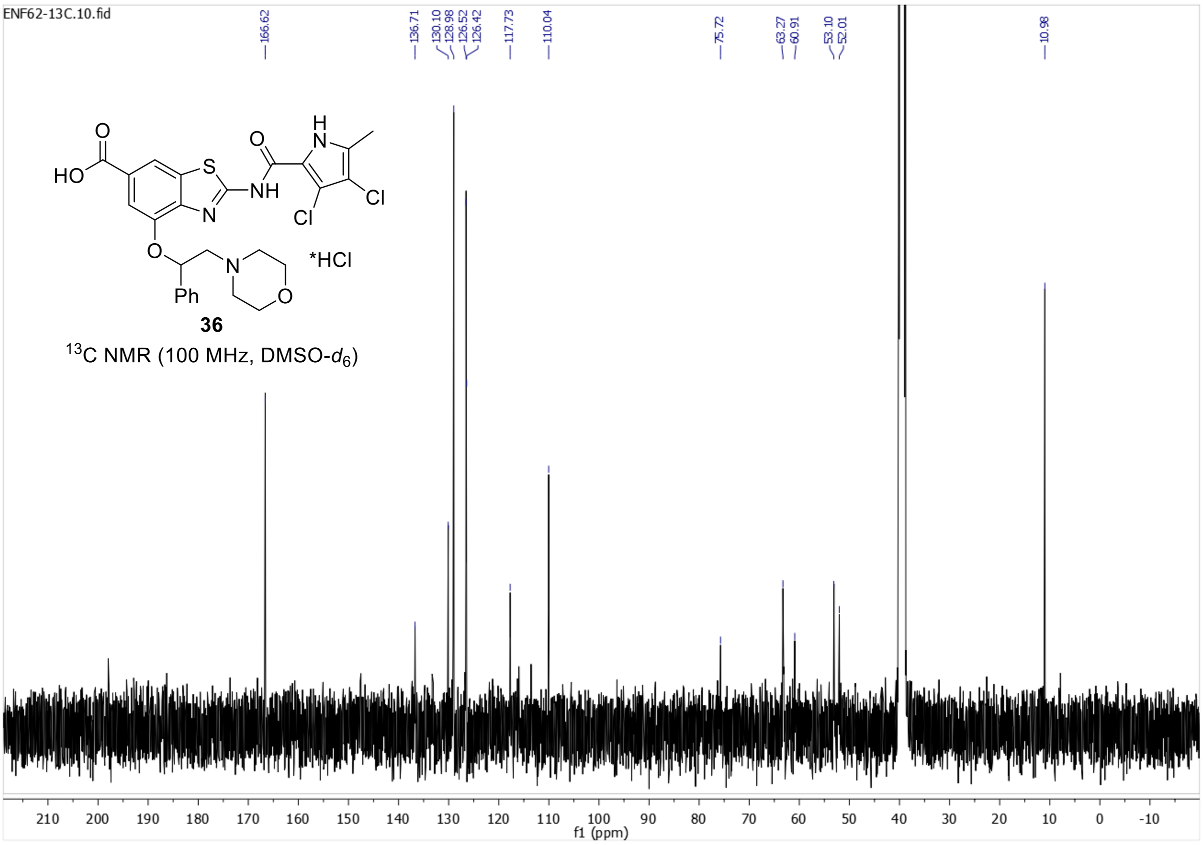
ENF62-13C.10.fid
166.62
136.71
130.10
128.98
126.52
126.42
117.73
110.04
75.72
63.27
60.91
53.10
52.01
10.98
HO
O
S
N
NH
O
H
N
Cl
Cl
O
N
O
Ph
*HCl
36
13C NMR (100 MHz, DMSO-d6)
210 200 190 180 170 160 150 140 130 120 110 100 90 80 70 60 50 40 30 20 10 0 -10
f1 (ppm)

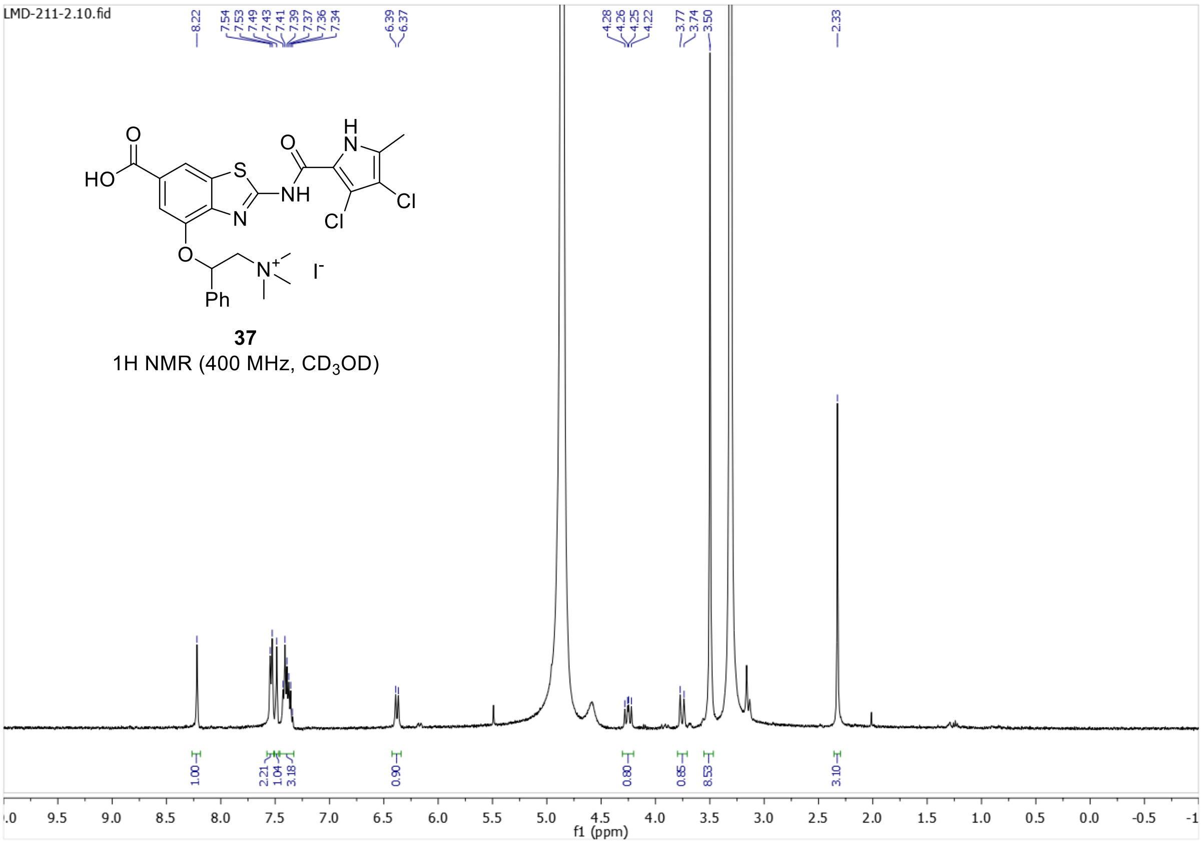
LMD-211-2.10.fid
8.22
7.54
7.53
7.49
7.43
7.41
7.39
7.37
7.36
7.34
6.39
6.37
4.28
4.26
4.25
4.22
3.77
3.74
3.50
2.33
HO
O
S
N
NH
O
H
N
Cl
Cl
O
N+
I-
Ph
37
1H NMR (400 MHz, CD3OD)
1.00
2.21
1.04
3.18
0.90
0.80
0.85
8.53
3.10
9.5 9.0 8.5 8.0 7.5 7.0 6.5 6.0 5.5 5.0 4.5 4.0 3.5 3.0 2.5 2.0 1.5 1.0 0.5 0.0 -0.5
f1 (ppm)

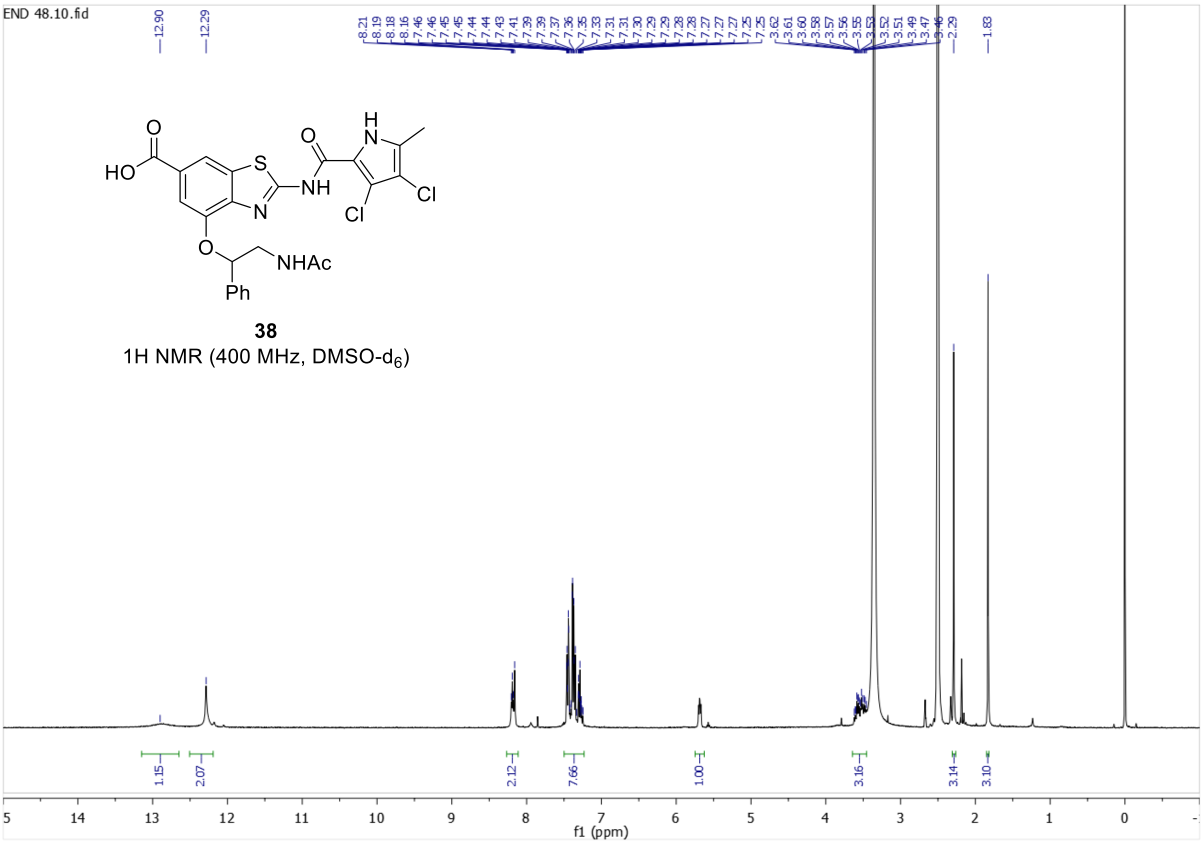
END 48.10.fid
12.90
12.29
8.21 8.19 8.18 8.16 7.46 7.46 7.45 7.45 7.44 7.43 7.41 7.39 7.39 7.37 7.36 7.35 7.33 7.31 7.31 7.30 7.29 7.29 7.28 7.28 7.27 7.27 7.27 7.25 7.25
3.62 3.61 3.60 3.58 3.57 3.56 3.55 3.53 3.52 3.51 3.49 3.47 3.46
2.29
1.83
O
HO
S
N
NH
O
H
N
Cl
Cl
O
NHAc
Ph
38
1H NMR (400 MHz, DMSO-d6)
1.15
2.07
2.12
7.66
1.00
3.16
3.14
3.10
f1 (ppm)

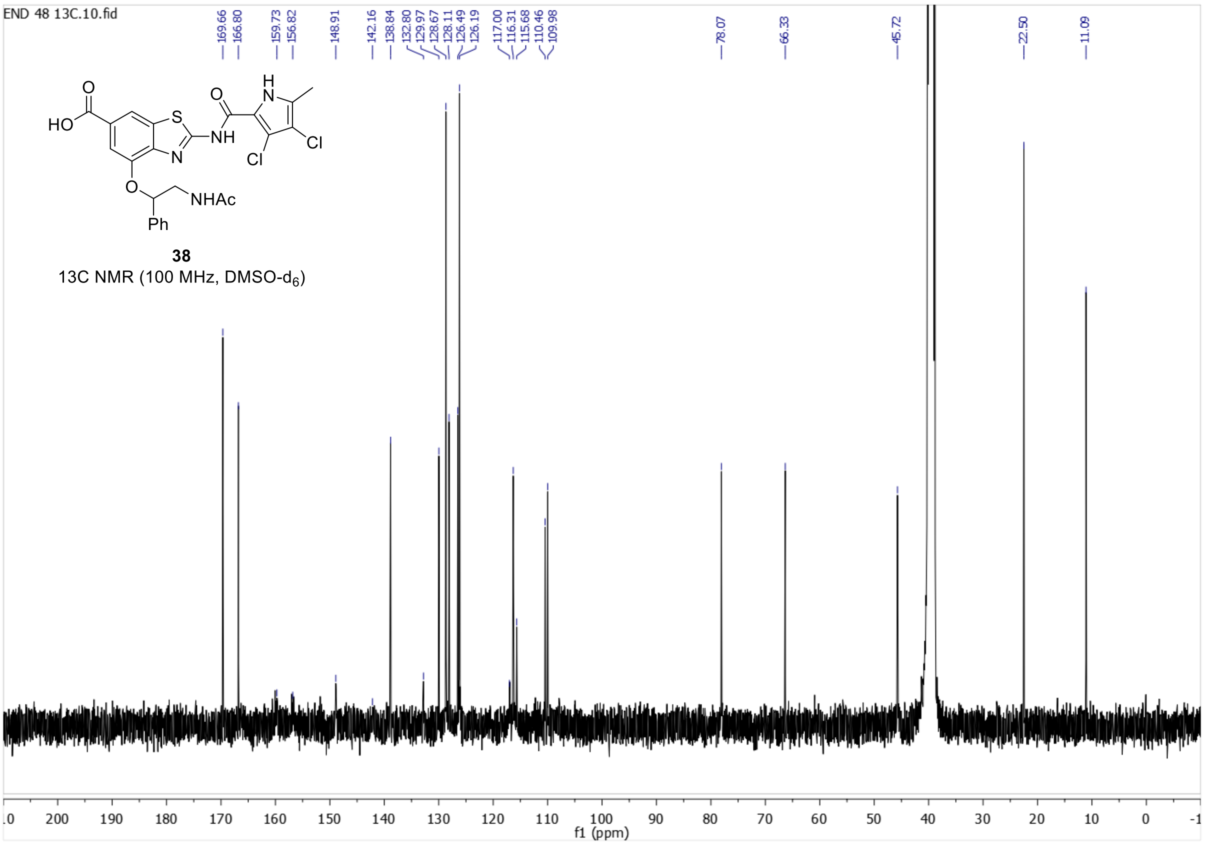
END 48 13C.10.fid
169.66
166.80
159.73
156.82
148.91
142.16
138.84
132.80
129.97
128.67
128.11
126.49
126.19
117.00
116.31
115.68
110.46
109.98
78.07
66.33
45.72
22.50
11.09
O
HO
S
N
NH
O
H
N
Cl
Cl
O
NHAc
Ph
38
13C NMR (100 MHz, DMSO-d6)
f1 (ppm)

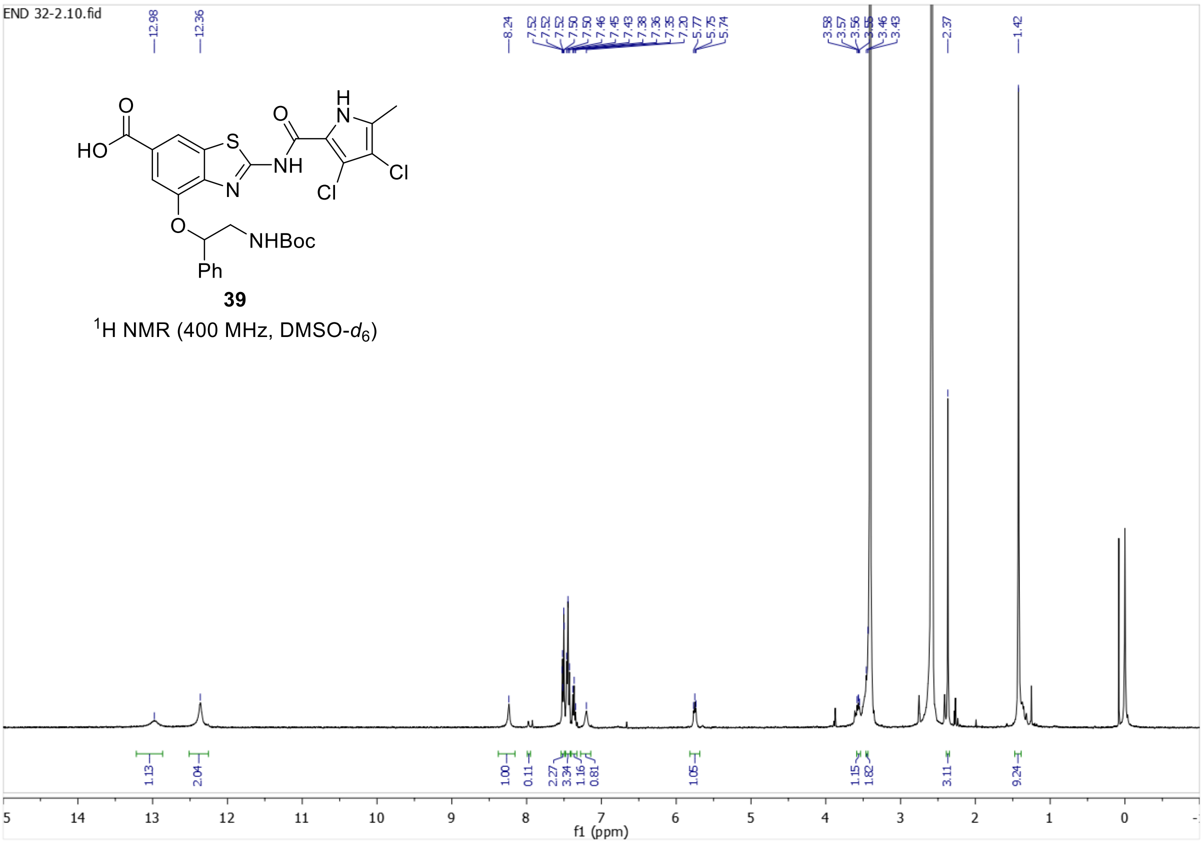
END 32-2.10.fid
HO
O
S
N
NH
O
H
N
Cl
Cl
O
NHBoc
Ph
39
1H NMR (400 MHz, DMSO-d6)
f1 (ppm)

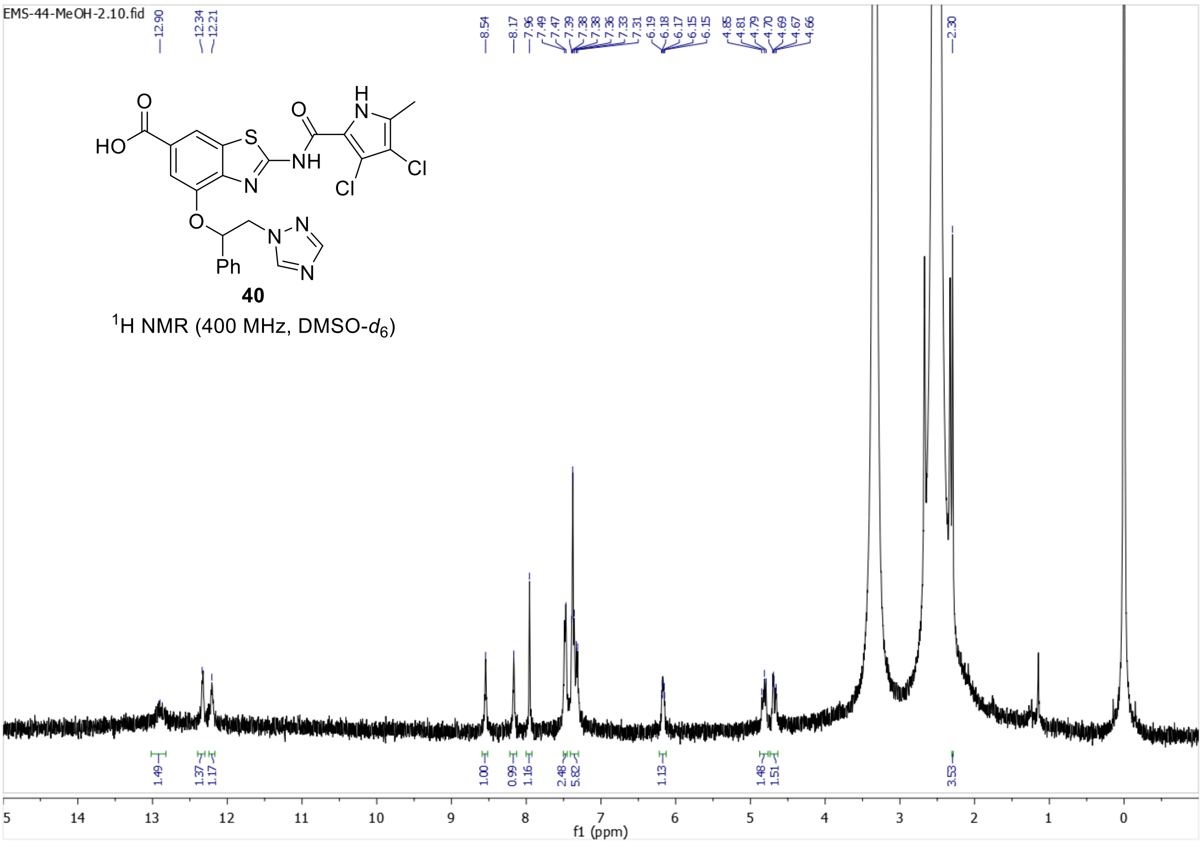
EMS-44-MeOH-2.10.fid
HO
O
S
N
NH
O
H
N
Cl
Cl
O
N
N
N
Ph
40
1H NMR (400 MHz, DMSO-d6)
f1 (ppm)

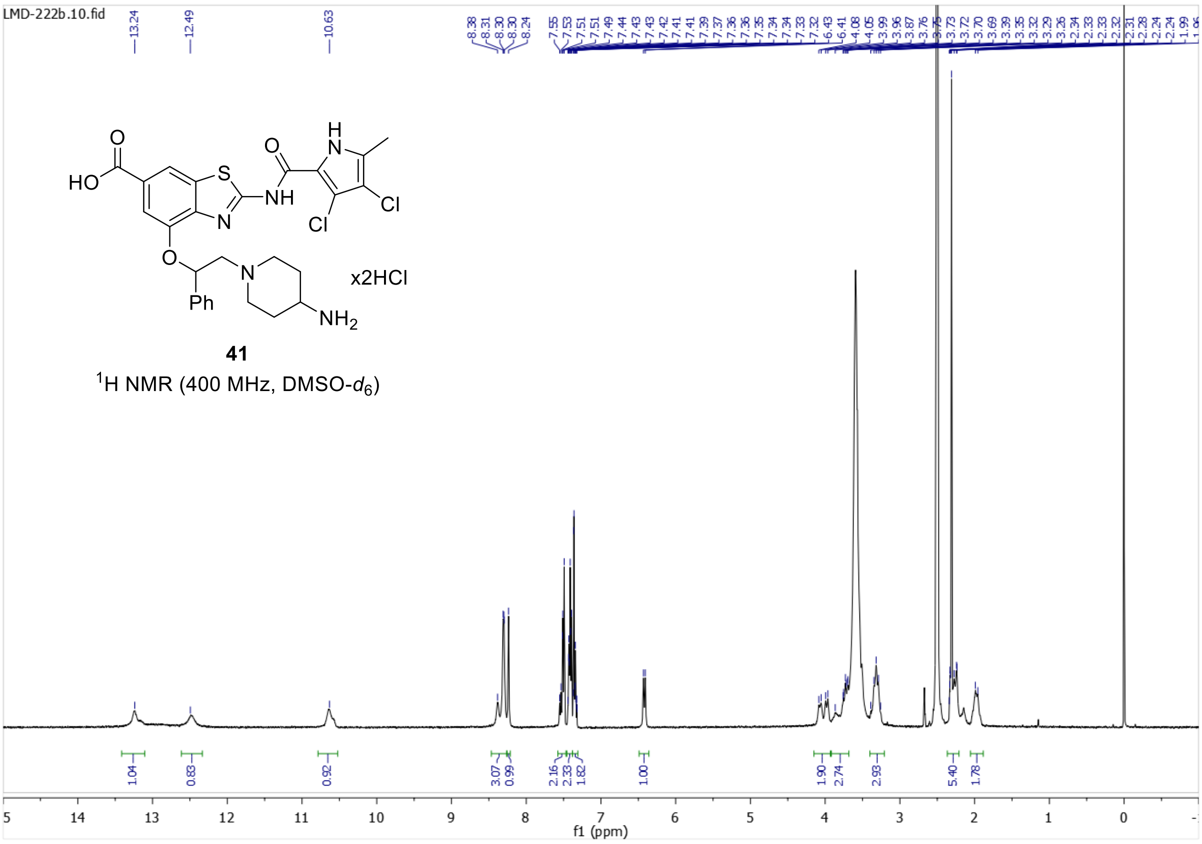

41

^{1}H NMR (400 MHz, DMSO-d_6)

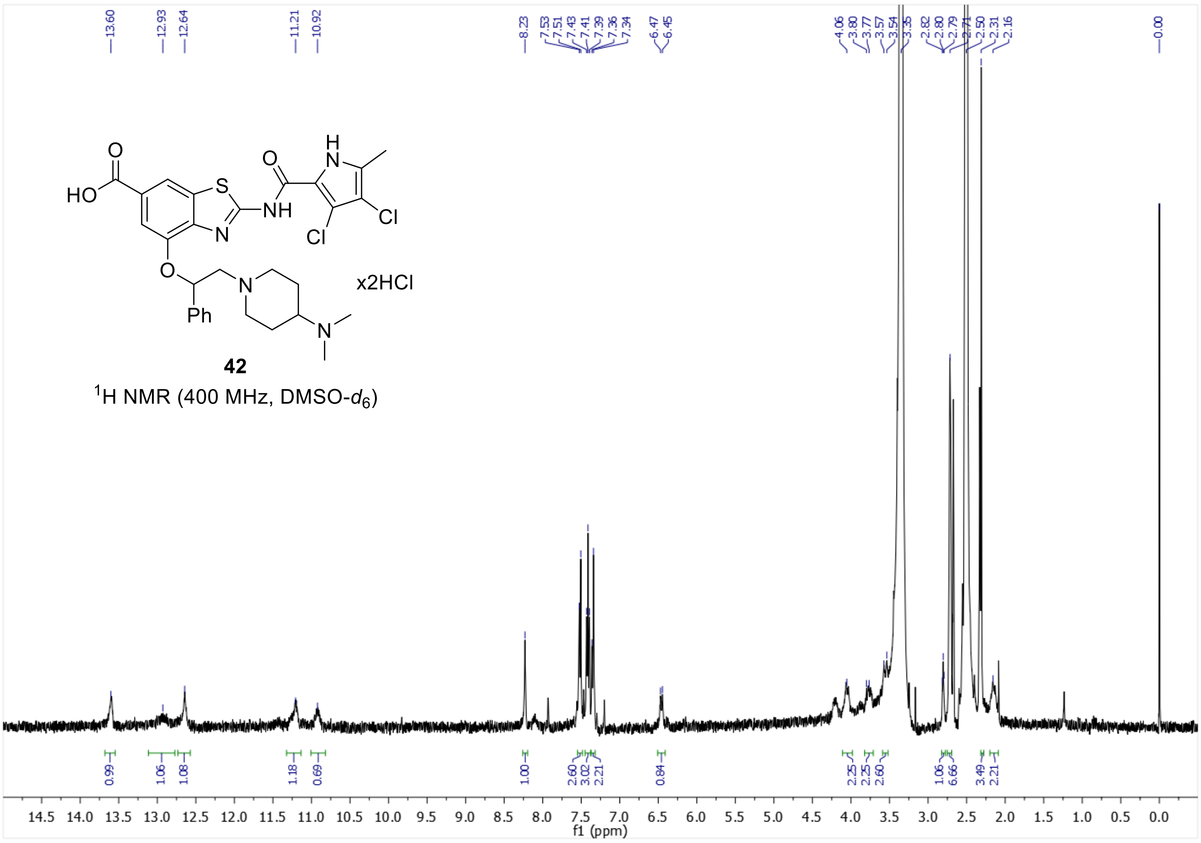

42

^{1}H NMR (400 MHz, DMSO-d_6)

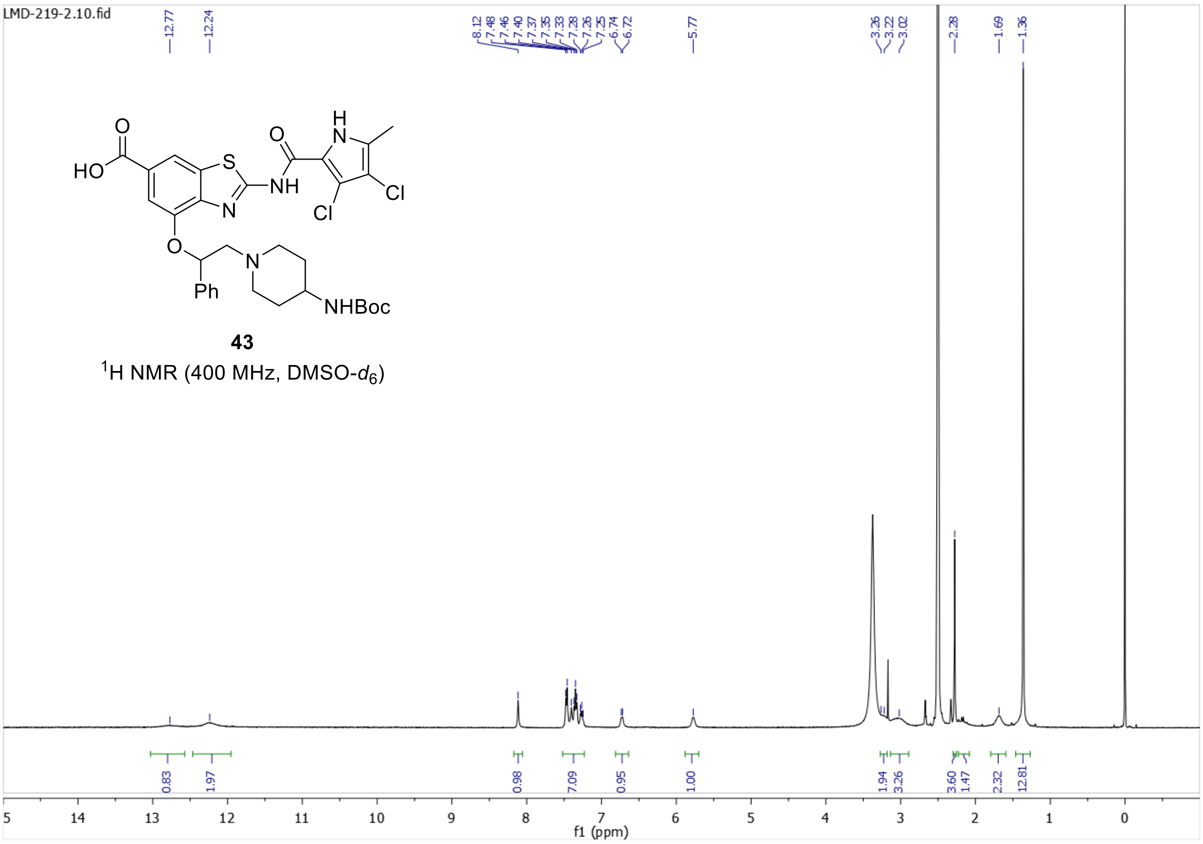

43

^{1}H NMR (400 MHz, DMSO-d_6)

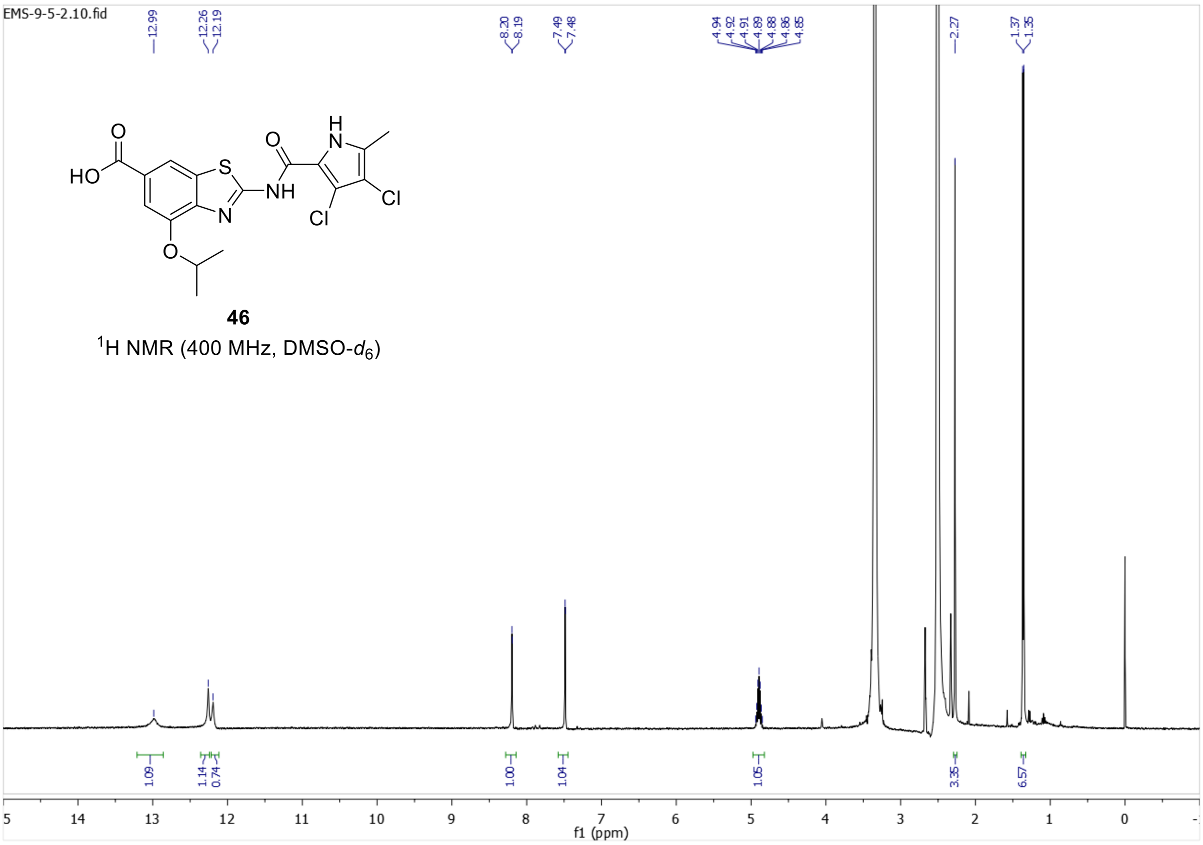

46

^{1}H NMR (400 MHz, DMSO-d_6)

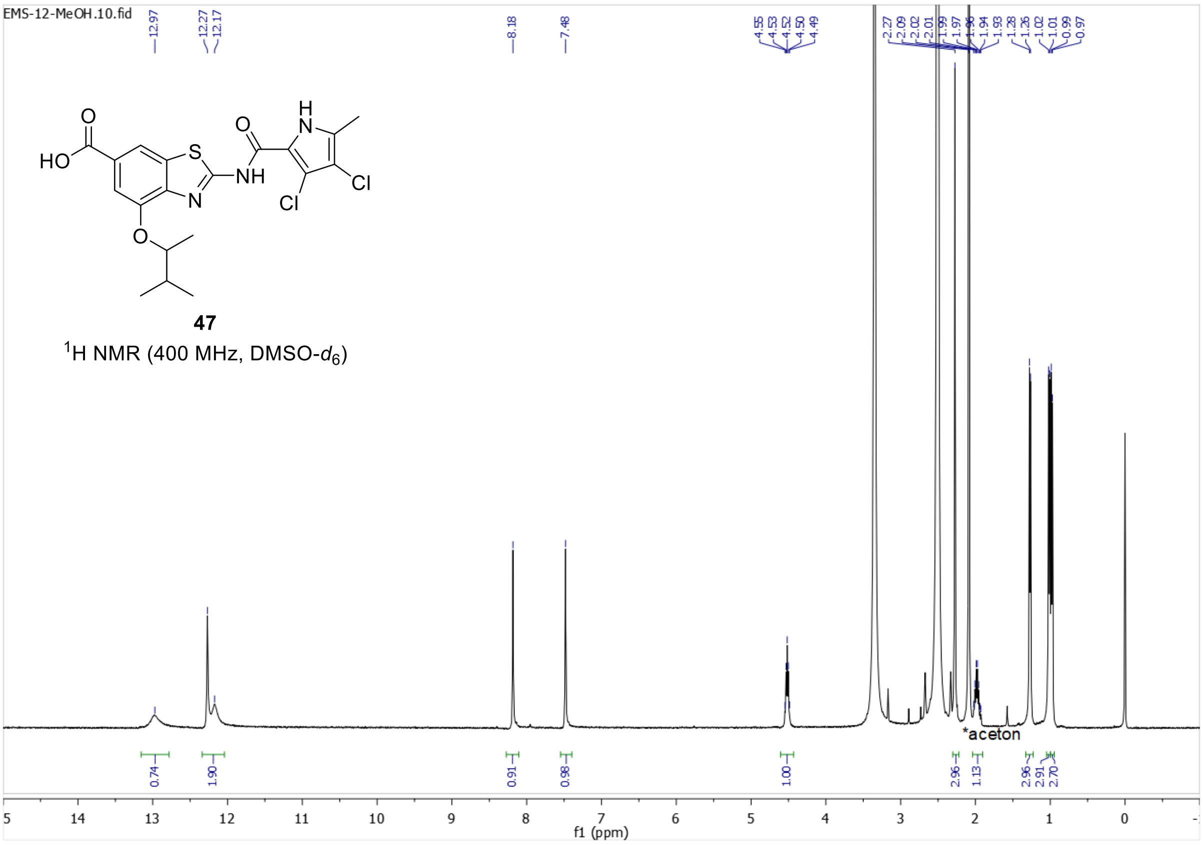
EMS-12-MeOH.10.fid
47
1H NMR (400 MHz, DMSO-d6)
*aceton
f1 (ppm)

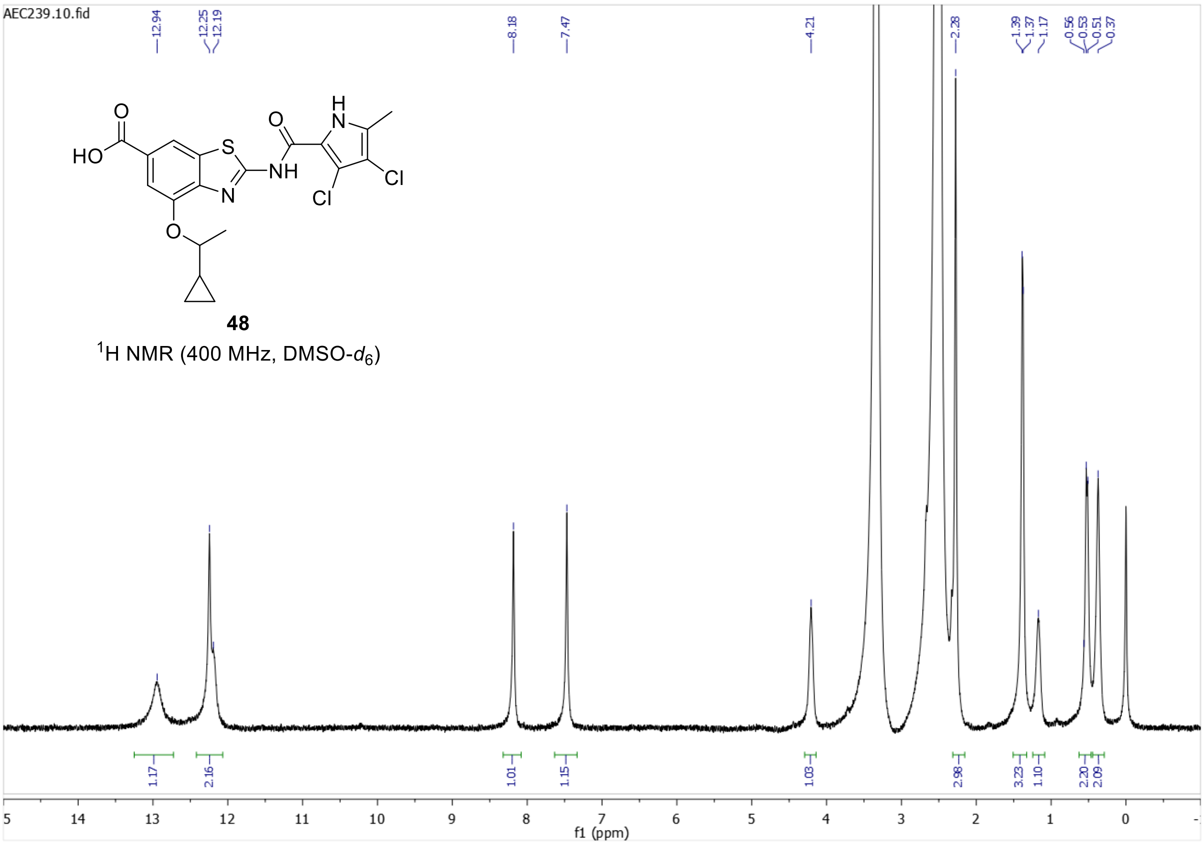
AEC239.10.fid
48
1H NMR (400 MHz, DMSO-d6)
f1 (ppm)

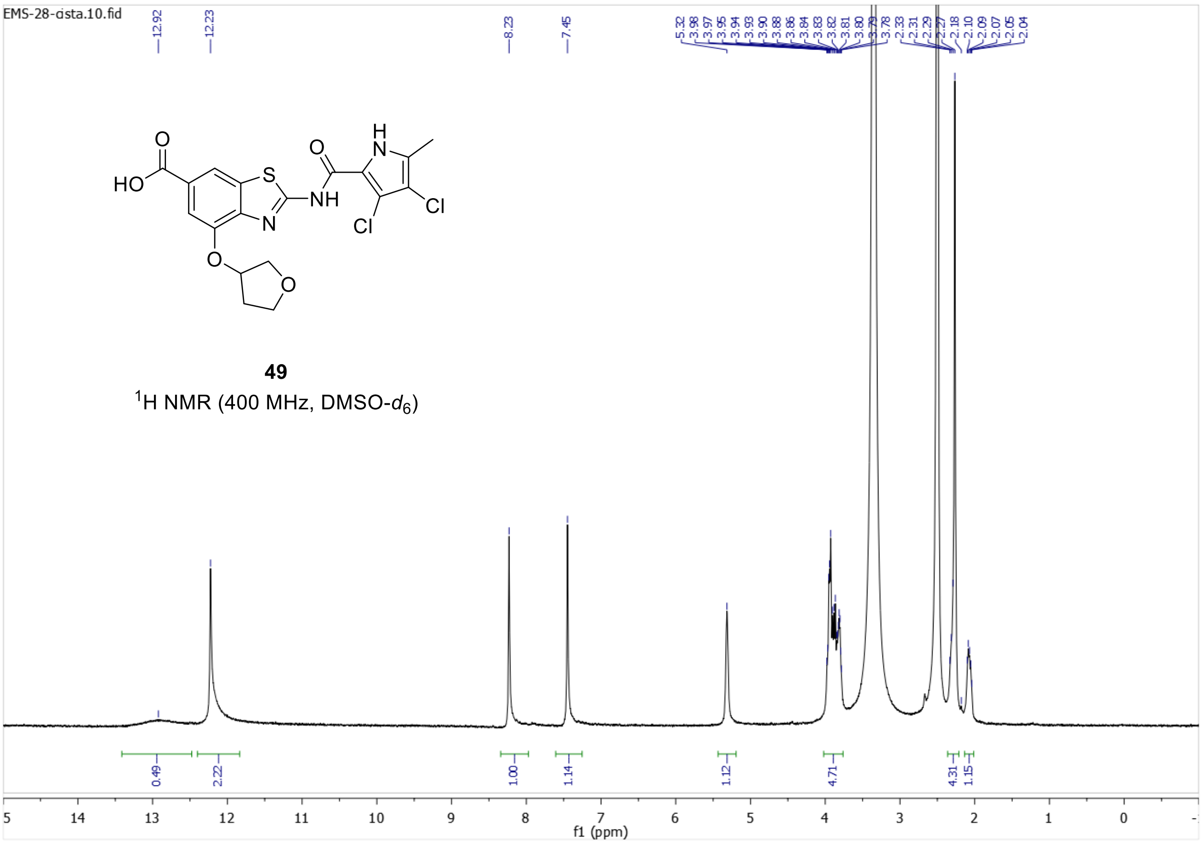
EMS-28-cista.10.fid
49
^{1}H NMR (400 MHz, DMSO-d_6)
f1 (ppm)

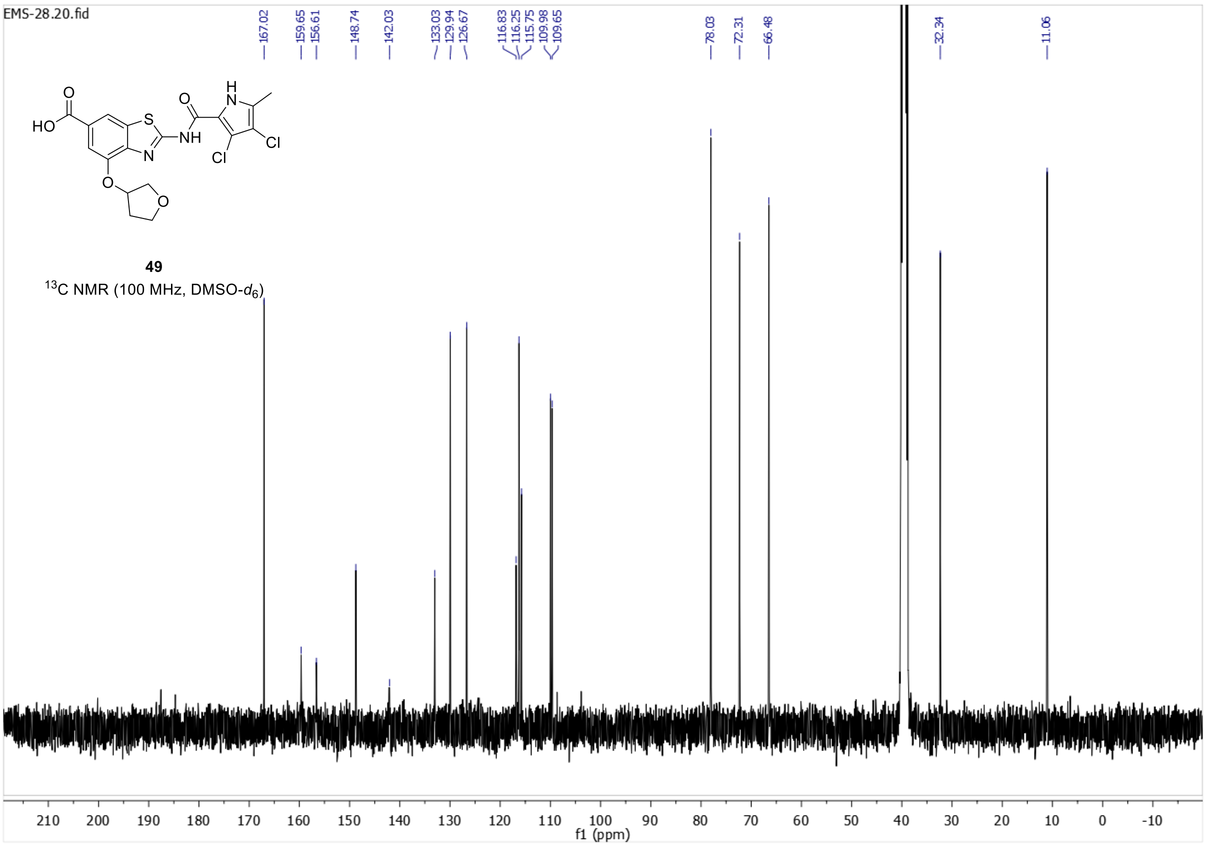
EMS-28.20.fid
49
^{13}C NMR (100 MHz, DMSO-d_6)
f1 (ppm)

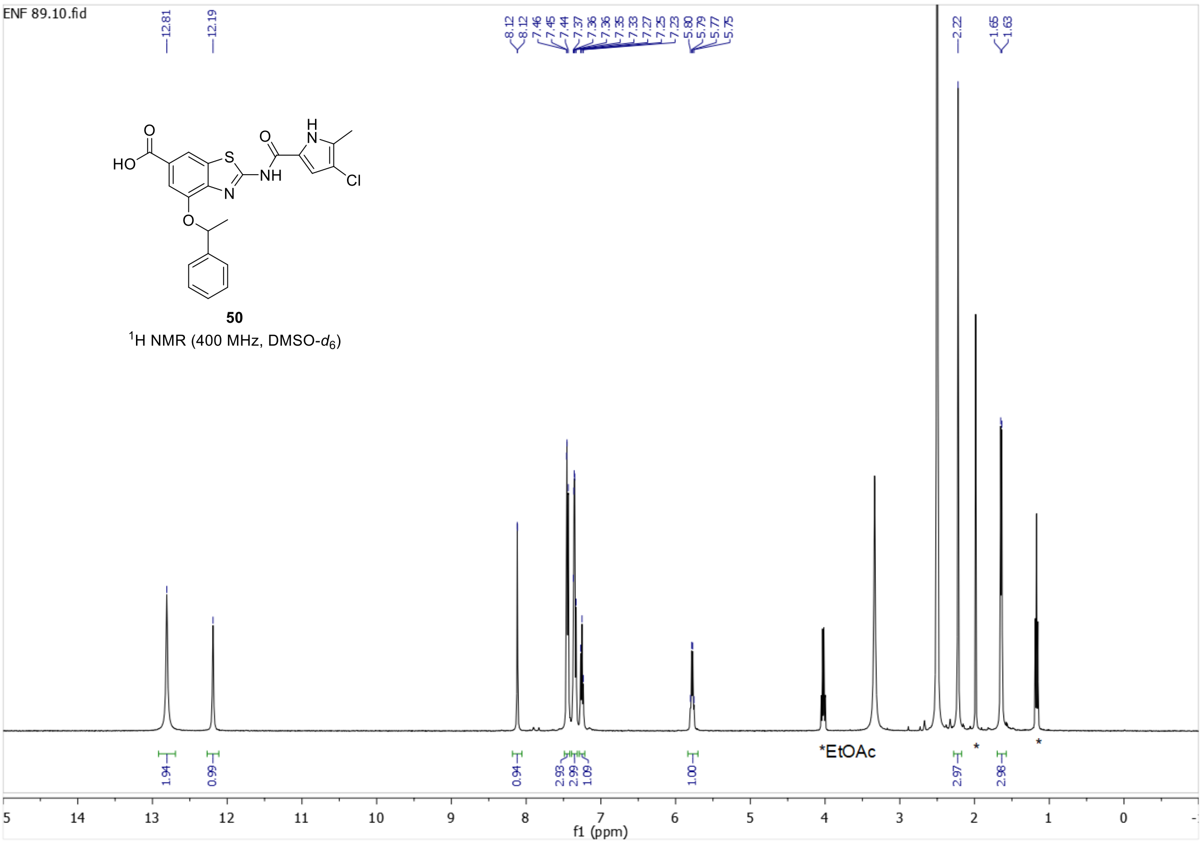
ENF 89.10.fid
12.81
12.19
8.12
8.12
7.46
7.45
7.44
7.37
7.36
7.36
7.35
7.33
7.27
7.25
7.23
5.80
5.79
5.77
5.75
2.22
1.65
1.63
50
^{1}H NMR (400 MHz, DMSO-d_6)
*EtOAc
1.94
0.99
0.94
2.93
2.99
1.09
1.00
2.97
2.98
f1 (ppm)

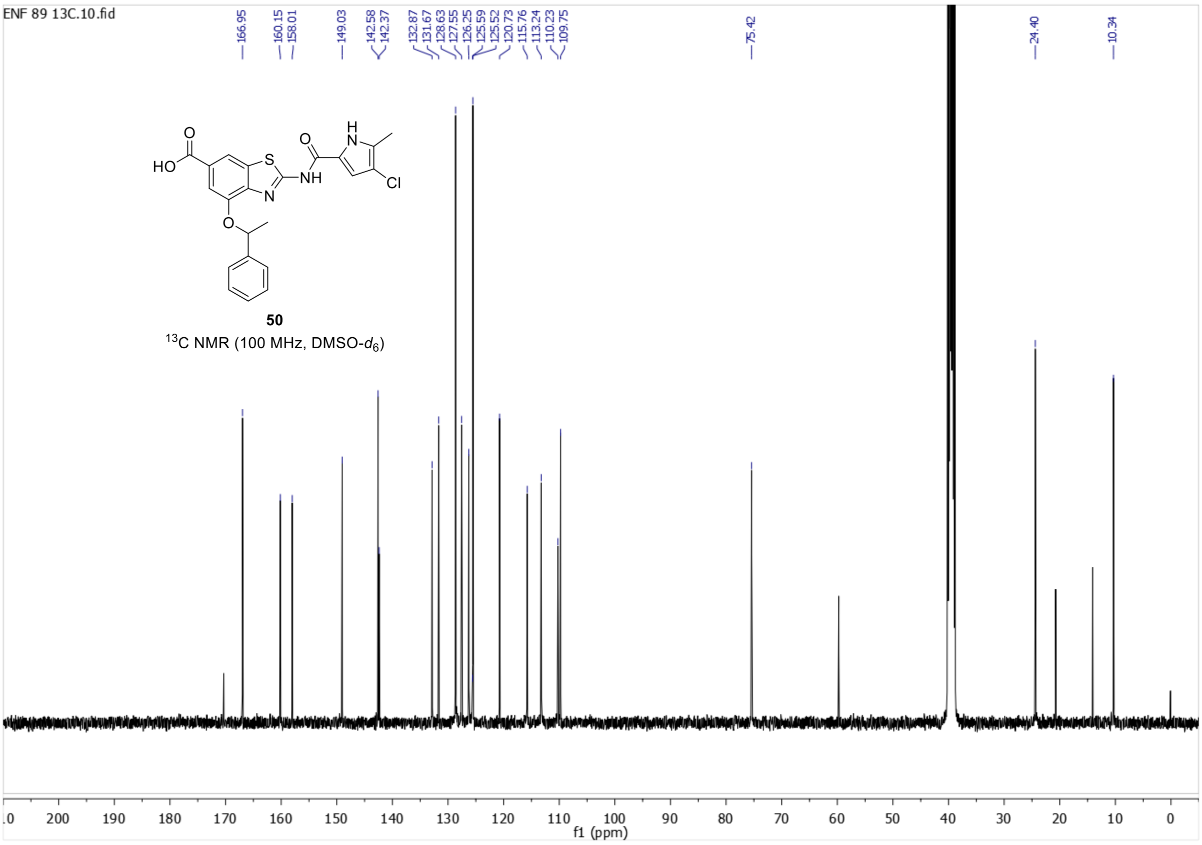
ENF 89 13C.10.fid
166.95
160.15
158.01
149.03
142.58
142.37
132.87
131.67
128.63
127.55
126.25
125.59
125.52
120.73
115.76
113.24
110.23
109.75
75.42
24.40
10.34
50
^{13}C NMR (100 MHz, DMSO-d_6)
f1 (ppm)

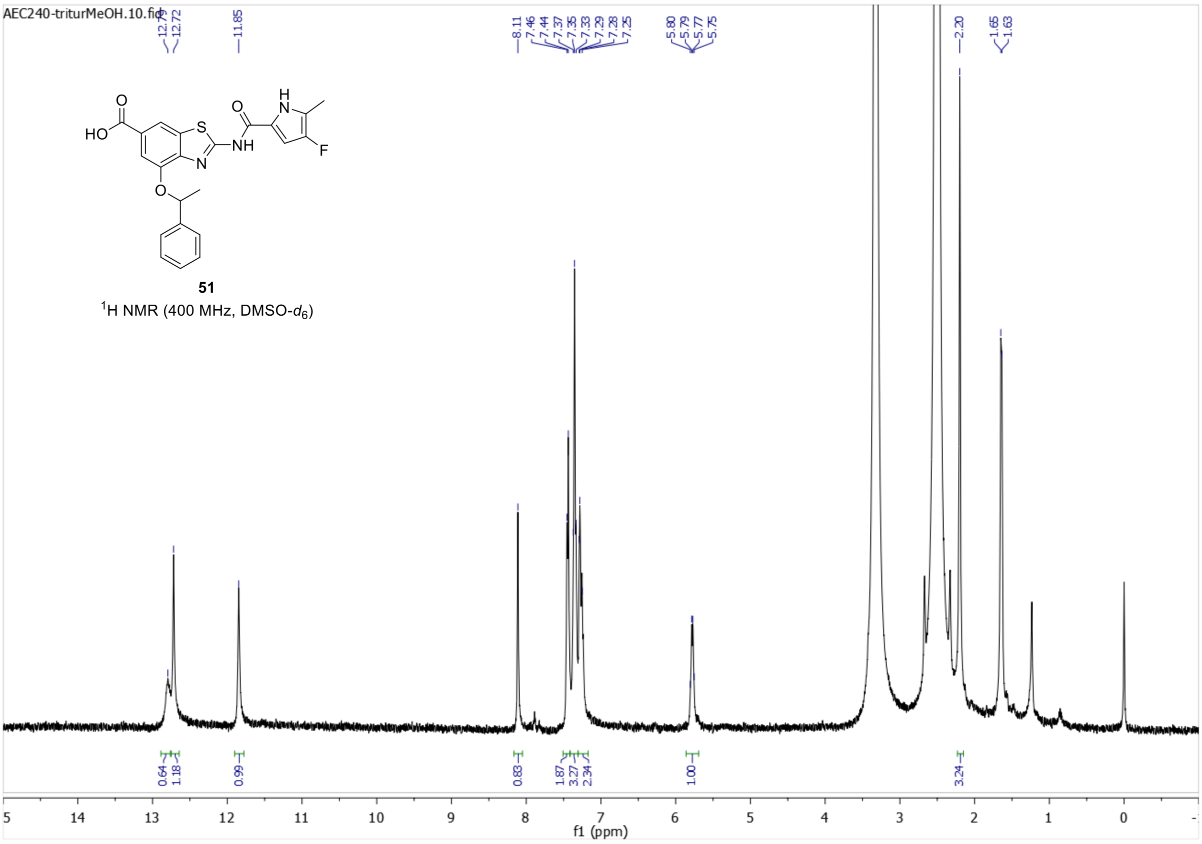
AEC240-triturMeOH.10.fid
12.79
12.72
11.85
8.11
7.46
7.44
7.37
7.35
7.33
7.29
7.28
7.25
5.80
5.79
5.77
5.75
2.20
1.65
1.63
HO
O
S
N
NH
O
H
N
F
O
51
^{1}H NMR (400 MHz, DMSO-d_6)
0.64
1.18
0.99
0.83
1.87
3.27
2.34
1.00
3.24
f1 (ppm)

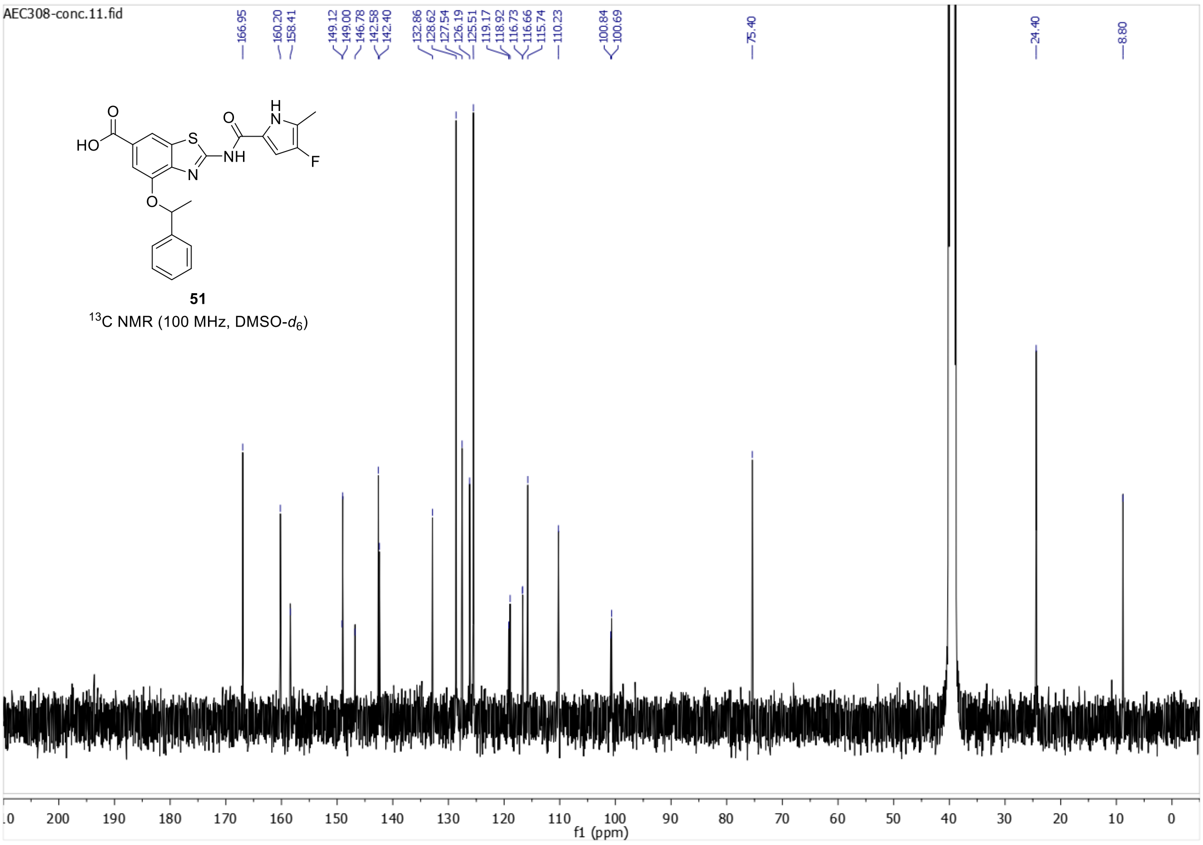
AEC308-conc.11.fid
166.95
160.20
158.41
149.12
149.00
146.78
142.58
142.40
132.86
128.62
127.54
126.19
125.51
119.17
118.92
116.73
116.66
115.74
110.23
100.84
100.69
75.40
24.40
8.80
HO
O
S
N
NH
O
H
N
F
O
51
^{13}C NMR (100 MHz, DMSO-d_6)
f1 (ppm)

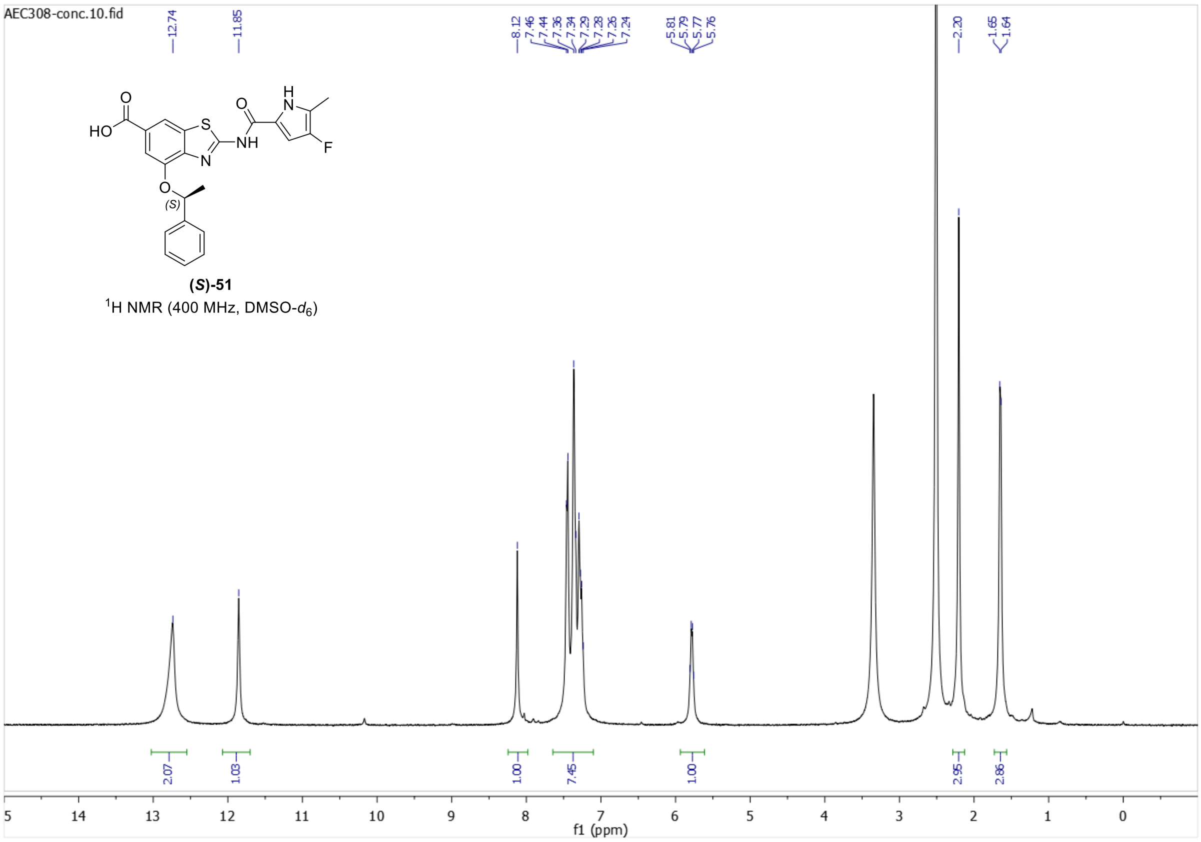
AEC308-conc.10.fid
(S)-51
^{1}H NMR (400 MHz, DMSO-d_6)
f1 (ppm)

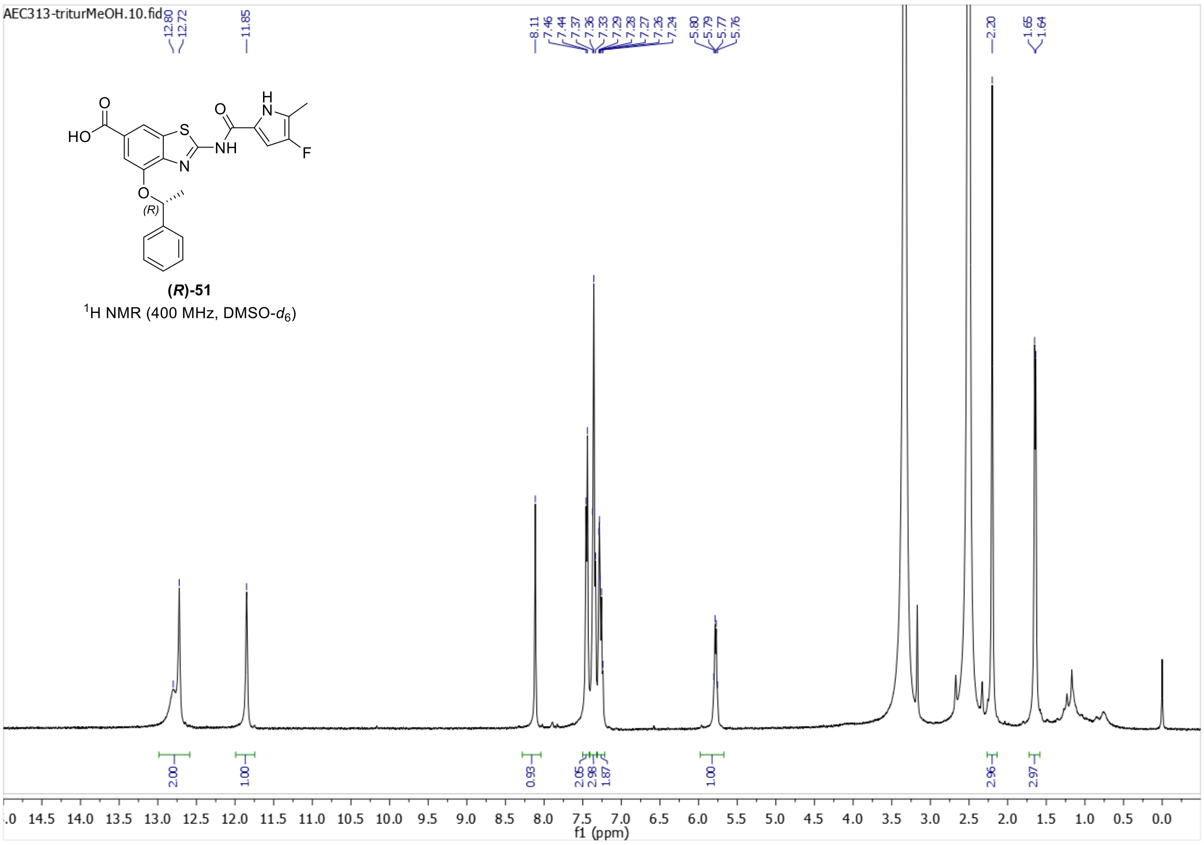
AEC313-triturMeOH.10.fid
(R)-51
^{1}H NMR (400 MHz, DMSO-d_6)
f1 (ppm)

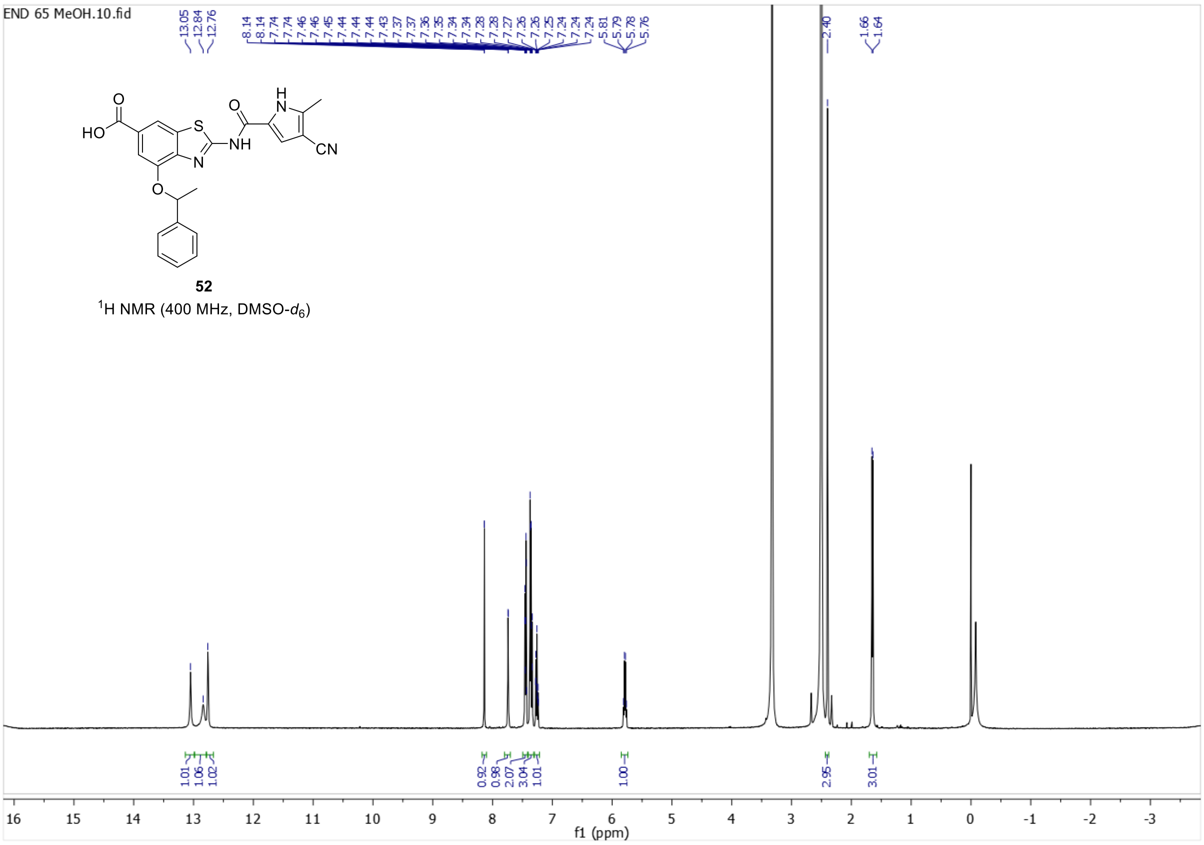
END 65 MeOH.10.fid
HO
O
S
N
NH
O
H
N
CN
O
52
1H NMR (400 MHz, DMSO-d6)
f1 (ppm)

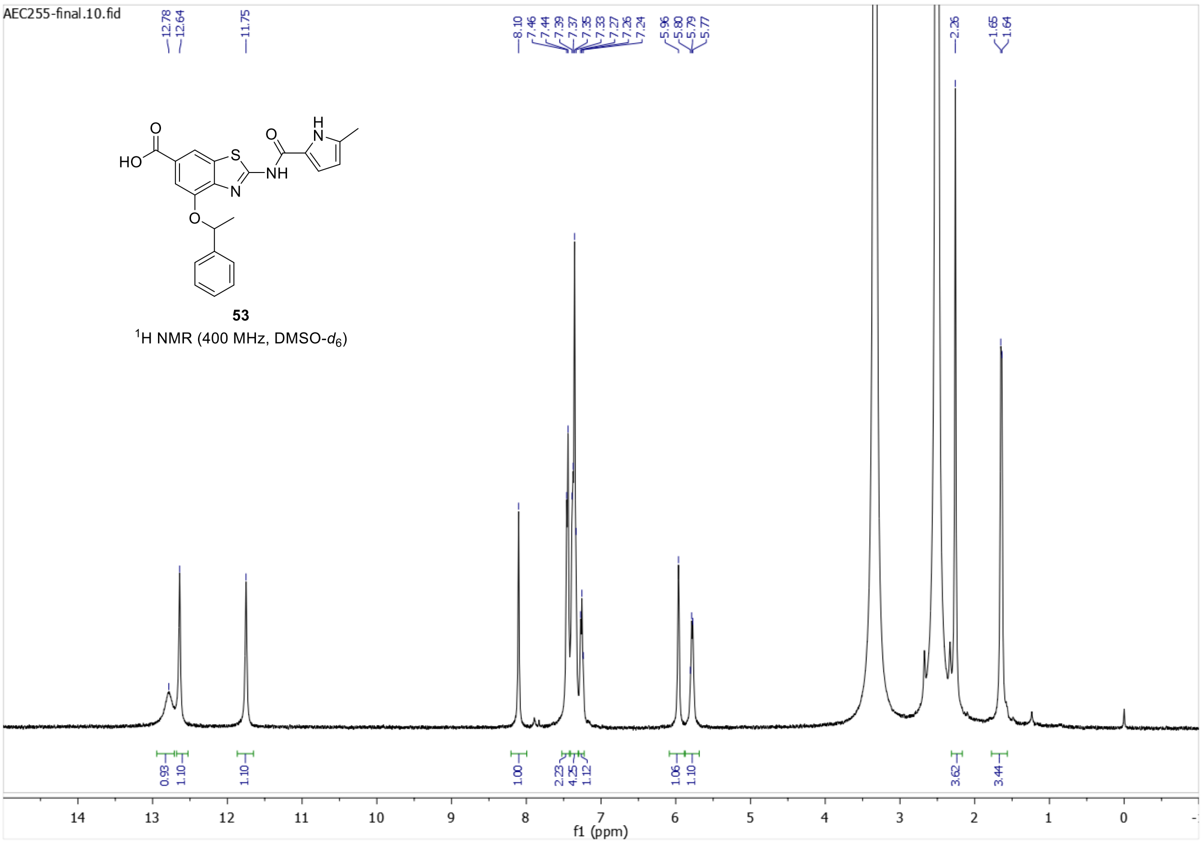
AEC255-final.10.fid
HO
O
S
N
NH
O
H
N
O
53
1H NMR (400 MHz, DMSO-d6)
f1 (ppm)

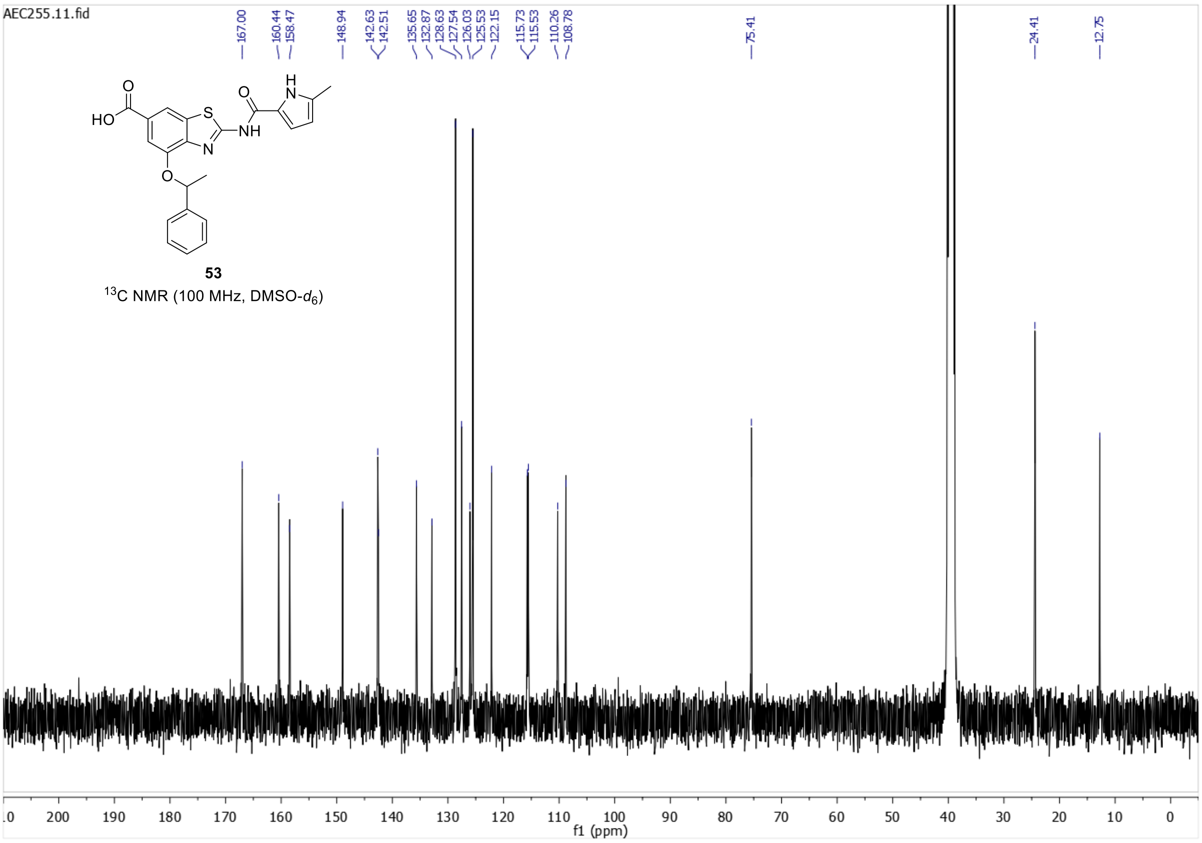

^{13}C NMR (100 MHz, DMSO-d_6)

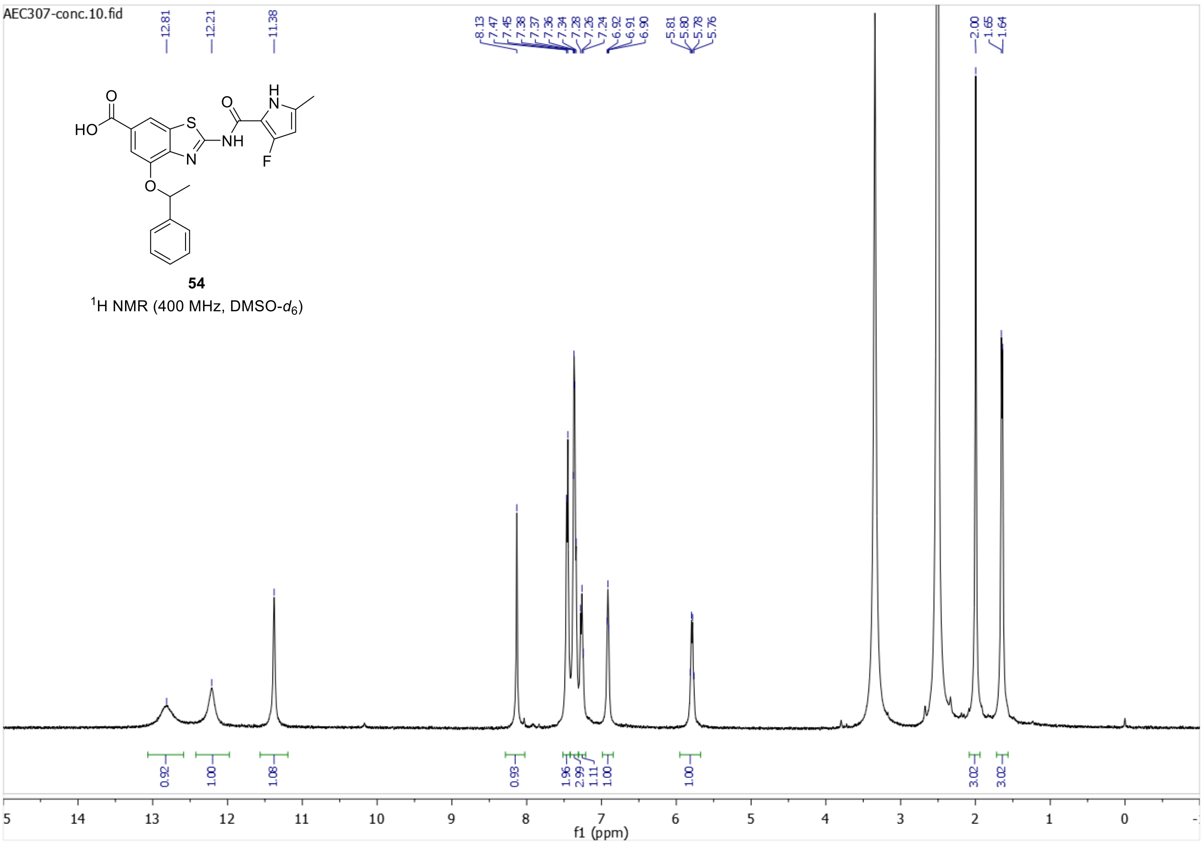

^{1}H NMR (400 MHz, DMSO-d_6)

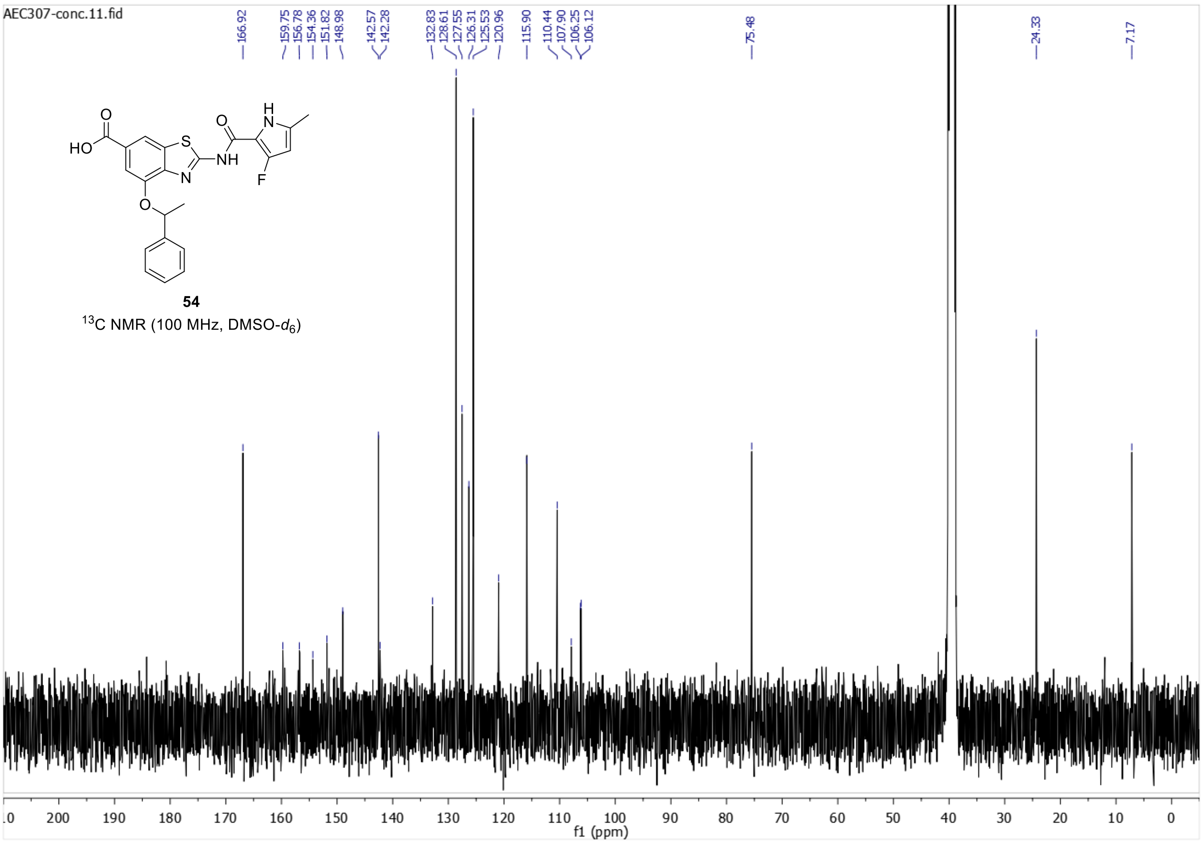

54

^{13}C NMR (100 MHz, DMSO-d_6)

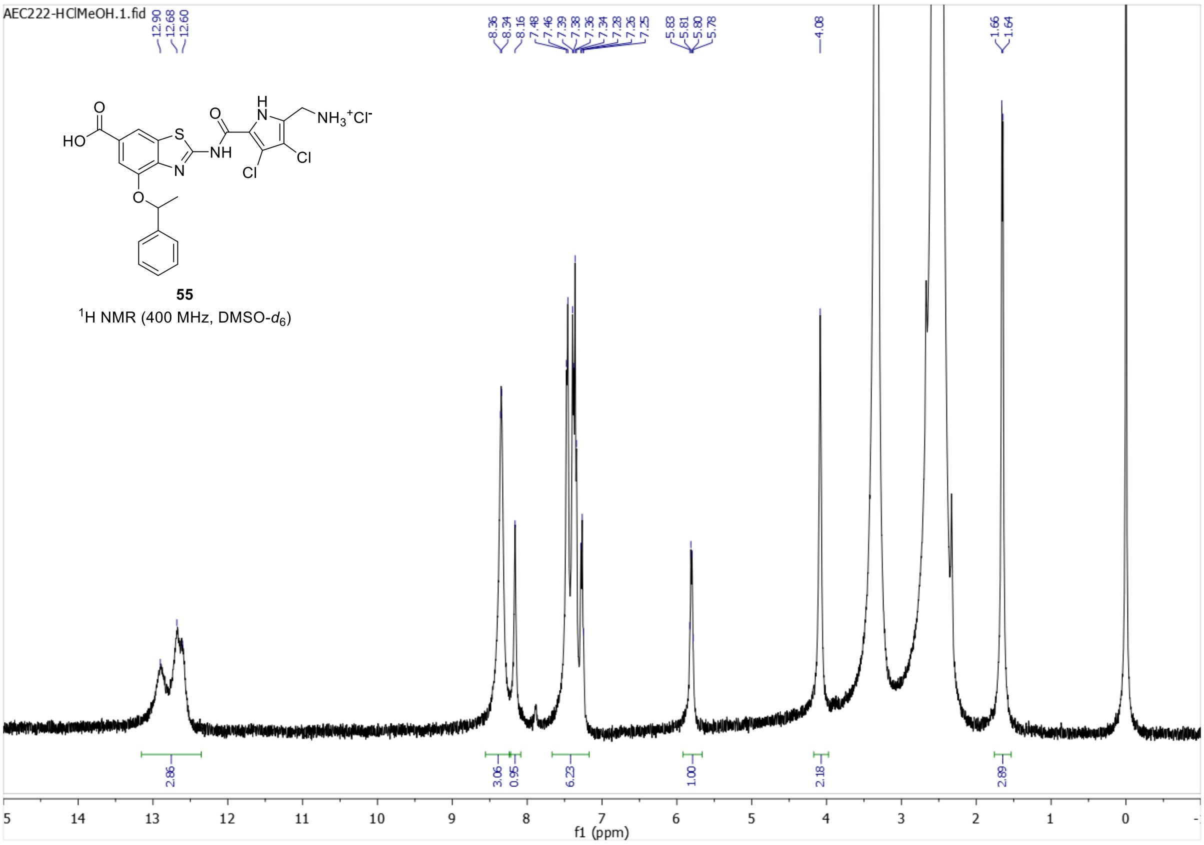

55

^{1}H NMR (400 MHz, DMSO-d_6)

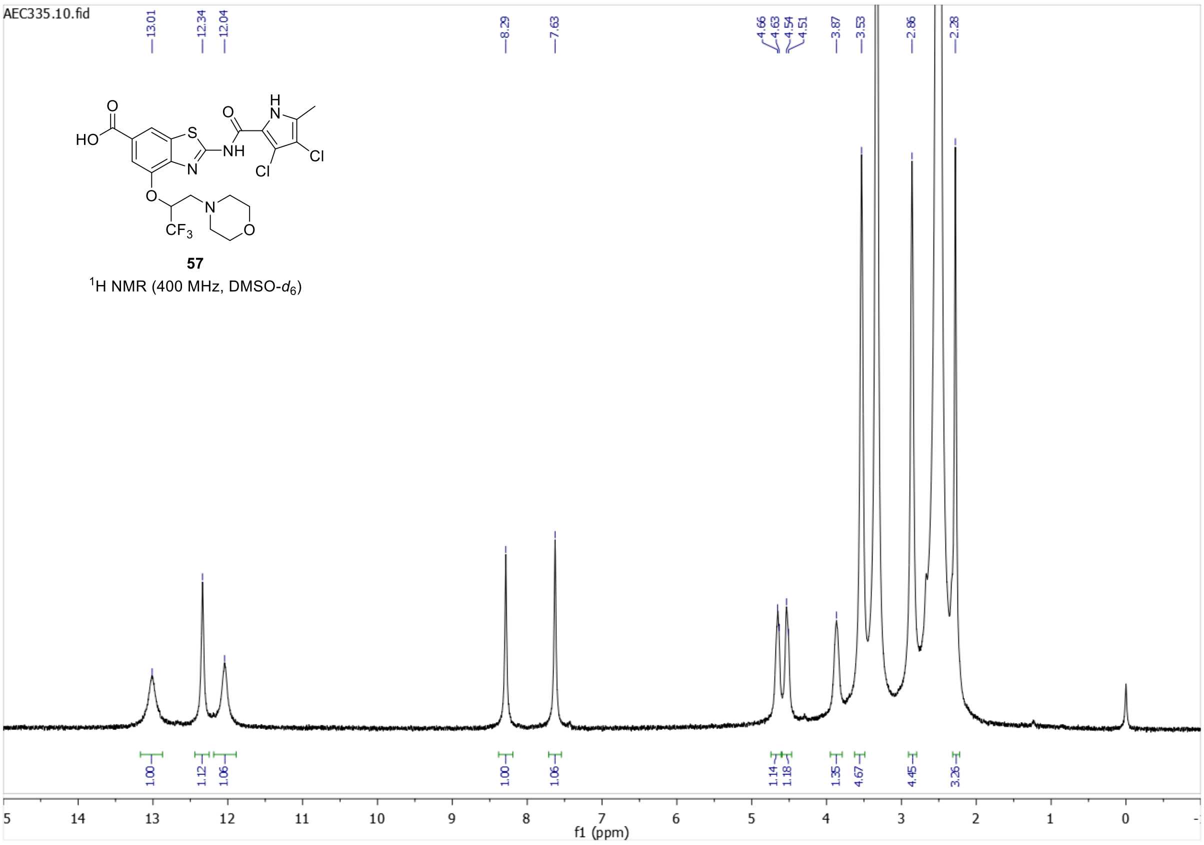
AEC335.10.fid
57
^{1}H NMR (400 MHz, DMSO-d_6)
f1 (ppm)

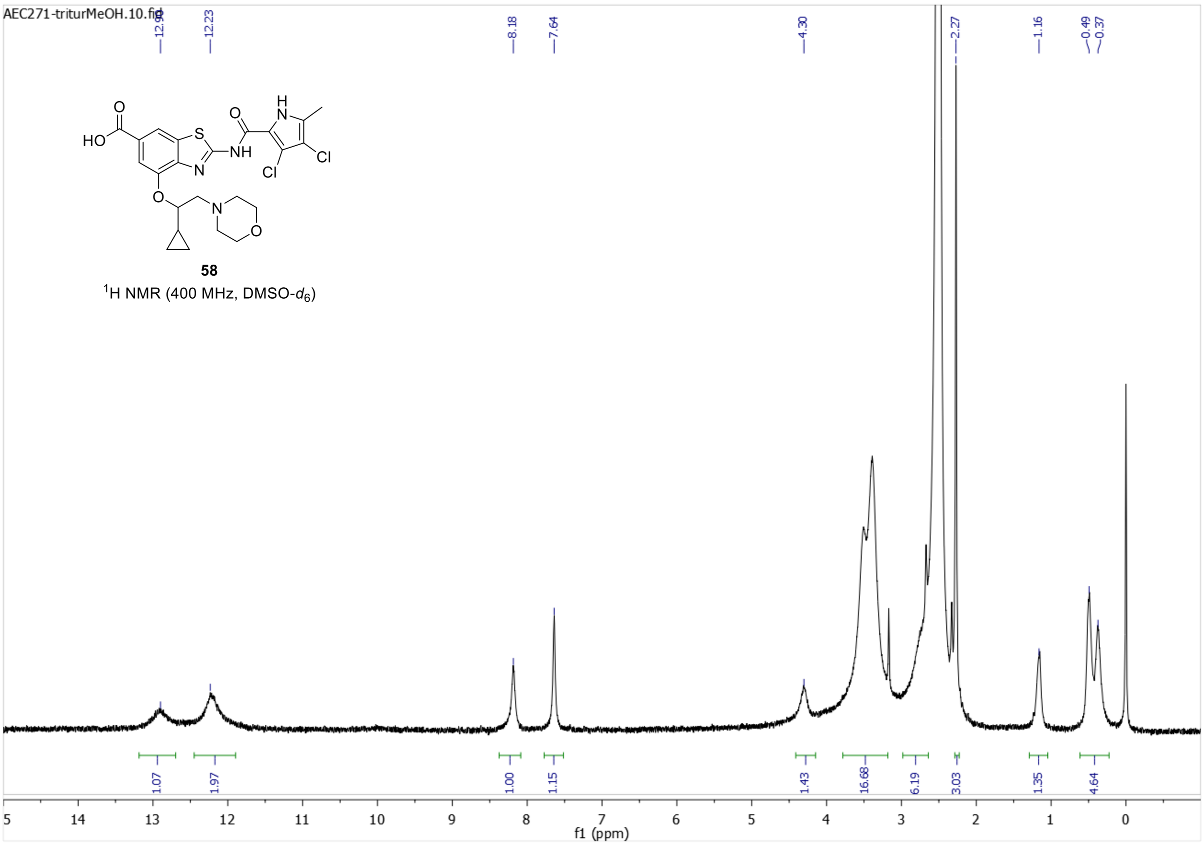
AEC271-triturMeOH.10.fid
58
^{1}H NMR (400 MHz, DMSO-d_6)
f1 (ppm)

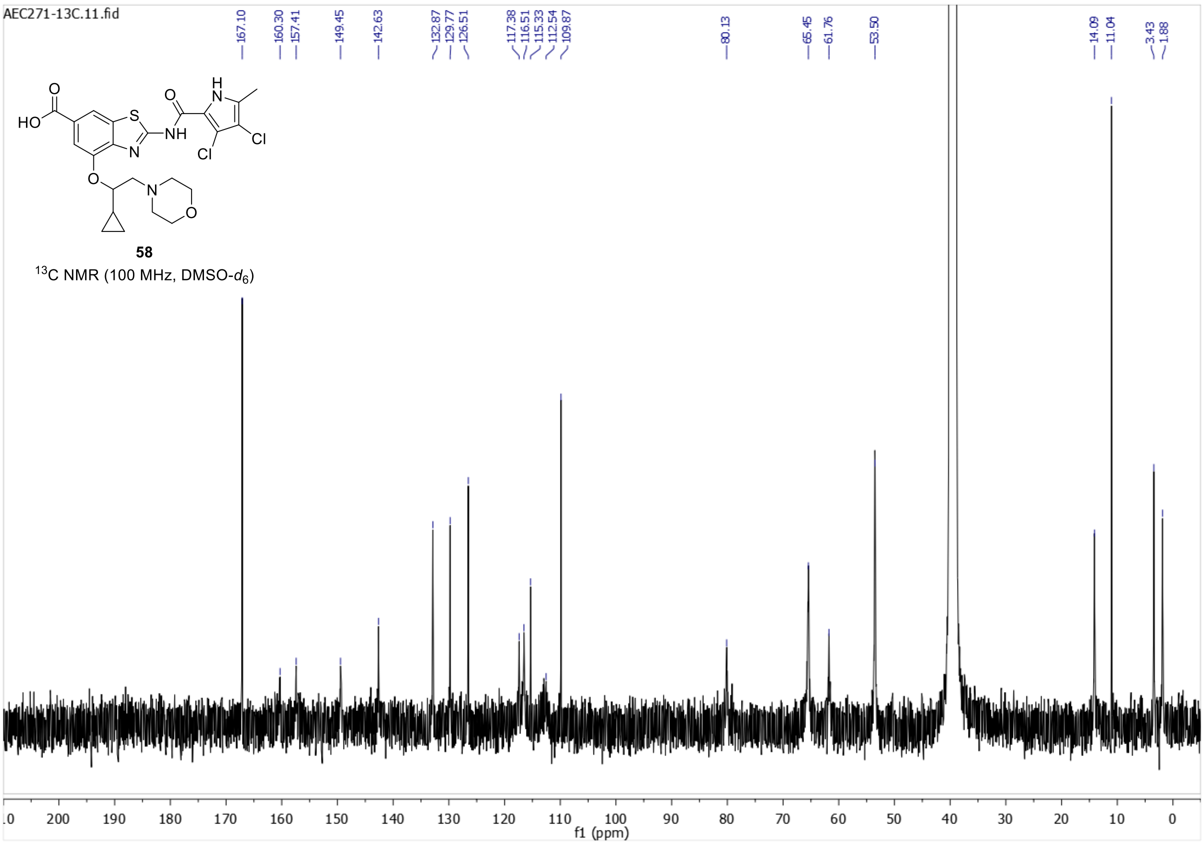

58

^{13}C NMR (100 MHz, DMSO-d_6)

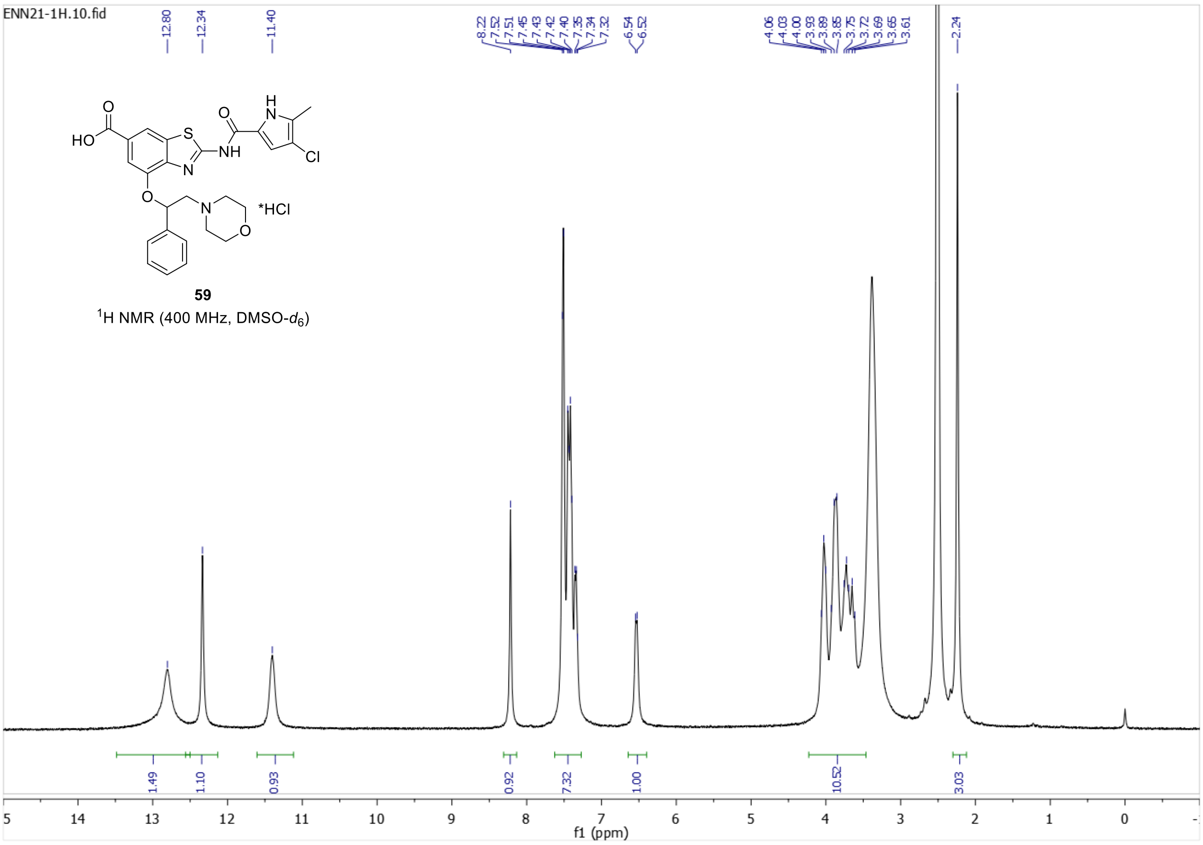

59

^{1}H NMR (400 MHz, DMSO-d_6)

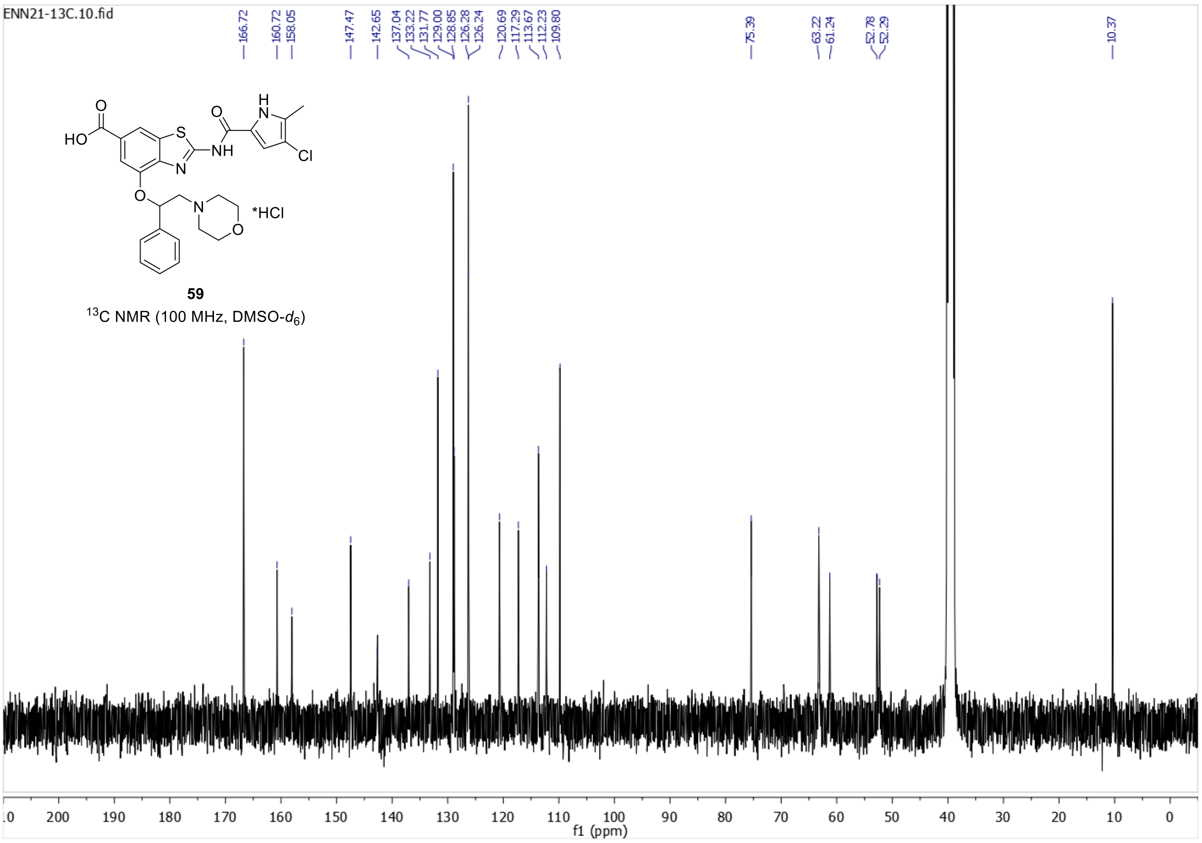
ENN21-13C.10.fid
166.72
160.72
158.05
147.47
142.65
137.04
133.22
131.77
129.00
128.85
126.28
126.24
120.69
117.29
113.67
112.23
109.80
75.39
63.22
61.24
52.78
52.29
10.37
HO
O
S
N
NH
O
H
N
Cl
O
N
O
*HCl
59
13C NMR (100 MHz, DMSO-d6)
f1 (ppm)

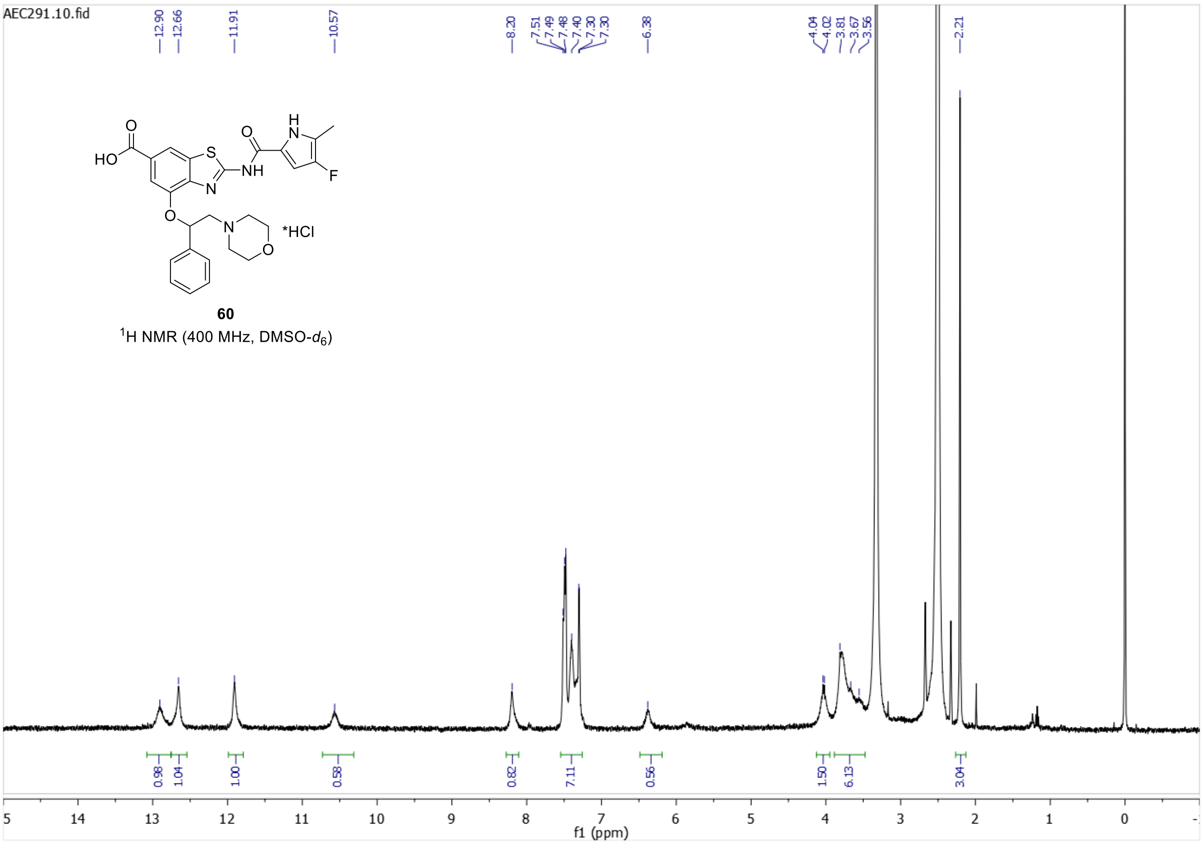
AEC291.10.fid
12.90
12.66
11.91
10.57
8.20
7.51
7.49
7.48
7.40
7.30
7.30
6.38
4.04
4.02
3.81
3.67
3.56
2.21
HO
O
S
N
NH
O
H
N
F
O
N
O
*HCl
60
1H NMR (400 MHz, DMSO-d6)
0.98
1.04
1.00
0.58
0.82
7.11
0.56
1.50
6.13
3.04
f1 (ppm)

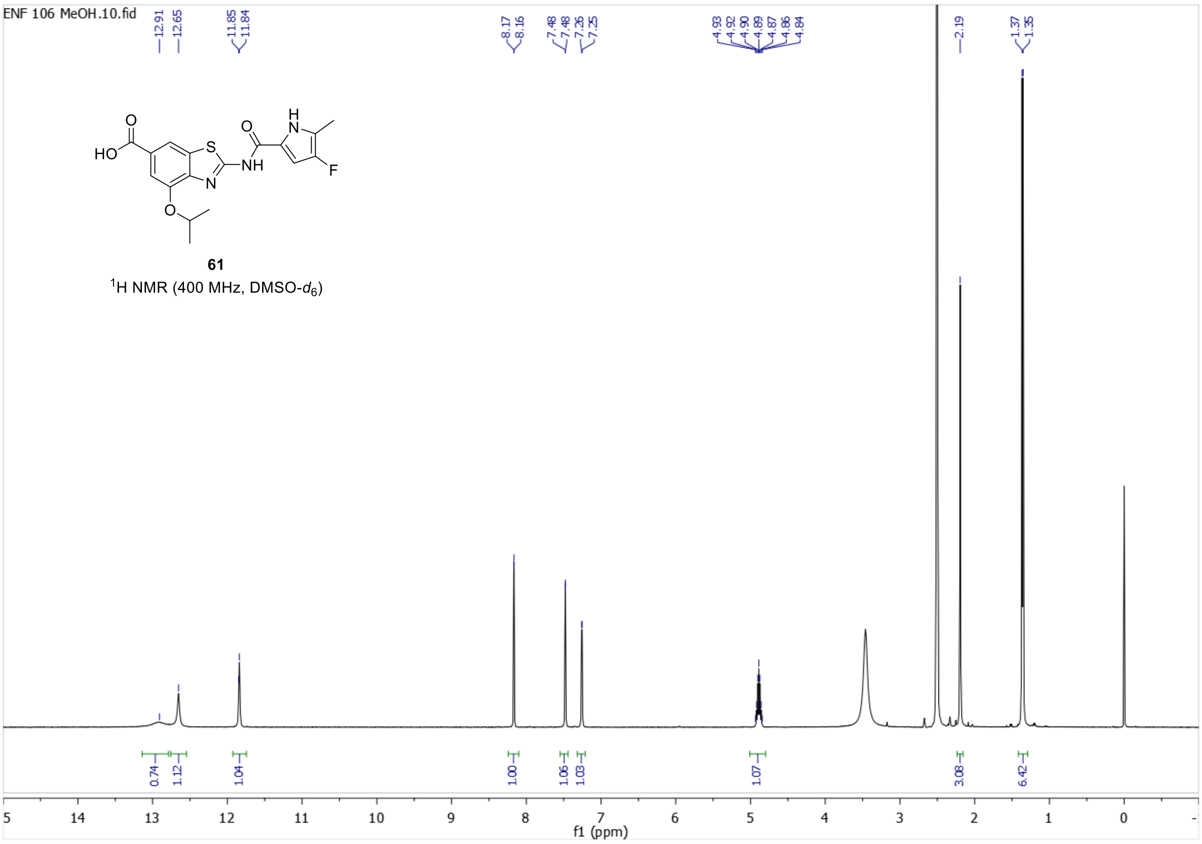
ENF 106 MeOH.10.fid
12.91
12.65
11.85
11.84
8.17
8.16
7.48
7.48
7.26
7.25
4.93
4.92
4.90
4.89
4.87
4.86
4.84
2.19
1.37
1.35
O
HO
S
N
NH
O
H
N
F
O
61
1H NMR (400 MHz, DMSO-d6)
0.74
1.12
1.04
1.00
1.06
1.03
1.07
3.08
6.42
f1 (ppm)

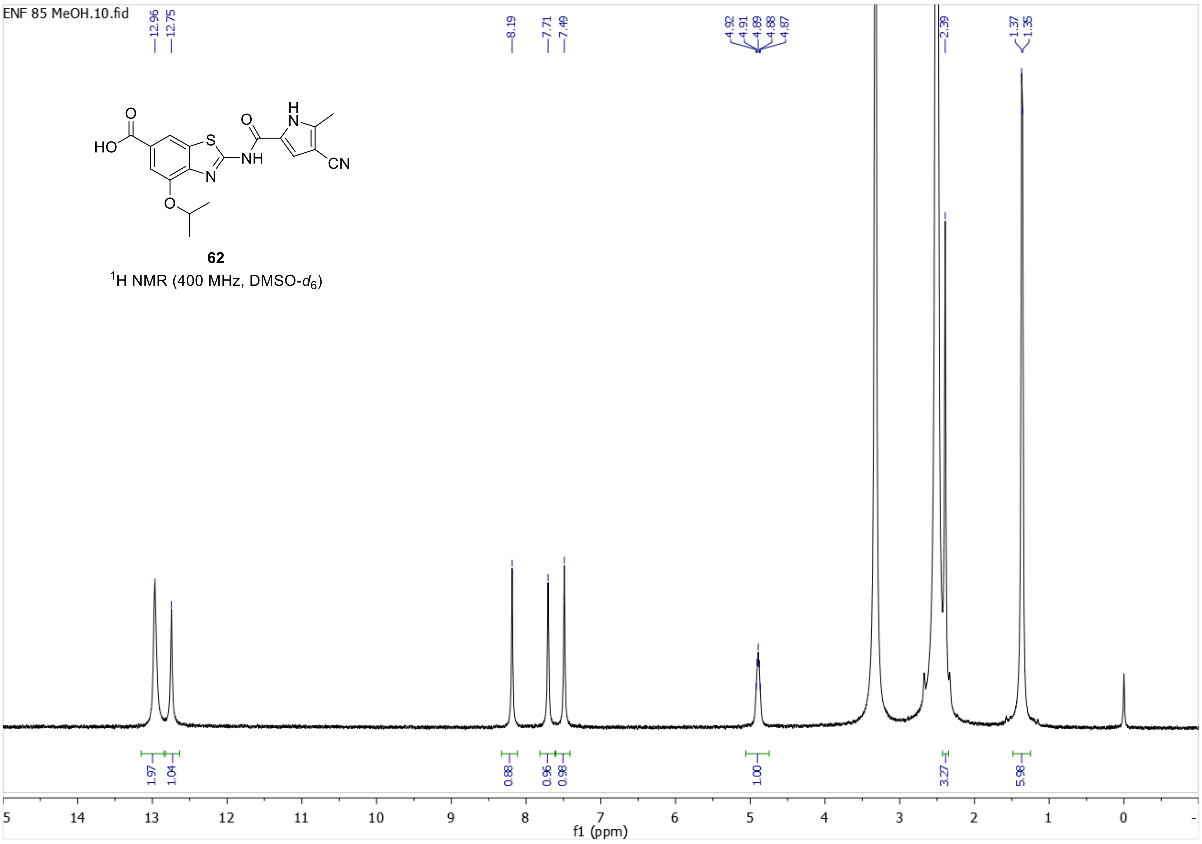
ENF 85 MeOH.10.fid
12.96
12.75
8.19
7.71
7.49
4.92
4.91
4.89
4.88
4.87
2.39
1.37
1.35
O
HO
S
N
NH
O
H
N
CN
O
62
1H NMR (400 MHz, DMSO-d6)
1.97
1.04
0.88
0.96
0.98
1.00
3.27
5.98
f1 (ppm)

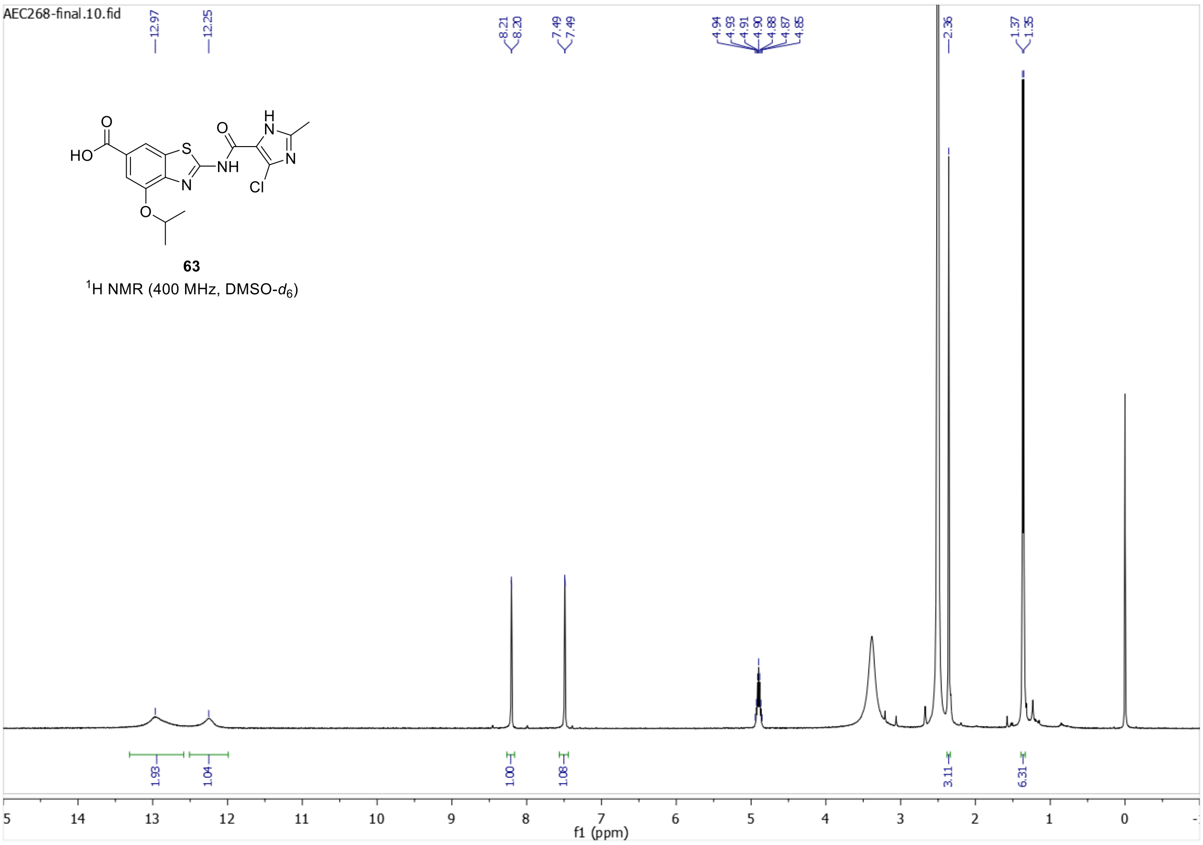
AEC268-final.10.fid
12.97
12.25
8.21
8.20
7.49
7.49
4.94
4.93
4.91
4.90
4.88
4.87
4.85
2.36
1.37
1.35
O
HO
S
N
NH
O
H
N
N
Cl
63
1H NMR (400 MHz, DMSO-d6)
1.93
1.04
1.00
1.08
3.11
6.31
f1 (ppm)

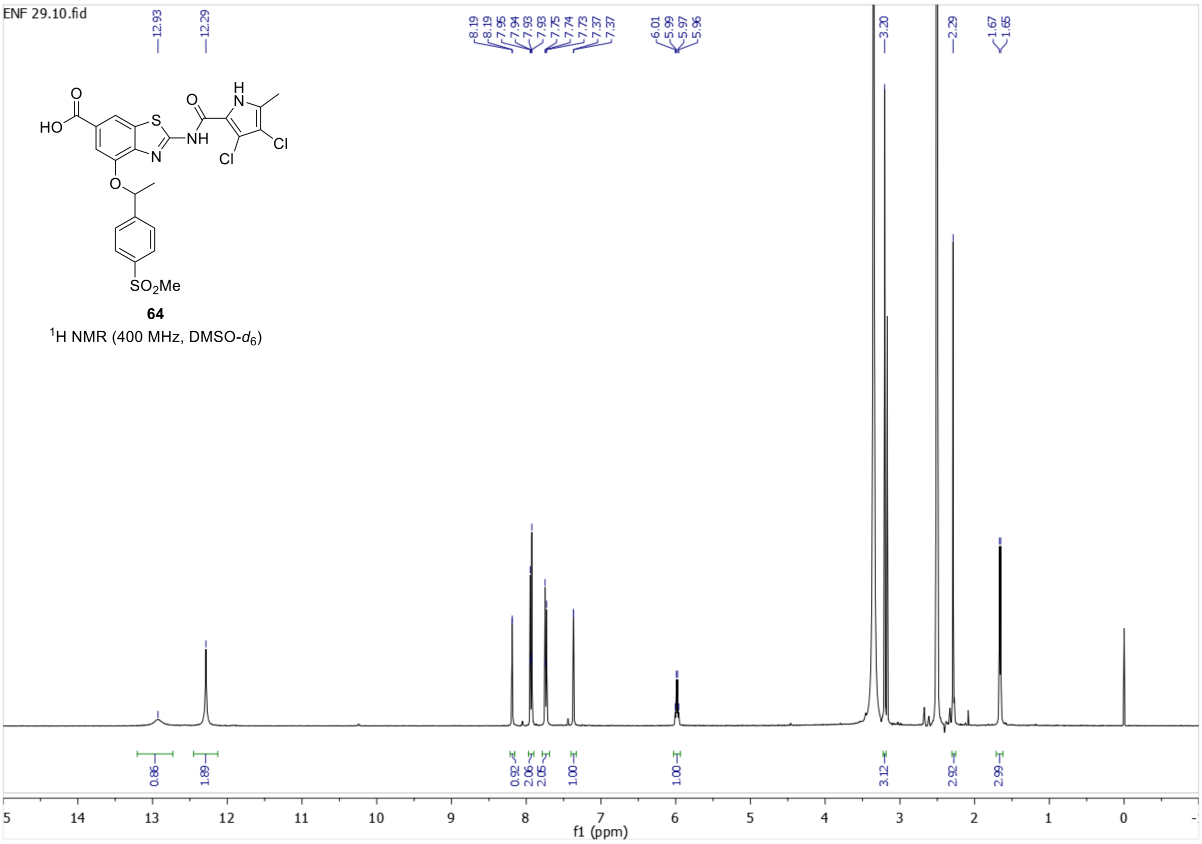
ENF 29.10.fid
12.93
12.29
8.19
8.19
7.95
7.94
7.93
7.93
7.75
7.74
7.73
7.37
7.37
6.01
5.99
5.97
5.96
3.20
2.29
1.67
1.65
O
HO
S
N
NH
O
H
N
Cl
Cl
SO2Me
64
1H NMR (400 MHz, DMSO-d6)
0.86
1.89
0.92
2.06
2.05
1.00
1.00
3.12
2.92
2.99
f1 (ppm)

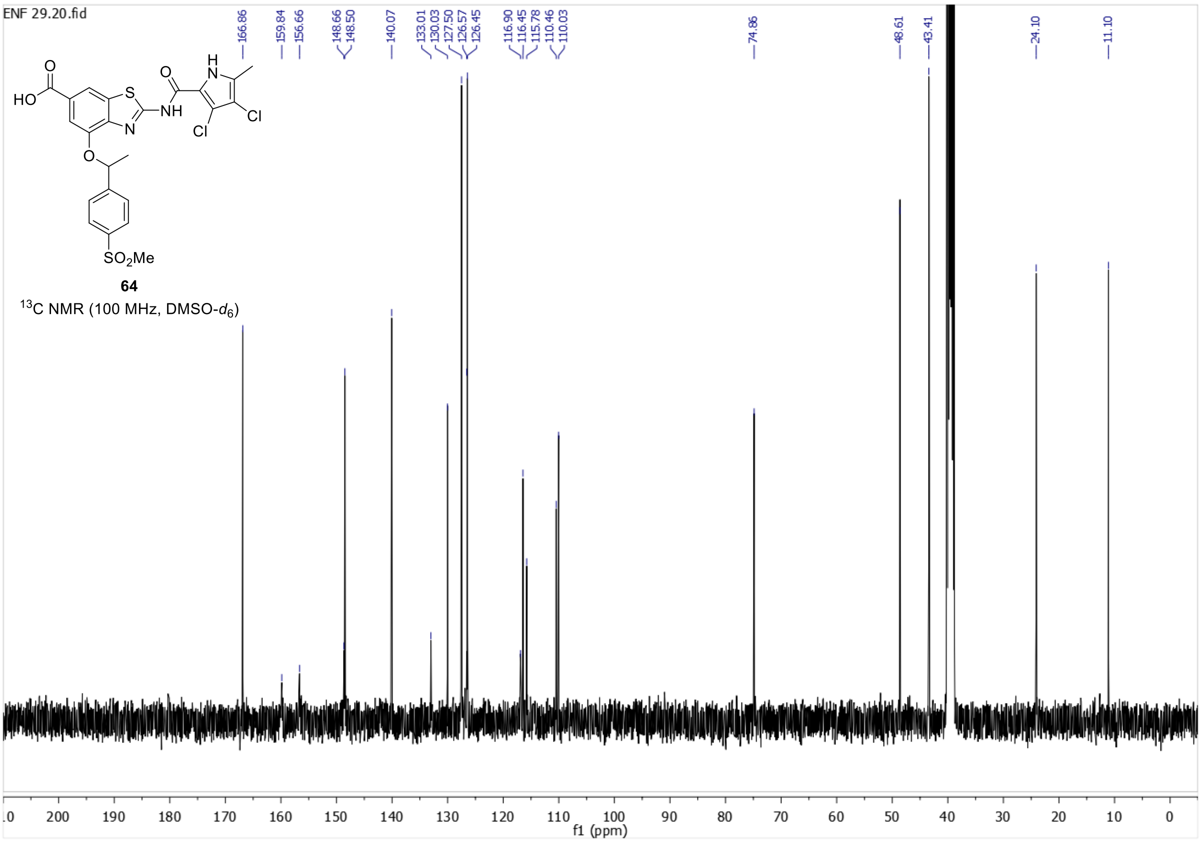
ENF 29.20.fid
166.86
159.84
156.66
148.66
148.50
140.07
133.01
130.03
127.50
126.57
126.45
116.90
116.45
115.78
110.46
110.03
74.86
48.61
43.41
24.10
11.10
HO
O
S
N
NH
O
H
N
Cl
Cl
O
SO_2Me
64
^{13}C NMR (100 MHz, DMSO-d_6)
f1 (ppm)

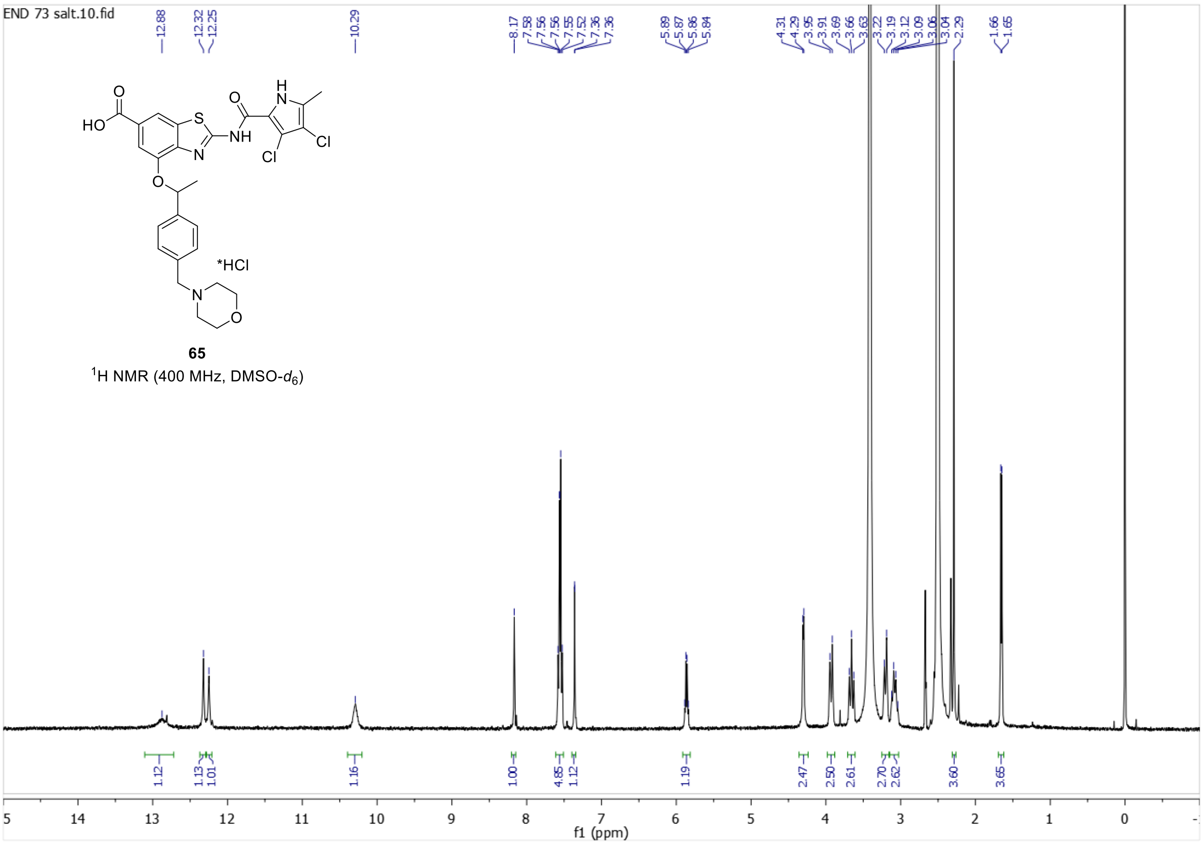
END 73 salt.10.fid
12.88
12.32
12.25
10.29
8.17
7.58
7.56
7.56
7.55
7.52
7.36
7.36
5.89
5.87
5.86
5.84
4.31
4.29
3.95
3.91
3.69
3.66
3.63
3.22
3.19
3.12
3.09
3.06
3.04
2.29
1.66
1.65
HO
O
S
N
NH
O
H
N
Cl
Cl
O
*HCl
N
O
65
^1H NMR (400 MHz, DMSO-d_6)
1.12
1.13
1.01
1.16
1.00
4.85
1.12
1.19
2.47
2.50
2.61
2.70
2.62
3.60
3.65
f1 (ppm)

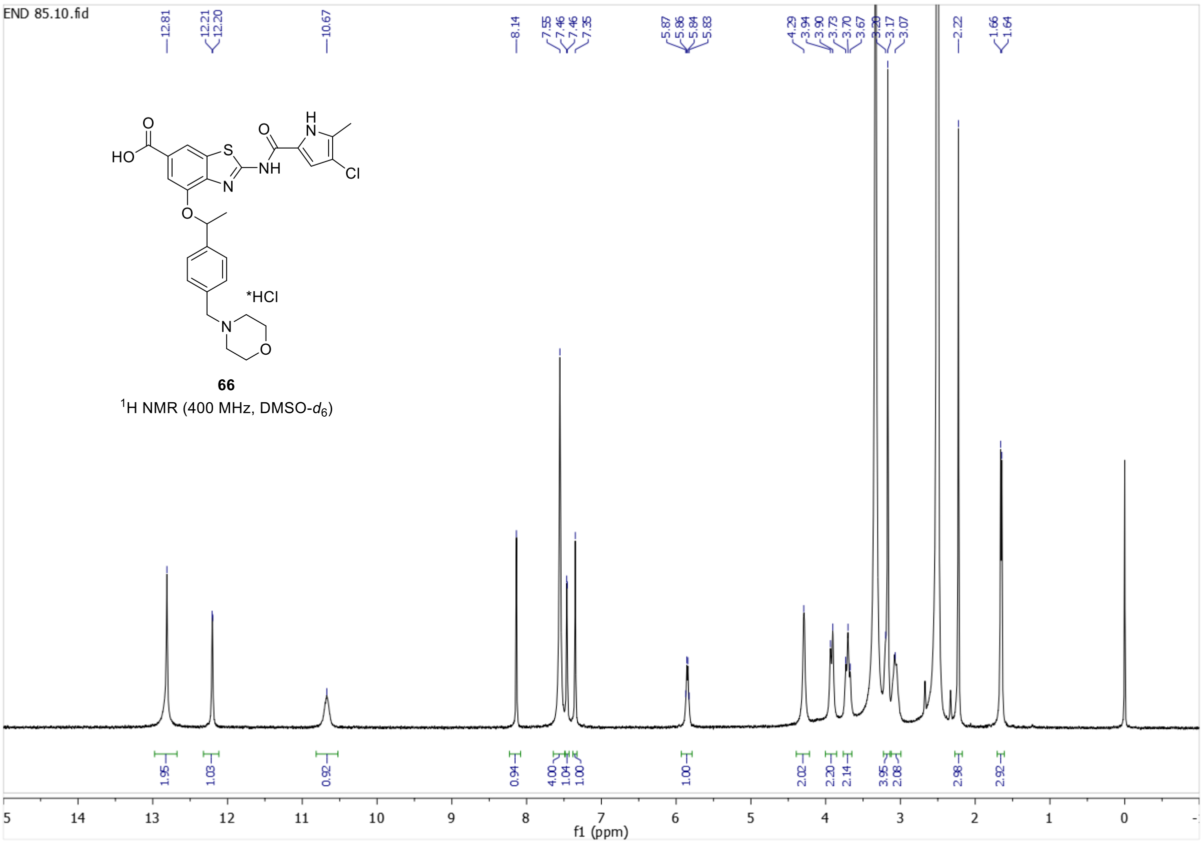
END 85.10.fid
*HCl
66
1H NMR (400 MHz, DMSO-d6)
f1 (ppm)

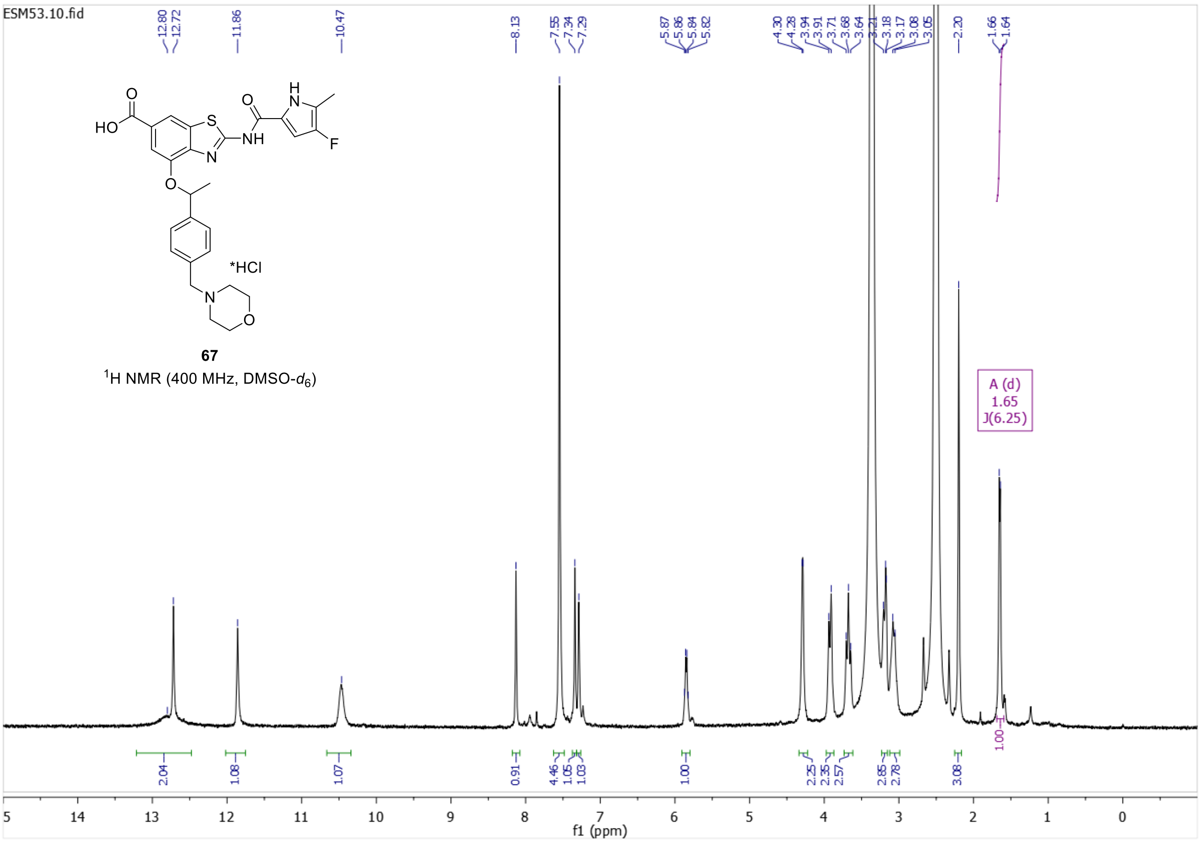
ESM53.10.fid
*HCl
67
1H NMR (400 MHz, DMSO-d6)
A (d)
1.65
J(6.25)
f1 (ppm)

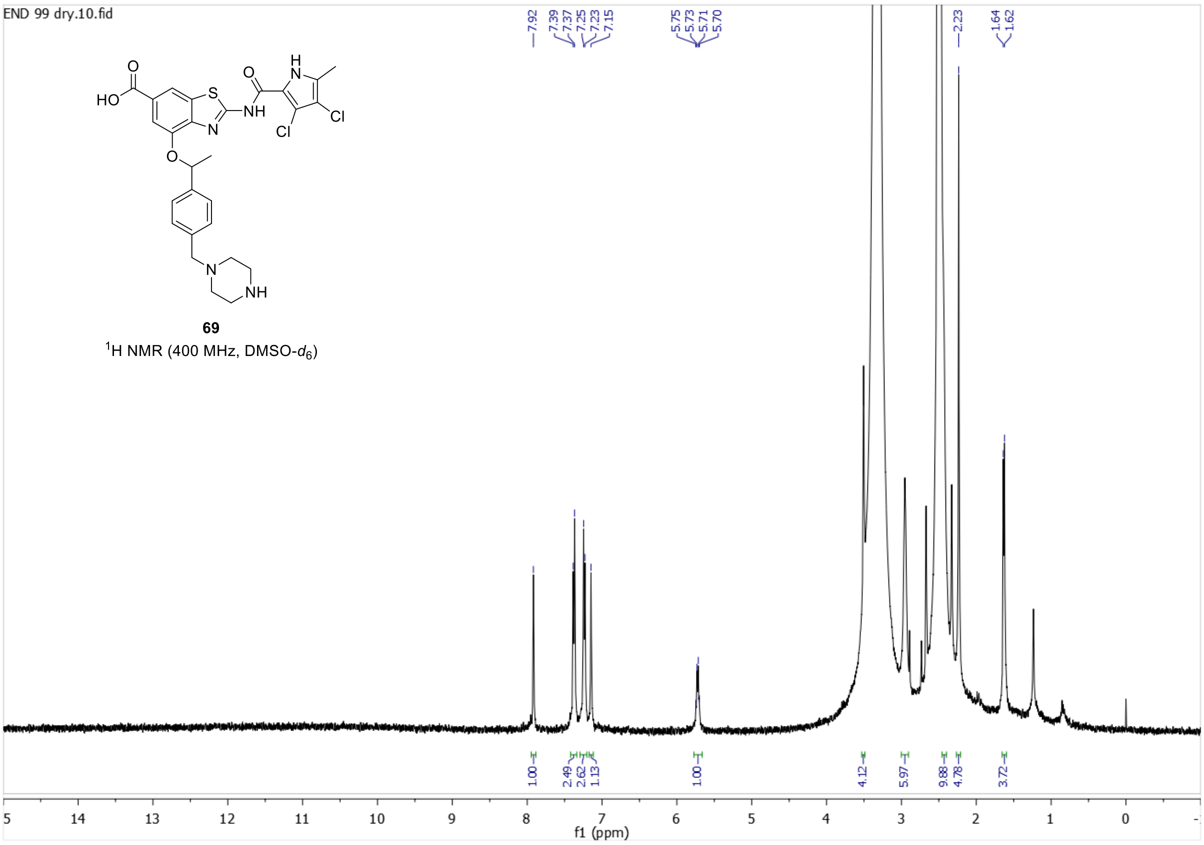
END 99 dry.10.fid
69
1H NMR (400 MHz, DMSO-d6)
f1 (ppm)

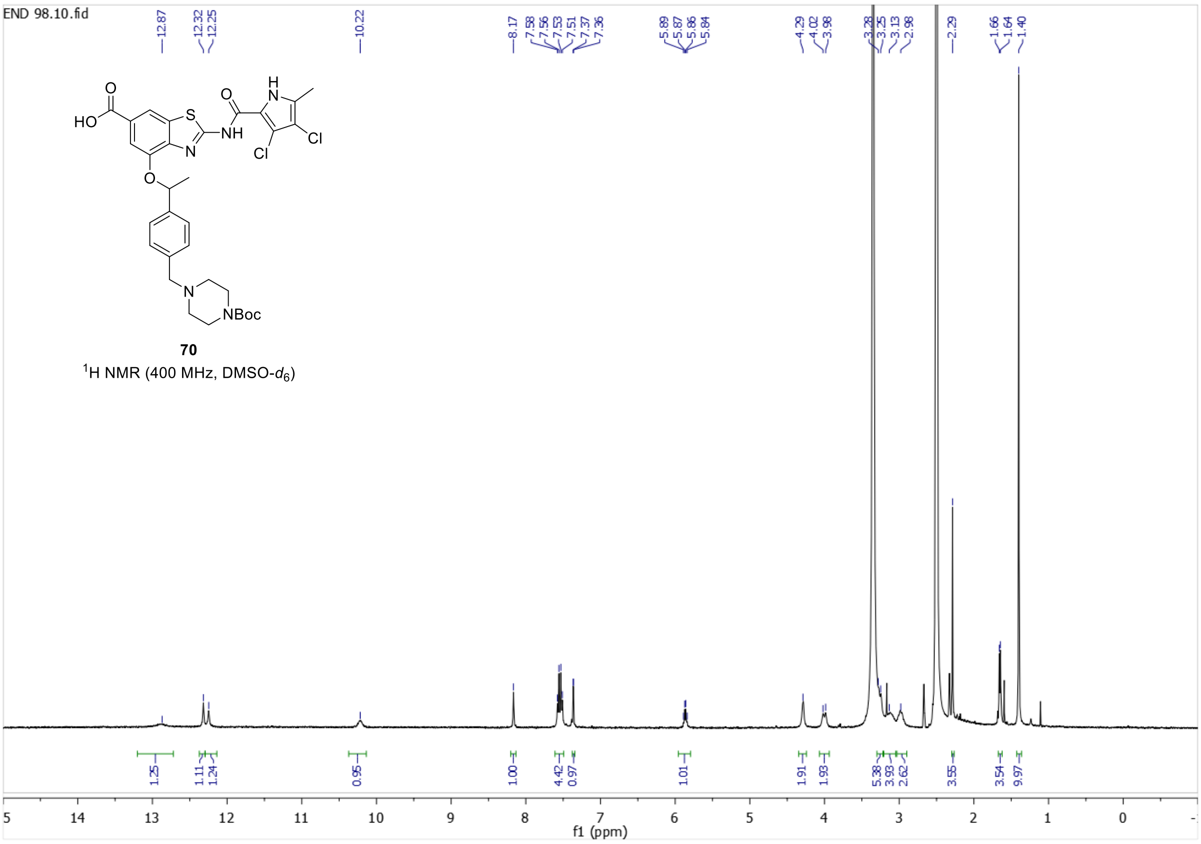
END 98.10.fid
70
1H NMR (400 MHz, DMSO-d6)
f1 (ppm)

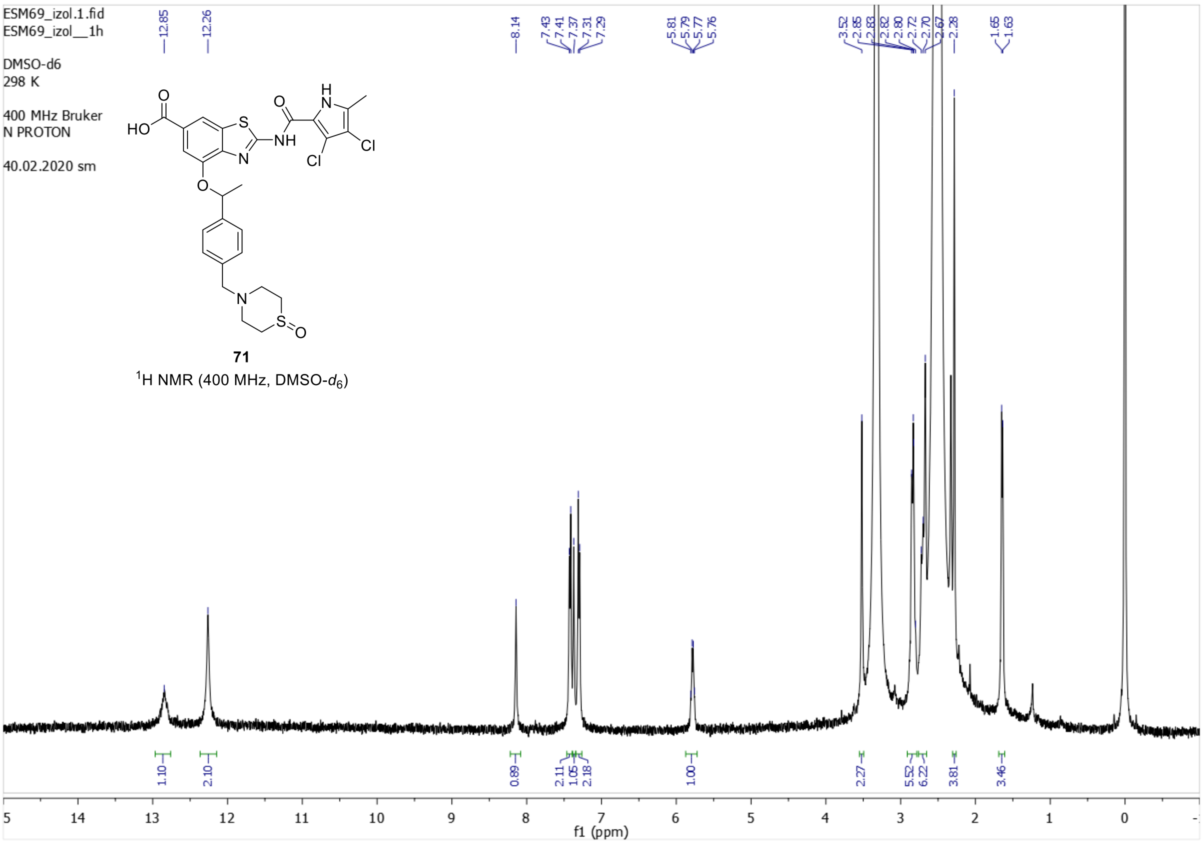

71

^{1}H NMR (400 MHz, DMSO-d_6)

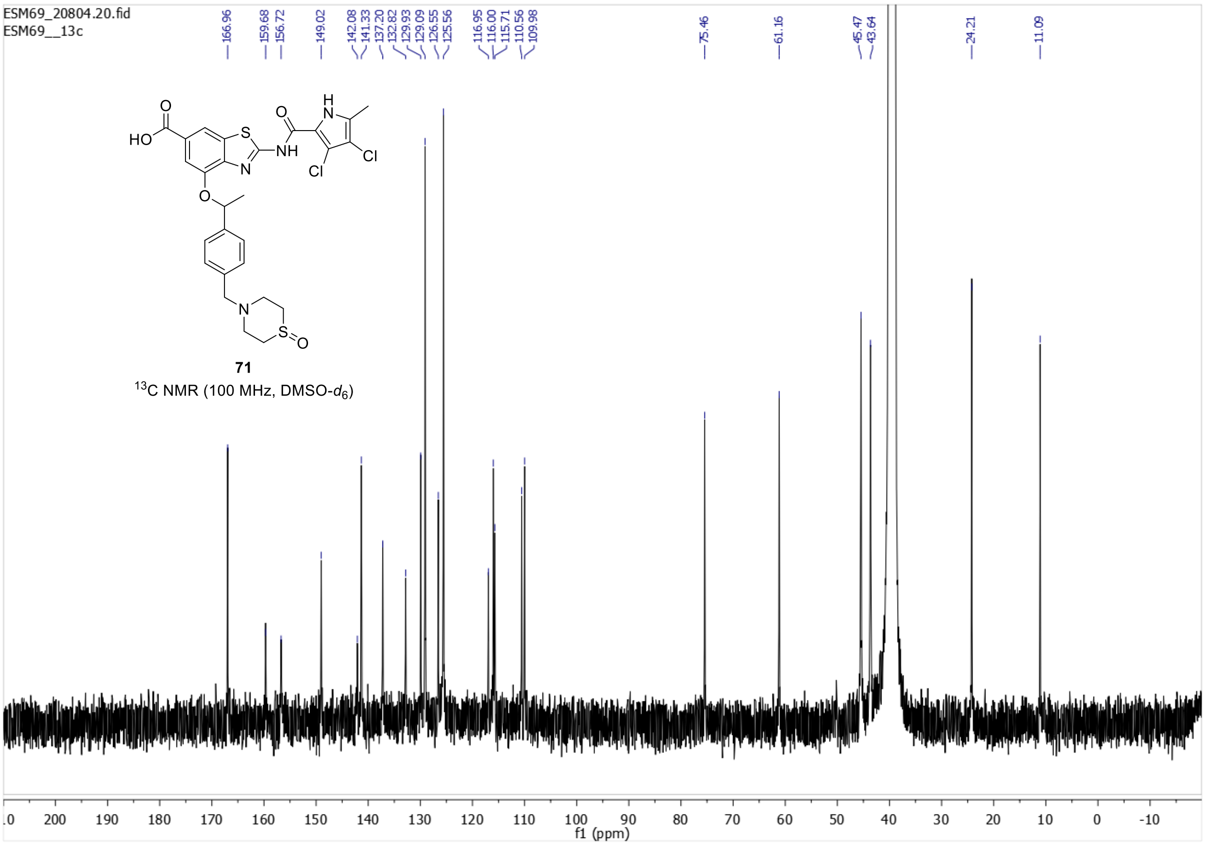

71

^{13}C NMR (100 MHz, DMSO-d_6)

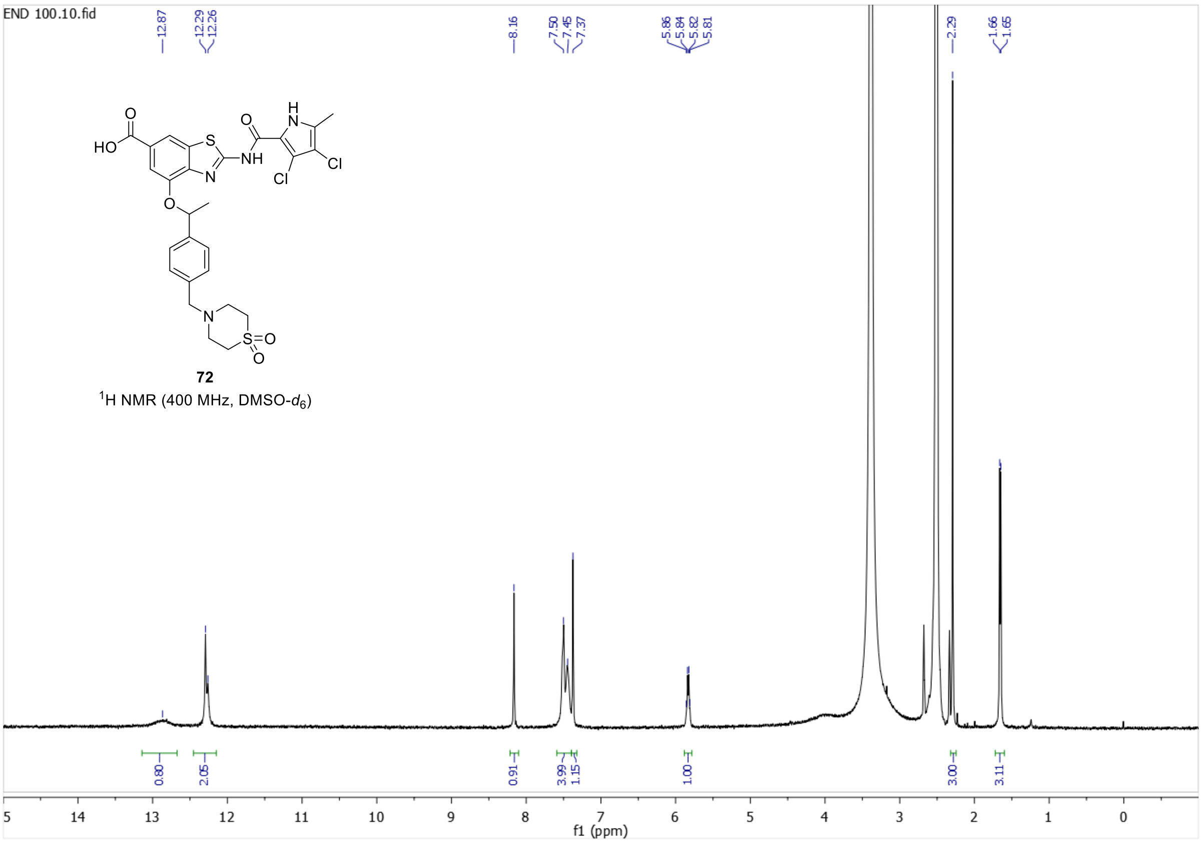
END 100.10.fid
72
^{1}H NMR (400 MHz, DMSO-d_6)
f1 (ppm)

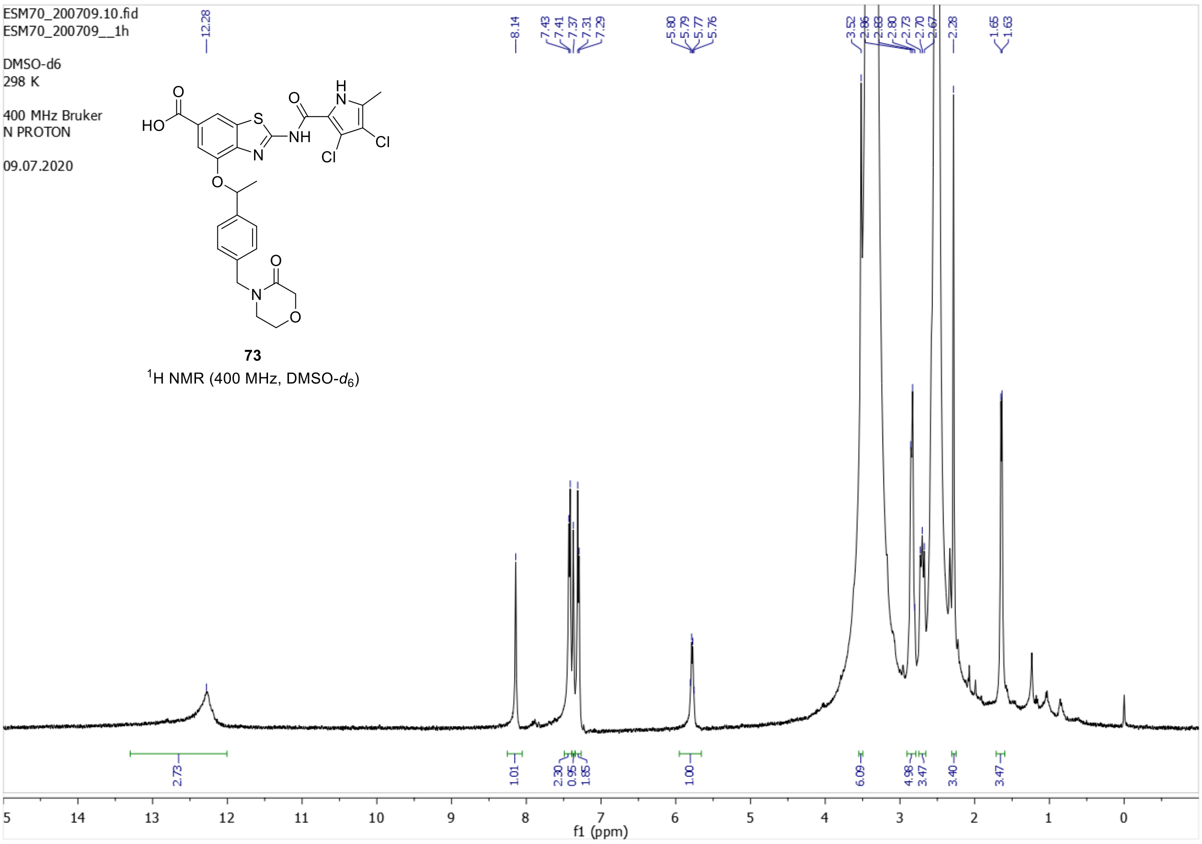
ESM70_200709.10.fid
ESM70_200709__1h
DMSO-d6
298 K
400 MHz Bruker
N PROTON
09.07.2020
73
^{1}H NMR (400 MHz, DMSO-d_6)
f1 (ppm)

HPLC traces

Compound 2:

Chromatogram

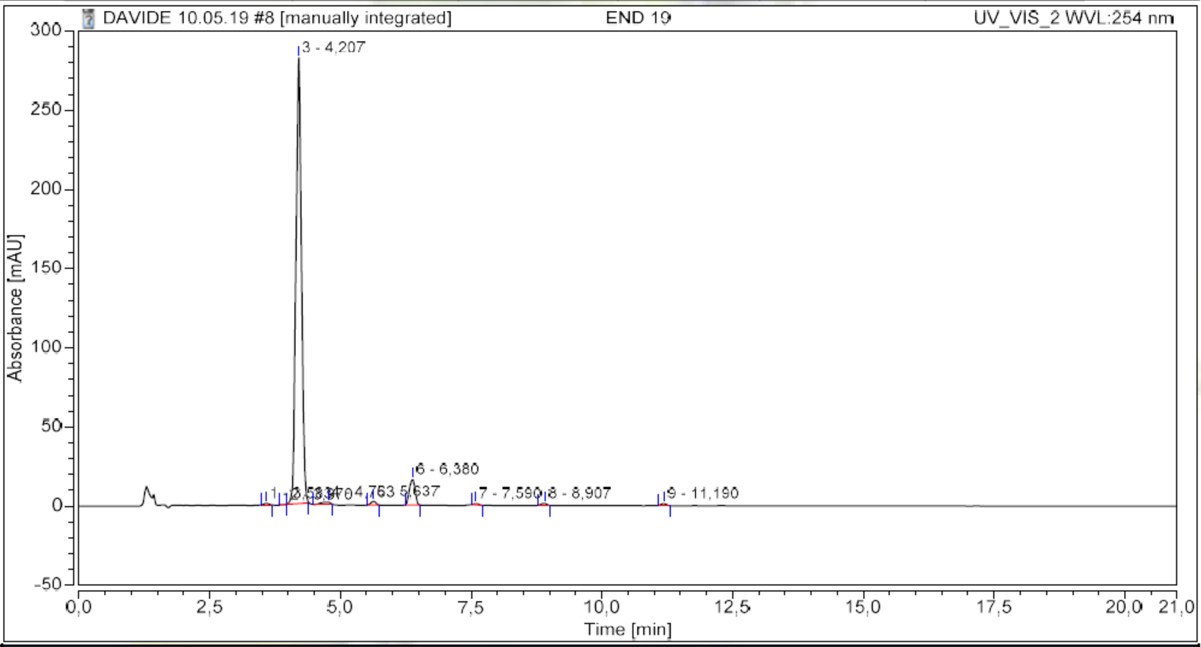

Integration Results

No.	Peak Name	Retention Time min	Area mAU*min	Height mAU	Relative Area %	Relative Height %	Amount n.a.
1		3,583	0,098	0,901	0,26	0,29	n.a.
2		3,970	0,006	0,000	0,02	0,00	n.a.
3		4,207	34,353	281,618	91,80	92,15	n.a.
4		4,763	0,250	1,376	0,67	0,45	n.a.
5		5,637	0,254	2,321	0,68	0,76	n.a.
6		6,380	2,053	16,177	5,49	5,29	n.a.
7		7,590	0,083	0,755	0,22	0,25	n.a.
8		8,907	0,154	1,178	0,41	0,39	n.a.
9		11,190	0,170	1,299	0,45	0,42	n.a.
**Total:**			**37,419**	**305,625**	**100,00**	**100,00**	

Compound 3:

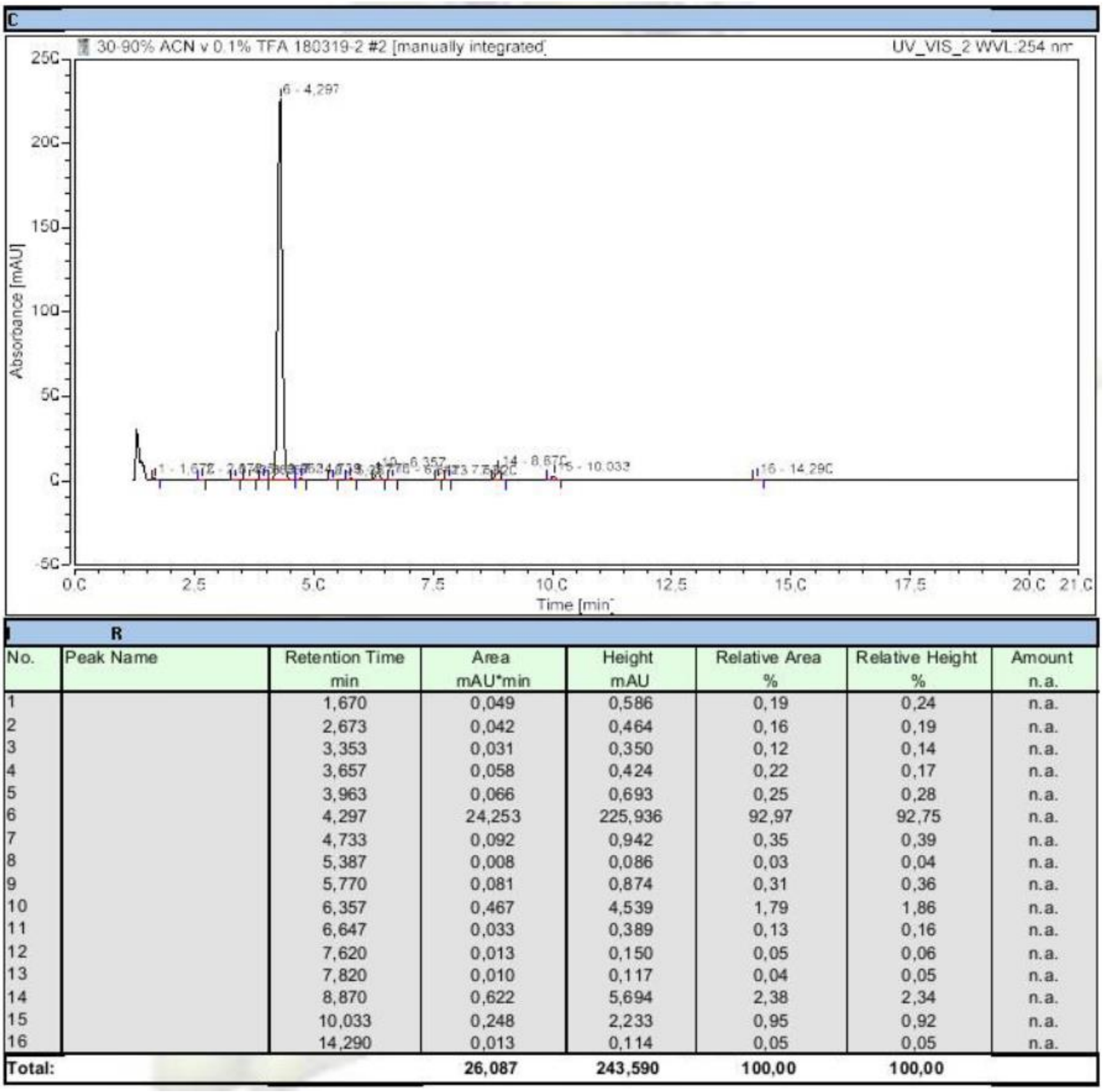

No.	Peak Name	Retention Time min	Area mAU*min	Height mAU	Relative Area %	Relative Height %	Amount n.a.
1		1,670	0,049	0,586	0,19	0,24	n.a.
2		2,673	0,042	0,464	0,16	0,19	n.a.
3		3,353	0,031	0,350	0,12	0,14	n.a.
4		3,657	0,058	0,424	0,22	0,17	n.a.
5		3,963	0,066	0,693	0,25	0,28	n.a.
6		4,297	24,253	225,936	92,97	92,75	n.a.
7		4,733	0,092	0,942	0,35	0,39	n.a.
8		5,387	0,008	0,086	0,03	0,04	n.a.
9		5,770	0,081	0,874	0,31	0,36	n.a.
10		6,357	0,467	4,539	1,79	1,86	n.a.
11		6,647	0,033	0,389	0,13	0,16	n.a.
12		7,620	0,013	0,150	0,05	0,06	n.a.
13		7,820	0,010	0,117	0,04	0,05	n.a.
14		8,870	0,622	5,694	2,38	2,34	n.a.
15		10,033	0,248	2,233	0,95	0,92	n.a.
16		14,290	0,013	0,114	0,05	0,05	n.a.
**Total:**			**26,087**	**243,590**	**100,00**	**100,00**	

Compound 4:

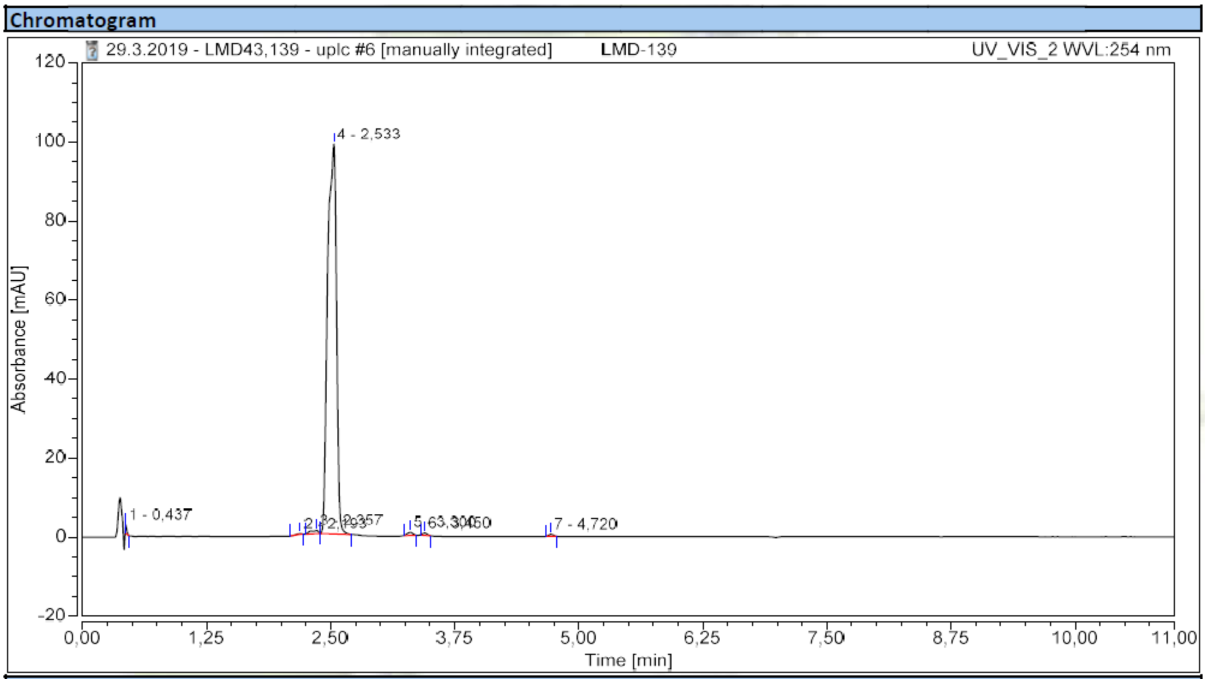

Integration Results

No.	Peak Name	Retention Time min	Area mAU*min	Height mAU	Relative Area %	Relative Height %	Amount n.a.
1		0,437	0,030	2,105	0,29	2,03	n.a.
2		2,193	0,014	0,156	0,13	0,15	n.a.
3		2,357	0,077	0,782	0,74	0,75	n.a.
4		2,533	10,140	98,582	97,72	95,19	n.a.
5		3,300	0,053	0,789	0,51	0,76	n.a.
6		3,450	0,034	0,617	0,33	0,60	n.a.
7		4,720	0,028	0,531	0,27	0,51	n.a.
**Total:**			**10,377**	**103,563**	**100,00**	**100,00**	

Compound 5:

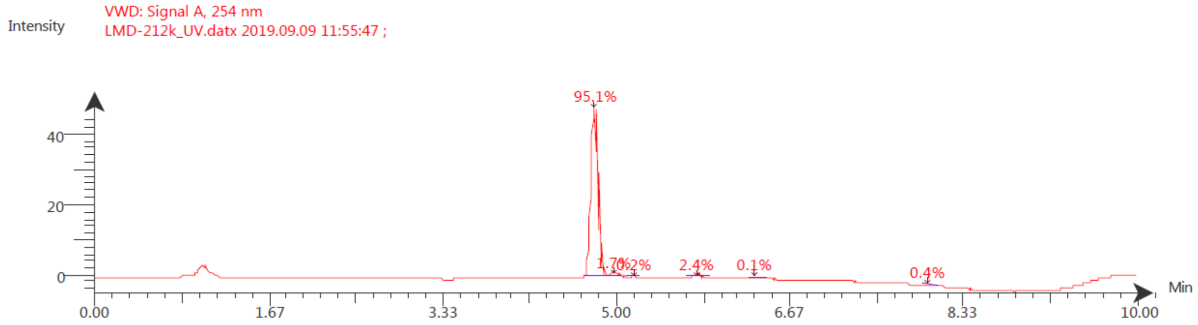

Compound 6:

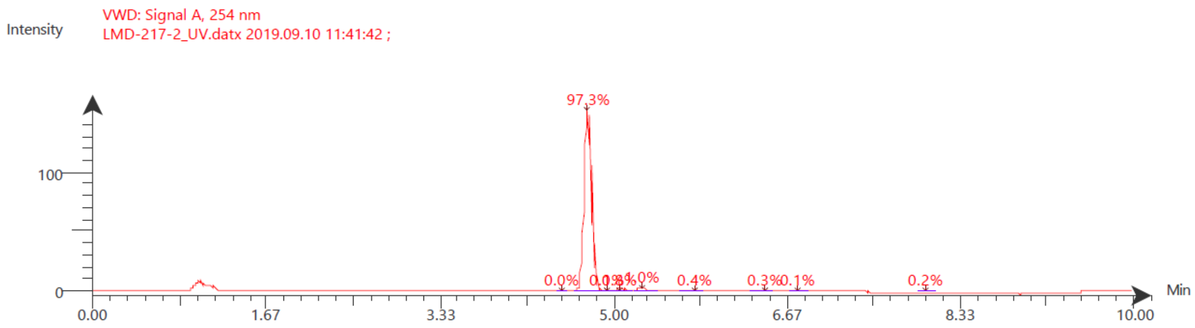

Compound 8:

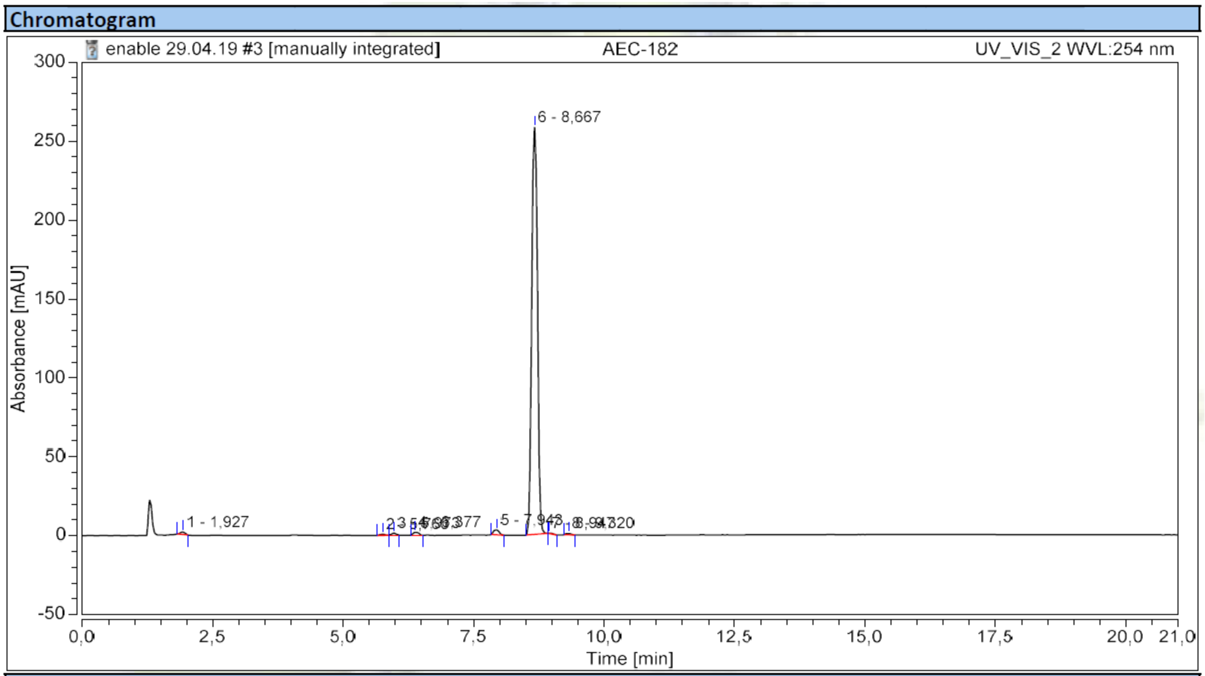

Integration Results

No.	Peak Name	Retention Time min	Area mAU*min	Height mAU	Relative Area %	Relative Height %	Amount n.a.
1		1,927	0,173	1,598	0,51	0,60	n.a.
2		5,763	0,077	0,653	0,23	0,24	n.a.
3		5,973	0,119	1,261	0,35	0,47	n.a.
4		6,377	0,231	1,785	0,68	0,67	n.a.
5		7,943	0,389	3,076	1,15	1,15	n.a.
6		8,667	32,691	257,931	96,69	96,50	n.a.
7		8,947	0,032	0,176	0,10	0,07	n.a.
8		9,320	0,096	0,803	0,28	0,30	n.a.
**Total:**			**33,809**	**267,283**	**100,00**	**100,00**	

Compound 9:

Chromatogram

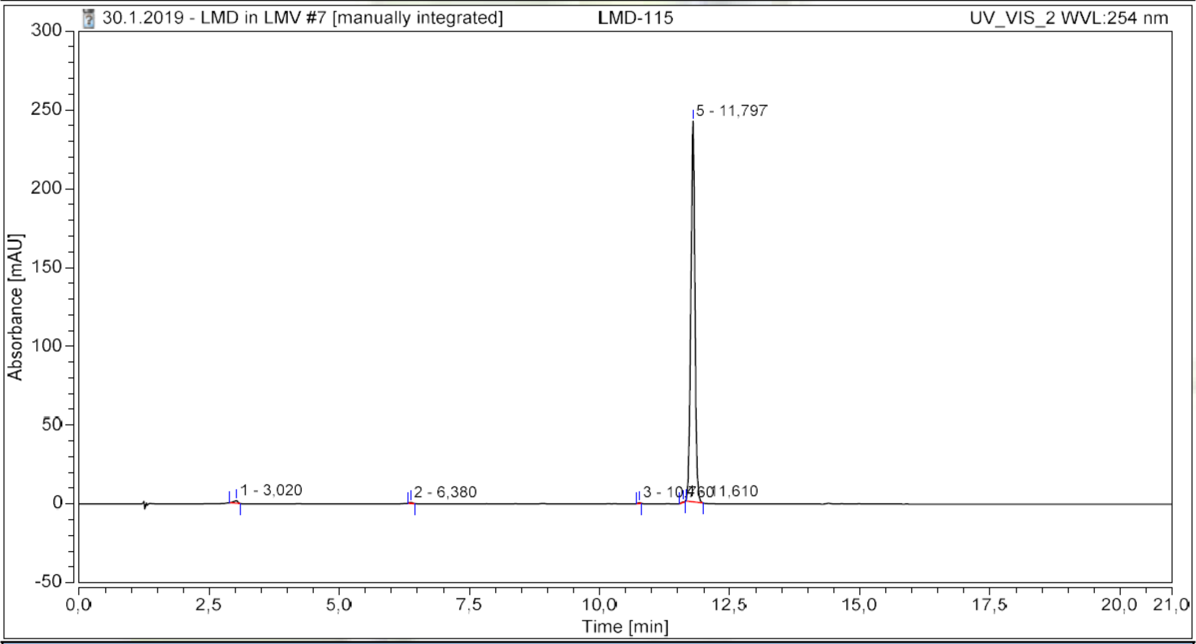

Integration Results

No.	Peak Name	Retention Time min	Area mAU*min	Height mAU	Relative Area %	Relative Height %	Amount n.a.
1		3,020	0,170	1,754	0,77	0,72	n.a.
2		6,380	0,057	0,841	0,26	0,34	n.a.
3		10,760	0,040	0,673	0,18	0,27	n.a.
4		11,610	0,025	0,466	0,12	0,19	n.a.
5		11,797	21,718	241,499	98,67	98,48	n.a.
**Total:**			**22,010**	**245,234**	**100,00**	**100,00**	

Compound 10:

Chromatogram

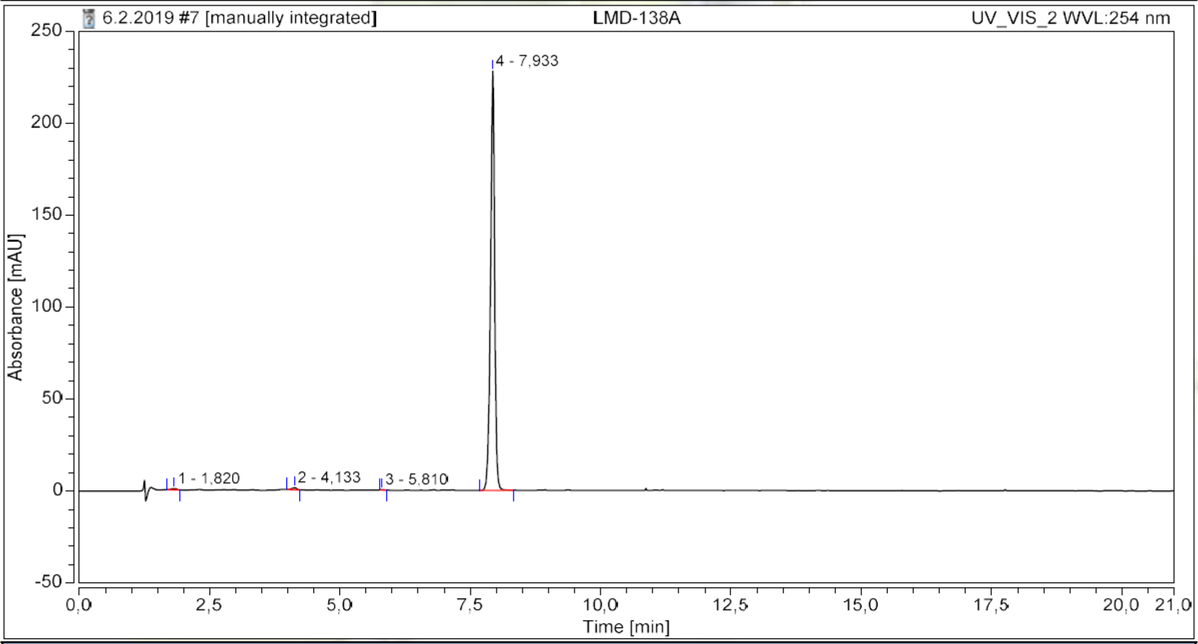

Integration Results

No.	Peak Name	Retention Time min	Area mAU*min	Height mAU	Relative Area %	Relative Height %	Amount n.a.
1		1,820	0,088	0,753	0,42	0,33	n.a.
2		4,133	0,108	1,050	0,52	0,46	n.a.
3		5,810	0,013	0,177	0,06	0,08	n.a.
4		7,933	20,792	227,984	99,00	99,14	n.a.
**Total:**			**21,001**	**229,964**	**100,00**	**100,00**	

Compound 11:

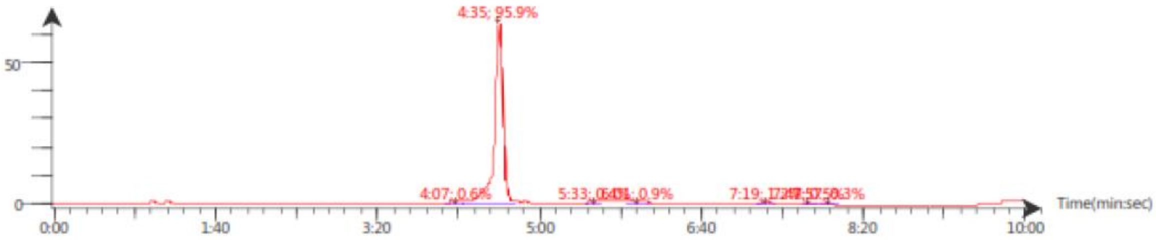

Time (Peak Maximum M:S/Minutes)	Maximum Intensity (c/s)	Time (Peak Centroid M:S/Minutes)	Peak Area	% Peak Area	Peak Resolution	Label
4:07.36	3.9E-1	4:07.74	2.3E0	0.6	11.2	
4:34.96	6.4E1	4:33.89	3.9E2	95.9	5.0	
5:33.36	3.4E-1	5:33.66	1.5E0	0.4	4.5	
5:42.56	2.8E-1	5:42.76	1.1E0	0.3	3.9	
6:00.96	4.5E-1	6:00.42	3.7E0	0.9	8.0	
7:18.96	9.1E-1	7:19.14	4.8E0	1.2	5.0	
7:46.56	3.7E-1	7:46.32	1.9E0	0.5	5.3	
7:57.36	2.4E-1	7:57.69	1.4E0	0.3	5.7	

Compound 12:

Chromatogram

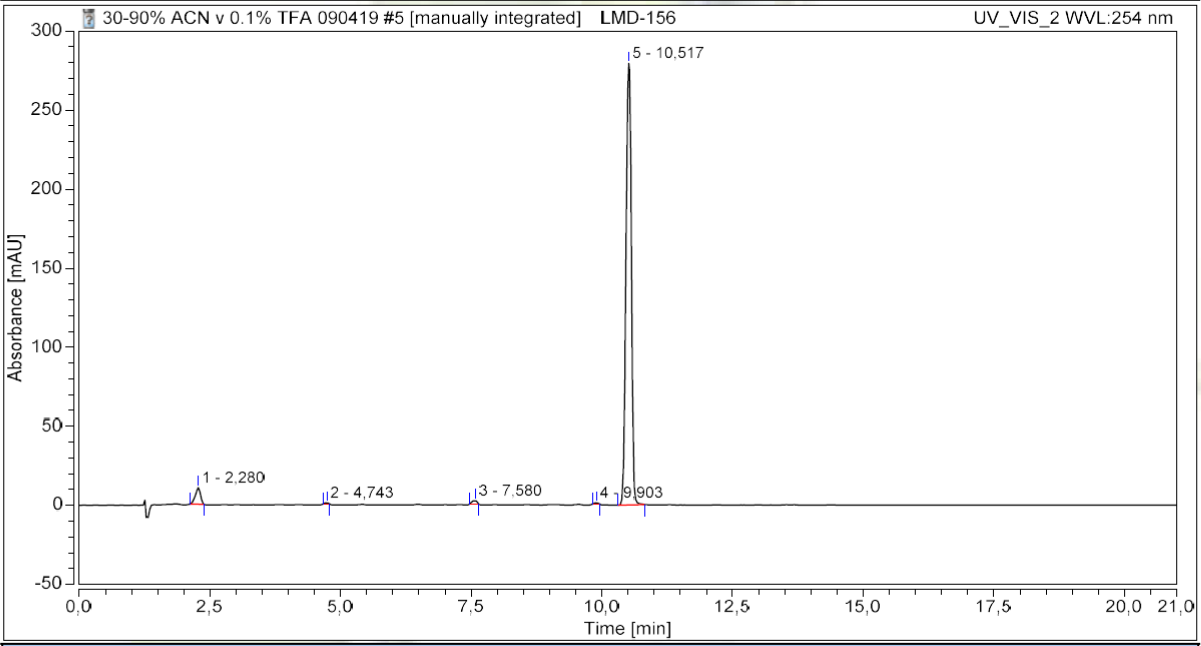

Integration Results

No.	Peak Name	Retention Time min	Area mAU*min	Height mAU	Relative Area %	Relative Height %	Amount n.a.
1		2,280	1,207	10,699	3,44	3,64	n.a.
2		4,743	0,068	0,779	0,19	0,27	n.a.
3		7,580	0,272	2,355	0,78	0,80	n.a.
4		9,903	0,076	0,830	0,22	0,28	n.a.
5		10,517	33,421	279,232	95,37	95,01	n.a.
**Total:**			**35,043**	**293,895**	**100,00**	**100,00**	

Compound 14:

Chromatogram

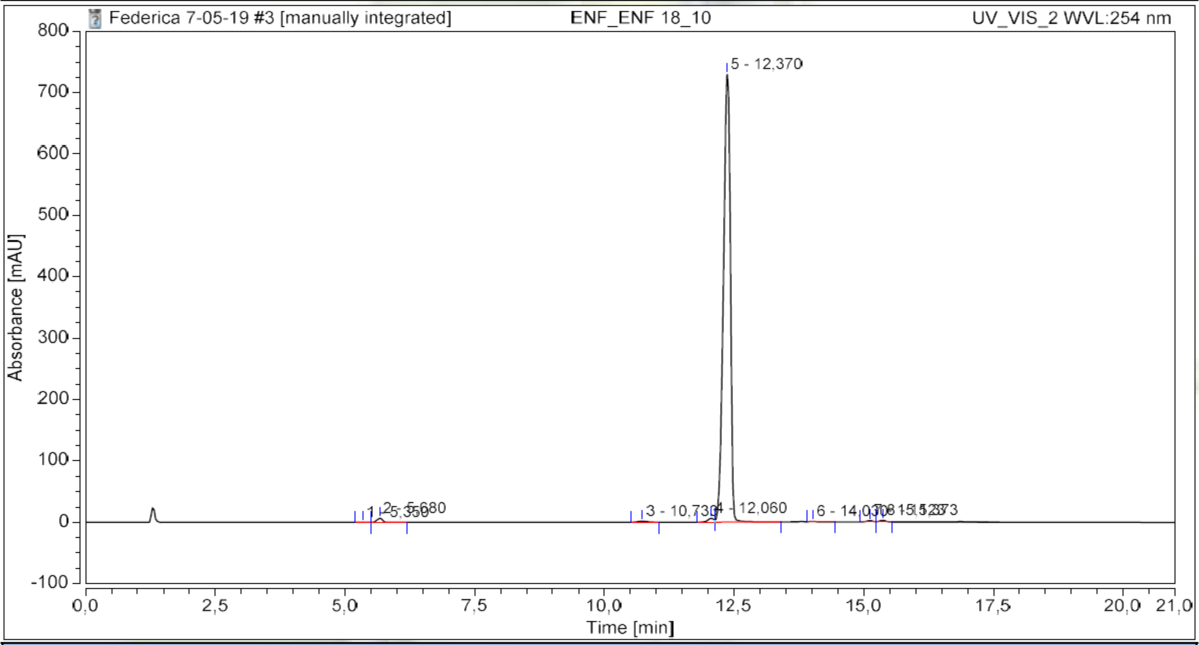

Integration Results

No.	Peak Name	Retention Time min	Area mAU*min	Height mAU	Relative Area %	Relative Height %	Amount n.a.
1		5,350	0,031	0,253	0,03	0,03	n.a.
2		5,680	0,780	6,467	0,68	0,86	n.a.
3		10,733	0,259	1,367	0,23	0,18	n.a.
4		12,060	0,800	6,053	0,69	0,81	n.a.
5		12,370	112,688	729,398	97,74	97,43	n.a.
6		14,030	0,124	0,654	0,11	0,09	n.a.
7		15,123	0,258	1,899	0,22	0,25	n.a.
8		15,373	0,349	2,572	0,30	0,34	n.a.
**Total:**			**115,289**	**748,663**	**100,00**	**100,00**	

Compound 17:

Chromatogram

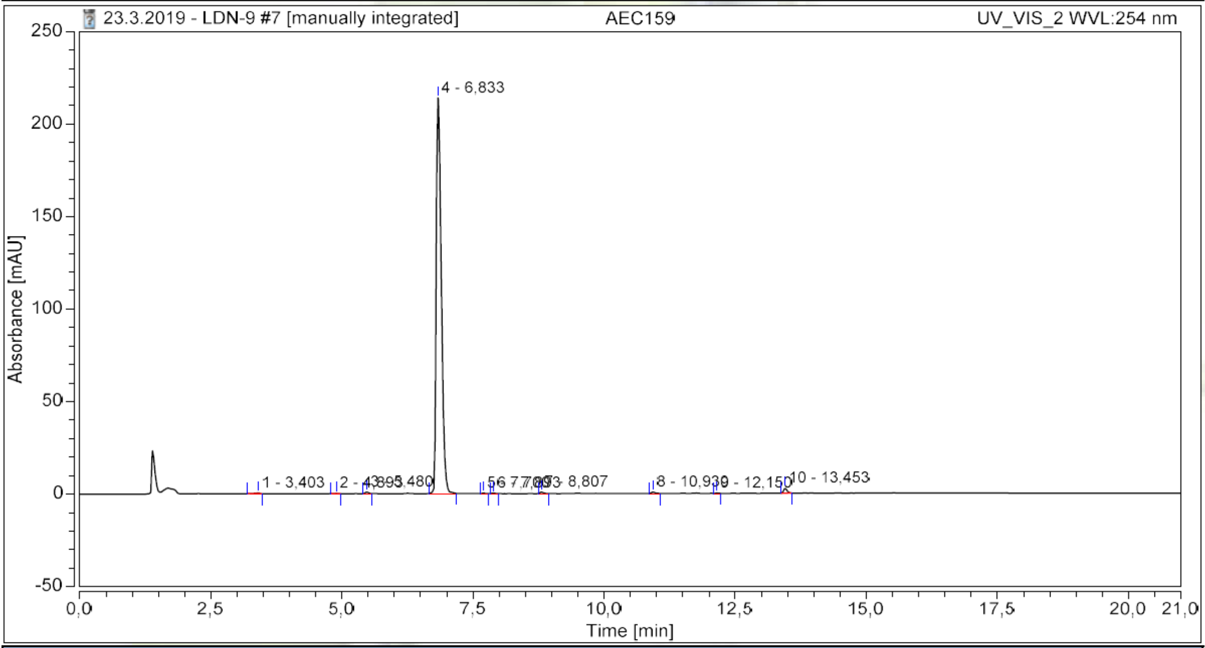

Integration Results

No.	Peak Name	Retention Time min	Area mAU*min	Height mAU	Relative Area %	Relative Height %	Amount n.a.
1		3,403	0,049	0,440	0,20	0,20	n.a.
2		4,893	0,020	0,258	0,08	0,12	n.a.
3		5,480	0,069	0,907	0,28	0,41	n.a.
4		6,833	23,897	213,819	97,85	96,86	n.a.
5		7,700	0,019	0,290	0,08	0,13	n.a.
6		7,893	0,018	0,228	0,08	0,10	n.a.
7		8,807	0,059	0,862	0,24	0,39	n.a.
8		10,930	0,065	0,847	0,26	0,38	n.a.
9		12,150	0,017	0,249	0,07	0,11	n.a.
10		13,453	0,209	2,853	0,85	1,29	n.a.
**Total:**			**24,423**	**220,752**	**100,00**	**100,00**	

Compound 18:

Chromatogram

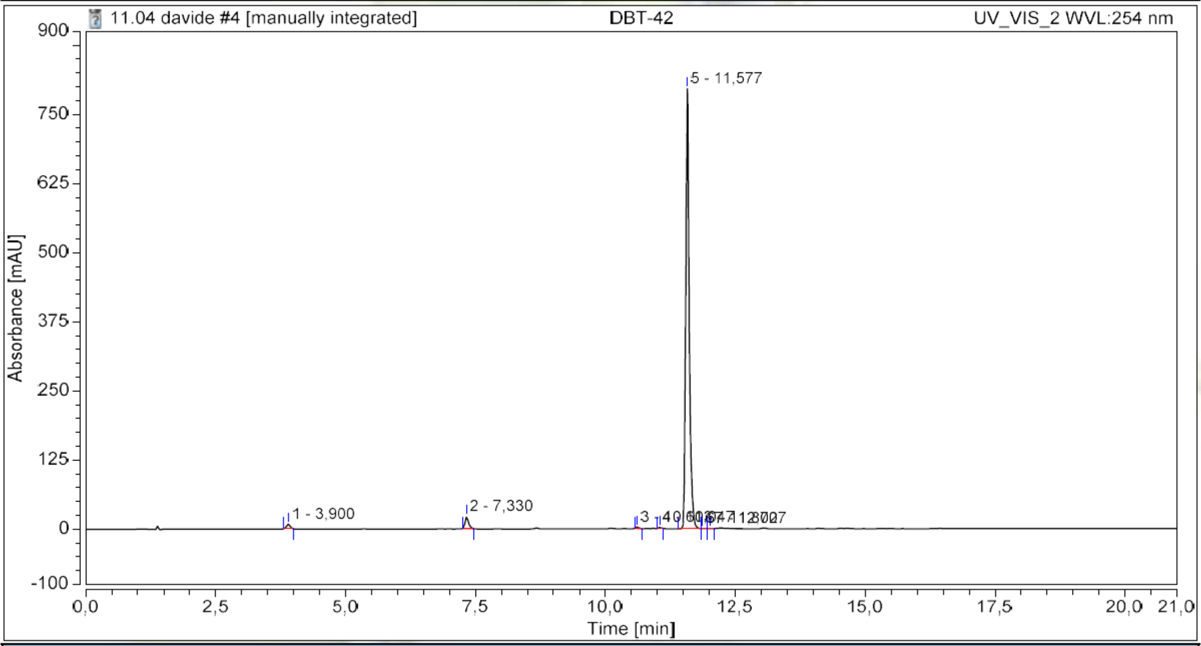

Integration Results

No.	Peak Name	Retention Time min	Area mAU*min	Height mAU	Relative Area %	Relative Height %	Amount n.a.
1		3,900	0,685	8,411	1,06	1,01	n.a.
2		7,330	1,679	21,869	2,60	2,63	n.a.
3		10,603	0,187	3,033	0,29	0,36	n.a.
4		11,047	0,107	1,849	0,17	0,22	n.a.
5		11,577	61,779	795,839	95,75	95,65	n.a.
6		11,870	0,045	0,526	0,07	0,06	n.a.
7		12,027	0,041	0,472	0,06	0,06	n.a.
**Total:**			**64,523**	**831,997**	**100,00**	**100,00**	

Compound 19

Chromatogram

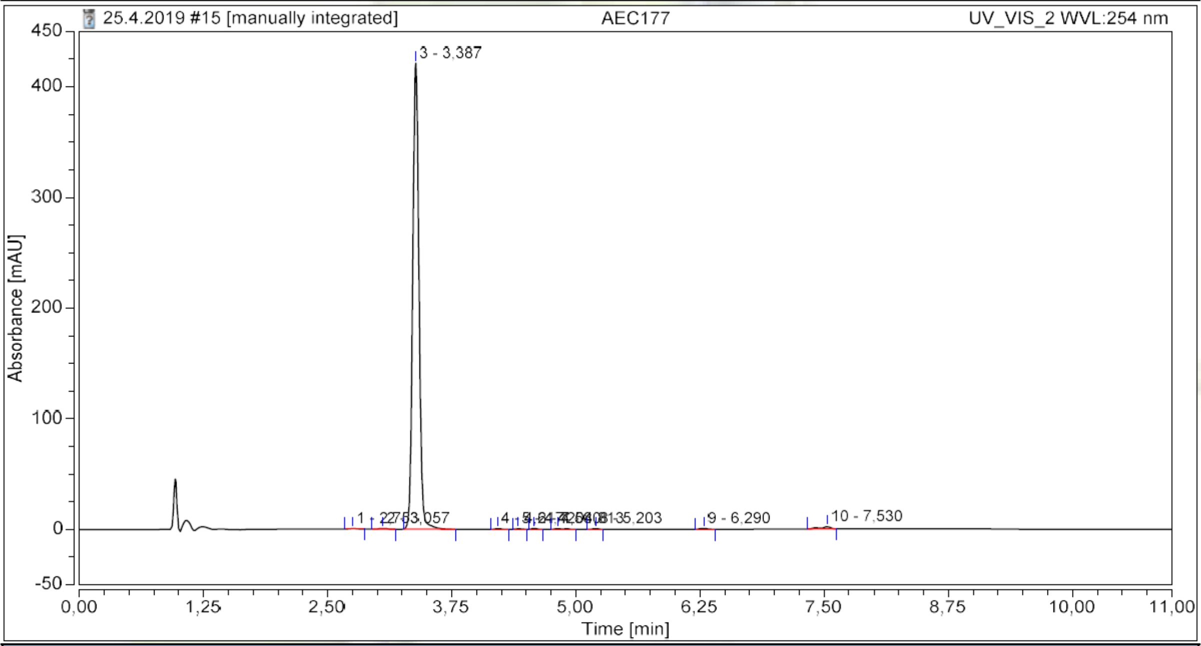

Integration Results

No.	Peak Name	Retention Time min	Area mAU*min	Height mAU	Relative Area %	Relative Height %	Amount n.a.
1		2,753	0,037	0,456	0,12	0,11	n.a.
2		3,057	0,063	0,678	0,20	0,16	n.a.
3		3,387	30,666	421,099	98,06	98,46	n.a.
4		4,217	0,033	0,500	0,10	0,12	n.a.
5		4,420	0,031	0,474	0,10	0,11	n.a.
6		4,580	0,042	0,695	0,13	0,16	n.a.
7		4,813	0,076	0,551	0,24	0,13	n.a.
8		5,203	0,042	0,645	0,13	0,15	n.a.
9		6,290	0,064	0,760	0,20	0,18	n.a.
10		7,530	0,217	1,841	0,69	0,43	n.a.
**Total:**			**31,271**	**427,699**	**100,00**	**100,00**	

Compound 20:

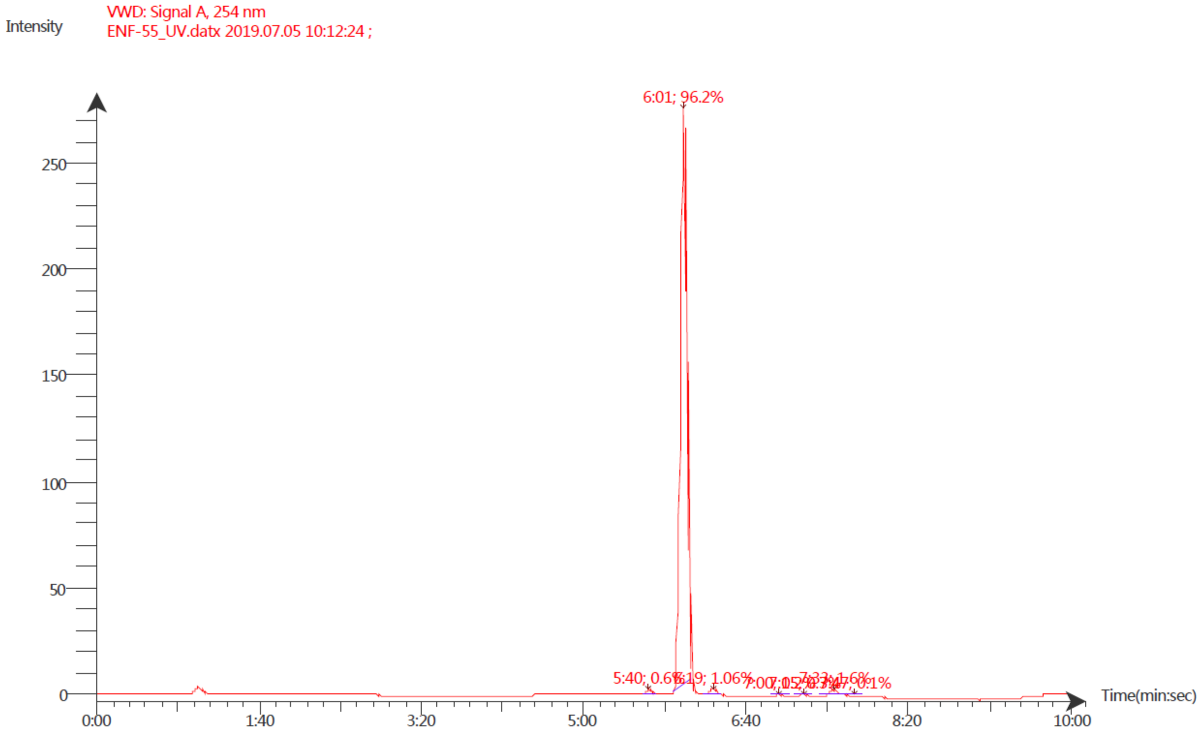

Compound 21:

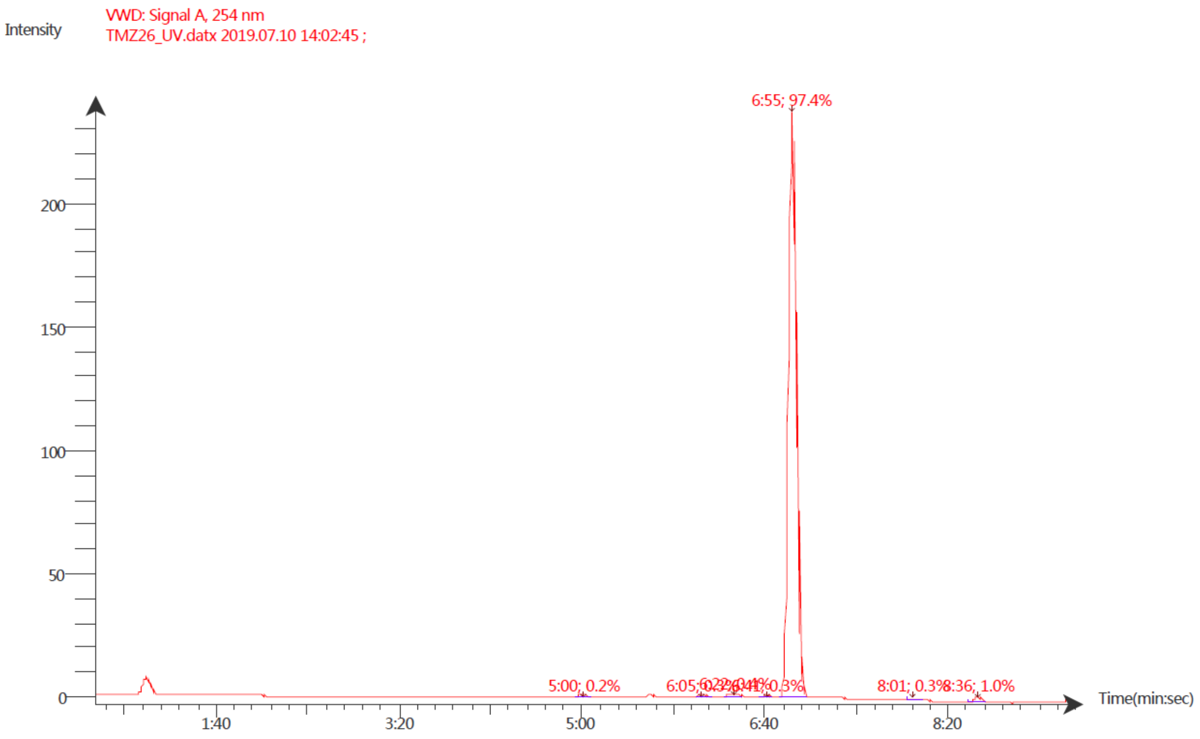

Compound 22:

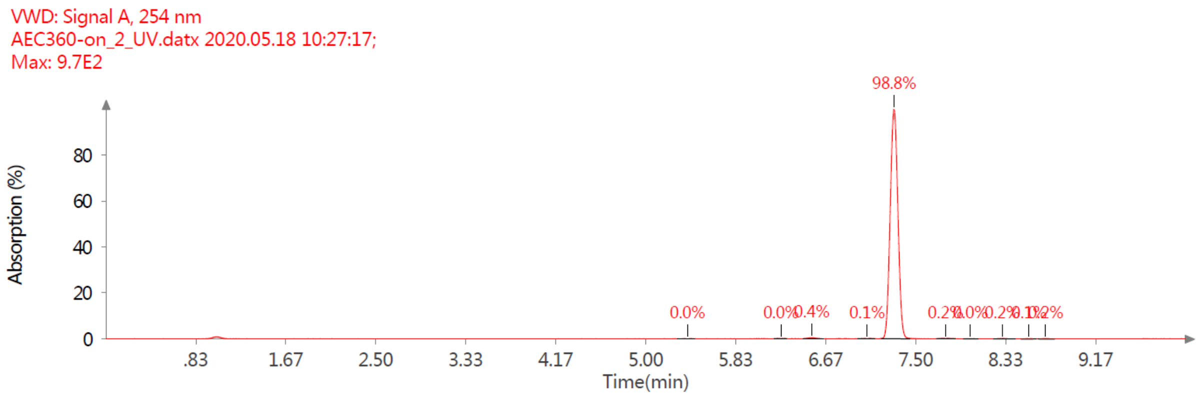

Time (Peak Maximum M:S/Minutes)	Maximum Absorption (mAU)	Time (Peak Centroid M:S/Minutes)	Peak Area	% Peak Area	Peak Resolution	Label
5.38	4.4E-1	5.39	2.1E0	0.0	4.4	
6.25	4.3E-1	6.25	1.6E0	0.0	3.9	
6.54	3.7E0	6.54	1.8E1	0.4	4.6	
7.05	5.8E-1	7.05	2.7E0	0.1	4.6	
7.30	9.8E2	7.30	4.8E3	98.8	4.7	
7.78	1.6E0	7.78	7.8E0	0.2	4.7	
8.01	4.5E-1	8.01	2E0	0.0	4.7	
8.30	1.8E0	8.30	9.5E0	0.2	5.0	
8.55	1.6E0	8.55	6.9E0	0.1	4.3	
8.70	1.8E0	8.70	7.8E0	0.2	4.4	

Compound 23:

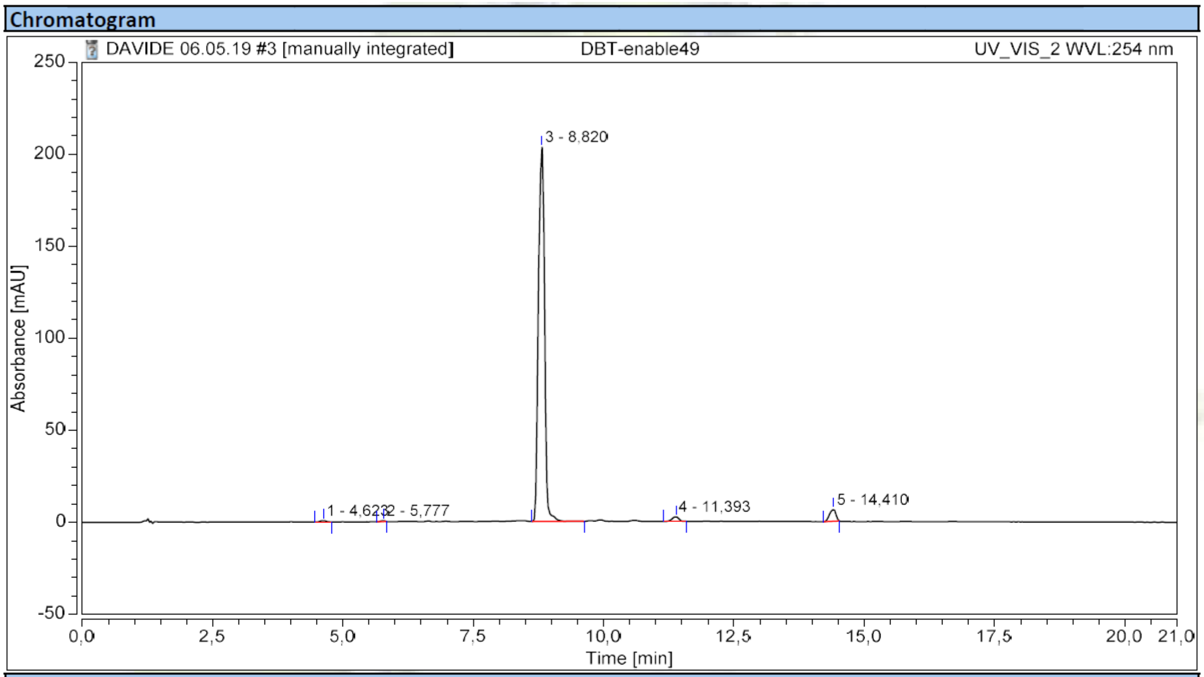

Integration Results

No.	Peak Name	Retention Time min	Area mAU*min	Height mAU	Relative Area %	Relative Height %	Amount n.a.
1		4,623	0,098	0,774	0,34	0,36	n.a.
2		5,777	0,055	0,517	0,19	0,24	n.a.
3		8,820	27,730	203,411	95,03	95,21	n.a.
4		11,393	0,386	2,535	1,32	1,19	n.a.
5		14,410	0,910	6,397	3,12	2,99	n.a.
**Total:**			**29,180**	**213,635**	**100,00**	**100,00**	

Compound 24:

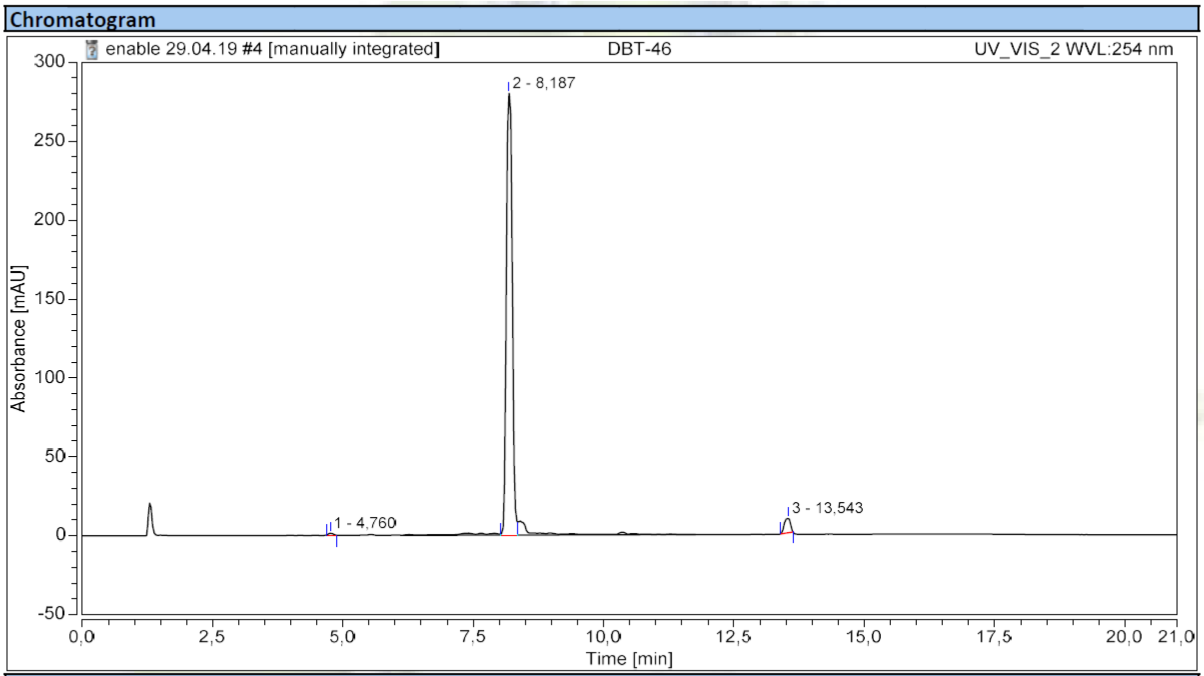

Integration Results

No.	Peak Name	Retention Time min	Area mAU*min	Height mAU	Relative Area %	Relative Height %	Amount n.a.
1		4,760	0,117	1,132	0,31	0,39	n.a.
2		8,187	36,811	279,807	96,43	96,48	n.a.
3		13,543	1,244	9,088	3,26	3,13	n.a.
**Total:**			**38,173**	**290,028**	**100,00**	**100,00**	

Compound 25:

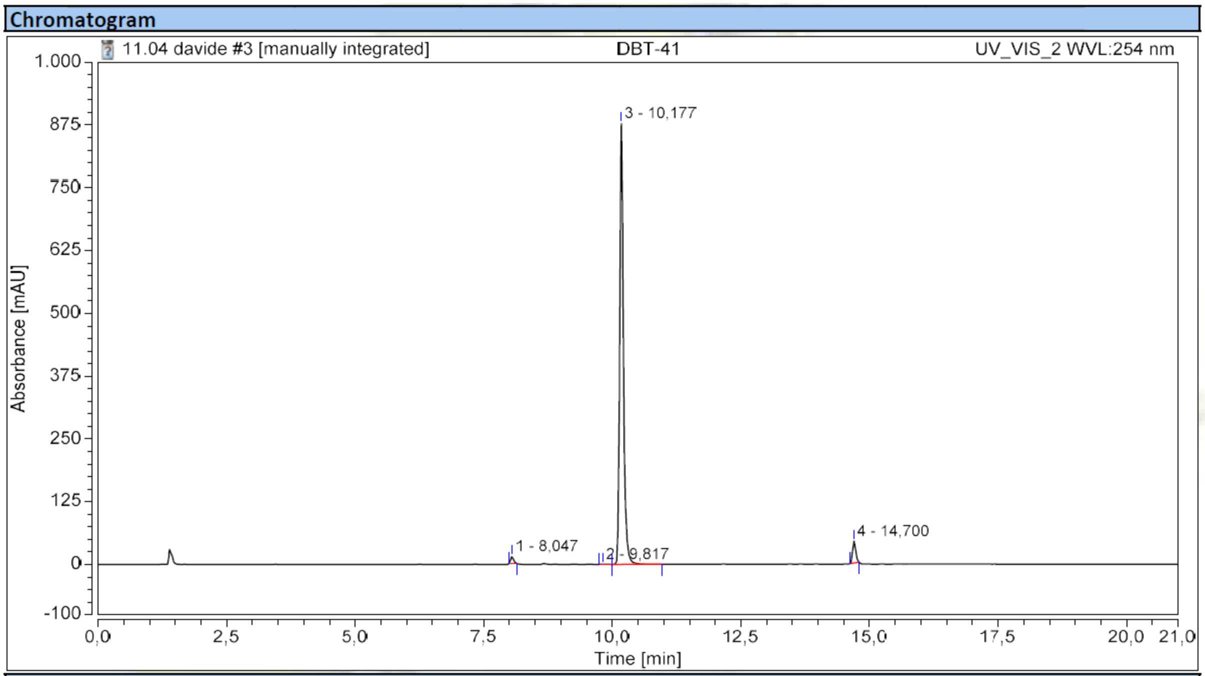

Integration Results

No.	Peak Name	Retention Time min	Area mAU*min	Height mAU	Relative Area %	Relative Height %	Amount n.a.
1		8,047	1,010	14,691	1,32	1,57	n.a.
2		9,817	0,043	0,505	0,06	0,05	n.a.
3		10,177	72,365	877,741	94,62	93,70	n.a.
4		14,700	3,059	43,866	4,00	4,68	n.a.
**Total:**			**76,477**	**936,803**	**100,00**	**100,00**	

Compound 26:

Chromatogram

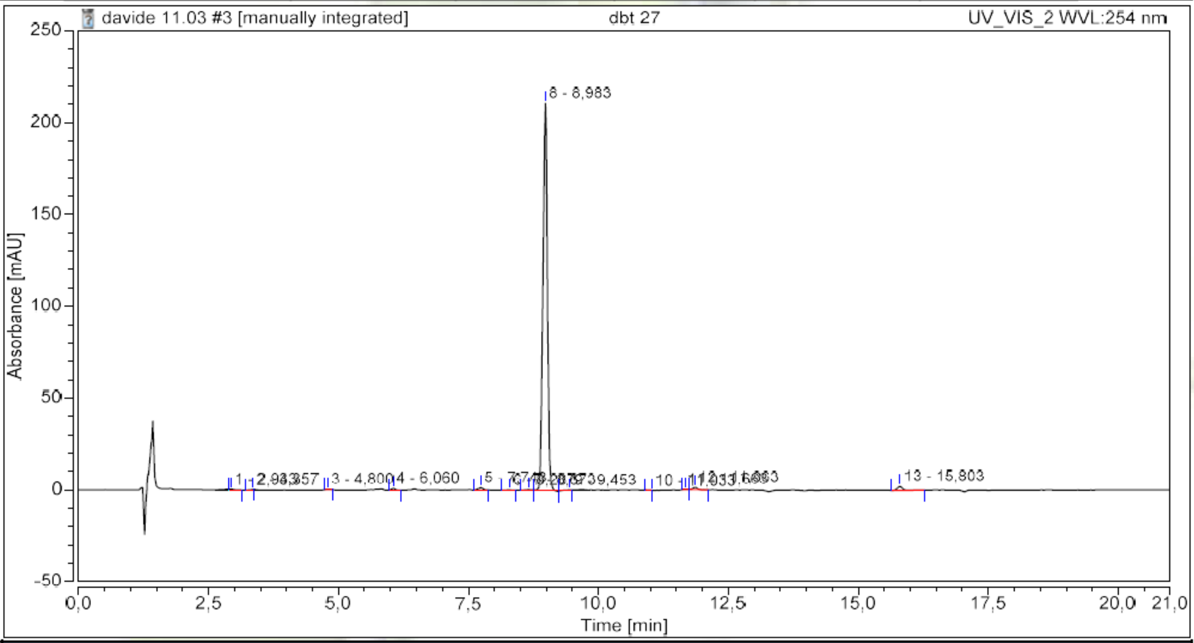

Integration Results

No.	Peak Name	Retention Time min	Area mAU*min	Height mAU	Relative Area %	Relative Height %	Amount n.a.
1		2,943	0,034	0,353	0,16	0,16	n.a.
2		3,357	0,029	0,415	0,13	0,19	n.a.
3		4,800	0,025	0,298	0,11	0,14	n.a.
4		6,060	0,087	1,019	0,40	0,47	n.a.
5		7,743	0,139	1,367	0,64	0,63	n.a.
6		8,287	0,019	0,181	0,09	0,08	n.a.
7		8,673	0,029	0,247	0,13	0,11	n.a.
8		8,983	20,829	210,740	96,29	96,42	n.a.
9		9,453	0,022	0,052	0,10	0,02	n.a.
10		11,033	0,004	0,000	0,02	0,00	n.a.
11		11,663	0,020	0,286	0,09	0,13	n.a.
12		11,863	0,136	1,331	0,63	0,61	n.a.
13		15,803	0,259	2,274	1,20	1,04	n.a.
**Total:**			**21,631**	**218,563**	**100,00**	**100,00**	

Compound 27:

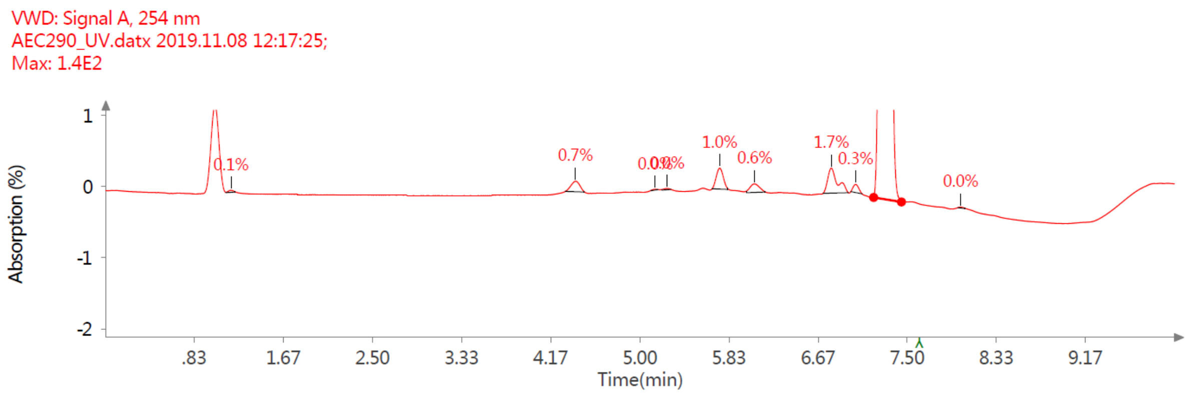

Time (Peak Maximum M:S/Minutes)	Maximum Absorption (mAU)	Time (Peak Centroid M:S/Minutes)	Peak Area	% Peak Area	Peak Resolution	Label
1.17	1.6E-1	1.18	5.2E-1	0.1	3.3	
4.40	8.4E-1	4.40	4.8E0	0.7	5.7	
5.15	5.3E-2	5.14	1.7E-1	0.0	4.0	
5.25	9.8E-2	5.25	3.5E-1	0.0	3.6	
5.75	1.7E0	5.75	7.5E0	1.0	4.5	
6.07	6.8E-1	6.07	4.1E0	0.6	6.2	
6.79	2E0	6.79	1.3E1	1.7	4.6	
7.02	6.5E-1	7.02	2.3E0	0.3	3.6	
7.29	1.4E2	7.29	6.9E2	95.5	4.7	
8.00	6.3E-2	8.00	1.9E-1	0.0	3.6	

Compound 28:

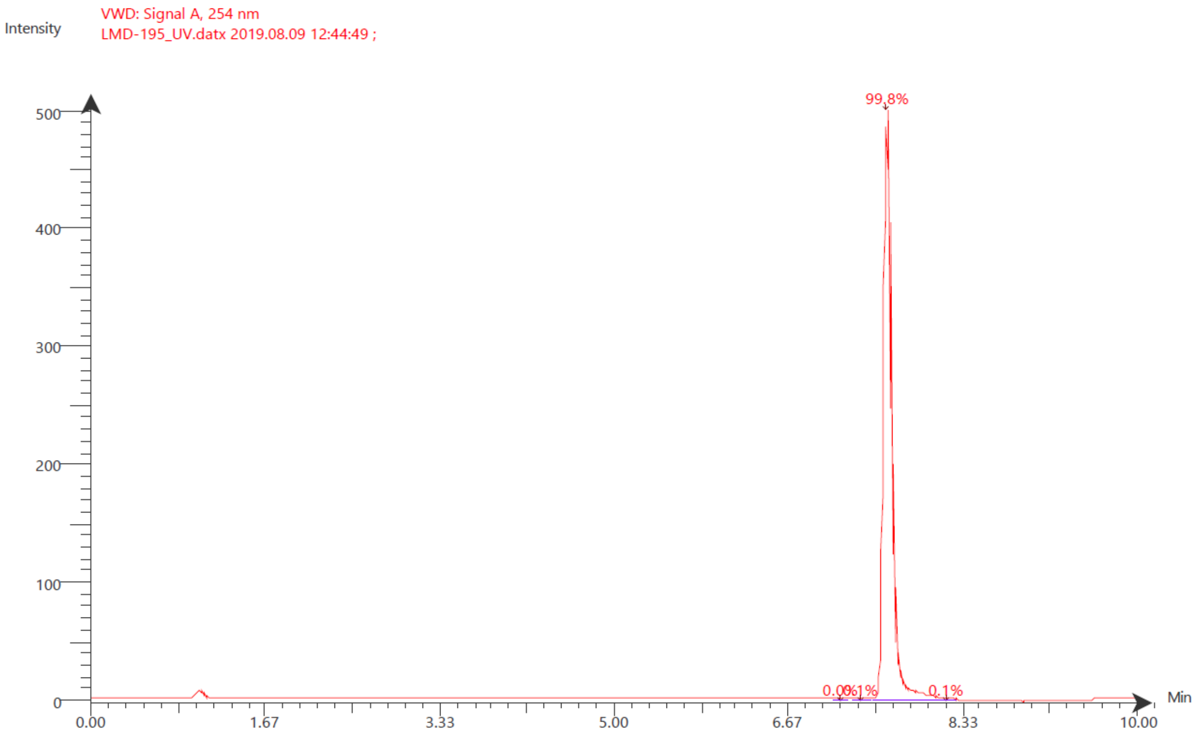

Compound 29:

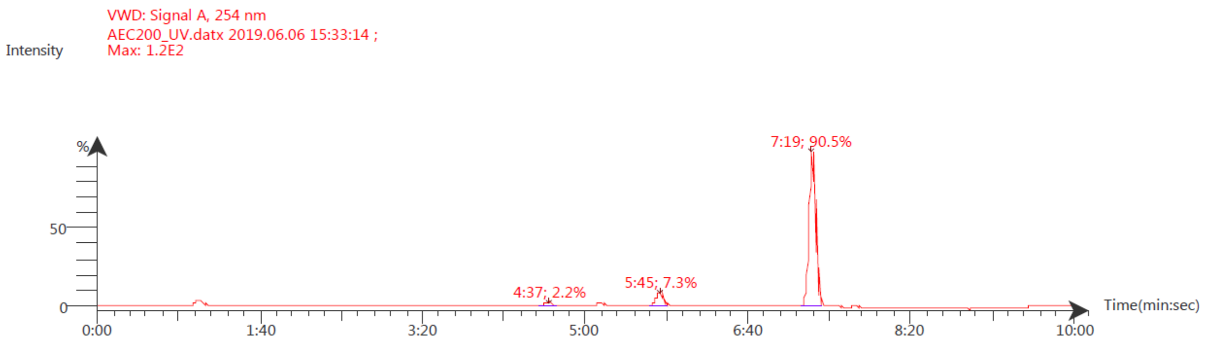

Time (Peak Maximum M:S/Minutes)	Maximum Intensity (c/s)	Time (Peak Centroid M:S/Minutes)	Peak Area	% Peak Area	Peak Resolution	Label
4:36.64	2.9E0	4:36.86	1.4E1	2.2	4.8	
5:45.44	9.7E0	5:45.30	4.7E1	7.3	4.6	
7:19.44	1.2E2	7:19.35	5.8E2	90.5	4.7	

Compound 30:

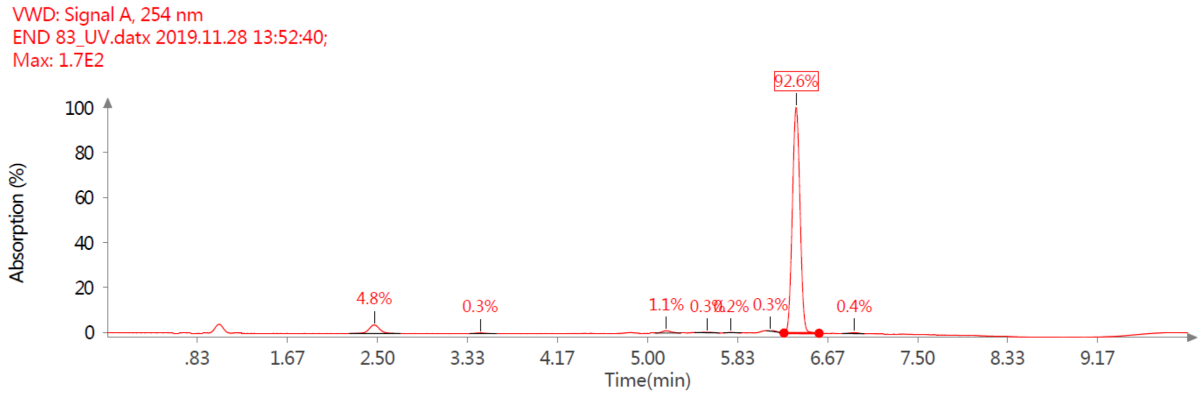

Time (Peak Maximum M:S/Minutes)	Maximum Absorption (mAU)	Time (Peak Centroid M:S/Minutes)	Peak Area	% Peak Area	Peak Resolution	Label
2.47	6.3E0	2.47	4.3E1	4.8	6.1	
3.45	4.5E-1	3.45	2.8E0	0.3	5.9	
5.17	1.7E0	5.17	9.7E0	1.1	5.4	
5.56	3.5E-1	5.56	2.3E0	0.3	13.6	
5.77	4E-1	5.77	1.9E0	0.2	4.7	
6.14	4.4E-1	6.14	2.5E0	0.3	7.2	
6.37	1.7E2	6.38	8.2E2	92.6	4.7	
6.91	6.8E-1	6.91	3.4E0	0.4	4.8	

Compound 31:

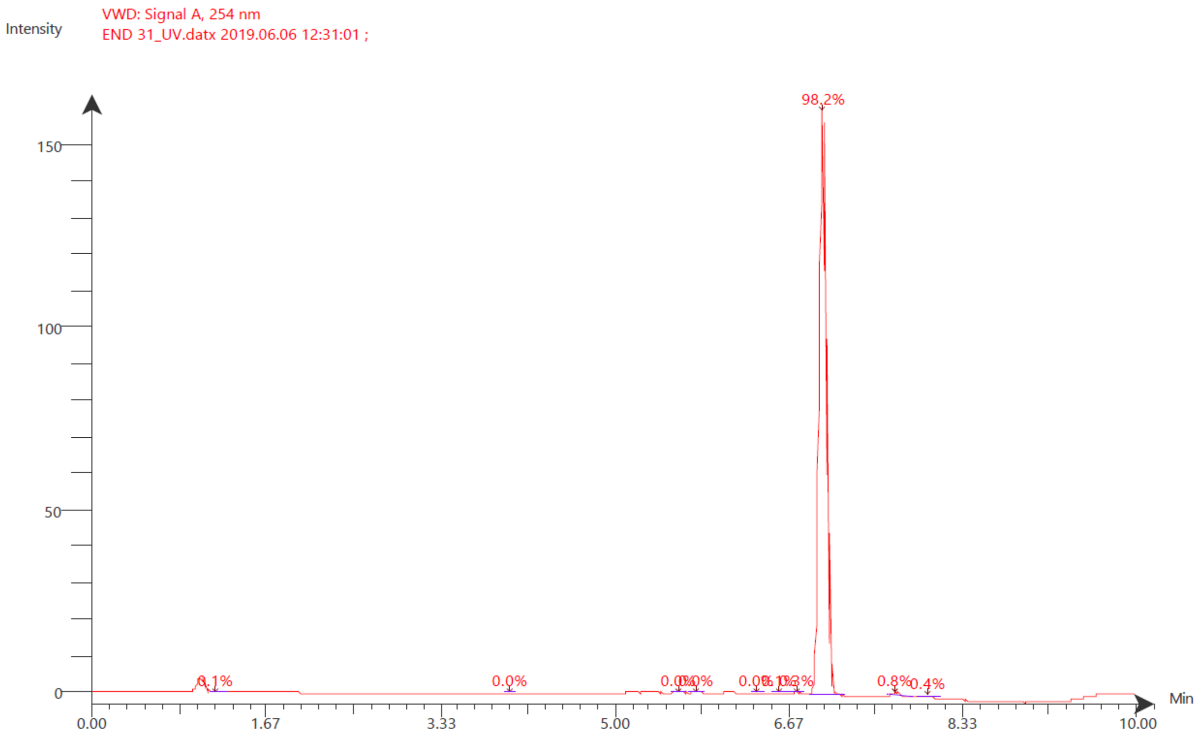

Compound 32:

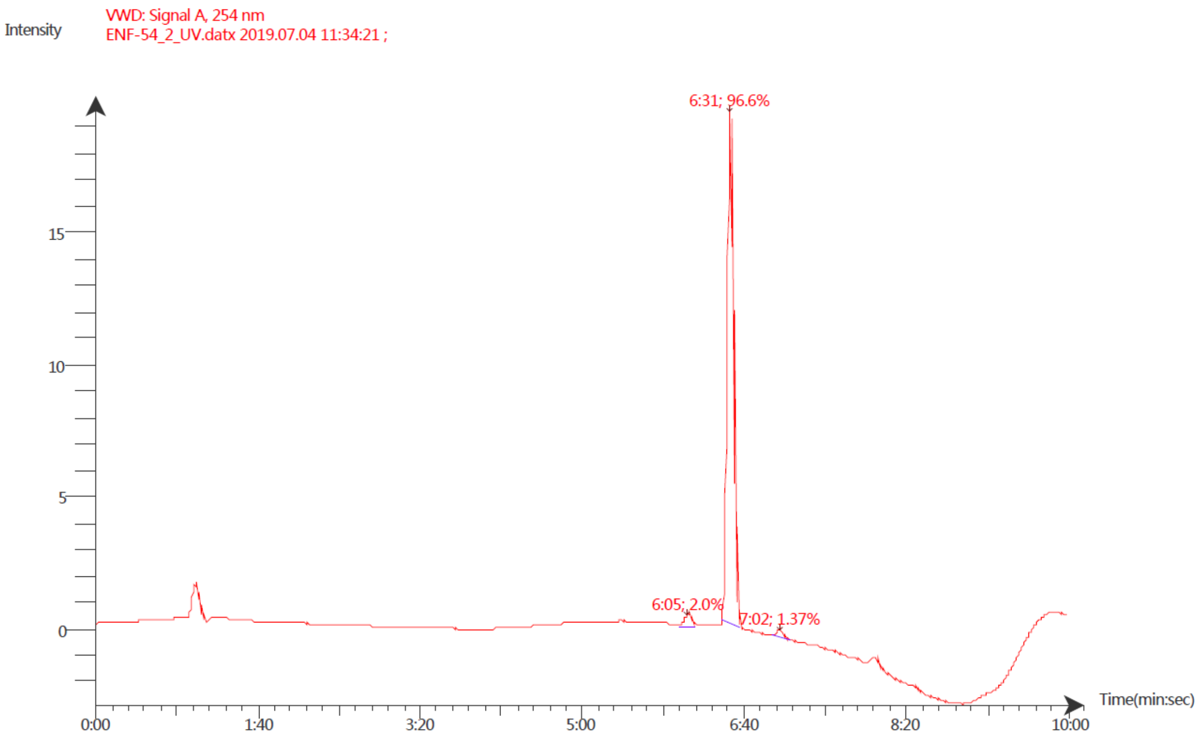

Compound 33:

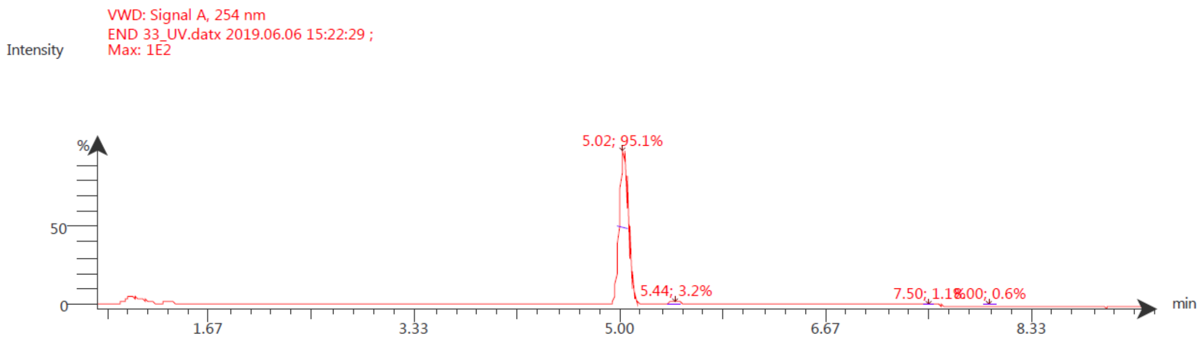

Time (Peak Maximum M:S/Minutes)	Maximum Intensity (c/s)	Time (Peak Centroid M:S/Minutes)	Peak Area	% Peak Area	Peak Resolution	Label
5.02	5.2E1	5.02	1.5E2	95.1	3.1	
5.44	1.4E0	5.44	5.2E0	3.2	3.8	
7.50	5.4E-1	7.50	1.8E0	1.1	3.5	
8.00	2.5E-1	8.00	1E0	0.6	4.3	

Compound 34:

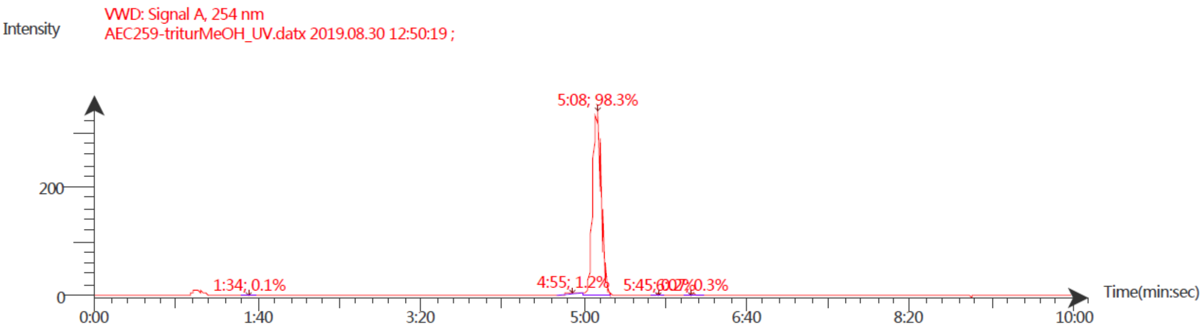

Time (Peak Maximum M:S/Minutes)	Maximum Intensity (c/s)	Time (Peak Centroid M:S/Minutes)	Peak Area	% Peak Area	Peak Resolution	Label
1:34.13	3.2E-1	1:33.72	1.7E0	0.1	5.6	
4:55.33	3E0	4:53.46	2.5E1	1.2	7.8	
5:07.73	3.4E2	5:07.69	2E3	98.3	5.5	
5:45.33	7.3E-1	5:45.29	3.3E0	0.2	4.5	
6:06.93	9.4E-1	6:06.31	5.9E0	0.3	6.2	

Compound 35:

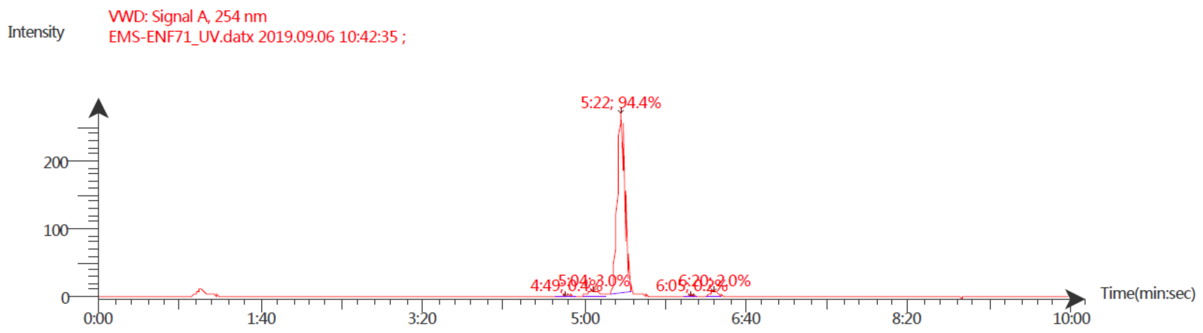

Time (Peak Maximum M:S/Minutes)	Maximum Intensity (c/s)	Time (Peak Centroid M:S/Minutes)	Peak Area	% Peak Area	Peak Resolution	Label
4:48.89	1.1E0	4:48.96	6.3E0	0.4	5.7	
5:04.09	6.9E0	5:05.46	4.5E1	3.0	6.2	
5:22.09	2.7E2	5:22.22	1.4E3	94.4	5.2	
6:05.29	6.3E-1	6:05.42	3E0	0.2	4.8	
6:20.09	6.5E0	6:19.99	3.1E1	2.0	4.6	

Compound 36:

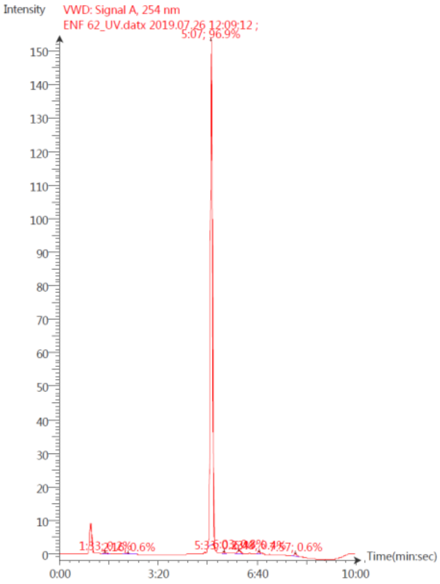

Compound 37:

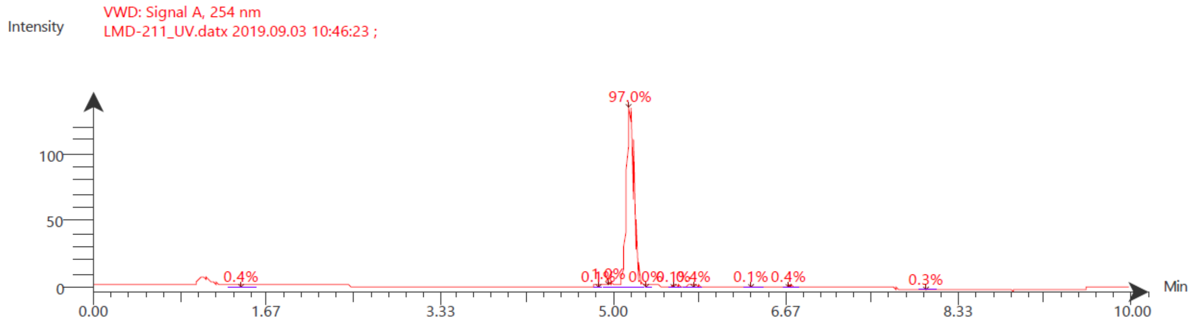

Compound 38:

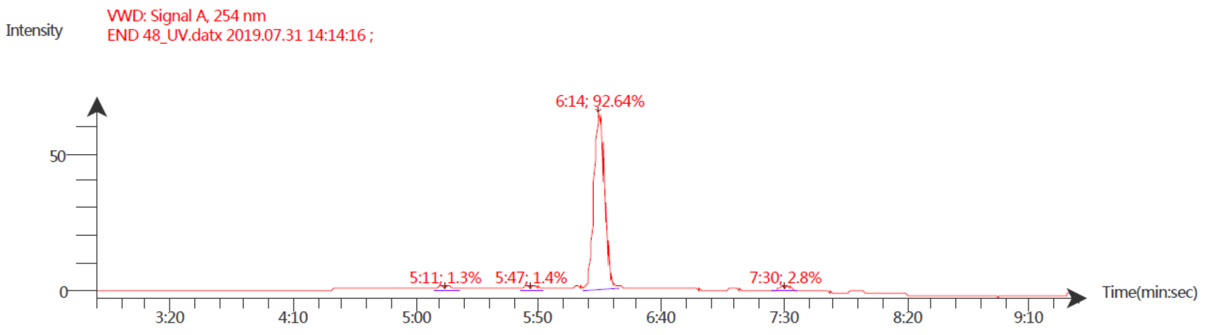

Compound 39:

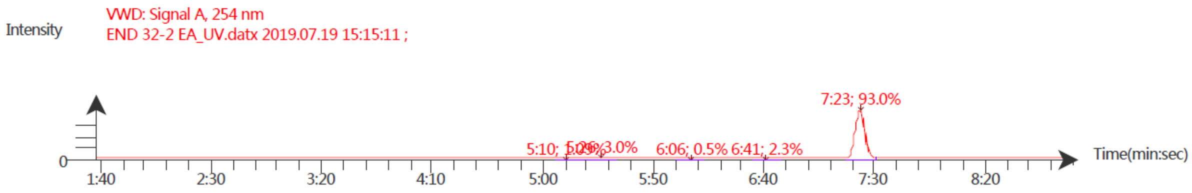

Compound 40:

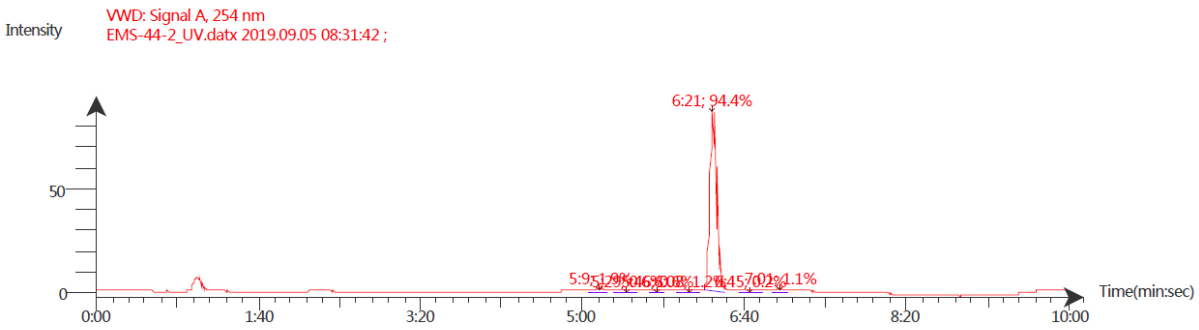

Time (Peak Maximum M:S/Minutes)	Maximum Intensity (c/s)	Time (Peak Centroid M:S/Minutes)	Peak Area	% Peak Area	Peak Resolution	Label
5:9.41	1.6E0	5:9.47	8.3E0	1.9	4.8	
5:29.41	3.3E-1	5:27.71	2.6E0	0.6	6.0	
5:45.81	5.7E-1	5:45.99	2.6E0	0.6	4.6	
6:08.21	6.4E-1	6:06.41	5.2E0	1.2	8.2	
6:20.61	8.6E1	6:20.49	4.2E2	94.4	4.7	
6:44.61	1.4E-1	6:43.41	1E0	0.2	8.4	
7:01.01	1.1E0	7:01.18	5.1E0	1.1	4.5	

Compound 41:

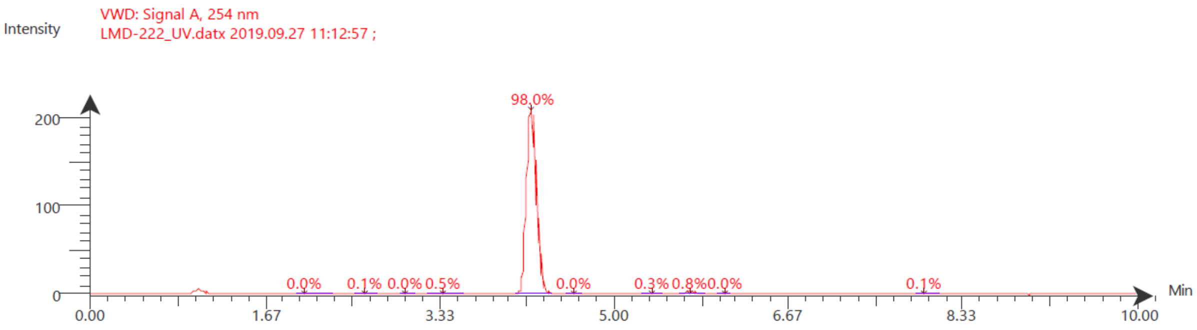

Compound 42:

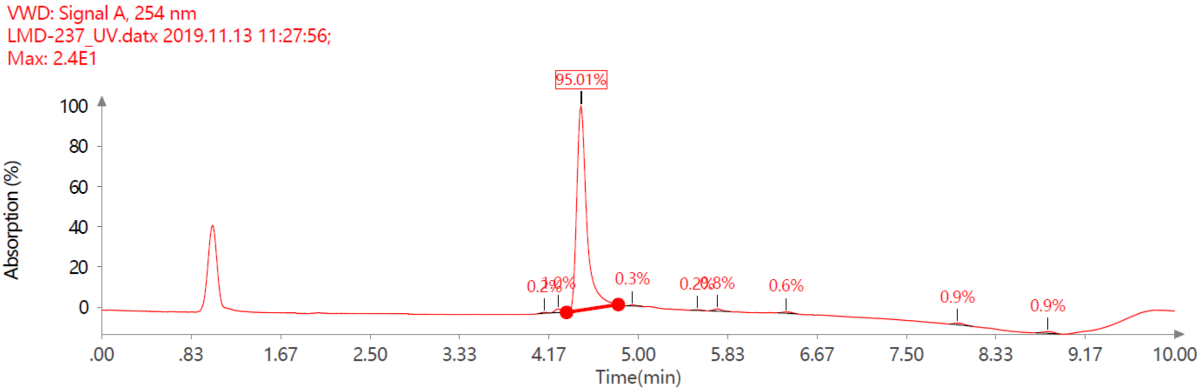

Compound 43:

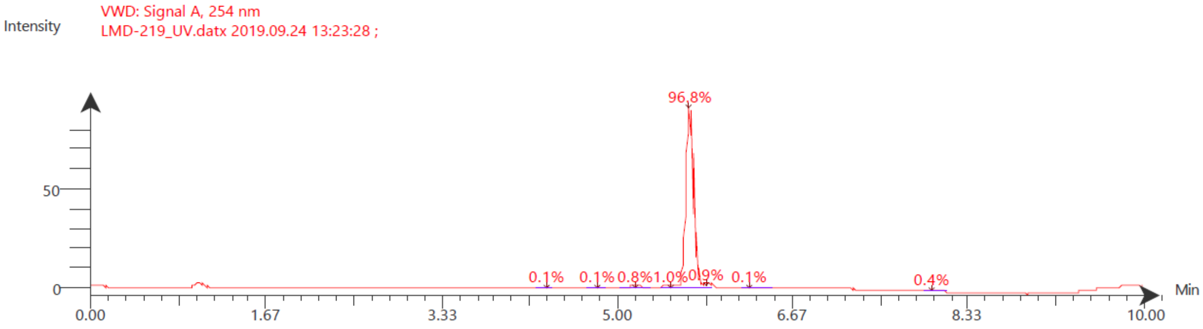

Compound 44:

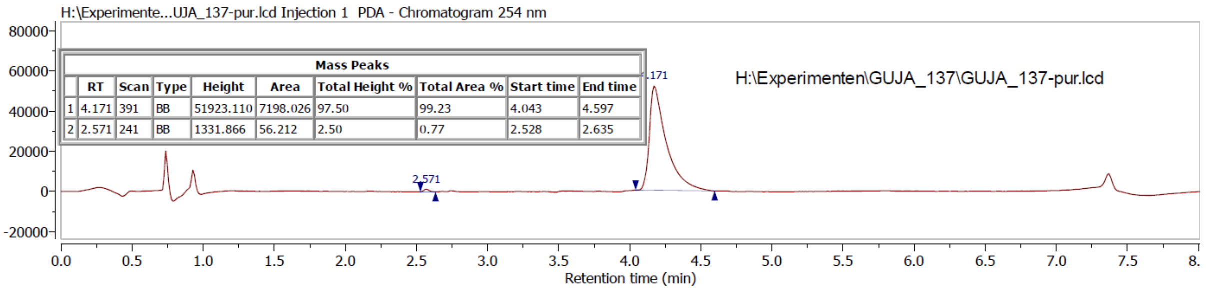

	RT	Scan	Type	Height	Area	Total Height %	Total Area %	Start time	End time
1	4.171	391	BB	51923.110	7198.026	97.50	99.23	4.043	4.597
2	2.571	241	BB	1331.866	56.212	2.50	0.77	2.528	2.635

Compound 45:

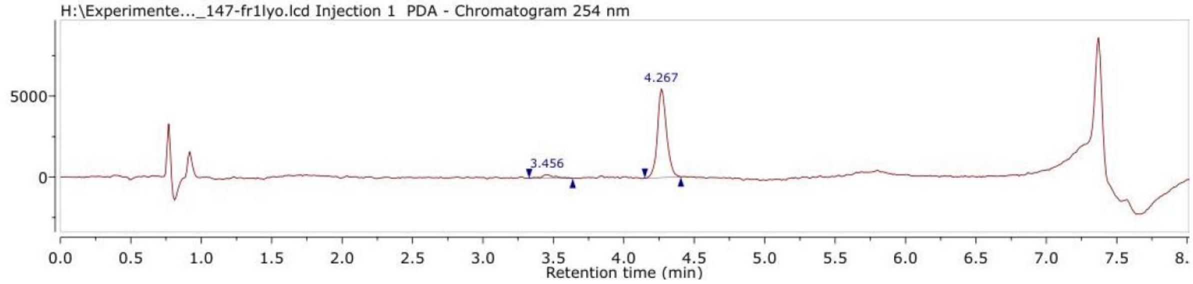

Compound 46:

Chromatogram

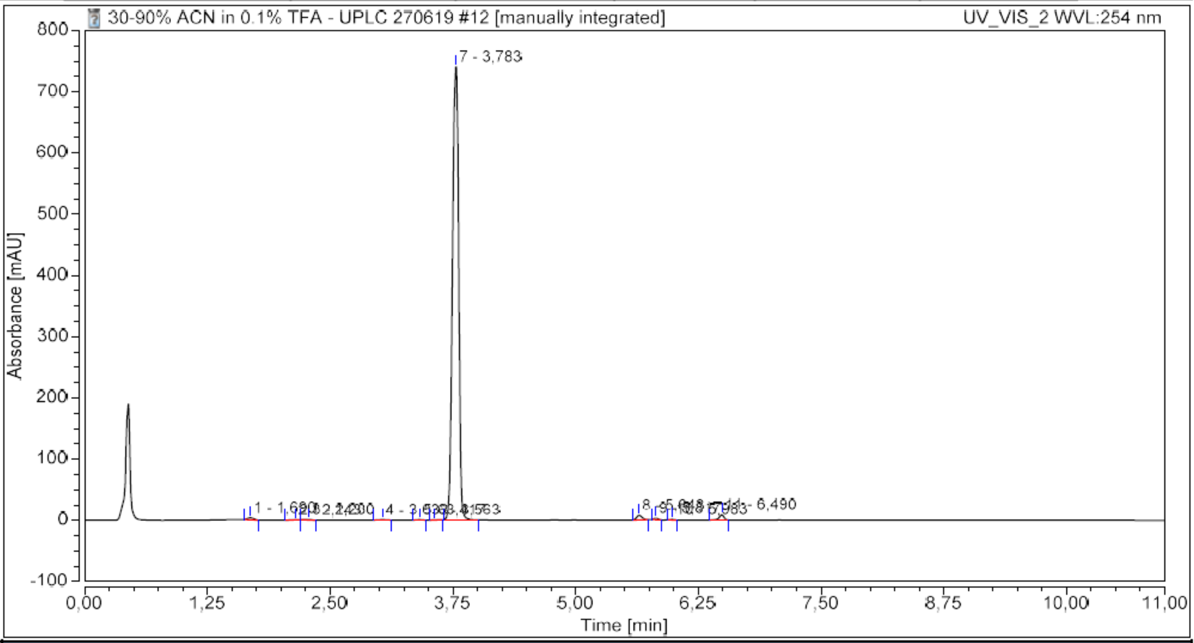

Integration Results

No.	Peak Name	Retention Time min	Area mAU*min	Height mAU	Relative Area %	Relative Height %	Amount n.a.
1		1,690	0,237	3,229	0,42	0,42	n.a.
2		2,143	0,020	0,202	0,03	0,03	n.a.
3		2,280	0,071	0,687	0,13	0,09	n.a.
4		3,033	0,070	0,678	0,13	0,09	n.a.
5		3,417	0,051	0,690	0,09	0,09	n.a.
6		3,563	0,022	0,374	0,04	0,05	n.a.
7		3,783	54,413	740,369	97,07	96,57	n.a.
8		5,643	0,496	7,660	0,88	1,00	n.a.
9		5,817	0,124	2,530	0,22	0,33	n.a.
10		5,983	0,052	1,140	0,09	0,15	n.a.
11		6,490	0,501	9,091	0,89	1,19	n.a.
**Total:**			**56,056**	**766,650**	**100,00**	**100,00**	

Compound 47:

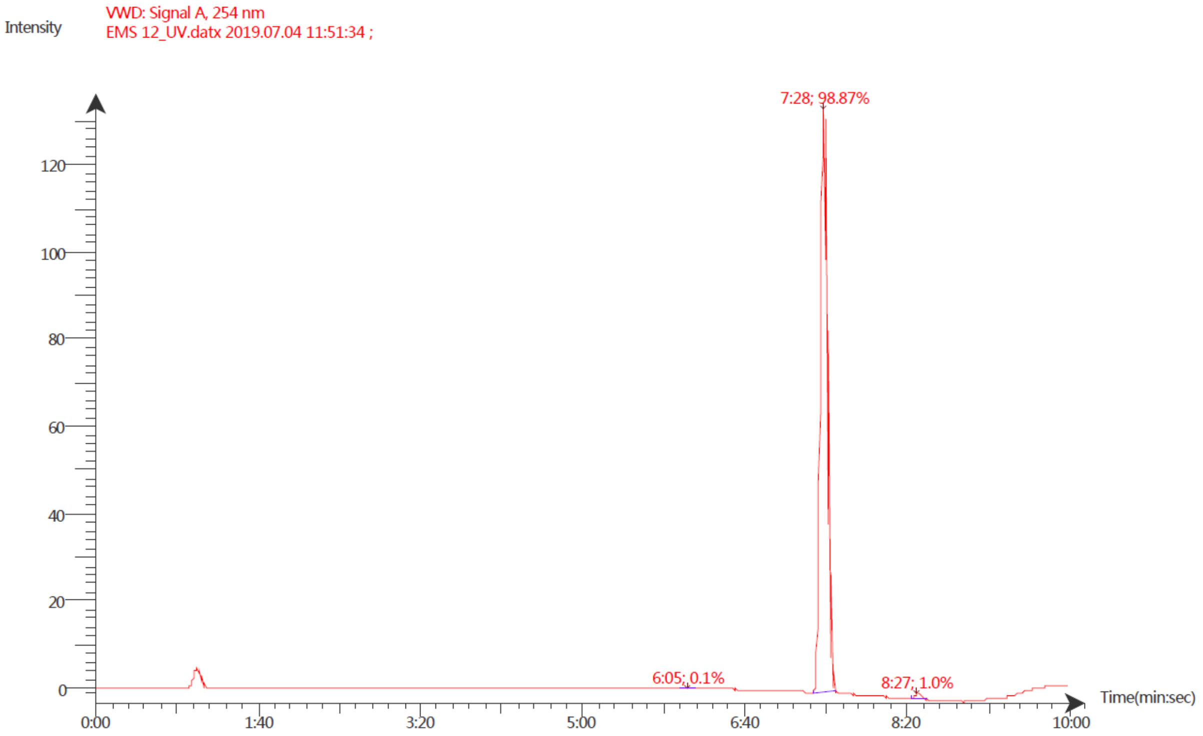

Compound 48:

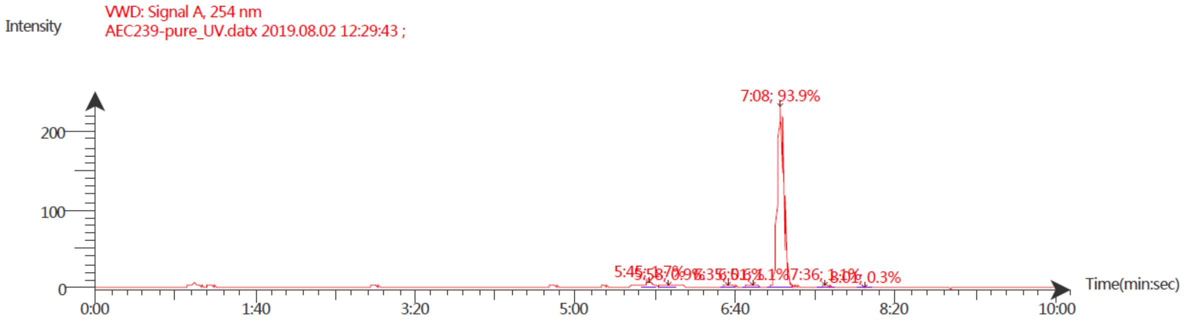

Time (Peak Maximum M:S/Minutes)	Maximum Intensity (c/s)	Time (Peak Centroid M:S/Minutes)	Peak Area	% Peak Area	Peak Resolution	Label
2:53.87	4.7E-1	2:53.93	3E0	0.2	6.2	
4:45.47	2.6E-1	4:46.38	1.7E0	0.1	7.1	
5:45.47	4.5E0	5:45.57	2.1E1	1.7	4.5	
5:58.27	2E0	5:58.45	1.1E1	0.9	5.2	
6:35.47	1.6E0	6:35.48	7.3E0	0.6	4.3	
6:50.67	2.7E0	6:50.45	1.3E1	1.1	4.9	
7:07.87	2.3E2	7:07.99	1.2E3	93.9	4.7	
7:36.27	2.8E0	7:36.34	1.3E1	1.1	4.6	
8:01.07	7.4E-1	8:00.28	4E0	0.3	5.5	

Compound 49:

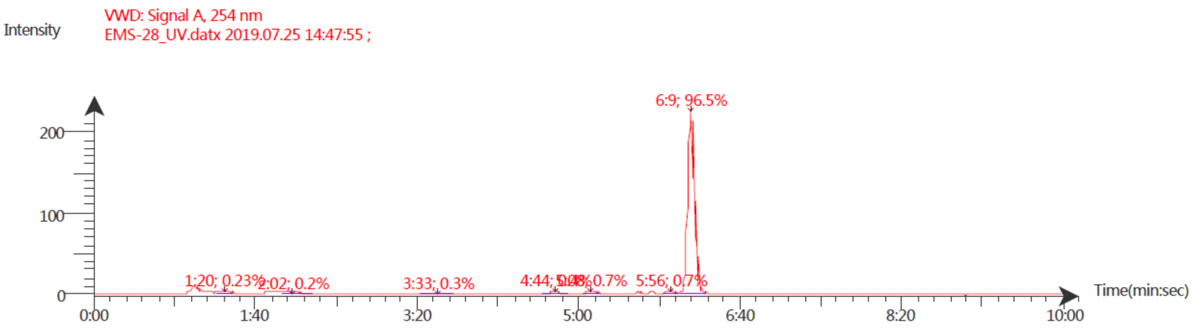

Compound 50:

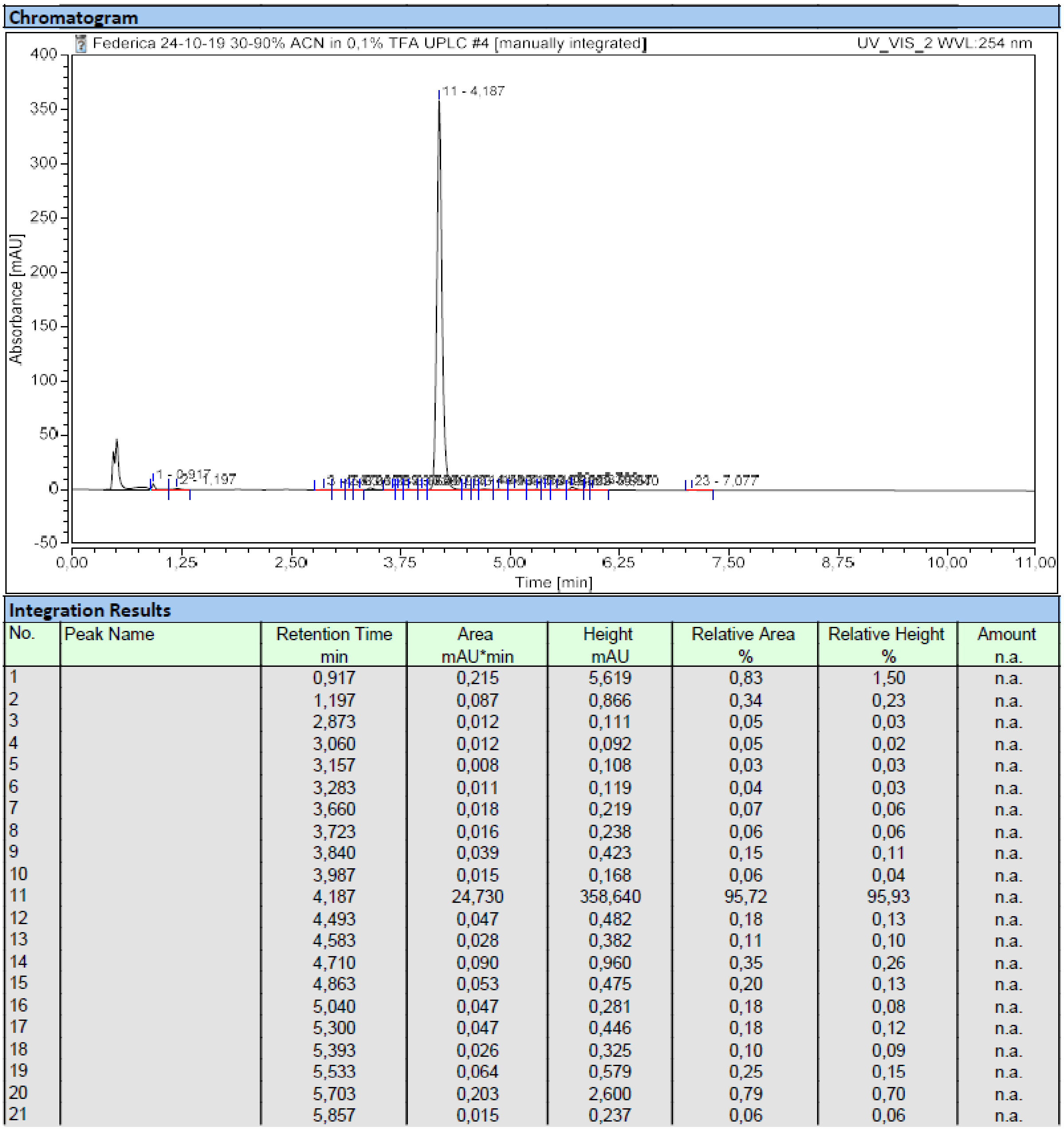

Integration Results

No.	Peak Name	Retention Time min	Area mAU*min	Height mAU	Relative Area %	Relative Height %	Amount n.a.
1		0,917	0,215	5,619	0,83	1,50	n.a.
2		1,197	0,087	0,866	0,34	0,23	n.a.
3		2,873	0,012	0,111	0,05	0,03	n.a.
4		3,060	0,012	0,092	0,05	0,02	n.a.
5		3,157	0,008	0,108	0,03	0,03	n.a.
6		3,283	0,011	0,119	0,04	0,03	n.a.
7		3,660	0,018	0,219	0,07	0,06	n.a.
8		3,723	0,016	0,238	0,06	0,06	n.a.
9		3,840	0,039	0,423	0,15	0,11	n.a.
10		3,987	0,015	0,168	0,06	0,04	n.a.
11		4,187	24,730	358,640	95,72	95,93	n.a.
12		4,493	0,047	0,482	0,18	0,13	n.a.
13		4,583	0,028	0,382	0,11	0,10	n.a.
14		4,710	0,090	0,960	0,35	0,26	n.a.
15		4,863	0,053	0,475	0,20	0,13	n.a.
16		5,040	0,047	0,281	0,18	0,08	n.a.
17		5,300	0,047	0,446	0,18	0,12	n.a.
18		5,393	0,026	0,325	0,10	0,09	n.a.
19		5,533	0,064	0,579	0,25	0,15	n.a.
20		5,703	0,203	2,600	0,79	0,70	n.a.
21		5,857	0,015	0,237	0,06	0,06	n.a.

Compound 51:

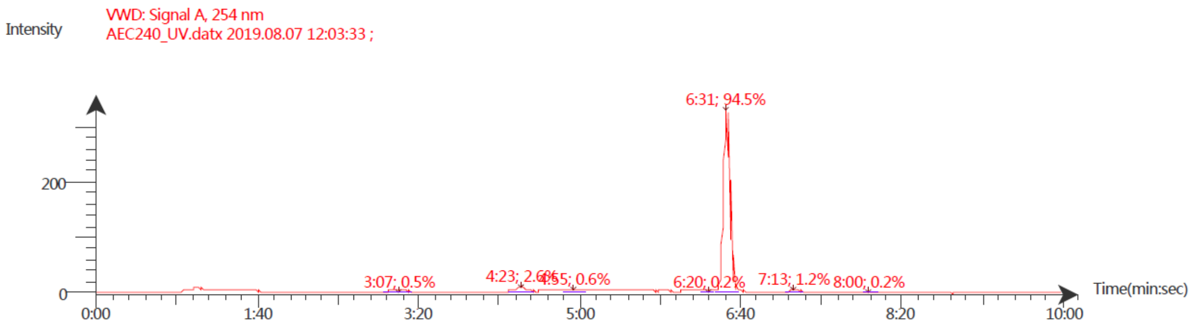

Time (Peak Maximum M:S/Minutes)	Maximum Intensity (c/s)	Time (Peak Centroid M:S/Minutes)	Peak Area	% Peak Area	Peak Resolution	Label
3:07.24	1.1E0	3:06.71	9.4E0	0.5	8.9	
4:23.24	7E0	4:22.99	4.5E1	2.6	6.2	
4:54.84	1.7E0	4:55.54	1E1	0.6	5.4	
6:06.44	5.1E-1	6:06.37	2.3E0	0.1	4.5	
6:19.64	9.5E-1	6:19.28	4.1E0	0.2	4.2	
6:30.84	3.3E2	6:30.92	1.6E3	94.5	4.7	
7:13.24	4.5E0	7:13.04	2.1E1	1.2	4.5	
8:00.04	5.9E-1	7:59.80	3.1E0	0.2	5.3	

Compound 52:

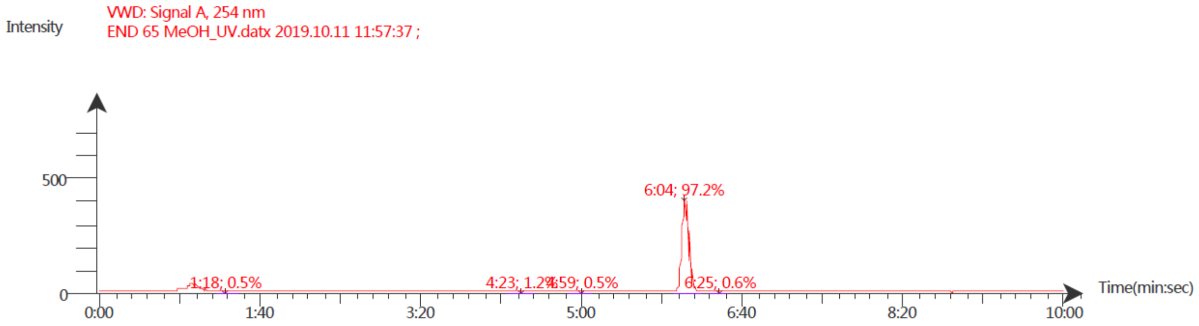

Time (Peak Maximum M:S/Minutes)	Maximum Intensity (c/s)	Time (Peak Centroid M:S/Minutes)	Peak Area	% Peak Area	Peak Resolution	Label
1:18.03	1.5E0	1:17.85	9.9E0	0.5	6.1	
4:22.83	3.1E0	4:22.63	2.5E1	1.2	7.6	
4:58.83	1.8E0	4:59.08	1.1E1	0.5	5.6	
6:04.03	4E2	6:04.22	2E3	97.2	4.8	
6:24.83	2.3E0	6:25.46	1.3E1	0.6	5.2	

Compound 53:

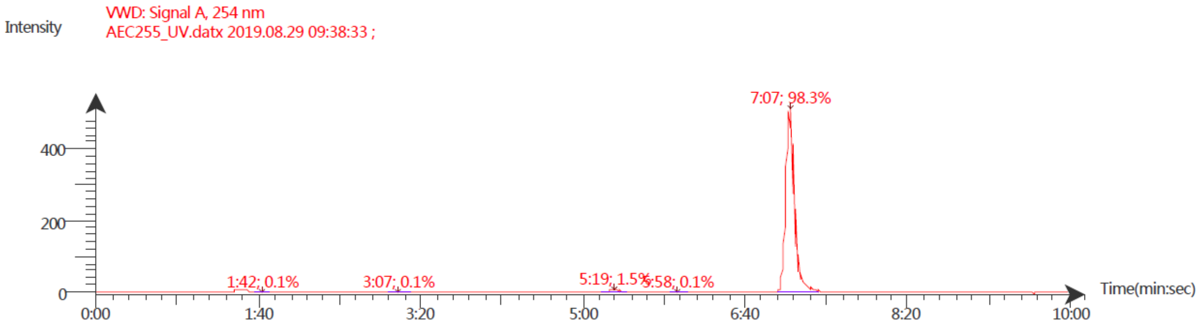

Time (Peak Maximum M:S/Minutes)	Maximum Intensity (c/s)	Time (Peak Centroid M:S/Minutes)	Peak Area	% Peak Area	Peak Resolution	Label
1:42.07	3.4E-1	1:42.77	1.8E0	0.1	5.7	
3:07.27	2.9E-1	3:06.33	2.2E0	0.1	8.1	
5:18.87	8.3E0	5:18.87	4.9E1	1.5	5.7	
5:57.67	2.7E-1	5:58.82	1.9E0	0.1	7.7	
7:06.87	5.2E2	7:07.70	3.2E3	98.3	5.6	

Compound 54:

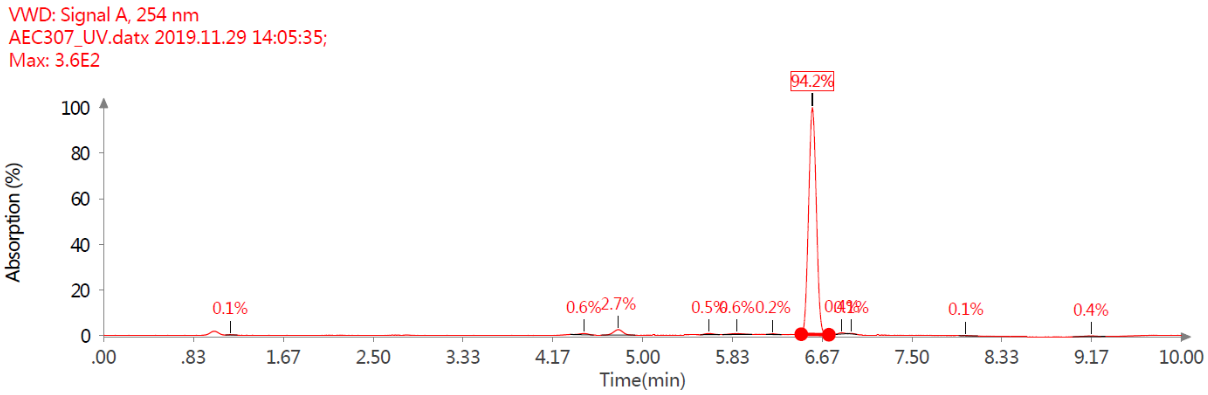

Time (Peak Maximum M:S/Minutes)	Maximum Absorption (mAU)	Time (Peak Centroid M:S/Minutes)	Peak Area	% Peak Area	Peak Resolution	Label
4.46	2E0	4.45	1.1E1	0.6	5.3	
4.78	8.6E0	4.77	5.2E1	2.7	5.6	
5.62	1.9E0	5.62	9.6E0	0.5	5.1	
5.87	1.4E0	5.87	1.2E1	0.6	8.0	
6.21	9.1E-1	6.21	3.9E0	0.2	4.2	
6.58	3.6E2	6.58	1.8E3	94.2	4.7	
6.85	1.8E0	6.85	6.7E0	0.4	3.8	
6.94	7.1E-1	6.94	2.3E0	0.1	4.8	
8.00	3.3E-1	8.00	1.8E0	0.1	10.0	
9.16	1E0	9.16	8.2E0	0.4	7.2	

Compound 55:

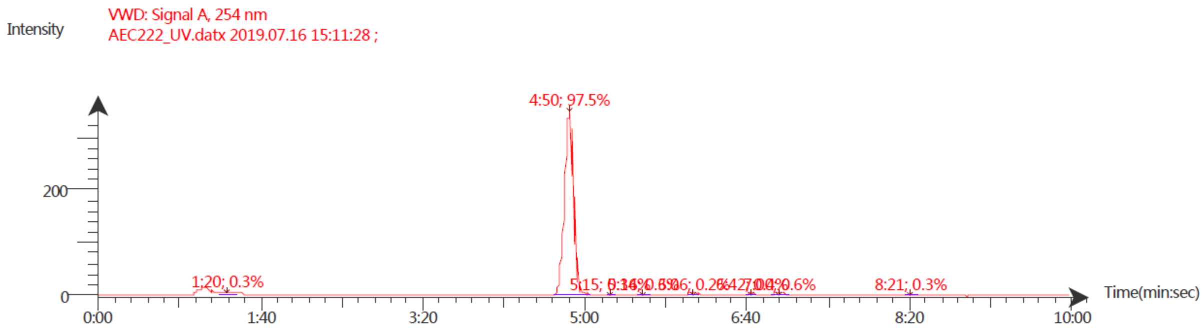

Compound 57:

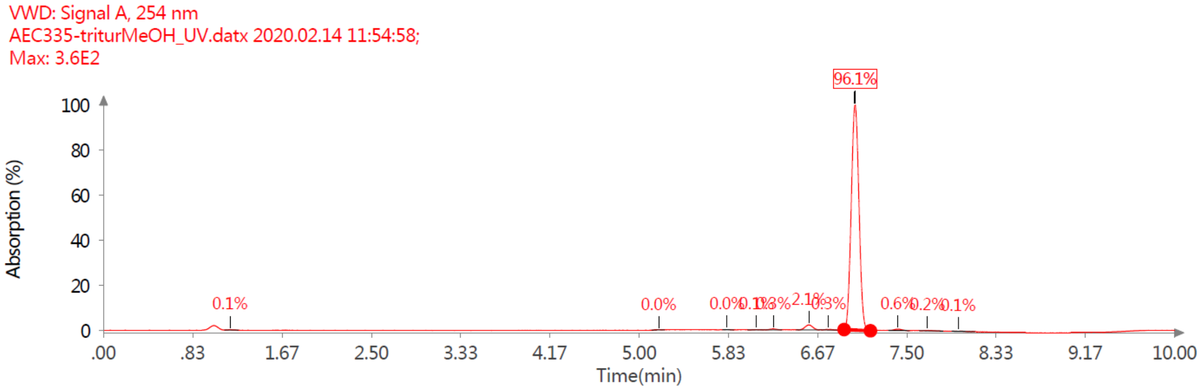

Time (Peak Maximum M:S/Minutes)	Maximum Absorption (mAU)	Time (Peak Centroid M:S/Minutes)	Peak Area	% Peak Area	Peak Resolution	Label
1.18	4.3E-1	1.18	1.6E0	0.1	3.7	
5.82	1.8E-1	5.82	7.1E-1	0.0	3.9	
6.10	3.4E-1	6.10	1.7E0	0.1	5.1	
6.25	1.2E0	6.25	5.3E0	0.3	4.4	
6.59	7.8E0	6.58	3.9E1	2.1	4.8	
6.77	9.8E-1	6.77	5E0	0.3	5.0	
7.02	3.5E2	7.02	1.8E3	96.1	4.8	
7.42	2.2E0	7.42	1.1E1	0.6	4.7	
7.69	7.6E-1	7.69	4.6E0	0.2	5.5	
7.98	3.2E-1	7.98	1.9E0	0.1	12.8	

Compound 58:

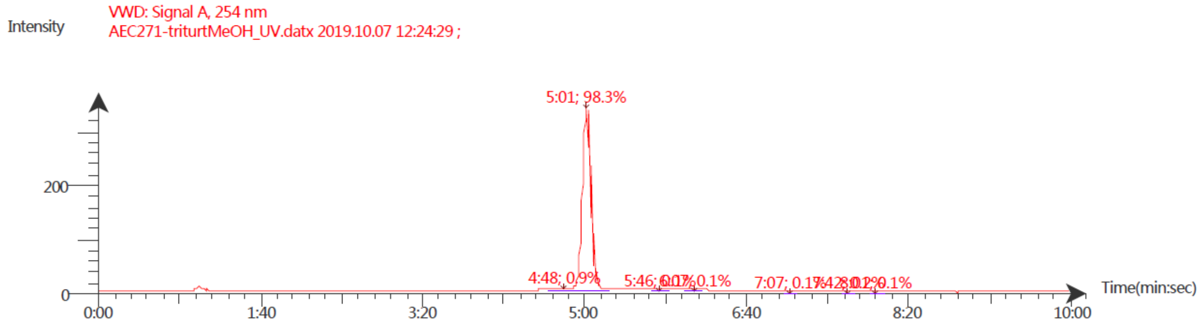

Time (Peak Maximum M:S/Minutes)	Maximum Intensity (c/s)	Time (Peak Centroid M:S/Minutes)	Peak Area	% Peak Area	Peak Resolution	Label
4:37.14	2.4E-2	4:34.04	9.9E-1	0.0	9.6	
4:37.14	1.7E-1	4:34.85	1.2E0	0.1	6.4	
4:48.34	2.2E0	4:46.24	1.8E1	0.9	5.6	
5:01.14	3.4E2	5:00.97	2.1E3	98.3	5.9	
5:45.94	5.8E-1	5:46.14	2.8E0	0.1	4.8	
6:07.14	4.8E-1	6:07.03	3E0	0.1	6.4	
7:06.74	3.5E-1	7:06.37	1.9E0	0.1	5.5	
7:06.74	3.7E-1	7:06.51	2.1E0	0.1	6.0	
7:41.94	9.3E-1	7:42.05	4.2E0	0.2	4.6	
8:00.74	4.6E-1	8:00.12	2.7E0	0.1	6.4	

Compound 59:

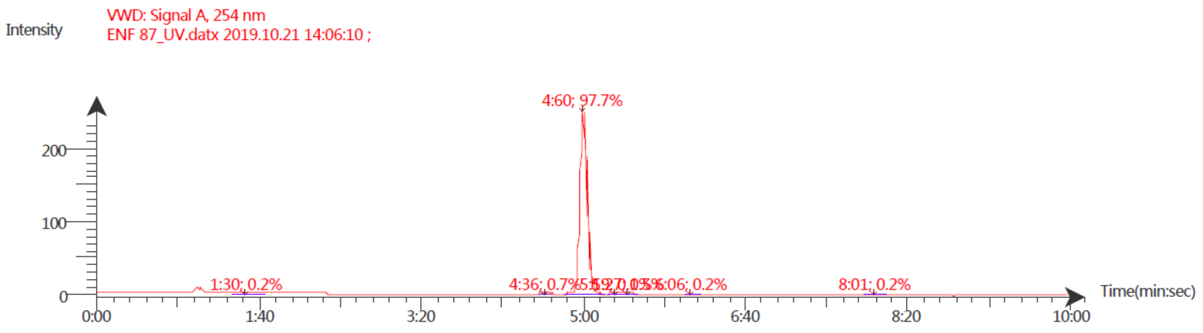

Time (Peak Maximum M:S/Minutes)	Maximum Intensity (c/s)	Time (Peak Centroid M:S/Minutes)	Peak Area	% Peak Area	Peak Resolution	Label
1:29.66	1.8E-1	1:31.71	2.3E0	0.2	13.6	
2:41.26	1E-1	2:39.41	1.1E0	0.1	19.6	
4:16.46	2.1E-1	4:16.97	1.2E0	0.1	5.4	
4:35.66	2E0	4:35.87	9.9E0	0.7	5.2	
4:59.66	2.5E2	4:59.69	1.4E3	97.7	5.1	
5:19.26	4.1E-1	5:18.88	2.1E0	0.1	4.7	
5:26.86	1.5E0	5:26.86	7.5E0	0.5	4.6	
6:06.06	5.4E-1	6:06.12	3.1E0	0.2	5.5	
6:52.86	2.6E-1	6:51.58	1.3E0	0.1	4.9	
8:01.26	4.8E-1	7:59.66	2.9E0	0.2	5.5	

Compound 60:

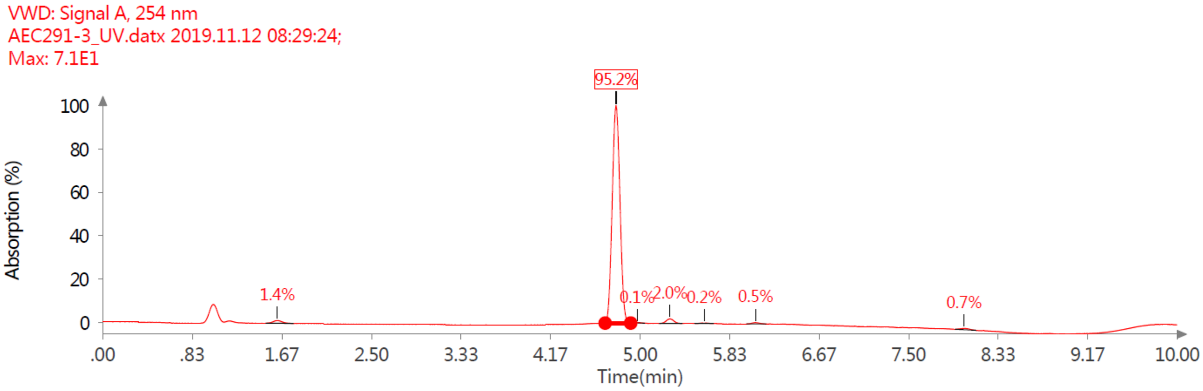

Time (Peak Maximum M:S/Minutes)	Maximum Absorption (mAU)	Time (Peak Centroid M:S/Minutes)	Peak Area	% Peak Area	Peak Resolution	Label
1.62	8.8E-1	1.62	5.1E0	1.4	5.4	
4.78	7.1E1	4.78	3.5E2	95.2	4.8	
4.98	5.8E-2	4.97	2.5E-1	0.1	6.4	
5.28	1.5E0	5.28	7.3E0	2.0	4.6	
5.60	1.7E-1	5.60	8.4E-1	0.2	4.8	
6.08	3.9E-1	6.07	1.9E0	0.5	4.8	
8.02	4.2E-1	8.02	2.5E0	0.7	6.1	

Compound 61:

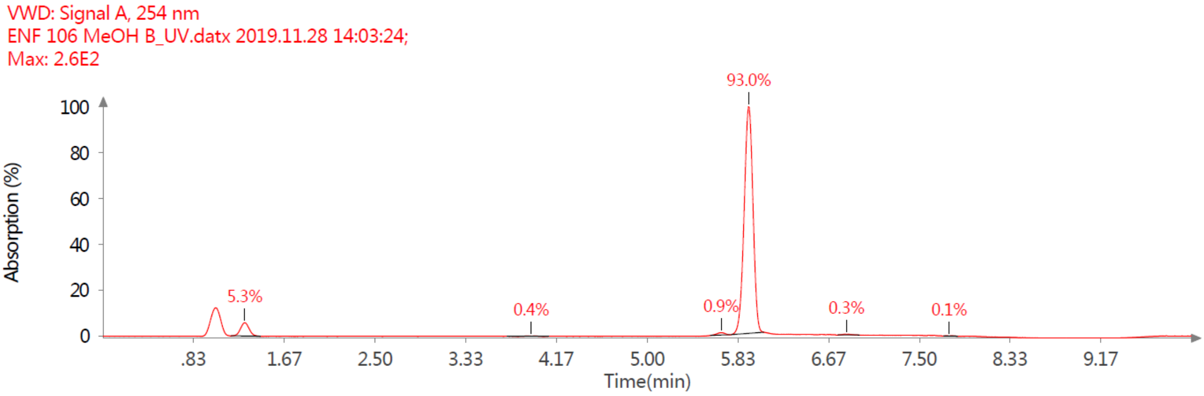

Time (Peak Maximum M:S/Minutes)	Maximum Absorption (mAU)	Time (Peak Centroid M:S/Minutes)	Peak Area	% Peak Area	Peak Resolution	Label
1.30	1.5E1	1.30	8.3E1	5.3	5.1	
3.94	7.1E-1	3.93	6.8E0	0.4	9.2	
5.68	2.7E0	5.68	1.4E1	0.9	5.0	
5.93	2.6E2	5.93	1.5E3	93.0	5.3	
6.83	8.4E-1	6.83	4.6E0	0.3	5.2	
7.78	2.9E-1	7.77	1.2E0	0.1	4.4	

Compound 62:

Chromatogram

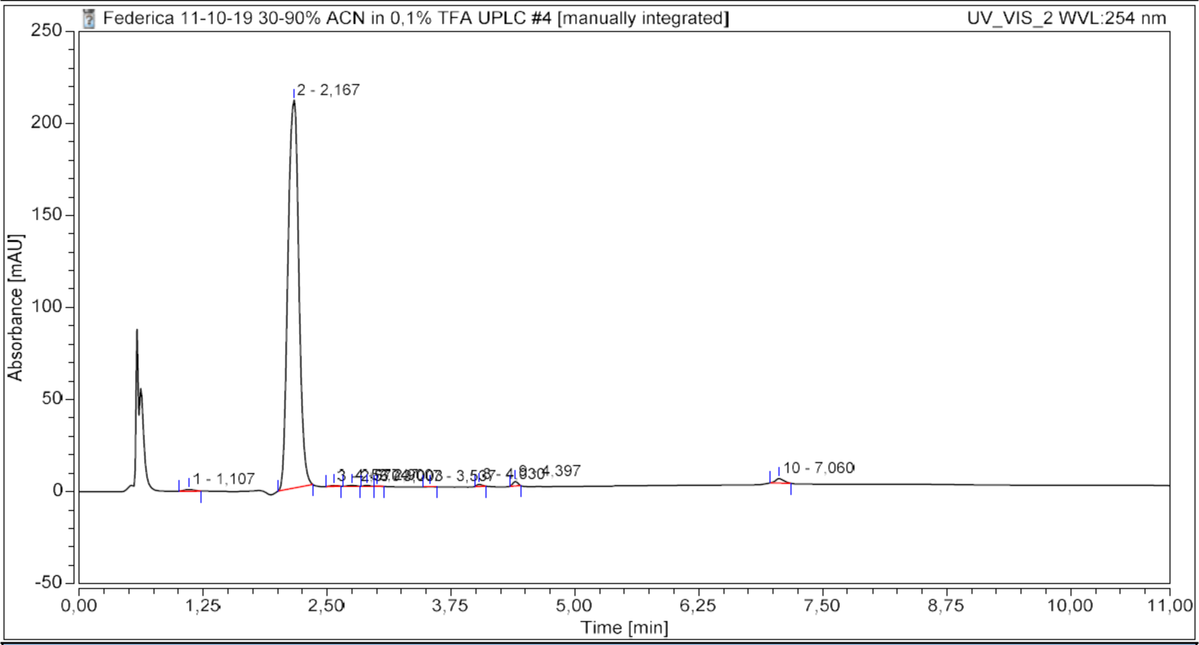

Integration Results

No.	Peak Name	Retention Time min	Area mAU*min	Height mAU	Relative Area %	Relative Height %	Amount n.a.
1		1,107	0,097	0,768	0,35	0,35	n.a.
2		2,167	26,837	210,738	97,72	96,37	n.a.
3		2,570	0,030	0,343	0,11	0,16	n.a.
4		2,747	0,024	0,303	0,09	0,14	n.a.
5		2,900	0,019	0,287	0,07	0,13	n.a.
6		3,003	0,006	0,090	0,02	0,04	n.a.
7		3,537	0,015	0,208	0,05	0,09	n.a.
8		4,030	0,068	1,097	0,25	0,50	n.a.
9		4,397	0,142	2,449	0,52	1,12	n.a.
10		7,060	0,226	2,393	0,82	1,09	n.a.
**Total:**			**27,464**	**218,675**	**100,00**	**100,00**	

Compound 63:

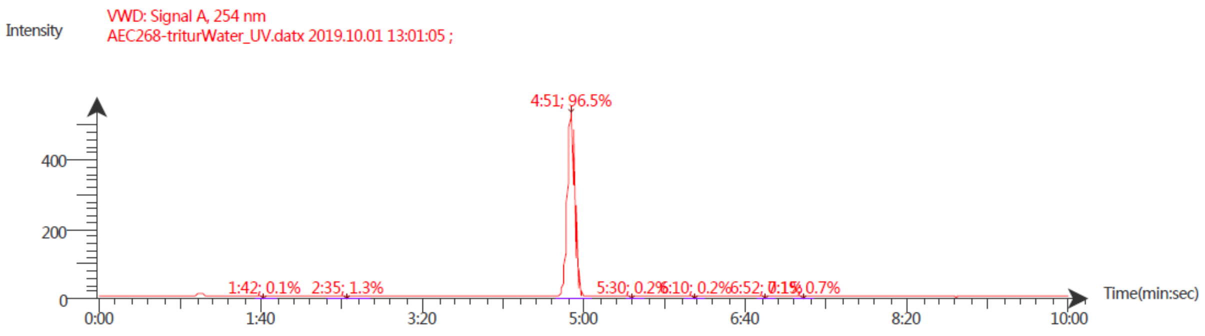

Time (Peak Maximum M:S/Minutes)	Maximum Intensity (c/s)	Time (Peak Centroid M:S/Minutes)	Peak Area	% Peak Area	Peak Resolution	Label
1:10.46	3.8E-1	1:10.80	1.4E0	0.0	3.7	
1:10.46	3.8E-1	1:10.80	1.4E0	0.0	3.7	
1:42.46	5.2E-1	1:41.96	3.6E0	0.1	7.0	
1:42.46	4.8E-1	1:41.98	3.1E0	0.1	6.6	
1:42.46	4.4E-1	1:42.09	2.7E0	0.1	6.2	
2:34.86	3.4E0	2:33.11	3.9E1	1.3	10.9	
4:27.66	4.9E-1	4:27.48	3.2E0	0.1	6.2	
4:27.66	5E-1	4:27.55	3.2E0	0.1	6.1	
4:27.66	4.6E-1	4:27.56	2.7E0	0.1	5.8	
4:27.66	4.8E-1	4:27.56	2.9E0	0.1	5.9	
4:27.66	5.1E-1	4:27.56	3.3E0	0.1	6.3	
4:51.26	5.4E2	4:51.42	2.9E3	96.5	5.2	
5:29.66	1.1E0	5:29.99	5.9E0	0.2	5.4	
6:10.06	9.8E-1	6:08.85	6.4E0	0.2	6.2	
6:52.46	7E-1	6:52.46	3.3E0	0.1	4.6	
7:14.86	4.4E0	7:14.82	2.2E1	0.7	4.7	
8:00.46	3.7E-1	7:59.81	2.1E0	0.1	6.1	
8:00.46	3.2E-1	8:00.12	1.6E0	0.1	5.4	

Compound 64:

Chromatogram

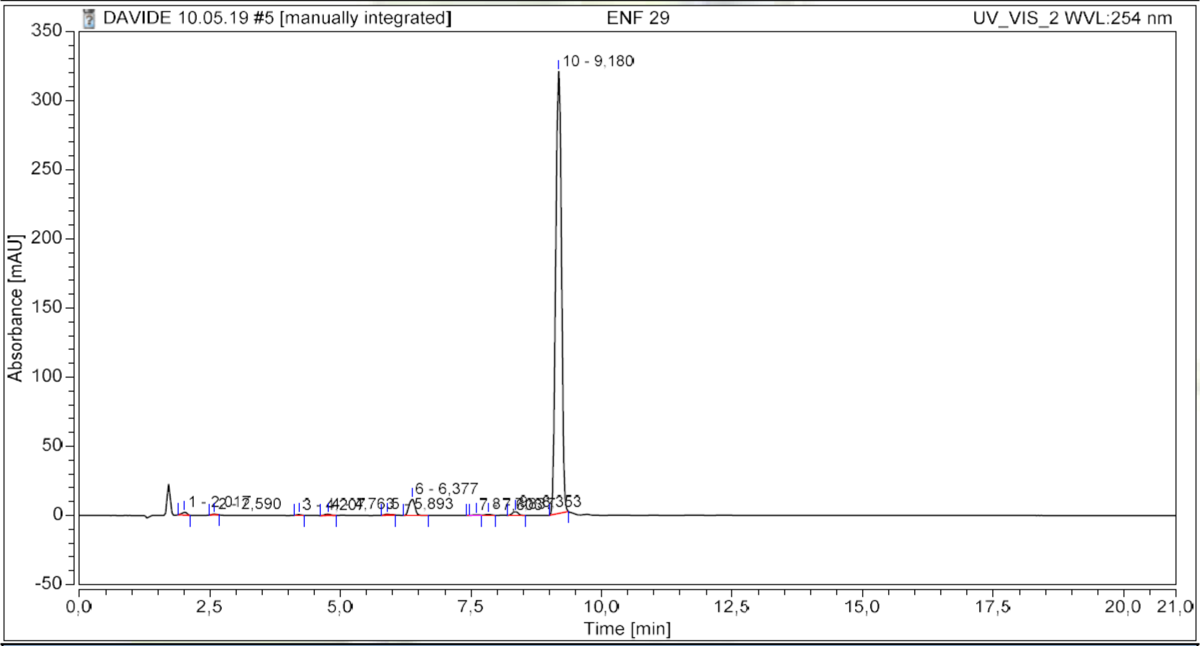

Integration Results

No.	Peak Name	Retention Time<br>min	Area<br>mAU*min	Height<br>mAU	Relative Area<br>%	Relative Height<br>%	Amount<br>n.a.
1		2,017	0,225	1,996	0,52	0,59	n.a.
2		2,590	0,065	0,612	0,15	0,18	n.a.
3		4,207	0,050	0,469	0,12	0,14	n.a.
4		4,763	0,107	0,860	0,25	0,25	n.a.
5		5,893	0,110	0,685	0,25	0,20	n.a.
6		6,377	1,514	11,813	3,48	3,48	n.a.
7		7,603	0,042	0,345	0,10	0,10	n.a.
8		7,837	0,130	0,681	0,30	0,20	n.a.
9		8,353	0,341	2,623	0,78	0,77	n.a.
10		9,180	40,902	319,465	94,06	94,09	n.a.
**Total:**			**43,485**	**339,548**	**100,00**	**100,00**	

Compound 65:

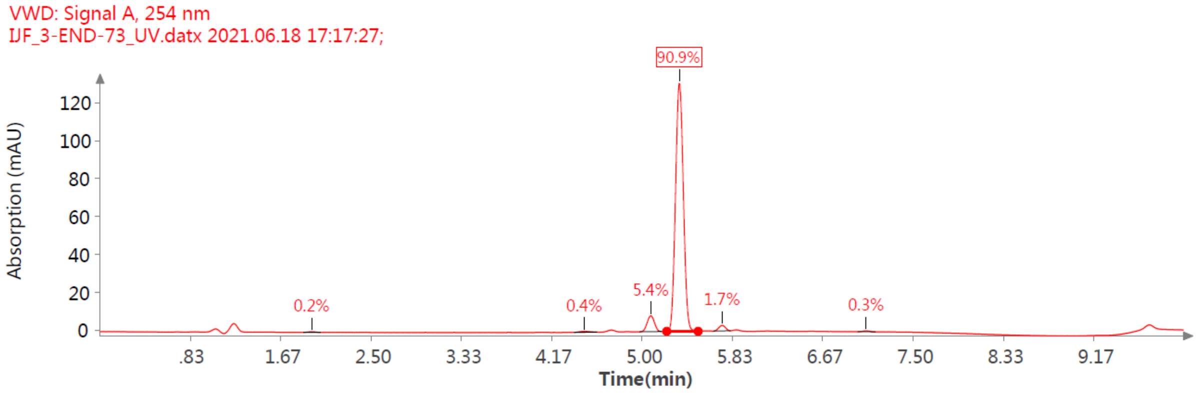

Time (Peak Maximum M:S/Minutes)	Maximum Absorption (mAU)	Time (Peak Centroid M:S/Minutes)	Peak Area	% Peak Area	Peak Resolution	Label
1.96	2.9E-1	1.95	1.3E0	0.2	4.5	
4.46	4.6E-1	4.47	2.8E0	0.4	6.3	
5.08	8.4E0	5.08	3.9E1	5.4	4.5	
5.34	1.3E2	5.35	6.6E2	90.9	4.8	
5.74	2.9E0	5.74	1.3E1	1.7	4.2	
7.07	4.4E-1	7.07	2E0	0.3	4.4	

Compound 66:

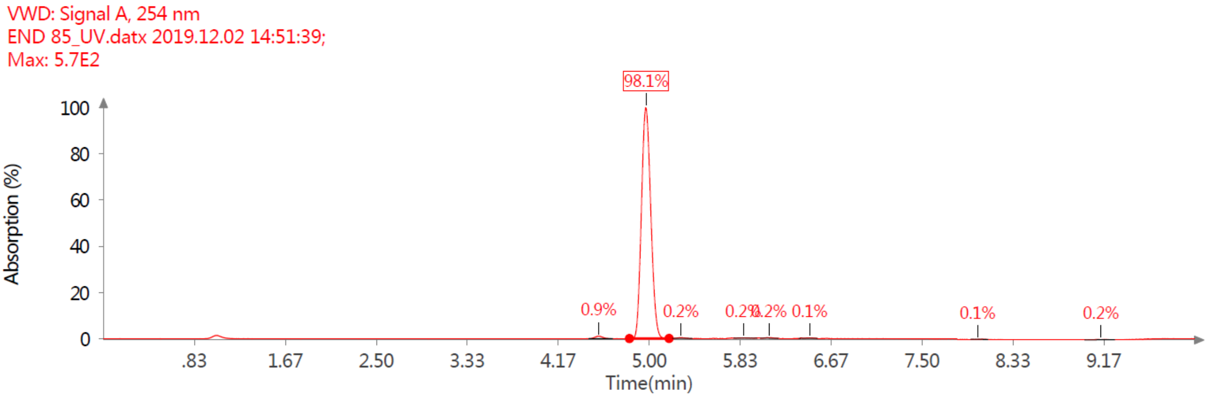

Time (Peak Maximum M:S/Minutes)	Maximum Absorption (mAU)	Time (Peak Centroid M:S/Minutes)	Peak Area	% Peak Area	Peak Resolution	Label
4.53	6E0	4.54	3E1	0.9	4.7	
4.97	5.7E2	4.97	3.4E3	98.1	5.6	
5.29	1.7E0	5.29	7.9E0	0.2	4.6	
5.86	8.1E-1	5.86	5.2E0	0.2	6.1	
6.09	1.6E0	6.10	7.6E0	0.2	4.6	
6.47	1.1E0	6.47	5.1E0	0.1	4.3	
8.01	3.6E-1	8.01	1.8E0	0.1	9.2	
9.14	8.9E-1	9.14	6.9E0	0.2	7.5	

Compound 67:

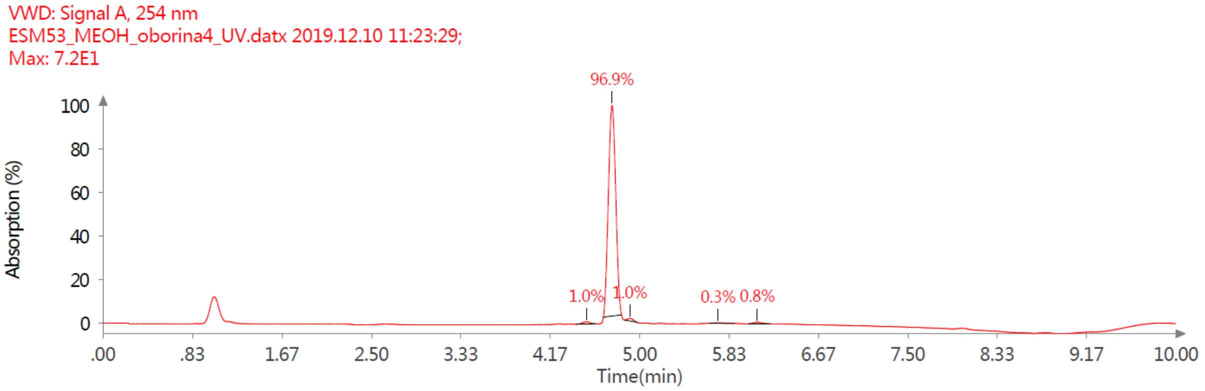

Time (Peak Maximum M:S/Minutes)	Maximum Absorption (mAU)	Time (Peak Centroid M:S/Minutes)	Peak Area	% Peak Area	Peak Resolution	Label
4.50	7.2E-1	4.50	3.5E0	1.0	4.7	
4.74	7E1	4.74	3.3E2	96.9	4.6	
4.91	8.5E-1	4.91	3.3E0	1.0	3.9	
5.73	1.7E-1	5.73	1.1E0	0.3	6.1	
6.10	4.8E-1	6.10	2.8E0	0.8	5.5	

Compound 68:

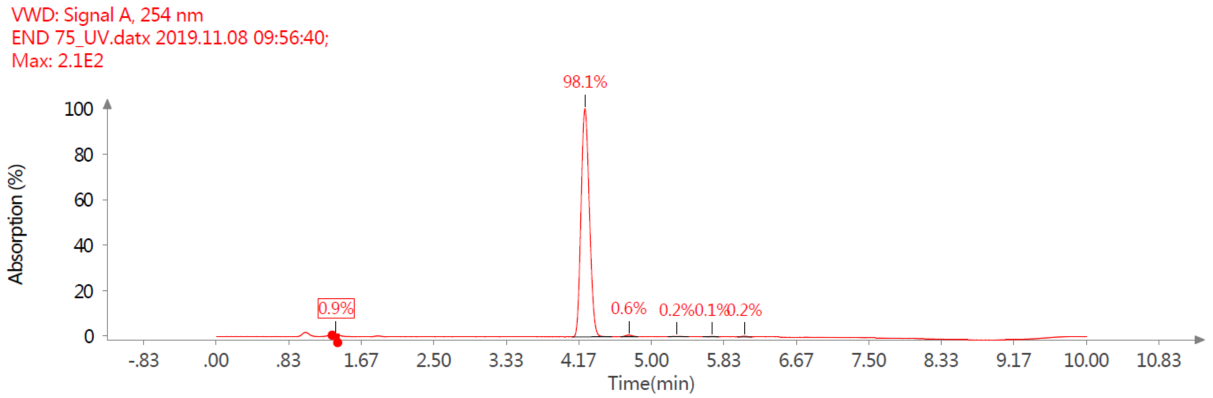

Time (Peak Maximum M:S/Minutes)	Maximum Absorption (mAU)	Time (Peak Centroid M:S/Minutes)	Peak Area	% Peak Area	Peak Resolution	Label
1.38	4.8E0	1.38	1.3E1	0.9	3.6	
4.24	2.1E2	4.24	1.4E3	98.1	6.3	
4.74	1.5E0	4.74	7.9E0	0.6	5.2	
5.29	3.3E-1	5.29	2.4E0	0.2	7.2	
5.70	2.8E-1	5.70	1.2E0	0.1	4.3	
6.07	5.5E-1	6.07	2.7E0	0.2	4.7	

Compound 69:

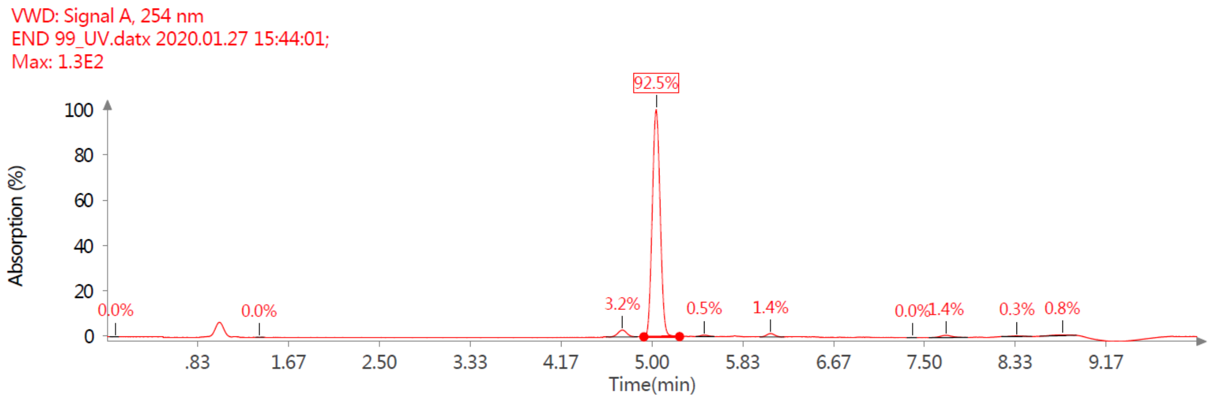

Time (Peak Maximum M:S/Minutes)	Maximum Absorption (mAU)	Time (Peak Centroid M:S/Minutes)	Peak Area	% Peak Area	Peak Resolution	Label
0.08	1.2E-2	0.08	3.4E-2	0.0	2.8	
1.40	1.7E-2	1.40	4.7E-2	0.0	2.9	
4.73	4E0	4.73	2.3E1	3.2	5.3	
5.04	1.3E2	5.04	6.7E2	92.5	4.8	
5.48	8.1E-1	5.48	3.8E0	0.5	4.6	
6.09	2E0	6.09	1E1	1.4	4.9	
7.39	3E-2	7.39	9.7E-2	0.0	3.2	
7.69	1.3E0	7.70	9.9E0	1.4	7.0	
8.35	2.8E-1	8.35	2E0	0.3	6.7	
8.75	4.7E-1	8.77	5.5E0	0.8	13.0	

Compound 70:

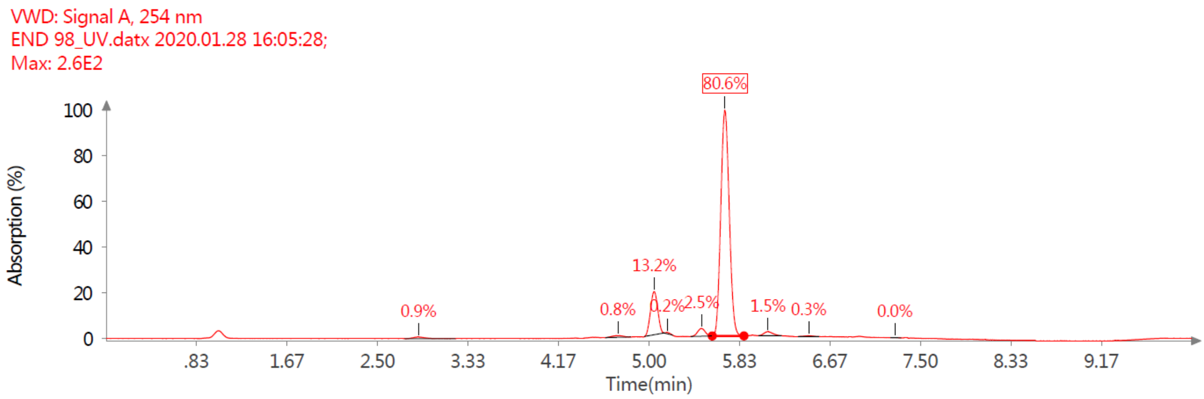

Time (Peak Maximum M:S/Minutes)	Maximum Absorption (mAU)	Time (Peak Centroid M:S/Minutes)	Peak Area	% Peak Area	Peak Resolution	Label
2.88	1.9E0	2.88	1.6E1	0.9	7.3	
4.72	1.9E0	4.71	1.3E1	0.8	7.1	
5.05	4.9E1	5.05	2.3E2	13.2	4.5	
5.17	1.5E0	5.17	4.2E0	0.2	4.4	
5.48	8.9E0	5.48	4.3E1	2.5	4.7	
5.70	2.6E2	5.70	1.4E3	80.6	5.1	
6.10	4.5E0	6.10	2.6E1	1.5	5.6	
6.47	9E-1	6.47	4.6E0	0.3	4.9	
7.26	1.6E-1	7.26	5.8E-1	0.0	5.6	

Compound 71:

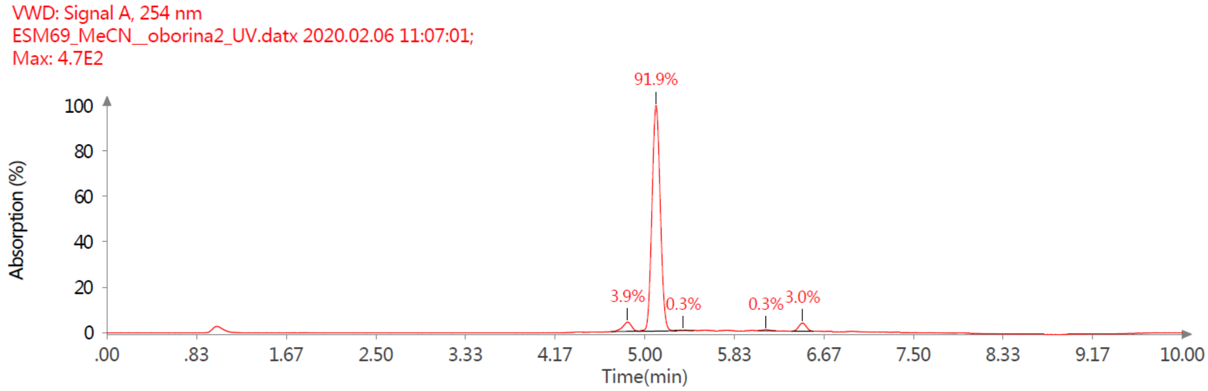

Time (Peak Maximum M:S/Minutes)	Maximum Absorption (mAU)	Time (Peak Centroid M:S/Minutes)	Peak Area	% Peak Area	Peak Resolution	Label
4.84	1.9E1	4.84	1.1E2	3.9	5.2	
5.10	4.6E2	5.11	2.5E3	91.9	5.0	
5.36	1.2E0	5.36	7.2E0	0.3	6.4	
6.13	2E0	6.13	8.9E0	0.3	4.4	
6.47	1.7E1	6.47	8E1	3.0	4.7	

Compound 72:

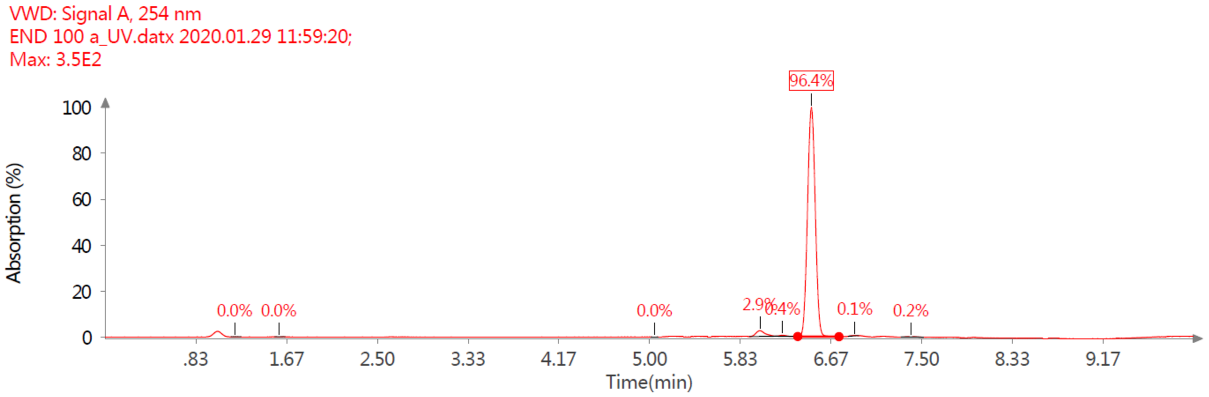

Time (Peak Maximum M:S/Minutes)	Maximum Absorption (mAU)	Time (Peak Centroid M:S/Minutes)	Peak Area	% Peak Area	Peak Resolution	Label
1.19	6E-2	1.19	1.8E-1	0.0	3.0	
1.59	9.2E-2	1.59	3.3E-1	0.0	3.5	
5.05	3.6E-2	5.05	9.9E-2	0.0	2.8	
6.01	8.9E0	6.01	5.2E1	2.9	5.3	
6.22	1.6E0	6.22	6.5E0	0.4	4.0	
6.49	3.5E2	6.49	1.7E3	96.4	4.7	
6.89	5E-1	6.89	2E0	0.1	5.2	
7.40	5.9E-1	7.40	3.5E0	0.2	5.6	

Compound 73:

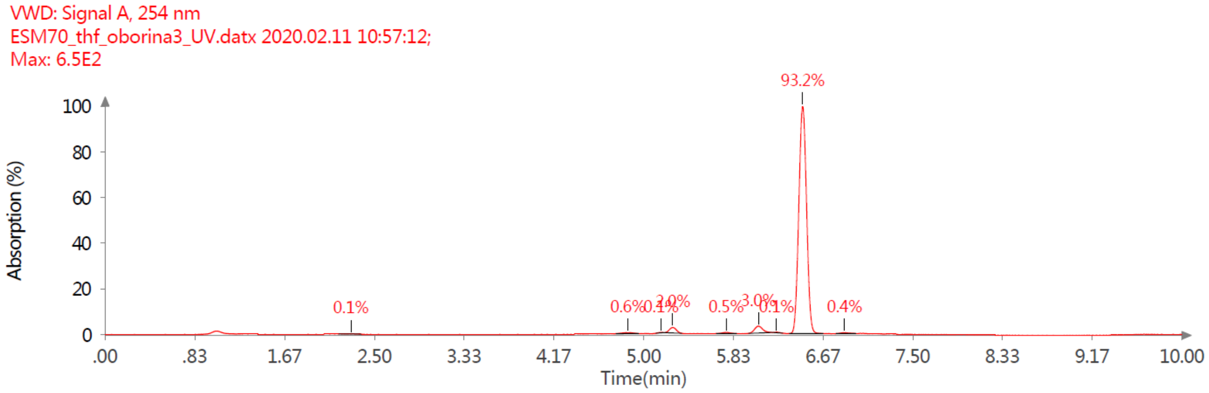

Time (Peak Maximum M:S/Minutes)	Maximum Absorption (mAU)	Time (Peak Centroid M:S/Minutes)	Peak Area	% Peak Area	Peak Resolution	Label
2.28	5.2E-1	2.28	3.4E0	0.1	6.8	
4.85	2.8E0	4.85	2E1	0.6	7.4	
5.16	1.1E0	5.16	3E0	0.1	4.0	
5.27	1.6E1	5.27	7E1	2.0	4.3	
5.77	3.1E0	5.77	1.6E1	0.5	4.9	
6.07	2E1	6.07	1E2	3.0	4.9	
6.23	1.2E0	6.24	4.2E0	0.1	5.6	
6.47	6.5E2	6.48	3.2E3	93.2	4.7	
6.86	2.5E0	6.87	1.3E1	0.4	5.1	
